# CavitOmiX Drug Discovery: Engineering Antivirals with Enhanced Spectrum and Reduced Side Effects for Arboviral Diseases

**DOI:** 10.3390/v16081186

**Published:** 2024-07-24

**Authors:** Lena Parigger, Andreas Krassnigg, Michael Hetmann, Anna Hofmann, Karl Gruber, Georg Steinkellner, Christian C. Gruber

**Affiliations:** 1 Innophore GmbH, 8010 Graz, Austria; 2 Institute of Molecular Biosciences, University of Graz, 8010 Graz, Austria

**Keywords:** arbovirus, antivirals, drug discovery, drug repurposing, drug design, side effects, virus variants, chikungunya, small-molecule drugs, CavitOmiX

## Abstract

Advancing climate change increases the risk of future infectious disease outbreaks, particularly of zoonotic diseases, by affecting the abundance and spread of viral vectors. Concerningly, there are currently no approved drugs for some relevant diseases, such as the arboviral diseases chikungunya, dengue or zika. The development of novel inhibitors takes 10–15 years to reach the market and faces critical challenges in preclinical and clinical trials, with approximately 30% of trials failing due to side effects. As an early response to emerging infectious diseases, CavitOmiX allows for a rapid computational screening of databases containing 3D point-clouds representing binding sites of approved drugs to identify candidates for off-label use. This process, known as drug repurposing, reduces the time and cost of regulatory approval. Here, we present potential approved drug candidates for off-label use, targeting the ADP-ribose binding site of *Alphavirus chikungunya* non-structural protein 3. Additionally, we demonstrate a novel in silico drug design approach, considering potential side effects at the earliest stages of drug development. We use a genetic algorithm to iteratively refine potential inhibitors for (i) reduced off-target activity and (ii) improved binding to different viral variants or across related viral species, to provide broad-spectrum and safe antivirals for the future.

## 1. Introduction

Arthropod-borne viruses (arboviruses) are a major global health challenge, causing serious diseases in humans and animals [1,2,3]. Climate change and the associated spread of arthropod vectors across the world increases the risk of future outbreaks and ultimately could lead to pandemic behavior of transmitted diseases [4,5]. In this sense, viruses such as *Alphavirus chikungunya* (CHIKV), *Dengue virus* and *Zika virus* are of particular concern due to their high morbidity and mortality rates [6]. They are transmitted via the mosquitoes *Aedes aegypti* and *Aedes albopictus*, which have both experienced significant changes in geographic distribution due to progressing climate change [7].

While live attenuated and nanoparticle-based vaccines are available to protect against a number of arboviral diseases [8], no approved antivirals for arbovirus infections currently exist [9] that can be used for the immediate treatment of active infections or immunocompromised patients who cannot be protected by vaccines. By April 2024, there were two clinical studies concerning small-molecule inhibitors of arboviruses listed at https://clinicaltrials.gov/ (accessed on 25 June 2024), namely NCT05466240 and NCT04906980, which, however, have been terminated due to deprioritization by sponsors and the impact of COVID. These studies were targeting *Dengue virus* infections by investigating molecules which inhibit NS5 [10] and the interaction of NS3-NS4B [11], respectively. No active clinical studies on small-molecule inhibitors against arboviruses existed at the time this paper was submitted.

Typically, the development of novel inhibitors takes 10–15 years and USD $1–2 billion per drug to reach the market and comes with significant risks of failure during preclinical and clinical trials [12]. Sun et al. states that 9 out of 10 clinical trials are not successful, 40–50% of which fail due to lack of efficiency and 30% fail due to toxicity or side effects [13,14]. The off-label use of already approved drugs, so-called drug repurposing, is an effective alternative to the development of novel therapeutics, as it allows shortened trials and thus leads to significant reductions in time and cost until market release. However, for novel drug development or refinement, these numbers stress the need to consider potential side effects very early in drug discovery. Innophore and NVIDIA recently provided the most comprehensive dataset of human protein structures with thousands of potential drug-binding sites for public use, providing a valuable resource for identifying potential off-target sites of lead drug-candidates [15]. This allows for the in silico screening of the human proteome for potential side effects, which should be considered in drug development and discovery at an early stage.

Besides the consideration of potential off-target activity, the emergence of viral variants must be observed and included in the development of effective, broad-spectrum antivirals. Past pandemics demonstrated the need for genetic surveillance of viral variants, and to consider the mutational behavior in the development of vaccines and antivirals [16,17]. Public genome databases like GISAID [18] or NCBI [19] allow for such monitoring, where full or partial genome sequences can be submitted and retrieved.

Here, we present our efforts to combat CHIKV and related viruses, presenting potential inhibitors targeting the non-structural protein 3 (nsP3). CHIKV is an alphavirus of the family *Togaviridae* that, upon infection of humans, causes a high fever, headache, rash and, in most cases, severe joint pain, with long-term arthralgia and arthritis in ~30% of cases [20]. nsP3 plays multiple essential roles during viral genome replication and transcription, which makes it a promising drug target against CHIKV [21]. It is assumed that compounds targeting the ADP-ribose binding site of nsP3 are interfering already in the early stages of CHIKV replication; however, concerns about binding to human macrodomains involved in ADP-ribosylation are raised [22]. This makes nsP3 an interesting target for CavitOmiX drug discovery, which is especially suitable for identifying and refining candidate inhibitors against targets with a higher potential to induce side effects.

In this regard, we aim to discover small molecule inhibitors to combat arboviral diseases using Innophore’s DrugSolver CavitOmiX, a method for rapidly screening databases of 3D point-clouds representing the binding sites of already approved drugs, for which the toxicity profiles have been studied and reported. This presents an efficient first step for drug discovery as it allows for the identification of already approved drug candidates for off-label use, which aims to reduce the time and cost of regulatory approval.

For the development of novel therapeutics, we employed a genetic algorithm to iteratively refine lead candidates for (i) reduced binding to potential off-target sites, using the proteome resource provided by Innophore and NVIDIA [15] and (ii) improved binding to different viral variants identified via continuous mutation monitoring. This approach further allows for a refinement of small molecules towards improved binding across related viral species, such as the medically relevant alphaviruses *Eastern equine encephalitis virus* (EEEV) or *Sindbis virus* (SINV), and thus facilitates the development of broad-spectrum and safe antivirals for the future.

## 2. Materials and Methods

**Figure 1:**
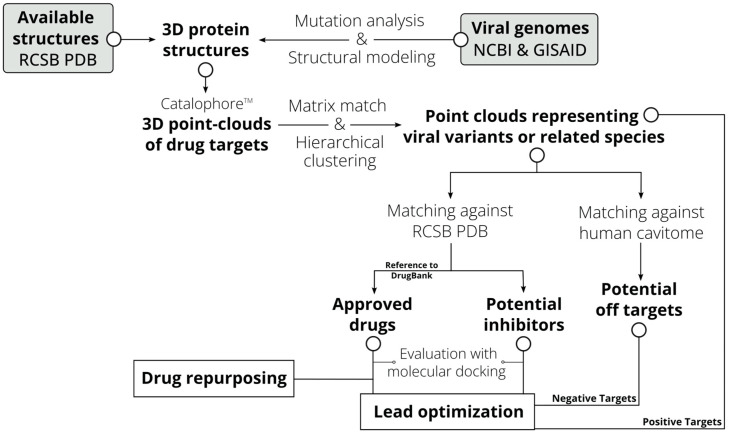
Workflow of the CavitOmiX drug discovery approach.

Our workflow efficiently combined several tools from modern bioinformatics and drug discovery. An overview of the workflow is given in Figure 1. All elements are described in detail below.

### 2.1. Mutation Analysis of Viral Genome Sequences

To identify all mutations occurring in viral samples sequenced to date, 8785 and 9909 CHIKV genome sequences were downloaded from GISAID [18] and NCBI [23] in May 2024, respectively. Genome sequences were translated for all six reading frames and aligned to the CHIKV nsP3 protein sequence, which was extracted from the CHIKV reference genome NC_004162, in Biopython 1.79 [24], employing the SeqIO module and blastp [25]. With this, 3437 and 2579 nsP3 sequences were extracted from genomes retrieved from GISAID and NCBI, respectively. Together, 502 unique nsP3 protein sequences were identified and mutations in these sequences were parsed by aligning each variant’s protein sequence to the reference protein sequence using the Bio.pairwise2 module. To accurately map mutations to their positions within the protein sequence, only high-quality amino-acid exchanges were considered for subsequent analysis. These exchanges excluded those adjacent to deletions, insertions or ambiguous residues (“X”). Additionally, the surrounding region of 10 residues on either side of the mutation must be largely intact, allowing no more than 50% mismatches within this span if deletions or insertions are present. Only mutations within positions 1–160 were considered, since the region apart from the ADP-ribose binding domain have not yet been structurally solved. Finally, a set of 100 unique nsP3 variants were identified.

### 2.2. Generation of Structural Models and Point-Cloud Representations of Druggable Sites

Three-dimensional structures of the reference nsP3/NS3 proteins from CHIKV, EEEV (NCBI accession number: NP_740651.1) and SINV (NP_740672.1) were generated by homology modeling using the CavitOmiX DrugSolver Platform employing Yasara [26], with 6 PSI-BLAST iterations, a maximal expect value of 0.5, five templates to consider, a maximum of five alignment variations per template and 50 conformations tested per loop. Terminal residues that protruded beyond the alignment were omitted from modeling. Homology models of the CHIKV nsP3 protein variants, which resulted from the mutational analysis, were generated by using the homology model of the reference nsP3 protein as a modeling template. The drug target cavities, represented as 3D point-clouds, of CHIKV, EEEV, SINV and the CHIKV nsP3 protein variants were created with the Catalophore^TM^ technology [27,28], employing the LIGSITE algorithm [29] with a cutoff value of 5. Different physico-chemical properties, i.e., hydrophobicity, desolvation or electrostatics potential, were calculated for each point in the cavity point-clouds. By using homology models instead of, where available, raw structural data obtained from the Protein Data Bank [30] (PDB) as a basis for cavity calculations, we ensured that observed structural differences in the ADP-ribose binding sites of nsP3 variants were due to the mutations themselves and not due to discrepancies arising from different experimental conditions or different methodologies.

### 2.3. Comparison and Clustering of Binding Site Cavities

The identification of candidates for drug repurposing or refinement was achieved by matching the viral drug-target cavity against a precomputed set of 321,407 ligand-binding cavities calculated from 3D structures deposited in the PDB. Potential off-targets were found by matching against a set of 437,297 human binding sites, represented as point-clouds, which were calculated using human protein structures provided by Innophore and NVIDIA [15].

Point-cloud matching was achieved by superimposing and optimizing the alignment of the cavities so that the matching score was minimized. The matching score was built from a series of property scores (e.g., hydrophobicity or desolvation potential, shape) with adjustable weights. A lower matching score relates to higher cavity similarity (identical cavities show a matching score of 0). The details of point-cloud matching can be found in the “Catalophore Halo analysis” section of the Materials and Methods in our previous publication [31]. Cavity matching results were filtered by the extent of point-cloud overlaps between the query and target cavity (min. 50% or ligand-bound and 20% for empty cavities) and a maximum matching score of 0.1. The cloud overlap gives the percentage of cloud volume that matches between two point-clouds and is reported separately for the query and target cavity.

Representatives from a set of Catalophore^TM^ cavities were selected by performing a matrix match, matching each possible cavity pair, and subsequent hierarchical clustering based on the matching scores with the Python module scipy [32].

### 2.4. Selection of Small-Molecule Candidates for Refinement

The drug candidates considered for optimization were identified via (i) cavity matching of the drug target cavities (virus mutants or CHIKV, EEEV and SINV) against the ligand-binding cavities of all PDB structures available (accessed in February, 2024) and subsequent molecular docking of the respective ligands into the query cavity, and (ii) via docking of 10,693 drug-like molecules obtained from ChEMBL [33] into the ADP-ribose binding site of the homology model of the reference CHIKV nsP3. Details on the docking and cavity matching results for the input drug candidates are available in the Supplementary Information (Tables S1 and S2). Ligands were collected and treated in SMILES [34] format and respective 3D structures were built within the Catalophore^TM^ platform, employing YASARA, prior to docking. Ligands evaluated via molecular docking were selected by their binding energy of the lowest-energy predicted binding-mode.

Molecular docking was performed using Yasara within the Catalophore DrugSolver Platform, allowing a docking box extension of 2 Å around the cavity. Docking runs were performed with VINA, employing the amber03 force field, and repeated five times. Results were clustered using an RMSD of 5 Å.

### 2.5. Refinement of Molecules with a Genetic Algorithm

Once a promising set of lead candidates for potential binders in the target cavities has been found, a standard step is to perform lead optimization on these candidate molecules. In particular, beneficial properties are sought to be enhanced and negative properties avoided.

In our case, we employed this kind of optimization algorithm in order to increase the binding affinity of candidate molecules to the target cavities of our investigation. This included several positive binding targets due to our attempted coverage of viral variants. At the same time, a set of relevant negative targets was chosen, the binding to which we tried to minimize in order to avoid possible side effects.

In addition to these main objectives of the optimization, we imposed some additional criteria such that the resulting molecules were more likely to have reasonable properties in the context of drug design. For example, a quantitative estimate of drug-likeness (QED) [35], string length, ring sizes and less important estimators were included as further fitness objectives. While synthesizability is generally a factor considered in the validation and selection processes within the optimization algorithm, it is essential to individually evaluate the resulting candidate compounds for this characteristic.

Overall, this led to a multi-objective optimization problem, which we treated in the standard scalarization approach [36] by summing all objective functions with suitable weights in order to arrive at a meaningful and effective total fitness. Each candidate molecule was represented in the algorithm by its SMILES string [34], which is a standard strategy for molecule description. At the same time, SMILES strings are a powerful genetic code due to their combination of stability and potential innovation under the genetic operations of mutations as well as crossovers.

### 2.6. Visualizations

Data were visualized using the Matplotlib [37] and Seaborn [38] package in Python. Structural representations were created with Pymol 3.0.2 (Schrodinger Inc., New York, NY 10036, USA) Open Source, https://pymol.org (accessed on 14 May 2024) and the CavitOmiX plugin (Innophore, https://innophore.com/cavitomix (accessed on 22 June 2024)).

## 3. Results

In the following sections, we describe potential repurposable drugs targeting CHIKV nsP3, which were identified using the CavitOmix drug discovery approach. We further highlight potential human off-targets and show how viral mutations in the ADP-ribose binding site might influence drug binding. Finally, we demonstrate the early results of our efforts to iteratively refine drug candidates for (i) enhanced binding to viral variants or related virus species and (ii) reduced binding to potential human off-target sites.

### 3.1. Approved Drugs for Off-Label Use against CHIKV Disease

As the first step in drug discovery research, it is recommended to search for already approved or investigated therapeutics, which might be suitable for off-label use against the target in question. This approach saves time and resources in bringing a candidate molecule to market. CavitOmiX allows for a rapid comparison of a viral drug binding site to the binding sites of approved drugs, which have been calculated from structures deposited in the PDB, and it has previously been successfully applied to search for repurposable drugs against SARS-CoV-2 [39].

Using the Catalophore^TM^ cavity of the ADP-ribose binding site in CHIKV nsP3 as a search query, more than 200 similar binding sites were identified, 37 of which were listed as approved drugs in ChEMBL [33] or had high structural similarity to approved drugs. The binding mode and affinity of these to CHIKV nsP3 were evaluated via molecular docking. Finally, Remdesivir and Naproxen were identified as the most promising candidates for off-label use in terms of binding site similarity, docking mode and predicted binding energy. Sunitinib also has a significantly similar binding site to CHIKV nsP3; however, its binding affinity to the original human drug target was predicted to be significantly higher than its affinity to nsP3, which might lead to serious side effects.

**Figure 2:**
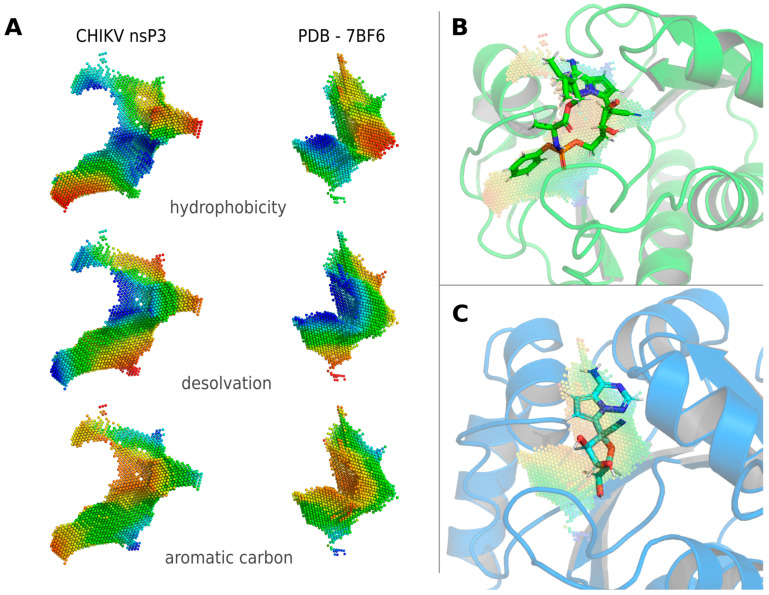
Identification of Remdesivir for off-label use against CHIKV via Catalophore^TM^ matching. (**A**) Physico-chemical property comparison of the ADP binding site in CHIKV nsP3 and GS-441524 binding site in SARS-CoV-2 papain-like protease (PDB ID: 7BF6). (**B**) Binding mode of Remdesivir to CHIKV nsP3 identified via molecular docking. (**C**) Binding mode of GS-441524 to SARS-CoV-2 papain-like protease as in PDB 7BF6. CHIKV nsP3 and SARS-CoV-2 papain-like protease show the same fold (TM-score = 0.77) [41,42] and a sequence identity of 25%. Point-clouds represent property gradients and are colored from red to blue, indicating decreasing hydrophobicity, increasing solvent accessibility (decreasing desolvation) and decreasing density of aromatic carbon atoms, respectively.

The Remdesivir metabolite GS-441524 (CHEMBL2016757), an experimental drug targeting severe acute respiratory syndrome coronavirus 2 (SARS-CoV-2) and Ebola infections [40], was one of the top hits in the cavity comparison. Its binding site in SARS-CoV-2 papain-like protease (PDB ID: 7BF6) shows a strong similarity in terms of its hydrophobicity, desolvation potential and the distribution of aromatic carbon atoms to the ADP-ribose binding site in CHIKV nsP3 (Figure 2A). Molecular docking of Remdesivir into CHIKV nsP3 shows a similar, yet, due to sterical reasons, slightly different binding mode compared to the binding behavior of GS-441524 in 7BF6 (Figure 2B,C). The binding energies of GS-441524 and Remdesivir to CHIKV nsP3 resulting from molecular docking are −7.75 and −8.73 kcal/mol, whereas the binding energy for 7BF6, determined via redocking into the structure, is −7.30 and −7.73 kcal/mol, respectively, indicating a higher binding affinity, especially for Remdesivir, to CHIKV nsP3 than to 7BF6. The binding mode of GS-441524 to 7BF6, which was determined in the redocking, was identical to the binding mode in 7BF6 stored in the PDB.

**Figure 3:**
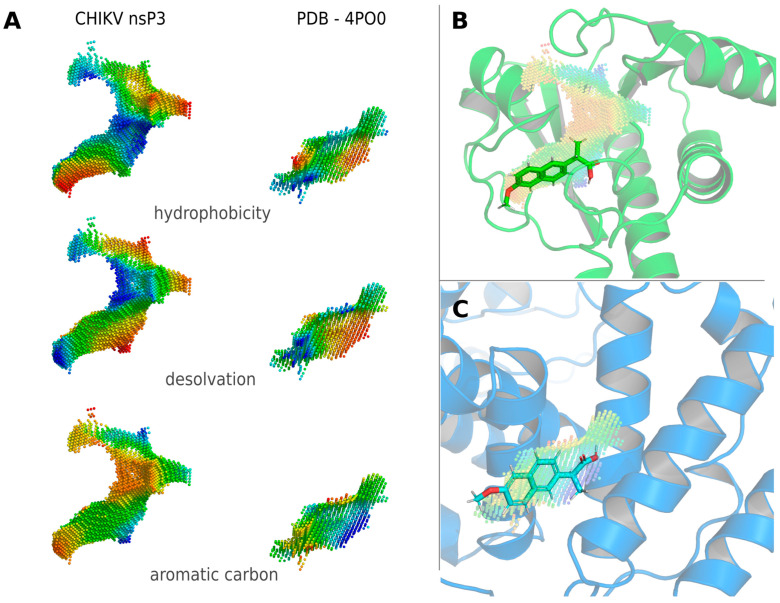
Identification of Naproxen for off-label use against CHIKV via Catalophore^TM^ matching. (**A**) Physico-chemical property comparison of the ADP binding site in CHIKV nsP3 and Naproxen binding site in Leporine Serum Albumin (PDB ID: 4PO0). (**B**) Binding mode of Naproxen to CHIKV nsP3 identified via molecular docking. (**C**) Binding mode of Naproxen to Leporine Serum Albumin as in PDB 4PO0. Leporine Serum Albumin shows no structural similarity to CHIKV nsP3 (TM-score = 0.13) and no significant sequence identity. Point-clouds represent property gradients and are colored from red to blue, indicating decreasing hydrophobicity, increasing solvent accessibility (decreasing desolvation) and decreasing density of aromatic carbon atoms, respectively.

The binding site of the anti-inflammatory arthritis medication Naproxen (CHEMBL154) to Leporine Serum Albumin (PDB ID: 4PO0 [43]) is also particularly similar to the ADP-ribose binding site of CHIKV nsP3 (Figure 3) Molecular docking of Naproxen to nsP3 and 4PO0 reveals binding energies of −8.33 and −7.33 kcal/mol, respectively.

Additionally, we found that the binding site of Sunitinib (CHEMBL1567), a therapeutic used to treat renal cancer, to its target (vascular endothelial growth factor receptor 2) has a strong similarity to the ADP-ribose binding site of CHIKV nsP3. The molecular docking of Sunitinib revealed a very similar binding mode related to the alignment of binding sites, with predicted binding energies of −9.04 kcal/mol to CHIKV nsP3 and −10.25 kcal/mol to its original target, 4AGD [44]. The tighter binding to human vascular endothelial growth factor receptor 2 than to the viral target is a concern, and side-effects resulting from the original purpose of this medication are expected and must be considered. A visual comparison of binding sites and the ligand binding mode is available in the Supplementary Information (Figure S1). Vascular endothelial growth factor receptor 2 and CHIKV nsP3 do not show a similar protein fold (TM-score = 0.23) or amino-acid sequence.

Drug repurposing using CavitOmiX is a promising way to identify already approved drugs for off-label use. By reducing the search space to the most important part, the drug binding sites, the available pool of hits reaches even beyond similar sequences or similar protein folds. The development of novel inhibitors requires a more thorough investigation and is assumed to be most effective if different viral variants and potential side-effects are considered from the very beginning of the drug discovery and development process.

### 3.2. Identification of Potential Human Off-Target Sites for nsP3 Inhibitors

**Figure 4:**
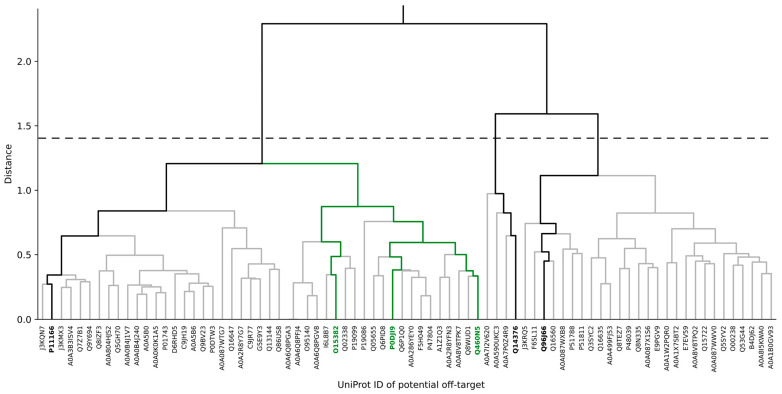
Clustering of potential human off-target sites for CHIKV-nsP3 targeting therapeutics. Hierarchical clustering was performed based on the matching score of a set of human binding-site cavities to each other via a Catalophore^TM^ matrix match. Cluster representatives at a cutoff distance of 1.4 (dashed line) are indicated via bold black lines and bold Uniprot IDs. Proteins containing the most similar binding sites to CHIKV nsP3, while still having three distinct protein folds, are highlighted in green.

Considering potential off-target and related side effects of drugs early in drug discovery can improve drug safety and decrease the risk of failure in (pre-)clinical trials. The structural dataset of the human proteome provided by Innophore and NVIDIA [15] enables a thorough in silico screening of human binding site cavities to facilitate the identification of potential off-target sites for the drug target cavity, in this case CHIKV nsP3. The off-target search resulted in 78 human binding sites meeting the requirements (minimum point-cloud overlap of query cavity: 50%; minimum point-cloud overlap of target cavity: 20%; and a maximum matching score of 0.1). A hierarchical clustering based on the matching scores resulted in two major clusters at a distance threshold of 1.4 (Figure 4). The cluster representatives, P11166, Q14376 and Q96J66, refer to the glucose transporter SLC2A1, the UDP-glucose 4-epimerase GALE and the ATP-binding cassette member ABCC11, respectively, and are highlighted in bold in Figure 4. Figure S2 in the supplementary material allows for a visual inspection of the representative potential off-target binding sites.

**Figure 5:**
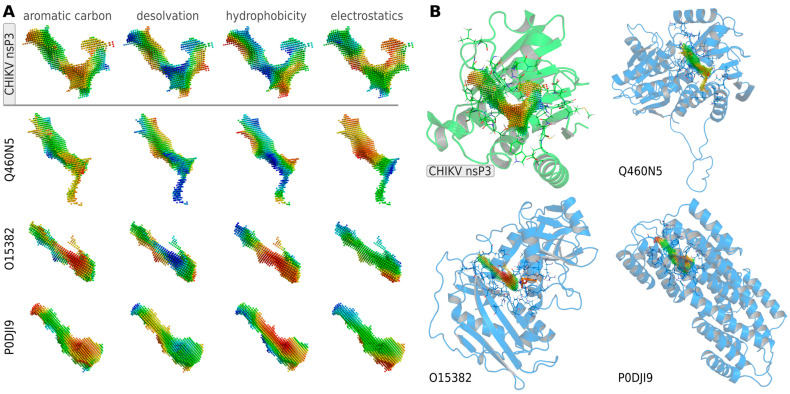
Visual inspection of binding-site similarities between human proteins and CHIKV nsP3. (**A**) Comparison of binding site properties of three of the most similar human binding sites, found in proteins Q460N5, O15382 and P0DJI9. (**B**) The overall structures of the proteins are shown with their binding-site cavities colored by the distribution of aromatic carbon atoms. Residues within 5 Å of the binding site cavity are shown as lines, mutated residues are shown as sticks. Point-clouds represent property gradients and are colored from red to blue, indicating decreasing density of aromatic carbon atoms, increasing solvent accessibility (decreasing desolvation), decreasing hydrophobicity and increasing negative charge, respectively.

In addition to the three cluster representatives, three of the most similar off-targets to CHIKV nsP3, based on their binding site properties, which present distinct protein folds, were selected as negative targets for lead optimization. These belong to the mono-ADP-ribosyltransferase PARP14, the branched-chain amino acid aminotransferase BCAT2 and the serum amyloid A-2 protein SAA2 (UniProt IDs: Q460N5, O15382, P0DJI9), which are highlighted in green in Figure 4. They show a strong similarity to CHIKV nsP3, especially in terms of the distribution of aromatic carbon atoms and desolvation potential, with Q460N5 being identified as the most similar cavity to nsP3 (Figure 5).

In this article, we present a process of refining molecules towards those with a lower binding energy to (i) the most probable off-targets (Q460N5, O15382, P0DJI9), which represent the three most similar binding sites to that of nsP3 which still show different protein folds, and (ii) the representatives of the three major clusters, P11166, Q14376 and Q96J66, together with Q460N5, which comprises the most similar binding site to that of nsP3.

### 3.3. Identification of Concerning Viral Variants

For the design of effective antivirals, continuous mutation monitoring is important to identify potentially drug-resistant variants in time and consider them as early as possible in the drug discovery process. Mutations at drug binding sites are especially concerning, and effects on binding site properties and shape can be evaluated via comparing Catalophore^TM^ cavities.

**Figure 6:**
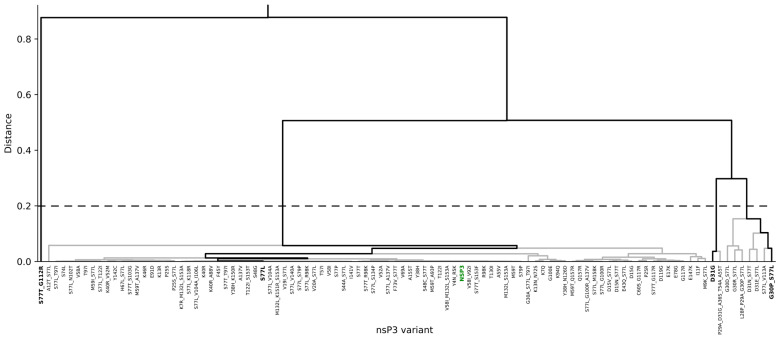
Clustering of nsP3 druggable sites of CHIKV mutants. Hierarchical clustering was performed based on the matching score of cavities calculated for all viral variants. Cluster representatives at a cutoff distance of 0.2 (dashed line) are highlighted in bold. The reference CHIKV nsP3 (NC_004162) is highlighted in green.

**Figure 7:**
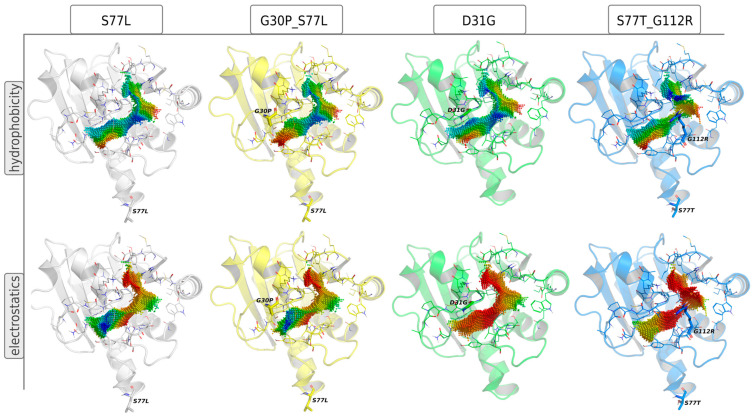
Effects of mutations on ADP-ribose binding-site properties in CHIKV nsP3. Four representative virus mutants were selected based on the properties of the ADP-ribose binding site of nsP3. Mutations are noted in single-letter codes (original-position-mutation), and multiple mutations are separated by “_”. Residues within 5 Å of the binding site cavity are shown as lines; mutated residues are shown as sticks. Point-clouds represent property gradients and are colored from red to blue, indicating decreasing hydrophobicity and increasing negative charge, respectively.

To inspect the effects of viral mutations on the properties and shape of the ADP-ribose binding site in CHIKV nsP3, all available genome sequences of CHIKV were downloaded from GISAID and NCBI (accessed in May 2024) and mutations within the nsP3 gene were identified. Within residues 1–160, which build the ADP-ribose binding domain, 100 non-redundant mutations were identified. Point-clouds representing the ADP-ribose binding sites of variants show that mutations at positions 30, 31 and 112, which are located at the binding interface, resulted in the most significant differences in the shape, as well as different properties of the respective point-clouds (Figure 6 and Figure 7). The protein variants S77L, G30P_S77L, D31G and S77T_G112R were selected as representatives in a hierarchical clustering based on binding site similarity. S77L is the most abundant nsP3 variant, with 1783 occurrences in 3437 nsP3 sequences extracted from genomes deposited on GISAID [18]. The mutation additionally occurs in almost every multipoint mutation identified. Mutations at position 77 do not affect the properties of the binding site because they are very distant from it.

Compared to the wild-type CHIKV ADP-ribose binding site (relating to S77L in Figure 7), the G30P mutation reduces the hydrophilicity of the binding site and results in a slightly different shape. D31G and G112R show a significant shift in electrostatics, by reducing negative charge and increasing positive charge in the binding site, respectively. G112R additionally introduces changes in cavity shape, slightly reducing its diameter in the core (Figure 7). These observations indicate the different binding behaviors of small molecules targeting the ADP-ribose binding site of the respective virus mutants.

### 3.4. Refinement of Small Molecules towards Safe and Effective Antivirals

A critical step in drug development is lead optimization once a promising set of candidate molecules has been found. We employed a genetic optimization algorithm to increase the binding affinity of molecules to viral druggable sites and reduce binding to potential off-target sites, which were identified by screening the human proteome for cavities similar to the viral drug target. At this stage, one can consider optimizing the binding to concerning virus mutants and/or optimizing binding to related viral species. The ADP-ribose binding sites of EEEV and SINV show a suitable similarity to that of CHIKV, and thus broad-spectrum antivirals, targeting all three species, might be developed.

#### 3.4.1. Inhibitor Refinement for Binding the Related Viral Species EEEV and SINV

**Figure 8:**
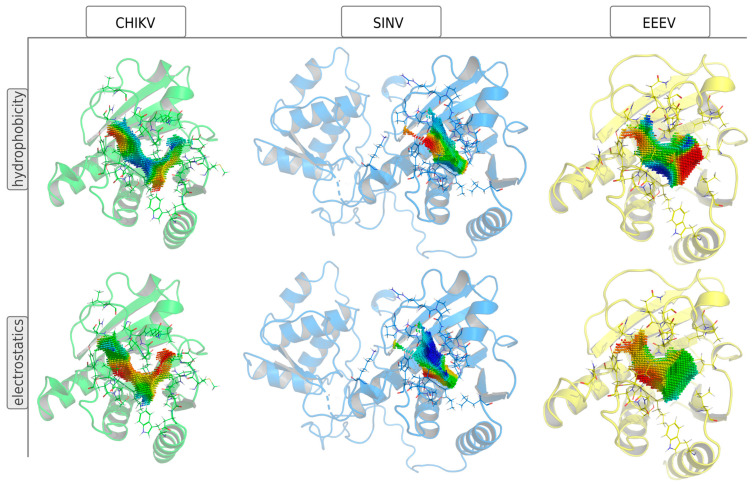
Structural comparison of ADP-ribose binding sites in CHIKV-related species. The ADP-ribose binding sites in nsP3 (CHIKV and SINV) and NS3 (EEEV) proteins are represented by 3D point-clouds, which are colored by hydrophobicity or electrostatics potential. Residues within 5 Å of the binding site cavity are shown as lines. Point-clouds represent property gradients and are colored from red to blue, indicating decreasing hydrophobicity and increasing negative charge, respectively.

Generating broad-spectrum antivirals, which are active against several related species is an important task. Here, we present our efforts to design molecules inhibiting the alphaviruses CHIKV, EEEV and SINV while decreasing binding to potential human off-targets. These three viruses contain structurally similar ADP-ribosyl hydrolases; while a visual inspection of the binding site properties reveals significant differences in the ADP-ribose binding sites, both in shape and properties like hydrophobicity and electrostatics (Figure 8).

The most similar human binding sites to the ADP-ribose binding site in CHIKV nsP3 are contained in Q460N5, O15382 and P0DJI9 (shown in Figure 5). These proteins of different protein folds were considered as negative targets in the optimization algorithm. Candidate molecules were selected by matching the three viral ADP-ribose binding sites to ligand binding cavities calculated from structures deposited in the PDB and subsequent docking of the ligands contained in the most similar cavities into the viral binding sites. The final selection was based on a predicted binding energy of lower than −8 kcal/mol to one or all three viral sites, resulting in 66 ligands. Additionally, 10 candidates were selected for their great binding-site similarity to all three viral ADP-ribose binding sites. Details regarding the selection of candidates are available in Table S1.

The best-performing molecule throughout the 20 generations originated from 2-Amino-Phenylamino-Dibenzosuberone (Table 1), which was initially identified via Catalophore^TM^ matching of the ADP-ribose binding site in CHIKV nsP3 to the ligand-binding cavities calculated from all structures stored in the PDB (Figure S3). 2-Amino-Phenylamino-Dibenzosuberone (ligand PDB-ID: 2A8) is found in PDB-ID 3ZYA, a human p38 MAP Kinase, and is a selective inhibitor developed for biological research, to inhibit a special kind of kinase for signaling pathway studies [45].

2-Amino-Phenylamino-Dibenzosuberone initially shows its highest binding affinity to off-target P0DJI9, with −11.70 kcal/mol. The addition of substituents at the benzene ring and the six-membered ring reduced the binding to P0DJI9 by up to 4.58 kcal/mol, while binding to Q460N5 and O15382 was unchanged or reduced by up to 1.29 kcal/mol. The binding to 3ZYA, the human kinase for which the inhibitor was intentionally designed, additionally decreased drastically by up to 2.9 kcal/mol, reducing an additional threatening side effect, even though this structure was not included as a negative target in the optimization algorithm. As a trade-off, these changes led to a decreased binding to CHIKV nsP3; however, less significantly, by up to 1.18 kcal/mol, bringing it to a similar binding-affinity level with EEEV and SINV, for which binding did not change or was decreased slightly by up to 0.39 kcal/mol.

Notably, the optimization algorithm produced tight binders, also based on 2-Amino-Phenylamino-Dibenzosuberone, when no negative targets were given (Table 1, batch 2). However, the generated molecules bound unsuitably tightly to the off-targets and exhibited strong binding to the human kinase 3YZA, the original target of the inhibitor, when separately assessed via molecular docking. Optimization without considering off-target effects clearly produces strong binders to viral proteins, but these molecules are highly likely to induce serious side effects. This stresses the importance of including side effect screening from the beginning of the drug discovery process. In batch 1, binding to 3ZYA was reduced by the optimization algorithm, even though it was not included as a negative target (the binding energy was calculated separately). This indicates that including several potential off-targets as negative targets in the optimization algorithm might also reduce binding to other potential off-targets, if the binding site is similar.

#### 3.4.2. Inhibitor Refinement for Binding to Several Viral Variants

Viral mutations might introduce changes into drug binding sites which can alter drug binding significantly. Through continuous viral mutation monitoring, emerging concerning virus variants can be considered early in drug development or refinement. Our analysis revealed four major clusters of binding sites for CHIKV variants, mainly differing in the electrostatic potential at the ADP-ribose binding site (Figure 6 and Figure 7). For molecular optimization towards improved binding to viral variants, these four representatives, namely variants S77L, G30P_S77L, D31G and S77T_G112R, were selected as positive targets.

Potential off-target sites have been identified and compared to each other (Figure 4 and Figure 5). Three representative off-targets (P11166, Q96J66, Q14376) were selected, together with the most similar human binding site to the ADP-ribose binding site in CHIKV nsP3, (Q460N5) as negative targets for molecular optimization (Table 2). Candidate molecules for the optimization algorithm included the natural substrate ADP-ribose, together with 39 ligands identified via cavity matching of the virus mutant’s ADP-ribose binding site to ligand-binding sites in proteins obtained from the PDB. Additionally, 37 drug-like molecules retrieved from ChEMBL, which were selected for their low binding energy for the reference CHIKV nsP3 (evaluated via docking, energies ranging from −8.83 to −12.07 kcal/mol) were considered. Details on the selection of candidates are available in Table S2.

Gliclazide (DrugBank Accession Number: DB01120, PDB ligand ID: GCZ, included in structure 4ZFC [46]), a sulfonylurea, and its optimized variants were the top-performing candidates after 20 generations of refinement (Table 2). Gliclazide is an approved therapeutic for treating hyperglycemia in patients with type 2 diabetes [47]. The drug initially showed especially strong binding to off-target P11166 (−10.56 kcal/mol), with a similar binding affinity for this off-target as for three of the four considered viral variants. Molecular changes, which led to an overall decreased off-target binding and increased binding to viral nsP3 variants were introduced to the bicyclic ring system or onto the sulfonamide group, whereas the carbamid and the benzene ring was not changed by the optimization algorithm.

While the genetic optimization algorithm experiences a clear trade-off between improving binding to positive and decreasing binding to negative targets, the gap between binding affinities evaluated for the viral variants compared to off-targets increased significantly. Notably, at this stage, protein backbone dynamics are not considered in the binding evaluation, which, however, will certainly influence the binding mode and affinity of small-molecule drugs to positive and negative target proteins.

One more comment is important at this point: In addition to seeding our optimization runs with promising candidates augmented by randomly generated SMILES strings, we also started the algorithm on a purely randomly generated set of SMILES strings. This was carried out in order to understand the capabilities of the approach if used from scratch as well as to create a possible baseline of results beyond which we could push our preferred leads by means of optimization. While the results from runs based on random populations are obviously of mixed quality, we were able to recognize certain substructures in the optimized population that were similar to our chosen leads. That finding allows us to assume that interesting results could also be obtained without any concrete leads regarding a target cavity.

## 4. Discussion

In this article, we discuss the potential of repurposing drugs targeting CHIKV nsP3, present a novel drug discovery approach for designing inhibitors with enhanced efficacy towards viral variants or related species and, most importantly, we demonstrate a protocol to discover potential side effects at the earliest stage of drug discovery. Once the drug target, i.e., a druggable viral binding site, is defined, a rapid side effect screening can be performed to search for similar binding sites in the human organism via the CavitOmiX technology [15]. On the other hand, potential drug candidates can be identified by searching a ligand-containing database of binding sites, which may result in the selection of already approved drugs suitable for off-label use [39]. For nsP3, we identified the antiviral Remdesivir and its analog GS-441524, as well as the pain medications Naproxen and, although less suitable because of potential side-effects, Sunitinib, as candidates for drug-repurposing. It is worth mentioning that two out of three proteins from which these compounds originate show no significant sequence identity and are structurally different from CHIKV nsP3. This shows the potential of the CavitOmiX technology to identify potential inhibitors which might have been missed by methods that focus on sequence and structure similarity. Utilizing CavitOmiX for drug repurposing therefore presents a promising strategy for identifying approved drugs for off-label applications. By narrowing the focus to crucial drug binding sites, it expands the potential pool of hits beyond those with similar sequences or overall protein structures.

As Shimizu et al. proposed, drugs which are selected to bind to the ADP-ribose binding site of CHIKV nsP3 most probably will also bind to ADP-ribose binding sites of cellular macrodomains [22] and thus will lead to side effects in patients. We support this statement, and we show the significant similarity of some human binding sites, especially of ADP-ribosyltransferases and -hydrolases, to the viral drug-target nsP3. Lead optimization without considering off-target binding resulted in molecules strongly binding to related viral species, but also increased binding to human proteins to a similar extent. On the contrary, when potential off-targets are considered, molecules are specifically refined for lower binding to these human binding sites, or increased binding for positive targets, which widens the binding energy gap between viral drug targets and human off-targets. Consequently, our results emphasize that drug development, particularly when targeting proteins with a likely unfavorable toxicity profile, must include an evaluation of the potential inhibition of human proteins at the earliest stage possible.

Considering the use of our genetic algorithm, it is interesting to note that a scalarized approach to a multi-objective optimization problem such as the one described herein is both advantageous and limiting. The advantage comes from the fact that a good choice of scalarization weights for the individual scores enables a user to run the algorithm without much further thought or steering. However, at the same time further details are available from our approach for comparing individual score performance and informing the user’s choice of scalarization weights in the first place. Still, a more general approach to the underlying optimization problem can and should be employed in future studies.

In the same spirit, there is a tradeoff between increasing binding to viral variants and decreasing binding to potential off-targets. Such an effect becomes more pronounced, the more different from each other the viral variants or off-target sites are. However, the important conclusion at this point is to recognize the ability to keep binding levels required for targets at a desirably high level (or even increase those) while significantly decreasing the binding affinity to off-targets. This is the most remarkable finding from our investigation at the present time. More detailed insights will be the outcome of our ongoing investigation.

We are certain that the in silico drug discovery approach presented herein, together with the human proteome resource which is provided by Innophore in cooperation with NVIDIA [15], will have great value in future drug development.

## Figures and Tables

**Table 1:** Binding evaluation for optimized molecules targeting CHIKV, EEEV and SINV. Top performing molecules after 20 generations of the genetic optimization algorithm are given, together with the final rank, the generation number of first occurrence of the molecule (#gen), and binding energies (as well as difference in binding compared to the original molecule) retrieved from molecular docking. Negative targets (potential off-targets) were considered in batch 1, but not in batch 2. Values in brackets were determined separately, as these targets were not included during optimization. BE…binding energy, given in kcal/mol.

Final Rank	#Gen	Structure	BE to Viral Species [kcal/mol] CHIKV|EEEV|SINV	BE to Off-Targets [kcal/mol] Q460N5|O15382|P0DJI9	BE to 3ZYA [kcal/mol]
Original Molecule—2-Amino-Phenylamino-Dibenzosuberone					
-	0	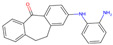	−10.69|−9.88|−9.70	−8.72|−8.78|−11.70	(−10.67)
Batch 1|Considering off-target effects					
1	1	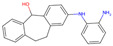	−10.07|−9.59|−9.31 +0.62|+0.29|+0.39	−8.78|−8.27|−7.12 −0.06|+0.51|+4.58	(−9.78) (+0.89)
2	19	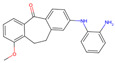	−9.72|−9.62|−9.63 +0.97|+0.28|+0.07	−7.43|−8.37|−9.15 +1.29|+0.41|+2.55	(−7.77) (+2.90)
3	15	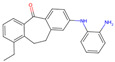	−9.51|−9.88|−9.74 +1.18|0|−0.04	−8.74|−8.07|−8.66 −0.02|+0.71|+3.04	(−8.16) (+2.51)
Batch 2|Without considering off-target effects					
1	8	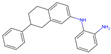	−11.37|−10.91|−10.38 −0.68|−1.03|−0.68	(−9.71|−10.40|−11.59) (−0.99|−1.62|+0.11)	(−11.55) (−0.88)
2	2	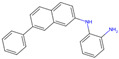	−10.87|−10.85|−10.10 −0.18|−0.97|−0.40	(−9.03|−10.80|−12.15) (−0.31|−2.02|−0.45)	(−11.42) (−0.75)
3	17	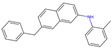	−11.02|−10.38|−10.06 −0.33|−0.50|−0.36	(−9.57|−10.39|−12.03) (−0.85|−1.61|−0.33)	(−10.20) (+0.47)

**Table 2:** Binding evaluation of optimized molecules for viral variants and potential off-targets. Top performing molecules after 20 generations of the genetic optimization algorithm are given, together with the final rank, the generation number of first occurrence of the molecule (#gen), and binding energies retrieved from molecular docking. Values in brackets were determined separately, as these targets were not included during optimization. BE…binding energy, given in kcal/mol.

Final Rank	#Gen	Structure	BE to Viral Variants S77L|S77T_G112R|D31G|G30P_S77L [kcal/mol]	BE to Off-Target Sites Q460N5|P11166|Q96J66|Q14376 [kcal/mol]	BE to 4ZFC [kcal/mol]
Original Molecule—Gliclazide					
-	0	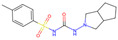	−10.97|−11.71|−10.71|−10.03	−8.16|−10.56|−8.52|−9.70	(−9.14)
Batch 1					
1	15	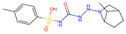	−12.18|−12.55|−12.02|−10.74 −1.21|−0.84|−1.31|−0.71	−8.19|−9.94|−8.49|−8.79 −0.03|+0.62|+0.03|+0.91	(−8.93) (+0.21)
2	6	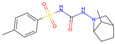	−11.38|−12.10|−11.16|−10.59 −0.41|−0.39|−0.45|−0.56	−7.68|−9.80|−8.07|−8.46 +0.48|+0.76|+0.45|+1.24	(−9.17) (−0.03)
3	8	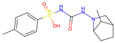	−11.37|−12.13|−11.17|−10.61 −0.40|−0.42|−0.46|−0.58	−7.82|−9.73|−8.18|−8.85 +0.34|+0.83|+0.34|+0.85	(−8.81) (+0.33)

## Data Availability

Publicly available genome sequences were downloaded from https://www.gisaid.org/ (accessed on 15 June 2024) and https://www.ncbi.nlm.nih.gov/ (accessed on 15 June 2024). An acknowledgment table including accession numbers and the origin of the processed sequences is accessible in the Supplementary Material (Table S3), together with additional supporting data.

## Supplementary Materials

The following supporting information can be downloaded at: https://www.mdpi.com/article/10.3390/v16081186/s1, Figure S1: Binding-site comparison of Sunitinib to CHIKV nsP3 via Catalophore^TM^ matching; Figure S2: Structural representation of potential off-targets for therapeutics targeting CHIKV nsP3; Figure S3: Binding-site comparison of CHIKV nsP3 and 3ZYA; Table S1: Candidate_smiles_1; Table S2: Candidate_smiles_2; Table S3: Acknowledgement_Table.
