# Supplementary material for: CavitOmiX Drug Discovery: Engineering Antivirals with Enhanced Spectrum and Reduced Side Effects for Arboviral Diseases
Source: Viruses. 2024 Jul 24;16(8):1186. doi: 10.3390/v16081186 (PMC11360613; doi:10.3390/v16081186)
Supplement: Supplementary file 1 [file viruses-16-01186-s001.zip › Supplementary_Information.pdf]

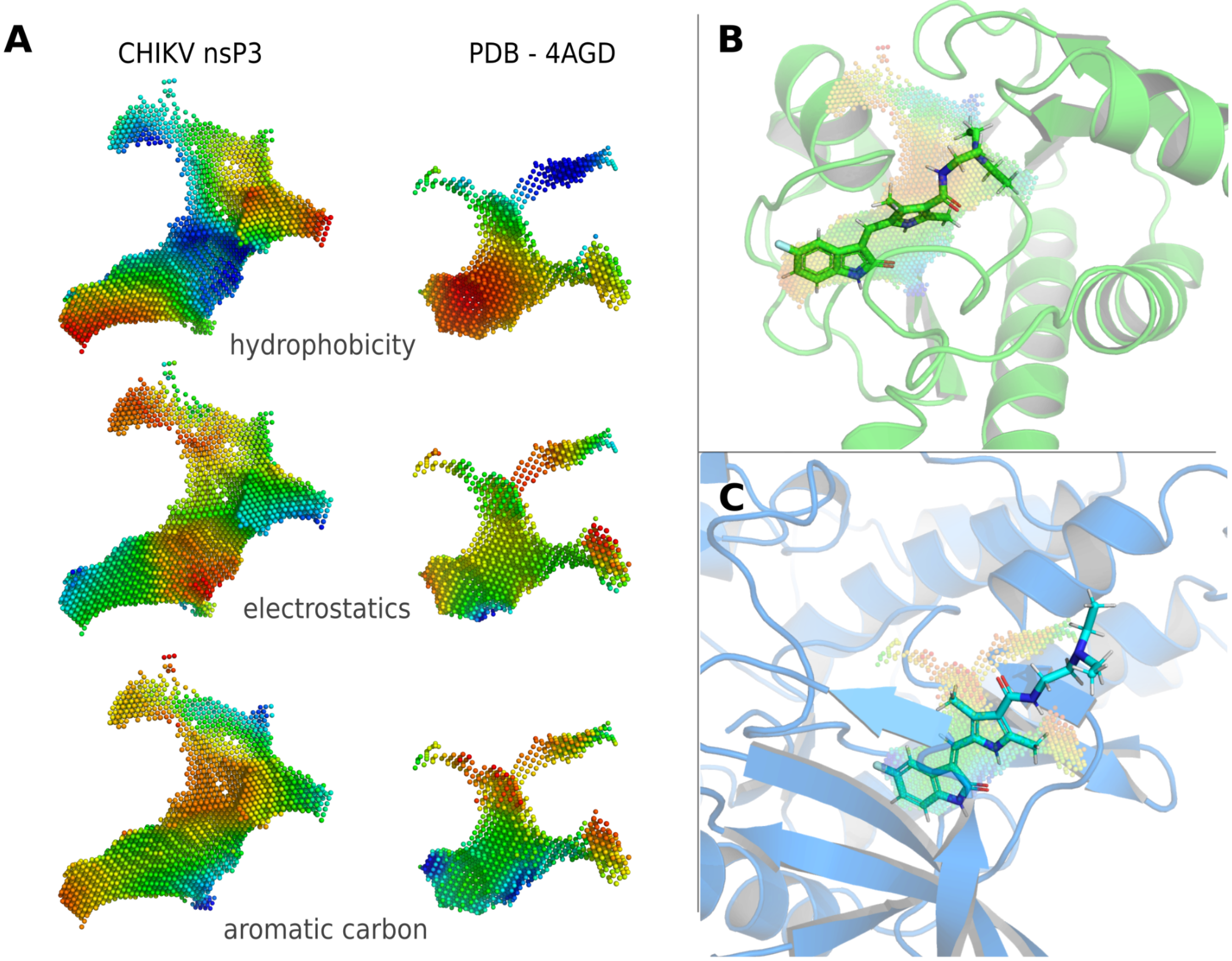

Figure S1 | Binding-site comparison of Sunitinib to CHIKV nsP3 via Catalophore™ matching. A) Physico-chemical property comparison of the ADP-binding site in CHIKV nsP3 and Sunitinib-binding site in human vascular endothelial growth factor receptor 2 (PDB ID: 4AGD[1]). B) Binding mode of Sunitinib to CHIKV nsP3 identified via molecular docking. C) Binding mode of Sunitinib to vascular endothelial growth factor receptor 2 as in PDB 4AGD. Vascular endothelial growth factor receptor 2 and CHIKV nsP3 do not show a similar fold (TM-score= 0.23002) and so no significant sequence identity.

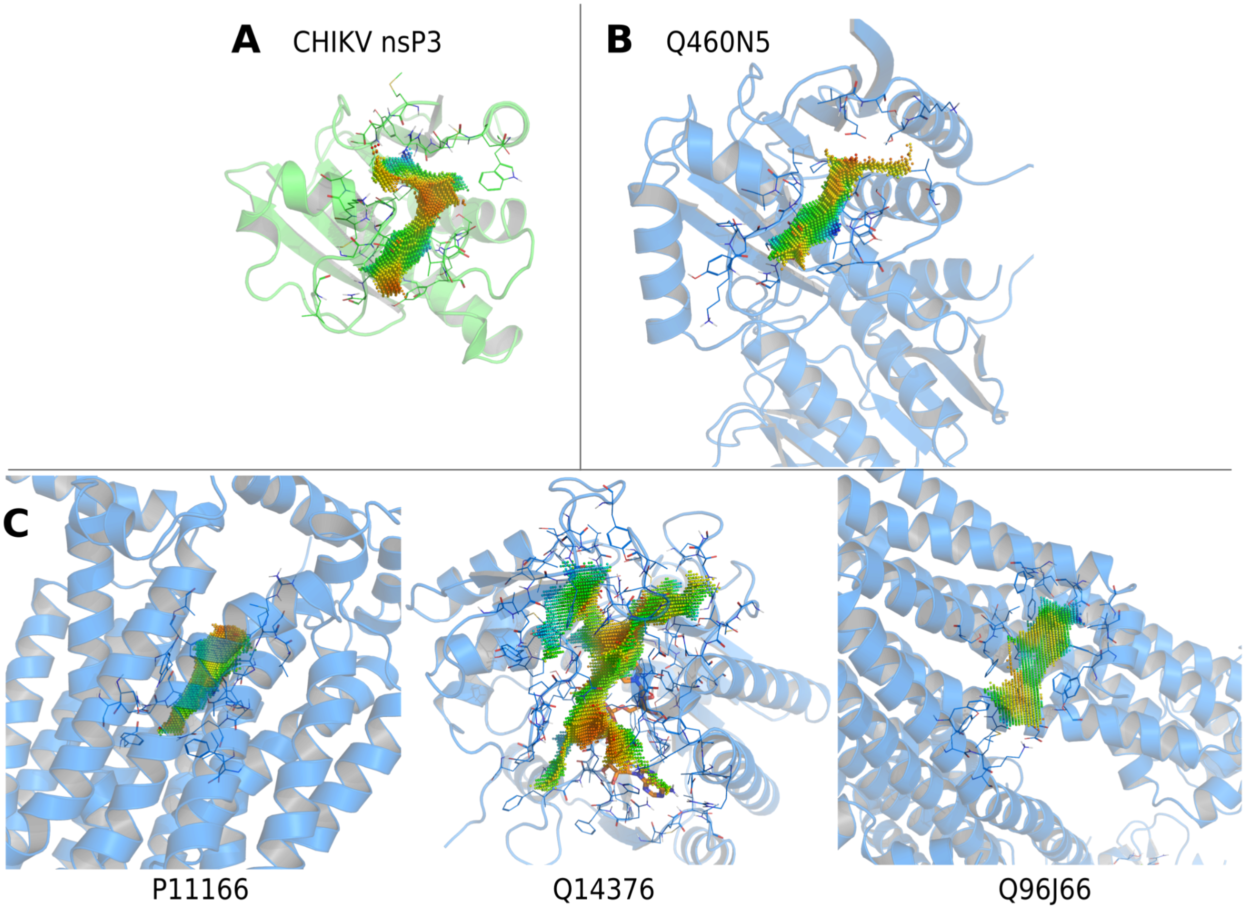

Figure S2 | Structural representation of potential off-targets for therapeutics targeting CHIKV nsP3. A) ADP-ribosyl binding cavity of CHIKV nsP3. B) The cavity of human mono-ADP-ribosyltransferase PARP14 (UniProt ID Q460N5) was identified to contain the most similar binding site to CHIKV nsP3. C) Three cluster representatives originating from a hierarchical clustering of the most similar human cavities to CHIKV nsP3. Point-cloud representations of binding sites are colored by the distribution of aromatic carbon atoms. Residues within 5 Å of the binding-site cavity are shown as sticks.

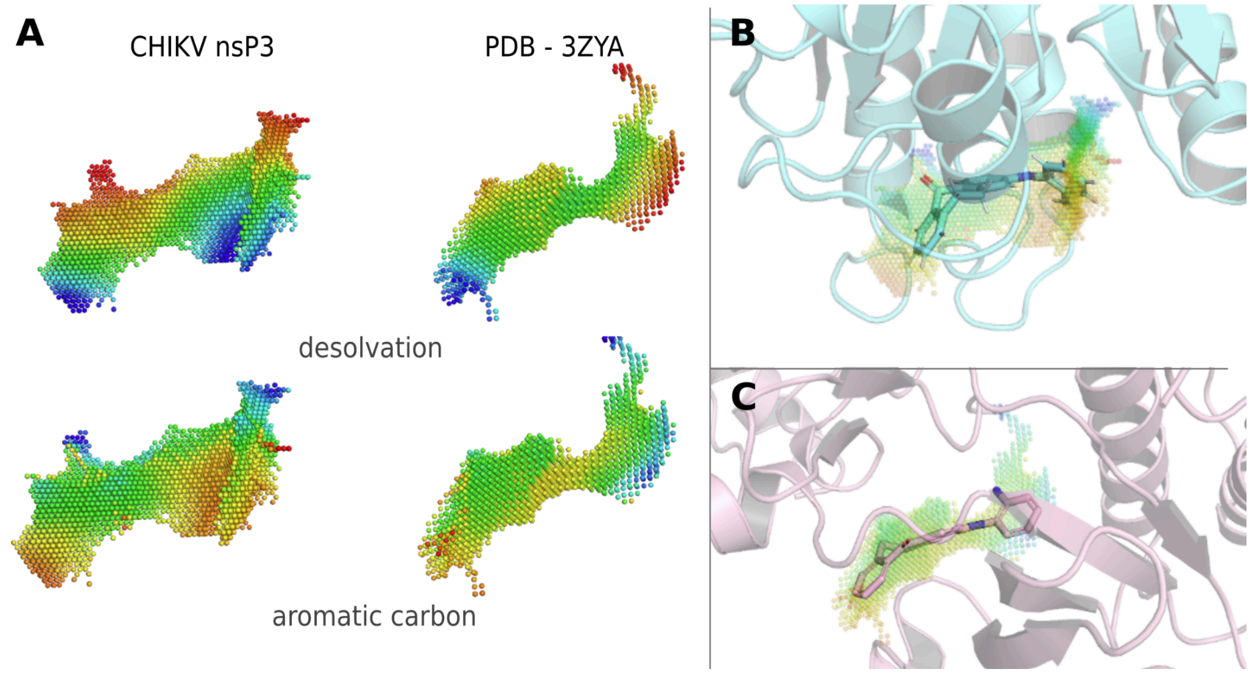

Figure S3 | Binding-site comparison of CHIKV nsP3 and 3ZYA[2]. A) Comparison of desolvation and the distribution of aromatic carbon in the ADP-ribose binding site of CHIKV nsP3 and the binding-site of 2A8 to 3ZYA. B) Molecular docking of 2A8 into CHIKV nsP3. C) Experimentally determined binding pose of 2A8 in 3ZYA.

The following data is available as separate files:

Table S1 | Candidate smiles for refinement of drugs against CHIKV, EEEV and SINV. The table contains SMILES, selection basis, binding energy derived from docking (if applicable) and binding-site similarity to CHIKV nsP3 ADP-ribose binding sites (if applicable).

Table S2 | Candidate smiles for refinement of drugs against CHIKV variants. The table contains SMILES, selection basis, binding energy derived from docking (if applicable) and binding-site similarity to CHIKV nsP3 ADP-ribose binding sites (if applicable).

Table S3 | Acknowledgement table containing accession numbers of genome sequences used in this study.

References

1. McTigue, M. *et al.* Molecular conformations, interactions, and properties associated with drug efficiency and clinical performance among VEGFR TK inhibitors. *Proc. Natl. Acad. Sci. U.S.A.* **109**, 18281–18289 (2012).

2. Koeberle, S. C. *et al.* Skepinone-L is a selective p38 mitogen-activated protein kinase inhibitor. *Nat Chem Biol* **8**, 141–143 (2012).
