# Supplementary material for: CavitOmiX Drug Discovery: Engineering Antivirals with Enhanced Spectrum and Reduced Side Effects for Arboviral Diseases
Source: Viruses. 2024 Jul 24;16(8):1186. doi: 10.3390/v16081186 (PMC11360613; doi:10.3390/v16081186)
Supplement: Supplementary file 1 [file viruses-16-01186-s001.zip › Table S3. Acknowledgement_Table.pdf]

We gratefully acknowledge the following Authors from the Originating laboratories responsible for obtaining the specimens, as well as the Submitting laboratories where the genome data were generated and shared via GISAID, on which this research is based.

All Submitters of data may be contacted directly via www.gisaid.org

Authors are sorted alphabetically.

Accession ID	Originating Laboratory	Submitting Laboratory	Authors
EPI_ISL_17454776	All India Institute of Medical Sciences	All India Institute of Medical Sciences	Chahar,H.S., Bharaj,P., Dar,L., Guleria,R., Kabra,S.K. and Broor,S.
EPI_ISL_17454872	Aix-Marseille Université	Aix-Marseille Université	Moyen,N., Thiberville,S.D., Pastorino,B., Nougairede,A., Thirion,L., Mombouli,J.V., Dimi,Y., Leparc-Goffart,I., Capobianchi,M.R., Lepfoundzou,A.D. and de Lamballerie,X.
EPI_ISL_17454873	Aix-Marseille Université	Aix-Marseille Université	Baronti,C., Piorkowski,G. and de Lamballerie,X.
EPI_ISL_17454874, EPI_ISL_17454875, EPI_ISL_17454876, EPI_ISL_17454877, EPI_ISL_17454878, EPI_ISL_17454879	Aix-Marseille Université	Aix-Marseille Université	Moyen,N., Thiberville,S.D., Pastorino,B., Nougairede,A., Thirion,L., Mombouli,J.V., Dimi,Y., Leparc-Goffart,I., Capobianchi,M.R., Lepfoundzou,A.D. and de Lamballerie,X.
EPI_ISL_17454880	All India Institute of Medical Sciences	All India Institute of Medical Sciences	Brijwal,M., Sahu,M., Kumar,P., Choudhary,A., Namdeo,D., Baruah,K. and Dar,L.
EPI_ISL_17454881	All India Institute of Medical Sciences	All India Institute of Medical Sciences	Siva Raghavendhar,B. and Pratima,R.
EPI_ISL_17454882	All India Institute of Medical Sciences	All India Institute of Medical Sciences	Ray,P. and Raghavendhar,B.S.
EPI_ISL_17454883	All India Institute of Medical Sciences	All India Institute of Medical Sciences	Siva Raghavendhar,B. and Pratima,R.
EPI_ISL_17454884, EPI_ISL_17454885	All India Institute of Medical Sciences	All India Institute of Medical Sciences	Ray,P. and Raghavendhar,B.S.
EPI_ISL_17454886	All India Institute of Medical Sciences	All India Institute of Medical Sciences	Siva Raghavendhar,B. and Pratima,R.
EPI_ISL_17454887, EPI_ISL_17454888	All India Institute of Medical Sciences	All India Institute of Medical Sciences	Ray,P. and Raghavendhar,B.S.
EPI_ISL_17454889	All India Institute of Medical Sciences	All India Institute of Medical Sciences	Ray,P. and Raghavendhar,S.
EPI_ISL_17454890, EPI_ISL_17454891, EPI_ISL_17454892	All India Institute of Medical Sciences	All India Institute of Medical Sciences	Siva Raghavendhar,B. and Pratima,R.
EPI_ISL_17454893	All India Institute of Medical Sciences	All India Institute of Medical Sciences	Ray,P. and Raghavendhar,S.
EPI_ISL_17454894	All India Institute of Medical Sciences	All India Institute of Medical Sciences	Agarwal,A., Gupta,S. and Biswas,D.
EPI_ISL_17454895, EPI_ISL_17454896	All India Institute of Medical Sciences	All India Institute of Medical Sciences	Kushekhar,B.V. and Ray,P.
EPI_ISL_17454897	All India Institute of Medical Sciences	All India Institute of Medical Sciences	Siva Raghavendhar,B. and Pratima,R.
EPI_ISL_17454898	All India Institute of Medical Sciences	All India Institute of Medical Sciences	Ray,P. and Raghavendhar,S.
EPI_ISL_17454899, EPI_ISL_17454900	All India Institute of Medical Sciences	All India Institute of Medical Sciences	Agarwal,A., Gupta,S. and Biswas,D.
EPI_ISL_17454901	All India Institute of Medical Sciences	All India Institute of Medical Sciences	Ray,P. and Raghavendhar,B.S.
EPI_ISL_17454902, EPI_ISL_17454903	All India Institute of Medical Sciences	All India Institute of Medical Sciences	Kushekhar,B.V. and Ray,P.
EPI_ISL_17454904	All India Institute of Medical Sciences	All India Institute of Medical Sciences	Agarwal,A., Gupta,S. and Biswas,D.
EPI_ISL_17454905, EPI_ISL_17454906, EPI_ISL_17454907, EPI_ISL_17454908, EPI_ISL_17454909	All India Institute of Medical Sciences	All India Institute of Medical Sciences	Ray,P. and Raghavendhar,B.S.
EPI_ISL_17454910	All India Institute of Medical Sciences	All India Institute of Medical Sciences	Agarwal,A., Gupta,S. and Biswas,D.
EPI_ISL_17454911	All India Institute of Medical Sciences	All India Institute of Medical Sciences	Ray,P. and Raghavendhar,B.S.
EPI_ISL_17454912, EPI_ISL_17454913	All India Institute of Medical Sciences	All India Institute of Medical Sciences	Siva Raghavendhar,B. and Pratima,R.
EPI_ISL_17454914	All India Institute of Medical Sciences	All India Institute of Medical Sciences	Chahar,H.S., Bharaj,P., Dar,L., Guleria,R., Kabra,S.K. and Broor,S.
EPI_ISL_17454915	All India Institute of Medical Sciences	All India Institute of Medical Sciences	Siva Raghavendhar,B. and Pratima,R.
EPI_ISL_17454916, EPI_ISL_17454917, EPI_ISL_17454918	All India Institute of Medical Sciences	All India Institute of Medical Sciences	Chahar,H.S., Bharaj,P., Dar,L., Guleria,R., Kabra,S.K. and Broor,S.
EPI_ISL_17454919	All India Institute of Medical Sciences	All India Institute of Medical Sciences	Diddi,K., Kaushik,S., Dar,L. and Broor,S.
EPI_ISL_17454920	All India Institute of Medical Sciences	All India Institute of Medical Sciences	Kushekhar,B.V. and Ray,P.
EPI_ISL_17454921	All India Institute of Medical Sciences	All India Institute of Medical Sciences	Diddi,K., Kaushik,S., Dar,L. and Broor,S.
EPI_ISL_17454922	All India Institute of Medical Sciences	All India Institute of Medical Sciences	Kushekhar,B.V. and Ray,P.
EPI_ISL_17454923	All India Institute of Medical Sciences	All India Institute of Medical Sciences	Agarwal,A., Gupta,S. and Biswas,D.
EPI_ISL_17454924	All India Institute of Medical Sciences	All India Institute of Medical Sciences	Kushekhar,B.V. and Ray,P.
EPI_ISL_17454925	All India Institute of Medical Sciences	All India Institute of Medical Sciences	Ray,P. and Raghavendhar,S.
EPI_ISL_17454926	All India Institute of Medical Sciences	All India Institute of Medical Sciences	Kushekhar,B.V. and Ray,P.
EPI_ISL_17454927	All India Institute of Medical Sciences	All India Institute of Medical Sciences	Agarwal,A., Gupta,S. and Biswas,D.
EPI_ISL_17454928	All India Institute of Medical Sciences	All India Institute of Medical Sciences	Siva Raghavendhar,B. and Pratima,R.
EPI_ISL_17454929	All India Institute of Medical Sciences	All India Institute of Medical Sciences	Diddi,K., Kaushik,S., Dar,L. and Broor,S.
EPI_ISL_17454930	All India Institute of Medical Sciences	All India Institute of Medical Sciences	Agarwal,A., Gupta,S. and Biswas,D.
EPI_ISL_17454931, EPI_ISL_17454932, EPI_ISL_17454933, EPI_ISL_17454934	All India Institute of Medical Sciences	All India Institute of Medical Sciences	Kushekhar,B.V. and Ray,P.
EPI_ISL_17454935, EPI_ISL_17454936	All India Institute of Medical Sciences	All India Institute of Medical Sciences	Diddi,K., Kaushik,S., Dar,L. and Broor,S.
EPI_ISL_17454937	All India Institute of Medical Sciences	All India Institute of Medical Sciences	Kushekhar,B.V. and Ray,P.
EPI_ISL_17454938, EPI_ISL_17454939	All India Institute of Medical Sciences	All India Institute of Medical Sciences	Ray,P. and Raghavendhar,B.S.
EPI_ISL_17454940	All India Institute of Medical Sciences	All India Institute of Medical Sciences	Kushekhar,B.V. and Ray,P.
EPI_ISL_17454941	All India Institute of Medical Sciences	All India Institute of Medical Sciences	Ray,P. and Raghavendhar,B.S.
EPI_ISL_17454942, EPI_ISL_17454943, EPI_ISL_17454944	All India Institute of Medical Sciences	All India Institute of Medical Sciences	Siva Raghavendhar,B. and Pratima,R.
EPI_ISL_17454945	All India Institute of Medical Sciences	All India Institute of Medical Sciences	Ray,P. and Raghavendhar,B.S.
EPI_ISL_17454946, EPI_ISL_17454947	All India Institute of Medical Sciences	All India Institute of Medical Sciences	Siva Raghavendhar,B. and Pratima,R.
EPI_ISL_17454948	All India Institute of Medical Sciences	All India Institute of Medical Sciences	Kushekhar,B.V. and Ray,P.
EPI_ISL_17454949	All India Institute of Medical Sciences	All India Institute of Medical Sciences	Siva Raghavendhar,B. and Pratima,R.
EPI_ISL_17454950	All India Institute of Medical Sciences	All India Institute of Medical Sciences	Ray,P. and Raghavendhar,B.S.
EPI_ISL_17454951, EPI_ISL_17454952	All India Institute of Medical Sciences	All India Institute of Medical Sciences	Kushekhar,B.V. and Ray,P.
EPI_ISL_17454953	All India Institute of Medical Sciences	All India Institute of Medical Sciences	Agarwal,A., Gupta,S. and Biswas,D.
EPI_ISL_17454954	All India Institute of Medical Sciences	All India Institute of Medical Sciences	Kushekhar,B.V. and Ray,P.
EPI_ISL_17454955	All India Institute of Medical Sciences	All India Institute of Medical Sciences	Agarwal,A., Gupta,S. and Biswas,D.
EPI_ISL_17454956	All India Institute of Medical Sciences	All India Institute of Medical Sciences	Kushekhar,B.V. and Ray,P.
EPI_ISL_17454957	All India Institute of Medical Sciences	All India Institute of Medical Sciences	Ray,P. and Raghavendhar,B.S.
EPI_ISL_17454958	All India Institute of Medical Sciences	All India Institute of Medical Sciences	Chahar,H.S., Bharaj,P., Dar,L., Guleria,R., Kabra,S.K. and Broor,S.
EPI_ISL_17454959	Annamalai University	Annamalai University	Prasanna Kumar,C.
EPI_ISL_17454960, EPI_ISL_17454961, EPI_ISL_17454962, EPI_ISL_17454963, EPI_ISL_17454964, EPI_ISL_17454965, EPI_ISL_17454966	Army Hospital Research And Referral, Department of Lab Sciences and Molecular Biology	Army Hospital Research And Referral, Department of Lab Sciences and Molecular Biology	Tandel,K., Mahadevan Kumar, Shergill,S.P.S., Sahai,K. and Gupta,R.M.
EPI_ISL_17454967	Beijing Institute of Microbiology and Epidemiology	Beijing Institute of Microbiology and Epidemiology	Guo,X.X., Zhang,Y.M., Li,C.X., Zhang,G.L., Zheng,Z., Dong,Y.D., Xue,R.D., Xing,D. and Zhao,T.Y.
EPI_ISL_17454968	Beijing Institute of Microbiology and Epidemiology	Beijing Institute of Microbiology and Epidemiology	Li,X.F., Jiang,T., Deng,Y.Q., Zhao,H., Yu,X.D., Ye,Q., Wang,H.J., Zhu,S.Y., Zhang,F.C., Qin,E.D. and Qin,C.F.

EPI_ISL_17454969, EPI_ISL_17454970, EPI_ISL_17454971, EPI_ISL_17454972, EPI_ISL_17454973, EPI_ISL_17454974	Bharat Biotech	Bharat Biotech	ELLA,K.M. and KANDASWAMY,S.
EPI_ISL_17454975	Bharat Biotech	Bharat Biotech	Ella,K.M., Sumathy,K., Pydigummala,J.S. and Hedge,N.R.
EPI_ISL_17454976	Bharat Biotech	Bharat Biotech	Murthy,E.K. and Sumathy,K.
EPI_ISL_17454977, EPI_ISL_17454978	Bharat Biotech	Bharat Biotech	ELLA,K.M. and KANDASWAMY,S.
EPI_ISL_17454979	Bharat Biotech	Bharat Biotech	Ella,K.M., Sumathy,K., Pydigummala,J.S. and Hedge,N.R.
EPI_ISL_17454980	Bharat Biotech	Bharat Biotech	ELLA,K.M. and KANDASWAMY,S.
EPI_ISL_17454981	Bharat Biotech	Bharat Biotech	Ella,K.M., Sumathy,K., Pydigummala,J.S. and Hedge,N.R.
EPI_ISL_17454982, EPI_ISL_17454983	Bharat Biotech	Bharat Biotech	ELLA,K.M. and KANDASWAMY,S.
EPI_ISL_17454984	Bharat Biotech	Bharat Biotech	Sumathy,K. and Ella,K.M.
EPI_ISL_17454985, EPI_ISL_17454986, EPI_ISL_17454987, EPI_ISL_17454988	Bharat Biotech	Bharat Biotech	Murthy,E.K. and Sumathy,K.
EPI_ISL_17454989	Bharat Biotech	Bharat Biotech	Sumathy,K. and Ella,K.M.
EPI_ISL_17454990, EPI_ISL_17454991	Bharat Biotech	Bharat Biotech	Murthy,E.K. and Sumathy,K.
EPI_ISL_17454992	Bharat Biotech	Bharat Biotech	Sumathy,K. and Ella,K.M.
EPI_ISL_17454993	Bharat Biotech	Bharat Biotech	ELLA,K.M. and KANDASWAMY,S.
EPI_ISL_17454994	Bharat Biotech	Bharat Biotech	Murthy,E.K. and Sumathy,K.
EPI_ISL_17454995, EPI_ISL_17454996, EPI_ISL_17454997	Bharat Biotech	Bharat Biotech	Sumathy,K. and Ella,K.M.
EPI_ISL_17454998	Bharat Biotech	Bharat Biotech	Murthy,E.K. and Sumathy,K.
EPI_ISL_17454999	Bharat Biotech	Bharat Biotech	Sumathy,K. and Ella,K.M.
EPI_ISL_17455000, EPI_ISL_17455001, EPI_ISL_17455002, EPI_ISL_17455003	Bharat Biotech	Bharat Biotech	Murthy,E.K. and Sumathy,K.
EPI_ISL_17455004	Bharat Biotech	Bharat Biotech	ELLA,K.M. and KANDASWAMY,S.
EPI_ISL_17455005, EPI_ISL_17455006	Bharathiar University, Department of Biotechnology / King Institute of Preventive Medicine and Research, Department of Virology	Bharathiar University, Department of Biotechnology / King Institute of Preventive Medicine and Research, Department of Virology	Saravanamurali,K., Sathishkumar,R., Gunasekaran,P., Kaveri,K., Prabhakaran,M., Varghese,I., Raja,A. and Senthilkumar,V.
EPI_ISL_17455007	Bharati Vidyapeeth Deemed University	Bharati Vidyapeeth Deemed University	Patil,H., Kulkarni,R. and Mhaske,S.
EPI_ISL_17455008, EPI_ISL_17455009	Bigtec Labs	Bigtec Labs	JAGANNATH MANJULA,[.I.N.]., NAIR CHANDRASEKHAR BHASKARAN,[.I.N.]. and SUBBARAO PILLARISETTI VENKATA,[.I.N.].
EPI_ISL_17455010	Bigtec Labs	Bigtec Labs	Subbarao,P.V., Jagannath,M. and Nair,C.B.
EPI_ISL_17455011	Bigtec Labs	Bigtec Labs	JAGANNATH MANJULA,[.I.N.]., NAIR CHANDRASEKHAR BHASKARAN,[.I.N.]. and SUBBARAO PILLARISETTI VENKATA,[.I.N.].
EPI_ISL_17455012	Bigtec Labs	Bigtec Labs	Subbarao,P.V., Jagannath,M. and Nair,C.B.
EPI_ISL_17455013	Bigtec Labs	Bigtec Labs	JAGANNATH MANJULA,[.I.N.]., NAIR CHANDRASEKHAR BHASKARAN,[.I.N.]. and SUBBARAO PILLARISETTI VENKATA,[.I.N.].
EPI_ISL_17455014, EPI_ISL_17455015	Bigtec Labs	Bigtec Labs	Nair,C.B., Jagannath,M. and Subbarao,P.V.
EPI_ISL_17455016	Bigtec Labs	Bigtec Labs	Subbarao,P.V., Jagannath,M. and Nair,C.B.
EPI_ISL_17455017, EPI_ISL_17455018, EPI_ISL_17455019	Bigtec Labs	Bigtec Labs	Nair,C.B., Jagannath,M. and Subbarao,P.V.
EPI_ISL_17455020	Bigtec Labs	Bigtec Labs	JAGANNATH MANJULA,[.I.N.]., NAIR CHANDRASEKHAR BHASKARAN,[.I.N.]. and SUBBARAO PILLARISETTI VENKATA,[.I.N.].
EPI_ISL_17455021, EPI_ISL_17455022, EPI_ISL_17455023	Bigtec Labs	Bigtec Labs	Subbarao,P.V., Jagannath,M. and Nair,C.B.
EPI_ISL_17455024	Bigtec Labs	Bigtec Labs	JAGANNATH MANJULA,[.I.N.]., NAIR CHANDRASEKHAR BHASKARAN,[.I.N.]. and SUBBARAO PILLARISETTI VENKATA,[.I.N.].
EPI_ISL_17455025	Bigtec Labs	Bigtec Labs	Nair,C.B., Jagannath,M. and Subbarao,P.V.
EPI_ISL_17455026, EPI_ISL_17455027	Bundeswehr Institute for Microbiology	Bundeswehr Institute for Microbiology	Hucke,F.I.L., Bestehorn-Willmann,M., Bassetto,M., Brancale,A., Zanetta,P. and Bugert,J.J.
EPI_ISL_17455028	Bundeswehr Institute of Microbiology	Bundeswehr Institute of Microbiology	Wolfel,S., Vollmar,P., Poluda,D., Zange,S., Antwerpen,M.H., Loscher,T. and Dobler,G.
EPI_ISL_17455029	Center for Disease Control and Prevention of Southern Theater	Center for Disease Control and Prevention of Southern Theater	Yin,X., Hu,T.S., Zhang,H., Liu,Y., Zhou,Z., Liu,L., Li,P., Wang,Y., Yang,Z., Yu,J., Chen,S. and Zhang,F.Q.
EPI_ISL_17455030	Center for Disease Control and Prevention of Southern Theater	Center for Disease Control and Prevention of Southern Theater	Hu,T.
EPI_ISL_17455031, EPI_ISL_17455032	Center for Disease Control and Prevention of Southern Theater	Center for Disease Control and Prevention of Southern Theater	Wu,D., Zhang,Y., Zhouhui,Q., Kou,J., Liang,W., Zhang,H., Monagin,C., Zhang,Q., Li,W., Zhong,H., He,J., Li,H., Cai,S., Ke,C. and Lin,J.
EPI_ISL_17455033, EPI_ISL_17455034, EPI_ISL_17455035	Center for Disease Control and Prevention of Southern Theater	Center for Disease Control and Prevention of Southern Theater	Hu,T.
EPI_ISL_17455036, EPI_ISL_17455037	Center for Disease Control and Prevention of Southern Theater	Center for Disease Control and Prevention of Southern Theater	Yin,X., Hu,T.S., Zhang,H., Liu,Y., Zhou,Z., Liu,L., Li,P., Wang,Y., Yang,Z., Yu,J., Chen,S. and Zhang,F.Q.
EPI_ISL_17455038, EPI_ISL_17455039	Center for Disease Control and Prevention of Southern Theater	Center for Disease Control and Prevention of Southern Theater	Hu,T.
EPI_ISL_17455040	Center for Disease Control and Prevention of Southern Theater	Center for Disease Control and Prevention of Southern Theater	Wu,D., Wu,J., Zhang,Q., Zhong,H., Ke,C., Deng,X., Guan,D., Li,H., Zhang,Y., Zhou,H., He,J., Li,L. and Yang,X.
EPI_ISL_17455041	Center for Disease Control and Prevention of Southern Theater	Center for Disease Control and Prevention of Southern Theater	Hu,T.
EPI_ISL_17455042	Center for Disease Control and Prevention of Southern Theater	Center for Disease Control and Prevention of Southern Theater	Wu,D., Wu,J., Zhang,Q., Zhong,H., Ke,C., Deng,X., Guan,D., Li,H., Zhang,Y., Zhou,H., He,J., Li,L. and Yang,X.
EPI_ISL_17455043, EPI_ISL_17455044, EPI_ISL_17455045, EPI_ISL_17455046	Center for Disease Control and Prevention of Southern Theater	Center for Disease Control and Prevention of Southern Theater	Hu,T.
EPI_ISL_17455047	Center for Disease Control and Prevention of Southern Theater	Center for Disease Control and Prevention of Southern Theater	Wu,D., Wu,J., Li,Z.Q., Jie,Z.H., Wen,K.C., Ling,D.X., Wei,G.D., Hui,L., Hui,Z.Y., Qiong,Z.H., Fen,H.J. and Fen,Y.X.
EPI_ISL_17455048, EPI_ISL_17455049, EPI_ISL_17455050	Center for Disease Control and Prevention of Southern Theater	Center for Disease Control and Prevention of Southern Theater	Hu,T.
EPI_ISL_17455051	Center for Disease Control and Prevention of Southern Theater	Center for Disease Control and Prevention of Southern Theater	Wu,D., Wu,J., Li,Z.Q., Jie,Z.H., Wen,K.C., Ling,D.X., Wei,G.D., Hui,L., Hui,Z.Y., Qiong,Z.H., Fen,H.J. and Fen,Y.X.
EPI_ISL_17455052, EPI_ISL_17455053, EPI_ISL_17455054, EPI_ISL_17455055, EPI_ISL_17455056, EPI_ISL_17455057, EPI_ISL_17455058, EPI_ISL_17455059, EPI_ISL_17455060, EPI_ISL_17455061, EPI_ISL_17455062, EPI_ISL_17455063			
see above	Center for Disease Control and Prevention of Southern Theater	Center for Disease Control and Prevention of Southern Theater	Hu,T.
EPI_ISL_17455064, EPI_ISL_17455065	Center for Disease Control and Prevention of Southern Theater	Center for Disease Control and Prevention of Southern Theater	Wu,D., Zhang,Y., Zhouhui,Q., Kou,J., Liang,W., Zhang,H., Monagin,C., Zhang,Q., Li,W., Zhong,H., He,J., Li,H., Cai,S., Ke,C. and Lin,J.
EPI_ISL_17455066, EPI_ISL_17455067	Center for Disease Control and Prevention of Southern Theater	Center for Disease Control and Prevention of Southern Theater	Wu,D., Wu,J., Li,Z.Q., Jie,Z.H., Wen,K.C., Ling,D.X., Wei,G.D., Hui,L., Hui,Z.Y., Qiong,Z.H., Fen,H.J. and Fen,Y.X.
EPI_ISL_17455068, EPI_ISL_17455069, EPI_ISL_17455070, EPI_ISL_17455071, EPI_ISL_17455072, EPI_ISL_17455073, EPI_ISL_17455074, EPI_ISL_17455075	Center for Disease Control and Prevention of Southern Theater	Center for Disease Control and Prevention of Southern Theater	Hu,T.
EPI_ISL_17455076, EPI_ISL_17455077, EPI_ISL_17455078, EPI_ISL_17455079, EPI_ISL_17455080, EPI_ISL_17455081, EPI_ISL_17455082, EPI_ISL_17455083, EPI_ISL_17455084, EPI_ISL_17455085, EPI_ISL_17455086, EPI_ISL_17455087, EPI_ISL_17455088, EPI_ISL_17455089, EPI_ISL_17455090, EPI_ISL_17455091, EPI_ISL_17455092, EPI_ISL_17455093, EPI_ISL_17455094, EPI_ISL_17455095, EPI_ISL_17455096, EPI_ISL_17455097, EPI_ISL_17455098, EPI_ISL_17455099, EPI_ISL_17455100, EPI_ISL_17455101, EPI_ISL_17455102, EPI_ISL_17455103, EPI_ISL_17455104, EPI_ISL_17455105, EPI_ISL_17455106, EPI_ISL_17455107, EPI_ISL_17455108, EPI_ISL_17455109, EPI_ISL_17455110, EPI_ISL_17455111, EPI_ISL_17455112, EPI_ISL_17455113, EPI_ISL_17455114, EPI_ISL_17455115, EPI_ISL_17455116, EPI_ISL_17455117, EPI_ISL_17455118, EPI_ISL_17455119, EPI_ISL_17455120, EPI_ISL_17455121, EPI_ISL_17455122, EPI_ISL_17455123, EPI_ISL_17455124, EPI_ISL_17455125, EPI_ISL_17455126, EPI_ISL_17455127, EPI_ISL_17455128, EPI_ISL_17455129, EPI_ISL_17455130, EPI_ISL_17455131, EPI_ISL_17455132, EPI_ISL_17455133, EPI_ISL_17455134, EPI_ISL_17455135, EPI_ISL_17455136, EPI_ISL_17455137, EPI_ISL_17455138, EPI_ISL_17455139, EPI_ISL_17455140, EPI_ISL_17455141, EPI_ISL_17455142, EPI_ISL_17455143, EPI_ISL_17455144, EPI_ISL_17455145, EPI_ISL_17455146, EPI_ISL_17455147, EPI_ISL_17455148, EPI_ISL_17455149, EPI_ISL_17455150, EPI_ISL_17455151, EPI_ISL_17455152, EPI_ISL_17455153, EPI_ISL_17455154, EPI_ISL_17455155, EPI_ISL_17455156, EPI_ISL_17455157, EPI_ISL_17455158, EPI_ISL_17455159, EPI_ISL_17455160, EPI_ISL_17455161, EPI_ISL_17455162, EPI_ISL_17455163, EPI_ISL_17455164, EPI_ISL_17455165, EPI_ISL_17455166, EPI_ISL_17455167, EPI_ISL_17455168, EPI_ISL_17455169, EPI_ISL_17455170, EPI_ISL_17455171, EPI_ISL_17455172, EPI_ISL_17455173, EPI_ISL_17455174, EPI_ISL_17455175, EPI_ISL_17455176, EPI_ISL_17455177, EPI_ISL_17455178, EPI_ISL_17455179, EPI_ISL_17455180, EPI_ISL_17455181, EPI_ISL_17455182, EPI_ISL_17455183, EPI_ISL_17455184, EPI_ISL_17455185, EPI_ISL_17455186, EPI_ISL_17455187, EPI_ISL_17455188, EPI_ISL_17455189, EPI_ISL_17455190, EPI_ISL_17455191, EPI_ISL_17455192, EPI_ISL_17455193, EPI_ISL_17455194, EPI_ISL_17455195, EPI_ISL_17455196, EPI_ISL_17455197, EPI_ISL_17455198, EPI_ISL_17455199, EPI_ISL_17455200, EPI_ISL_17455201, EPI_ISL_17455202, EPI_ISL_17455203, EPI_ISL_17455204, EPI_ISL_17455205, EPI_ISL_17455206, EPI_ISL_17455207, EPI_ISL_17455208, EPI_ISL_17455209, EPI_ISL_17455210, EPI_ISL_17455211, EPI_ISL_17455212, EPI_ISL_17455213, EPI_ISL_17455214, EPI_ISL_17455215, EPI_ISL_17455216, EPI_ISL_17455217, EPI_ISL_17455218, EPI_ISL_17455219, EPI_ISL_17455220, EPI_ISL_17455221, EPI_ISL_17455222, EPI_ISL_17455223, EPI_ISL_17455224, EPI_ISL_17455225, EPI_ISL_17455226, EPI_ISL_17455227, EPI_ISL_17455228, EPI_ISL_17455229, EPI_ISL_17455230, EPI_ISL_17455231, EPI_ISL_17455232, EPI_ISL_17455233, EPI_ISL_17455234, EPI_ISL_17455235, EPI_ISL_17455236, EPI_ISL_17455237, EPI_ISL_17455238, EPI_ISL_17455239, EPI_ISL_17455240, EPI_ISL_17455241, EPI_ISL_17455242, EPI_ISL_17455243, EPI_ISL_17455244, EPI_ISL_17455245, EPI_ISL_17455246, EPI_ISL_17455247, EPI_ISL_17455248, EPI_ISL_17455249			

see above	Center of Excellence in Clinical Virology, Faculty of Medical, Chulalongkorn University	Center of Excellence in Clinical Virology, Faculty of Medical, Chulalongkorn University	Chansaenroj,J., Khongwichit,S., Thongmee,T., Benjamanukul,S., Chirathaworn,C. and Poovorawan,Y.
EPI_ISL_17455250	Center of Excellence in Clinical Virology, Faculty of Medical, Chulalongkorn University	Center of Excellence in Clinical Virology, Faculty of Medical, Chulalongkorn University	Chansaenroj,J., Wanlapakorn,N., Ngamsaithong,C., Thongmee,T., Na Nakorn,N., Siriyasatien,P., Vongpunsawad,S. and Poovorawan,Y.
EPI_ISL_17455251, EPI_ISL_17455252, EPI_ISL_17455253, EPI_ISL_17455254, EPI_ISL_17455255, EPI_ISL_17455256, EPI_ISL_17455257, EPI_ISL_17455258, EPI_ISL_17455259, EPI_ISL_17455260, EPI_ISL_17455261, EPI_ISL_17455262, EPI_ISL_17455263, EPI_ISL_17455264, EPI_ISL_17455265, EPI_ISL_17455266, EPI_ISL_17455267, EPI_ISL_17455268, EPI_ISL_17455269, EPI_ISL_17455270, EPI_ISL_17455271, EPI_ISL_17455272, EPI_ISL_17455273, EPI_ISL_17455274, EPI_ISL_17455275, EPI_ISL_17455276, EPI_ISL_17455277, EPI_ISL_17455278, EPI_ISL_17455279, EPI_ISL_17455280, EPI_ISL_17455281, EPI_ISL_17455282, EPI_ISL_17455283, EPI_ISL_17455284, EPI_ISL_17455285, EPI_ISL_17455286, EPI_ISL_17455287, EPI_ISL_17455288, EPI_ISL_17455289, EPI_ISL_17455290, EPI_ISL_17455291, EPI_ISL_17455292, EPI_ISL_17455293, EPI_ISL_17455294, EPI_ISL_17455295, EPI_ISL_17455296, EPI_ISL_17455297, EPI_ISL_17455298, EPI_ISL_17455299, EPI_ISL_17455300, EPI_ISL_17455301, EPI_ISL_17455302			
see above	Center of Excellence in Clinical Virology, Faculty of Medical, Chulalongkorn University	Center of Excellence in Clinical Virology, Faculty of Medical, Chulalongkorn University	Chansaenroj,J., Khongwichit,S., Thongmee,T., Benjamanukul,S., Chirathaworn,C. and Poovorawan,Y.
EPI_ISL_17455303, EPI_ISL_17455304	Center of Excellence in Clinical Virology, Faculty of Medical, Chulalongkorn University	Center of Excellence in Clinical Virology, Faculty of Medical, Chulalongkorn University	Chansaenroj,J., Wanlapakorn,N., Ngamsaithong,C., Thongmee,T., Na Nakorn,N., Siriyasatien,P., Vongpunsawad,S. and Poovorawan,Y.
EPI_ISL_17455305, EPI_ISL_17455306, EPI_ISL_17455307, EPI_ISL_17455308, EPI_ISL_17455309, EPI_ISL_17455310, EPI_ISL_17455311, EPI_ISL_17455312, EPI_ISL_17455313, EPI_ISL_17455314, EPI_ISL_17455315, EPI_ISL_17455316, EPI_ISL_17455317, EPI_ISL_17455318, EPI_ISL_17455319, EPI_ISL_17455320, EPI_ISL_17455321, EPI_ISL_17455322, EPI_ISL_17455323, EPI_ISL_17455324, EPI_ISL_17455325, EPI_ISL_17455326, EPI_ISL_17455327, EPI_ISL_17455328, EPI_ISL_17455329			
see above	Center of Excellence in Clinical Virology, Faculty of Medical, Chulalongkorn University	Center of Excellence in Clinical Virology, Faculty of Medical, Chulalongkorn University	Chansaenroj,J., Khongwichit,S., Thongmee,T., Benjamanukul,S., Chirathaworn,C. and Poovorawan,Y.
EPI_ISL_17455330, EPI_ISL_17455331	Center of Excellence in Clinical Virology, Faculty of Medical, Chulalongkorn University	Center of Excellence in Clinical Virology, Faculty of Medical, Chulalongkorn University	Chansaenroj,J., Wanlapakorn,N., Ngamsaithong,C., Thongmee,T., Na Nakorn,N., Siriyasatien,P., Vongpunsawad,S. and Poovorawan,Y.
EPI_ISL_17455332, EPI_ISL_17455333, EPI_ISL_17455334	Center of Excellence in Clinical Virology, Faculty of Medical, Chulalongkorn University	Center of Excellence in Clinical Virology, Faculty of Medical, Chulalongkorn University	Chansaenroj,J., Khongwichit,S., Thongmee,T., Benjamanukul,S., Chirathaworn,C. and Poovorawan,Y.
EPI_ISL_17455335	Center of Excellence in Clinical Virology, Faculty of Medical, Chulalongkorn University	Center of Excellence in Clinical Virology, Faculty of Medical, Chulalongkorn University	Chansaenroj,J., Wanlapakorn,N., Ngamsaithong,C., Thongmee,T., Na Nakorn,N., Siriyasatien,P., Vongpunsawad,S. and Poovorawan,Y.
EPI_ISL_17455336, EPI_ISL_17455337, EPI_ISL_17455338, EPI_ISL_17455339, EPI_ISL_17455340, EPI_ISL_17455341, EPI_ISL_17455342, EPI_ISL_17455343, EPI_ISL_17455344, EPI_ISL_17455345, EPI_ISL_17455346, EPI_ISL_17455347			
see above	Center of Excellence in Clinical Virology, Faculty of Medical, Chulalongkorn University	Center of Excellence in Clinical Virology, Faculty of Medical, Chulalongkorn University	Chansaenroj,J., Khongwichit,S., Thongmee,T., Benjamanukul,S., Chirathaworn,C. and Poovorawan,Y.
EPI_ISL_17455348, EPI_ISL_17455349, EPI_ISL_17455350, EPI_ISL_17455351	Center of Excellence in Clinical Virology, Faculty of Medical, Chulalongkorn University	Center of Excellence in Clinical Virology, Faculty of Medical, Chulalongkorn University	Chansaenroj,J., Wanlapakorn,N., Ngamsaithong,C., Thongmee,T., Na Nakorn,N., Siriyasatien,P., Vongpunsawad,S. and Poovorawan,Y.
EPI_ISL_17455352	Center of Excellence in Clinical Virology, Faculty of Medical, Chulalongkorn University	Center of Excellence in Clinical Virology, Faculty of Medical, Chulalongkorn University	Chansaenroj,J., Khongwichit,S., Thongmee,T., Benjamanukul,S., Chirathaworn,C. and Poovorawan,Y.
EPI_ISL_17455353, EPI_ISL_17455354	Center of Excellence in Clinical Virology, Faculty of Medical, Chulalongkorn University	Center of Excellence in Clinical Virology, Faculty of Medical, Chulalongkorn University	Chansaenroj,J., Wanlapakorn,N., Ngamsaithong,C., Thongmee,T., Na Nakorn,N., Siriyasatien,P., Vongpunsawad,S. and Poovorawan,Y.
EPI_ISL_17455355, EPI_ISL_17455356, EPI_ISL_17455357, EPI_ISL_17455358, EPI_ISL_17455359, EPI_ISL_17455360, EPI_ISL_17455361, EPI_ISL_17455362, EPI_ISL_17455363, EPI_ISL_17455364, EPI_ISL_17455365, EPI_ISL_17455366, EPI_ISL_17455367, EPI_ISL_17455368, EPI_ISL_17455369, EPI_ISL_17455370, EPI_ISL_17455371, EPI_ISL_17455372, EPI_ISL_17455373, EPI_ISL_17455374, EPI_ISL_17455375, EPI_ISL_17455376, EPI_ISL_17455377, EPI_ISL_17455378, EPI_ISL_17455379, EPI_ISL_17455380, EPI_ISL_17455381, EPI_ISL_17455382, EPI_ISL_17455383, EPI_ISL_17455384, EPI_ISL_17455385, EPI_ISL_17455386, EPI_ISL_17455387, EPI_ISL_17455388, EPI_ISL_17455389, EPI_ISL_17455390, EPI_ISL_17455391, EPI_ISL_17455392, EPI_ISL_17455393, EPI_ISL_17455394, EPI_ISL_17455395, EPI_ISL_17455396, EPI_ISL_17455397, EPI_ISL_17455398, EPI_ISL_17455399, EPI_ISL_17455400, EPI_ISL_17455401, EPI_ISL_17455402, EPI_ISL_17455403, EPI_ISL_17455404, EPI_ISL_17455405, EPI_ISL_17455406, EPI_ISL_17455407, EPI_ISL_17455408, EPI_ISL_17455409, EPI_ISL_17455410, EPI_ISL_17455411, EPI_ISL_17455412, EPI_ISL_17455413, EPI_ISL_17455414, EPI_ISL_17455415, EPI_ISL_17455416, EPI_ISL_17455417, EPI_ISL_17455418, EPI_ISL_17455419, EPI_ISL_17455420, EPI_ISL_17455421, EPI_ISL_17455422, EPI_ISL_17455423, EPI_ISL_17455424, EPI_ISL_17455425, EPI_ISL_17455426, EPI_ISL_17455427, EPI_ISL_17455428, EPI_ISL_17455429, EPI_ISL_17455430, EPI_ISL_17455431, EPI_ISL_17455432, EPI_ISL_17455433, EPI_ISL_17455434, EPI_ISL_17455435, EPI_ISL_17455436, EPI_ISL_17455437, EPI_ISL_17455438, EPI_ISL_17455439, EPI_ISL_17455440, EPI_ISL_17455441, EPI_ISL_17455442, EPI_ISL_17455443			
see above	Center of Excellence in Clinical Virology, Faculty of Medical, Chulalongkorn University	Center of Excellence in Clinical Virology, Faculty of Medical, Chulalongkorn University	Chansaenroj,J., Khongwichit,S., Thongmee,T., Benjamanukul,S., Chirathaworn,C. and Poovorawan,Y.
EPI_ISL_17455444, EPI_ISL_17455445, EPI_ISL_17455446, EPI_ISL_17455447	Centers for Disease Control and Prevention	Centers for Disease Control and Prevention	Lanciotti,R.S.
EPI_ISL_17455448, EPI_ISL_17455449	Centers for Disease Control and Prevention	Centers for Disease Control and Prevention	Lanciotti,R.S., Kosoy,O.L., Laven,J.J., Panella,A.J., Velez,J.O., Lambert,A.J. and Campbell,G.L.
EPI_ISL_17455450, EPI_ISL_17455451, EPI_ISL_17455452, EPI_ISL_17455453, EPI_ISL_17455454, EPI_ISL_17455455	Centers for Disease Control and Prevention	Centers for Disease Control and Prevention	Lanciotti,R.S.
EPI_ISL_17455456	Centers for Disease Control and Prevention	Centers for Disease Control and Prevention	Lanciotti,R.S., Kosoy,O.L., Laven,J.J., Panella,A.J., Velez,J.O., Lambert,A.J. and Campbell,G.L.
EPI_ISL_17455457	Centers for Disease Control and Prevention	Centers for Disease Control and Prevention	Kinney,R.M. and Pfeffer,M.
EPI_ISL_17455458, EPI_ISL_17455459, EPI_ISL_17455460, EPI_ISL_17455461	Centers for Disease Control and Prevention	Centers for Disease Control and Prevention	Lanciotti,R.S.
EPI_ISL_17455462	Centers for Disease Control and Prevention	Centers for Disease Control and Prevention	Lanciotti,R.S., Kosoy,O.L., Laven,J.J., Panella,A.J., Velez,J.O., Lambert,A.J. and Campbell,G.L.
EPI_ISL_17455463, EPI_ISL_17455464, EPI_ISL_17455465	Centers for Disease Control and Prevention	Centers for Disease Control and Prevention	Lanciotti,R.S.
EPI_ISL_17455466	Centers for Disease Control and Prevention	Centers for Disease Control and Prevention	Lanciotti,R.S., Kosoy,O.L., Laven,J.J., Panella,A.J., Velez,J.O., Lambert,A.J. and Campbell,G.L.
EPI_ISL_17455467	Centers for Disease Control and Prevention	Centers for Disease Control and Prevention	Lanciotti,R.S.
EPI_ISL_17455468	Centers for Disease Control and Prevention	Centers for Disease Control and Prevention	Lanciotti,R.S., Kosoy,O.L., Laven,J.J., Panella,A.J., Velez,J.O., Lambert,A.J. and Campbell,G.L.
EPI_ISL_17455469, EPI_ISL_17455470, EPI_ISL_17455471, EPI_ISL_17455472	Centers for Disease Control and Prevention	Centers for Disease Control and Prevention	Lanciotti,R.S.
EPI_ISL_17455473	Centers for Disease Control and Prevention	Centers for Disease Control and Prevention	Lanciotti,R.S., Kosoy,O.L., Laven,J.J., Panella,A.J., Velez,J.O., Lambert,A.J. and Campbell,G.L.
EPI_ISL_17455474, EPI_ISL_17455475, EPI_ISL_17455476, EPI_ISL_17455477, EPI_ISL_17455478, EPI_ISL_17455479, EPI_ISL_17455480	Centers for Disease Control and Prevention	Centers for Disease Control and Prevention	Lanciotti,R.S.
EPI_ISL_17455481	Centers for Disease Control and Prevention	Centers for Disease Control and Prevention	Lanciotti,R.S., Kosoy,O.L., Laven,J.J., Panella,A.J., Velez,J.O., Lambert,A.J. and Campbell,G.L.
EPI_ISL_17455482, EPI_ISL_17455483	Centers for Disease Control and Prevention, Division of Vector-borne Diseases	Centers for Disease Control and Prevention, Division of Vector-borne Diseases	Savage,H.M., Ledermann,J.P., Yug,L., Burkhalter,K.L., Marfel,M. and Hancock,W.T.
EPI_ISL_17455484, EPI_ISL_17455485, EPI_ISL_17455486	Centre International de Recherches Médicales de Franceville	Centre International de Recherches Médicales de Franceville	Caron,M., Paupy,C., Grard,G., Becquart,P., Mombo,I., Nso,B.B., Kassa Kassa,F., Nkoghe,D. and Leroy,E.M.
EPI_ISL_17455487, EPI_ISL_17455488, EPI_ISL_17455489	Centre Pasteur du Cameroun	Centre Pasteur du Cameroun	Demanou,M., Sadeuh-Mba,S.A., Vanhecke,C., Ndikweti,R., Kouna Tsala,I., Inais Nsizoa,M. and Njouom,R.
EPI_ISL_17455490, EPI_ISL_17455491	Centre Pasteur du Cameroun	Centre Pasteur du Cameroun	Yonga Wansi,G.M.
EPI_ISL_17455492, EPI_ISL_17455493, EPI_ISL_17455494, EPI_ISL_17455495, EPI_ISL_17455496, EPI_ISL_17455497	Centre for Emergency Preparedness and Response	Centre for Emergency Preparedness and Response	Edwards,C.J., Welch,S.R., Chamberlain,J., Hewson,R., Tolley,H., Cane,P.A. and Lloyd,G.
EPI_ISL_17455498	Centre for Emergency Preparedness and Response	Centre for Emergency Preparedness and Response	Lewthwaite,P., Vasanthapuram,R., Osborne,J.C., Begum,A., Plank,J.L.M., Shankar,M.V., Hewson,R., Desai,A., Beeching,N.J., Ravikumar,R. and Solomon,T.
EPI_ISL_17455499	Centre for Emergency Preparedness and Response	Centre for Emergency Preparedness and Response	Edwards,C.J., Welch,S.R., Chamberlain,J., Hewson,R., Tolley,H., Cane,P.A. and Lloyd,G.
EPI_ISL_17455500	Centre for Emergency Preparedness and Response	Centre for Emergency Preparedness and Response	Lewthwaite,P., Vasanthapuram,R., Osborne,J.C., Begum,A., Plank,J.L.M., Shankar,M.V., Hewson,R., Desai,A., Beeching,N.J., Ravikumar,R. and Solomon,T.
EPI_ISL_17455501	Centre for Emergency Preparedness and Response	Centre for Emergency Preparedness and Response	Chamberlain,J. and Hewson,R.
EPI_ISL_17455502	Centre for Emergency Preparedness and Response	Centre for Emergency Preparedness and Response	Lewthwaite,P., Vasanthapuram,R., Osborne,J.C., Begum,A., Plank,J.L.M., Shankar,M.V., Hewson,R., Desai,A., Beeching,N.J., Ravikumar,R. and Solomon,T.
EPI_ISL_17455503	Centre for Emergency Preparedness and Response	Centre for Emergency Preparedness and Response	Edwards,C.J., Welch,S.R., Chamberlain,J., Hewson,R., Tolley,H., Cane,P.A. and Lloyd,G.
EPI_ISL_17455504, EPI_ISL_17455505	Centre for Emergency Preparedness and Response	Centre for Emergency Preparedness and Response	Lewthwaite,P., Vasanthapuram,R., Osborne,J.C., Begum,A., Plank,J.L.M., Shankar,M.V., Hewson,R., Desai,A., Beeching,N.J., Ravikumar,R. and Solomon,T.
EPI_ISL_17455506	Centre for Emergency Preparedness and Response	Centre for Emergency Preparedness and Response	Edwards,C.J., Welch,S.R., Chamberlain,J., Hewson,R., Tolley,H., Cane,P.A. and Lloyd,G.
EPI_ISL_17455507	Centre for Emergency Preparedness and Response	Centre for Emergency Preparedness and Response	Lewthwaite,P., Vasanthapuram,R., Osborne,J.C., Begum,A., Plank,J.L.M., Shankar,M.V., Hewson,R., Desai,A., Beeching,N.J., Ravikumar,R. and Solomon,T.
EPI_ISL_17455508	Centre for Health Protection	Centre for Health Protection	Cheng,P.K.C. and Lim,W.W.L.
EPI_ISL_17455509	Centre for Interdisciplinary Research in Basic Sciences, Jamia Millia Islamia	Centre for Interdisciplinary Research in Basic Sciences, Jamia Millia Islamia	Afreen,N., Deeba,F., Khan,W.H., Haider,S.H., Kazim,S.N., Ishrat,R., Naqvi,I.H., Shareef,M.Y., Broor,S., Ahmed,A. and Parveen,S.
EPI_ISL_17455510, EPI_ISL_17455511	Centre for Interdisciplinary Research in Basic	Centre for Interdisciplinary Research in Basic	Hisamuddin,M., Tazeen,A., Abdullah,M., Islamuddin,M., Parveen,N., Islam,A., Faizan,M.I., Hamza,A., Naqvi,I.H., Verma,H.N., Malik,A., Ahmed,A. and Parveen,S.

	Sciences, Jamia Millia Islamia	Sciences, Jamia Millia Islamia	
EPI_ISL_17455512, EPI_ISL_17455513, EPI_ISL_17455514	Centre for Interdisciplinary Research in Basic Sciences, Jamia Millia Islamia	Centre for Interdisciplinary Research in Basic Sciences, Jamia Millia Islamia	Afreen,N., Deeba,F., Khan,W.H., Haider,S.H., Kazim,S.N., Ishrat,R., Naqvi,I.H., Shareef,M.Y., Broor,S., Ahmed,A. and Parveen,S.
EPI_ISL_17455515, EPI_ISL_17455516, EPI_ISL_17455517	Centre for Interdisciplinary Research in Basic Sciences, Jamia Millia Islamia	Centre for Interdisciplinary Research in Basic Sciences, Jamia Millia Islamia	Hisamuddin,M., Tazeen,A., Abdullah,M., Islamuddin,M., Parveen,N., Islam,A., Faizan,M.I., Hamza,A., Naqvi,I.H., Verma,H.N., Malik,A., Ahmed,A. and Parveen,S.
EPI_ISL_17455518	Centre for Interdisciplinary Research in Basic Sciences, Jamia Millia Islamia	Centre for Interdisciplinary Research in Basic Sciences, Jamia Millia Islamia	Afreen,N., Deeba,F., Khan,W.H., Haider,S.H., Kazim,S.N., Ishrat,R., Naqvi,I.H., Shareef,M.Y., Broor,S., Ahmed,A. and Parveen,S.
EPI_ISL_17455519	Centre for Interdisciplinary Research in Basic Sciences, Jamia Millia Islamia	Centre for Interdisciplinary Research in Basic Sciences, Jamia Millia Islamia	Hisamuddin,M., Tazeen,A., Abdullah,M., Islamuddin,M., Parveen,N., Islam,A., Faizan,M.I., Hamza,A., Naqvi,I.H., Verma,H.N., Malik,A., Ahmed,A. and Parveen,S.
EPI_ISL_17455520, EPI_ISL_17455521, EPI_ISL_17455522, EPI_ISL_17455523	Centre for Interdisciplinary Research in Basic Sciences, Jamia Millia Islamia	Centre for Interdisciplinary Research in Basic Sciences, Jamia Millia Islamia	Afreen,N., Deeba,F., Khan,W.H., Haider,S.H., Kazim,S.N., Ishrat,R., Naqvi,I.H., Shareef,M.Y., Broor,S., Ahmed,A. and Parveen,S.
EPI_ISL_17455524	Centre for Interdisciplinary Research in Basic Sciences, Jamia Millia Islamia	Centre for Interdisciplinary Research in Basic Sciences, Jamia Millia Islamia	Hisamuddin,M., Tazeen,A., Abdullah,M., Islamuddin,M., Parveen,N., Islam,A., Faizan,M.I., Hamza,A., Naqvi,I.H., Verma,H.N., Malik,A., Ahmed,A. and Parveen,S.
EPI_ISL_17455525	Centre for Interdisciplinary Research in Basic Sciences, Jamia Millia Islamia	Centre for Interdisciplinary Research in Basic Sciences, Jamia Millia Islamia	Afreen,N., Deeba,F., Khan,W.H., Haider,S.H., Kazim,S.N., Ishrat,R., Naqvi,I.H., Shareef,M.Y., Broor,S., Ahmed,A. and Parveen,S.
EPI_ISL_17455526, EPI_ISL_17455527	Centre for Interdisciplinary Research in Basic Sciences, Jamia Millia Islamia	Centre for Interdisciplinary Research in Basic Sciences, Jamia Millia Islamia	Hisamuddin,M., Tazeen,A., Abdullah,M., Islamuddin,M., Parveen,N., Islam,A., Faizan,M.I., Hamza,A., Naqvi,I.H., Verma,H.N., Malik,A., Ahmed,A. and Parveen,S.
EPI_ISL_17455528, EPI_ISL_17455529, EPI_ISL_17455530, EPI_ISL_17455531, EPI_ISL_17455532	Centre for Interdisciplinary Research in Basic Sciences, Jamia Millia Islamia	Centre for Interdisciplinary Research in Basic Sciences, Jamia Millia Islamia	Afreen,N., Deeba,F., Khan,W.H., Haider,S.H., Kazim,S.N., Ishrat,R., Naqvi,I.H., Shareef,M.Y., Broor,S., Ahmed,A. and Parveen,S.
EPI_ISL_17455533, EPI_ISL_17455534	Centre for Interdisciplinary Research in Basic Sciences, Jamia Millia Islamia	Centre for Interdisciplinary Research in Basic Sciences, Jamia Millia Islamia	Hisamuddin,M., Tazeen,A., Abdullah,M., Islamuddin,M., Parveen,N., Islam,A., Faizan,M.I., Hamza,A., Naqvi,I.H., Verma,H.N., Malik,A., Ahmed,A. and Parveen,S.
EPI_ISL_17455535, EPI_ISL_17455536, EPI_ISL_17455537, EPI_ISL_17455538, EPI_ISL_17455539	Centre for Interdisciplinary Research in Basic Sciences, Jamia Millia Islamia	Centre for Interdisciplinary Research in Basic Sciences, Jamia Millia Islamia	Afreen,N., Deeba,F., Khan,W.H., Haider,S.H., Kazim,S.N., Ishrat,R., Naqvi,I.H., Shareef,M.Y., Broor,S., Ahmed,A. and Parveen,S.
EPI_ISL_17455540, EPI_ISL_17455541, EPI_ISL_17455542	Indian Council of Medical Research (ICMR), Centre for Research in Medical Entomology	Indian Council of Medical Research (ICMR), Centre for Research in Medical Entomology	Paramasivan,R., Philip Samuel,P., Thenmozhi,V., Rajendran,R., Victor Jerald Leo,S., Dhananjeyan,K.J., Krishnamoorthi,R., Arunachalam,N. and Tyagi,B.K.
EPI_ISL_17455543, EPI_ISL_17455544, EPI_ISL_17455545	Centro de Biotecnologia Genomica del Instituto Politecnico Nacional, Laboratorio de Biomedicina	Centro de Biotecnologia Genomica del Instituto Politecnico Nacional, Laboratorio de Biomedicina	Laredo-Tiscareno,S.V., Machain-Williams,C., Rodriguez-Perez,M.A., Garza-Hernandez,J.A., Doria-Cobos,G.L., Cetina-Trejo,R.C., Bacab-Cab,L.A., Tangudu,C.S., Charles,J., De Luna-Santillana,E.J., Garcia-Rejon,J.E. and Blitvich,B.J.
EPI_ISL_17455546	Centro de Investigación y de Estudios Avanzados	Centro de Investigación y de Estudios Avanzados	Mussaret Bano,Z., Garcia Cordero,J., Rivero-Gomez,R., Corzo-Gomez,J., Gonzalez y Almeida,M.E., Bonilla-Moreno,R., Villegas Sepulveda,N. and Cedillo Barron,L.
EPI_ISL_17455547, EPI_ISL_17455548, EPI_ISL_17455549, EPI_ISL_17455550, EPI_ISL_17455551, EPI_ISL_17455552, EPI_ISL_17455553	Chiba University	Chiba University	Ido,E. and Taty Taty,R.
EPI_ISL_17455554	Chinese Academy of Medical Sciences	Chinese Academy of Medical Sciences	Liu,C., Hu,Y., Hu,N., Shi,J. and Guo,X.
EPI_ISL_17455555, EPI_ISL_17455556, EPI_ISL_17455557, EPI_ISL_17455558, EPI_ISL_17455559, EPI_ISL_17455560, EPI_ISL_17455561, EPI_ISL_17455562	Chinese Academy of Medical Sciences & Peking Union Medical College	Chinese Academy of Medical Sciences & Peking Union Medical College	Liu,H., Liu,J., Su,C. and Zou,M.
EPI_ISL_17455563, EPI_ISL_17455564, EPI_ISL_17455565, EPI_ISL_17455566, EPI_ISL_17455567, EPI_ISL_17455568, EPI_ISL_17455569, EPI_ISL_17455570	Chinese Academy of Sciences, Wuhan Institute of Virology, Key Laboratory of Special Pathogens and Biosafety, Center for Emerging Infectious Diseases,	Chinese Academy of Sciences, Wuhan Institute of Virology, Key Laboratory of Special Pathogens and Biosafety, Center for Emerging Infectious Diseases,	Liu,S.Q., Li,X., Zhang,Y.N., Gao,A.L., Deng,C.L., Li,J.H., Jehan,S., Jamil,N., Deng,F., Wei,H. and Zhang,B.
EPI_ISL_17455571, EPI_ISL_17455572, EPI_ISL_17455573, EPI_ISL_17455574, EPI_ISL_17455575, EPI_ISL_17455576, EPI_ISL_17455577	Chulalongkorn University	Chulalongkorn University	Rianthavorn,P., Prianantathavorn,K., Wuttirattanakowit,N., Theamboonlers,A. and Poovorawan,Y.
EPI_ISL_17455578	Chulalongkorn University	Chulalongkorn University	Khongwichit,S., Chansaenroj,J., Thongmee,T., Vongpunsawad,S. and Poovorawan,Y.
EPI_ISL_17455579	Chulalongkorn University	Chulalongkorn University	Rianthavorn,P., Prianantathavorn,K., Wuttirattanakowit,N., Theamboonlers,A. and Poovorawan,Y.
EPI_ISL_17455580, EPI_ISL_17455581	Chulalongkorn University	Chulalongkorn University	Khongwichit,S., Chansaenroj,J., Thongmee,T., Wanlapakorn,N. and Poovorawan,Y.
EPI_ISL_17455582	Chulalongkorn University	Chulalongkorn University	Vongpunsawad,S., Intharasongkroh,D., Chansaenroj,J. and Poovorawan,Y.
EPI_ISL_17455583, EPI_ISL_17455584, EPI_ISL_17455585	Chulalongkorn University	Chulalongkorn University	Rianthavorn,P., Prianantathavorn,K., Wuttirattanakowit,N., Theamboonlers,A. and Poovorawan,Y.
EPI_ISL_17455586	Chulalongkorn University	Chulalongkorn University	Vongpunsawad,S., Intharasongkroh,D., Chansaenroj,J. and Poovorawan,Y.
EPI_ISL_17455587, EPI_ISL_17455588, EPI_ISL_17455589, EPI_ISL_17455590	Chulalongkorn University	Chulalongkorn University	Rianthavorn,P., Prianantathavorn,K., Wuttirattanakowit,N., Theamboonlers,A. and Poovorawan,Y.
EPI_ISL_17455591	Chulalongkorn University	Chulalongkorn University	Pongsiri,P., Auksornkitti,V., Theamboonlers,A., Luplertlop,N., Rianthavorn,P. and Poovorawan,Y.
EPI_ISL_17455592	Chulalongkorn University	Chulalongkorn University	Rianthavorn,P., Prianantathavorn,K., Wuttirattanakowit,N., Theamboonlers,A. and Poovorawan,Y.
EPI_ISL_17455593	Chulalongkorn University	Chulalongkorn University	Auksornkitti,V., Pongsiri,P., Theamboonlers,A., Rianthavorn,P., Poovorawan,Y., Manujum,K. and Luplertlop,N.
EPI_ISL_17455594	Chulalongkorn University	Chulalongkorn University	Praianantathavorn,K., Vichaiwattana,P., Theamboonlers,A., Rianthavorn,P., Wuttirattanakowit,N. and Poovorawan,Y.
EPI_ISL_17455595	Chulalongkorn University	Chulalongkorn University	Rianthavorn,P., Prianantathavorn,K., Wuttirattanakowit,N., Theamboonlers,A. and Poovorawan,Y.
EPI_ISL_17455596, EPI_ISL_17455597	Chulalongkorn University	Chulalongkorn University	Praianantathavorn,K., Vichaiwattana,P., Theamboonlers,A., Rianthavorn,P., Wuttirattanakowit,N. and Poovorawan,Y.
EPI_ISL_17455598, EPI_ISL_17455599	Chulalongkorn University	Chulalongkorn University	Pongsiri,P., Auksornkitti,V., Theamboonlers,A., Luplertlop,N., Rianthavorn,P. and Poovorawan,Y.
EPI_ISL_17455600, EPI_ISL_17455601, EPI_ISL_17455602, EPI_ISL_17455603	Chulalongkorn University	Chulalongkorn University	Rianthavorn,P., Prianantathavorn,K., Wuttirattanakowit,N., Theamboonlers,A. and Poovorawan,Y.
EPI_ISL_17455604	Chulalongkorn University	Chulalongkorn University	Khongwichit,S., Chansaenroj,J., Thongmee,T., Wanlapakorn,N. and Poovorawan,Y.
EPI_ISL_17455605, EPI_ISL_17455606, EPI_ISL_17455607, EPI_ISL_17455608, EPI_ISL_17455609, EPI_ISL_17455610, EPI_ISL_17455611, EPI_ISL_17455612	Chulalongkorn University	Chulalongkorn University	Rianthavorn,P., Prianantathavorn,K., Wuttirattanakowit,N., Theamboonlers,A. and Poovorawan,Y.
EPI_ISL_17455613, EPI_ISL_17455614	Chulalongkorn University	Chulalongkorn University	Vongpunsawad,S., Intharasongkroh,D., Chansaenroj,J. and Poovorawan,Y.
EPI_ISL_17455615, EPI_ISL_17455616, EPI_ISL_17455617, EPI_ISL_17455618	Chulalongkorn University	Chulalongkorn University	Rianthavorn,P., Prianantathavorn,K., Wuttirattanakowit,N., Theamboonlers,A. and Poovorawan,Y.
EPI_ISL_17455619	Chulalongkorn University	Chulalongkorn University	Theamboonlers,A., Praianantathavorn,K., Thongmee,C., Vichaiwattana,P., Wutthirattanakowit,N. and Poovorawan,Y.
EPI_ISL_17455620	Chulalongkorn University	Chulalongkorn University	Rianthavorn,P., Prianantathavorn,K., Wuttirattanakowit,N., Theamboonlers,A. and Poovorawan,Y.
EPI_ISL_17455621	Chulalongkorn University	Chulalongkorn University	Theamboonlers,A., Praianantathavorn,K., Thongmee,C., Vichaiwattana,P., Wutthirattanakowit,N. and Poovorawan,Y.
EPI_ISL_17455622	Chulalongkorn University	Chulalongkorn University	Kittichai,V., Montriwat,P., Basanayake,C., Chen,J.-H., Thavara,U., Tawatsin,A. and Siriyasatien,P.
EPI_ISL_17455623, EPI_ISL_17455624	Chulalongkorn University	Chulalongkorn University	Rianthavorn,P., Prianantathavorn,K., Wuttirattanakowit,N., Theamboonlers,A. and Poovorawan,Y.
EPI_ISL_17455625	Chulalongkorn University	Chulalongkorn University	Kittichai,V., Montriwat,P., Basanayake,C., Chen,J.-H., Thavara,U., Tawatsin,A. and Siriyasatien,P.
EPI_ISL_17455626	Chulalongkorn University	Chulalongkorn University	Theamboonlers,A., Praianantathavorn,K., Thongmee,C., Vichaiwattana,P., Wutthirattanakowit,N. and Poovorawan,Y.
EPI_ISL_17455627, EPI_ISL_17455628	Chulalongkorn University	Chulalongkorn University	Rianthavorn,P., Prianantathavorn,K., Wuttirattanakowit,N., Theamboonlers,A. and Poovorawan,Y.
EPI_ISL_17455629, EPI_ISL_17455630, EPI_ISL_17455631, EPI_ISL_17455632, EPI_ISL_17455633	Chulalongkorn University	Chulalongkorn University	Kittichai,V., Montriwat,P., Basanayake,C., Chen,J.-H., Thavara,U., Tawatsin,A. and Siriyasatien,P.
EPI_ISL_17455634, EPI_ISL_17455635	Chulalongkorn University	Chulalongkorn University	Rianthavorn,P., Prianantathavorn,K., Wuttirattanakowit,N., Theamboonlers,A. and Poovorawan,Y.
EPI_ISL_17455636	Chulalongkorn University	Chulalongkorn University	Kittichai,V., Montriwat,P., Basanayake,C., Chen,J.-H., Thavara,U., Tawatsin,A. and Siriyasatien,P.
EPI_ISL_17455637	Chulalongkorn University	Chulalongkorn University	Pongsiri,P., Auksornkitti,V., Theamboonlers,A., Luplertlop,N., Rianthavorn,P. and Poovorawan,Y.
EPI_ISL_17455638	Chulalongkorn University	Chulalongkorn University	Praianantathavorn,K., Vichaiwattana,P., Theamboonlers,A., Rianthavorn,P., Wuttirattanakowit,N. and Poovorawan,Y.
EPI_ISL_17455639, EPI_ISL_17455640, EPI_ISL_17455641, EPI_ISL_17455642	Chulalongkorn University	Chulalongkorn University	Kittichai,V., Montriwat,P., Basanayake,C., Chen,J.-H., Thavara,U., Tawatsin,A. and Siriyasatien,P.
EPI_ISL_17455643	Chulalongkorn University	Chulalongkorn University	Rianthavorn,P., Prianantathavorn,K., Wuttirattanakowit,N., Theamboonlers,A. and Poovorawan,Y.
EPI_ISL_17455644	Chulalongkorn University	Chulalongkorn University	Kittichai,V., Montriwat,P., Basanayake,C., Chen,J.-H., Thavara,U., Tawatsin,A. and Siriyasatien,P.
EPI_ISL_17455645	Chulalongkorn University	Chulalongkorn University	Wanlapakorn,N., Thongmee,T., Linsuwanon,P., Chattakul,P., Vongpunsawad,S., Payungporn,S. and Poovorawan,Y.

EPI_ISL_17455646	Chulalongkorn University	Chulalongkorn University	Theamboonlers,A., Praianantathavorn,K., Thongmee,C., Vichaiwattana,P., Wutthirattanakowit,N. and Poovorawan,Y.
EPI_ISL_17455647	Chulalongkorn University	Chulalongkorn University	Wanlapakorn,N., Thongmee,T., Linsuwanon,P., Chattakul,P., Vongpunsawad,S., Payungporn,S. and Poovorawan,Y.
EPI_ISL_17455648	Chulalongkorn University	Chulalongkorn University	Theamboonlers,A., Praianantathavorn,K., Thongmee,C., Vichaiwattana,P., Wutthirattanakowit,N. and Poovorawan,Y.
EPI_ISL_17455649	Chulalongkorn University	Chulalongkorn University	Pongsiri,P., Theamboonlers,A. and Poovorawan,Y.
EPI_ISL_17455650	Chulalongkorn University	Chulalongkorn University	Wanlapakorn,N., Thongmee,T., Linsuwanon,P., Chattakul,P., Vongpunsawad,S., Payungporn,S. and Poovorawan,Y.
EPI_ISL_17455651, EPI_ISL_17455652, EPI_ISL_17455653, EPI_ISL_17455654	Chulalongkorn University	Chulalongkorn University	Kittichai,V., Montriwat,P., Basanayake,C., Chen,J.-H., Thavara,U., Tawatsin,A. and Siriyasatien,P.
EPI_ISL_17455655	Chulalongkorn University	Chulalongkorn University	Wanlapakorn,N., Thongmee,T., Linsuwanon,P., Chattakul,P., Vongpunsawad,S., Payungporn,S. and Poovorawan,Y.
EPI_ISL_17455656	Chulalongkorn University	Chulalongkorn University	Praianantathavorn,K., Vichaiwattana,P., Theamboonlers,A., Rianthavorn,P., Wuttirattanakowit,N. and Poovorawan,Y.
EPI_ISL_17455657	Chulalongkorn University	Chulalongkorn University	Rianthavorn,P., Prianantathavorn,K., Wuttirattanakowit,N., Theamboonlers,A. and Poovorawan,Y.
EPI_ISL_17455658	Chulalongkorn University	Chulalongkorn University	Kittichai,V., Montriwat,P., Basanayake,C., Chen,J.-H., Thavara,U., Tawatsin,A. and Siriyasatien,P.
EPI_ISL_17455659, EPI_ISL_17455660, EPI_ISL_17455661, EPI_ISL_17455662, EPI_ISL_17455663, EPI_ISL_17455664, EPI_ISL_17455665, EPI_ISL_17455666, EPI_ISL_17455667	Chulalongkorn University		Rianthavorn,P., Prianantathavorn,K., Wuttirattanakowit,N., Theamboonlers,A. and Poovorawan,Y.
EPI_ISL_17455668	Chulalongkorn University	Chulalongkorn University	Vongpunsawad,S., Intharasongkroh,D., Chansaenroj,J. and Poovorawan,Y.
EPI_ISL_17455669, EPI_ISL_17455670, EPI_ISL_17455671, EPI_ISL_17455672, EPI_ISL_17455673, EPI_ISL_17455674, EPI_ISL_17455675, EPI_ISL_17455676, EPI_ISL_17455677, EPI_ISL_17455678	Chulalongkorn University	Chulalongkorn University	Rianthavorn,P., Prianantathavorn,K., Wuttirattanakowit,N., Theamboonlers,A. and Poovorawan,Y.
EPI_ISL_17455679, EPI_ISL_17455680	Chulalongkorn University	Chulalongkorn University	Kittichai,V., Montriwat,P., Basanayake,C., Chen,J.-H., Thavara,U., Tawatsin,A. and Siriyasatien,P.
EPI_ISL_17455681	Chulalongkorn University	Chulalongkorn University	Rianthavorn,P., Prianantathavorn,K., Wuttirattanakowit,N., Theamboonlers,A. and Poovorawan,Y.
EPI_ISL_17455682, EPI_ISL_17455683	Chulalongkorn University	Chulalongkorn University	Praianantathavorn,K., Vichaiwattana,P., Theamboonlers,A., Rianthavorn,P., Wuttirattanakowit,N. and Poovorawan,Y.
EPI_ISL_17455684, EPI_ISL_17455685	Chulalongkorn University	Chulalongkorn University	Rianthavorn,P., Prianantathavorn,K., Wuttirattanakowit,N., Theamboonlers,A. and Poovorawan,Y.
EPI_ISL_17455686	Chulalongkorn University	Chulalongkorn University	Praianantathavorn,K., Vichaiwattana,P., Theamboonlers,A., Rianthavorn,P., Wuttirattanakowit,N. and Poovorawan,Y.
EPI_ISL_17455687	Chulalongkorn University	Chulalongkorn University	Rianthavorn,P., Prianantathavorn,K., Wuttirattanakowit,N., Theamboonlers,A. and Poovorawan,Y.
EPI_ISL_17455688	Chulalongkorn University	Chulalongkorn University	Praianantathavorn,K., Vichaiwattana,P., Theamboonlers,A., Rianthavorn,P., Wuttirattanakowit,N. and Poovorawan,Y.
EPI_ISL_17455689	Chulalongkorn University	Chulalongkorn University	Rianthavorn,P., Prianantathavorn,K., Wuttirattanakowit,N., Theamboonlers,A. and Poovorawan,Y.
EPI_ISL_17455690, EPI_ISL_17455691, EPI_ISL_17455692, EPI_ISL_17455693, EPI_ISL_17455694, EPI_ISL_17455695, EPI_ISL_17455696, EPI_ISL_17455697	Chulalongkorn University	Chulalongkorn University	Praianantathavorn,K., Vichaiwattana,P., Theamboonlers,A., Rianthavorn,P., Wuttirattanakowit,N. and Poovorawan,Y.
EPI_ISL_17455698, EPI_ISL_17455699, EPI_ISL_17455700, EPI_ISL_17455701, EPI_ISL_17455702, EPI_ISL_17455703, EPI_ISL_17455704, EPI_ISL_17455705, EPI_ISL_17455706, EPI_ISL_17455707, EPI_ISL_17455708, EPI_ISL_17455709, EPI_ISL_17455710, EPI_ISL_17455711, EPI_ISL_17455712, EPI_ISL_17455713, EPI_ISL_17455714, EPI_ISL_17455715, EPI_ISL_17455716, EPI_ISL_17455717, EPI_ISL_17455718, EPI_ISL_17455719, EPI_ISL_17455720, EPI_ISL_17455721, EPI_ISL_17455722, EPI_ISL_17455723, EPI_ISL_17455724			
see above	Colombian National Institute of Health	Colombian National Institute of Health	Laiton-Donato,K., Usme-Ciro,J., Rico,A. and Paez,A.
EPI_ISL_17455725	Commonwealth Scientific and Industrial Research Organisation	Commonwealth Scientific and Industrial Research Organisation	Vedururu,R., Neave,M., Duchemin,J.-B., Tachedjian,M., Gorry,P. and Paradkar,P.N.
EPI_ISL_17455726	D.I. Ivanovsky Institute of Virology, Laboratory of Biotechnology	D.I. Ivanovsky Institute of Virology, Laboratory of Biotechnology	Shchelkanov,M.Y., Lvov,D.K., Alkhovsky,S.V. and Shchetinin,A.M.
EPI_ISL_17455727, EPI_ISL_17455728	Defence Research & Development Establishment	Defence Research & Development Establishment	Dash,P.K., Parida,M.M., Santhosh,S.R., Verma,S.K., Tripathi,N.K., Ambuj,S., Saxena,P., Gupta,N., Chaudhary,M., Babu,J.P., Lakshmi,V., Mamidi,N., Subhalaxmi,M.V., Lakshmana Rao,P.V. and Sekhar,K.
EPI_ISL_17455729	Defence Research & Development Establishment	Defence Research & Development Establishment	Dash,P.K., Parida,M.M., Santhosh,S.R., Tripathi,N.K., Srivastava,A., Saxena,P., Gupta,N., Babu,J.P., Verma,S.K., Rao,P.V.L. and Sekhar,K.
EPI_ISL_17455730	Defence Research & Development Establishment	Defence Research & Development Establishment	Santhosh,S.R., Dash,P.K., Parida,M., Khan,M. and Rao,P.V.
EPI_ISL_17455731, EPI_ISL_17455732	Defence Research & Development Establishment	Defence Research & Development Establishment	Dash,P.K., Parida,M.M., Santhosh,S.R., Verma,S.K., Tripathi,N.K., Srivastava,A., Saxena,P., Gupta,N., Babu,J.P., Rao,P.V.L. and Sekhar,K.
EPI_ISL_17455733	Defence Research & Development Establishment	Defence Research & Development Establishment	Dash,P.K., Parida,M.M., Santhosh,S.R., Verma,S.K., Tripathi,N.K., Ambuj,S., Saxena,P., Gupta,N., Chaudhary,M., Babu,J.P., Lakshmi,V., Mamidi,N., Subhalaxmi,M.V., Lakshmana Rao,P.V. and Sekhar,K.
EPI_ISL_17455734, EPI_ISL_17455735	Defence Research & Development Establishment	Defence Research & Development Establishment	Dash,P.K., Parida,M.M., Santhosh,S.R., Verma,S.K., Tripathi,N.K., Srivastava,A., Saxena,P., Gupta,N., Babu,J.P., Rao,P.V.L. and Sekhar,K.
EPI_ISL_17455736, EPI_ISL_17455737	Defence Research & Development Establishment	Defence Research & Development Establishment	Soni,M., Singh,A.K., Sharma,S., Agarwal,A., Gopalan,N., Rao,P.V., Parida,M. and Dash,P.K.
EPI_ISL_17455738	Defence Research & Development Establishment	Defence Research & Development Establishment	Dash,P.K., Parida,M.M., Santhosh,S.R., Verma,S.K., Tripathi,N.K., Srivastava,A., Saxena,P., Gupta,N., Babu,J.P., Rao,P.V.L. and Sekhar,K.
EPI_ISL_17455739	Defence Research & Development Establishment	Defence Research & Development Establishment	Dash,P.K., Parida,M.M., Santhosh,S.R., Verma,S.K., Tripathi,N.K., Ambuj,S., Saxena,P., Gupta,N., Chaudhary,M., Babu,J.P., Lakshmi,V., Mamidi,N., Subhalaxmi,M.V., Lakshmana Rao,P.V. and Sekhar,K.
EPI_ISL_17455740	Defence Research & Development Establishment	Defence Research & Development Establishment	Singh,A.K., Soni,M., Agarwal,A., Dash,P.K., Parida,M. and Gopalan,N.
EPI_ISL_17455741, EPI_ISL_17455742	Defence Research & Development Establishment	Defence Research & Development Establishment	Dash,P.K., Parida,M.M., Santhosh,S.R., Tripathi,N.K., Srivastava,A., Saxena,P., Gupta,N., Babu,J.P., Verma,S.K., Rao,P.V.L. and Sekhar,K.
EPI_ISL_17455743	Defence Research & Development Establishment	Defence Research & Development Establishment	Dash,P.K., Parida,M.M., Santhosh,S.R., Verma,S.K., Tripathi,N.K., Srivastava,A., Saxena,P., Gupta,N., Babu,J.P., Rao,P.V.L. and Sekhar,K.
EPI_ISL_17455744	Defence Research & Development Establishment	Defence Research & Development Establishment	Dash,P.K., Parida,M.M., Santhosh,S.R., Tripathi,N.K., Srivastava,A., Saxena,P., Gupta,N., Babu,J.P., Verma,S.K., Rao,P.V.L. and Sekhar,K.
EPI_ISL_17455745	Defence Research & Development Establishment	Defence Research & Development Establishment	Dash,P.K., Soni,M., Sharma,S., Singh,A.K., Agarwal,A., Gopalan,N., Parida,M.M. and Rao,P.V.L.
EPI_ISL_17455746	Defence Research & Development Establishment	Defence Research & Development Establishment	Santhosh,S.R., Dash,P.K., Parida,M.M., Khan,M., Tiwari,M. and Lakshmana Rao,P.V.
EPI_ISL_17455747	Defence Research & Development Establishment	Defence Research & Development Establishment	Dash,P.K., Soni,M., Sharma,S., Singh,A.K., Agarwal,A., Gopalan,N., Parida,M.M. and Rao,P.V.L.
EPI_ISL_17455748, EPI_ISL_17455749	Defence Research & Development Establishment	Defence Research & Development Establishment	Soni,M., Singh,A.K., Sharma,S., Agarwal,A., Gopalan,N., Rao,P.V., Parida,M. and Dash,P.K.
EPI_ISL_17455750, EPI_ISL_17455751	Defence Research & Development Establishment	Defence Research & Development Establishment	Santhosh,S.R., Dash,P.K., Parida,M., Khan,M. and Rao,P.V.
EPI_ISL_17455752	Defence Research & Development Establishment	Defence Research & Development Establishment	Soni,M., Singh,A.K., Sharma,S., Agarwal,A., Gopalan,N., Rao,P.V., Parida,M. and Dash,P.K.
EPI_ISL_17455753, EPI_ISL_17455754	Defence Research & Development Establishment	Defence Research & Development Establishment	Santhosh,S.R., Dash,P.K., Parida,M., Khan,M. and Rao,P.V.
EPI_ISL_17455755	Defence Research & Development Establishment	Defence Research & Development Establishment	Dash,P.K., Parida,M.M., Santhosh,S.R., Verma,S.K., Tripathi,N.K., Srivastava,A., Saxena,P., Gupta,N., Babu,J.P., Rao,P.V.L. and Sekhar,K.
EPI_ISL_17455756	Defence Research & Development Establishment	Defence Research & Development Establishment	Santhosh,S.R., Dash,P.K., Parida,M.M., Khan,M., Tiwari,M. and Lakshmana Rao,P.V.
EPI_ISL_17455757, EPI_ISL_17455758	Defence Research & Development Establishment	Defence Research & Development Establishment	Dash,P.K., Parida,M.M., Santhosh,S.R., Tripathi,N.K., Srivastava,A., Saxena,P., Gupta,N., Babu,J.P., Verma,S.K., Rao,P.V.L. and Sekhar,K.
EPI_ISL_17455759	Defence Research & Development Establishment	Defence Research & Development Establishment	Soni,M., Singh,A.K., Sharma,S., Agarwal,A., Gopalan,N., Rao,P.V., Parida,M. and Dash,P.K.
EPI_ISL_17455760, EPI_ISL_17455761, EPI_ISL_17455762	Defence Research & Development Establishment	Defence Research & Development Establishment	Dash,P.K., Parida,M.M., Santhosh,S.R., Tripathi,N.K., Srivastava,A., Saxena,P., Gupta,N., Babu,J.P., Verma,S.K., Rao,P.V.L. and Sekhar,K.
EPI_ISL_17455763	Defence Research & Development	Defence Research & Development	Dash,P.K., Parida,M.M., Santhosh,S.R., Verma,S.K., Tripathi,N.K., Ambuj,S., Saxena,P., Gupta,N., Chaudhary,M., Babu,J.P., Lakshmi,V., Mamidi,N., Subhalaxmi,M.V., Lakshmana Rao,P.V. and Sekhar,K.

	Establishment	Establishment	
EPI_ISL_17455764	Defence Research & Development Establishment	Defence Research & Development Establishment	Singh,A.K., Soni,M., Agarwal,A., Dash,P.K., Parida,M. and Gopalan,N.
EPI_ISL_17455765, EPI_ISL_17455766, EPI_ISL_17455767	Defence Research & Development Establishment	Defence Research & Development Establishment	Santhosh,S.R., Dash,P.K., Parida,M., Khan,M. and Rao,P.V.
EPI_ISL_17455768	Defence Research & Development Establishment	Defence Research & Development Establishment	Dash,P.K., Parida,M.M., Santhosh,S.R., Verma,S.K., Tripathi,N.K., Ambuj,S., Saxena,P., Gupta,N., Chaudhary,M., Babu,J.P., Lakshmi,V., Mamidi,N., Subhalaxmi,M.V., Lakshmana Rao,P.V. and Sekhar,K.
EPI_ISL_17455769	Defence Research & Development Establishment	Defence Research & Development Establishment	Dash,P.K., Parida,M.M., Santhosh,S.R., Tripathi,N.K., Srivastava,A., Saxena,P., Gupta,N., Babu,J.P., Verma,S.K., Rao,P.V.L. and Sekhar,K.
EPI_ISL_17455770, EPI_ISL_17455771	Defence Research & Development Establishment	Defence Research & Development Establishment	Dash,P.K., Parida,M.M., Santhosh,S.R., Verma,S.K., Tripathi,N.K., Srivastava,A., Saxena,P., Gupta,N., Babu,J.P., Rao,P.V.L. and Sekhar,K.
EPI_ISL_17455772, EPI_ISL_17455773, EPI_ISL_17455774, EPI_ISL_17455775, EPI_ISL_17455776, EPI_ISL_17455777, EPI_ISL_17455778, EPI_ISL_17455779, EPI_ISL_17455780, EPI_ISL_17455781, EPI_ISL_17455782, EPI_ISL_17455783, EPI_ISL_17455784, EPI_ISL_17455785, EPI_ISL_17455786, EPI_ISL_17455787, EPI_ISL_17455788			
see above	Department of Biochemistry and Medical Biotechnology, Calcutta School of Tropical Medicine	Department of Biochemistry and Medical Biotechnology, Calcutta School of Tropical Medicine	Dutta,S.K. and Tripathi,A.
EPI_ISL_17455789	Department of Biotechnology, Faculty of Science, Jamia Hamdard	Department of Biotechnology, Faculty of Science, Jamia Hamdard	Khan,W.H. and Ray,P.
EPI_ISL_17455790	Department of Biotechnology, Faculty of Science, Jamia Hamdard	Department of Biotechnology, Faculty of Science, Jamia Hamdard	Khan,M.N., Ray,P. and Khan,W.H.
EPI_ISL_17455791, EPI_ISL_17455792, EPI_ISL_17455793	Department of Biotechnology, Faculty of Science, Jamia Hamdard	Department of Biotechnology, Faculty of Science, Jamia Hamdard	Khan,N. and Ray,P.
EPI_ISL_17455794	Department of Infectious Disease, Istituto Superiore Di Sanita	Department of Infectious Disease, Istituto Superiore Di Sanita	Lindh,E., Argentini,C., Remoli,M.E., Fortuna,C., Faggioni,G., Benedetti,E., Amendola,A., Marsili,G., Lista,F., Rezza,G. and Venturi,G.
EPI_ISL_17455795	Department of Infectious Disease, Istituto Superiore Di Sanita	Department of Infectious Disease, Istituto Superiore Di Sanita	Rezza,G., El-Sawaf,G., Faggioni,G., Vescio,F., Al Ameri,R., De Santis,R., Helaly,G., Pomponi,A., Metwally,D., Fantini,M., Qadi,H., Ciccozzi,M. and Lista,F.
EPI_ISL_17455796	Department of Infectious Disease, Istituto Superiore Di Sanita	Department of Infectious Disease, Istituto Superiore Di Sanita	Lindh,E., Argentini,C., Remoli,M.E., Fortuna,C., Faggioni,G., Benedetti,E., Amendola,A., Marsili,G., Lista,F., Rezza,G. and Venturi,G.
EPI_ISL_17455797	Department of Infectious Disease, Istituto Superiore Di Sanita	Department of Infectious Disease, Istituto Superiore Di Sanita	Rezza,G., El-Sawaf,G., Faggioni,G., Vescio,F., Al Ameri,R., De Santis,R., Helaly,G., Pomponi,A., Metwally,D., Fantini,M., Qadi,H., Ciccozzi,M. and Lista,F.
EPI_ISL_17455798, EPI_ISL_17455799	Department of Infectious Disease, Istituto Superiore Di Sanita	Department of Infectious Disease, Istituto Superiore Di Sanita	Lindh,E., Argentini,C., Remoli,M.E., Fortuna,C., Faggioni,G., Benedetti,E., Amendola,A., Marsili,G., Lista,F., Rezza,G. and Venturi,G.
EPI_ISL_17455800	Department of Infectious Disease, Istituto Superiore Di Sanita	Department of Infectious Disease, Istituto Superiore Di Sanita	Magurano,F., Bartoloni,A., Zammarchi,L., Baggieri,M., Fortuna,C. and Nicoletti,L.
EPI_ISL_17455801, EPI_ISL_17455802	Department of Infectious Disease, Istituto Superiore Di Sanita	Department of Infectious Disease, Istituto Superiore Di Sanita	Rezza,G., El-Sawaf,G., Faggioni,G., Vescio,F., Al Ameri,R., De Santis,R., Helaly,G., Pomponi,A., Metwally,D., Fantini,M., Qadi,H., Ciccozzi,M. and Lista,F.
EPI_ISL_17455803, EPI_ISL_17455804	Department of Infectious Disease, Istituto Superiore Di Sanita	Department of Infectious Disease, Istituto Superiore Di Sanita	Lindh,E., Argentini,C., Remoli,M.E., Fortuna,C., Faggioni,G., Benedetti,E., Amendola,A., Marsili,G., Lista,F., Rezza,G. and Venturi,G.
EPI_ISL_17455805	Department of Infectious Disease, Istituto Superiore Di Sanita	Department of Infectious Disease, Istituto Superiore Di Sanita	Rezza,G., El-Sawaf,G., Faggioni,G., Vescio,F., Al Ameri,R., De Santis,R., Helaly,G., Pomponi,A., Metwally,D., Fantini,M., Qadi,H., Ciccozzi,M. and Lista,F.
EPI_ISL_17455806	Department of Infectious Disease, Istituto Superiore Di Sanita	Department of Infectious Disease, Istituto Superiore Di Sanita	Lindh,E., Argentini,C., Remoli,M.E., Fortuna,C., Faggioni,G., Benedetti,E., Amendola,A., Marsili,G., Lista,F., Rezza,G. and Venturi,G.
EPI_ISL_17455807	Department of Infectious Disease, Istituto Superiore Di Sanita	Department of Infectious Disease, Istituto Superiore Di Sanita	Rezza,G., El-Sawaf,G., Faggioni,G., Vescio,F., Al Ameri,R., De Santis,R., Helaly,G., Pomponi,A., Metwally,D., Fantini,M., Qadi,H., Ciccozzi,M. and Lista,F.
EPI_ISL_17455808, EPI_ISL_17455809	Department of Infectious Disease, Istituto Superiore Di Sanita	Department of Infectious Disease, Istituto Superiore Di Sanita	Lindh,E., Argentini,C., Remoli,M.E., Fortuna,C., Faggioni,G., Benedetti,E., Amendola,A., Marsili,G., Lista,F., Rezza,G. and Venturi,G.
EPI_ISL_17455810, EPI_ISL_17455811	Department of Infectious Disease, Istituto Superiore Di Sanita	Department of Infectious Disease, Istituto Superiore Di Sanita	Rezza,G., El-Sawaf,G., Faggioni,G., Vescio,F., Al Ameri,R., De Santis,R., Helaly,G., Pomponi,A., Metwally,D., Fantini,M., Qadi,H., Ciccozzi,M. and Lista,F.
EPI_ISL_17455812	Department of Infectious Diseases, Istituto Superiore di Sanita	Department of Infectious Diseases, Istituto Superiore di Sanita	Lindh,E., Argentini,C., Remoli,M.E., Fortuna,C., Faggioni,G., Benedetti,E., Amendola,A., Marsili,G., Lista,F., Rezza,G. and Venturi,G.
EPI_ISL_17455813, EPI_ISL_17455814, EPI_ISL_17455815, EPI_ISL_17455816, EPI_ISL_17455817, EPI_ISL_17455818, EPI_ISL_17455819, EPI_ISL_17455820, EPI_ISL_17455821, EPI_ISL_17455822, EPI_ISL_17455823, EPI_ISL_17455824, EPI_ISL_17455825, EPI_ISL_17455826, EPI_ISL_17455827, EPI_ISL_17455828, EPI_ISL_17455829, EPI_ISL_17455830, EPI_ISL_17455831, EPI_ISL_17455832, EPI_ISL_17455833, EPI_ISL_17455834, EPI_ISL_17455835, EPI_ISL_17455836, EPI_ISL_17455837, EPI_ISL_17455838			
see above	Department of Infectious Diseases, Istituto Superiore di Sanita	Department of Infectious Diseases, Istituto Superiore di Sanita	Remoli,M.
EPI_ISL_17455839, EPI_ISL_17455840	Department of Infectious Diseases, Istituto Superiore di Sanita	Department of Infectious Diseases, Istituto Superiore di Sanita	Fortuna,C., Remoli,M.E., Rizzo,C., Benedetti,E., Fiorentini,C., Bella,A., Argentini,C., Farchi,F., Castilletti,C., Capobianchi,M.R., Zammarchi,L., Bartoloni,A., Zanchetta,N., Gismondo,M.R., Nelli,L.C., Vitale,G., Baldelli,F., D'Agaro,P., Sodano,G., Rezza,G., Venturi,G., Caratelli,A., Bizzotti,V., Casale,D., Lepore,D., Cecchetti,V., Caporali,M.G., Bordi,L., Carletti,F., Colavita,F., Lalle,E., Quartu,S., Malincarne,L., Caracciolo,I., Tiberio,C. and Falco,E.
EPI_ISL_17455841, EPI_ISL_17455842, EPI_ISL_17455843, EPI_ISL_17455844	Department of Infectious Diseases, Istituto Superiore di Sanita	Department of Infectious Diseases, Istituto Superiore di Sanita	Remoli,M.
EPI_ISL_17455845, EPI_ISL_17455846	Department of Inspection, Centers for Disease Control and Prevention of Lishui	Department of Inspection, Centers for Disease Control and Prevention of Lishui	Wang,X.G. and Yang,R.J.
EPI_ISL_17455847	Department of Medical Microbiology, Faculty of Medicine, University of Malaya	Department of Medical Microbiology, Faculty of Medicine, University of Malaya	Chee,Y.S., Ahamad Fouzi,N., Chong,Y.M., Sam,I.C., Chan,Y.F., Chua,C.L. and Wang,Q.Y.
EPI_ISL_17455848, EPI_ISL_17455849, EPI_ISL_17455850	Department of Medical Microbiology, Faculty of Medicine, University of Malaya	Department of Medical Microbiology, Faculty of Medicine, University of Malaya	Sam,I.C., Chan,Y.F., Loong,S.K., Chiam,C.W., Chua,C.L. and Wong,H.V.
EPI_ISL_17455851	Department of Medical Microbiology, Faculty of Medicine, University of Malaya	Department of Medical Microbiology, Faculty of Medicine, University of Malaya	Chee,Y.S., Ahamad Fouzi,N., Chong,Y.M., Sam,I.C., Chan,Y.F., Chua,C.L. and Wang,Q.Y.
EPI_ISL_17455852, EPI_ISL_17455853	Department of Medical Microbiology, Faculty of Medicine, University of Malaya	Department of Medical Microbiology, Faculty of Medicine, University of Malaya	Sam,I.C., Chan,Y.F., Loong,S.K., Chiam,C.W., Chua,C.L. and Wong,H.V.
EPI_ISL_17455854, EPI_ISL_17455855, EPI_ISL_17455856, EPI_ISL_17455857, EPI_ISL_17455858, EPI_ISL_17455859, EPI_ISL_17455860, EPI_ISL_17455861, EPI_ISL_17455862, EPI_ISL_17455863, EPI_ISL_17455864, EPI_ISL_17455865, EPI_ISL_17455866, EPI_ISL_17455867, EPI_ISL_17455868			
see above	Department of Medical Research	Department of Medical Research	Kyaw,A.K., Tun,M.M.N., Nabeshima,T., Soe,A.M., Thida,T., Aung,T.H., Htwe,T.T., Myaing,S.S., Mar,T.T., Aung,T., Win,K.M.M., Mar Myint,K., Lwin,E.P., Thu,H.M., Buerano,C.C., Thant,K.Z. and Morita,K.
EPI_ISL_17455869, EPI_ISL_17455870, EPI_ISL_17455871	Department of Microbiology, School of Basic Medical Sciences, Capital Medical University	Department of Microbiology, School of Basic Medical Sciences, Capital Medical University	Liu,L., Gao,N., Li,M., Shen,J., Sheng,Z., Fan,D., Zhou,H., Yin,X., Mao,J., Jiang,J., Wang,P. and An,J.
EPI_ISL_17455872	Department of Microbiology, Sri Ramachandra Medical College & Research Institute, Sri Ramachandra University	Department of Microbiology, Sri Ramachandra Medical College & Research Institute, Sri Ramachandra University	Srikanth,P., Gopalsamy,S., Seema Nayar,A., Ramya,B., Gracyfathima,S., Matthew,T., Sheriff,A.K., Gunasekaran,P., Karthikbabu,M. and Muthumani,K.
EPI_ISL_17455873	Department of Microbiology, Sri Ramachandra Medical College & Research Institute, Sri Ramachandra University	Department of Microbiology, Sri Ramachandra Medical College & Research Institute, Sri Ramachandra University	Seema,N.A., Gopalsamy,S., Ramya,B., Seshapoornima,B., Damodharan,J., Mathew,T., Selvaraj,G., Sheriff,A., Muthumani,K. and Srikanth,P.
EPI_ISL_17455874, EPI_ISL_17455875, EPI_ISL_17455876, EPI_ISL_17455877, EPI_ISL_17455878, EPI_ISL_17455879, EPI_ISL_17455880	Department of Microbiology, Sri Ramachandra Medical College & Research Institute, Sri Ramachandra University	Department of Microbiology, Sri Ramachandra Medical College & Research Institute, Sri Ramachandra University	Srikanth,P., Gopalsamy,S., Seema Nayar,A., Ramya,B., Gracyfathima,S., Matthew,T., Sheriff,A.K., Gunasekaran,P., Karthikbabu,M. and Muthumani,K.
EPI_ISL_17455881, EPI_ISL_17455882	Department of Microbiology, Sri Ramachandra Medical College & Research Institute, Sri Ramachandra University	Department of Microbiology, Sri Ramachandra Medical College & Research Institute, Sri Ramachandra University	Seema,N.A., Gopalsamy,S., Ramya,B., Seshapoornima,B., Damodharan,J., Mathew,T., Selvaraj,G., Sheriff,A., Muthumani,K. and Srikanth,P.
EPI_ISL_17455883, EPI_ISL_17455884, EPI_ISL_17455885	Department of Microbiology, Sri Ramachandra Medical College & Research Institute, Sri Ramachandra University	Department of Microbiology, Sri Ramachandra Medical College & Research Institute, Sri Ramachandra University	Srikanth,P., Gopalsamy,S., Seema Nayar,A., Ramya,B., Gracyfathima,S., Matthew,T., Sheriff,A.K., Gunasekaran,P., Karthikbabu,M. and Muthumani,K.
EPI_ISL_17455886	Department of Microbiology, Sri Ramachandra Medical College & Research Institute, Sri Ramachandra University	Department of Microbiology, Sri Ramachandra Medical College & Research Institute, Sri Ramachandra University	Seema,N.A., Gopalsamy,S., Ramya,B., Seshapoornima,B., Damodharan,J., Mathew,T., Selvaraj,G., Sheriff,A., Muthumani,K. and Srikanth,P.
EPI_ISL_17455887	Department of Microbiology, Sri Ramachandra	Department of Microbiology, Sri Ramachandra	Gopalsamy,S., Nayar,S.A., Ramya,B., Seshapoornima,B., Damodharan,J., Mathew,T., Selvarai,G., Sheriff,A., Palani,G., Muthumani,K. and Srikanth,P.

	Medical College & Research Institute, Sri Ramachandra University	Medical College & Research Institute, Sri Ramachandra University	
EPI_ISL_17455888	Department of Microbiology, Sri Ramachandra Medical College & Research Institute, Sri Ramachandra University	Department of Microbiology, Sri Ramachandra Medical College & Research Institute, Sri Ramachandra University	Srikanth,P., Gopalsamy,S., Seema Nayar,A., Ramya,B., Gracyfathima,S., Matthew,T., Sheriff,A.K., Gunasekaran,P., Karthikbabu,M. and Muthumani,K.
EPI_ISL_17455889	Department of Microbiology, Sri Ramachandra Medical College & Research Institute, Sri Ramachandra University	Department of Microbiology, Sri Ramachandra Medical College & Research Institute, Sri Ramachandra University	Gopalsamy,S., Nayar,S.A., Ramya,B., Seshapoornima,B., Damodharan,J., Mathew,T., Selvarai,G., Sheriff,A., Palani,G., Muthumani,K. and Srikanth,P.
EPI_ISL_17455890	Department of Microbiology, Sri Ramachandra Medical College & Research Institute, Sri Ramachandra University	Department of Microbiology, Sri Ramachandra Medical College & Research Institute, Sri Ramachandra University	Srikanth,P., Gopalsamy,S., Seema Nayar,A., Ramya,B., Gracyfathima,S., Matthew,T., Sheriff,A.K., Gunasekaran,P., Karthikbabu,M. and Muthumani,K.
EPI_ISL_17455891, EPI_ISL_17455892	Department of Microbiology, Sri Ramachandra Medical College & Research Institute, Sri Ramachandra University	Department of Microbiology, Sri Ramachandra Medical College & Research Institute, Sri Ramachandra University	Gopalsamy,S., Nayar,S.A., Ramya,B., Seshapoornima,B., Damodharan,J., Mathew,T., Selvarai,G., Sheriff,A., Palani,G., Muthumani,K. and Srikanth,P.
EPI_ISL_17455893	Department of Microbiology, Sri Ramachandra Medical College & Research Institute, Sri Ramachandra University	Department of Microbiology, Sri Ramachandra Medical College & Research Institute, Sri Ramachandra University	Srikanth,P., Gopalsamy,S., Seema Nayar,A., Ramya,B., Gracyfathima,S., Matthew,T., Sheriff,A.K., Gunasekaran,P., Karthikbabu,M. and Muthumani,K.
EPI_ISL_17455894	Department of Microbiology, Sri Ramachandra Medical College & Research Institute, Sri Ramachandra University	Department of Microbiology, Sri Ramachandra Medical College & Research Institute, Sri Ramachandra University	Gopalsamy,S., Nayar,S.A., Ramya,B., Seshapoornima,B., Damodharan,J., Mathew,T., Selvarai,G., Sheriff,A., Palani,G., Muthumani,K. and Srikanth,P.
EPI_ISL_17455895	Department of Microbiology, Sri Ramachandra Medical College & Research Institute, Sri Ramachandra University	Department of Microbiology, Sri Ramachandra Medical College & Research Institute, Sri Ramachandra University	Seema,N.A., Gopalsamy,S., Ramya,B., Seshapoornima,B., Damodharan,J., Mathew,T., Selvaraj,G., Sheriff,A., Muthumani,K. and Srikanth,P.
EPI_ISL_17455896, EPI_ISL_17455897, EPI_ISL_17455898, EPI_ISL_17455899, EPI_ISL_17455900, EPI_ISL_17455901, EPI_ISL_17455902	Department of Neurovirology, National Institute of Mental Health and Neurosciences	Department of Neurovirology, National Institute of Mental Health and Neurosciences	Vijayalakshmi,R., Desai,A. and Ravi,V.
EPI_ISL_17455903	Department of Neurovirology, National Institute of Mental Health and Neurosciences	Department of Neurovirology, National Institute of Mental Health and Neurosciences	Rasheed,R., Prasad,P., Pattabiraman,C., Desai,A. and Ravi,V.
EPI_ISL_17455904, EPI_ISL_17455905, EPI_ISL_17455906, EPI_ISL_17455907	Department of Neurovirology, National Institute of Mental Health and Neurosciences	Department of Neurovirology, National Institute of Mental Health and Neurosciences	Vijayalakshmi,R., Desai,A. and Ravi,V.
EPI_ISL_17455908, EPI_ISL_17455909, EPI_ISL_17455910, EPI_ISL_17455911, EPI_ISL_17455912, EPI_ISL_17455913, EPI_ISL_17455914, EPI_ISL_17455915, EPI_ISL_17455916, EPI_ISL_17455917, EPI_ISL_17455918, EPI_ISL_17455919, EPI_ISL_17455920, EPI_ISL_17455921, EPI_ISL_17455922, EPI_ISL_17455923			
see above	Department of Parasitology, Faculty of Medicine, Chulalongkorn University	Department of Parasitology, Faculty of Medicine, Chulalongkorn University	Intayot,P., Phumee,A., Boonserm,R., Sor-Suwan,S., Buathong,R., Wacharapluesadee,S., Brownell,N., Poovorawan,Y. and Siriyasatien,P.
EPI_ISL_17455924, EPI_ISL_17455925, EPI_ISL_17455926	Department of Parasitology, Faculty of Medicine, Chulalongkorn University	Department of Parasitology, Faculty of Medicine, Chulalongkorn University	Intayot,P., Phumee,A. and Siriyasatien,P.
EPI_ISL_17455927, EPI_ISL_17455928, EPI_ISL_17455929, EPI_ISL_17455930	Department of Parasitology, Faculty of Medicine, Chulalongkorn University	Department of Parasitology, Faculty of Medicine, Chulalongkorn University	Intayot,P., Phumee,A., Boonserm,R., Sor-Suwan,S., Buathong,R., Wacharapluesadee,S., Brownell,N., Poovorawan,Y. and Siriyasatien,P.
EPI_ISL_17455931	Department of Parasitology, Faculty of Medicine, Chulalongkorn University	Department of Parasitology, Faculty of Medicine, Chulalongkorn University	Intayot,P., Phumee,A. and Siriyasatien,P.
EPI_ISL_17455932, EPI_ISL_17455933, EPI_ISL_17455934, EPI_ISL_17455935, EPI_ISL_17455936, EPI_ISL_17455937, EPI_ISL_17455938, EPI_ISL_17455939, EPI_ISL_17455940, EPI_ISL_17455941, EPI_ISL_17455942, EPI_ISL_17455943, EPI_ISL_17455944, EPI_ISL_17455945, EPI_ISL_17455946, EPI_ISL_17455947, EPI_ISL_17455948, EPI_ISL_17455949, EPI_ISL_17455950, EPI_ISL_17455951, EPI_ISL_17455952, EPI_ISL_17455953, EPI_ISL_17455954, EPI_ISL_17455955, EPI_ISL_17455956, EPI_ISL_17455957, EPI_ISL_17455958, EPI_ISL_17455959, EPI_ISL_17455960, EPI_ISL_17455961, EPI_ISL_17455962, EPI_ISL_17455963, EPI_ISL_17455964, EPI_ISL_17455965, EPI_ISL_17455966, EPI_ISL_17455967, EPI_ISL_17455968, EPI_ISL_17455969, EPI_ISL_17455970, EPI_ISL_17455971, EPI_ISL_17455972, EPI_ISL_17455973, EPI_ISL_17455974, EPI_ISL_17455975, EPI_ISL_17455976, EPI_ISL_17455977, EPI_ISL_17455978			
see above	Department of Parasitology, Faculty of Medicine, Chulalongkorn University	Department of Parasitology, Faculty of Medicine, Chulalongkorn University	Intayot,P., Phumee,A., Boonserm,R., Sor-Suwan,S., Buathong,R., Wacharapluesadee,S., Brownell,N., Poovorawan,Y. and Siriyasatien,P.
EPI_ISL_17455979, EPI_ISL_17455980, EPI_ISL_17455981, EPI_ISL_17455982, EPI_ISL_17455983, EPI_ISL_17455984, EPI_ISL_17455985	Department of Pathology, University of Texas Medical Branch	Department of Pathology, University of Texas Medical Branch	Volk,S.M., Chen,R., Tsetsarkin,K.A., Adams,A.P., Garcia,T.I., Sall,A.A., Nasar,F., Schuh,A.J., Holmes,E.C., Higgs,S., Maharaj,P.D., Brault,A.C. and Weaver,S.C.
EPI_ISL_17455986, EPI_ISL_17455987	Department of Pathology, University of Texas Medical Branch	Department of Pathology, University of Texas Medical Branch	Chen,R., Puri,V., Fedorova,N., Lin,D., Hari,K.L., Jain,R., Rodas,J.D., Das,S.R., Shabman,R.S. and Weaver,S.C.
EPI_ISL_17455988	Department of Pathology, University of Texas Medical Branch	Department of Pathology, University of Texas Medical Branch	Volk,S.M., Chen,R., Tsetsarkin,K.A., Adams,A.P., Garcia,T.I., Sall,A.A., Nasar,F., Schuh,A.J., Holmes,E.C., Higgs,S., Maharaj,P.D., Brault,A.C. and Weaver,S.C.
EPI_ISL_17455989	Department of Pathology, University of Texas Medical Branch	Department of Pathology, University of Texas Medical Branch	Chen,R., Puri,V., Fedorova,N., Lin,D., Hari,K.L., Jain,R., Rodas,J.D., Das,S.R., Shabman,R.S. and Weaver,S.C.
EPI_ISL_17455990, EPI_ISL_17455991	Department of Pathology, University of Texas Medical Branch	Department of Pathology, University of Texas Medical Branch	Volk,S.M., Chen,R., Tsetsarkin,K.A., Adams,A.P., Garcia,T.I., Sall,A.A., Nasar,F., Schuh,A.J., Holmes,E.C., Higgs,S., Maharaj,P.D., Brault,A.C. and Weaver,S.C.
EPI_ISL_17455992	Department of Pathology, University of Texas Medical Branch	Department of Pathology, University of Texas Medical Branch	Chen,R., Puri,V., Fedorova,N., Lin,D., Hari,K.L., Jain,R., Rodas,J.D., Das,S.R., Shabman,R.S. and Weaver,S.C.
EPI_ISL_17455993, EPI_ISL_17455994	Department of Pathology, University of Texas Medical Branch	Department of Pathology, University of Texas Medical Branch	Volk,S.M., Chen,R., Tsetsarkin,K.A., Adams,A.P., Garcia,T.I., Sall,A.A., Nasar,F., Schuh,A.J., Holmes,E.C., Higgs,S., Maharaj,P.D., Brault,A.C. and Weaver,S.C.
EPI_ISL_17455995	Department of Pathology, University of Texas Medical Branch	Department of Pathology, University of Texas Medical Branch	Chen,R., Puri,V., Fedorova,N., Lin,D., Hari,K.L., Jain,R., Rodas,J.D., Das,S.R., Shabman,R.S. and Weaver,S.C.
EPI_ISL_17455996, EPI_ISL_17455997, EPI_ISL_17455998, EPI_ISL_17455999, EPI_ISL_17456000, EPI_ISL_17456001, EPI_ISL_17456002, EPI_ISL_17456003, EPI_ISL_17456004, EPI_ISL_17456005, EPI_ISL_17456006, EPI_ISL_17456007, EPI_ISL_17456008, EPI_ISL_17456009, EPI_ISL_17456010, EPI_ISL_17456011, EPI_ISL_17456012, EPI_ISL_17456013, EPI_ISL_17456014, EPI_ISL_17456015, EPI_ISL_17456016, EPI_ISL_17456017, EPI_ISL_17456018, EPI_ISL_17456019, EPI_ISL_17456020, EPI_ISL_17456021, EPI_ISL_17456022			
see above	Department of Pathology, University of Texas Medical Branch	Department of Pathology, University of Texas Medical Branch	Volk,S.M., Chen,R., Tsetsarkin,K.A., Adams,A.P., Garcia,T.I., Sall,A.A., Nasar,F., Schuh,A.J., Holmes,E.C., Higgs,S., Maharaj,P.D., Brault,A.C. and Weaver,S.C.
EPI_ISL_17456023	Department of Pathology, University of Texas Medical Branch	Department of Pathology, University of Texas Medical Branch	Chen,R., Puri,V., Fedorova,N., Lin,D., Hari,K.L., Jain,R., Rodas,J.D., Das,S.R., Shabman,R.S. and Weaver,S.C.
EPI_ISL_17456024	Department of Pathology, University of Texas Medical Branch	Department of Pathology, University of Texas Medical Branch	Volk,S.M., Chen,R., Tsetsarkin,K.A., Adams,A.P., Garcia,T.I., Sall,A.A., Nasar,F., Schuh,A.J., Holmes,E.C., Higgs,S., Maharaj,P.D., Brault,A.C. and Weaver,S.C.
EPI_ISL_17456025, EPI_ISL_17456026, EPI_ISL_17456027, EPI_ISL_17456028, EPI_ISL_17456029, EPI_ISL_17456030, EPI_ISL_17456031, EPI_ISL_17456032, EPI_ISL_17456033, EPI_ISL_17456034, EPI_ISL_17456035, EPI_ISL_17456036, EPI_ISL_17456037, EPI_ISL_17456038, EPI_ISL_17456039, EPI_ISL_17456040, EPI_ISL_17456041, EPI_ISL_17456042, EPI_ISL_17456043, EPI_ISL_17456044, EPI_ISL_17456045, EPI_ISL_17456046, EPI_ISL_17456047, EPI_ISL_17456048, EPI_ISL_17456049, EPI_ISL_17456050			
see above	Department of Pre-Clinical Sciences, Faculty of Medical Sciences, University of the West Indies	Department of Pre-Clinical Sciences, Faculty of Medical Sciences, University of the West Indies	Sahadeo,N.S.D., Allicock,O.M., De Salazar,P.M., Auguste,A.J., Widen,S., Olowokure,B., Gutierrez,C., Valadere,A.M., Polson-Edwards,K., Weaver,S.C. and Carrington,C.V.F.
EPI_ISL_17456051, EPI_ISL_17456052	Department of Pre-Clinical Sciences, Faculty of Medical Sciences, University of the West Indies	Department of Pre-Clinical Sciences, Faculty of Medical Sciences, University of the West Indies	Sahadeo,N., Mohammed,H., Allicock,O.M., Auguste,A.J., Widen,S.G., Badal,K., Pulchan,K., Foster,J.E., Weaver,S.C. and Carrington,C.V.
EPI_ISL_17456053	Department of Pre-Clinical Sciences, Faculty of Medical Sciences, University of the West Indies	Department of Pre-Clinical Sciences, Faculty of Medical Sciences, University of the West Indies	Sahadeo,N.S.D., Allicock,O.M., De Salazar,P.M., Auguste,A.J., Widen,S., Olowokure,B., Gutierrez,C., Valadere,A.M., Polson-Edwards,K., Weaver,S.C. and Carrington,C.V.F.
EPI_ISL_17456054	Department of Pre-Clinical Sciences, Faculty of Medical Sciences, University of the West Indies	Department of Pre-Clinical Sciences, Faculty of Medical Sciences, University of the West Indies	Sahadeo,N., Mohammed,H., Allicock,O.M., Auguste,A.J., Widen,S.G., Badal,K., Pulchan,K., Foster,J.E., Weaver,S.C. and Carrington,C.V.
EPI_ISL_17456055, EPI_ISL_17456056, EPI_ISL_17456057	Department of Pre-Clinical Sciences, Faculty of Medical Sciences, University of the West Indies	Department of Pre-Clinical Sciences, Faculty of Medical Sciences, University of the West Indies	Sahadeo,N.S.D., Allicock,O.M., De Salazar,P.M., Auguste,A.J., Widen,S., Olowokure,B., Gutierrez,C., Valadere,A.M., Polson-Edwards,K., Weaver,S.C. and Carrington,C.V.F.
EPI_ISL_17456058	Department of Pre-Clinical Sciences, Faculty of Medical Sciences, University of the West Indies	Department of Pre-Clinical Sciences, Faculty of Medical Sciences, University of the West Indies	Sahadeo,N., Mohammed,H., Allicock,O.M., Auguste,A.J., Widen,S.G., Badal,K., Pulchan,K., Foster,J.E., Weaver,S.C. and Carrington,C.V.
EPI_ISL_17456059, EPI_ISL_17456060	Department of Pre-Clinical Sciences, Faculty of Medical Sciences, University of the West Indies	Department of Pre-Clinical Sciences, Faculty of Medical Sciences, University of the West Indies	Sahadeo,N.S.D., Allicock,O.M., De Salazar,P.M., Auguste,A.J., Widen,S., Olowokure,B., Gutierrez,C., Valadere,A.M., Polson-Edwards,K., Weaver,S.C. and Carrington,C.V.F.
EPI_ISL_17456061, EPI_ISL_17456062, EPI_ISL_17456063, EPI_ISL_17456064	Department of Pre-Clinical Sciences, Faculty of Medical Sciences, University of the West Indies	Department of Pre-Clinical Sciences, Faculty of Medical Sciences, University of the West Indies	Sahadeo,N., Mohammed,H., Allicock,O.M., Auguste,A.J., Widen,S.G., Badal,K., Pulchan,K., Foster,J.E., Weaver,S.C. and Carrington,C.V.
EPI_ISL_17456065	Department of Pre-Clinical Sciences, Faculty of Medical Sciences, University of the West Indies	Department of Pre-Clinical Sciences, Faculty of Medical Sciences, University of the West Indies	Sahadeo,N.S.D., Allicock,O.M., De Salazar,P.M., Auguste,A.J., Widen,S., Olowokure,B., Gutierrez,C., Valadere,A.M., Polson-Edwards,K., Weaver,S.C. and Carrington,C.V.F.
EPI_ISL_17456066, EPI_ISL_17456067, EPI_ISL_17456068, EPI_ISL_17456069, EPI_ISL_17456070, EPI_ISL_17456071, EPI_ISL_17456072, EPI_ISL_17456073, EPI_ISL_17456074, EPI_ISL_17456075, EPI_ISL_17456076, EPI_ISL_17456077, EPI_ISL_17456078, EPI_ISL_17456079			
see above	Department of Virology, Sri Venkateswara University	Department of Virology, Sri Venkateswara University	Naresh Kumar,C.V.M. and Sai Gopal,D.V.R.

EPI_ISL_17456080	Department of Virology, Sri Venkateswara University	Department of Virology, Sri Venkateswara University	Naresh Kumar,C.V.M., Mahathi,N. and Sai Gopal,D.V.R.
EPI_ISL_17456081	Department of Virology, Sri Venkateswara University	Department of Virology, Sri Venkateswara University	Sangamithra,P. and Sai Gopal,D.V.R.
EPI_ISL_17456082, EPI_ISL_17456083	Department of Virology, Sri Venkateswara University	Department of Virology, Sri Venkateswara University	Naresh Kumar,C.V.M. and Sai Gopal,D.V.R.
EPI_ISL_17456084	Department of Virology, Sri Venkateswara University	Department of Virology, Sri Venkateswara University	Naresh Kumar,C.V.M. and Saigopal,D.V.R.
EPI_ISL_17456085	Department of Virology, Sri Venkateswara University	Department of Virology, Sri Venkateswara University	Mahathi,N., Naresh Kumar,C.V.M. and Sai Gopal,D.V.R.
EPI_ISL_17456086	Department of Virology, Sri Venkateswara University	Department of Virology, Sri Venkateswara University	Sangamithra,P. and Sai Gopal,D.V.R.
EPI_ISL_17456087	Department of Virology, Sri Venkateswara University	Department of Virology, Sri Venkateswara University	M Naresh Kumar,C.V., Anthony Johnson,A.M. and R Sai Gopal,D.V.
EPI_ISL_17456088, EPI_ISL_17456089	Department of Virology, Sri Venkateswara University, S.V.U. College of Sciences	Department of Virology, Sri Venkateswara University, S.V.U. College of Sciences	Naresh Kumar,C.V.M. and Saigopal,D.V.R.
EPI_ISL_17456090, EPI_ISL_17456091, EPI_ISL_17456092, EPI_ISL_17456093	Department of Zoology, University of Oxford	Department of Zoology, University of Oxford	Naveca,F.G., Claro,I., Giovanetti,M., Jesus,J.G., Xavier,J., do Nascimento,V.A., Iani,F.C.M., de Souza,V.C., Silveira,P.P., Lourenco,J., Santillana,M., Kraemer,M.U.G., Quick,J., Hill,S.C., Theze,J., Wallau,G.L., Carvalho,R.D.O., Azevedo,V., Salles,F.C.S., Candido,D.S., Nunes,M.R.T., Lemos,P.S., Meneses,C.A.R., Maito,R.M., Cunha,C.R.S.B., Campos,D.P.S., Castilho,M.C., Siqueira,T.C.S., Terra,T.M., de Albuquerque,C.F.C., da Cruz,L.N., Abreu,A.L., Simoes,D.S.M.V., Aguiar,R.S., Sabino,E.C., Pybus,O.G., Loman,N., Okumoto,O., Alcantara,L.C.J. and Faria,N.R.
EPI_ISL_17456094	Department of Zoology, University of Oxford	Department of Zoology, University of Oxford	da Costa,A.C., Theze,J., Komninakis,S.C.V., Sanz-Duro,R.L., Felinto,M.R.L., Moura,L.C.C., Barroso,I.M.O., Santos,L.E.C., Nunes,M.A.L., Moura,A.A., Lourenco,J., Deng,X., Delwart,E.L., Guimaraes,M.R.A.S., Pybus,O.G., Sabino,E.C. and Faria,N.R.
EPI_ISL_17456095, EPI_ISL_17456096, EPI_ISL_17456097, EPI_ISL_17456098	Department of Zoology, University of Oxford	Department of Zoology, University of Oxford	Naveca,F.G., Claro,I., Giovanetti,M., Jesus,J.G., Xavier,J., do Nascimento,V.A., Iani,F.C.M., de Souza,V.C., Silveira,P.P., Lourenco,J., Santillana,M., Kraemer,M.U.G., Quick,J., Hill,S.C., Theze,J., Wallau,G.L., Carvalho,R.D.O., Azevedo,V., Salles,F.C.S., Candido,D.S., Nunes,M.R.T., Lemos,P.S., Meneses,C.A.R., Maito,R.M., Cunha,C.R.S.B., Campos,D.P.S., Castilho,M.C., Siqueira,T.C.S., Terra,T.M., de Albuquerque,C.F.C., da Cruz,L.N., Abreu,A.L., Simoes,D.S.M.V., Aguiar,R.S., Sabino,E.C., Pybus,O.G., Loman,N., Okumoto,O., Alcantara,L.C.J. and Faria,N.R.
EPI_ISL_17456099, EPI_ISL_17456100, EPI_ISL_17456101, EPI_ISL_17456102, EPI_ISL_17456103	Department of Zoology, University of Oxford	Department of Zoology, University of Oxford	da Costa,A.C., Theze,J., Komninakis,S.C.V., Sanz-Duro,R.L., Felinto,M.R.L., Moura,L.C.C., Barroso,I.M.O., Santos,L.E.C., Nunes,M.A.L., Moura,A.A., Lourenco,J., Deng,X., Delwart,E.L., Guimaraes,M.R.A.S., Pybus,O.G., Sabino,E.C. and Faria,N.R.
EPI_ISL_17456104	Department of Zoology, University of Oxford	Department of Zoology, University of Oxford	Naveca,F.G., Claro,I., Giovanetti,M., Jesus,J.G., Xavier,J., do Nascimento,V.A., Iani,F.C.M., de Souza,V.C., Silveira,P.P., Lourenco,J., Santillana,M., Kraemer,M.U.G., Quick,J., Hill,S.C., Theze,J., Wallau,G.L., Carvalho,R.D.O., Azevedo,V., Salles,F.C.S., Candido,D.S., Nunes,M.R.T., Lemos,P.S., Meneses,C.A.R., Maito,R.M., Cunha,C.R.S.B., Campos,D.P.S., Castilho,M.C., Siqueira,T.C.S., Terra,T.M., de Albuquerque,C.F.C., da Cruz,L.N., Abreu,A.L., Simoes,D.S.M.V., Aguiar,R.S., Sabino,E.C., Pybus,O.G., Loman,N., Okumoto,O., Alcantara,L.C.J. and Faria,N.R.
EPI_ISL_17456105, EPI_ISL_17456106	Department of Zoology, University of Oxford	Department of Zoology, University of Oxford	da Costa,A.C., Theze,J., Komninakis,S.C.V., Sanz-Duro,R.L., Felinto,M.R.L., Moura,L.C.C., Barroso,I.M.O., Santos,L.E.C., Nunes,M.A.L., Moura,A.A., Lourenco,J., Deng,X., Delwart,E.L., Guimaraes,M.R.A.S., Pybus,O.G., Sabino,E.C. and Faria,N.R.
EPI_ISL_17456107, EPI_ISL_17456108, EPI_ISL_17456109	Department of Zoology, University of Oxford	Department of Zoology, University of Oxford	Naveca,F.G., Claro,I., Giovanetti,M., Jesus,J.G., Xavier,J., do Nascimento,V.A., Iani,F.C.M., de Souza,V.C., Silveira,P.P., Lourenco,J., Santillana,M., Kraemer,M.U.G., Quick,J., Hill,S.C., Theze,J., Wallau,G.L., Carvalho,R.D.O., Azevedo,V., Salles,F.C.S., Candido,D.S., Nunes,M.R.T., Lemos,P.S., Meneses,C.A.R., Maito,R.M., Cunha,C.R.S.B., Campos,D.P.S., Castilho,M.C., Siqueira,T.C.S., Terra,T.M., de Albuquerque,C.F.C., da Cruz,L.N., Abreu,A.L., Simoes,D.S.M.V., Aguiar,R.S., Sabino,E.C., Pybus,O.G., Loman,N., Okumoto,O., Alcantara,L.C.J. and Faria,N.R.
EPI_ISL_17456110	Department of Zoology, University of Oxford	Department of Zoology, University of Oxford	da Costa,A.C., Theze,J., Komninakis,S.C.V., Sanz-Duro,R.L., Felinto,M.R.L., Moura,L.C.C., Barroso,I.M.O., Santos,L.E.C., Nunes,M.A.L., Moura,A.A., Lourenco,J., Deng,X., Delwart,E.L., Guimaraes,M.R.A.S., Pybus,O.G., Sabino,E.C. and Faria,N.R.
EPI_ISL_17456111	Department of Zoology, University of Oxford	Department of Zoology, University of Oxford	Naveca,F.G., Claro,I., Giovanetti,M., Jesus,J.G., Xavier,J., do Nascimento,V.A., Iani,F.C.M., de Souza,V.C., Silveira,P.P., Lourenco,J., Santillana,M., Kraemer,M.U.G., Quick,J., Hill,S.C., Theze,J., Wallau,G.L., Carvalho,R.D.O., Azevedo,V., Salles,F.C.S., Candido,D.S., Nunes,M.R.T., Lemos,P.S., Meneses,C.A.R., Maito,R.M., Cunha,C.R.S.B., Campos,D.P.S., Castilho,M.C., Siqueira,T.C.S., Terra,T.M., de Albuquerque,C.F.C., da Cruz,L.N., Abreu,A.L., Simoes,D.S.M.V., Aguiar,R.S., Sabino,E.C., Pybus,O.G., Loman,N., Okumoto,O., Alcantara,L.C.J. and Faria,N.R.
EPI_ISL_17456112, EPI_ISL_17456113, EPI_ISL_17456114	Department of Zoology, University of Oxford	Department of Zoology, University of Oxford	da Costa,A.C., Theze,J., Komninakis,S.C.V., Sanz-Duro,R.L., Felinto,M.R.L., Moura,L.C.C., Barroso,I.M.O., Santos,L.E.C., Nunes,M.A.L., Moura,A.A., Lourenco,J., Deng,X., Delwart,E.L., Guimaraes,M.R.A.S., Pybus,O.G., Sabino,E.C. and Faria,N.R.
EPI_ISL_17456115, EPI_ISL_17456116, EPI_ISL_17456117	Department of Zoology, University of Oxford	Department of Zoology, University of Oxford	Naveca,F.G., Claro,I., Giovanetti,M., Jesus,J.G., Xavier,J., do Nascimento,V.A., Iani,F.C.M., de Souza,V.C., Silveira,P.P., Lourenco,J., Santillana,M., Kraemer,M.U.G., Quick,J., Hill,S.C., Theze,J., Wallau,G.L., Carvalho,R.D.O., Azevedo,V., Salles,F.C.S., Candido,D.S., Nunes,M.R.T., Lemos,P.S., Meneses,C.A.R., Maito,R.M., Cunha,C.R.S.B., Campos,D.P.S., Castilho,M.C., Siqueira,T.C.S., Terra,T.M., de Albuquerque,C.F.C., da Cruz,L.N., Abreu,A.L., Simoes,D.S.M.V., Aguiar,R.S., Sabino,E.C., Pybus,O.G., Loman,N., Okumoto,O., Alcantara,L.C.J. and Faria,N.R.
EPI_ISL_17456118, EPI_ISL_17456119	Department of Zoology, University of Oxford	Department of Zoology, University of Oxford	da Costa,A.C., Theze,J., Komninakis,S.C.V., Sanz-Duro,R.L., Felinto,M.R.L., Moura,L.C.C., Barroso,I.M.O., Santos,L.E.C., Nunes,M.A.L., Moura,A.A., Lourenco,J., Deng,X., Delwart,E.L., Guimaraes,M.R.A.S., Pybus,O.G., Sabino,E.C. and Faria,N.R.
EPI_ISL_17456120	Department of Zoology, University of Oxford	Department of Zoology, University of Oxford	Naveca,F.G., Claro,I., Giovanetti,M., Jesus,J.G., Xavier,J., do Nascimento,V.A., Iani,F.C.M., de Souza,V.C., Silveira,P.P., Lourenco,J., Santillana,M., Kraemer,M.U.G., Quick,J., Hill,S.C., Theze,J., Wallau,G.L., Carvalho,R.D.O., Azevedo,V., Salles,F.C.S., Candido,D.S., Nunes,M.R.T., Lemos,P.S., Meneses,C.A.R., Maito,R.M., Cunha,C.R.S.B., Campos,D.P.S., Castilho,M.C., Siqueira,T.C.S., Terra,T.M., de Albuquerque,C.F.C., da Cruz,L.N., Abreu,A.L., Simoes,D.S.M.V., Aguiar,R.S., Sabino,E.C., Pybus,O.G., Loman,N., Okumoto,O., Alcantara,L.C.J. and Faria,N.R.
EPI_ISL_17456121, EPI_ISL_17456122, EPI_ISL_17456123, EPI_ISL_17456124, EPI_ISL_17456125, EPI_ISL_17456126, EPI_ISL_17456127	Department of Zoology, University of Oxford	Department of Zoology, University of Oxford	da Costa,A.C., Theze,J., Komninakis,S.C.V., Sanz-Duro,R.L., Felinto,M.R.L., Moura,L.C.C., Barroso,I.M.O., Santos,L.E.C., Nunes,M.A.L., Moura,A.A., Lourenco,J., Deng,X., Delwart,E.L., Guimaraes,M.R.A.S., Pybus,O.G., Sabino,E.C. and Faria,N.R.
EPI_ISL_17456128	Department of Zoology, University of Oxford	Department of Zoology, University of Oxford	Naveca,F.G., Claro,I., Giovanetti,M., Jesus,J.G., Xavier,J., do Nascimento,V.A., Iani,F.C.M., de Souza,V.C., Silveira,P.P., Lourenco,J., Santillana,M., Kraemer,M.U.G., Quick,J., Hill,S.C., Theze,J., Wallau,G.L., Carvalho,R.D.O., Azevedo,V., Salles,F.C.S., Candido,D.S., Nunes,M.R.T., Lemos,P.S., Meneses,C.A.R., Maito,R.M., Cunha,C.R.S.B., Campos,D.P.S., Castilho,M.C., Siqueira,T.C.S., Terra,T.M., de Albuquerque,C.F.C., da Cruz,L.N., Abreu,A.L., Simoes,D.S.M.V., Aguiar,R.S., Sabino,E.C., Pybus,O.G., Loman,N., Okumoto,O., Alcantara,L.C.J. and Faria,N.R.
EPI_ISL_17456129, EPI_ISL_17456130	Department of Zoology, University of Oxford	Department of Zoology, University of Oxford	da Costa,A.C., Theze,J., Komninakis,S.C.V., Sanz-Duro,R.L., Felinto,M.R.L., Moura,L.C.C., Barroso,I.M.O., Santos,L.E.C., Nunes,M.A.L., Moura,A.A., Lourenco,J., Deng,X., Delwart,E.L., Guimaraes,M.R.A.S., Pybus,O.G., Sabino,E.C. and Faria,N.R.
EPI_ISL_17456131, EPI_ISL_17456132	Department of Zoology, University of Oxford	Department of Zoology, University of Oxford	Naveca,F.G., Claro,I., Giovanetti,M., Jesus,J.G., Xavier,J., do Nascimento,V.A., Iani,F.C.M., de Souza,V.C., Silveira,P.P., Lourenco,J., Santillana,M., Kraemer,M.U.G., Quick,J., Hill,S.C., Theze,J., Wallau,G.L., Carvalho,R.D.O., Azevedo,V., Salles,F.C.S., Candido,D.S., Nunes,M.R.T., Lemos,P.S., Meneses,C.A.R., Maito,R.M., Cunha,C.R.S.B., Campos,D.P.S., Castilho,M.C., Siqueira,T.C.S., Terra,T.M., de Albuquerque,C.F.C., da Cruz,L.N., Abreu,A.L., Simoes,D.S.M.V., Aguiar,R.S., Sabino,E.C., Pybus,O.G., Loman,N., Okumoto,O., Alcantara,L.C.J. and Faria,N.R.
EPI_ISL_17456133, EPI_ISL_17456134, EPI_ISL_17456135, EPI_ISL_17456136, EPI_ISL_17456137, EPI_ISL_17456138, EPI_ISL_17456139, EPI_ISL_17456140, EPI_ISL_17456141, EPI_ISL_17456142, EPI_ISL_17456143, EPI_ISL_17456144, EPI_ISL_17456145, EPI_ISL_17456146, EPI_ISL_17456147, EPI_ISL_17456148, EPI_ISL_17456149, EPI_ISL_17456150, EPI_ISL_17456151, EPI_ISL_17456152, EPI_ISL_17456153, EPI_ISL_17456154, EPI_ISL_17456155, EPI_ISL_17456156, EPI_ISL_17456157, EPI_ISL_17456158, EPI_ISL_17456159, EPI_ISL_17456160, EPI_ISL_17456161, EPI_ISL_17456162, EPI_ISL_17456163, EPI_ISL_17456164, EPI_ISL_17456165			
see above	Depatment of Biotechnology, University of Karachi	Depatment of Biotechnology, University of Karachi	Khan,B.A., Khan,S. and Ullah,S.
EPI_ISL_17456166, EPI_ISL_17456167, EPI_ISL_17456168, EPI_ISL_17456169, EPI_ISL_17456170, EPI_ISL_17456171, EPI_ISL_17456172, EPI_ISL_17456173, EPI_ISL_17456174, EPI_ISL_17456175	Detection and Diagnostics Laboratory, DSO National Laboratories	Detection and Diagnostics Laboratory, DSO National Laboratories	Tan,B.-H., Manokaran,G., Chew,S.-W. and Sugrue,R.J.
EPI_ISL_17456176, EPI_ISL_17456177	Diagnostic & Reference Laboratory, Arbovirus Diseases Branch, Centers for Disease Control & Prevention	Diagnostic & Reference Laboratory, Arbovirus Diseases Branch, Centers for Disease Control & Prevention	Lanciotti,R.S. and Valadere,A.M.
EPI_ISL_17456178	Diagnostic & Reference Laboratory, Arbovirus Diseases Branch, Centers for Disease Control & Prevention	Diagnostic & Reference Laboratory, Arbovirus Diseases Branch, Centers for Disease Control & Prevention	Lanciotti,R.S. and Van Cleave,C.
EPI_ISL_17456179	Diagnostic & Reference Laboratory, Arbovirus Diseases Branch, Centers for Disease Control & Prevention	Diagnostic & Reference Laboratory, Arbovirus Diseases Branch, Centers for Disease Control & Prevention	Lanciotti,R.S. and Valadere,A.M.
EPI_ISL_17456180, EPI_ISL_17456181, EPI_ISL_17456182, EPI_ISL_17456183, EPI_ISL_17456184	Division of Vector-Borne Infectious Diseases	Division of Vector-Borne Infectious Diseases	Kariuki Njenga,M., Nderitu,L., Ledermann,J.P., Ndirangu,A., Logue,C.H., Kelly,C.H., Sang,R., Sergon,K., Breiman,R. and Powers,A.M.
EPI_ISL_17456185	Eijkman Institute for Molecular Biology	Eijkman Institute for Molecular Biology	Sasmono,R.T., Perkasa,A., Yohan,B., Haryanto,S., Yudhaputri,F.A., Hayati,R.F., Ma'roef,C.N., Ledermann,J.P., Aye Myint,K.S. and Powers,A.M.
EPI_ISL_17456186	Eijkman Institute for Molecular Biology	Eijkman Institute for Molecular Biology	Sari,N.L.P.E.K., Jaya,U.A., Andayani,A.R., Adi,P.D., Dhenni,R., Perkasa,A., Ma'roef,C.N., Witari,N.P.D., Megawati,A.D., Myint,K.S. and Powers,A.M.
EPI_ISL_17456187	Eijkman Institute for Molecular Biology	Eijkman Institute for Molecular Biology	Santoso,M.S., Haryanto,S., Rulian,F., Hayati,R.F., Kristiani,A., Kartika,R., Yohan,B., Hibberd,M.L. and Sasmono,R.T.
EPI_ISL_17456188	Eijkman Institute for Molecular Biology	Eijkman Institute for Molecular Biology	Sasmono,R.T., Perkasa,A., Yohan,B., Haryanto,S., Yudhaputri,F.A., Hayati,R.F., Ma'roef,C.N., Ledermann,J.P., Aye Myint,K.S. and Powers,A.M.
EPI_ISL_17456189	Eijkman Institute for Molecular Biology	Eijkman Institute for Molecular Biology	Sari,N.L.P.E.K., Jaya,U.A., Andayani,A.R., Adi,P.D., Dhenni,R., Perkasa,A., Ma'roef,C.N., Witari,N.P.D., Megawati,A.D., Myint,K.S. and Powers,A.M.
EPI_ISL_17456190, EPI_ISL_17456191	Eijkman Institute for Molecular Biology	Eijkman Institute for Molecular Biology	Santoso,M.S., Haryanto,S., Rulian,F., Hayati,R.F., Kristiani,A., Kartika,R., Yohan,B., Hibberd,M.L. and Sasmono,R.T.
EPI_ISL_17456192	Eijkman Institute for Molecular Biology	Eijkman Institute for Molecular Biology	Sasmono,R.T., Perkasa,A., Yohan,B., Haryanto,S., Yudhaputri,F.A., Hayati,R.F., Ma'roef,C.N., Ledermann,J.P., Aye Myint,K.S. and Powers,A.M.
EPI_ISL_17456193	Eijkman Institute for Molecular Biology	Eijkman Institute for Molecular Biology	Sari,N.L.P.E.K., Jaya,U.A., Andayani,A.R., Adi,P.D., Dhenni,R., Perkasa,A., Ma'roef,C.N., Witari,N.P.D., Megawati,A.D., Myint,K.S. and Powers,A.M.
EPI_ISL_17456194	Eijkman Institute for Molecular Biology	Eijkman Institute for Molecular Biology	Sasmono,R.T., Perkasa,A., Yohan,B., Haryanto,S., Yudhaputri,F.A., Hayati,R.F., Ma'roef,C.N., Ledermann,J.P., Aye Myint,K.S. and Powers,A.M.
EPI_ISL_17456195	Eijkman Institute for Molecular Biology	Eijkman Institute for Molecular Biology	Sari,N.L.P.E.K., Jaya,U.A., Andayani,A.R., Adi,P.D., Dhenni,R., Perkasa,A., Ma'roef,C.N., Witari,N.P.D., Megawati,A.D., Myint,K.S. and Powers,A.M.
EPI_ISL_17456196, EPI_ISL_17456197, EPI_ISL_17456198	Eijkman Institute for Molecular Biology	Eijkman Institute for Molecular Biology	Sasmono,R.T., Perkasa,A., Yohan,B., Haryanto,S., Yudhaputri,F.A., Hayati,R.F., Ma'roef,C.N., Ledermann,J.P., Aye Myint,K.S. and Powers,A.M.
EPI_ISL_17456199	Eijkman Institute for Molecular Biology	Eijkman Institute for Molecular Biology	Sari,N.L.P.E.K., Jaya,U.A., Andayani,A.R., Adi,P.D., Dhenni,R., Perkasa,A., Ma'roef,C.N., Witari,N.P.D., Megawati,A.D., Myint,K.S. and Powers,A.M.
EPI_ISL_17456200	Eijkman Institute for Molecular Biology	Eijkman Institute for Molecular Biology	Sasmono,R.T., Perkasa,A., Yohan,B., Haryanto,S., Yudhaputri,F.A., Hayati,R.F., Ma'roef,C.N., Ledermann,J.P., Aye Myint,K.S. and Powers,A.M.
EPI_ISL_17456201	Eijkman Institute for Molecular Biology	Eijkman Institute for Molecular Biology	Sari,N.L.P.E.K., Jaya,U.A., Andayani,A.R., Adi,P.D., Dhenni,R., Perkasa,A., Ma'roef,C.N., Witari,N.P.D., Megawati,A.D., Myint,K.S. and Powers,A.M.
EPI_ISL_17456202	Environmental Health Institute, National	Environmental Health Institute, National	Hapuarachchi,H.C., Bandara,K.B., Sumanadasa,S.D., Hapugoda,M.D., Lai,Y.L., Lee,K.S., Tan,L.K., Lin,R.T., Ng,L.F., Bucht,G., Abeyewickreme,W. and Ng,L.C.

	Environment Agency	Environment Agency	
EPI_ISL_17456203	Environmental Health Institute, National Environment Agency	Environmental Health Institute, National Environment Agency	Hapuarachchi,H.C., Wong,W.Y., Koo,C., Tien,W.P., Lu,T.Y., Yeo,G., Rajarethinam,J., Tan,E., Chong,C.S., Tan,C.H., Yap,G., Tan,L.K. and Ng,L.C.
EPI_ISL_17456204, EPI_ISL_17456205	Environmental Health Institute, National Environment Agency	Environmental Health Institute, National Environment Agency	Hapuarachchi,H.C., Bandara,K.B., Sumanadasa,S.D., Hapugoda,M.D., Lai,Y.L., Lee,K.S., Tan,L.K., Lin,R.T., Ng,L.F., Bucht,G., Abeyewickreme,W. and Ng,L.C.
EPI_ISL_17456206	Environmental Health Institute, National Environment Agency	Environmental Health Institute, National Environment Agency	Hapuarachchi,H.C., Wong,W.Y., Koo,C., Tien,W.P., Lu,T.Y., Yeo,G., Rajarethinam,J., Tan,E., Chong,C.S., Tan,C.H., Yap,G., Tan,L.K. and Ng,L.C.
EPI_ISL_17456207	Environmental Health Institute, National Environment Agency	Environmental Health Institute, National Environment Agency	Hapuarachchi,H.C., Bandara,K.B., Sumanadasa,S.D., Hapugoda,M.D., Lai,Y.L., Lee,K.S., Tan,L.K., Lin,R.T., Ng,L.F., Bucht,G., Abeyewickreme,W. and Ng,L.C.
EPI_ISL_17456208	Environmental Health Institute, National Environment Agency	Environmental Health Institute, National Environment Agency	Hapuarachchi,H.C., Wong,W.Y., Koo,C., Tien,W.P., Lu,T.Y., Yeo,G., Rajarethinam,J., Tan,E., Chong,C.S., Tan,C.H., Yap,G., Tan,L.K. and Ng,L.C.
EPI_ISL_17456209, EPI_ISL_17456210, EPI_ISL_17456211	Environmental Health Institute, National Environment Agency	Environmental Health Institute, National Environment Agency	Hapuarachchi,H.C., Bandara,K.B., Sumanadasa,S.D., Hapugoda,M.D., Lai,Y.L., Lee,K.S., Tan,L.K., Lin,R.T., Ng,L.F., Bucht,G., Abeyewickreme,W. and Ng,L.C.
EPI_ISL_17456212	Environmental Health Institute, National Environment Agency	Environmental Health Institute, National Environment Agency	Hapuarachchi,H.C., Wong,W.Y., Koo,C., Tien,W.P., Lu,T.Y., Yeo,G., Rajarethinam,J., Tan,E., Chong,C.S., Tan,C.H., Yap,G., Tan,L.K. and Ng,L.C.
EPI_ISL_17456213, EPI_ISL_17456214, EPI_ISL_17456215	Environmental Health Institute, National Environment Agency	Environmental Health Institute, National Environment Agency	Hapuarachchi,H.C., Bandara,K.B., Sumanadasa,S.D., Hapugoda,M.D., Lai,Y.L., Lee,K.S., Tan,L.K., Lin,R.T., Ng,L.F., Bucht,G., Abeyewickreme,W. and Ng,L.C.
EPI_ISL_17456216, EPI_ISL_17456217, EPI_ISL_17456218, EPI_ISL_17456219, EPI_ISL_17456220, EPI_ISL_17456221	Environmental Health Institute, National Environment Agency	Environmental Health Institute, National Environment Agency	Hapuarachchi,H.C., Wong,W.Y., Koo,C., Tien,W.P., Lu,T.Y., Yeo,G., Rajarethinam,J., Tan,E., Chong,C.S., Tan,C.H., Yap,G., Tan,L.K. and Ng,L.C.
EPI_ISL_17456222	Environmental Health Institute, National Environment Agency	Environmental Health Institute, National Environment Agency	Hapuarachchi,H.C., Bandara,K.B., Sumanadasa,S.D., Hapugoda,M.D., Lai,Y.L., Lee,K.S., Tan,L.K., Lin,R.T., Ng,L.F., Bucht,G., Abeyewickreme,W. and Ng,L.C.
EPI_ISL_17456223	Environmental Health Institute, National Environment Agency	Environmental Health Institute, National Environment Agency	Hapuarachchi,H.C., Wong,W.Y., Koo,C., Tien,W.P., Lu,T.Y., Yeo,G., Rajarethinam,J., Tan,E., Chong,C.S., Tan,C.H., Yap,G., Tan,L.K. and Ng,L.C.
EPI_ISL_17456224	Environmental Health Institute, National Environment Agency	Environmental Health Institute, National Environment Agency	Hapuarachchi,H.C., Bandara,K.B., Sumanadasa,S.D., Hapugoda,M.D., Lai,Y.L., Lee,K.S., Tan,L.K., Lin,R.T., Ng,L.F., Bucht,G., Abeyewickreme,W. and Ng,L.C.
EPI_ISL_17456225, EPI_ISL_17456226, EPI_ISL_17456227, EPI_ISL_17456228, EPI_ISL_17456229, EPI_ISL_17456230	Environmental Health Institute, National Environment Agency	Environmental Health Institute, National Environment Agency	Hapuarachchi,H.C., Wong,W.Y., Koo,C., Tien,W.P., Lu,T.Y., Yeo,G., Rajarethinam,J., Tan,E., Chong,C.S., Tan,C.H., Yap,G., Tan,L.K. and Ng,L.C.
EPI_ISL_17456231	Environmental Health Institute, National Environment Agency	Environmental Health Institute, National Environment Agency	Hapuarachchi,H.C., Bandara,K.B., Sumanadasa,S.D., Hapugoda,M.D., Lai,Y.L., Lee,K.S., Tan,L.K., Lin,R.T., Ng,L.F., Bucht,G., Abeyewickreme,W. and Ng,L.C.
EPI_ISL_17456232, EPI_ISL_17456233, EPI_ISL_17456234	Environmental Health Institute, National Environment Agency	Environmental Health Institute, National Environment Agency	Hapuarachchi,H.C., Wong,W.Y., Koo,C., Tien,W.P., Lu,T.Y., Yeo,G., Rajarethinam,J., Tan,E., Chong,C.S., Tan,C.H., Yap,G., Tan,L.K. and Ng,L.C.
EPI_ISL_17456235	Environmental Health Institute, National Environment Agency	Environmental Health Institute, National Environment Agency	Hapuarachchi,H.C., Bandara,K.B., Sumanadasa,S.D., Hapugoda,M.D., Lai,Y.L., Lee,K.S., Tan,L.K., Lin,R.T., Ng,L.F., Bucht,G., Abeyewickreme,W. and Ng,L.C.
EPI_ISL_17456236, EPI_ISL_17456237, EPI_ISL_17456238	Environmental Health Institute, National Environment Agency	Environmental Health Institute, National Environment Agency	Hapuarachchi,H.C., Wong,W.Y., Koo,C., Tien,W.P., Lu,T.Y., Yeo,G., Rajarethinam,J., Tan,E., Chong,C.S., Tan,C.H., Yap,G., Tan,L.K. and Ng,L.C.
EPI_ISL_17456239	Environmental Health Institute, National Environment Agency	Environmental Health Institute, National Environment Agency	Hapuarachchi,H.C., Bandara,K.B., Sumanadasa,S.D., Hapugoda,M.D., Lai,Y.L., Lee,K.S., Tan,L.K., Lin,R.T., Ng,L.F., Bucht,G., Abeyewickreme,W. and Ng,L.C.
EPI_ISL_17456240, EPI_ISL_17456241, EPI_ISL_17456242, EPI_ISL_17456243, EPI_ISL_17456244, EPI_ISL_17456245, EPI_ISL_17456246	Environmental Health Institute, National Environment Agency	Environmental Health Institute, National Environment Agency	Hapuarachchi,H.C., Wong,W.Y., Koo,C., Tien,W.P., Lu,T.Y., Yeo,G., Rajarethinam,J., Tan,E., Chong,C.S., Tan,C.H., Yap,G., Tan,L.K. and Ng,L.C.
EPI_ISL_17456247	Environmental Health Institute, National Environment Agency	Environmental Health Institute, National Environment Agency	Hapuarachchi,H.C., Bandara,K.B., Sumanadasa,S.D., Hapugoda,M.D., Lai,Y.L., Lee,K.S., Tan,L.K., Lin,R.T., Ng,L.F., Bucht,G., Abeyewickreme,W. and Ng,L.C.
EPI_ISL_17456248, EPI_ISL_17456249	Environmental Health Institute, National Environment Agency	Environmental Health Institute, National Environment Agency	Hapuarachchi,H.C., Wong,W.Y., Koo,C., Tien,W.P., Lu,T.Y., Yeo,G., Rajarethinam,J., Tan,E., Chong,C.S., Tan,C.H., Yap,G., Tan,L.K. and Ng,L.C.
EPI_ISL_17456250	Environmental Health Institute, National Environment Agency	Environmental Health Institute, National Environment Agency	Hapuarachchi,H.C., Bandara,K.B., Sumanadasa,S.D., Hapugoda,M.D., Lai,Y.L., Lee,K.S., Tan,L.K., Lin,R.T., Ng,L.F., Bucht,G., Abeyewickreme,W. and Ng,L.C.
EPI_ISL_17456251, EPI_ISL_17456252, EPI_ISL_17456253, EPI_ISL_17456254, EPI_ISL_17456255	Environmental Health Institute, National Environment Agency	Environmental Health Institute, National Environment Agency	Hapuarachchi,H.C., Wong,W.Y., Koo,C., Tien,W.P., Lu,T.Y., Yeo,G., Rajarethinam,J., Tan,E., Chong,C.S., Tan,C.H., Yap,G., Tan,L.K. and Ng,L.C.
EPI_ISL_17456256, EPI_ISL_17456257, EPI_ISL_17456258, EPI_ISL_17456259, EPI_ISL_17456260, EPI_ISL_17456261, EPI_ISL_17456262, EPI_ISL_17456263, EPI_ISL_17456264, EPI_ISL_17456265, EPI_ISL_17456266			
see above	Environmental Health Institute, National Environment Agency	Environmental Health Institute, National Environment Agency	Hapuarachchi,H.C., Bandara,K.B., Sumanadasa,S.D., Hapugoda,M.D., Lai,Y.L., Lee,K.S., Tan,L.K., Lin,R.T., Ng,L.F., Bucht,G., Abeyewickreme,W. and Ng,L.C.
EPI_ISL_17456267	Environmental Health Institute, National Environment Agency	Environmental Health Institute, National Environment Agency	Ng,L.C., Tan,L.K., Tan,C.H., Tan,S.S., Hapuarachchi,H.C., Pok,K.Y., Lai,Y.L., Lam-Phua,S.G., Bucht,G., Lin,R.T., Leo,Y.S., Tan,B.H., Han,H.K., Ooi,P.L., James,L. and Khoo,S.P.
EPI_ISL_17456268, EPI_ISL_17456269, EPI_ISL_17456270, EPI_ISL_17456271, EPI_ISL_17456272, EPI_ISL_17456273, EPI_ISL_17456274, EPI_ISL_17456275, EPI_ISL_17456276, EPI_ISL_17456277, EPI_ISL_17456278, EPI_ISL_17456279, EPI_ISL_17456280			
see above	Environmental Health Institute, National Environment Agency	Environmental Health Institute, National Environment Agency	Hapuarachchi,H.C., Wong,W.Y., Koo,C., Tien,W.P., Lu,T.Y., Yeo,G., Rajarethinam,J., Tan,E., Chong,C.S., Tan,C.H., Yap,G., Tan,L.K. and Ng,L.C.
EPI_ISL_17456281, EPI_ISL_17456282	Environmental Health Institute, National Environment Agency	Environmental Health Institute, National Environment Agency	Ng,L.C., Tan,L.K., Tan,C.H., Tan,S.S., Hapuarachchi,H.C., Pok,K.Y., Lai,Y.L., Lam-Phua,S.G., Bucht,G., Lin,R.T., Leo,Y.S., Tan,B.H., Han,H.K., Ooi,P.L., James,L. and Khoo,S.P.
EPI_ISL_17456283	Environmental Health Institute, National Environment Agency	Environmental Health Institute, National Environment Agency	Hapuarachchi,H.C., Wong,W.Y., Koo,C., Tien,W.P., Lu,T.Y., Yeo,G., Rajarethinam,J., Tan,E., Chong,C.S., Tan,C.H., Yap,G., Tan,L.K. and Ng,L.C.
EPI_ISL_17456284	Environmental Health Institute, National Environment Agency	Environmental Health Institute, National Environment Agency	Ng,L.C., Tan,L.K., Tan,C.H., Tan,S.S., Hapuarachchi,H.C., Pok,K.Y., Lai,Y.L., Lam-Phua,S.G., Bucht,G., Lin,R.T., Leo,Y.S., Tan,B.H., Han,H.K., Ooi,P.L., James,L. and Khoo,S.P.
EPI_ISL_17456285, EPI_ISL_17456286, EPI_ISL_17456287, EPI_ISL_17456288, EPI_ISL_17456289, EPI_ISL_17456290, EPI_ISL_17456291, EPI_ISL_17456292, EPI_ISL_17456293, EPI_ISL_17456294, EPI_ISL_17456295, EPI_ISL_17456296, EPI_ISL_17456297, EPI_ISL_17456298, EPI_ISL_17456299, EPI_ISL_17456300, EPI_ISL_17456301, EPI_ISL_17456302, EPI_ISL_17456303, EPI_ISL_17456304, EPI_ISL_17456305, EPI_ISL_17456306, EPI_ISL_17456307, EPI_ISL_17456308, EPI_ISL_17456309, EPI_ISL_17456310, EPI_ISL_17456311, EPI_ISL_17456312, EPI_ISL_17456313, EPI_ISL_17456314, EPI_ISL_17456315			
see above	Environmental Health Institute, National Environment Agency	Environmental Health Institute, National Environment Agency	Hapuarachchi,H.C., Wong,W.Y., Koo,C., Tien,W.P., Lu,T.Y., Yeo,G., Rajarethinam,J., Tan,E., Chong,C.S., Tan,C.H., Yap,G., Tan,L.K. and Ng,L.C.
EPI_ISL_17456316	Environmental Health Institute, National Environment Agency	Environmental Health Institute, National Environment Agency	Tan,C.H., Wong,P.J., Li,M.I., Yang,H. and Ng,L.C.
EPI_ISL_17456317	Environmental Health Institute, National Environment Agency	Environmental Health Institute, National Environment Agency	Hapuarachchi,H.C., Wong,W.Y., Koo,C., Tien,W.P., Lu,T.Y., Yeo,G., Rajarethinam,J., Tan,E., Chong,C.S., Tan,C.H., Yap,G., Tan,L.K. and Ng,L.C.
EPI_ISL_17456318	Environmental Health Institute, National Environment Agency	Environmental Health Institute, National Environment Agency	Hapuarachchi,H.C., Bandara,K.B., Sumanadasa,S.D., Hapugoda,M.D., Lai,Y.L., Lee,K.S., Tan,L.K., Lin,R.T., Ng,L.F., Bucht,G., Abeyewickreme,W. and Ng,L.C.
EPI_ISL_17456319, EPI_ISL_17456320, EPI_ISL_17456321, EPI_ISL_17456322	Environmental Health Institute, National Environment Agency	Environmental Health Institute, National Environment Agency	Hapuarachchi,H.C., Wong,W.Y., Koo,C., Tien,W.P., Lu,T.Y., Yeo,G., Rajarethinam,J., Tan,E., Chong,C.S., Tan,C.H., Yap,G., Tan,L.K. and Ng,L.C.
EPI_ISL_17456323, EPI_ISL_17456324	Environmental Health Institute, National Environment Agency	Environmental Health Institute, National Environment Agency	Ng,L.C., Tan,L.K., Tan,C.H., Tan,S.S., Hapuarachchi,H.C., Pok,K.Y., Lai,Y.L., Lam-Phua,S.G., Bucht,G., Lin,R.T., Leo,Y.S., Tan,B.H., Han,H.K., Ooi,P.L., James,L. and Khoo,S.P.
EPI_ISL_17456325	Environmental Health Institute, National Environment Agency	Environmental Health Institute, National Environment Agency	Hapuarachchi,H.C., Wong,W.Y., Koo,C., Tien,W.P., Lu,T.Y., Yeo,G., Rajarethinam,J., Tan,E., Chong,C.S., Tan,C.H., Yap,G., Tan,L.K. and Ng,L.C.
EPI_ISL_17456326	Environmental Health Institute, National Environment Agency	Environmental Health Institute, National Environment Agency	Hapuarachchi,H.C., Bandara,K.B., Sumanadasa,S.D., Hapugoda,M.D., Lai,Y.L., Lee,K.S., Tan,L.K., Lin,R.T., Ng,L.F., Bucht,G., Abeyewickreme,W. and Ng,L.C.
EPI_ISL_17456327	Environmental Health Institute, National Environment Agency	Environmental Health Institute, National Environment Agency	Ng,L.C., Tan,L.K., Tan,C.H., Tan,S.S., Hapuarachchi,H.C., Pok,K.Y., Lai,Y.L., Lam-Phua,S.G., Bucht,G., Lin,R.T., Leo,Y.S., Tan,B.H., Han,H.K., Ooi,P.L., James,L. and Khoo,S.P.
EPI_ISL_17456328, EPI_ISL_17456329	Environmental Health Institute, National Environment Agency	Environmental Health Institute, National Environment Agency	Hapuarachchi,H.C., Wong,W.Y., Koo,C., Tien,W.P., Lu,T.Y., Yeo,G., Rajarethinam,J., Tan,E., Chong,C.S., Tan,C.H., Yap,G., Tan,L.K. and Ng,L.C.
EPI_ISL_17456330	Environmental Health Institute, National Environment Agency	Environmental Health Institute, National Environment Agency	Hapuarachchi,H.C., Koo,C., Kek,R. and Ng,L.C.
EPI_ISL_17456331, EPI_ISL_17456332, EPI_ISL_17456333	Environmental Health Institute, National Environment Agency	Environmental Health Institute, National Environment Agency	Hapuarachchi,H.C., Wong,W.Y., Koo,C., Tien,W.P., Lu,T.Y., Yeo,G., Rajarethinam,J., Tan,E., Chong,C.S., Tan,C.H., Yap,G., Tan,L.K. and Ng,L.C.
EPI_ISL_17456334	Environmental Health Institute, National Environment Agency	Environmental Health Institute, National Environment Agency	Ng,L.C., Tan,L.K., Tan,C.H., Tan,S.S., Hapuarachchi,H.C., Pok,K.Y., Lai,Y.L., Lam-Phua,S.G., Bucht,G., Lin,R.T., Leo,Y.S., Tan,B.H., Han,H.K., Ooi,P.L., James,L. and Khoo,S.P.

EPI_ISL_17456335	Environmental Health Institute, National Environment Agency	Environmental Health Institute, National Environment Agency	Hapuarachchi,H.C., Bandara,K.B., Sumanadasa,S.D., Hapugoda,M.D., Lai,Y.L., Lee,K.S., Tan,L.K., Lin,R.T., Ng,L.F., Bucht,G., Abeyewickreme,W. and Ng,L.C.
EPI_ISL_17456336	Environmental Health Institute, National Environment Agency	Environmental Health Institute, National Environment Agency	Hapuarachchi,H.C., Wong,W.Y., Koo,C., Tien,W.P., Lu,T.Y., Yeo,G., Rajarethinam,J., Tan,E., Chong,C.S., Tan,C.H., Yap,G., Tan,L.K. and Ng,L.C.
EPI_ISL_17456337	Environmental Health Institute, National Environment Agency	Environmental Health Institute, National Environment Agency	Hapuarachchi,H.C., Bandara,K.B., Sumanadasa,S.D., Hapugoda,M.D., Lai,Y.L., Lee,K.S., Tan,L.K., Lin,R.T., Ng,L.F., Bucht,G., Abeyewickreme,W. and Ng,L.C.
EPI_ISL_17456338	Environmental Health Institute, National Environment Agency	Environmental Health Institute, National Environment Agency	Ng,L.C., Tan,L.K., Tan,C.H., Tan,S.S., Hapuarachchi,H.C., Pok,K.Y., Lai,Y.L., Lam-Phua,S.G., Bucht,G., Lin,R.T., Leo,Y.S., Tan,B.H., Han,H.K., Ooi,P.L., James,L. and Khoo,S.P.
EPI_ISL_17456339, EPI_ISL_17456340, EPI_ISL_17456341	Environmental Health Institute, National Environment Agency	Environmental Health Institute, National Environment Agency	Hapuarachchi,H.C., Wong,W.Y., Koo,C., Tien,W.P., Lu,T.Y., Yeo,G., Rajarethinam,J., Tan,E., Chong,C.S., Tan,C.H., Yap,G., Tan,L.K. and Ng,L.C.
EPI_ISL_17456342	Environmental Health Institute, National Environment Agency	Environmental Health Institute, National Environment Agency	Ng,L.C., Tan,L.K., Tan,C.H., Tan,S.S., Hapuarachchi,H.C., Pok,K.Y., Lai,Y.L., Lam-Phua,S.G., Bucht,G., Lin,R.T., Leo,Y.S., Tan,B.H., Han,H.K., Ooi,P.L., James,L. and Khoo,S.P.
EPI_ISL_17456343, EPI_ISL_17456344, EPI_ISL_17456345, EPI_ISL_17456346, EPI_ISL_17456347, EPI_ISL_17456348	Environmental Health Institute, National Environment Agency	Environmental Health Institute, National Environment Agency	Hapuarachchi,H.C., Wong,W.Y., Koo,C., Tien,W.P., Lu,T.Y., Yeo,G., Rajarethinam,J., Tan,E., Chong,C.S., Tan,C.H., Yap,G., Tan,L.K. and Ng,L.C.
EPI_ISL_17456349, EPI_ISL_17456350, EPI_ISL_17456351	Environmental Health Institute, National Environment Agency	Environmental Health Institute, National Environment Agency	Ng,L.C., Tan,L.K., Tan,C.H., Tan,S.S., Hapuarachchi,H.C., Pok,K.Y., Lai,Y.L., Lam-Phua,S.G., Bucht,G., Lin,R.T., Leo,Y.S., Tan,B.H., Han,H.K., Ooi,P.L., James,L. and Khoo,S.P.
EPI_ISL_17456352	Environmental Health Institute, National Environment Agency	Environmental Health Institute, National Environment Agency	Hapuarachchi,H.C., Wong,W.Y., Koo,C., Tien,W.P., Lu,T.Y., Yeo,G., Rajarethinam,J., Tan,E., Chong,C.S., Tan,C.H., Yap,G., Tan,L.K. and Ng,L.C.
EPI_ISL_17456353	Environmental Health Institute, National Environment Agency	Environmental Health Institute, National Environment Agency	Ng,L.C., Tan,L.K., Tan,C.H., Tan,S.S., Hapuarachchi,H.C., Pok,K.Y., Lai,Y.L., Lam-Phua,S.G., Bucht,G., Lin,R.T., Leo,Y.S., Tan,B.H., Han,H.K., Ooi,P.L., James,L. and Khoo,S.P.
EPI_ISL_17456354, EPI_ISL_17456355, EPI_ISL_17456356, EPI_ISL_17456357, EPI_ISL_17456358, EPI_ISL_17456359, EPI_ISL_17456360	Environmental Health Institute, National Environment Agency	Environmental Health Institute, National Environment Agency	Hapuarachchi,H.C., Wong,W.Y., Koo,C., Tien,W.P., Lu,T.Y., Yeo,G., Rajarethinam,J., Tan,E., Chong,C.S., Tan,C.H., Yap,G., Tan,L.K. and Ng,L.C.
EPI_ISL_17456361, EPI_ISL_17456362, EPI_ISL_17456363, EPI_ISL_17456364	Environmental Health Institute, National Environment Agency	Environmental Health Institute, National Environment Agency	Hapuarachchi,H.C., Bandara,K.B., Sumanadasa,S.D., Hapugoda,M.D., Lai,Y.L., Lee,K.S., Tan,L.K., Lin,R.T., Ng,L.F., Bucht,G., Abeyewickreme,W. and Ng,L.C.
EPI_ISL_17456365, EPI_ISL_17456366, EPI_ISL_17456367, EPI_ISL_17456368, EPI_ISL_17456369, EPI_ISL_17456370, EPI_ISL_17456371, EPI_ISL_17456372	Environmental Health Institute, National Environment Agency	Environmental Health Institute, National Environment Agency	Hapuarachchi,H.C., Wong,W.Y., Koo,C., Tien,W.P., Lu,T.Y., Yeo,G., Rajarethinam,J., Tan,E., Chong,C.S., Tan,C.H., Yap,G., Tan,L.K. and Ng,L.C.
EPI_ISL_17456373, EPI_ISL_17456374, EPI_ISL_17456375, EPI_ISL_17456376, EPI_ISL_17456377, EPI_ISL_17456378, EPI_ISL_17456379, EPI_ISL_17456380, EPI_ISL_17456381, EPI_ISL_17456382, EPI_ISL_17456383			
see above	Environmental Health Institute, National Environment Agency	Environmental Health Institute, National Environment Agency	Ng,L.C., Tan,L.K., Tan,C.H., Tan,S.S., Hapuarachchi,H.C., Pok,K.Y., Lai,Y.L., Lam-Phua,S.G., Bucht,G., Lin,R.T., Leo,Y.S., Tan,B.H., Han,H.K., Ooi,P.L., James,L. and Khoo,S.P.
EPI_ISL_17456384	Environmental Health Institute, National Environment Agency	Environmental Health Institute, National Environment Agency	Hapuarachchi,H.C., Wong,W.Y., Koo,C., Tien,W.P., Lu,T.Y., Yeo,G., Rajarethinam,J., Tan,E., Chong,C.S., Tan,C.H., Yap,G., Tan,L.K. and Ng,L.C.
EPI_ISL_17456385	Environmental Health Institute, National Environment Agency	Environmental Health Institute, National Environment Agency	Ng,L.C., Tan,L.K., Tan,C.H., Tan,S.S., Hapuarachchi,H.C., Pok,K.Y., Lai,Y.L., Lam-Phua,S.G., Bucht,G., Lin,R.T., Leo,Y.S., Tan,B.H., Han,H.K., Ooi,P.L., James,L. and Khoo,S.P.
EPI_ISL_17456386	Environmental Health Institute, National Environment Agency	Environmental Health Institute, National Environment Agency	Hapuarachchi,H.C., Wong,W.Y., Koo,C., Tien,W.P., Lu,T.Y., Yeo,G., Rajarethinam,J., Tan,E., Chong,C.S., Tan,C.H., Yap,G., Tan,L.K. and Ng,L.C.
EPI_ISL_17456387, EPI_ISL_17456388, EPI_ISL_17456389, EPI_ISL_17456390, EPI_ISL_17456391	Environmental Health Institute, National Environment Agency	Environmental Health Institute, National Environment Agency	Ng,L.C., Tan,L.K., Tan,C.H., Tan,S.S., Hapuarachchi,H.C., Pok,K.Y., Lai,Y.L., Lam-Phua,S.G., Bucht,G., Lin,R.T., Leo,Y.S., Tan,B.H., Han,H.K., Ooi,P.L., James,L. and Khoo,S.P.
EPI_ISL_17456392	Environmental Health Institute, National Environment Agency	Environmental Health Institute, National Environment Agency	Hapuarachchi,H.C., Wong,W.Y., Koo,C., Tien,W.P., Lu,T.Y., Yeo,G., Rajarethinam,J., Tan,E., Chong,C.S., Tan,C.H., Yap,G., Tan,L.K. and Ng,L.C.
EPI_ISL_17456393, EPI_ISL_17456394, EPI_ISL_17456395, EPI_ISL_17456396	Environmental Health Institute, National Environment Agency	Environmental Health Institute, National Environment Agency	Ng,L.C., Tan,L.K., Tan,C.H., Tan,S.S., Hapuarachchi,H.C., Pok,K.Y., Lai,Y.L., Lam-Phua,S.G., Bucht,G., Lin,R.T., Leo,Y.S., Tan,B.H., Han,H.K., Ooi,P.L., James,L. and Khoo,S.P.
EPI_ISL_17456397	Environmental Health Institute, National Environment Agency	Environmental Health Institute, National Environment Agency	Hapuarachchi,H.C., Wong,W.Y., Koo,C., Tien,W.P., Lu,T.Y., Yeo,G., Rajarethinam,J., Tan,E., Chong,C.S., Tan,C.H., Yap,G., Tan,L.K. and Ng,L.C.
EPI_ISL_17456398	Environmental Health Institute, National Environment Agency	Environmental Health Institute, National Environment Agency	Ng,L.C., Tan,L.K., Tan,C.H., Tan,S.S., Hapuarachchi,H.C., Pok,K.Y., Lai,Y.L., Lam-Phua,S.G., Bucht,G., Lin,R.T., Leo,Y.S., Tan,B.H., Han,H.K., Ooi,P.L., James,L. and Khoo,S.P.
EPI_ISL_17456399, EPI_ISL_17456400, EPI_ISL_17456401	Environmental Health Institute, National Environment Agency	Environmental Health Institute, National Environment Agency	Hapuarachchi,H.C., Wong,W.Y., Koo,C., Tien,W.P., Lu,T.Y., Yeo,G., Rajarethinam,J., Tan,E., Chong,C.S., Tan,C.H., Yap,G., Tan,L.K. and Ng,L.C.
EPI_ISL_17456402	Environmental Health Institute, National Environment Agency	Environmental Health Institute, National Environment Agency	Ng,L.C., Tan,L.K., Tan,C.H., Tan,S.S., Hapuarachchi,H.C., Pok,K.Y., Lai,Y.L., Lam-Phua,S.G., Bucht,G., Lin,R.T., Leo,Y.S., Tan,B.H., Han,H.K., Ooi,P.L., James,L. and Khoo,S.P.
EPI_ISL_17456403, EPI_ISL_17456404, EPI_ISL_17456405, EPI_ISL_17456406, EPI_ISL_17456407, EPI_ISL_17456408, EPI_ISL_17456409, EPI_ISL_17456410, EPI_ISL_17456411, EPI_ISL_17456412, EPI_ISL_17456413			
see above	Environmental Health Institute, National Environment Agency	Environmental Health Institute, National Environment Agency	Hapuarachchi,H.C., Wong,W.Y., Koo,C., Tien,W.P., Lu,T.Y., Yeo,G., Rajarethinam,J., Tan,E., Chong,C.S., Tan,C.H., Yap,G., Tan,L.K. and Ng,L.C.
EPI_ISL_17456414	Environmental Health Institute, National Environment Agency	Environmental Health Institute, National Environment Agency	Hapuarachchi,H.C., Bandara,K.B., Sumanadasa,S.D., Hapugoda,M.D., Lai,Y.L., Lee,K.S., Tan,L.K., Lin,R.T., Ng,L.F., Bucht,G., Abeyewickreme,W. and Ng,L.C.
EPI_ISL_17456415, EPI_ISL_17456416	Environmental Health Institute, National Environment Agency	Environmental Health Institute, National Environment Agency	Hapuarachchi,H.C., Wong,W.Y., Koo,C., Tien,W.P., Lu,T.Y., Yeo,G., Rajarethinam,J., Tan,E., Chong,C.S., Tan,C.H., Yap,G., Tan,L.K. and Ng,L.C.
EPI_ISL_17456417, EPI_ISL_17456418, EPI_ISL_17456419, EPI_ISL_17456420, EPI_ISL_17456421, EPI_ISL_17456422, EPI_ISL_17456423, EPI_ISL_17456424, EPI_ISL_17456425, EPI_ISL_17456426	Environmental Health Institute, National Environment Agency	Environmental Health Institute, National Environment Agency	Hapuarachchi,H.C., Bandara,K.B., Sumanadasa,S.D., Hapugoda,M.D., Lai,Y.L., Lee,K.S., Tan,L.K., Lin,R.T., Ng,L.F., Bucht,G., Abeyewickreme,W. and Ng,L.C.
EPI_ISL_17456427	Environmental Health Institute, National Environment Agency	Environmental Health Institute, National Environment Agency	Hapuarachchi,H.C., Wong,W.Y., Koo,C., Tien,W.P., Lu,T.Y., Yeo,G., Rajarethinam,J., Tan,E., Chong,C.S., Tan,C.H., Yap,G., Tan,L.K. and Ng,L.C.
EPI_ISL_17456428, EPI_ISL_17456429	Environmental Health Institute, National Environment Agency	Environmental Health Institute, National Environment Agency	Hapuarachchi,H.C., Bandara,K.B., Sumanadasa,S.D., Hapugoda,M.D., Lai,Y.L., Lee,K.S., Tan,L.K., Lin,R.T., Ng,L.F., Bucht,G., Abeyewickreme,W. and Ng,L.C.
EPI_ISL_17456430, EPI_ISL_17456431, EPI_ISL_17456432, EPI_ISL_17456433, EPI_ISL_17456434	Environmental Health Institute, National Environment Agency	Environmental Health Institute, National Environment Agency	Hapuarachchi,H.C., Wong,W.Y., Koo,C., Tien,W.P., Lu,T.Y., Yeo,G., Rajarethinam,J., Tan,E., Chong,C.S., Tan,C.H., Yap,G., Tan,L.K. and Ng,L.C.
EPI_ISL_17456435, EPI_ISL_17456436	Environmental Health Institute, National Environment Agency	Environmental Health Institute, National Environment Agency	Hapuarachchi,H.C., Bandara,K.B., Sumanadasa,S.D., Hapugoda,M.D., Lai,Y.L., Lee,K.S., Tan,L.K., Lin,R.T., Ng,L.F., Bucht,G., Abeyewickreme,W. and Ng,L.C.
EPI_ISL_17456437, EPI_ISL_17456438, EPI_ISL_17456439, EPI_ISL_17456440, EPI_ISL_17456441, EPI_ISL_17456442, EPI_ISL_17456443, EPI_ISL_17456444, EPI_ISL_17456445, EPI_ISL_17456446, EPI_ISL_17456447, EPI_ISL_17456448, EPI_ISL_17456449			
see above	Environmental Health Institute, National Environment Agency	Environmental Health Institute, National Environment Agency	Hapuarachchi,H.C., Wong,W.Y., Koo,C., Tien,W.P., Lu,T.Y., Yeo,G., Rajarethinam,J., Tan,E., Chong,C.S., Tan,C.H., Yap,G., Tan,L.K. and Ng,L.C.
EPI_ISL_17456450	Environmental Health Institute, National Environment Agency	Environmental Health Institute, National Environment Agency	Tien,W.P., Lim,G., Yeo,G., Chiang,S.N., Chong,C.S., Ng,L.C. and Hapuarachchi,H.C.
EPI_ISL_17456451, EPI_ISL_17456452, EPI_ISL_17456453, EPI_ISL_17456454, EPI_ISL_17456455, EPI_ISL_17456456, EPI_ISL_17456457, EPI_ISL_17456458, EPI_ISL_17456459, EPI_ISL_17456460, EPI_ISL_17456461, EPI_ISL_17456462			
see above	Environmental Health Institute, National Environment Agency	Environmental Health Institute, National Environment Agency	Hapuarachchi,H.C., Wong,W.Y., Koo,C., Tien,W.P., Lu,T.Y., Yeo,G., Rajarethinam,J., Tan,E., Chong,C.S., Tan,C.H., Yap,G., Tan,L.K. and Ng,L.C.
EPI_ISL_17456463	Environmental Health Institute, National Environment Agency	Environmental Health Institute, National Environment Agency	Hapuarachchi,H.C., Bandara,K.B., Sumanadasa,S.D., Hapugoda,M.D., Lai,Y.L., Lee,K.S., Tan,L.K., Lin,R.T., Ng,L.F., Bucht,G., Abeyewickreme,W. and Ng,L.C.
EPI_ISL_17456464, EPI_ISL_17456465, EPI_ISL_17456466, EPI_ISL_17456467, EPI_ISL_17456468, EPI_ISL_17456469, EPI_ISL_17456470, EPI_ISL_17456471, EPI_ISL_17456472, EPI_ISL_17456473, EPI_ISL_17456474, EPI_ISL_17456475, EPI_ISL_17456476, EPI_ISL_17456477, EPI_ISL_17456478, EPI_ISL_17456479, EPI_ISL_17456480, EPI_ISL_17456481, EPI_ISL_17456482, EPI_ISL_17456483, EPI_ISL_17456484, EPI_ISL_17456485, EPI_ISL_17456486, EPI_ISL_17456487, EPI_ISL_17456488, EPI_ISL_17456489, EPI_ISL_17456490, EPI_ISL_17456491, EPI_ISL_17456492, EPI_ISL_17456493, EPI_ISL_17456494, EPI_ISL_17456495, EPI_ISL_17456496, EPI_ISL_17456497, EPI_ISL_17456498, EPI_ISL_17456499, EPI_ISL_17456500, EPI_ISL_17456501, EPI_ISL_17456502, EPI_ISL_17456503, EPI_ISL_17456504			
see above	Environmental Health Institute, National Environment Agency	Environmental Health Institute, National Environment Agency	Hapuarachchi,H.C., Wong,W.Y., Koo,C., Tien,W.P., Lu,T.Y., Yeo,G., Rajarethinam,J., Tan,E., Chong,C.S., Tan,C.H., Yap,G., Tan,L.K. and Ng,L.C.
EPI_ISL_17456505	Environmental Health Institute, National Environment Agency	Environmental Health Institute, National Environment Agency	Tan,S., Yeo,G., Hapuarachchi,H.C., Wong,J., Tan,C.H. and Ng,L.C.
EPI_ISL_17456506, EPI_ISL_17456507, EPI_ISL_17456508, EPI_ISL_17456509, EPI_ISL_17456510, EPI_ISL_17456511, EPI_ISL_17456512, EPI_ISL_17456513, EPI_ISL_17456514, EPI_ISL_17456515, EPI_ISL_17456516, EPI_ISL_17456517, EPI_ISL_17456518, EPI_ISL_17456519, EPI_ISL_17456520, EPI_ISL_17456521, EPI_ISL_17456522, EPI_ISL_17456523, EPI_ISL_17456524, EPI_ISL_17456525, EPI_ISL_17456526, EPI_ISL_17456527, EPI_ISL_17456528, EPI_ISL_17456529, EPI_ISL_17456530, EPI_ISL_17456531, EPI_ISL_17456532, EPI_ISL_17456533, EPI_ISL_17456534, EPI_ISL_17456535, EPI_ISL_17456536, EPI_ISL_17456537, EPI_ISL_17456538, EPI_ISL_17456539, EPI_ISL_17456540, EPI_ISL_17456541, EPI_ISL_17456542, EPI_ISL_17456543, EPI_ISL_17456544, EPI_ISL_17456545, EPI_ISL_17456546, EPI_ISL_17456547, EPI_ISL_17456548, EPI_ISL_17456549, EPI_ISL_17456550, EPI_ISL_17456551, EPI_ISL_17456552, EPI_ISL_17456553, EPI_ISL_17456554, EPI_ISL_17456555, EPI_ISL_17456556, EPI_ISL_17456557, EPI_ISL_17456558, EPI_ISL_17456559, EPI_ISL_17456560, EPI_ISL_17456561, EPI_ISL_17456562, EPI_ISL_17456563, EPI_ISL_17456564, EPI_ISL_17456565,			

EPI_ISL_17456566, EPI_ISL_17456567, EPI_ISL_17456568, EPI_ISL_17456569, EPI_ISL_17456570, EPI_ISL_17456571, EPI_ISL_17456572, EPI_ISL_17456573			
see above	Environmental Health Institute, National Environment Agency	Environmental Health Institute, National Environment Agency	Hapuarachchi,H.C., Wong,W.Y., Koo,C., Tien,W.P., Lu,T.Y., Yeo,G., Rajarethinam,J., Tan,E., Chong,C.S., Tan,C.H., Yap,G., Tan,L.K. and Ng,L.C.
EPI_ISL_17456574, EPI_ISL_17456575, EPI_ISL_17456576, EPI_ISL_17456577, EPI_ISL_17456578	Environmental Health Institute, National Environment Agency	Environmental Health Institute, National Environment Agency	Ng,L.C., Tan,L.K., Tan,C.H., Tan,S.S., Hapuarachchi,H.C., Pok,K.Y., Lai,Y.L., Lam-Phua,S.G., Bucht,G., Lin,R.T., Leo,Y.S., Tan,B.H., Han,H.K., Ooi,P.L., James,L. and Khoo,S.P.
EPI_ISL_17456579	Environmental Health Institute, National Environment Agency	Environmental Health Institute, National Environment Agency	Hapuarachchi,H.C., Wong,W.Y., Koo,C., Tien,W.P., Lu,T.Y., Yeo,G., Rajarethinam,J., Tan,E., Chong,C.S., Tan,C.H., Yap,G., Tan,L.K. and Ng,L.C.
EPI_ISL_17456580, EPI_ISL_17456581, EPI_ISL_17456582, EPI_ISL_17456583, EPI_ISL_17456584, EPI_ISL_17456585, EPI_ISL_17456586, EPI_ISL_17456587, EPI_ISL_17456588, EPI_ISL_17456589, EPI_ISL_17456590, EPI_ISL_17456591			
see above	Environmental Health Institute, National Environment Agency	Environmental Health Institute, National Environment Agency	Ng,L.C., Tan,L.K., Tan,C.H., Tan,S.S., Hapuarachchi,H.C., Pok,K.Y., Lai,Y.L., Lam-Phua,S.G., Bucht,G., Lin,R.T., Leo,Y.S., Tan,B.H., Han,H.K., Ooi,P.L., James,L. and Khoo,S.P.
EPI_ISL_17456592	Environmental Health Institute, National Environment Agency	Environmental Health Institute, National Environment Agency	Hapuarachchi,H.C., Wong,W.Y., Koo,C., Tien,W.P., Lu,T.Y., Yeo,G., Rajarethinam,J., Tan,E., Chong,C.S., Tan,C.H., Yap,G., Tan,L.K. and Ng,L.C.
EPI_ISL_17456593	Environmental Health Institute, National Environment Agency	Environmental Health Institute, National Environment Agency	Ng,L.C., Tan,L.K., Tan,C.H., Tan,S.S., Hapuarachchi,H.C., Pok,K.Y., Lai,Y.L., Lam-Phua,S.G., Bucht,G., Lin,R.T., Leo,Y.S., Tan,B.H., Han,H.K., Ooi,P.L., James,L. and Khoo,S.P.
EPI_ISL_17456594	Environmental Health Institute, National Environment Agency	Environmental Health Institute, National Environment Agency	Hapuarachchi,H.C., Wong,W.Y., Koo,C., Tien,W.P., Lu,T.Y., Yeo,G., Rajarethinam,J., Tan,E., Chong,C.S., Tan,C.H., Yap,G., Tan,L.K. and Ng,L.C.
EPI_ISL_17456595	Environmental Health Institute, National Environment Agency	Environmental Health Institute, National Environment Agency	Ng,L.C., Tan,L.K., Tan,C.H., Tan,S.S., Hapuarachchi,H.C., Pok,K.Y., Lai,Y.L., Lam-Phua,S.G., Bucht,G., Lin,R.T., Leo,Y.S., Tan,B.H., Han,H.K., Ooi,P.L., James,L. and Khoo,S.P.
EPI_ISL_17456596	Environmental Health Institute, National Environment Agency	Environmental Health Institute, National Environment Agency	Hapuarachchi,H.C., Wong,W.Y., Koo,C., Tien,W.P., Lu,T.Y., Yeo,G., Rajarethinam,J., Tan,E., Chong,C.S., Tan,C.H., Yap,G., Tan,L.K. and Ng,L.C.
EPI_ISL_17456597, EPI_ISL_17456598	Environmental Health Institute, National Environment Agency	Environmental Health Institute, National Environment Agency	Ng,L.C., Tan,L.K., Tan,C.H., Tan,S.S., Hapuarachchi,H.C., Pok,K.Y., Lai,Y.L., Lam-Phua,S.G., Bucht,G., Lin,R.T., Leo,Y.S., Tan,B.H., Han,H.K., Ooi,P.L., James,L. and Khoo,S.P.
EPI_ISL_17456599, EPI_ISL_17456600	Environmental Health Institute, National Environment Agency	Environmental Health Institute, National Environment Agency	Hapuarachchi,H.C., Wong,W.Y., Koo,C., Tien,W.P., Lu,T.Y., Yeo,G., Rajarethinam,J., Tan,E., Chong,C.S., Tan,C.H., Yap,G., Tan,L.K. and Ng,L.C.
EPI_ISL_17456601, EPI_ISL_17456602, EPI_ISL_17456603	Environmental Health Institute, National Environment Agency	Environmental Health Institute, National Environment Agency	Ng,L.C., Tan,L.K., Tan,C.H., Tan,S.S., Hapuarachchi,H.C., Pok,K.Y., Lai,Y.L., Lam-Phua,S.G., Bucht,G., Lin,R.T., Leo,Y.S., Tan,B.H., Han,H.K., Ooi,P.L., James,L. and Khoo,S.P.
EPI_ISL_17456604	Environmental Health Institute, National Environment Agency	Environmental Health Institute, National Environment Agency	Hapuarachchi,H.C., Wong,W.Y., Koo,C., Tien,W.P., Lu,T.Y., Yeo,G., Rajarethinam,J., Tan,E., Chong,C.S., Tan,C.H., Yap,G., Tan,L.K. and Ng,L.C.
EPI_ISL_17456605, EPI_ISL_17456606, EPI_ISL_17456607	Environmental Health Institute, National Environment Agency	Environmental Health Institute, National Environment Agency	Ng,L.C., Tan,L.K., Tan,C.H., Tan,S.S., Hapuarachchi,H.C., Pok,K.Y., Lai,Y.L., Lam-Phua,S.G., Bucht,G., Lin,R.T., Leo,Y.S., Tan,B.H., Han,H.K., Ooi,P.L., James,L. and Khoo,S.P.
EPI_ISL_17456608, EPI_ISL_17456609, EPI_ISL_17456610	Environmental Health Institute, National Environment Agency	Environmental Health Institute, National Environment Agency	Hapuarachchi,H.C., Wong,W.Y., Koo,C., Tien,W.P., Lu,T.Y., Yeo,G., Rajarethinam,J., Tan,E., Chong,C.S., Tan,C.H., Yap,G., Tan,L.K. and Ng,L.C.
EPI_ISL_17456611, EPI_ISL_17456612, EPI_ISL_17456613, EPI_ISL_17456614, EPI_ISL_17456615, EPI_ISL_17456616, EPI_ISL_17456617, EPI_ISL_17456618, EPI_ISL_17456619, EPI_ISL_17456620	Environmental Health Institute, National Environment Agency	Environmental Health Institute, National Environment Agency	Ng,L.C., Tan,L.K., Tan,C.H., Tan,S.S., Hapuarachchi,H.C., Pok,K.Y., Lai,Y.L., Lam-Phua,S.G., Bucht,G., Lin,R.T., Leo,Y.S., Tan,B.H., Han,H.K., Ooi,P.L., James,L. and Khoo,S.P.
EPI_ISL_17456621, EPI_ISL_17456622, EPI_ISL_17456623, EPI_ISL_17456624, EPI_ISL_17456625, EPI_ISL_17456626	Environmental Health Institute, National Environment Agency	Environmental Health Institute, National Environment Agency	Hapuarachchi,H.C., Wong,W.Y., Koo,C., Tien,W.P., Lu,T.Y., Yeo,G., Rajarethinam,J., Tan,E., Chong,C.S., Tan,C.H., Yap,G., Tan,L.K. and Ng,L.C.
EPI_ISL_17456627	Environmental Health Institute, National Environment Agency	Environmental Health Institute, National Environment Agency	Ng,L.C., Tan,L.K., Tan,C.H., Tan,S.S., Hapuarachchi,H.C., Pok,K.Y., Lai,Y.L., Lam-Phua,S.G., Bucht,G., Lin,R.T., Leo,Y.S., Tan,B.H., Han,H.K., Ooi,P.L., James,L. and Khoo,S.P.
EPI_ISL_17456628, EPI_ISL_17456629, EPI_ISL_17456630, EPI_ISL_17456631, EPI_ISL_17456632, EPI_ISL_17456633, EPI_ISL_17456634, EPI_ISL_17456635, EPI_ISL_17456636, EPI_ISL_17456637, EPI_ISL_17456638			
see above	Environmental Health Institute, National Environment Agency	Environmental Health Institute, National Environment Agency	Hapuarachchi,H.C., Wong,W.Y., Koo,C., Tien,W.P., Lu,T.Y., Yeo,G., Rajarethinam,J., Tan,E., Chong,C.S., Tan,C.H., Yap,G., Tan,L.K. and Ng,L.C.
EPI_ISL_17456639, EPI_ISL_17456640	Environmental Health Institute, National Environment Agency	Environmental Health Institute, National Environment Agency	Ng,L.C., Tan,L.K., Tan,C.H., Tan,S.S., Hapuarachchi,H.C., Pok,K.Y., Lai,Y.L., Lam-Phua,S.G., Bucht,G., Lin,R.T., Leo,Y.S., Tan,B.H., Han,H.K., Ooi,P.L., James,L. and Khoo,S.P.
EPI_ISL_17456641	Environmental Health Institute, National Environment Agency	Environmental Health Institute, National Environment Agency	Hapuarachchi,H.C., Wong,W.Y., Koo,C., Tien,W.P., Lu,T.Y., Yeo,G., Rajarethinam,J., Tan,E., Chong,C.S., Tan,C.H., Yap,G., Tan,L.K. and Ng,L.C.
EPI_ISL_17456642, EPI_ISL_17456643, EPI_ISL_17456644	Environmental Health Institute, National Environment Agency	Environmental Health Institute, National Environment Agency	Ng,L.C., Tan,L.K., Tan,C.H., Tan,S.S., Hapuarachchi,H.C., Pok,K.Y., Lai,Y.L., Lam-Phua,S.G., Bucht,G., Lin,R.T., Leo,Y.S., Tan,B.H., Han,H.K., Ooi,P.L., James,L. and Khoo,S.P.
EPI_ISL_17456645, EPI_ISL_17456646, EPI_ISL_17456647, EPI_ISL_17456648, EPI_ISL_17456649, EPI_ISL_17456650, EPI_ISL_17456651, EPI_ISL_17456652, EPI_ISL_17456653, EPI_ISL_17456654, EPI_ISL_17456655, EPI_ISL_17456656			
see above	Environmental Health Institute, National Environment Agency	Environmental Health Institute, National Environment Agency	Hapuarachchi,H.C., Wong,W.Y., Koo,C., Tien,W.P., Lu,T.Y., Yeo,G., Rajarethinam,J., Tan,E., Chong,C.S., Tan,C.H., Yap,G., Tan,L.K. and Ng,L.C.
EPI_ISL_17456657, EPI_ISL_17456658, EPI_ISL_17456659, EPI_ISL_17456660	Environmental Health Institute, National Environment Agency	Environmental Health Institute, National Environment Agency	Ng,L.C., Tan,L.K., Tan,C.H., Tan,S.S., Hapuarachchi,H.C., Pok,K.Y., Lai,Y.L., Lam-Phua,S.G., Bucht,G., Lin,R.T., Leo,Y.S., Tan,B.H., Han,H.K., Ooi,P.L., James,L. and Khoo,S.P.
EPI_ISL_17456661, EPI_ISL_17456662, EPI_ISL_17456663, EPI_ISL_17456664, EPI_ISL_17456665	Environmental Health Institute, National Environment Agency	Environmental Health Institute, National Environment Agency	Hapuarachchi,H.C., Wong,W.Y., Koo,C., Tien,W.P., Lu,T.Y., Yeo,G., Rajarethinam,J., Tan,E., Chong,C.S., Tan,C.H., Yap,G., Tan,L.K. and Ng,L.C.
EPI_ISL_17456666	Environmental Health Institute, National Environment Agency	Environmental Health Institute, National Environment Agency	Ng,L.C., Tan,L.K., Tan,C.H., Tan,S.S., Hapuarachchi,H.C., Pok,K.Y., Lai,Y.L., Lam-Phua,S.G., Bucht,G., Lin,R.T., Leo,Y.S., Tan,B.H., Han,H.K., Ooi,P.L., James,L. and Khoo,S.P.
EPI_ISL_17456667, EPI_ISL_17456668	Environmental Health Institute, National Environment Agency	Environmental Health Institute, National Environment Agency	Hapuarachchi,H.C., Wong,W.Y., Koo,C., Tien,W.P., Lu,T.Y., Yeo,G., Rajarethinam,J., Tan,E., Chong,C.S., Tan,C.H., Yap,G., Tan,L.K. and Ng,L.C.
EPI_ISL_17456669, EPI_ISL_17456670, EPI_ISL_17456671	Environmental Health Institute, National Environment Agency	Environmental Health Institute, National Environment Agency	Ng,L.C., Tan,L.K., Tan,C.H., Tan,S.S., Hapuarachchi,H.C., Pok,K.Y., Lai,Y.L., Lam-Phua,S.G., Bucht,G., Lin,R.T., Leo,Y.S., Tan,B.H., Han,H.K., Ooi,P.L., James,L. and Khoo,S.P.
EPI_ISL_17456672	Environmental and Global Health, University of Florida	Environmental and Global Health, University of Florida	White,S.K., Subramaniam,K., Waltzek,T.B., Elbadry,M.A., Morris,J.G. Jr. and Lednicky,J.A.
EPI_ISL_17456673, EPI_ISL_17456674, EPI_ISL_17456675	Environmental and Global Health, University of Florida	Environmental and Global Health, University of Florida	White,S.K., Morris,J.G. Jr., Elbadry,M.A., Beau De Rochars,M.V. and Lednicky,J.A.
EPI_ISL_17456676	Environmental and Global Health, University of Florida	Environmental and Global Health, University of Florida	White,S.K., Elbadry,M.A., Morris,J.G. Jr. and Lednicky,J.A.
EPI_ISL_17456677, EPI_ISL_17456678, EPI_ISL_17456679	Environmental and Global Health, University of Florida	Environmental and Global Health, University of Florida	White,S.K., Morris,J.G. Jr., Elbadry,M.A., Beau De Rochars,M.V. and Lednicky,J.A.
EPI_ISL_17456680, EPI_ISL_17456681	Environmental and Global Health, University of Florida	Environmental and Global Health, University of Florida	White,S.K., Mavian,C., Salemi,M., Morris,J.G. Jr., Elbadry,M.A., Okech,B.A., Lednicky,J.A. and Dunford,J.C.
EPI_ISL_17456682	Environmental and Global Health, University of Florida	Environmental and Global Health, University of Florida	White,S.K., Morris,J.G. Jr., Elbadry,M.A., Loeb,J.C., Beau De Rochars,M.V., Okech,B.A. and Lednicky,J.A.
EPI_ISL_17456683, EPI_ISL_17456684	Environmental and Global Health, University of Florida	Environmental and Global Health, University of Florida	White,S.K., Morris,J.G. Jr., Elbadry,M.A., Beau De Rochars,M.V. and Lednicky,J.A.
EPI_ISL_17456685	Environmental and Global Health, University of Florida	Environmental and Global Health, University of Florida	Lednicky,J.A., White,S.K., Morris,J.G. Jr., Iovine,N.M., Cherabuddi,K. and Loeb,J.C.
EPI_ISL_17456686, EPI_ISL_17456687, EPI_ISL_17456688, EPI_ISL_17456689, EPI_ISL_17456690, EPI_ISL_17456691, EPI_ISL_17456692, EPI_ISL_17456693, EPI_ISL_17456694, EPI_ISL_17456695, EPI_ISL_17456696, EPI_ISL_17456697, EPI_ISL_17456698, EPI_ISL_17456699			
see above	Erasmus Medical Center	Erasmus Medical Center	Anfasa,F., Provacia,L., GeurtsvanKessel,C., Wever,R., Gerstenbluth,I., Osterhaus,A.D.M.E. and Martina,B.E.E.
EPI_ISL_17456700	Etubics Corporation	Etubics Corporation	Jones,F.R., Balint,J., Rice,A., Latchman,Y. and Gabitzsch,E.
EPI_ISL_17456701	Evandro Chagas Institute	Evandro Chagas Institute	Nunes,M.R., Faria,N.R., de Vasconcelos,J.M., Golding,N., Kraemer,M.U., de Oliveira,L.F., Azevedo Rdo,S., da Silva,D.E., da Silva,E.V., da Silva,S.P., Carvalho,V.L., Coelho,G.E., Cruz,A.C., Rodrigues,S.G., da Silva Goncalves Vianez,J.L. Jr., Nunes,B.T., Cardoso,J.F., Tesh,R.B., Hay,S.I., Pybus,O.G. and da Costa Vasconcelos,P.F.
EPI_ISL_17456702	Evandro Chagas Institute	Evandro Chagas Institute	Aragao,C.F., Pinheiro,V.C.S., Nunes Neto,J.P., Silva,E.V.P.D., Pereira,G.J.G., Nascimento,B.L.S.D., Castro,K.D.S., Maia,A.M., Catete,C.P., Martins,L.C., Tadei,W.P., Silva,S.P.D. and Cruz,A.C.R.
EPI_ISL_17456703	Evandro Chagas Institute	Evandro Chagas Institute	Nunes,M.R., Faria,N.R., de Vasconcelos,J.M., Golding,N., Kraemer,M.U., de Oliveira,L.F., Azevedo Rdo,S., da Silva,D.E., da Silva,E.V., da Silva,S.P., Carvalho,V.L., Coelho,G.E., Cruz,A.C., Rodrigues,S.G., da Silva Goncalves Vianez,J.L. Jr., Nunes,B.T., Cardoso,J.F., Tesh,R.B., Hay,S.I., Pybus,O.G. and da Costa Vasconcelos,P.F.
EPI_ISL_17456704, EPI_ISL_17456705, EPI_ISL_17456706	Evandro Chagas Institute	Evandro Chagas Institute	Ribeiro Cruz,A.C., Pinto Nunes Neto,J., Patroca da Silva,S., Vieira Pinto da Silva,E., Juscely Galvao Pereira,G., Maia Santos,M., Antonio de Oliveira Monteiro,H., Barreto Dos Santos,F., Jose de Paula Souza E Guimaraes,R., Fortes Aragao,C. and Caricio Martins,L.
EPI_ISL_17456707, EPI_ISL_17456708	Evandro Chagas Institute	Evandro Chagas Institute	Nunes,M.R., Faria,N.R., de Vasconcelos,J.M., Golding,N., Kraemer,M.U., de Oliveira,L.F., Azevedo Rdo,S., da Silva,D.E., da Silva,E.V., da Silva,S.P., Carvalho,V.L., Coelho,G.E., Cruz,A.C., Rodrigues,S.G., da Silva Goncalves Vianez,J.L. Jr.,

			Nunes,B.T., Cardoso,J.F., Tesh,R.B., Hay,S.I., Pybus,O.G. and da Costa Vasconcelos,P.F.
EPI_ISL_17456709, EPI_ISL_17456710	Evandro Chagas Institute	Evandro Chagas Institute	Ribeiro Cruz,A.C., Pinto Nunes Neto,J., Patroca da Silva,S., Vieira Pinto da Silva,E., Juscely Galvao Pereira,G., Maia Santos,M., Antonio de Oliveira Monteiro,H., Barreto Dos Santos,F., Jose de Paula Souza E Guimaraes,R., Fortes Aragao,C. and Caricio Martins,L.
EPI_ISL_17456711, EPI_ISL_17456712	Evandro Chagas Institute	Evandro Chagas Institute	Nunes,M.R., Faria,N.R., de Vasconcelos,J.M., Golding,N., Kraemer,M.U., de Oliveira,L.F., Azevedo Rdo,S., da Silva,D.E., da Silva,E.V., da Silva,S.P., Carvalho,V.L., Coelho,G.E., Cruz,A.C., Rodrigues,S.G., da Silva Goncalves Vianez,J.L. Jr., Nunes,B.T., Cardoso,J.F., Tesh,R.B., Hay,S.I., Pybus,O.G. and da Costa Vasconcelos,P.F.
EPI_ISL_17456713, EPI_ISL_17456714	Faculty of Medicine Padjadjaran University	Faculty of Medicine Padjadjaran University	Riswari,S.F., Ma'roef,C.N., Djauhari,H., Kosasih,H., Perkasa,A., Yudhaputri,F.A., Artika,I.M., Williams,M., van der Ven,A., Myint,K.S., Alisjahbana,B., Ledermann,J.P., Powers,A.M. and Jaya,U.A.
EPI_ISL_17456715, EPI_ISL_17456716, EPI_ISL_17456717	Federal University of Bahia	Federal University of Bahia	Campos,G.S., Sardi,S.I., de Paula,F.L. and Tigre,D.M.
EPI_ISL_17456718, EPI_ISL_17456719, EPI_ISL_17456720, EPI_ISL_17456721, EPI_ISL_17456722, EPI_ISL_17456723, EPI_ISL_17456724	Federal University of Bahia, Institute of Health Sciences, Department of biointeraction	Federal University of Bahia, Institute of Health Sciences, Department of biointeraction	Rodrigues,A.M., Souza,R.R.M., Sardi,S.I. and Campos,G.S.
EPI_ISL_17456725, EPI_ISL_17456726	Federal University of Espírito Santo	Federal University of Espírito Santo	Ventorim,D.P., Garcia,F.M., Vianna,L.A., Spinasse,R.D., Valli,L.P., Silva,M.O. and Louro,I.D.
EPI_ISL_17456727, EPI_ISL_17456728	Federal University of Espírito Santo	Federal University of Espírito Santo	Ventorim,D.P., Rodrigues,F.M.G.S., Vianna,L.A., Spinasse,R.D., Valli,L.C.P., Louro,I.D. and Oliveira-Silva,M.
EPI_ISL_17456729, EPI_ISL_17456730, EPI_ISL_17456731, EPI_ISL_17456732, EPI_ISL_17456733	Federal University of Espírito Santo	Federal University of Espírito Santo	Ventorim,D.P., Garcia,F.M., Vianna,L.A., Spinasse,R.D., Valli,L.P., Silva,M.O. and Louro,I.D.
EPI_ISL_17456734	Federal University of Espírito Santo	Federal University of Espírito Santo	Ventorim,D.P., Rodrigues,F.M.G.S., Vianna,L.A., Spinasse,R.D., Valli,L.C.P., Louro,I.D. and Oliveira-Silva,M.
EPI_ISL_17456735, EPI_ISL_17456736, EPI_ISL_17456737, EPI_ISL_17456738, EPI_ISL_17456739	Federal University of Espírito Santo	Federal University of Espírito Santo	Ventorim,D.P., Garcia,F.M., Vianna,L.A., Spinasse,R.D., Valli,L.P., Silva,M.O. and Louro,I.D.
EPI_ISL_17456740	Federal University of Espírito Santo	Federal University of Espírito Santo	Ventorim,D.P., Rodrigues,F.M.G.S., Vianna,L.A., Spinasse,R.D., Valli,L.C.P., Louro,I.D. and Oliveira-Silva,M.
EPI_ISL_17456741, EPI_ISL_17456742	Federal University of Mato Grosso	Federal University of Mato Grosso	de Souza Costa,M.C., Siqueira Maia,L.M., Costa de Souza,V., Gonzaga,A.M., Correa de Azevedo,V., Ramos Martins,L., Chavez Pavoni,J.H., Gomes Naveca,F. and Dezengrini Slhessarenko,R.
EPI_ISL_17456743, EPI_ISL_17456744, EPI_ISL_17456745, EPI_ISL_17456746, EPI_ISL_17456747, EPI_ISL_17456748, EPI_ISL_17456749, EPI_ISL_17456750, EPI_ISL_17456751, EPI_ISL_17456752	Federal University of Para	Federal University of Para	de Oliveira Ribeiro,G., Gill,D.E., do Socorro Foro Ramos,E., Villanova,F., Soares D'Athaide Ribeiro,E., Monteiro,F.J.C., Morais,V.S., Rego,M.Od.S., Araujo,E.L.L., Pandey,R.P., Raj,V.S., Deng,X., Delwart,E., da Costa,A.C. and Leal,E.
EPI_ISL_17456753, EPI_ISL_17456754, EPI_ISL_17456755, EPI_ISL_17456756, EPI_ISL_17456757	Federal University of Piaui	Federal University of Piaui	Cardoso,F.D., Garces,T.C.C.S., Barros,E.L.T., Rezende,I.M., Oliveira,J.G., Kroon,E.G., Oliveira,J.S., Pereira,A.C.T.C., Drumond,B.P. and Ferreira,G.P.
EPI_ISL_17456758, EPI_ISL_17456759, EPI_ISL_17456760, EPI_ISL_17456761, EPI_ISL_17456762, EPI_ISL_17456763, EPI_ISL_17456764, EPI_ISL_17456765, EPI_ISL_17456766, EPI_ISL_17456767, EPI_ISL_17456768, EPI_ISL_17456769, EPI_ISL_17456770, EPI_ISL_17456771, EPI_ISL_17456772, EPI_ISL_17456773, EPI_ISL_17456774, EPI_ISL_17456775, EPI_ISL_17456776, EPI_ISL_17456777, EPI_ISL_17456778, EPI_ISL_17456779, EPI_ISL_17456780, EPI_ISL_17456781, EPI_ISL_17456782, EPI_ISL_17456783, EPI_ISL_17456784			
see above	Federal University of Piaui, Department of Biomedical Sciences	Federal University of Piaui, Department of Biomedical Sciences	Cardoso,F.D., Rezende,I.M., Barros,E.L.T., Sacchetto,L., Garces,T.C.C.S., Silva,N.I.O., Alves,P.A., Soares,J.O., Kroon,E.G., Pereira,A.C.T.D.C., Drumond,B.P. and Ferreira,G.P.
EPI_ISL_17456785, EPI_ISL_17456786, EPI_ISL_17456787	Federal University of Rio Grande do Norte	Federal University of Rio Grande do Norte	Teixeira,D.G. and Jeronimo,S.M.B.
EPI_ISL_17456788, EPI_ISL_17456789, EPI_ISL_17456790	Federal University of Rio de Janeiro	Federal University of Rio de Janeiro	Cirne-Santos,C., Azevedo,R.C., Barros,C.S., Nogueira,C.R., Yamamoto,K.A., Meira,G.L., Vasconcelos,Z., Ratcliffe,N.A., Teixeira,V.L., Schmidt-Chanasit,J. and Ferreira,D.F.
EPI_ISL_17456791, EPI_ISL_17456792, EPI_ISL_17456793, EPI_ISL_17456794, EPI_ISL_17456795	Federal University of Roraima	Federal University of Roraima	Acosta,P.O.A., Rodrigues,A.R.R., Nascimento,I.A.S., Granja,F., Corado,A., Souza,V.C., Nascimento,V.A. and Naveca,F.G.
EPI_ISL_17456796, EPI_ISL_17456797, EPI_ISL_17456798, EPI_ISL_17456799, EPI_ISL_17456800, EPI_ISL_17456801, EPI_ISL_17456802, EPI_ISL_17456803, EPI_ISL_17456804, EPI_ISL_17456805, EPI_ISL_17456806, EPI_ISL_17456807, EPI_ISL_17456808, EPI_ISL_17456809, EPI_ISL_17456810, EPI_ISL_17456811, EPI_ISL_17456812, EPI_ISL_17456813, EPI_ISL_17456814			
see above	Federal University of Sao Paulo	Federal University of Sao Paulo	Marinho,R., Santos,L., Duro,R., Hunter,J., Teles,M., Milagres,F., Sabino,E., Diaz,R., Kawakubo,F., Khouri,R. and Komninakis,S.
EPI_ISL_17456815, EPI_ISL_17456816, EPI_ISL_17456817, EPI_ISL_17456818, EPI_ISL_17456819, EPI_ISL_17456820, EPI_ISL_17456821, EPI_ISL_17456822, EPI_ISL_17456823, EPI_ISL_17456824, EPI_ISL_17456825, EPI_ISL_17456826, EPI_ISL_17456827, EPI_ISL_17456828, EPI_ISL_17456829, EPI_ISL_17456830			
see above	Federal University of Sergipe	Federal University of Sergipe	Jesus,M.C.S., Chagas,R.D.O., Santos,C.A., Santos,R.L.C., Batista,M.V.A. and Storti-Melo,L.M.
EPI_ISL_17456831, EPI_ISL_17456832, EPI_ISL_17456833, EPI_ISL_17456834, EPI_ISL_17456835, EPI_ISL_17456836, EPI_ISL_17456837, EPI_ISL_17456838, EPI_ISL_17456839, EPI_ISL_17456840, EPI_ISL_17456841, EPI_ISL_17456842, EPI_ISL_17456843, EPI_ISL_17456844, EPI_ISL_17456845, EPI_ISL_17456846, EPI_ISL_17456847, EPI_ISL_17456848, EPI_ISL_17456849, EPI_ISL_17456850, EPI_ISL_17456851, EPI_ISL_17456852, EPI_ISL_17456853, EPI_ISL_17456854, EPI_ISL_17456855, EPI_ISL_17456856, EPI_ISL_17456857			
see above	Federal University of Tocantins	Federal University of Tocantins	Souza,U.J.B., Santos,R.N.D., Giovanetti,M., Alcantara,L.C.J., Galvao,J.D., Cardoso,F.D.P., Brito,F.C.S., Franco,A.C., Roehe,P.M., Ribeiro,B.M., Spilki,F.R. and Campos,F.S.
EPI_ISL_17456858	French Military Biomedical Research Institute (IRBA)	French Military Biomedical Research Institute (IRBA)	Fourie,T., Dia,A., Piorkowski,G., Durand,G., Grard,G., de Lamballerie,X., Simon,F. and Leparc-Goffart,I.
EPI_ISL_17456859	Fundacao Oswaldo Cruz	Fundacao Oswaldo Cruz	Giovanetti,M., Fonseca,V., Trinta,K., Faria,N., Theze,J., Filippis,A.M.B., Alcantara,L.C.J. and Cunha,R.V.
EPI_ISL_17456860	Fundacao Oswaldo Cruz	Fundacao Oswaldo Cruz	Fritsch,H., Iani,F., Adelino,T., Fonseca,V., Giovanetti,M. and Alcantara,L.C.J.
EPI_ISL_17456861, EPI_ISL_17456862, EPI_ISL_17456863, EPI_ISL_17456864	Fundacao Oswaldo Cruz	Fundacao Oswaldo Cruz	Giovanetti,M., Fonseca,V.S., Lourenco,J., Xavier,J., Claro,I.M., Graf,T., Nascimento,V.A., Adelino,T., Iani,F.C.M., Sabino,E.C., Naveca,F.G., Salles,F.S., Nogueira,L., Fabri,A., Oliveira,E.C., Demarchi,L.H.F., Silva,V.L., Filippis,A.M.B., Abreu,A.L., Oliveira,W.K., Croda,J., Albuquerque,C.F.C., Loman,N., Faria,N.R., de Oliveira,T. and Alcantara,L.C.J.
EPI_ISL_17456865, EPI_ISL_17456866	Fundacao Oswaldo Cruz	Fundacao Oswaldo Cruz	Fritsch,H., Iani,F., Adelino,T., Fonseca,V., Giovanetti,M. and Alcantara,L.C.J.
EPI_ISL_17456867, EPI_ISL_17456868, EPI_ISL_17456869, EPI_ISL_17456870	Fundacao Oswaldo Cruz	Fundacao Oswaldo Cruz	Giovanetti,M., Fonseca,V.S., Lourenco,J., Xavier,J., Claro,I.M., Graf,T., Nascimento,V.A., Adelino,T., Iani,F.C.M., Sabino,E.C., Naveca,F.G., Salles,F.S., Nogueira,L., Fabri,A., Oliveira,E.C., Demarchi,L.H.F., Silva,V.L., Filippis,A.M.B., Abreu,A.L., Oliveira,W.K., Croda,J., Albuquerque,C.F.C., Loman,N., Faria,N.R., de Oliveira,T. and Alcantara,L.C.J.
EPI_ISL_17456871	Fundacao Oswaldo Cruz	Fundacao Oswaldo Cruz	Giovanetti,M., Shi,C., Fonseca,V.S., Matthijnssens,J., Alcantara,L.C.J., Vandamme,A.-M. and Cuypers,L.
EPI_ISL_17456872	Fundacao Oswaldo Cruz	Fundacao Oswaldo Cruz	Giovanetti,M., Fonseca,V.S., Lourenco,J., Xavier,J., Claro,I.M., Graf,T., Nascimento,V.A., Adelino,T., Iani,F.C.M., Sabino,E.C., Naveca,F.G., Salles,F.S., Nogueira,L., Fabri,A., Oliveira,E.C., Demarchi,L.H.F., Silva,V.L., Filippis,A.M.B., Abreu,A.L., Oliveira,W.K., Croda,J., Albuquerque,C.F.C., Loman,N., Faria,N.R., de Oliveira,T. and Alcantara,L.C.J.
EPI_ISL_17456873	Fundacao Oswaldo Cruz	Fundacao Oswaldo Cruz	Giovanetti,M., Fonseca,V. and Alcantara,L.C.J.
EPI_ISL_17456874	Fundacao Oswaldo Cruz	Fundacao Oswaldo Cruz	Fritsch,H., Iani,F., Adelino,T., Fonseca,V., Giovanetti,M. and Alcantara,L.C.J.
EPI_ISL_17456875, EPI_ISL_17456876, EPI_ISL_17456877, EPI_ISL_17456878	Fundacao Oswaldo Cruz	Fundacao Oswaldo Cruz	Giovanetti,M., Fonseca,V.S., Lourenco,J., Xavier,J., Claro,I.M., Graf,T., Nascimento,V.A., Adelino,T., Iani,F.C.M., Sabino,E.C., Naveca,F.G., Salles,F.S., Nogueira,L., Fabri,A., Oliveira,E.C., Demarchi,L.H.F., Silva,V.L., Filippis,A.M.B., Abreu,A.L., Oliveira,W.K., Croda,J., Albuquerque,C.F.C., Loman,N., Faria,N.R., de Oliveira,T. and Alcantara,L.C.J.
EPI_ISL_17456879, EPI_ISL_17456880, EPI_ISL_17456881	Fundacao Oswaldo Cruz	Fundacao Oswaldo Cruz	Xavier,J., Giovanetti,M., Fonseca,V., Theze,J., Mendonca,M., Mares-Guia,M.A., Goes de Jesus,J., Fabri,A., Faria,N.R., Alcantara,L.C.J. and Filippis,A.M.B.
EPI_ISL_17456882	Fundacao Oswaldo Cruz	Fundacao Oswaldo Cruz	Giovanetti,M., Fonseca,V. and Alcantara,L.C.J.
EPI_ISL_17456883, EPI_ISL_17456884, EPI_ISL_17456885	Fundacao Oswaldo Cruz	Fundacao Oswaldo Cruz	Fritsch,H., Iani,F., Adelino,T., Fonseca,V., Giovanetti,M. and Alcantara,L.C.J.
EPI_ISL_17456886	Fundacao Oswaldo Cruz	Fundacao Oswaldo Cruz	Giovanetti,M., Fonseca,V., Trinta,K., Faria,N., Theze,J., Filippis,A.M.B., Alcantara,L.C.J. and Cunha,R.V.
EPI_ISL_17456887, EPI_ISL_17456888	Fundacao Oswaldo Cruz	Fundacao Oswaldo Cruz	Fritsch,H., Iani,F., Adelino,T., Fonseca,V., Giovanetti,M. and Alcantara,L.C.J.
EPI_ISL_17456889	Fundacao Oswaldo Cruz	Fundacao Oswaldo Cruz	Giovanetti,M., Fonseca,V., Trinta,K., Faria,N., Theze,J., Filippis,A.M.B., Alcantara,L.C.J. and Cunha,R.V.
EPI_ISL_17456890	Fundacao Oswaldo Cruz	Fundacao Oswaldo Cruz	Giovanetti,M., Fonseca,V. and Alcantara,L.C.J.
EPI_ISL_17456891, EPI_ISL_17456892, EPI_ISL_17456893, EPI_ISL_17456894	Fundacao Oswaldo Cruz	Fundacao Oswaldo Cruz	Giovanetti,M., Fonseca,V.S., Lourenco,J., Xavier,J., Claro,I.M., Graf,T., Nascimento,V.A., Adelino,T., Iani,F.C.M., Sabino,E.C., Naveca,F.G., Salles,F.S., Nogueira,L., Fabri,A., Oliveira,E.C., Demarchi,L.H.F., Silva,V.L., Filippis,A.M.B., Abreu,A.L., Oliveira,W.K., Croda,J., Albuquerque,C.F.C., Loman,N., Faria,N.R., de Oliveira,T. and Alcantara,L.C.J.
EPI_ISL_17456895	Fundacao Oswaldo Cruz	Fundacao Oswaldo Cruz	Giovanetti,M., Fonseca,V., Trinta,K., Faria,N., Theze,J., Filippis,A.M.B., Alcantara,L.C.J. and Cunha,R.V.
EPI_ISL_17456896	Fundacao Oswaldo Cruz	Fundacao Oswaldo Cruz	Giovanetti,M., Fonseca,V.S., Lourenco,J., Xavier,J., Claro,I.M., Graf,T., Nascimento,V.A., Adelino,T., Iani,F.C.M., Sabino,E.C., Naveca,F.G., Salles,F.S., Nogueira,L., Fabri,A., Oliveira,E.C., Demarchi,L.H.F., Silva,V.L., Filippis,A.M.B., Abreu,A.L., Oliveira,W.K., Croda,J., Albuquerque,C.F.C., Loman,N., Faria,N.R., de Oliveira,T. and Alcantara,L.C.J.
EPI_ISL_17456897	Fundacao Oswaldo Cruz	Fundacao Oswaldo Cruz	Graf,T.C., Vazquez,C., Giovanetti,M., Fonseca,V.S., Bruycker,F.N., Gomez,A., Xavier,J., Mendonca,M.C.L., Jesus,J.G., Claro,I.M., Faria,N.R., de Oliveira,T., Filippis,A.M.B., Abreu,A.L., Oliveira,W.K., Croda,J., Albuquerque,C.F.C., Rico,J.M.A. and Alcantara,L.C.J.
EPI_ISL_17456898	Fundacao Oswaldo Cruz	Fundacao Oswaldo Cruz	Giovanetti,M., Fonseca,V.S., Lourenco,J., Xavier,J., Claro,I.M., Graf,T., Nascimento,V.A., Adelino,T., Iani,F.C.M., Sabino,E.C., Naveca,F.G., Salles,F.S., Nogueira,L., Fabri,A., Oliveira,E.C., Demarchi,L.H.F., Silva,V.L., Filippis,A.M.B., Abreu,A.L., Oliveira,W.K., Croda,J., Albuquerque,C.F.C., Loman,N., Faria,N.R., de Oliveira,T. and Alcantara,L.C.J.
EPI_ISL_17456899	Fundacao Oswaldo Cruz	Fundacao Oswaldo Cruz	Xavier,J., Giovanetti,M., Fonseca,V., Theze,J., Mendonca,M., Mares-Guia,M.A., Goes de Jesus,J., Fabri,A., Faria,N.R., Alcantara,L.C.J. and Filippis,A.M.B.
EPI_ISL_17456900, EPI_ISL_17456901, EPI_ISL_17456902, EPI_ISL_17456903	Fundacao Oswaldo Cruz	Fundacao Oswaldo Cruz	Giovanetti,M., Fonseca,V. and Alcantara,L.C.J.
EPI_ISL_17456904	Fundacao Oswaldo Cruz	Fundacao Oswaldo Cruz	Giovanetti,M., Fonseca,V., Trinta,K., Faria,N., Theze,J., Filippis,A.M.B., Alcantara,L.C.J. and Cunha,R.V.
EPI_ISL_17456905	Fundacao Oswaldo Cruz	Fundacao Oswaldo Cruz	Xavier,J., Giovanetti,M., Fonseca,V., Theze,J., Mendonca,M., Mares-Guia,M.A., Goes de Jesus,J., Fabri,A., Faria,N.R., Alcantara,L.C.J. and Filippis,A.M.B.
EPI_ISL_17456906	Fundacao Oswaldo Cruz	Fundacao Oswaldo Cruz	Fritsch,H., Iani,F., Adelino,T., Fonseca,V., Giovanetti,M. and Alcantara,L.C.J.
EPI_ISL_17456907	Fundacao Oswaldo Cruz	Fundacao Oswaldo Cruz	Giovanetti,M., Fonseca,V. and Alcantara,L.C.J.
EPI_ISL_17456908	Fundacao Oswaldo Cruz	Fundacao Oswaldo Cruz	Xavier,J., Giovanetti,M., Fonseca,V., Theze,J., Mendonca,M., Mares-Guia,M.A., Goes de Jesus,J., Fabri,A., Faria,N.R., Alcantara,L.C.J. and Filippis,A.M.B.
EPI_ISL_17456909, EPI_ISL_17456910, EPI_ISL_17456911, EPI_ISL_17456912	Fundacao Oswaldo Cruz	Fundacao Oswaldo Cruz	Giovanetti,M., Fonseca,V. and Alcantara,L.C.J.
EPI_ISL_17456913, EPI_ISL_17456914, EPI_ISL_17456915	Fundacao Oswaldo Cruz	Fundacao Oswaldo Cruz	Giovanetti,M., Fonseca,V.S., Lourenco,J., Xavier,J., Claro,I.M., Graf,T., Nascimento,V.A., Adelino,T., Iani,F.C.M., Sabino,E.C., Naveca,F.G., Salles,F.S., Nogueira,L., Fabri,A., Oliveira,E.C., Demarchi,L.H.F., Silva,V.L., Filippis,A.M.B., Abreu,A.L., Oliveira,W.K., Croda,J., Albuquerque,C.F.C., Loman,N., Faria,N.R., de Oliveira,T. and Alcantara,L.C.J.
EPI_ISL_17456916, EPI_ISL_17456917, EPI_ISL_17456918,	Fundacao Oswaldo Cruz	Fundacao Oswaldo Cruz	Graf,T.C., Vazquez,C., Giovanetti,M., Fonseca,V.S., Bruycker,F.N., Gomez,A., Xavier,J., Mendonca,M.C.L., Jesus,J.G., Claro,I.M., Faria,N.R., de Oliveira,T., Filippis,A.M.B., Abreu,A.L., Oliveira,W.K., Croda,J., Albuquerque,C.F.C., Rico,J.M.A. and

EPI_ISL_17456919			Alcantara,L.C.J.
EPI_ISL_17456920, EPI_ISL_17456921, EPI_ISL_17456922, EPI_ISL_17456923, EPI_ISL_17456924, EPI_ISL_17456925, EPI_ISL_17456926	Fundacao Oswaldo Cruz	Fundacao Oswaldo Cruz	Xavier,J., Bezerra,J.F.B., Alves,M.M., Fonseca,V., Mares-Guia,M.A., Morales,I., Adelino,T., Fabri,A.A., Souza,T.R., Oliveira,E.C., Siqueira,P., Said,R.F.C., Albuquerque,C.F.C., Filippis,A.M.B., Cunha,R.V., Luz,K.G., Giovanetti,M. and Alcantara,L.C.J.
EPI_ISL_17456927	Fundacao Oswaldo Cruz	Fundacao Oswaldo Cruz	Graf,T.C., Vazquez,C., Giovanetti,M., Fonseca,V.S., Bruycker,F.N., Gomez,A., Xavier,J., Mendonca,M.C.L., Jesus,J.G., Claro,I.M., Faria,N.R., de Oliveira,T., Filippis,A.M.B., Abreu,A.L., Oliveira,W.K., Croda,J., Albuquerque,C.F.C., Rico,J.M.A. and Alcantara,L.C.J.
EPI_ISL_17456928	Fundacao Oswaldo Cruz	Fundacao Oswaldo Cruz	Fritsch,H., Iani,F., Adelino,T., Fonseca,V., Giovanetti,M. and Alcantara,L.C.J.
EPI_ISL_17456929	Fundacao Oswaldo Cruz	Fundacao Oswaldo Cruz	Giovanetti,M., Shi,C., Fonseca,V.S., Matthijnssens,J., Alcantara,L.C.J., Vandamme,A.-M. and Cuypers,L.
EPI_ISL_17456930	Fundacao Oswaldo Cruz	Fundacao Oswaldo Cruz	Giovanetti,M., Fonseca,V.S., Lourenco,J., Xavier,J., Claro,I.M., Graf,T., Nascimento,V.A., Adelino,T., Iani,F.C.M., Sabino,E.C., Naveca,F.G., Salles,F.S., Nogueira,L., Fabri,A., Oliveira,E.C., Demarchi,L.H.F., Silva,V.L., Filippis,A.M.B., Abreu,A.L., Oliveira,W.K., Croda,J., Albuquerque,C.F.C., Loman,N., Faria,N.R., de Oliveira,T. and Alcantara,L.C.J.
EPI_ISL_17456931	Fundacao Oswaldo Cruz	Fundacao Oswaldo Cruz	Graf,T.C., Vazquez,C., Giovanetti,M., Fonseca,V.S., Bruycker,F.N., Gomez,A., Xavier,J., Mendonca,M.C.L., Jesus,J.G., Claro,I.M., Faria,N.R., de Oliveira,T., Filippis,A.M.B., Abreu,A.L., Oliveira,W.K., Croda,J., Albuquerque,C.F.C., Rico,J.M.A. and Alcantara,L.C.J.
EPI_ISL_17456932, EPI_ISL_17456933	Fundacao Oswaldo Cruz	Fundacao Oswaldo Cruz	Giovanetti,M., Fonseca,V., Trinta,K., Faria,N., Theze,J., Filippis,A.M.B., Alcantara,L.C.J. and Cunha,R.V.
EPI_ISL_17456934, EPI_ISL_17456935, EPI_ISL_17456936, EPI_ISL_17456937	Fundacao Oswaldo Cruz	Fundacao Oswaldo Cruz	Graf,T.C., Vazquez,C., Giovanetti,M., Fonseca,V.S., Bruycker,F.N., Gomez,A., Xavier,J., Mendonca,M.C.L., Jesus,J.G., Claro,I.M., Faria,N.R., de Oliveira,T., Filippis,A.M.B., Abreu,A.L., Oliveira,W.K., Croda,J., Albuquerque,C.F.C., Rico,J.M.A. and Alcantara,L.C.J.
EPI_ISL_17456938	Fundacao Oswaldo Cruz	Fundacao Oswaldo Cruz	Giovanetti,M., Fonseca,V.S., Lourenco,J., Xavier,J., Claro,I.M., Graf,T., Nascimento,V.A., Adelino,T., Iani,F.C.M., Sabino,E.C., Naveca,F.G., Salles,F.S., Nogueira,L., Fabri,A., Oliveira,E.C., Demarchi,L.H.F., Silva,V.L., Filippis,A.M.B., Abreu,A.L., Oliveira,W.K., Croda,J., Albuquerque,C.F.C., Loman,N., Faria,N.R., de Oliveira,T. and Alcantara,L.C.J.
EPI_ISL_17456939	Fundacao Oswaldo Cruz	Fundacao Oswaldo Cruz	Xavier,J., Bezerra,J.F.B., Alves,M.M., Fonseca,V., Mares-Guia,M.A., Morales,I., Adelino,T., Fabri,A.A., Souza,T.R., Oliveira,E.C., Siqueira,P., Said,R.F.C., Albuquerque,C.F.C., Filippis,A.M.B., Cunha,R.V., Luz,K.G., Giovanetti,M. and Alcantara,L.C.J.
EPI_ISL_17456940	Fundacao Oswaldo Cruz	Fundacao Oswaldo Cruz	Fritsch,H., Iani,F., Adelino,T., Fonseca,V., Giovanetti,M. and Alcantara,L.C.J.
EPI_ISL_17456941	Fundacao Oswaldo Cruz	Fundacao Oswaldo Cruz	Graf,T.C., Vazquez,C., Giovanetti,M., Fonseca,V.S., Bruycker,F.N., Gomez,A., Xavier,J., Mendonca,M.C.L., Jesus,J.G., Claro,I.M., Faria,N.R., de Oliveira,T., Filippis,A.M.B., Abreu,A.L., Oliveira,W.K., Croda,J., Albuquerque,C.F.C., Rico,J.M.A. and Alcantara,L.C.J.
EPI_ISL_17456942	Fundacao Oswaldo Cruz	Fundacao Oswaldo Cruz	Pereira Gusmao Maia,Z., Mota Pereira,F., do Carmo Said,R.F., Fonseca,V., Graf,T., de Bruycker Nogueira,F., Brandao Nardy,V., Xavier,J., Lima Maia,M., Abreu,A.L., Campelo de Albuquerque,C.F., Kleber Oliveira,W., Croda,J., de Filippis,A.M.B., Venancio Cunha,R., Lourenco,J., de Oliveira,T., Faria,N.R., Junior Alcantara,L.C. and Giovanetti,M.
EPI_ISL_17456943, EPI_ISL_17456944, EPI_ISL_17456945, EPI_ISL_17456946, EPI_ISL_17456947, EPI_ISL_17456948	Fundacao Oswaldo Cruz	Fundacao Oswaldo Cruz	Fritsch,H., Iani,F., Adelino,T., Fonseca,V., Giovanetti,M. and Alcantara,L.C.J.
EPI_ISL_17456949	Fundacao Oswaldo Cruz	Fundacao Oswaldo Cruz	Pereira Gusmao Maia,Z., Mota Pereira,F., do Carmo Said,R.F., Fonseca,V., Graf,T., de Bruycker Nogueira,F., Brandao Nardy,V., Xavier,J., Lima Maia,M., Abreu,A.L., Campelo de Albuquerque,C.F., Kleber Oliveira,W., Croda,J., de Filippis,A.M.B., Venancio Cunha,R., Lourenco,J., de Oliveira,T., Faria,N.R., Junior Alcantara,L.C. and Giovanetti,M.
EPI_ISL_17456950, EPI_ISL_17456951	Fundacao Oswaldo Cruz	Fundacao Oswaldo Cruz	Graf,T.C., Vazquez,C., Giovanetti,M., Fonseca,V.S., Bruycker,F.N., Gomez,A., Xavier,J., Mendonca,M.C.L., Jesus,J.G., Claro,I.M., Faria,N.R., de Oliveira,T., Filippis,A.M.B., Abreu,A.L., Oliveira,W.K., Croda,J., Albuquerque,C.F.C., Rico,J.M.A. and Alcantara,L.C.J.
EPI_ISL_17456952, EPI_ISL_17456953, EPI_ISL_17456954	Fundacao Oswaldo Cruz	Fundacao Oswaldo Cruz	Fritsch,H., Iani,F., Adelino,T., Fonseca,V., Giovanetti,M. and Alcantara,L.C.J.
EPI_ISL_17456955	Fundacao Oswaldo Cruz	Fundacao Oswaldo Cruz	Pereira Gusmao Maia,Z., Mota Pereira,F., do Carmo Said,R.F., Fonseca,V., Graf,T., de Bruycker Nogueira,F., Brandao Nardy,V., Xavier,J., Lima Maia,M., Abreu,A.L., Campelo de Albuquerque,C.F., Kleber Oliveira,W., Croda,J., de Filippis,A.M.B., Venancio Cunha,R., Lourenco,J., de Oliveira,T., Faria,N.R., Junior Alcantara,L.C. and Giovanetti,M.
EPI_ISL_17456956, EPI_ISL_17456957, EPI_ISL_17456958	Fundacao Oswaldo Cruz	Fundacao Oswaldo Cruz	Fritsch,H., Iani,F., Adelino,T., Fonseca,V., Giovanetti,M. and Alcantara,L.C.J.
EPI_ISL_17456959, EPI_ISL_17456960	Fundacao Oswaldo Cruz	Fundacao Oswaldo Cruz	de Souza,T.M.A., Azeredo,E.L., Badolato,J.C., Damasco,P.V., Santos,C.C., Petitinga Paiva,F., Nunes,P.C.G., Barbosa,L.S., Cipitelli,M.C., Chouin-Caneiro,T., Faria,N.R.C., Nogueira,R.M.R., de Bruycker-Nogueira,F. and dos Santos,F.B.
EPI_ISL_17456961, EPI_ISL_17456962, EPI_ISL_17456963, EPI_ISL_17456964, EPI_ISL_17456965	Fundacao Oswaldo Cruz	Fundacao Oswaldo Cruz	Fritsch,H., Iani,F., Adelino,T., Fonseca,V., Giovanetti,M. and Alcantara,L.C.J.
EPI_ISL_17456966	Fundacao Oswaldo Cruz	Fundacao Oswaldo Cruz	Pereira Gusmao Maia,Z., Mota Pereira,F., do Carmo Said,R.F., Fonseca,V., Graf,T., de Bruycker Nogueira,F., Brandao Nardy,V., Xavier,J., Lima Maia,M., Abreu,A.L., Campelo de Albuquerque,C.F., Kleber Oliveira,W., Croda,J., de Filippis,A.M.B., Venancio Cunha,R., Lourenco,J., de Oliveira,T., Faria,N.R., Junior Alcantara,L.C. and Giovanetti,M.
EPI_ISL_17456967, EPI_ISL_17456968, EPI_ISL_17456969, EPI_ISL_17456970, EPI_ISL_17456971	Fundacao Oswaldo Cruz	Fundacao Oswaldo Cruz	Fritsch,H., Iani,F., Adelino,T., Fonseca,V., Giovanetti,M. and Alcantara,L.C.J.
EPI_ISL_17456972	Fundacao Oswaldo Cruz	Fundacao Oswaldo Cruz	Graf,T.C., Vazquez,C., Giovanetti,M., Fonseca,V.S., Bruycker,F.N., Gomez,A., Xavier,J., Mendonca,M.C.L., Jesus,J.G., Claro,I.M., Faria,N.R., de Oliveira,T., Filippis,A.M.B., Abreu,A.L., Oliveira,W.K., Croda,J., Albuquerque,C.F.C., Rico,J.M.A. and Alcantara,L.C.J.
EPI_ISL_17456973, EPI_ISL_17456974	Fundacao Oswaldo Cruz	Fundacao Oswaldo Cruz	Xavier,J., Giovanetti,M., Fonseca,V., Theze,J., Mendonca,M., Mares-Guia,M.A., Goes de Jesus,J., Fabri,A., Faria,N.R., Alcantara,L.C.J. and Filippis,A.M.B.
EPI_ISL_17456975	Fundacao Oswaldo Cruz	Fundacao Oswaldo Cruz	Fritsch,H., Iani,F., Adelino,T., Fonseca,V., Giovanetti,M. and Alcantara,L.C.J.
EPI_ISL_17456976	Fundacao Oswaldo Cruz	Fundacao Oswaldo Cruz	Giovanetti,M., Fonseca,V., Trinta,K., Faria,N., Theze,J., Filippis,A.M.B., Alcantara,L.C.J. and Cunha,R.V.
EPI_ISL_17456977	Fundacao Oswaldo Cruz	Fundacao Oswaldo Cruz	Xavier,J., Giovanetti,M., Fonseca,V., Theze,J., Mendonca,M., Mares-Guia,M.A., Goes de Jesus,J., Fabri,A., Faria,N.R., Alcantara,L.C.J. and Filippis,A.M.B.
EPI_ISL_17456978	Fundacao Oswaldo Cruz	Fundacao Oswaldo Cruz	Pereira Gusmao Maia,Z., Mota Pereira,F., do Carmo Said,R.F., Fonseca,V., Graf,T., de Bruycker Nogueira,F., Brandao Nardy,V., Xavier,J., Lima Maia,M., Abreu,A.L., Campelo de Albuquerque,C.F., Kleber Oliveira,W., Croda,J., de Filippis,A.M.B., Venancio Cunha,R., Lourenco,J., de Oliveira,T., Faria,N.R., Junior Alcantara,L.C. and Giovanetti,M.
EPI_ISL_17456979	Fundacao Oswaldo Cruz	Fundacao Oswaldo Cruz	Xavier,J., Giovanetti,M., Fonseca,V., Theze,J., Mendonca,M., Mares-Guia,M.A., Goes de Jesus,J., Fabri,A., Faria,N.R., Alcantara,L.C.J. and Filippis,A.M.B.
EPI_ISL_17456980	Fundacao Oswaldo Cruz	Fundacao Oswaldo Cruz	Giovanetti,M., Fonseca,V., Trinta,K., Faria,N., Theze,J., Filippis,A.M.B., Alcantara,L.C.J. and Cunha,R.V.
EPI_ISL_17456981	Fundacao Oswaldo Cruz	Fundacao Oswaldo Cruz	Xavier,J., Giovanetti,M., Fonseca,V., Theze,J., Mendonca,M., Mares-Guia,M.A., Goes de Jesus,J., Fabri,A., Faria,N.R., Alcantara,L.C.J. and Filippis,A.M.B.
EPI_ISL_17456982, EPI_ISL_17456983, EPI_ISL_17456984, EPI_ISL_17456985, EPI_ISL_17456986	Fundacao Oswaldo Cruz	Fundacao Oswaldo Cruz	Giovanetti,M., Fonseca,V., Trinta,K., Faria,N., Theze,J., Filippis,A.M.B., Alcantara,L.C.J. and Cunha,R.V.
EPI_ISL_17456987	Fundacao Oswaldo Cruz	Fundacao Oswaldo Cruz	Graf,T.C., Vazquez,C., Giovanetti,M., Fonseca,V.S., Bruycker,F.N., Gomez,A., Xavier,J., Mendonca,M.C.L., Jesus,J.G., Claro,I.M., Faria,N.R., de Oliveira,T., Filippis,A.M.B., Abreu,A.L., Oliveira,W.K., Croda,J., Albuquerque,C.F.C., Rico,J.M.A. and Alcantara,L.C.J.
EPI_ISL_17456988	Fundacao Oswaldo Cruz	Fundacao Oswaldo Cruz	de Souza,T.M.A., Azeredo,E.L., Badolato,J.C., Damasco,P.V., Santos,C.C., Petitinga Paiva,F., Nunes,P.C.G., Barbosa,L.S., Cipitelli,M.C., Chouin-Caneiro,T., Faria,N.R.C., Nogueira,R.M.R., de Bruycker-Nogueira,F. and dos Santos,F.B.
EPI_ISL_17456989, EPI_ISL_17456990, EPI_ISL_17456991	Fundacao Oswaldo Cruz	Fundacao Oswaldo Cruz	Fritsch,H., Iani,F., Adelino,T., Fonseca,V., Giovanetti,M. and Alcantara,L.C.J.
EPI_ISL_17456992, EPI_ISL_17456993, EPI_ISL_17456994	Fundacao Oswaldo Cruz	Fundacao Oswaldo Cruz	Pereira Gusmao Maia,Z., Mota Pereira,F., do Carmo Said,R.F., Fonseca,V., Graf,T., de Bruycker Nogueira,F., Brandao Nardy,V., Xavier,J., Lima Maia,M., Abreu,A.L., Campelo de Albuquerque,C.F., Kleber Oliveira,W., Croda,J., de Filippis,A.M.B., Venancio Cunha,R., Lourenco,J., de Oliveira,T., Faria,N.R., Junior Alcantara,L.C. and Giovanetti,M.
EPI_ISL_17456995	Fundacao Oswaldo Cruz	Fundacao Oswaldo Cruz	de Souza,T.M.A., Azeredo,E.L., Badolato,J.C., Damasco,P.V., Santos,C.C., Petitinga Paiva,F., Nunes,P.C.G., Barbosa,L.S., Cipitelli,M.C., Chouin-Caneiro,T., Faria,N.R.C., Nogueira,R.M.R., de Bruycker-Nogueira,F. and dos Santos,F.B.
EPI_ISL_17456996, EPI_ISL_17456997	Fundacao Oswaldo Cruz	Fundacao Oswaldo Cruz	Fritsch,H., Iani,F., Adelino,T., Fonseca,V., Giovanetti,M. and Alcantara,L.C.J.
EPI_ISL_17456998	Fundacao Oswaldo Cruz	Fundacao Oswaldo Cruz	de Souza,T.M.A., Azeredo,E.L., Badolato,J.C., Damasco,P.V., Santos,C.C., Petitinga Paiva,F., Nunes,P.C.G., Barbosa,L.S., Cipitelli,M.C., Chouin-Caneiro,T., Faria,N.R.C., Nogueira,R.M.R., de Bruycker-Nogueira,F. and dos Santos,F.B.
EPI_ISL_17456999	Fundacao Oswaldo Cruz	Fundacao Oswaldo Cruz	Graf,T.C., Vazquez,C., Giovanetti,M., Fonseca,V.S., Bruycker,F.N., Gomez,A., Xavier,J., Mendonca,M.C.L., Jesus,J.G., Claro,I.M., Faria,N.R., de Oliveira,T., Filippis,A.M.B., Abreu,A.L., Oliveira,W.K., Croda,J., Albuquerque,C.F.C., Rico,J.M.A. and Alcantara,L.C.J.
EPI_ISL_17457000, EPI_ISL_17457001, EPI_ISL_17457002, EPI_ISL_17457003, EPI_ISL_17457004	Fundacao Oswaldo Cruz	Fundacao Oswaldo Cruz	de Souza,T.M.A., Azeredo,E.L., Badolato,J.C., Damasco,P.V., Santos,C.C., Petitinga Paiva,F., Nunes,P.C.G., Barbosa,L.S., Cipitelli,M.C., Chouin-Caneiro,T., Faria,N.R.C., Nogueira,R.M.R., de Bruycker-Nogueira,F. and dos Santos,F.B.
EPI_ISL_17457005	Fundacao Oswaldo Cruz	Fundacao Oswaldo Cruz	Pereira Gusmao Maia,Z., Mota Pereira,F., do Carmo Said,R.F., Fonseca,V., Graf,T., de Bruycker Nogueira,F., Brandao Nardy,V., Xavier,J., Lima Maia,M., Abreu,A.L., Campelo de Albuquerque,C.F., Kleber Oliveira,W., Croda,J., de Filippis,A.M.B., Venancio Cunha,R., Lourenco,J., de Oliveira,T., Faria,N.R., Junior Alcantara,L.C. and Giovanetti,M.
EPI_ISL_17457006	Gen-Probe	Gen-Probe	Linnen,J.M. and Carrick,J.M.
EPI_ISL_17457007, EPI_ISL_17457008	Gen-Probe	Gen-Probe	CARRICK,J. and LINNEN,J.
EPI_ISL_17457009, EPI_ISL_17457010, EPI_ISL_17457011, EPI_ISL_17457012, EPI_ISL_17457013	Gen-Probe	Gen-Probe	Linnen,J.M. and Carrick,J.M.
EPI_ISL_17457014, EPI_ISL_17457015, EPI_ISL_17457016, EPI_ISL_17457017, EPI_ISL_17457018, EPI_ISL_17457019, EPI_ISL_17457020, EPI_ISL_17457021	Gen-Probe	Gen-Probe	CARRICK,J. and LINNEN,J.
EPI_ISL_17457022	Gen-Probe	Gen-Probe	Linnen,J.M. and Carrick,J.M.
EPI_ISL_17457023, EPI_ISL_17457024, EPI_ISL_17457025, EPI_ISL_17457026, EPI_ISL_17457027, EPI_ISL_17457028, EPI_ISL_17457029, EPI_ISL_17457030, EPI_ISL_17457031	Gen-Probe	Gen-Probe	CARRICK,J. and LINNEN,J.
EPI_ISL_17457032	Gen-Probe	Gen-Probe	Carrick,J.M. and Linnen,J.M.
EPI_ISL_17457033, EPI_ISL_17457034	Gen-Probe	Gen-Probe	CARRICK,J. and LINNEN,J.
EPI_ISL_17457035	Gen-Probe	Gen-Probe	Linnen,J.M. and Carrick,J.M.
EPI_ISL_17457036	Gen-Probe	Gen-Probe	CARRICK,J. and LINNEN,J.
EPI_ISL_17457037, EPI_ISL_17457038, EPI_ISL_17457039, EPI_ISL_17457040, EPI_ISL_17457041, EPI_ISL_17457042, EPI_ISL_17457043, EPI_ISL_17457044, EPI_ISL_17457045, EPI_ISL_17457046, EPI_ISL_17457047, EPI_ISL_17457048, EPI_ISL_17457049, EPI_ISL_17457050, EPI_ISL_17457051, EPI_ISL_17457052, EPI_ISL_17457053, EPI_ISL_17457054, EPI_ISL_17457055, EPI_ISL_17457056, EPI_ISL_17457057, EPI_ISL_17457058, EPI_ISL_17457059, EPI_ISL_17457060, EPI_ISL_17457061, EPI_ISL_17457062, EPI_ISL_17457063, EPI_ISL_17457064, EPI_ISL_17457065, EPI_ISL_17457066, EPI_ISL_17457067, EPI_ISL_17457068, EPI_ISL_17457069, EPI_ISL_17457070			
see above	Gen-Probe	Gen-Probe	Linnen,J.M. and Carrick,J.M.

EPI_ISL_17457071, EPI_ISL_17457072, EPI_ISL_17457073, EPI_ISL_17457074, EPI_ISL_17457075, EPI_ISL_17457076, EPI_ISL_17457078, EPI_ISL_17457079, EPI_ISL_17457080, EPI_ISL_17457081, EPI_ISL_17457082, EPI_ISL_17457083, EPI_ISL_17457084, EPI_ISL_17457085, EPI_ISL_17457086, EPI_ISL_17457087, EPI_ISL_17457088, EPI_ISL_17457089, EPI_ISL_17457090, EPI_ISL_17457091, EPI_ISL_17457092, EPI_ISL_17457093, EPI_ISL_17457094, EPI_ISL_17457095, EPI_ISL_17457096			
see above	Gen-Probe	Gen-Probe	Carrick,J.M. and Linnen,J.M.
EPI_ISL_17457097, EPI_ISL_17457098	Gen-Probe	Gen-Probe	Linnen,J.M. and Carrick,J.M.
EPI_ISL_17457099	Gen-Probe	Gen-Probe	Carrick,J.M. and Linnen,J.M.
EPI_ISL_17457100	Gen-Probe	Gen-Probe	Linnen,J.M. and Carrick,J.M.
EPI_ISL_17457101, EPI_ISL_17457102	Gen-Probe	Gen-Probe	Carrick,J.M. and Linnen,J.M.
EPI_ISL_17457103, EPI_ISL_17457104, EPI_ISL_17457105, EPI_ISL_17457106, EPI_ISL_17457107, EPI_ISL_17457108, EPI_ISL_17457109, EPI_ISL_17457110, EPI_ISL_17457111, EPI_ISL_17457112	Gen-Probe	Gen-Probe	Linnen,J.M. and Carrick,J.M.
EPI_ISL_17457113, EPI_ISL_17457114, EPI_ISL_17457115	Gen-Probe	Gen-Probe	CARRICK,J. and LINNEN,J.
EPI_ISL_17457116	Gen-Probe	Gen-Probe	Linnen,J.M. and Carrick,J.M.
EPI_ISL_17457117, EPI_ISL_17457118, EPI_ISL_17457119, EPI_ISL_17457120, EPI_ISL_17457121, EPI_ISL_17457122, EPI_ISL_17457123, EPI_ISL_17457124, EPI_ISL_17457125, EPI_ISL_17457126, EPI_ISL_17457127			
see above	Gen-Probe	Gen-Probe	Carrick,J.M. and Linnen,J.M.
EPI_ISL_17457128, EPI_ISL_17457129, EPI_ISL_17457130	Gen-Probe	Gen-Probe	Linnen,J.M. and Carrick,J.M.
EPI_ISL_17457131, EPI_ISL_17457132, EPI_ISL_17457133, EPI_ISL_17457134, EPI_ISL_17457135, EPI_ISL_17457136, EPI_ISL_17457137	Gen-Probe	Gen-Probe	Carrick,J.M. and Linnen,J.M.
EPI_ISL_17457138	Gen-Probe	Gen-Probe	Linnen,J.M. and Carrick,J.M.
EPI_ISL_17457139, EPI_ISL_17457140, EPI_ISL_17457141, EPI_ISL_17457142, EPI_ISL_17457143, EPI_ISL_17457144, EPI_ISL_17457145, EPI_ISL_17457146, EPI_ISL_17457147, EPI_ISL_17457148, EPI_ISL_17457149, EPI_ISL_17457150, EPI_ISL_17457151, EPI_ISL_17457152			
see above	Gen-Probe	Gen-Probe	Carrick,J.M. and Linnen,J.M.
EPI_ISL_17457153, EPI_ISL_17457154, EPI_ISL_17457155	Gen-Probe	Gen-Probe	Linnen,J.M. and Carrick,J.M.
EPI_ISL_17457156, EPI_ISL_17457157, EPI_ISL_17457158, EPI_ISL_17457159	Gen-Probe	Gen-Probe	Carrick,J.M. and Linnen,J.M.
EPI_ISL_17457160, EPI_ISL_17457161, EPI_ISL_17457162, EPI_ISL_17457163, EPI_ISL_17457164, EPI_ISL_17457165, EPI_ISL_17457166, EPI_ISL_17457167, EPI_ISL_17457168	Gen-Probe	Gen-Probe	CARRICK,J. and LINNEN,J.
EPI_ISL_17457169	Gen-Probe	Gen-Probe	Carrick,J.M. and Linnen,J.M.
EPI_ISL_17457170, EPI_ISL_17457171, EPI_ISL_17457172, EPI_ISL_17457173, EPI_ISL_17457174, EPI_ISL_17457175, EPI_ISL_17457176, EPI_ISL_17457177, EPI_ISL_17457178, EPI_ISL_17457179, EPI_ISL_17457180, EPI_ISL_17457181, EPI_ISL_17457182, EPI_ISL_17457183, EPI_ISL_17457184, EPI_ISL_17457185, EPI_ISL_17457186			
see above	Gen-Probe	Gen-Probe	CARRICK,J. and LINNEN,J.
EPI_ISL_17457187, EPI_ISL_17457188, EPI_ISL_17457189, EPI_ISL_17457190	Gen-Probe	Gen-Probe	Linnen,J.M. and Carrick,J.M.
EPI_ISL_17457191, EPI_ISL_17457192, EPI_ISL_17457193, EPI_ISL_17457194, EPI_ISL_17457195, EPI_ISL_17457196, EPI_ISL_17457197, EPI_ISL_17457198, EPI_ISL_17457199, EPI_ISL_17457200, EPI_ISL_17457201, EPI_ISL_17457202, EPI_ISL_17457203			
see above	Gen-Probe	Gen-Probe	CARRICK,J. and LINNEN,J.
EPI_ISL_17457204, EPI_ISL_17457205, EPI_ISL_17457206, EPI_ISL_17457207, EPI_ISL_17457208, EPI_ISL_17457209, EPI_ISL_17457210, EPI_ISL_17457211	Gen-Probe	Gen-Probe	Carrick,J.M. and Linnen,J.M.
EPI_ISL_17457212, EPI_ISL_17457213, EPI_ISL_17457214	Gen-Probe	Gen-Probe	CARRICK,J. and LINNEN,J.
EPI_ISL_17457215, EPI_ISL_17457216, EPI_ISL_17457217	Gen-Probe	Gen-Probe	Linnen,J.M. and Carrick,J.M.
EPI_ISL_17457218	Gen-Probe	Gen-Probe	CARRICK,J. and LINNEN,J.
EPI_ISL_17457219	Gen-Probe	Gen-Probe	Linnen,J.M. and Carrick,J.M.
EPI_ISL_17457220, EPI_ISL_17457221, EPI_ISL_17457222	Gen-Probe	Gen-Probe	CARRICK,J. and LINNEN,J.
EPI_ISL_17457223, EPI_ISL_17457224, EPI_ISL_17457225, EPI_ISL_17457226, EPI_ISL_17457227	Gen-Probe	Gen-Probe	Linnen,J.M. and Carrick,J.M.
EPI_ISL_17457228	Gen-Probe	Gen-Probe	CARRICK,J. and LINNEN,J.
EPI_ISL_17457229, EPI_ISL_17457230, EPI_ISL_17457231	Gen-Probe	Gen-Probe	Linnen,J.M. and Carrick,J.M.
EPI_ISL_17457232, EPI_ISL_17457233, EPI_ISL_17457234, EPI_ISL_17457235, EPI_ISL_17457236, EPI_ISL_17457237, EPI_ISL_17457238, EPI_ISL_17457239, EPI_ISL_17457240, EPI_ISL_17457241, EPI_ISL_17457242, EPI_ISL_17457243, EPI_ISL_17457244, EPI_ISL_17457245, EPI_ISL_17457246, EPI_ISL_17457247, EPI_ISL_17457248, EPI_ISL_17457249, EPI_ISL_17457250			
see above	Gen-Probe	Gen-Probe	CARRICK,J. and LINNEN,J.
EPI_ISL_17457251, EPI_ISL_17457252	Gen-Probe	Gen-Probe	Linnen,J.M. and Carrick,J.M.
EPI_ISL_17457253, EPI_ISL_17457254, EPI_ISL_17457255, EPI_ISL_17457256, EPI_ISL_17457257, EPI_ISL_17457258, EPI_ISL_17457259, EPI_ISL_17457260, EPI_ISL_17457261, EPI_ISL_17457262, EPI_ISL_17457263, EPI_ISL_17457264, EPI_ISL_17457265, EPI_ISL_17457266, EPI_ISL_17457267, EPI_ISL_17457268, EPI_ISL_17457269, EPI_ISL_17457270, EPI_ISL_17457271, EPI_ISL_17457272, EPI_ISL_17457273, EPI_ISL_17457274, EPI_ISL_17457275, EPI_ISL_17457276, EPI_ISL_17457277, EPI_ISL_17457278, EPI_ISL_17457279, EPI_ISL_17457280, EPI_ISL_17457281, EPI_ISL_17457282, EPI_ISL_17457283, EPI_ISL_17457284, EPI_ISL_17457285, EPI_ISL_17457286, EPI_ISL_17457287, EPI_ISL_17457288, EPI_ISL_17457289, EPI_ISL_17457290, EPI_ISL_17457291, EPI_ISL_17457292, EPI_ISL_17457293, EPI_ISL_17457294, EPI_ISL_17457295, EPI_ISL_17457296, EPI_ISL_17457297, EPI_ISL_17457298, EPI_ISL_17457299, EPI_ISL_17457300, EPI_ISL_17457301, EPI_ISL_17457302, EPI_ISL_17457303, EPI_ISL_17457304, EPI_ISL_17457305, EPI_ISL_17457306, EPI_ISL_17457307, EPI_ISL_17457308, EPI_ISL_17457309, EPI_ISL_17457310, EPI_ISL_17457311, EPI_ISL_17457312, EPI_ISL_17457313, EPI_ISL_17457314, EPI_ISL_17457315, EPI_ISL_17457316, EPI_ISL_17457317, EPI_ISL_17457318, EPI_ISL_17457319, EPI_ISL_17457320, EPI_ISL_17457321, EPI_ISL_17457322, EPI_ISL_17457323, EPI_ISL_17457324, EPI_ISL_17457325, EPI_ISL_17457326, EPI_ISL_17457327, EPI_ISL_17457328, EPI_ISL_17457329, EPI_ISL_17457330, EPI_ISL_17457331, EPI_ISL_17457332, EPI_ISL_17457333, EPI_ISL_17457334, EPI_ISL_17457335, EPI_ISL_17457336			
see above	Gen-Probe	Gen-Probe	CARRICK,J. and LINNEN,J.
EPI_ISL_17457337, EPI_ISL_17457338, EPI_ISL_17457339, EPI_ISL_17457340, EPI_ISL_17457341, EPI_ISL_17457342, EPI_ISL_17457343, EPI_ISL_17457344, EPI_ISL_17457345, EPI_ISL_17457346, EPI_ISL_17457347, EPI_ISL_17457348, EPI_ISL_17457349, EPI_ISL_17457350, EPI_ISL_17457351, EPI_ISL_17457352, EPI_ISL_17457353, EPI_ISL_17457354, EPI_ISL_17457355, EPI_ISL_17457356, EPI_ISL_17457357, EPI_ISL_17457358, EPI_ISL_17457359, EPI_ISL_17457360, EPI_ISL_17457361, EPI_ISL_17457362, EPI_ISL_17457363, EPI_ISL_17457364, EPI_ISL_17457365, EPI_ISL_17457366, EPI_ISL_17457367, EPI_ISL_17457368, EPI_ISL_17457369, EPI_ISL_17457370, EPI_ISL_17457371, EPI_ISL_17457372, EPI_ISL_17457373, EPI_ISL_17457374, EPI_ISL_17457375, EPI_ISL_17457376, EPI_ISL_17457377, EPI_ISL_17457378, EPI_ISL_17457379, EPI_ISL_17457380, EPI_ISL_17457381, EPI_ISL_17457382, EPI_ISL_17457383, EPI_ISL_17457384, EPI_ISL_17457385, EPI_ISL_17457386, EPI_ISL_17457387, EPI_ISL_17457388, EPI_ISL_17457389, EPI_ISL_17457390, EPI_ISL_17457391, EPI_ISL_17457392, EPI_ISL_17457393, EPI_ISL_17457394, EPI_ISL_17457395, EPI_ISL_17457396, EPI_ISL_17457397, EPI_ISL_17457398, EPI_ISL_17457399, EPI_ISL_17457400, EPI_ISL_17457401, EPI_ISL_17457402, EPI_ISL_17457403, EPI_ISL_17457404, EPI_ISL_17457405, EPI_ISL_17457406, EPI_ISL_17457407, EPI_ISL_17457408, EPI_ISL_17457409, EPI_ISL_17457410, EPI_ISL_17457411, EPI_ISL_17457412, EPI_ISL_17457413, EPI_ISL_17457414, EPI_ISL_17457415, EPI_ISL_17457416, EPI_ISL_17457417, EPI_ISL_17457418, EPI_ISL_17457419, EPI_ISL_17457420, EPI_ISL_17457421, EPI_ISL_17457422, EPI_ISL_17457423, EPI_ISL_17457424, EPI_ISL_17457425, EPI_ISL_17457426, EPI_ISL_17457427, EPI_ISL_17457428, EPI_ISL_17457429, EPI_ISL_17457430, EPI_ISL_17457431, EPI_ISL_17457432, EPI_ISL_17457433, EPI_ISL_17457434, EPI_ISL_17457435, EPI_ISL_17457436, EPI_ISL_17457437, EPI_ISL_17457438, EPI_ISL_17457439, EPI_ISL_17457440, EPI_ISL_17457441, EPI_ISL_17457442, EPI_ISL_17457443, EPI_ISL_17457444, EPI_ISL_17457445, EPI_ISL_17457446, EPI_ISL_17457447, EPI_ISL_17457448, EPI_ISL_17457449, EPI_ISL_17457450, EPI_ISL_17457451, EPI_ISL_17457452, EPI_ISL_17457453, EPI_ISL_17457454, EPI_ISL_17457455, EPI_ISL_17457456, EPI_ISL_17457457, EPI_ISL_17457458, EPI_ISL_17457459, EPI_ISL_17457460, EPI_ISL_17457461, EPI_ISL_17457462, EPI_ISL_17457463, EPI_ISL_17457464, EPI_ISL_17457465, EPI_ISL_17457466, EPI_ISL_17457467, EPI_ISL_17457468, EPI_ISL_17457469, EPI_ISL_17457470, EPI_ISL_17457471, EPI_ISL_17457472, EPI_ISL_17457473, EPI_ISL_17457474, EPI_ISL_17457475, EPI_ISL_17457476, EPI_ISL_17457477, EPI_ISL_17457478, EPI_ISL_17457479, EPI_ISL_17457480, EPI_ISL_17457481, EPI_ISL_17457482, EPI_ISL_17457483, EPI_ISL_17457484, EPI_ISL_17457485, EPI_ISL_17457486, EPI_ISL_17457487, EPI_ISL_17457488, EPI_ISL_17457489, EPI_ISL_17457490, EPI_ISL_17457491, EPI_ISL_17457492, EPI_ISL_17457493, EPI_ISL_17457494, EPI_ISL_17457495, EPI_ISL_17457496, EPI_ISL_17457497, EPI_ISL_17457498, EPI_ISL_17457499, EPI_ISL_17457500, EPI_ISL_17457501, EPI_ISL_17457502, EPI_ISL_17457503, EPI_ISL_17457504, EPI_ISL_17457505, EPI_ISL_17457506, EPI_ISL_17457507, EPI_ISL_17457508, EPI_ISL_17457509, EPI_ISL_17457510, EPI_ISL_17457511, EPI_ISL_17457512, EPI_ISL_17457513, EPI_ISL_17457514, EPI_ISL_17457515, EPI_ISL_17457516, EPI_ISL_17457517, EPI_ISL_17457518, EPI_ISL_17457519, EPI_ISL_17457520, EPI_ISL_17457521, EPI_ISL_17457522, EPI_ISL_17457523, EPI_ISL_17457524, EPI_ISL_17457525, EPI_ISL_17457526, EPI_ISL_17457527, EPI_ISL_17457528, EPI_ISL_17457529, EPI_ISL_17457530, EPI_ISL_17457531, EPI_ISL_17457532, EPI_ISL_17457533, EPI_ISL_17457534, EPI_ISL_17457535, EPI_ISL_17457536, EPI_ISL_17457537, EPI_ISL_17457538, EPI_ISL_17457539, EPI_ISL_17457540, EPI_ISL_17457541, EPI_ISL_17457542, EPI_ISL_17457543, EPI_ISL_17457544, EPI_ISL_17457545, EPI_ISL_17457546, EPI_ISL_17457547, EPI_ISL_17457548, EPI_ISL_17457549, EPI_ISL_17457550, EPI_ISL_17457551, EPI_ISL_17457552, EPI_ISL_17457553, EPI_ISL_17457554, EPI_ISL_17457555, EPI_ISL_17457556, EPI_ISL_17457557, EPI_ISL_17457558, EPI_ISL_17457559, EPI_ISL_17457560, EPI_ISL_17457561, EPI_ISL_17457562, EPI_ISL_17457563, EPI_ISL_17457564, EPI_ISL_17457565, EPI_ISL_17457566, EPI_ISL_17457567, EPI_ISL_17457568, EPI_ISL_17457569, EPI_ISL_17457570, EPI_ISL_17457571, EPI_ISL_17457572, EPI_ISL_17457573, EPI_ISL_17457574, EPI_ISL_17457575, EPI_ISL_17457576, EPI_ISL_17457577, EPI_ISL_17457578, EPI_ISL_17457579, EPI_ISL_17457580, EPI_ISL_17457581, EPI_ISL_17457582, EPI_ISL_17457583, EPI_ISL_17457584, EPI_ISL_17457585, EPI_ISL_17457586, EPI_ISL_17457587, EPI_ISL_17457588, EPI_ISL_17457589, EPI_ISL_17457590, EPI_ISL_17457591, EPI_ISL_17457592, EPI_ISL_17457593, EPI_ISL_17457594, EPI_ISL_17457595, EPI_ISL_17457596, EPI_ISL_17457597, EPI_ISL_17457598, EPI_ISL_17457599, EPI_ISL_17457600, EPI_ISL_17457601, EPI_ISL_17457602, EPI_ISL_17457603, EPI_ISL_17457604, EPI_ISL_17457605, EPI_ISL_17457606, EPI_ISL_17457607, EPI_ISL_17457608, EPI_ISL_17457609, EPI_ISL_17457610, EPI_ISL_17457611, EPI_ISL_17457612, EPI_ISL_17457613, EPI_ISL_17457614, EPI_ISL_17457615, EPI_ISL_17457616, EPI_ISL_17457617, EPI_ISL_17457618, EPI_ISL_17457619, EPI_ISL_17457620, EPI_ISL_17457621, EPI_ISL_17457622, EPI_ISL_17457623, EPI_ISL_17457624, EPI_ISL_17457625, EPI_ISL_17457626, EPI_ISL_17457627, EPI_ISL_17457628, EPI_ISL_17457629, EPI_ISL_17457630, EPI_ISL_17457631, EPI_ISL_17457632, EPI_ISL_17457633, EPI_ISL_17457634, EPI_ISL_17457635, EPI_ISL_17457636, EPI_ISL_17457637, EPI_ISL_17457638, EPI_ISL_17457639, EPI_ISL_17457640, EPI_ISL_17457641, EPI_ISL_17457642, EPI_ISL_17457643, EPI_ISL_17457644, EPI_ISL_17457645, EPI_ISL_17457646, EPI_ISL_17457647, EPI_ISL_17457648, EPI_ISL_17457649, EPI_ISL_17457650, EPI_ISL_17457651, EPI_ISL_17457652, EPI_ISL_17457653, EPI_ISL_17457654, EPI_ISL_17457655, EPI_ISL_17457656, EPI_ISL_17457657, EPI_ISL_17457658, EPI_ISL_17457659, EPI_ISL_17457660, EPI_ISL_17457661, EPI_ISL_17457662, EPI_ISL_17457663, EPI_ISL_17457664, EPI_ISL_17457665, EPI_ISL_17457666, EPI_ISL_17457667, EPI_ISL_17457668, EPI_ISL_17457669, EPI_ISL_17457670, EPI_ISL_17457671, EPI_ISL_17457672			
see above	Gen-Probe	Gen-Probe	Carrick,J.M. and Linnen,J.M.
EPI_ISL_17457673	Genetech Molecular Diagnostics	Genetech Molecular Diagnostics	Herath,I. and Fernadopulle,N.
EPI_ISL_17457674, EPI_ISL_17457675, EPI_ISL_17457676, EPI_ISL_17457677, EPI_ISL_17457678, EPI_ISL_17457679, EPI_ISL_17457680, EPI_ISL_17457681, EPI_ISL_17457682, EPI_ISL_17457683	Gorgas Memorial Institute for Health Studies	Gorgas Memorial Institute for Health Studies	Carrera,J.P., Diaz,Y., Denis,B., Barahona de Mosca,I., Rodriguez,D., Cedeno,I., Arauz,D., Gonzalez,P., Cerezo,L., Moreno,L., Garcia,L., Saenz,L.E., Atencio,M.A., Rojas-Fermin,E., Vizcaino,F., Perez,N., Moreno,B., Lopez-Verges,S., Valderrama,A. and Armien,B.
EPI_ISL_17457684, EPI_ISL_17457685, EPI_ISL_17457686	Gorgas Memorial Institute of Health Studies	Gorgas Memorial Institute of Health Studies	Diaz,Y., Carrera,J.P., Cerezo,L., Arauz,D., Guerra,I., Cisneros,J., Armien,B., Botello,A.M., Arauz,A.B., Gonzalez,V., Lopez,Y., Moreno,L., Lopez-Verges,S. and Moreno,B.A.
EPI_ISL_17457687, EPI_ISL_17457688, EPI_ISL_17457689, EPI_ISL_17457690	Guangdong Center for Disease Control and Prevention	Guangdong Center for Disease Control and Prevention	Wu,D., Wu,J., Zhang,Q., Zhong,H., Ke,C., Deng,X., Guan,D., Li,H., Zhang,Y., Zhou,H., He,J., Li,L. and Yang,X.
EPI_ISL_17457691	Guangzhou Center For Disease Control and Prevention	Guangzhou Center For Disease Control and Prevention	Chen,W., Bai,Z., Wang,P. and Liu,L.
EPI_ISL_17457692, EPI_ISL_17457693, EPI_ISL_17457694, EPI_ISL_17457695, EPI_ISL_17457696, EPI_ISL_17457697, EPI_ISL_17457698, EPI_ISL_17457699	The State Key Laboratory of Respiratory Diseases, Guangzhou Medical University	Guangzhou Medical University	Lu,X., Li,X., Mo,Z., Jin,F., Wang,B., Huang,J., Huang,J., Zhao,H. and Shi,L.
EPI_ISL_17457700	Henan Provincial Center for Disease Control and	Henan Provincial Center for Disease Control and	Li,X., Li,D., Wang,H., Su,J., Huang,X. and Guo,W.

	Prevention	Prevention	
EPI_ISL_17457701	Henan Provincial Center for Disease Control and Prevention	Henan Provincial Center for Disease Control and Prevention	Xingle,L., Yi,L., Ruolin,W., Jia,S., Bianli,X. and Xueyong,H.
EPI_ISL_17457702, EPI_ISL_17457703, EPI_ISL_17457704, EPI_ISL_17457705	Hokkaido University	Hokkaido University	Rahman,M., Yamagishi,J., Rahim,R., Hasan,A. and Sobhan,A.
EPI_ISL_17457706	Hospital Clinic i Provincial de Barcelona	Hospital Clinic i Provincial de Barcelona	Navero-Castillejos,J., Gonzalo-Abril,V. and Martinez,M.J.
EPI_ISL_17457707, EPI_ISL_17457708	Indian Council of Medical Research	Indian Council of Medical Research	Nagarajan,M., Itta,K., Sundaram,S.G. and Paluru,V.
EPI_ISL_17457709, EPI_ISL_17457710	Indian Council of Medical Research	Indian Council of Medical Research	Taraphdar,D., Sarkar,A., Basak,S. and Chatterjee,S.
EPI_ISL_17457711	Indian Council of Medical Research	Indian Council of Medical Research	Taraphdar,D. and Chatterjee,S.
EPI_ISL_17457712	Indian Council of Medical Research	Indian Council of Medical Research	Nagarajan,M., Itta,K., Sundaram,S.G. and Paluru,V.
EPI_ISL_17457713	Indian Council of Medical Research	Indian Council of Medical Research	Taraphdar,D. and Chatterjee,S.
EPI_ISL_17457714	Indian Council of Medical Research	Indian Council of Medical Research	Taraphdar,D., Sarkar,A., Basak,S. and Chatterjee,S.
EPI_ISL_17457715, EPI_ISL_17457716, EPI_ISL_17457717	Indian Council of Medical Research	Indian Council of Medical Research	Nagarajan,M., Itta,K., Sundaram,S.G. and Paluru,V.
EPI_ISL_17457718, EPI_ISL_17457719	Indian Council of Medical Research	Indian Council of Medical Research	Taraphdar,D. and Chatterjee,S.
EPI_ISL_17457720	Indian Council of Medical Research	Indian Council of Medical Research	Taraphdar,D., Sarkar,A., Basak,S. and Chatterjee,S.
EPI_ISL_17457721	Indian Council of Medical Research	Indian Council of Medical Research	Nagarajan,M., Itta,K., Sundaram,S.G. and Paluru,V.
EPI_ISL_17457722	Indian Council of Medical Research	Indian Council of Medical Research	Taraphdar,D. and Chatterjee,S.
EPI_ISL_17457723	Indian Council of Medical Research	Indian Council of Medical Research	Taraphdar,D., Sarkar,A. and Chatterjee,S.
EPI_ISL_17457724	Indian Council of Medical Research	Indian Council of Medical Research	Taraphdar,D., Sarkar,A., Basak,S. and Chatterjee,S.
EPI_ISL_17457725, EPI_ISL_17457726	Indian Council of Medical Research	Indian Council of Medical Research	Taraphdar,D. and Chatterjee,S.
EPI_ISL_17457727, EPI_ISL_17457728, EPI_ISL_17457729, EPI_ISL_17457730, EPI_ISL_17457731, EPI_ISL_17457732, EPI_ISL_17457733	Indian Council of Medical Research	Indian Council of Medical Research	Nagarajan,M., Itta,K., Sundaram,S.G. and Paluru,V.
EPI_ISL_17457734	Indian Council of Medical Research	Indian Council of Medical Research	Taraphdar,D. and Chatterjee,S.
EPI_ISL_17457735	Indian Council of Medical Research	Indian Council of Medical Research	Nagarajan,M., Itta,K., Sundaram,S.G. and Paluru,V.
EPI_ISL_17457736	Indian Council of Medical Research	Indian Council of Medical Research	Taraphdar,D., Sarkar,A., Basak,S. and Chatterjee,S.
EPI_ISL_17457737	Indian Council of Medical Research	Indian Council of Medical Research	Nagarajan,M., Itta,K., Sundaram,S.G. and Paluru,V.
EPI_ISL_17457738, EPI_ISL_17457739, EPI_ISL_17457740	Indian Council of Medical Research	Indian Council of Medical Research	Taraphdar,D., Sarkar,A. and Chatterjee,S.
EPI_ISL_17457741, EPI_ISL_17457742	Indian Council of Medical Research	Indian Council of Medical Research	Taraphdar,D. and Chatterjee,S.
EPI_ISL_17457743	Indian Council of Medical Research	Indian Council of Medical Research	Taraphdar,D., Sarkar,A., Basak,S. and Chatterjee,S.
EPI_ISL_17457744	Indian Council of Medical Research	Indian Council of Medical Research	Nagarajan,M., Itta,K., Sundaram,S.G. and Paluru,V.
EPI_ISL_17457745, EPI_ISL_17457746, EPI_ISL_17457747, EPI_ISL_17457748, EPI_ISL_17457749, EPI_ISL_17457750, EPI_ISL_17457751, EPI_ISL_17457752, EPI_ISL_17457753	Indian Council of Medical Research	Indian Council of Medical Research	Taraphdar,D. and Chatterjee,S.
EPI_ISL_17457754	Indian Council of Medical Research	Indian Council of Medical Research	Taraphdar,D., Sarkar,A., Basak,S. and Chatterjee,S.
EPI_ISL_17457755	Indian Council of Medical Research	Indian Council of Medical Research	Taraphdar,D. and Chatterjee,S.
EPI_ISL_17457756	Indian Council of Medical Research	Indian Council of Medical Research	Nagarajan,M., Itta,K., Sundaram,S.G. and Paluru,V.
EPI_ISL_17457757, EPI_ISL_17457758, EPI_ISL_17457759, EPI_ISL_17457760, EPI_ISL_17457761	Indian Council of Medical Research, National Institute of Research in Tribal Health, Division Virology and Zooneses	Indian Council of Medical Research, National Institute of Research in Tribal Health, Division Virology and Zooneses	Barde,P.V., Chand,G., Joshi,P., Shivlata,L., Tiwari,S., Sahare,L., Ukey,M., Yadav,P., Mourya,D. and Das,A.
EPI_ISL_17457762	Indian Council of Medical Research, National Institute of Research in Tribal Health, Division Virology and Zooneses	Indian Council of Medical Research, National Institute of Research in Tribal Health, Division Virology and Zooneses	Barde,P.V., Ukey,M.J. and Joshi,P.D.
EPI_ISL_17457763, EPI_ISL_17457764	Indian Council of Medical Research, National Institute of Research in Tribal Health, Division Virology and Zooneses	Indian Council of Medical Research, National Institute of Research in Tribal Health, Division Virology and Zooneses	Barde,P.V., Chand,G., Joshi,P., Shivlata,L., Tiwari,S., Sahare,L., Ukey,M., Yadav,P., Mourya,D. and Das,A.
EPI_ISL_17457765	Indian Council of Medical Research, National Institute of Research in Tribal Health, Division Virology and Zooneses	Indian Council of Medical Research, National Institute of Research in Tribal Health, Division Virology and Zooneses	Barde,P.V., Ukey,M.J. and Joshi,P.D.
EPI_ISL_17457766, EPI_ISL_17457767	Indian Council of Medical Research, National Institute of Research in Tribal Health, Division Virology and Zooneses	Indian Council of Medical Research, National Institute of Research in Tribal Health, Division Virology and Zooneses	Barde,P.V., Chand,G., Joshi,P., Shivlata,L., Tiwari,S., Sahare,L., Ukey,M., Yadav,P., Mourya,D. and Das,A.
EPI_ISL_17457768	Indian Council of Medical Research, National Institute of Research in Tribal Health, Division Virology and Zooneses	Indian Council of Medical Research, National Institute of Research in Tribal Health, Division Virology and Zooneses	Chand,G., Godbole,S., Shivlata,L., Sahare,L., Ukey,M., Kaushal,L.S. and Barde,P.V.
EPI_ISL_17457769	Indian Council of Medical Research, National Institute of Research in Tribal Health, Division Virology and Zooneses	Indian Council of Medical Research, National Institute of Research in Tribal Health, Division Virology and Zooneses	Barde,P.V., Chand,G., Joshi,P., Shivlata,L., Tiwari,S., Sahare,L., Ukey,M., Yadav,P., Mourya,D. and Das,A.
EPI_ISL_17457770	Indian Council of Medical Research, National Institute of Research in Tribal Health, Division Virology and Zooneses	Indian Council of Medical Research, National Institute of Research in Tribal Health, Division Virology and Zooneses	Barde,P.V., Ukey,M.J. and Joshi,P.D.
EPI_ISL_17457771, EPI_ISL_17457772, EPI_ISL_17457773, EPI_ISL_17457774, EPI_ISL_17457775	Indian Council of Medical Research, National Institute of Research in Tribal Health, Division Virology and Zooneses	Indian Council of Medical Research, National Institute of Research in Tribal Health, Division Virology and Zooneses	Barde,P.V., Chand,G., Joshi,P., Shivlata,L., Tiwari,S., Sahare,L., Ukey,M., Yadav,P., Mourya,D. and Das,A.
EPI_ISL_17457776	Indian Council of Medical Research, National Institute of Research in Tribal Health, Division Virology and Zooneses	Indian Council of Medical Research, National Institute of Research in Tribal Health, Division Virology and Zooneses	Chand,G., Godbole,S., Shivlata,L., Sahare,L., Ukey,M., Kaushal,L.S. and Barde,P.V.
EPI_ISL_17457777	Indian Council of Medical Research, National Institute of Research in Tribal Health, Division Virology and Zooneses	Indian Council of Medical Research, National Institute of Research in Tribal Health, Division Virology and Zooneses	Barde,P.V., Chand,G., Joshi,P., Shivlata,L., Tiwari,S., Sahare,L., Ukey,M., Yadav,P., Mourya,D. and Das,A.
EPI_ISL_17457778, EPI_ISL_17457779, EPI_ISL_17457780	Indian Council of Medical Research, National Institute of Research in Tribal Health, Division Virology and Zooneses	Indian Council of Medical Research, National Institute of Research in Tribal Health, Division Virology and Zooneses	Barde,P.V., Ukey,M.J. and Joshi,P.D.
EPI_ISL_17457781, EPI_ISL_17457782	Indian Council of Medical Research, National Institute of Research in Tribal Health, Division Virology and Zooneses	Indian Council of Medical Research, National Institute of Research in Tribal Health, Division Virology and Zooneses	Barde,P.V., Chand,G., Joshi,P., Shivlata,L., Tiwari,S., Sahare,L., Ukey,M., Yadav,P., Mourya,D. and Das,A.
EPI_ISL_17457783	Insittuto Nacional de Medicina Tropical	Insittuto Nacional de Medicina Tropical	Tauro,L.B., Cardoso,C.W., Souza,R.L., Nascimento,L.C., Santos,D.Rd., Campos,G.S., Sardi,S., Reis,O.Bd., Reis,M.G., Kitron,U. and Ribeiro,G.S.
EPI_ISL_17457784	Insittuto Nacional de Medicina Tropical	Insittuto Nacional de Medicina Tropical	Tauro,L.B., Cardoso,C.W., Jacob,L.C., Lima,R.S., Campos,G.S., Sardi,S., Carvalho,R.H., Reis,M.G and Ribeiro,G.S.
EPI_ISL_17457785, EPI_ISL_17457786, EPI_ISL_17457787, EPI_ISL_17457788, EPI_ISL_17457789	Insittuto Nacional de Medicina Tropical	Insittuto Nacional de Medicina Tropical	Tauro,L.B., Cardoso,C.W., Souza,R.L., Nascimento,L.C., Santos,D.Rd., Campos,G.S., Sardi,S., Reis,O.Bd., Reis,M.G., Kitron,U. and Ribeiro,G.S.
EPI_ISL_17457790, EPI_ISL_17457791, EPI_ISL_17457792, EPI_ISL_17457793, EPI_ISL_17457794, EPI_ISL_17457795	Institut Louis Malarde	Institut Louis Malarde	Teissier,A., Naivalu,T. and Paofaaite,T.
EPI_ISL_17457796, EPI_ISL_17457797	Institut Louis Malarde	Institut Louis Malarde	Kama,M., Aubry,M., Naivalu,T., Vanhomwegen,J., Mariteragi-Helle,T., Teissier,A., Paoaafaite,T., Hue,S., Hibberd,M., Manuguerra,J.-C., Christi,K., Watson,C., Nilles,E., Aaskov,J., Lau,C., Musso,D., Kucharski,A. and Cao-Lormeau,V.-M.
EPI_ISL_17457798	Institut Louis Malarde	Institut Louis Malarde	Nhan,T.X., Claverie,A., Roche,C., Teissier,A., Colleuil,M., Baudet,J.M., Cao-Lormeau,V.M. and Musso,D.
EPI_ISL_17457799, EPI_ISL_17457800	Institut Louis Malarde	Institut Louis Malarde	Aubry,M., Teissier,A., Roche,C., Richard,V., Shan Yan,A., Zisou,K., Rouault,E., Maria,V., Lastere,S., Cao-Lormeau,V.M. and Musso,D.
EPI_ISL_17457801, EPI_ISL_17457802, EPI_ISL_17457803, EPI_ISL_17457804	Institut Louis Malarde	Institut Louis Malarde	Teissier,A., Naivalu,T. and Paofaaite,T.

EPI_ISL_17457805	Institut Louis Malarde	Institut Louis Malarde	Kama,M., Aubry,M., Naivalu,T., Vanhomwegen,J., Mariteragi-Helle,T., Teissier,A., Paoaafaite,T., Hue,S., Hibberd,M., Manuguerra,J.-C., Christi,K., Watson,C., Nilles,E., Aaskov,J., Lau,C., Musso,D., Kucharski,A. and Cao-Lormeau,V.-M.
EPI_ISL_17457806, EPI_ISL_17457807, EPI_ISL_17457808, EPI_ISL_17457809, EPI_ISL_17457810, EPI_ISL_17457811, EPI_ISL_17457812, EPI_ISL_17457813, EPI_ISL_17457814	Institut Louis Malarde	Institut Louis Malarde	Teissier,A., Naivalu,T. and Paofaaite,T.
EPI_ISL_17457815	Institut Louis Malarde	Institut Louis Malarde	Kama,M., Aubry,M., Naivalu,T., Vanhomwegen,J., Mariteragi-Helle,T., Teissier,A., Paoaafaite,T., Hue,S., Hibberd,M., Manuguerra,J.-C., Christi,K., Watson,C., Nilles,E., Aaskov,J., Lau,C., Musso,D., Kucharski,A. and Cao-Lormeau,V.-M.
EPI_ISL_17457816	Institut Louis Malarde	Institut Louis Malarde	Teissier,A., Naivalu,T. and Paofaaite,T.
EPI_ISL_17457817	Institut Louis Malarde	Institut Louis Malarde	Aubry,M., Teissier,A., Roche,C., Richard,V., Shan Yan,A., Zisou,K., Rouault,E., Maria,V., Lastere,S., Cao-Lormeau,V.M. and Musso,D.
EPI_ISL_17457818	Institut Louis Malarde	Institut Louis Malarde	Kama,M., Aubry,M., Naivalu,T., Vanhomwegen,J., Mariteragi-Helle,T., Teissier,A., Paoaafaite,T., Hue,S., Hibberd,M., Manuguerra,J.-C., Christi,K., Watson,C., Nilles,E., Aaskov,J., Lau,C., Musso,D., Kucharski,A. and Cao-Lormeau,V.-M.
EPI_ISL_17457819, EPI_ISL_17457820	Institut Louis Malarde	Institut Louis Malarde	Teissier,A., Naivalu,T. and Paofaaite,T.
EPI_ISL_17457821	Institut Pasteur	Institut Pasteur	Brian,A., Maresharu,V., Charneau,P., Suku,P. and Despres,P.
EPI_ISL_17457822	Institut Pasteur	Institut Pasteur	Brehin,A., Marechal,V., Charneau,P., Souque,P. and Despres,P.
EPI_ISL_17457823, EPI_ISL_17457824	Institut Pasteur	Institut Pasteur	Despres,P., Brehin,A.C., Marechal,V., Charneau,P. and Souque,P.
EPI_ISL_17457825	Institut Pasteur	Institut Pasteur	Brian,A., Maresharu,V., Charneau,P., Suku,P. and Despres,P.
EPI_ISL_17457826	Institut Pasteur	Institut Pasteur	Habel,A., Tangy,F., Brandler,S. and Despres,P.
EPI_ISL_17457827, EPI_ISL_17457828, EPI_ISL_17457829, EPI_ISL_17457830, EPI_ISL_17457831, EPI_ISL_17457832, EPI_ISL_17457833, EPI_ISL_17457834, EPI_ISL_17457835, EPI_ISL_17457836	Institut Pasteur	Institut Pasteur	Brian,A., Maresharu,V., Charneau,P., Suku,P. and Despres,P.
EPI_ISL_17457837	Institut Pasteur	Institut Pasteur	Despres,P., Brehin,A.C., Marechal,V., Charneau,P. and Souque,P.
EPI_ISL_17457838	Institut Pasteur	Institut Pasteur	Brian,A., Maresharu,V., Charneau,P., Suku,P. and Despres,P.
EPI_ISL_17457839, EPI_ISL_17457840	Institut Pasteur	Institut Pasteur	Despres,P., Brehin,A.C., Marechal,V., Charneau,P. and Souque,P.
EPI_ISL_17457841	Institut Pasteur	Institut Pasteur	Brian,A., Maresharu,V., Charneau,P., Suku,P. and Despres,P.
EPI_ISL_17457842	Institut Pasteur	Institut Pasteur	Despres,P., Brehin,A.C., Marechal,V., Charneau,P. and Souque,P.
EPI_ISL_17457843	Institut Pasteur	Institut Pasteur	Tangy,F., Brandler,S., Despres,P. and Habel,A.
EPI_ISL_17457844, EPI_ISL_17457845	Institut Pasteur	Institut Pasteur	Brehin,A., Marechal,V., Charneau,P., Souque,P. and Despres,P.
EPI_ISL_17457846, EPI_ISL_17457847	Institut Pasteur	Institut Pasteur	Despres,P., Brehin,A.C., Marechal,V., Charneau,P. and Souque,P.
EPI_ISL_17457848, EPI_ISL_17457849	Institut Pasteur	Institut Pasteur	Tangy,F., Brandler,S., Despres,P. and Habel,A.
EPI_ISL_17457850	Institut Pasteur, Genotyping of Pathogens and Public Health	Institut Pasteur, Genotyping of Pathogens and Public Health	Grandadam,M., Diancourt,L., Caro,V. and Brey,P.T.
EPI_ISL_17457851	Institut Pasteur	Institut Pasteur	Tangy,F., Brandler,S., Despres,P. and Habel,A.
EPI_ISL_17457852, EPI_ISL_17457853, EPI_ISL_17457854, EPI_ISL_17457855	Institut Pasteur	Institut Pasteur	Despres,P., Brehin,A.C., Marechal,V., Charneau,P. and Souque,P.
EPI_ISL_17457856	Institut Pasteur	Institut Pasteur	Brian,A., Maresharu,V., Charneau,P., Suku,P. and Despres,P.
EPI_ISL_17457857	Institut Pasteur	Institut Pasteur	Despres,P., Brehin,A.C., Marechal,V., Charneau,P. and Souque,P.
EPI_ISL_17457858	Institut Pasteur	Institut Pasteur	Brian,A., Maresharu,V., Charneau,P., Suku,P. and Despres,P.
EPI_ISL_17457859	Institut Pasteur	Institut Pasteur	Tangy,F., Brandler,S., Despres,P. and Habel,A.
EPI_ISL_17457860	Institut Pasteur	Institut Pasteur	Despres,P., Brehin,A.C., Marechal,V., Charneau,P. and Souque,P.
EPI_ISL_17457861	Institut Pasteur	Institut Pasteur	Brehin,A., Marechal,V., Charneau,P., Souque,P. and Despres,P.
EPI_ISL_17457862, EPI_ISL_17457863, EPI_ISL_17457864, EPI_ISL_17457865, EPI_ISL_17457866, EPI_ISL_17457867, EPI_ISL_17457868, EPI_ISL_17457869	Institut Pasteur	Institut Pasteur	Despres,P., Brehin,A.C., Marechal,V., Charneau,P. and Souque,P.
EPI_ISL_17457870, EPI_ISL_17457871	Institut Pasteur	Institut Pasteur	Brian,A., Maresharu,V., Charneau,P., Suku,P. and Despres,P.
EPI_ISL_17457872, EPI_ISL_17457873	Institut Pasteur	Institut Pasteur	Tangy,F., Brandler,S., Despres,P. and Habel,A.
EPI_ISL_17457874	Institut Pasteur	Institut Pasteur	Despres,P., Brehin,A.C., Marechal,V., Charneau,P. and Souque,P.
EPI_ISL_17457875, EPI_ISL_17457876, EPI_ISL_17457877, EPI_ISL_17457878, EPI_ISL_17457879, EPI_ISL_17457880	Institut Pasteur	Institut Pasteur	Brehin,A., Marechal,V., Charneau,P., Souque,P. and Despres,P.
EPI_ISL_17457881, EPI_ISL_17457882	Institut Pasteur	Institut Pasteur	Despres,P., Brehin,A.C., Marechal,V., Charneau,P. and Souque,P.
EPI_ISL_17457883, EPI_ISL_17457884, EPI_ISL_17457885, EPI_ISL_17457886, EPI_ISL_17457887	Institut Pasteur	Institut Pasteur	Brehin,A., Marechal,V., Charneau,P., Souque,P. and Despres,P.
EPI_ISL_17457888	Institut Pasteur	Institut Pasteur	Despres,P., Brehin,A.C., Marechal,V., Charneau,P. and Souque,P.
EPI_ISL_17457889	Institut Pasteur	Institut Pasteur	Brehin,A., Marechal,V., Charneau,P., Souque,P. and Despres,P.
EPI_ISL_17457890	Institut Pasteur	Institut Pasteur	Despres,P., Brehin,A.C., Marechal,V., Charneau,P. and Souque,P.
EPI_ISL_17457891, EPI_ISL_17457892, EPI_ISL_17457893, EPI_ISL_17457894	Institut Pasteur	Institut Pasteur	Brehin,A., Marechal,V., Charneau,P., Souque,P. and Despres,P.
EPI_ISL_17457895, EPI_ISL_17457896, EPI_ISL_17457897, EPI_ISL_17457898, EPI_ISL_17457899, EPI_ISL_17457900	Institut Pasteur, Genotyping of Pathogens and Public Health	Institut Pasteur, Genotyping of Pathogens and Public Health	Grandadam,M., Diancourt,L., Caro,V. and Brey,P.T.
EPI_ISL_17457901	Institut Pasteur	Institut Pasteur	Despres,P., Brehin,A.C., Marechal,V., Charneau,P. and Souque,P.
EPI_ISL_17457902	Institut Pasteur	Institut Pasteur	Brehin,A., Marechal,V., Charneau,P., Souque,P. and Despres,P.
EPI_ISL_17457903	Institut Pasteur, Genotyping of Pathogens and Public Health	Institut Pasteur, Genotyping of Pathogens and Public Health	Grandadam,M., Diancourt,L., Caro,V. and Brey,P.T.
EPI_ISL_17457904	Institut Pasteur	Institut Pasteur	Despres,P., Brehin,A.C., Marechal,V., Charneau,P. and Souque,P.
EPI_ISL_17457905	Institut Pasteur	Institut Pasteur	Habel,A., Tangy,F., Brandler,S. and Despres,P.
EPI_ISL_17457906	Institut Pasteur	Institut Pasteur	Brehin,A., Marechal,V., Charneau,P., Souque,P. and Despres,P.
EPI_ISL_17457907	Institut Pasteur	Institut Pasteur	Despres,P., Brehin,A.C., Marechal,V., Charneau,P. and Souque,P.
EPI_ISL_17457908	Institut Pasteur	Institut Pasteur	Brehin,A., Marechal,V., Charneau,P., Souque,P. and Despres,P.
EPI_ISL_17457909	Institut Pasteur	Institut Pasteur	Habel,A., Tangy,F., Brandler,S. and Despres,P.
EPI_ISL_17457910, EPI_ISL_17457911, EPI_ISL_17457912	Institut Pasteur	Institut Pasteur	Brehin,A., Marechal,V., Charneau,P., Souque,P. and Despres,P.
EPI_ISL_17457913, EPI_ISL_17457914, EPI_ISL_17457915	Institut Pasteur	Institut Pasteur	Despres,P., Brehin,A.C., Marechal,V., Charneau,P. and Souque,P.
EPI_ISL_17457916, EPI_ISL_17457917	Institut Pasteur	Institut Pasteur	Brehin,A., Marechal,V., Charneau,P., Souque,P. and Despres,P.
EPI_ISL_17457918, EPI_ISL_17457919, EPI_ISL_17457920, EPI_ISL_17457921, EPI_ISL_17457922, EPI_ISL_17457923, EPI_ISL_17457924, EPI_ISL_17457925, EPI_ISL_17457926	Institut Pasteur	Institut Pasteur	Despres,P., Brehin,A.C., Marechal,V., Charneau,P. and Souque,P.
EPI_ISL_17457927, EPI_ISL_17457928, EPI_ISL_17457929	Institut Pasteur	Institut Pasteur	Habel,A., Tangy,F., Brandler,S. and Despres,P.
EPI_ISL_17457930	Institut Pasteur	Institut Pasteur	Despres,P., Brehin,A.C., Marechal,V., Charneau,P. and Souque,P.
EPI_ISL_17457931, EPI_ISL_17457932	Institut Pasteur	Institut Pasteur	Brian,A., Maresharu,V., Charneau,P., Suku,P. and Despres,P.
EPI_ISL_17457933	Institut Pasteur	Institut Pasteur	Grandadam,M., Diancourt,L., Caro,V. and Brey,P.T.
EPI_ISL_17457934, EPI_ISL_17457935	Institut Pasteur	Institut Pasteur	Vidalain,P.O., Tangy,F., Jacob,Y. and Lucas-Hourani,M.
EPI_ISL_17457936, EPI_ISL_17457937	Institut Pasteur	Institut Pasteur	Vidalain,P.O., Tangy,F., Jacob,Y., Lucas-Hourani,M., Munier-Lehmann,H. and Lupan,A.
EPI_ISL_17457938, EPI_ISL_17457939	Institut Pasteur	Institut Pasteur	Despres,P., Brehin,A.C., Marechal,V., Charneau,P. and Souque,P.
EPI_ISL_17457940	Institut Pasteur	Institut Pasteur	Brian,A., Maresharu,V., Charneau,P., Suku,P. and Despres,P.
EPI_ISL_17457941	Institut Pasteur, Genotyping of Pathogens and Public Health	Institut Pasteur, Genotyping of Pathogens and Public Health	Grandadam,M., Diancourt,L., Caro,V. and Brey,P.T.

EPI_ISL_17457942	Institut Pasteur	Institut Pasteur	Schuffenecker,I., Iteman,I., Michault,A., Murri,S., Frangeul,L., Vaney,M.C., Lavenir,R., Pardigon,N., Reynes,J.M., Pettinelli,F., Biscornet,L., Diancourt,L., Michel,S., Duquerroy,S., Guigon,G., Frenkiel,M.P., Brehin,A.C., Cubito,N., Despres,P., Kunst,F., Rey,F.A., Zeller,H. and Brisse,S.
EPI_ISL_17457943	Institut Pasteur	Institut Pasteur	Carbonnier,M., Boumahni,B., Fourmaintraux,A., Michault,A. and Schuffenecker,I.
EPI_ISL_17457944, EPI_ISL_17457945, EPI_ISL_17457946, EPI_ISL_17457947	Institut Pasteur	Institut Pasteur	Schuffenecker,I., Iteman,I., Michault,A., Murri,S., Frangeul,L., Vaney,M.C., Lavenir,R., Pardigon,N., Reynes,J.M., Pettinelli,F., Biscornet,L., Diancourt,L., Michel,S., Duquerroy,S., Guigon,G., Frenkiel,M.P., Brehin,A.C., Cubito,N., Despres,P., Kunst,F., Rey,F.A., Zeller,H. and Brisse,S.
EPI_ISL_17457948	Institut Pasteur	Institut Pasteur	Pistone,T., Ezzedine,K., Schuffenecker,I., Receveur,M.C. and Malvy,D.
EPI_ISL_17457949	Institut Pasteur	Institut Pasteur	Schuffenecker,I., Iteman,I., Michault,A., Murri,S., Frangeul,L., Vaney,M.C., Lavenir,R., Pardigon,N., Reynes,J.M., Pettinelli,F., Biscornet,L., Diancourt,L., Michel,S., Duquerroy,S., Guigon,G., Frenkiel,M.P., Brehin,A.C., Cubito,N., Despres,P., Kunst,F., Rey,F.A., Zeller,H. and Brisse,S.
EPI_ISL_17457950	Institut Pasteur	Institut Pasteur	Brehin,A., Marechal,V., Charneau,P., Souque,P. and Despres,P.
EPI_ISL_17457951, EPI_ISL_17457952	Institut Pasteur	Institut Pasteur	Despres,P., Brehin,A.C., Marechal,V., Charneau,P. and Souque,P.
EPI_ISL_17457953	Institut Pasteur	Institut Pasteur	Habel,A., Tangy,F., Brandler,S. and Despres,P.
EPI_ISL_17457954	Institut Pasteur	Institut Pasteur	Brian,A., Maresharu,V., Charneau,P., Suku,P. and Despres,P.
EPI_ISL_17457955, EPI_ISL_17457956, EPI_ISL_17457957	Institut Pasteur	Institut Pasteur	Despres,P., Brehin,A.C., Marechal,V., Charneau,P. and Souque,P.
EPI_ISL_17457958, EPI_ISL_17457959	Institut Pasteur	Institut Pasteur	Brian,A., Maresharu,V., Charneau,P., Suku,P. and Despres,P.
EPI_ISL_17457960, EPI_ISL_17457961, EPI_ISL_17457962, EPI_ISL_17457963, EPI_ISL_17457964	Institut Pasteur	Institut Pasteur	Despres,P., Brehin,A.C., Marechal,V., Charneau,P. and Souque,P.
EPI_ISL_17457965, EPI_ISL_17457966	Institut Pasteur	Institut Pasteur	Brian,A., Maresharu,V., Charneau,P., Suku,P. and Despres,P.
EPI_ISL_17457967	Institut Pasteur	Institut Pasteur	Despres,P., Brehin,A.C., Marechal,V., Charneau,P. and Souque,P.
EPI_ISL_17457968, EPI_ISL_17457969, EPI_ISL_17457970	Institut Pasteur	Institut Pasteur	Brian,A., Maresharu,V., Charneau,P., Suku,P. and Despres,P.
EPI_ISL_17457971, EPI_ISL_17457972	Institut Pasteur	Institut Pasteur	Despres,P., Brehin,A.C., Marechal,V., Charneau,P. and Souque,P.
EPI_ISL_17457973	Institut Pasteur Guadeloupe	Institut Pasteur Guadeloupe	Vega Rua,A.
EPI_ISL_17457974, EPI_ISL_17457975, EPI_ISL_17457976	Institut Pasteur de Bangui	Institut Pasteur de Bangui	Desdouits,M., Kamgang,B., Berthet,N., Tricou,V., Ngoagouni,C., Gessain,A., Manuguerra,J.C., Nakoune,E. and Kazanji,M.
EPI_ISL_17457977	Institut Pasteur de Bangui	Institut Pasteur de Bangui	Tricou,V., Desdouits,M., Nakoune,E., Gessain,A., Kazanji,M. and Berthet,N.
EPI_ISL_17457978, EPI_ISL_17457979, EPI_ISL_17457980, EPI_ISL_17457981, EPI_ISL_17457982, EPI_ISL_17457983, EPI_ISL_17457984, EPI_ISL_17457985, EPI_ISL_17457986, EPI_ISL_17457987, EPI_ISL_17457988, EPI_ISL_17457989, EPI_ISL_17457990, EPI_ISL_17457991, EPI_ISL_17457992, EPI_ISL_17457993			
see above	Institut Pasteur de Bangui	Institut Pasteur de Bangui	Desdouits,M., Kamgang,B., Berthet,N., Tricou,V., Ngoagouni,C., Gessain,A., Manuguerra,J.C., Nakoune,E. and Kazanji,M.
EPI_ISL_17457994	Institut Pasteur de Bangui	Institut Pasteur de Bangui	Tricou,V., Desdouits,M., Nakoune,E., Gessain,A., Kazanji,M. and Berthet,N.
EPI_ISL_17457995, EPI_ISL_17457996, EPI_ISL_17457997, EPI_ISL_17457998, EPI_ISL_17457999, EPI_ISL_17458000, EPI_ISL_17458001, EPI_ISL_17458002, EPI_ISL_17458003, EPI_ISL_17458004, EPI_ISL_17458005			
see above	Institut Pasteur de Bangui	Institut Pasteur de Bangui	Desdouits,M., Kamgang,B., Berthet,N., Tricou,V., Ngoagouni,C., Gessain,A., Manuguerra,J.C., Nakoune,E. and Kazanji,M.
EPI_ISL_17458006, EPI_ISL_17458007, EPI_ISL_17458008, EPI_ISL_17458009, EPI_ISL_17458010, EPI_ISL_17458011, EPI_ISL_17458012, EPI_ISL_17458013, EPI_ISL_17458014, EPI_ISL_17458015, EPI_ISL_17458016, EPI_ISL_17458017, EPI_ISL_17458018			
see above	Institut Pasteur de Nouvelle-Caledonie	Institut Pasteur de Nouvelle-Caledonie	Aubry,M., Ishikawa,S., O'Connor,O., Naivalu,T., Henderson,A., Teissier,A., Paoaafaite,T., Inizan,C., Nilles,E.J., Kama,M., Christi,K., Dupont-Rouzeyrol,M., Kucharski,A.J., Simon-Lorriere,E. and Cao-Lormeau,V.-M.
EPI_ISL_17458019, EPI_ISL_17458020, EPI_ISL_17458021, EPI_ISL_17458022, EPI_ISL_17458023, EPI_ISL_17458024, EPI_ISL_17458025, EPI_ISL_17458026	Institut Pasteur du Cambodge	Institut Pasteur du Cambodge	Duong,V., Andries,A.C., Ngan,C., Sok,T., Richner,B., Asgari-Jirhandeh,N., Bjorge,S., Huy,R., Ly,S., Laurent,D., Hok,B., Roces,M.C., Ong,S., Char,M.C., Deubel,V., Tarantola,A. and Buchy,P.
EPI_ISL_17458027, EPI_ISL_17458028, EPI_ISL_17458029, EPI_ISL_17458030, EPI_ISL_17458031, EPI_ISL_17458032	Institut Pasteur du Laos	Institut Pasteur du Laos	Calvez,E. and Grandadam,M.
EPI_ISL_17458033, EPI_ISL_17458034, EPI_ISL_17458035, EPI_ISL_17458036, EPI_ISL_17458037	Institut Pasteur, Genotyping of Pathogens and Public Health	Institut Pasteur, Genotyping of Pathogens and Public Health	Grandadam,M., Diancourt,L., Caro,V. and Brey,P.
EPI_ISL_17458038	Institut Pasteur, Genotyping of Pathogens and Public Health	Institut Pasteur, Genotyping of Pathogens and Public Health	Dupont-Rouzeyrol,M., Caro,V., Guillaumot,L., Vazeille,M., D'Ortenzio,E., Thiberge,J.M., Baroux,N., Gourinat,A.C., Grandadam,M. and Failloux,A.B.
EPI_ISL_17458039, EPI_ISL_17458040, EPI_ISL_17458041, EPI_ISL_17458042, EPI_ISL_17458043, EPI_ISL_17458044, EPI_ISL_17458045, EPI_ISL_17458046, EPI_ISL_17458047, EPI_ISL_17458048, EPI_ISL_17458049, EPI_ISL_17458050, EPI_ISL_17458051, EPI_ISL_17458052, EPI_ISL_17458053, EPI_ISL_17458054, EPI_ISL_17458055, EPI_ISL_17458056, EPI_ISL_17458057, EPI_ISL_17458058, EPI_ISL_17458059, EPI_ISL_17458060, EPI_ISL_17458061, EPI_ISL_17458062, EPI_ISL_17458063, EPI_ISL_17458064, EPI_ISL_17458065, EPI_ISL_17458066, EPI_ISL_17458067			
see above	Institut Pasteur, Genotyping of Pathogens and Public Health	Institut Pasteur, Genotyping of Pathogens and Public Health	Grandadam,M., Diancourt,L., Caro,V. and Brey,P.
EPI_ISL_17458068, EPI_ISL_17458069	Institut Pasteur, Genotyping of Pathogens and Public Health	Institut Pasteur, Genotyping of Pathogens and Public Health	Grandadam,M., Caro,V., Plumet,S., Thiberge,J.M., Souares,Y., Failloux,A.B., Tolou,H.J., Budelot,M., Cosserat,D., Leparc-Goffart,I. and Despres,P.
EPI_ISL_17458070	Institut Pasteur, Genotyping of Pathogens and Public Health	Institut Pasteur, Genotyping of Pathogens and Public Health	Grandadam,M., Diancourt,L., Caro,V. and Brey,P.
EPI_ISL_17458071	Institut Pasteur, Genotyping of Pathogens and Public Health	Institut Pasteur, Genotyping of Pathogens and Public Health	Grandadam,M., Caro,V., Plumet,S., Thiberge,J.M., Souares,Y., Failloux,A.B., Tolou,H.J., Budelot,M., Cosserat,D., Leparc-Goffart,I. and Despres,P.
EPI_ISL_17458072, EPI_ISL_17458073	Institut Pasteur, Genotyping of Pathogens and Public Health	Institut Pasteur, Genotyping of Pathogens and Public Health	Couderc,T., Gangneux,N., Chretien,F., Caro,V., Le Luong,T., Ducloux,B., Tolou,H., Lecuit,M. and Grandadam,M.
EPI_ISL_17458074, EPI_ISL_17458075, EPI_ISL_17458076	Institut Pasteur, Genotyping of Pathogens and Public Health	Institut Pasteur, Genotyping of Pathogens and Public Health	Grandadam,M., Diancourt,L., Caro,V. and Brey,P.
EPI_ISL_17458077	Institut Pasteur, Genotyping of Pathogens and Public Health	Institut Pasteur, Genotyping of Pathogens and Public Health	Caro,V., Dupont-Rouzeyrol,M. and Grandadam,M.
EPI_ISL_17458078, EPI_ISL_17458079, EPI_ISL_17458080, EPI_ISL_17458081	Institut Pasteur, Genotyping of Pathogens and Public Health	Institut Pasteur, Genotyping of Pathogens and Public Health	Grandadam,M., Diancourt,L., Caro,V. and Brey,P.
EPI_ISL_17458082	Institut Pasteur, Genotyping of Pathogens and Public Health	Institut Pasteur, Genotyping of Pathogens and Public Health	Grandadam,M., Caro,V., Plumet,S., Thiberge,J.M., Souares,Y., Failloux,A.B., Tolou,H.J., Budelot,M., Cosserat,D., Leparc-Goffart,I. and Despres,P.
EPI_ISL_17458083	Institut Pasteur, Genotyping of Pathogens and Public Health	Institut Pasteur, Genotyping of Pathogens and Public Health	Grandadam,M., Diancourt,L., Caro,V. and Brey,P.
EPI_ISL_17458084, EPI_ISL_17458085, EPI_ISL_17458086, EPI_ISL_17458087, EPI_ISL_17458088, EPI_ISL_17458089, EPI_ISL_17458090, EPI_ISL_17458091, EPI_ISL_17458092, EPI_ISL_17458093, EPI_ISL_17458094, EPI_ISL_17458095, EPI_ISL_17458096, EPI_ISL_17458097, EPI_ISL_17458098, EPI_ISL_17458099, EPI_ISL_17458100, EPI_ISL_17458101, EPI_ISL_17458102, EPI_ISL_17458103			
see above	Institut Pasteur, Viral Populations and Pathogenesis Unit	Institut Pasteur, Viral Populations and Pathogenesis Unit	Stapleford,K.A., Moratorio,G., Henningsson,R., Chen,R., Matheus,S., Enfissi,A., Weissglas-Volkov,D., Isakov,O., Blanc,H., Mounce,B.C., Dupont-Rouzeyrol,M., Shomron,N., Weaver,S., Fontes,M., Rousset,D. and Vignuzzi,M.
EPI_ISL_17458104	Institut de Médecine Tropicale du Service de Santé des Armées	Institut de Médecine Tropicale du Service de Santé des Armées	Pastorino,B., Muyembe-Tamfum,J.J., Bessaud,M., Tock,F., Tolou,H., Durand,J.P. and Peyrefitte,C.N.
EPI_ISL_17458105	Institut de Médecine Tropicale du Service de Santé des Armées	Institut de Médecine Tropicale du Service de Santé des Armées	Bessaud,M., Peyrefitte,C.N., Pastorino,B.A., Tock,F., Merle,O., Colpart,J.J., Dehecq,J.S., Girod,R., Jaffar-Bandjee,M.C., Glass,P.J., Parker,M., Tolou,H.J. and Grandadam,M.
EPI_ISL_17458106	Institut de Médecine Tropicale du Service de Santé des Armées	Institut de Médecine Tropicale du Service de Santé des Armées	Peyrefitte,C.N., Bessaud,M., Pastorino,B.A., Gravier,P., Plumet,S., Merle,O.L., Moltini,I., Coppin,E., Tock,F., Daries,W., Ollivier,L., Pages,F., Martin,R., Boniface,F., Tolou,H.J. and Grandadam,M.
EPI_ISL_17458107, EPI_ISL_17458108	Institut de Médecine Tropicale du Service de Santé des Armées	Institut de Médecine Tropicale du Service de Santé des Armées	Pastorino,B., Muyembe-Tamfum,J.J., Bessaud,M., Tock,F., Tolou,H., Durand,J.P. and Peyrefitte,C.N.
EPI_ISL_17458109	Institut de Médecine Tropicale du Service de Santé des Armées	Institut de Médecine Tropicale du Service de Santé des Armées	Peyrefitte,C.N., Rousset,D., Pastorino,B.A., Pouillot,R., Bessaud,M., Tock,F., Mansaray,H., Merle,O.L., Pascual,A.M., Paupy,C., Vessiere,A., Imbert,P., Tchendjou,P., Durand,J.P., Tolou,H.J. and Grandadam,M.
EPI_ISL_17458110, EPI_ISL_17458111, EPI_ISL_17458112	Institut de Médecine Tropicale du Service de Santé des Armées	Institut de Médecine Tropicale du Service de Santé des Armées	Bessaud,M., Peyrefitte,C.N., Pastorino,B.A., Tock,F., Merle,O., Colpart,J.J., Dehecq,J.S., Girod,R., Jaffar-Bandjee,M.C., Glass,P.J., Parker,M., Tolou,H.J. and Grandadam,M.
EPI_ISL_17458113, EPI_ISL_17458114, EPI_ISL_17458115	Institut de Médecine Tropicale du Service de Santé des Armées	Institut de Médecine Tropicale du Service de Santé des Armées	Pastorino,B., Muyembe-Tamfum,J.J., Bessaud,M., Tock,F., Tolou,H., Durand,J.P. and Peyrefitte,C.N.
EPI_ISL_17458116	Institut de Médecine Tropicale du Service de Santé des Armées	Institut de Médecine Tropicale du Service de Santé des Armées	Peyrefitte,C.N., Bessaud,M., Pastorino,B.A., Gravier,P., Plumet,S., Merle,O.L., Moltini,I., Coppin,E., Tock,F., Daries,W., Ollivier,L., Pages,F., Martin,R., Boniface,F., Tolou,H.J. and Grandadam,M.
EPI_ISL_17458117	Institut de Médecine Tropicale du Service de Santé des Armées	Institut de Médecine Tropicale du Service de Santé des Armées	Bessaud,M., Peyrefitte,C.N., Pastorino,B.A., Tock,F., Merle,O., Colpart,J.J., Dehecq,J.S., Girod,R., Jaffar-Bandjee,M.C., Glass,P.J., Parker,M., Tolou,H.J. and Grandadam,M.
EPI_ISL_17458118, EPI_ISL_17458119	Institut de Médecine Tropicale du Service de Santé des Armées	Institut de Médecine Tropicale du Service de Santé des Armées	Pastorino,B., Muyembe-Tamfum,J.J., Bessaud,M., Tock,F., Tolou,H., Durand,J.P. and Peyrefitte,C.N.
EPI_ISL_17458120	Institut de Médecine Tropicale du Service de Santé des Armées	Institut de Médecine Tropicale du Service de Santé des Armées	Peyrefitte,C.N., Bessaud,M., Pastorino,B.A., Gravier,P., Plumet,S., Merle,O.L., Moltini,I., Coppin,E., Tock,F., Daries,W., Ollivier,L., Pages,F., Martin,R., Boniface,F., Tolou,H.J. and Grandadam,M.

EPI_ISL_17458121, EPI_ISL_17458122	Institut de Médecine Tropicale du Service de Santé des Armées	Institut de Médecine Tropicale du Service de Santé des Armées	Pastorino,B., Muyembe-Tamfum,J.J., Bessaud,M., Tock,F., Tolou,H., Durand,J.P. and Peyrefitte,C.N.
EPI_ISL_17458123	Institut de Médecine Tropicale du Service de Santé des Armées	Institut de Médecine Tropicale du Service de Santé des Armées	Pages,F., Peyrefitte,C.N., Mve,M.T., Jarjaval,F., Brisse,S., Iteman,I., Gravier,P., Nkoghe,D. and Grandadam,M.
EPI_ISL_17458124	Institute for Medical Research	Institute for Medical Research	Yusof,M.A., Lau,S.K., Nur Ismawati,I., Amal Nasir,M., Faudzi,Y., Wan Mansur,H., Hani,M.H. and Zainah,S.
EPI_ISL_17458125	Institute for Medical Research	Institute for Medical Research	Berendam,S.J., Thayan,R., Saat,Z., Kassim,F.M., Lau,S.K., Tengku Rashid,T.R. and Yusof,M.A.
EPI_ISL_17458126	Institute for Medical Research	Institute for Medical Research	Yusof,M.A., Sau Kuen,L., Adnan,N., Razak,N.I., Ahmad Zamri,L., Hulaimi,K.I. and Saat,Z.
EPI_ISL_17458127	Institute for Medical Research	Institute for Medical Research	Yusof,M.A., Lau,S.K., Nur Ismawati,I., Amal Nasir,M., Faudzi,Y., Wan Mansur,H., Hani,M.H. and Zainah,S.
EPI_ISL_17458128, EPI_ISL_17458129, EPI_ISL_17458130, EPI_ISL_17458131, EPI_ISL_17458132, EPI_ISL_17458133	Institute for Medical Research	Institute for Medical Research	Yusof,M.A., Sau Kuen,L., Adnan,N., Razak,N.I., Ahmad Zamri,L., Hulaimi,K.I. and Saat,Z.
EPI_ISL_17458134, EPI_ISL_17458135, EPI_ISL_17458136, EPI_ISL_17458137, EPI_ISL_17458138, EPI_ISL_17458139, EPI_ISL_17458140	Institute for Medical Research	Institute for Medical Research	Yusof,M.A., Lau,S.K., Nur Ismawati,I., Amal Nasir,M., Faudzi,Y., Wan Mansur,H., Hani,M.H. and Zainah,S.
EPI_ISL_17458141	Institute for Medical Research	Institute for Medical Research	Yusof,M.A., Sau Kuen,L., Adnan,N., Razak,N.I., Ahmad Zamri,L., Hulaimi,K.I. and Saat,Z.
EPI_ISL_17458142	Institute for Medical Research	Institute for Medical Research	Yusof,M.A., Lau,S.K., Nur Ismawati,I., Amal Nasir,M., Faudzi,Y., Wan Mansur,H., Hani,M.H. and Zainah,S.
EPI_ISL_17458143, EPI_ISL_17458144	Institute for Medical Research	Institute for Medical Research	Yusof,M.A., Sau Kuen,L., Adnan,N., Razak,N.I., Ahmad Zamri,L., Hulaimi,K.I. and Saat,Z.
EPI_ISL_17458145	Institute for Medical Research	Institute for Medical Research	Yusof,M.A., Lau,S.K., Nur Ismawati,I., Amal Nasir,M., Faudzi,Y., Wan Mansur,H., Hani,M.H. and Zainah,S.
EPI_ISL_17458146, EPI_ISL_17458147, EPI_ISL_17458148	Institute for Medical Research	Institute for Medical Research	Yusof,M.A., Sau Kuen,L., Adnan,N., Razak,N.I., Ahmad Zamri,L., Hulaimi,K.I. and Saat,Z.
EPI_ISL_17458149	Institute for Medical Research	Institute for Medical Research	Yusof,M.A., Lau,S.K., Nur Ismawati,I., Amal Nasir,M., Faudzi,Y., Wan Mansur,H., Hani,M.H. and Zainah,S.
EPI_ISL_17458150, EPI_ISL_17458151, EPI_ISL_17458152, EPI_ISL_17458153, EPI_ISL_17458154, EPI_ISL_17458155, EPI_ISL_17458156, EPI_ISL_17458157, EPI_ISL_17458158	Institute for Medical Research	Institute for Medical Research	Yusof,M.A., Sau Kuen,L., Adnan,N., Razak,N.I., Ahmad Zamri,L., Hulaimi,K.I. and Saat,Z.
EPI_ISL_17458159, EPI_ISL_17458160	Institute for Medical Research	Institute for Medical Research	Berendam,S.J., Thayan,R., Saat,Z., Kassim,F.M., Lau,S.K., Tengku Rashid,T.R. and Yusof,M.A.
EPI_ISL_17458161	Institute for Medical Research	Institute for Medical Research	Yusof,M.A., Sau Kuen,L., Adnan,N., Razak,N.I., Ahmad Zamri,L., Hulaimi,K.I. and Saat,Z.
EPI_ISL_17458162	Institute for Medical Research	Institute for Medical Research	Berendam,S.J., Thayan,R., Saat,Z., Kassim,F.M., Lau,S.K., Tengku Rashid,T.R. and Yusof,M.A.
EPI_ISL_17458163	Institute for Medical Research	Institute for Medical Research	Yusof,M.A., Lau,S.K., Nur Ismawati,I., Amal Nasir,M., Faudzi,Y., Wan Mansur,H., Hani,M.H. and Zainah,S.
EPI_ISL_17458164	Institute for Medical Research	Institute for Medical Research	Yusof,M.A., Sau Kuen,L., Adnan,N., Razak,N.I., Ahmad Zamri,L., Hulaimi,K.I. and Saat,Z.
EPI_ISL_17458165, EPI_ISL_17458166	Institute for Medical Research	Institute for Medical Research	Yusof,M.A., Lau,S.K., Nur Ismawati,I., Amal Nasir,M., Faudzi,Y., Wan Mansur,H., Hani,M.H. and Zainah,S.
EPI_ISL_17458167, EPI_ISL_17458168	Institute for Medical Research	Institute for Medical Research	Rozilawati,H., Mohd Masri,S., Nazni,W., Apandi,M., Zairi,J., Yahaya,M. and Lee,H.
EPI_ISL_17458169	Institute for Medical Research	Institute for Medical Research	Apandi,Y., Nazni,W., Noor Azleen,Z., Vythilingam,I., Noorazian,M., Azahari,A., Zainah,S. and Lee,H.
EPI_ISL_17458170	Institute for Medical Research	Institute for Medical Research	Rozilawati,H., Mohd Masri,S., Nazni,W., Apandi,M., Zairi,J., Yahaya,M. and Lee,H.
EPI_ISL_17458171, EPI_ISL_17458172	Institute for Medical Research	Institute for Medical Research	Suhana,O., Nazni,W.A., Apandi,Y., Farah,H., Zainah,S. and Sofian,A.
EPI_ISL_17458173	Institute for Medical Research	Institute for Medical Research	Apandi,Y., Nazni,W., Noor Azleen,Z., Vythilingam,I., Noorazian,M., Azahari,A., Zainah,S. and Lee,H.
EPI_ISL_17458174	Institute for Medical Research	Institute for Medical Research	Yusof,M.A., Sau Kuen,L., Adnan,N., Razak,N.I., Ahmad Zamri,L., Hulaimi,K.I. and Saat,Z.
EPI_ISL_17458175, EPI_ISL_17458176	Institute for Medical Research	Institute for Medical Research	Suhana,O., Nazni,W.A., Apandi,Y., Farah,H., Zainah,S. and Sofian,A.
EPI_ISL_17458177	Institute for Medical Research	Institute for Medical Research	Apandi,Y., Nazni,W., Noor Azleen,Z., Vythilingam,I., Noorazian,M., Azahari,A., Zainah,S. and Lee,H.
EPI_ISL_17458178, EPI_ISL_17458179	Institute for Medical Research	Institute for Medical Research	Yusof,M.A., Lau,S.K., Nur Ismawati,I., Amal Nasir,M., Faudzi,Y., Wan Mansur,H., Hani,M.H. and Zainah,S.
EPI_ISL_17458180	Institute for Medical Research	Institute for Medical Research	Yusof,M.A., Sau Kuen,L., Adnan,N., Razak,N.I., Ahmad Zamri,L., Hulaimi,K.I. and Saat,Z.
EPI_ISL_17458181, EPI_ISL_17458182	Institute for Medical Research	Institute for Medical Research	Yusof,M.A., Lau,S.K., Nur Ismawati,I., Amal Nasir,M., Faudzi,Y., Wan Mansur,H., Hani,M.H. and Zainah,S.
EPI_ISL_17458183	Institute for Medical Research	Institute for Medical Research	Apandi,Y., Nazni,W., Noor Azleen,Z., Vythilingam,I., Noorazian,M., Azahari,A., Zainah,S. and Lee,H.
EPI_ISL_17458184, EPI_ISL_17458185	Institute for Medical Research	Institute for Medical Research	Yusof,M.A., Sau Kuen,L., Adnan,N., Razak,N.I., Ahmad Zamri,L., Hulaimi,K.I. and Saat,Z.
EPI_ISL_17458186, EPI_ISL_17458187	Institute for Medical Research	Institute for Medical Research	Yusof,M.A., Lau,S.K., Nur Ismawati,I., Amal Nasir,M., Faudzi,Y., Wan Mansur,H., Hani,M.H. and Zainah,S.
EPI_ISL_17458188	Institute for Medical Research	Institute for Medical Research	Yusof,M.A., Sau Kuen,L., Adnan,N., Razak,N.I., Ahmad Zamri,L., Hulaimi,K.I. and Saat,Z.
EPI_ISL_17458189	Institute for Medical Research	Institute for Medical Research	Yusof,M.A., Lau,S.K., Nur Ismawati,I., Amal Nasir,M., Faudzi,Y., Wan Mansur,H., Hani,M.H. and Zainah,S.
EPI_ISL_17458190, EPI_ISL_17458191, EPI_ISL_17458192, EPI_ISL_17458193, EPI_ISL_17458194, EPI_ISL_17458195, EPI_ISL_17458196, EPI_ISL_17458197, EPI_ISL_17458198, EPI_ISL_17458199, EPI_ISL_17458200, EPI_ISL_17458201, EPI_ISL_17458202, EPI_ISL_17458203			
see above	Institute for Medical Research	Institute for Medical Research	Yusof,M.A., Sau Kuen,L., Adnan,N., Razak,N.I., Ahmad Zamri,L., Hulaimi,K.I. and Saat,Z.
EPI_ISL_17458204	Institute for Medical Research	Institute for Medical Research	Yusof,M.A., Lau,S.K., Nur Ismawati,I., Amal Nasir,M., Faudzi,Y., Wan Mansur,H., Hani,M.H. and Zainah,S.
EPI_ISL_17458205, EPI_ISL_17458206, EPI_ISL_17458207, EPI_ISL_17458208, EPI_ISL_17458209, EPI_ISL_17458210	Institute for Medical Research	Institute for Medical Research	Yusof,M.A., Sau Kuen,L., Adnan,N., Razak,N.I., Ahmad Zamri,L., Hulaimi,K.I. and Saat,Z.
EPI_ISL_17458211, EPI_ISL_17458212, EPI_ISL_17458213, EPI_ISL_17458214, EPI_ISL_17458215, EPI_ISL_17458216, EPI_ISL_17458217, EPI_ISL_17458218, EPI_ISL_17458219, EPI_ISL_17458220, EPI_ISL_17458221, EPI_ISL_17458222, EPI_ISL_17458223, EPI_ISL_17458224, EPI_ISL_17458225, EPI_ISL_17458226, EPI_ISL_17458227, EPI_ISL_17458228, EPI_ISL_17458229, EPI_ISL_17458230			
see above	Institute for Vector and Reservoir Control Research and Development	Institute for Vector and Reservoir Control Research and Development	Anggraeni,Y.M., Garjito,T.A., Prihatin,M.T., Handayani,S.W., Negari,K.S., Yanti,A.O., Hidajat,M.C., Prastowo,D., Satoto,T.B.T., Manguin,S., Gavotte,L. and Frutos,R.
EPI_ISL_17458231, EPI_ISL_17458232, EPI_ISL_17458233, EPI_ISL_17458234	Institute for Viral Disease Control and Prevention	Institute for Viral Disease Control and Prevention	Zheng,K., Li,J., Zhang,Q., Liang,M., Li,C., Lin,M., Huang,J., Li,H., Xiang,D., Wang,N., Hong,Y., Huang,L., Li,X., Pan,D., Song,W., Dai,J., Guo,B. and Li,D.
EPI_ISL_17458235	Institute of Disease Prevention and Control of Chinese People's Liberation Army	Institute of Disease Prevention and Control of Chinese People's Liberation Army	Li,P.
EPI_ISL_17458236, EPI_ISL_17458237, EPI_ISL_17458238, EPI_ISL_17458239, EPI_ISL_17458240, EPI_ISL_17458241, EPI_ISL_17458242, EPI_ISL_17458243, EPI_ISL_17458244, EPI_ISL_17458245, EPI_ISL_17458246, EPI_ISL_17458247			
see above	Institute of Life Sciences	Institute of Life Sciences	Saswat,T., Kumar,A., Kumar,S., Mamidi,P., Muduli,S., Debata,N.K., Pal,N.S., Pratheek,B.M., Chattopadhyay,S. and Chattopadhyay,S.
EPI_ISL_17458248, EPI_ISL_17458249	Institute of Life Sciences, Department of Infectious Disease Biology	Institute of Life Sciences, Department of Infectious Disease Biology	Saswat,T., Sahoo,N., Muduli,S., Debata,N.K., Chattopadhyay,S. and Chattopadhyay,S.
EPI_ISL_17458250, EPI_ISL_17458251, EPI_ISL_17458252, EPI_ISL_17458253, EPI_ISL_17458254, EPI_ISL_17458255, EPI_ISL_17458256, EPI_ISL_17458257, EPI_ISL_17458258	Institute of Liver and Biliary Sciences	Institute of Liver and Biliary Sciences	Choudhary,M.C., Paul,M. and Gupta,E.
EPI_ISL_17458259	Institute of Microbiology and Immunology, Faculty of Medicine, University of Ljubljana, Laboratory for Diagnostics of Zoonoses and WHO Centre	Institute of Microbiology and Immunology, Faculty of Medicine, University of Ljubljana, Laboratory for Diagnostics of Zoonoses and WHO Centre	Zakotnik,S., Korva,M., Knap,N., Zorec,T.M. and Avsic Zupanc,T.
EPI_ISL_17458260, EPI_ISL_17458261	Institute of Microbiology of Bundeswehr	Institute of Microbiology of Bundeswehr	Pfeffer,M., Zoeller,G., Essbauer,S., Tomaso,H., Berens-Riha,N., Loescher,T. and Dobler,G.
EPI_ISL_17458262	Institute of Tropical Medicine Antwerp	Institute of Tropical Medicine Antwerp	Selhorst,P., Makiala-Mandanda,S., Smet,B., Marien,J., Anthony,C., Binene-Mbuka,G., Weggheleire,A., Ilombe,G., Kinganda-Lusamaki,E., Pukuta-Simbu,E., Lubula,L., Mbala-Kingebeni,P., Nkuba-Ndaye,A., Vogt,F., Watsenga,F., Bortel,W.V., Vanlerberghe,V., Arien,K.K. and Ahuka-Mundeke,S.
EPI_ISL_17458263, EPI_ISL_17458264	Institute of Tropical Medicine Antwerp	Institute of Tropical Medicine Antwerp	Tuekprakhon,A., Nakayama,E.E., Bartholomeeusen,K., Puiprom,O., Sasaki,T., Huits,R., Luplertlop,N., Kosoltanapiwat,N., Maneekan,P., Arien,K.K., Shioda,T. and Leaungwutiwong,P.
EPI_ISL_17458265	Institute of Tropical Medicine Antwerp	Institute of Tropical Medicine Antwerp	Selhorst,P., Makiala-Mandanda,S., Smet,B., Marien,J., Anthony,C., Binene-Mbuka,G., Weggheleire,A., Ilombe,G., Kinganda-Lusamaki,E., Pukuta-Simbu,E., Lubula,L., Mbala-Kingebeni,P., Nkuba-Ndaye,A., Vogt,F., Watsenga,F., Bortel,W.V., Vanlerberghe,V., Arien,K.K. and Ahuka-Mundeke,S.
EPI_ISL_17458266, EPI_ISL_17458267, EPI_ISL_17458268, EPI_ISL_17458269	Institute of Tropical Medicine Antwerp	Institute of Tropical Medicine Antwerp	Tuekprakhon,A., Nakayama,E.E., Bartholomeeusen,K., Puiprom,O., Sasaki,T., Huits,R., Luplertlop,N., Kosoltanapiwat,N., Maneekan,P., Arien,K.K., Shioda,T. and Leaungwutiwong,P.
EPI_ISL_17458270, EPI_ISL_17458271	Institute of Tropical Medicine Antwerp	Institute of Tropical Medicine Antwerp	Selhorst,P., Makiala-Mandanda,S., Smet,B., Marien,J., Anthony,C., Binene-Mbuka,G., Weggheleire,A., Ilombe,G., Kinganda-Lusamaki,E., Pukuta-Simbu,E., Lubula,L., Mbala-Kingebeni,P., Nkuba-Ndaye,A., Vogt,F., Watsenga,F., Bortel,W.V., Vanlerberghe,V., Arien,K.K. and Ahuka-Mundeke,S.
EPI_ISL_17458272	Institute of Tropical Medicine Antwerp	Institute of Tropical Medicine Antwerp	Tuekprakhon,A., Nakayama,E.E., Bartholomeeusen,K., Puiprom,O., Sasaki,T., Huits,R., Luplertlop,N., Kosoltanapiwat,N., Maneekan,P., Arien,K.K., Shioda,T. and Leaungwutiwong,P.
EPI_ISL_17458273	Institute of Tropical Medicine Antwerp	Institute of Tropical Medicine Antwerp	Selhorst,P., Makiala-Mandanda,S., Smet,B., Marien,J., Anthony,C., Binene-Mbuka,G., Weggheleire,A., Ilombe,G., Kinganda-Lusamaki,E., Pukuta-Simbu,E., Lubula,L., Mbala-Kingebeni,P., Nkuba-Ndaye,A., Vogt,F., Watsenga,F., Bortel,W.V., Vanlerberghe,V., Arien,K.K. and Ahuka-Mundeke,S.
EPI_ISL_17458274, EPI_ISL_17458275	Institute of Tropical Medicine Antwerp	Institute of Tropical Medicine Antwerp	Tuekprakhon,A., Nakayama,E.E., Bartholomeeusen,K., Puiprom,O., Sasaki,T., Huits,R., Luplertlop,N., Kosoltanapiwat,N., Maneekan,P., Arien,K.K., Shioda,T. and Leaungwutiwong,P.
EPI_ISL_17458276, EPI_ISL_17458277	Institute of Tropical Medicine Antwerp	Institute of Tropical Medicine Antwerp	Selhorst,P., Makiala-Mandanda,S., Smet,B., Marien,J., Anthony,C., Binene-Mbuka,G., Weggheleire,A., Ilombe,G., Kinganda-Lusamaki,E., Pukuta-Simbu,E., Lubula,L., Mbala-Kingebeni,P., Nkuba-Ndaye,A., Vogt,F., Watsenga,F., Bortel,W.V., Vanlerberghe,V., Arien,K.K. and Ahuka-Mundeke,S.
EPI_ISL_17458278	Institute of Tropical Medicine Antwerp	Institute of Tropical Medicine Antwerp	Tuekprakhon,A., Nakayama,E.E., Bartholomeeusen,K., Puiprom,O., Sasaki,T., Huits,R., Luplertlop,N., Kosoltanapiwat,N., Maneekan,P., Arien,K.K., Shioda,T. and Leaungwutiwong,P.

EPI_ISL_17458279, EPI_ISL_17458280, EPI_ISL_17458281, EPI_ISL_17458282, EPI_ISL_17458283, EPI_ISL_17458284, EPI_ISL_17458285, EPI_ISL_17458286	Institute of Tropical Medicine Antwerp	Institute of Tropical Medicine Antwerp	Selhorst,P., Makiala-Mandanda,S., Smet,B., Marien,J., Anthony,C., Binene-Mbuka,G., Weggheleire,A., Ilombe,G., Kinganda-Lusamaki,E., Pukuta-Simbu,E., Lubula,L., Mbala-Kingebeni,P., Nkuba-Ndaye,A., Vogt,F., Watsenga,F., Bortel,W.V., Vanlerberghe,V., Arien,K.K. and Ahuka-Mundeke,S.
EPI_ISL_17458287	Institute of Tropical Medicine Antwerp	Institute of Tropical Medicine Antwerp	Tuekprakhon,A., Nakayama,E.E., Bartholomeeusen,K., Puiprom,O., Sasaki,T., Huits,R., Luplertlop,N., Kosoltanapiwat,N., Maneekan,P., Arien,K.K., Shioda,T. and Leaungwutiwong,P.
EPI_ISL_17458288	Institute of Tropical Medicine Antwerp	Institute of Tropical Medicine Antwerp	Selhorst,P., Makiala-Mandanda,S., Smet,B., Marien,J., Anthony,C., Binene-Mbuka,G., Weggheleire,A., Ilombe,G., Kinganda-Lusamaki,E., Pukuta-Simbu,E., Lubula,L., Mbala-Kingebeni,P., Nkuba-Ndaye,A., Vogt,F., Watsenga,F., Bortel,W.V., Vanlerberghe,V., Arien,K.K. and Ahuka-Mundeke,S.
EPI_ISL_17458289, EPI_ISL_17458290, EPI_ISL_17458291, EPI_ISL_17458292	Institute of Virology, Wenzhou University	Institute of Virology, Wenzhou University	Xiao,P.
EPI_ISL_17458293	Institute of Virology, Wenzhou University	Institute of Virology, Wenzhou University	Feng,G., Zhang,J., Zhang,Y., Li,C., Zhang,D., Li,Y., Zhou,H., Li,N. and Xiao,P.
EPI_ISL_17458294, EPI_ISL_17458295, EPI_ISL_17458296, EPI_ISL_17458297, EPI_ISL_17458298, EPI_ISL_17458299, EPI_ISL_17458300, EPI_ISL_17458301, EPI_ISL_17458302, EPI_ISL_17458303, EPI_ISL_17458304, EPI_ISL_17458305, EPI_ISL_17458306			
see above	Institute of Virology, Wenzhou University	Institute of Virology, Wenzhou University	Xiao,P.
EPI_ISL_17458307, EPI_ISL_17458308, EPI_ISL_17458309, EPI_ISL_17458310, EPI_ISL_17458311, EPI_ISL_17458312	Institute of Virology, Wenzhou University	Institute of Virology, Wenzhou University	Feng,G., Zhang,J., Zhang,Y., Li,C., Zhang,D., Li,Y., Zhou,H., Li,N. and Xiao,P.
EPI_ISL_17458313, EPI_ISL_17458314	Institute of Virology, Wenzhou University	Institute of Virology, Wenzhou University	Xiao,P.
EPI_ISL_17458315	Institute of Virology, Wenzhou University	Institute of Virology, Wenzhou University	Feng,G., Zhang,J., Zhang,Y., Li,C., Zhang,D., Li,Y., Zhou,H., Li,N. and Xiao,P.
EPI_ISL_17458316, EPI_ISL_17458317, EPI_ISL_17458318, EPI_ISL_17458319, EPI_ISL_17458320	Institute of Virology, Wenzhou University	Institute of Virology, Wenzhou University	Xiao,P.
EPI_ISL_17458321	Institute of Virology, Wenzhou University	Institute of Virology, Wenzhou University	Feng,G., Zhang,J., Zhang,Y., Li,C., Zhang,D., Li,Y., Zhou,H., Li,N. and Xiao,P.
EPI_ISL_17458322, EPI_ISL_17458323, EPI_ISL_17458324	Institute of Virology, Wenzhou University	Institute of Virology, Wenzhou University	Xiao,P.
EPI_ISL_17458325, EPI_ISL_17458326, EPI_ISL_17458327, EPI_ISL_17458328, EPI_ISL_17458329, EPI_ISL_17458330, EPI_ISL_17458331, EPI_ISL_17458332, EPI_ISL_17458333, EPI_ISL_17458334, EPI_ISL_17458335, EPI_ISL_17458336, EPI_ISL_17458337, EPI_ISL_17458338, EPI_ISL_17458339, EPI_ISL_17458340, EPI_ISL_17458341, EPI_ISL_17458342, EPI_ISL_17458343, EPI_ISL_17458344, EPI_ISL_17458345, EPI_ISL_17458346, EPI_ISL_17458347, EPI_ISL_17458348, EPI_ISL_17458349, EPI_ISL_17458350, EPI_ISL_17458351, EPI_ISL_17458352, EPI_ISL_17458353, EPI_ISL_17458354, EPI_ISL_17458355, EPI_ISL_17458356, EPI_ISL_17458357, EPI_ISL_17458358, EPI_ISL_17458359, EPI_ISL_17458360, EPI_ISL_17458361, EPI_ISL_17458362, EPI_ISL_17458363, EPI_ISL_17458364, EPI_ISL_17458365, EPI_ISL_17458366, EPI_ISL_17458367, EPI_ISL_17458368, EPI_ISL_17458369, EPI_ISL_17458370, EPI_ISL_17458371, EPI_ISL_17458372, EPI_ISL_17458373, EPI_ISL_17458374, EPI_ISL_17458375, EPI_ISL_17458376, EPI_ISL_17458377			
see above	Instituto Adolfo Lutz, Laboratorio Estrategico	Instituto Adolfo Lutz, Laboratorio Estrategico	Maeda,A.Y., Nogueira,J.S., Campos,K.R., Camargo,C.H., da Silva Vasami,F.G., Arvigo,A.P.B., Santos,M.B.N., Abbud,A. and Sacchi,C.T.
EPI_ISL_17458378	Instituto Nacional de Investigación en Salud Pública	Instituto Nacional de Investigación en Salud Pública	Cevallos,V., Ponce,P., Waggoner,J.J., Pinsky,B.A., Coloma,J., Quiroga,C., Morales,D. and Cardenas,M.J.
EPI_ISL_17458379, EPI_ISL_17458380	Instituto de Biofisica Carlos Chagas Filho, Universidade Federal do Rio de Janeiro	Instituto de Biofisica Carlos Chagas Filho, Universidade Federal do Rio de Janeiro	Cunha,M.S., Cruz,N.V.G., Schnellrath,L.C., Medaglia,M.L.G., Casotto,M.E., Albano,R.M., Costa,L.J. and Damaso,C.R.
EPI_ISL_17458381, EPI_ISL_17458382, EPI_ISL_17458383, EPI_ISL_17458384, EPI_ISL_17458385, EPI_ISL_17458386, EPI_ISL_17458387, EPI_ISL_17458388, EPI_ISL_17458389, EPI_ISL_17458390, EPI_ISL_17458391			
see above	Instituto de Ciencias Biologicas e da Saude - ICBS, Universidade Federal de Alagoas - UFAL	Instituto de Ciencias Biologicas e da Saude - ICBS, Universidade Federal de Alagoas - UFAL	Tanabe,E.Ld.L., Tanabe,I.S.B., Santos,E.Cd., Marques,J.Pd.S., Borges,A.A., Lima,M.Cd., Anderson,L. and Bassi,E.J.
EPI_ISL_17458392	Instituto de Diagnostico y Referencia Epidemiologicos	Instituto de Diagnostico y Referencia Epidemiologicos	Torres-Longoria,B., Fragoso-Fonseca,D.E., Nunez-Leon,A., de la Luz Torres,M., Vazquez-Pichardo,M., Escobar-Escamilla,N., Wong-Arambula,C., Ramirez-Gonzalez,J.E., Mendez-Tenorio,A., Castro-Mussot,M.E., Moreno-Altamirano,M.M.B., Membrillo-Hernandez,J., Lopez-Martinez,I. and Diaz-Quinonez,J.A.
EPI_ISL_17458393	Instituto de Diagnostico y Referencia Epidemiologicos	Instituto de Diagnostico y Referencia Epidemiologicos	Diaz-Quinonez,J.A., Ortiz-Alcantara,J., Fragoso-Fonseca,D.E., Garces-Ayala,F., Escobar-Escamilla,N., Vazquez-Pichardo,M., Nunez-Leon,A., Torres-Rodriguez Mde,L., Torres-Longoria,B., Lopez-Martinez,I., Ruiz-Matus,C., Kuri-Morales,P. and Ramirez-Gonzalez,J.E.
EPI_ISL_17458394	Instituto de Diagnostico y Referencia Epidemiologicos	Instituto de Diagnostico y Referencia Epidemiologicos	Vazquez-Pichardo,M., Nunez-Leon,A., Torres-Rodriguez,M.L., Escobar-Escamilla,N., Fragoso-Fonseca,D.E., Ramirez-Gonzalez,J.E., Torres-Longoria,B., Lopez-Martinez,I., Ruiz-Matus,C., Kuri-Morales,P. and Diaz-Quinonez,J.A.
EPI_ISL_17458395, EPI_ISL_17458396, EPI_ISL_17458397, EPI_ISL_17458398, EPI_ISL_17458399, EPI_ISL_17458400, EPI_ISL_17458401, EPI_ISL_17458402	Instituto de Diagnostico y Referencia Epidemiologicos	Instituto de Diagnostico y Referencia Epidemiologicos	Torres-Longoria,B., Fragoso-Fonseca,D.E., Nunez-Leon,A., de la Luz Torres,M., Vazquez-Pichardo,M., Escobar-Escamilla,N., Wong-Arambula,C., Ramirez-Gonzalez,J.E., Mendez-Tenorio,A., Castro-Mussot,M.E., Moreno-Altamirano,M.M.B., Membrillo-Hernandez,J., Lopez-Martinez,I. and Diaz-Quinonez,J.A.
EPI_ISL_17458403	Instituto de Diagnostico y Referencia Epidemiologicos	Instituto de Diagnostico y Referencia Epidemiologicos	Vazquez-Pichardo,M., Nunez-Leon,A., Torres-Rodriguez,M.L., Escobar-Escamilla,N., Fragoso-Fonseca,D.E., Ramirez Gonzalez,J., Torres-Longoria,B., Lopez-Martinez,I., Ruiz-Matus,C., Kuri-Morales,P. and Diaz-Quinonez,J.A.
EPI_ISL_17458404, EPI_ISL_17458405, EPI_ISL_17458406, EPI_ISL_17458407	Instituto de Diagnostico y Referencia Epidemiologicos	Instituto de Diagnostico y Referencia Epidemiologicos	Torres-Longoria,B., Fragoso-Fonseca,D.E., Nunez-Leon,A., de la Luz Torres,M., Vazquez-Pichardo,M., Escobar-Escamilla,N., Wong-Arambula,C., Ramirez-Gonzalez,J.E., Mendez-Tenorio,A., Castro-Mussot,M.E., Moreno-Altamirano,M.M.B., Membrillo-Hernandez,J., Lopez-Martinez,I. and Diaz-Quinonez,J.A.
EPI_ISL_17458408	Instituto de Diagnostico y Referencia Epidemiologicos	Instituto de Diagnostico y Referencia Epidemiologicos	Vazquez-Pichardo,M., Nunez-Leon,A., Torres-Rodriguez,M., Escobar-Escamilla,N., Fragoso-Fonseca,D., Ramirez-Gonzalez,J.E., Torres-Longoria,B., Lopez-Martinez,I., Ruiz-Matus,C., Kuri-Morales,P. and Diaz-Quinonez,J.A.
EPI_ISL_17458409, EPI_ISL_17458410, EPI_ISL_17458411, EPI_ISL_17458412	Instituto de Diagnostico y Referencia Epidemiologicos	Instituto de Diagnostico y Referencia Epidemiologicos	Torres-Longoria,B., Fragoso-Fonseca,D.E., Nunez-Leon,A., de la Luz Torres,M., Vazquez-Pichardo,M., Escobar-Escamilla,N., Wong-Arambula,C., Ramirez-Gonzalez,J.E., Mendez-Tenorio,A., Castro-Mussot,M.E., Moreno-Altamirano,M.M.B., Membrillo-Hernandez,J., Lopez-Martinez,I. and Diaz-Quinonez,J.A.
EPI_ISL_17458413	Instituto de Diagnostico y Referencia Epidemiologicos	Instituto de Diagnostico y Referencia Epidemiologicos	Diaz-Quinonez,J.A., Ortiz-Alcantara,J., Fragoso-Fonseca,D.E., Garces-Ayala,F., Escobar-Escamilla,N., Vazquez-Pichardo,M., Nunez-Leon,A., Torres-Rodriguez Mde,L., Torres-Longoria,B., Lopez-Martinez,I., Ruiz-Matus,C., Kuri-Morales,P. and Ramirez-Gonzalez,J.E.
EPI_ISL_17458414	Instituto de Higiene e Medicina Tropical	Instituto de Higiene e Medicina Tropical	Parreira,R., Centeno-Lima,S., Lopes,A., Portugal-Calisto,D., Constantino,A. and Nina,J.
EPI_ISL_17458415	Instituto de Salud Carlos III	Instituto de Salud Carlos III	Requena-Mendez,A., Garcia,C., Aldasoro,E., Vicente,J.A., Martinez,M.J., Perez-Molina,J.A., Calvo-Cano,A., Franco,L., Parron,I., Molina,A., Ruiz,M., Alvarez,J., Sanchez-Seco,M.P. and Gascon,J.
EPI_ISL_17458416	Instituto de Salud Carlos III, Diagnostic Microbiology Service	Instituto de Salud Carlos III, Diagnostic Microbiology Service	Collao,X., Cano,J., Negredo,A.I., Tenorio,A., Benito,A. and Sanchez-Seco,M.P.
EPI_ISL_17458417	Instituto de Salud Carlos III, Diagnostic Microbiology Service	Instituto de Salud Carlos III, Diagnostic Microbiology Service	Collao,X., Negredo,A.I., Cano,J., Tenorio,A., Ory,F., Benito,A., Masia,M. and Sanchez-Seco,M.P.
EPI_ISL_17458418, EPI_ISL_17458419	Instituto de Salud Carlos III, Diagnostic Microbiology Service	Instituto de Salud Carlos III, Diagnostic Microbiology Service	Collao,X., Cano,J., Negredo,A.I., Tenorio,A., Benito,A. and Sanchez-Seco,M.P.
EPI_ISL_17458420	Instituto de Salud Carlos III, Diagnostic Microbiology Service	Instituto de Salud Carlos III, Diagnostic Microbiology Service	Sanchez-Seco,M.P., Rosario,D., Quiroz,E., Guzman,G. and Tenorio,A.
EPI_ISL_17458421, EPI_ISL_17458422, EPI_ISL_17458423, EPI_ISL_17458424	Instituto de Salud Carlos III, Diagnostic Microbiology Service	Instituto de Salud Carlos III, Diagnostic Microbiology Service	Collao,X., Cano,J., Negredo,A.I., Tenorio,A., Benito,A. and Sanchez-Seco,M.P.
EPI_ISL_17458425, EPI_ISL_17458426, EPI_ISL_17458427	Instituto de Salud Carlos III, Diagnostic Microbiology Service	Instituto de Salud Carlos III, Diagnostic Microbiology Service	Collao,X., Negredo,A.I., Cano,J., Tenorio,A., Ory,F., Benito,A., Masia,M. and Sanchez-Seco,M.P.
EPI_ISL_17458428, EPI_ISL_17458429, EPI_ISL_17458430, EPI_ISL_17458431, EPI_ISL_17458432	Integral Molecular	Integral Molecular	DORANZ,B., SIMMONS,G., KAHLE,K., MATTIA,K., FONG,R.H. and BANIK,B.S.
EPI_ISL_17458433	Interactive Research School for Health Affairs	Interactive Research School for Health Affairs	Shrivastava,S.
EPI_ISL_17458434, EPI_ISL_17458435, EPI_ISL_17458436, EPI_ISL_17458437, EPI_ISL_17458438	International Centre for Diarrhoeal Disease Research	International Centre for Diarrhoeal Disease Research	Mazumder,R., Ahmed,D., Chowdhury,A.H. and Ahmed,N.
EPI_ISL_17458439	International Centre for Diarrhoeal Disease Research	International Centre for Diarrhoeal Disease Research	Mazumder,R. and Ahmed,D.
EPI_ISL_17458440, EPI_ISL_17458441	International Centre for Diarrhoeal Disease Research	International Centre for Diarrhoeal Disease Research	Mazumder,R., Ahmed,D., Chowdhury,A.H. and Ahmed,N.
EPI_ISL_17458442	International Centre for Diarrhoeal Disease Research	International Centre for Diarrhoeal Disease Research	Mazumder,R. and Ahmed,D.
EPI_ISL_17458443	International Centre for Diarrhoeal Disease Research	International Centre for Diarrhoeal Disease Research	Mazumder,R., Ahmed,D., Chowdhury,A.H. and Ahmed,N.
EPI_ISL_17458444, EPI_ISL_17458445, EPI_ISL_17458446	International Centre for Diarrhoeal Disease Research	International Centre for Diarrhoeal Disease Research	Mazumder,R. and Ahmed,D.
EPI_ISL_17458447, EPI_ISL_17458448, EPI_ISL_17458449, EPI_ISL_17458450, EPI_ISL_17458451, EPI_ISL_17458452, EPI_ISL_17458453	International Centre for Genetic Engineering & Biotechnology	International Centre for Genetic Engineering & Biotechnology	Sunil,S., Jain,S., Shrinet,J., Sharma,A., Rana,V., Bhatnagar,R.K., Gupta,B., Gaind,R. and Deb,M.
EPI_ISL_17458454	International Centre for Genetic Engineering & Biotechnology	International Centre for Genetic Engineering & Biotechnology	Sunil,S., Jain,J., Kushwah,R., Singh,S.S., Sharma,A., Adak,T., Singh,O.P., Bhatnagar,R. and Subbarao,S.
EPI_ISL_17458455, EPI_ISL_17458456, EPI_ISL_17458457, EPI_ISL_17458458, EPI_ISL_17458459, EPI_ISL_17458460, EPI_ISL_17458461, EPI_ISL_17458462, EPI_ISL_17458463, EPI_ISL_17458464	International Centre for Genetic Engineering & Biotechnology	International Centre for Genetic Engineering & Biotechnology	Sunil,S., Jain,S., Shrinet,J., Sharma,A., Rana,V., Bhatnagar,R.K., Gupta,B., Gaind,R. and Deb,M.
EPI_ISL_17458465, EPI_ISL_17458466, EPI_ISL_17458467, EPI_ISL_17458468, EPI_ISL_17458469, EPI_ISL_17458470,	International Centre for Genetic Engineering & Biotechnology	International Centre for Genetic Engineering & Biotechnology	Jain,J., Mathur,K., Shrinet,J., Bhatnagar,R.K. and Sunil,S.

EPI_ISL_17458471, EPI_ISL_17458472, EPI_ISL_17458473, EPI_ISL_17458474			
EPI_ISL_17458475, EPI_ISL_17458476	International Centre for Genetic Engineering & Biotechnology	International Centre for Genetic Engineering & Biotechnology	Sunil,S., Jain,S., Shrinet,J., Sharma,A., Rana,V., Bhatnagar,R.K., Gupta,B., Gaind,R. and Deb,M.
EPI_ISL_17458477, EPI_ISL_17458478	International Centre for Genetic Engineering & Biotechnology	International Centre for Genetic Engineering & Biotechnology	Sunil,S., Jain,J., Kushwah,R., Singh,S.S., Sharma,A., Adak,T., Singh,O.P., Bhatnagar,R. and Subbarao,S.
EPI_ISL_17458479	International Centre for Genetic Engineering & Biotechnology	International Centre for Genetic Engineering & Biotechnology	Sunil,S., Jain,S., Shrinet,J., Sharma,A., Rana,V., Bhatnagar,R.K., Gupta,B., Gaind,R. and Deb,M.
EPI_ISL_17458480	International Centre for Genetic Engineering & Biotechnology	International Centre for Genetic Engineering & Biotechnology	Sunil,S., Jain,J., Kushwah,R., Singh,S.S., Sharma,A., Adak,T., Singh,O.P., Bhatnagar,R. and Subbarao,S.
EPI_ISL_17458481	International Centre for Genetic Engineering & Biotechnology	International Centre for Genetic Engineering & Biotechnology	Sunil,S., Jain,S., Shrinet,J., Sharma,A., Rana,V., Bhatnagar,R.K., Gupta,B., Gaind,R. and Deb,M.
EPI_ISL_17458482	International Centre for Genetic Engineering & Biotechnology	International Centre for Genetic Engineering & Biotechnology	Jain,J., Mathur,K., Shrinet,J., Bhatnagar,R.K. and Sunil,S.
EPI_ISL_17458483, EPI_ISL_17458484, EPI_ISL_17458485, EPI_ISL_17458486, EPI_ISL_17458487, EPI_ISL_17458488, EPI_ISL_17458489, EPI_ISL_17458490, EPI_ISL_17458491, EPI_ISL_17458492, EPI_ISL_17458493, EPI_ISL_17458494, EPI_ISL_17458495, EPI_ISL_17458496, EPI_ISL_17458497			
see above	International Centre for Genetic Engineering & Biotechnology	International Centre for Genetic Engineering & Biotechnology	Sunil,S., Jain,S., Shrinet,J., Sharma,A., Rana,V., Bhatnagar,R.K., Gupta,B., Gaind,R. and Deb,M.
EPI_ISL_17458498, EPI_ISL_17458499, EPI_ISL_17458500, EPI_ISL_17458501, EPI_ISL_17458502, EPI_ISL_17458503, EPI_ISL_17458504, EPI_ISL_17458505, EPI_ISL_17458506, EPI_ISL_17458507, EPI_ISL_17458508, EPI_ISL_17458509, EPI_ISL_17458510, EPI_ISL_17458511, EPI_ISL_17458512, EPI_ISL_17458513, EPI_ISL_17458514, EPI_ISL_17458515			
see above	International Centre for Genetic Engineering and Biotechnology	International Centre for Genetic Engineering and Biotechnology	Sunil,S.
EPI_ISL_17458516	International Centre for Genetic Engineering and Biotechnology	International Centre for Genetic Engineering and Biotechnology	Chaudhary,S., Jain,J., Kumar,R., Shrinet,J., Weaver,S.C., Auguste,A.J. and Sunil,S.
EPI_ISL_17458517, EPI_ISL_17458518, EPI_ISL_17458519, EPI_ISL_17458520	International Centre for Genetic Engineering and Biotechnology	International Centre for Genetic Engineering and Biotechnology	Sunil,S.
EPI_ISL_17458521	International Centre for Genetic Engineering and Biotechnology	International Centre for Genetic Engineering and Biotechnology	Chaudhary,S., Jain,J., Kumar,R., Shrinet,J., Weaver,S.C., Auguste,A.J. and Sunil,S.
EPI_ISL_17458522, EPI_ISL_17458523, EPI_ISL_17458524, EPI_ISL_17458525, EPI_ISL_17458526	International Centre for Genetic Engineering and Biotechnology	International Centre for Genetic Engineering and Biotechnology	Jain,J., Kaur,N., Haller,S.L., Singh,G.P., Kumar,A., Rossi,S.L., Narayanan,V., Kumar,D., Gaind,R., Weaver,S.C., Auguste,A.J. and Sunil,S.
EPI_ISL_17458527	International Centre for Genetic Engineering and Biotechnology	International Centre for Genetic Engineering and Biotechnology	Sunil,S.
EPI_ISL_17458528, EPI_ISL_17458529, EPI_ISL_17458530, EPI_ISL_17458531, EPI_ISL_17458532, EPI_ISL_17458533, EPI_ISL_17458534, EPI_ISL_17458535, EPI_ISL_17458536	International Centre for Genetic Engineering and Biotechnology	International Centre for Genetic Engineering and Biotechnology	Jain,J., Kaur,N., Haller,S.L., Singh,G.P., Kumar,A., Rossi,S.L., Narayanan,V., Kumar,D., Gaind,R., Weaver,S.C., Auguste,A.J. and Sunil,S.
EPI_ISL_17458537	International Centre for Genetic Engineering and Biotechnology	International Centre for Genetic Engineering and Biotechnology	Sunil,S.
EPI_ISL_17458538	International Livestock Research Institute	International Livestock Research Institute	Wasonga,C., Inoue,S., Rumberia,C., Michuki,G., Kimotho,J., Ongus,J.R., Sang,R. and Musila,L.
EPI_ISL_17458539, EPI_ISL_17458540	International Livestock Research Institute,	International Livestock Research Institute,	Wasonga,C., Inoue,S., Rumberia,C., Michuki,G., Kimotho,J., Ongus,J.R., Sang,R. and Musila,L.
EPI_ISL_17458541, EPI_ISL_17458542, EPI_ISL_17458543	Iowa State University	Iowa State University	Cigarroa-Toledo,N., Blitvich,B.J., Cetina-Trejo,R.C., Talavera-Aguilar,L.G., Baak-Baak,C.M., Torres-Chable,O.M., Hamid,M.N., Friedberg,I., Gonzalez-Martinez,P., Alonzo-Salomon,G., Rosado-Paredes,E.P., Rivero-Cardenas,N., Reyes-Solis,G.C., Farfan-Ale,J.A., Garcia-Rejon,J.E. and Machain-Williams,C.
EPI_ISL_17458544, EPI_ISL_17458545	Iowa State University	Iowa State University	Nunez-Avellaneda,D., Villagomez,F.R., Villegas-Pineda,J.C., Barrios-Palacios,J., Salazar,M.I., Machain-Williams,C. and Blitvich,B.J.
EPI_ISL_17458546, EPI_ISL_17458547, EPI_ISL_17458548	Iowa State University	Iowa State University	Cigarroa-Toledo,N., Blitvich,B.J., Cetina-Trejo,R.C., Talavera-Aguilar,L.G., Baak-Baak,C.M., Torres-Chable,O.M., Hamid,M.N., Friedberg,I., Gonzalez-Martinez,P., Alonzo-Salomon,G., Rosado-Paredes,E.P., Rivero-Cardenas,N., Reyes-Solis,G.C., Farfan-Ale,J.A., Garcia-Rejon,J.E. and Machain-Williams,C.
EPI_ISL_17458549	Iowa State University	Iowa State University	Nunez-Avellaneda,D., Villagomez,F.R., Villegas-Pineda,J.C., Barrios-Palacios,J., Salazar,M.I., Machain-Williams,C. and Blitvich,B.J.
EPI_ISL_17458550, EPI_ISL_17458551, EPI_ISL_17458552, EPI_ISL_17458553, EPI_ISL_17458554, EPI_ISL_17458555, EPI_ISL_17458556	Iowa State University	Iowa State University	Cigarroa-Toledo,N., Blitvich,B.J., Cetina-Trejo,R.C., Talavera-Aguilar,L.G., Baak-Baak,C.M., Torres-Chable,O.M., Hamid,M.N., Friedberg,I., Gonzalez-Martinez,P., Alonzo-Salomon,G., Rosado-Paredes,E.P., Rivero-Cardenas,N., Reyes-Solis,G.C., Farfan-Ale,J.A., Garcia-Rejon,J.E. and Machain-Williams,C.
EPI_ISL_17458557, EPI_ISL_17458558	Iowa State University	Iowa State University	Nunez-Avellaneda,D., Villagomez,F.R., Villegas-Pineda,J.C., Barrios-Palacios,J., Salazar,M.I., Machain-Williams,C. and Blitvich,B.J.
EPI_ISL_17458559	Iowa State University	Iowa State University	Cigarroa-Toledo,N., Blitvich,B.J., Cetina-Trejo,R.C., Talavera-Aguilar,L.G., Baak-Baak,C.M., Torres-Chable,O.M., Hamid,M.N., Friedberg,I., Gonzalez-Martinez,P., Alonzo-Salomon,G., Rosado-Paredes,E.P., Rivero-Cardenas,N., Reyes-Solis,G.C., Farfan-Ale,J.A., Garcia-Rejon,J.E. and Machain-Williams,C.
EPI_ISL_17458560	Iowa State University	Iowa State University	Nunez-Avellaneda,D., Villagomez,F.R., Villegas-Pineda,J.C., Barrios-Palacios,J., Salazar,M.I., Machain-Williams,C. and Blitvich,B.J.
EPI_ISL_17458561	Istituto Zooprofilattico Sperimentale Della Lombardia E Dell'emilia Romagna	Istituto Zooprofilattico Sperimentale Della Lombardia E Dell'emilia Romagna	Fallacara,F. and Bonilauri,P.
EPI_ISL_17458562, EPI_ISL_17458563	J. Craig Venter Institute	J. Craig Venter Institute	Shabman,R., Pickett,B., Das,S.R., Fedorova,N., Puri,V., Shrivastava,S., Amedeo,P., Durbin,A., Balmaseda,A.L., Guillermina,K., Gresh,L., Gordon,A. and Harris,E.
EPI_ISL_17458564, EPI_ISL_17458565	J. Craig Venter Institute	J. Craig Venter Institute	Shabman,R., Pickett,B., Das,S.R., Fedorova,N., Puri,V., Shrivastava,S., Amedeo,P., Durbin,A., Gordon,A. and Harris,E.
EPI_ISL_17458566, EPI_ISL_17458567	J. Craig Venter Institute	J. Craig Venter Institute	Shabman,R., Pickett,B., Das,S.R., Fedorova,N., Puri,V., Shrivastava,S., Amedeo,P., Durbin,A., Balmaseda,A.L., Guillermina,K., Gresh,L., Gordon,A. and Harris,E.
EPI_ISL_17458568	J. Craig Venter Institute	J. Craig Venter Institute	Shabman,R., Pickett,B., Das,S.R., Fedorova,N., Puri,V., Shrivastava,S., Amedeo,P., Durbin,A. and Heberlein-Larson,L.
EPI_ISL_17458569, EPI_ISL_17458570, EPI_ISL_17458571, EPI_ISL_17458572	J. Craig Venter Institute	J. Craig Venter Institute	Shabman,R., Pickett,B., Das,S.R., Fedorova,N., Puri,V., Shrivastava,S., Amedeo,P., Durbin,A., Balmaseda,A.L., Guillermina,K., Gresh,L., Gordon,A. and Harris,E.
EPI_ISL_17458573	J. Craig Venter Institute	J. Craig Venter Institute	Shabman,R., Pickett,B., Das,S.R., Fedorova,N., Puri,V., Shrivastava,S., Amedeo,P., Durbin,A. and Heberlein-Larson,L.
EPI_ISL_17458574	J. Craig Venter Institute	J. Craig Venter Institute	Shabman,R., Pickett,B., Das,S.R., Fedorova,N., Puri,V., Shrivastava,S., Amedeo,P., Durbin,A., Balmaseda,A.L., Guillermina,K., Gresh,L., Gordon,A. and Harris,E.
EPI_ISL_17458575	J. Craig Venter Institute	J. Craig Venter Institute	Shabman,R., Pickett,B., Das,S.R., Fedorova,N., Puri,V., Shrivastava,S., Amedeo,P., Durbin,A., Gordon,A. and Harris,E.
EPI_ISL_17458576, EPI_ISL_17458577, EPI_ISL_17458578, EPI_ISL_17458579, EPI_ISL_17458580, EPI_ISL_17458581, EPI_ISL_17458582, EPI_ISL_17458583, EPI_ISL_17458584	J. Craig Venter Institute	J. Craig Venter Institute	Shabman,R., Pickett,B., Das,S.R., Fedorova,N., Puri,V., Shrivastava,S., Amedeo,P., Durbin,A., Balmaseda,A.L., Guillermina,K., Gresh,L., Gordon,A. and Harris,E.
EPI_ISL_17458585	J. Craig Venter Institute	J. Craig Venter Institute	Shabman,R., Pickett,B., Das,S.R., Fedorova,N., Puri,V., Shrivastava,S., Amedeo,P., Durbin,A. and Heberlein-Larson,L.
EPI_ISL_17458586	J. Craig Venter Institute	J. Craig Venter Institute	Shabman,R., Pickett,B., Das,S.R., Fedorova,N., Puri,V., Shrivastava,S., Amedeo,P., Durbin,A., Balmaseda,A.L., Guillermina,K., Gresh,L., Gordon,A. and Harris,E.
EPI_ISL_17458587, EPI_ISL_17458588	J. Craig Venter Institute	J. Craig Venter Institute	Shabman,R., Pickett,B., Das,S.R., Fedorova,N., Puri,V., Shrivastava,S., Amedeo,P., Durbin,A. and Heberlein-Larson,L.
EPI_ISL_17458589	J. Craig Venter Institute	J. Craig Venter Institute	Shabman,R., Pickett,B., Das,S.R., Fedorova,N., Puri,V., Shrivastava,S., Amedeo,P., Durbin,A., Gordon,A. and Harris,E.
EPI_ISL_17458590, EPI_ISL_17458591, EPI_ISL_17458592	J. Craig Venter Institute	J. Craig Venter Institute	Shabman,R., Pickett,B., Das,S.R., Fedorova,N., Puri,V., Shrivastava,S., Amedeo,P., Durbin,A. and Heberlein-Larson,L.
EPI_ISL_17458593, EPI_ISL_17458594, EPI_ISL_17458595, EPI_ISL_17458596	J. Craig Venter Institute	J. Craig Venter Institute	Shabman,R., Pickett,B., Das,S.R., Fedorova,N., Puri,V., Shrivastava,S., Amedeo,P., Durbin,A., Balmaseda,A.L., Guillermina,K., Gresh,L., Gordon,A. and Harris,E.
EPI_ISL_17458597	J. Craig Venter Institute	J. Craig Venter Institute	Shabman,R., Pickett,B., Das,S.R., Fedorova,N., Puri,V., Shrivastava,S., Amedeo,P., Durbin,A., Gordon,A. and Harris,E.
EPI_ISL_17458598, EPI_ISL_17458599	J. Craig Venter Institute	J. Craig Venter Institute	Shabman,R., Pickett,B., Das,S.R., Fedorova,N., Puri,V., Shrivastava,S., Amedeo,P., Durbin,A., Balmaseda,A.L., Guillermina,K., Gresh,L., Gordon,A. and Harris,E.
EPI_ISL_17458600	J. Craig Venter Institute	J. Craig Venter Institute	Shabman,R., Pickett,B., Das,S.R., Fedorova,N., Puri,V., Shrivastava,S., Amedeo,P., Durbin,A. and Heberlein-Larson,L.
EPI_ISL_17458601	J. Craig Venter Institute	J. Craig Venter Institute	Shabman,R., Pickett,B., Das,S.R., Fedorova,N., Puri,V., Shrivastava,S., Amedeo,P., Durbin,A., Balmaseda,A.L., Guillermina,K., Gresh,L., Gordon,A. and Harris,E.
EPI_ISL_17458602	J. Craig Venter Institute	J. Craig Venter Institute	Shabman,R., Pickett,B., Das,S.R., Fedorova,N., Puri,V., Shrivastava,S., Amedeo,P., Durbin,A. and Heberlein-Larson,L.
EPI_ISL_17458603	J. Craig Venter Institute	J. Craig Venter Institute	Shabman,R., Pickett,B., Das,S.R., Fedorova,N., Puri,V., Shrivastava,S., Amedeo,P., Durbin,A., Gordon,A. and Harris,E.
EPI_ISL_17458604, EPI_ISL_17458605, EPI_ISL_17458606, EPI_ISL_17458607	J. Craig Venter Institute	J. Craig Venter Institute	Shabman,R., Pickett,B., Das,S.R., Fedorova,N., Puri,V., Shrivastava,S., Amedeo,P., Durbin,A. and Heberlein-Larson,L.
EPI_ISL_17458608	J. Craig Venter Institute	J. Craig Venter Institute	Shabman,R., Pickett,B., Das,S.R., Fedorova,N., Puri,V., Shrivastava,S., Amedeo,P., Durbin,A., Balmaseda,A.L., Guillermina,K., Gresh,L., Gordon,A. and Harris,E.
EPI_ISL_17458609, EPI_ISL_17458610, EPI_ISL_17458611	J. Craig Venter Institute	J. Craig Venter Institute	Shabman,R., Pickett,B., Das,S.R., Fedorova,N., Puri,V., Shrivastava,S., Amedeo,P., Durbin,A. and Heberlein-Larson,L.
EPI_ISL_17458612, EPI_ISL_17458613	J. Craig Venter Institute	J. Craig Venter Institute	Shabman,R., Pickett,B., Das,S.R., Fedorova,N., Puri,V., Shrivastava,S., Amedeo,P., Durbin,A., Balmaseda,A.L., Guillermina,K., Gresh,L., Gordon,A. and Harris,E.
EPI_ISL_17458614	J. Craig Venter Institute	J. Craig Venter Institute	Shabman,R., Pickett,B., Das,S.R., Fedorova,N., Puri,V., Shrivastava,S., Amedeo,P., Durbin,A. and Heberlein-Larson,L.
EPI_ISL_17458615	J. Craig Venter Institute	J. Craig Venter Institute	Das,S.R., Shabman,R., Halpin,R.A., Shilts,M., Akopov,A., Fedorova,N., Puri,V., Stockwell,T., Amedeo,P., Katzel,D., Schobel,S., Shrivastava,S. and Weaver,S.C.

EPI_ISL_17458616	J. Craig Venter Institute	J. Craig Venter Institute	Shabman,R., Pickett,B., Das,S.R., Fedorova,N., Puri,V., Shrivastava,S., Amedeo,P., Durbin,A., Balmaseda,A.L., Guillermina,K., Gresh,L., Gordon,A. and Harris,E.
EPI_ISL_17458617, EPI_ISL_17458618	J. Craig Venter Institute	J. Craig Venter Institute	Shabman,R., Pickett,B., Das,S.R., Fedorova,N., Puri,V., Shrivastava,S., Amedeo,P., Durbin,A., Gordon,A. and Harris,E.
EPI_ISL_17458619, EPI_ISL_17458620, EPI_ISL_17458621, EPI_ISL_17458622, EPI_ISL_17458623, EPI_ISL_17458624, EPI_ISL_17458625, EPI_ISL_17458626, EPI_ISL_17458627	J. Craig Venter Institute	J. Craig Venter Institute	Shabman,R., Pickett,B., Das,S.R., Fedorova,N., Puri,V., Shrivastava,S., Amedeo,P., Durbin,A., Balmaseda,A.L., Guillermina,K., Gresh,L., Gordon,A. and Harris,E.
EPI_ISL_17458628, EPI_ISL_17458629	J. Craig Venter Institute	J. Craig Venter Institute	Shabman,R., Pickett,B., Das,S.R., Fedorova,N., Puri,V., Shrivastava,S., Amedeo,P., Durbin,A. and Heberlein-Larson,L.
EPI_ISL_17458630, EPI_ISL_17458631, EPI_ISL_17458632, EPI_ISL_17458633, EPI_ISL_17458634	J. Craig Venter Institute	J. Craig Venter Institute	Shabman,R., Pickett,B., Das,S.R., Fedorova,N., Puri,V., Shrivastava,S., Amedeo,P., Durbin,A., Balmaseda,A.L., Guillermina,K., Gresh,L., Gordon,A. and Harris,E.
EPI_ISL_17458635	J. Craig Venter Institute	J. Craig Venter Institute	Shabman,R., Pickett,B., Das,S.R., Fedorova,N., Puri,V., Shrivastava,S., Amedeo,P., Durbin,A., Gordon,A. and Harris,E.
EPI_ISL_17458636, EPI_ISL_17458637	J. Craig Venter Institute	J. Craig Venter Institute	Shabman,R., Pickett,B., Das,S.R., Fedorova,N., Puri,V., Shrivastava,S., Amedeo,P., Durbin,A., Balmaseda,A.L., Guillermina,K., Gresh,L., Gordon,A. and Harris,E.
EPI_ISL_17458638	J. Craig Venter Institute	J. Craig Venter Institute	Shabman,R., Pickett,B., Das,S.R., Fedorova,N., Puri,V., Shrivastava,S., Amedeo,P., Durbin,A. and Heberlein-Larson,L.
EPI_ISL_17458639	J. Craig Venter Institute	J. Craig Venter Institute	Shabman,R., Pickett,B., Das,S.R., Fedorova,N., Puri,V., Shrivastava,S., Amedeo,P., Durbin,A., Balmaseda,A.L., Guillermina,K., Gresh,L., Gordon,A. and Harris,E.
EPI_ISL_17458640	J. Craig Venter Institute	J. Craig Venter Institute	Shabman,R., Pickett,B., Das,S.R., Fedorova,N., Puri,V., Shrivastava,S., Amedeo,P., Durbin,A. and Heberlein-Larson,L.
EPI_ISL_17458641	J. Craig Venter Institute	J. Craig Venter Institute	Shabman,R., Pickett,B., Das,S.R., Fedorova,N., Puri,V., Shrivastava,S., Amedeo,P., Durbin,A., Balmaseda,A.L., Guillermina,K., Gresh,L., Gordon,A. and Harris,E.
EPI_ISL_17458642	J. Craig Venter Institute	J. Craig Venter Institute	Shabman,R., Pickett,B., Das,S.R., Fedorova,N., Puri,V., Shrivastava,S., Amedeo,P., Durbin,A., Gordon,A. and Harris,E.
EPI_ISL_17458643	J. Craig Venter Institute	J. Craig Venter Institute	Shabman,R., Pickett,B., Das,S.R., Fedorova,N., Puri,V., Shrivastava,S., Amedeo,P., Durbin,A. and Heberlein-Larson,L.
EPI_ISL_17458644	J. Craig Venter Institute	J. Craig Venter Institute	Shabman,R., Pickett,B., Das,S.R., Fedorova,N., Puri,V., Shrivastava,S., Amedeo,P., Durbin,A., Balmaseda,A.L., Guillermina,K., Gresh,L., Gordon,A. and Harris,E.
EPI_ISL_17458645	J. Craig Venter Institute	J. Craig Venter Institute	Shabman,R., Pickett,B., Das,S.R., Fedorova,N., Puri,V., Shrivastava,S., Amedeo,P., Durbin,A. and Heberlein-Larson,L.
EPI_ISL_17458646, EPI_ISL_17458647, EPI_ISL_17458648, EPI_ISL_17458649, EPI_ISL_17458650	J. Craig Venter Institute	J. Craig Venter Institute	Shabman,R., Pickett,B., Das,S.R., Fedorova,N., Puri,V., Shrivastava,S., Amedeo,P., Durbin,A., Balmaseda,A.L., Guillermina,K., Gresh,L., Gordon,A. and Harris,E.
EPI_ISL_17458651	J. Craig Venter Institute	J. Craig Venter Institute	Shabman,R., Pickett,B., Das,S.R., Fedorova,N., Puri,V., Shrivastava,S., Amedeo,P., Durbin,A. and Heberlein-Larson,L.
EPI_ISL_17458652, EPI_ISL_17458653, EPI_ISL_17458654	J. Craig Venter Institute	J. Craig Venter Institute	Shabman,R., Pickett,B., Das,S.R., Fedorova,N., Puri,V., Shrivastava,S., Amedeo,P., Durbin,A., Balmaseda,A.L., Guillermina,K., Gresh,L., Gordon,A. and Harris,E.
EPI_ISL_17458655	J. Craig Venter Institute	J. Craig Venter Institute	Shabman,R., Pickett,B., Das,S.R., Fedorova,N., Puri,V., Shrivastava,S., Amedeo,P., Durbin,A., Gordon,A. and Harris,E.
EPI_ISL_17458656	J. Craig Venter Institute	J. Craig Venter Institute	Shabman,R., Pickett,B., Das,S.R., Fedorova,N., Puri,V., Shrivastava,S., Amedeo,P., Durbin,A. and Heberlein-Larson,L.
EPI_ISL_17458657, EPI_ISL_17458658	J. Craig Venter Institute	J. Craig Venter Institute	Shabman,R., Pickett,B., Das,S.R., Fedorova,N., Puri,V., Shrivastava,S., Amedeo,P., Durbin,A., Balmaseda,A.L., Guillermina,K., Gresh,L., Gordon,A. and Harris,E.
EPI_ISL_17458659	J. Craig Venter Institute	J. Craig Venter Institute	Shabman,R., Pickett,B., Das,S.R., Fedorova,N., Puri,V., Shrivastava,S., Amedeo,P., Durbin,A. and Heberlein-Larson,L.
EPI_ISL_17458660, EPI_ISL_17458661, EPI_ISL_17458662	J. Craig Venter Institute	J. Craig Venter Institute	Shabman,R., Pickett,B., Das,S.R., Fedorova,N., Puri,V., Shrivastava,S., Amedeo,P., Durbin,A., Balmaseda,A.L., Guillermina,K., Gresh,L., Gordon,A. and Harris,E.
EPI_ISL_17458663	J. Craig Venter Institute	J. Craig Venter Institute	Shabman,R., Pickett,B., Das,S.R., Fedorova,N., Puri,V., Shrivastava,S., Amedeo,P., Durbin,A., Gordon,A. and Harris,E.
EPI_ISL_17458664, EPI_ISL_17458665	J. Craig Venter Institute	J. Craig Venter Institute	Shabman,R., Pickett,B., Das,S.R., Fedorova,N., Puri,V., Shrivastava,S., Amedeo,P., Durbin,A., Balmaseda,A.L., Guillermina,K., Gresh,L., Gordon,A. and Harris,E.
EPI_ISL_17458666	J. Craig Venter Institute	J. Craig Venter Institute	Shabman,R., Pickett,B., Das,S.R., Fedorova,N., Puri,V., Shrivastava,S., Amedeo,P., Durbin,A., Gordon,A. and Harris,E.
EPI_ISL_17458667	J. Craig Venter Institute	J. Craig Venter Institute	Shabman,R., Pickett,B., Das,S.R., Fedorova,N., Puri,V., Shrivastava,S., Amedeo,P., Durbin,A. and Heberlein-Larson,L.
EPI_ISL_17458668	J. Craig Venter Institute	J. Craig Venter Institute	Shabman,R., Pickett,B., Das,S.R., Fedorova,N., Puri,V., Shrivastava,S., Amedeo,P., Durbin,A., Balmaseda,A.L., Guillermina,K., Gresh,L., Gordon,A. and Harris,E.
EPI_ISL_17458669	J. Craig Venter Institute	J. Craig Venter Institute	Shabman,R., Pickett,B., Das,S.R., Fedorova,N., Puri,V., Shrivastava,S., Amedeo,P., Durbin,A. and Heberlein-Larson,L.
EPI_ISL_17458670, EPI_ISL_17458671, EPI_ISL_17458672, EPI_ISL_17458673, EPI_ISL_17458674, EPI_ISL_17458675, EPI_ISL_17458676, EPI_ISL_17458677	J. Craig Venter Institute	J. Craig Venter Institute	Shabman,R., Pickett,B., Das,S.R., Fedorova,N., Puri,V., Shrivastava,S., Amedeo,P., Durbin,A., Balmaseda,A.L., Guillermina,K., Gresh,L., Gordon,A. and Harris,E.
EPI_ISL_17458678	J. Craig Venter Institute	J. Craig Venter Institute	Shabman,R., Pickett,B., Das,S.R., Fedorova,N., Puri,V., Shrivastava,S., Amedeo,P., Durbin,A., Gordon,A. and Harris,E.
EPI_ISL_17458679, EPI_ISL_17458680	J. Craig Venter Institute	J. Craig Venter Institute	Shabman,R., Pickett,B., Das,S.R., Fedorova,N., Puri,V., Shrivastava,S., Amedeo,P., Durbin,A. and Heberlein-Larson,L.
EPI_ISL_17458681, EPI_ISL_17458682, EPI_ISL_17458683, EPI_ISL_17458684, EPI_ISL_17458685, EPI_ISL_17458686, EPI_ISL_17458687, EPI_ISL_17458688, EPI_ISL_17458689, EPI_ISL_17458690	J. Craig Venter Institute	J. Craig Venter Institute	Shabman,R., Pickett,B.E., Das,S.R., Fedorova,N., Shrivastava,S., Puri,V., Tan,Y., Hu,L., Amedeo,P., Jones,S. and Kramer,L.
EPI_ISL_17458691	J. Craig Venter Institute	J. Craig Venter Institute	Shabman,R., Pickett,B., Das,S.R., Fedorova,N., Puri,V., Shrivastava,S., Amedeo,P., Durbin,A., Balmaseda,A.L., Guillermina,K., Gresh,L., Gordon,A. and Harris,E.
EPI_ISL_17458692	J. Craig Venter Institute	J. Craig Venter Institute	Shabman,R., Pickett,B., Das,S.R., Fedorova,N., Puri,V., Shrivastava,S., Amedeo,P., Durbin,A. and Heberlein-Larson,L.
EPI_ISL_17458693	J. Craig Venter Institute	J. Craig Venter Institute	Shabman,R., Pickett,B., Das,S.R., Fedorova,N., Puri,V., Shrivastava,S., Amedeo,P., Durbin,A., Gordon,A. and Harris,E.
EPI_ISL_17458694	J. Craig Venter Institute	J. Craig Venter Institute	Shabman,R., Pickett,B., Das,S.R., Fedorova,N., Puri,V., Shrivastava,S., Amedeo,P., Durbin,A., Balmaseda,A.L., Guillermina,K., Gresh,L., Gordon,A. and Harris,E.
EPI_ISL_17458695	J. Craig Venter Institute	J. Craig Venter Institute	Shabman,R., Pickett,B., Das,S.R., Fedorova,N., Puri,V., Shrivastava,S., Amedeo,P., Durbin,A., Gordon,A. and Harris,E.
EPI_ISL_17458696	J. Craig Venter Institute	J. Craig Venter Institute	Das,S.R., Shabman,R., Halpin,R.A., Shilts,M., Akopov,A., Fedorova,N., Puri,V., Stockwell,T., Amedeo,P., Katzel,D., Schobel,S., Shrivastava,S. and Weaver,S.C.
EPI_ISL_17458697	J. Craig Venter Institute	J. Craig Venter Institute	Shabman,R., Pickett,B., Das,S.R., Fedorova,N., Puri,V., Shrivastava,S., Amedeo,P., Durbin,A. and Heberlein-Larson,L.
EPI_ISL_17458698	J. Craig Venter Institute	J. Craig Venter Institute	Shabman,R., Pickett,B., Das,S.R., Fedorova,N., Puri,V., Shrivastava,S., Amedeo,P., Durbin,A., Balmaseda,A.L., Guillermina,K., Gresh,L., Gordon,A. and Harris,E.
EPI_ISL_17458699	J. Craig Venter Institute	J. Craig Venter Institute	Shabman,R., Pickett,B., Das,S.R., Fedorova,N., Puri,V., Shrivastava,S., Amedeo,P., Durbin,A., Gordon,A. and Harris,E.
EPI_ISL_17458700, EPI_ISL_17458701, EPI_ISL_17458702, EPI_ISL_17458703, EPI_ISL_17458704	J. Craig Venter Institute	J. Craig Venter Institute	Shabman,R., Pickett,B., Das,S.R., Fedorova,N., Puri,V., Shrivastava,S., Amedeo,P., Durbin,A., Balmaseda,A.L., Guillermina,K., Gresh,L., Gordon,A. and Harris,E.
EPI_ISL_17458705	J. Craig Venter Institute	J. Craig Venter Institute	Shabman,R., Pickett,B., Das,S.R., Fedorova,N., Puri,V., Shrivastava,S., Amedeo,P., Durbin,A., Gordon,A. and Harris,E.
EPI_ISL_17458706, EPI_ISL_17458707, EPI_ISL_17458708, EPI_ISL_17458709, EPI_ISL_17458710, EPI_ISL_17458711, EPI_ISL_17458712, EPI_ISL_17458713, EPI_ISL_17458714, EPI_ISL_17458715	J. Craig Venter Institute	J. Craig Venter Institute	Shabman,R., Pickett,B., Das,S.R., Fedorova,N., Puri,V., Shrivastava,S., Amedeo,P., Durbin,A., Balmaseda,A.L., Guillermina,K., Gresh,L., Gordon,A. and Harris,E.
EPI_ISL_17458716	J. Craig Venter Institute	J. Craig Venter Institute	Shabman,R., Pickett,B., Das,S.R., Fedorova,N., Puri,V., Shrivastava,S., Amedeo,P., Durbin,A., Gordon,A. and Harris,E.
EPI_ISL_17458717, EPI_ISL_17458718, EPI_ISL_17458719, EPI_ISL_17458720, EPI_ISL_17458721, EPI_ISL_17458722, EPI_ISL_17458723, EPI_ISL_17458724	J. Craig Venter Institute	J. Craig Venter Institute	Shabman,R., Pickett,B., Das,S.R., Fedorova,N., Puri,V., Shrivastava,S., Amedeo,P., Durbin,A. and Heberlein-Larson,L.
EPI_ISL_17458725	J. Craig Venter Institute	J. Craig Venter Institute	Shabman,R., Pickett,B., Das,S.R., Fedorova,N., Puri,V., Shrivastava,S., Amedeo,P., Durbin,A., Balmaseda,A.L., Guillermina,K., Gresh,L., Gordon,A. and Harris,E.
EPI_ISL_17458726, EPI_ISL_17458727, EPI_ISL_17458728, EPI_ISL_17458729, EPI_ISL_17458730, EPI_ISL_17458731, EPI_ISL_17458732, EPI_ISL_17458733	J. Craig Venter Institute	J. Craig Venter Institute	Shabman,R., Pickett,B., Das,S.R., Fedorova,N., Puri,V., Shrivastava,S., Amedeo,P., Durbin,A. and Heberlein-Larson,L.
EPI_ISL_17458734	J. Craig Venter Institute	J. Craig Venter Institute	Das,S.R., Shabman,R., Halpin,R.A., Shilts,M., Akopov,A., Fedorova,N., Puri,V., Stockwell,T., Amedeo,P., Katzel,D., Schobel,S., Shrivastava,S. and Weaver,S.C.
EPI_ISL_17458735, EPI_ISL_17458736, EPI_ISL_17458737, EPI_ISL_17458738, EPI_ISL_17458739, EPI_ISL_17458740	J. Craig Venter Institute	J. Craig Venter Institute	Shabman,R., Pickett,B., Das,S.R., Fedorova,N., Puri,V., Shrivastava,S., Amedeo,P., Durbin,A. and Heberlein-Larson,L.
EPI_ISL_17458741, EPI_ISL_17458742	J. Craig Venter Institute	J. Craig Venter Institute	Shabman,R., Pickett,B., Das,S.R., Fedorova,N., Puri,V., Shrivastava,S., Amedeo,P., Durbin,A., Balmaseda,A.L., Guillermina,K., Gresh,L., Gordon,A. and Harris,E.
EPI_ISL_17458743	J. Craig Venter Institute	J. Craig Venter Institute	Shabman,R., Pickett,B., Das,S.R., Fedorova,N., Puri,V., Shrivastava,S., Amedeo,P., Durbin,A., Gordon,A. and Harris,E.
EPI_ISL_17458744, EPI_ISL_17458745, EPI_ISL_17458746, EPI_ISL_17458747, EPI_ISL_17458748	J. Craig Venter Institute	J. Craig Venter Institute	Shabman,R., Pickett,B., Das,S.R., Fedorova,N., Puri,V., Shrivastava,S., Amedeo,P., Durbin,A. and Heberlein-Larson,L.
EPI_ISL_17458749	J. Craig Venter Institute	J. Craig Venter Institute	Shabman,R., Pickett,B., Das,S.R., Fedorova,N., Puri,V., Shrivastava,S., Amedeo,P., Durbin,A., Balmaseda,A.L., Guillermina,K., Gresh,L., Gordon,A. and Harris,E.
EPI_ISL_17458750	J. Craig Venter Institute	J. Craig Venter Institute	Shabman,R., Pickett,B., Das,S.R., Fedorova,N., Puri,V., Shrivastava,S., Amedeo,P., Durbin,A. and Heberlein-Larson,L.
EPI_ISL_17458751, EPI_ISL_17458752	J. Craig Venter Institute	J. Craig Venter Institute	Das,S.R., Shabman,R., Halpin,R.A., Shilts,M., Akopov,A., Fedorova,N., Puri,V., Stockwell,T., Amedeo,P., Katzel,D., Schobel,S., Shrivastava,S. and Weaver,S.C.
EPI_ISL_17458753	J. Craig Venter Institute	J. Craig Venter Institute	Shabman,R., Pickett,B., Das,S.R., Fedorova,N., Puri,V., Shrivastava,S., Amedeo,P., Durbin,A. and Heberlein-Larson,L.
EPI_ISL_17458754, EPI_ISL_17458755, EPI_ISL_17458756, EPI_ISL_17458757, EPI_ISL_17458758	J. Craig Venter Institute	J. Craig Venter Institute	Das,S.R., Shabman,R., Halpin,R.A., Shilts,M., Akopov,A., Fedorova,N., Puri,V., Stockwell,T., Amedeo,P., Katzel,D., Schobel,S., Shrivastava,S. and Weaver,S.C.
EPI_ISL_17458759, EPI_ISL_17458760	J. Craig Venter Institute	J. Craig Venter Institute	Shabman,R., Pickett,B., Das,S.R., Fedorova,N., Puri,V., Shrivastava,S., Amedeo,P., Durbin,A. and Heberlein-Larson,L.
EPI_ISL_17458761	J. Craig Venter Institute	J. Craig Venter Institute	Das,S.R., Shabman,R., Halpin,R.A., Shilts,M., Akopov,A., Fedorova,N., Puri,V., Stockwell,T., Amedeo,P., Katzel,D., Schobel,S., Shrivastava,S. and Weaver,S.C.
EPI_ISL_17458762, EPI_ISL_17458763, EPI_ISL_17458764	J. Craig Venter Institute	J. Craig Venter Institute	Shabman,R., Pickett,B., Das,S.R., Fedorova,N., Puri,V., Shrivastava,S., Amedeo,P., Durbin,A. and Heberlein-Larson,L.
EPI_ISL_17458765	J. Craig Venter Institute	J. Craig Venter Institute	Das,S.R., Shabman,R., Halpin,R.A., Shilts,M., Akopov,A., Fedorova,N., Puri,V., Stockwell,T., Amedeo,P., Katzel,D., Schobel,S., Shrivastava,S. and Weaver,S.C.

EPI_ISL_17458766	J. Craig Venter Institute	J. Craig Venter Institute	Shabman,R., Pickett,B., Das,S.R., Fedorova,N., Puri,V., Shrivastava,S., Amedeo,P., Durbin,A. and Heberlein-Larson,L.
EPI_ISL_17458767, EPI_ISL_17458768, EPI_ISL_17458769, EPI_ISL_17458770, EPI_ISL_17458771, EPI_ISL_17458772, EPI_ISL_17458773, EPI_ISL_17458774, EPI_ISL_17458775, EPI_ISL_17458776, EPI_ISL_17458777, EPI_ISL_17458778, EPI_ISL_17458779, EPI_ISL_17458780, EPI_ISL_17458781, EPI_ISL_17458782, EPI_ISL_17458783, EPI_ISL_17458784, EPI_ISL_17458785, EPI_ISL_17458786, EPI_ISL_17458787, EPI_ISL_17458788, EPI_ISL_17458789			
see above	Jamia Hamdard University	Jamia Hamdard University	Khan,W.H. and Ray,P.
EPI_ISL_17458790	Jaypee Institute of Information Technology, Biotechnology	Jaypee Institute of Information Technology, Biotechnology	Sreejith,R., Rana,J., Dudha,N., Kumar,K., Gabrani,R., Sharma,S.K., Gupta,A., Vrati,S., Chaudhary,V.K. and Gupta,S.
EPI_ISL_17458791	Jaypee Institute of Information Technology, Biotechnology	Jaypee Institute of Information Technology, Biotechnology	Rana,J., Sreejith,R., Gulati,S., Bharti,I., Jain,S. and Gupta,S.
EPI_ISL_17458792, EPI_ISL_17458793	Jaypee Institute of Information Technology, Biotechnology	Jaypee Institute of Information Technology, Biotechnology	Gupta,S., Dudha,N., Kumar,K., Gabrani,R., Sharma,S.K., Gupta,A. and Chaudhary,V.K.
EPI_ISL_17458794	Jaypee Institute of Information Technology, Biotechnology	Jaypee Institute of Information Technology, Biotechnology	Sreejith,R., Rana,J., Dudha,N., Kumar,K., Gabrani,R., Sharma,S.K., Gupta,A., Vrati,S., Chaudhary,V.K. and Gupta,S.
EPI_ISL_17458795	Jaypee Institute of Information Technology, Biotechnology	Jaypee Institute of Information Technology, Biotechnology	Rana,J., Sreejith,R., Gulati,S., Bharti,I., Jain,S. and Gupta,S.
EPI_ISL_17458796	Jaypee Institute of Information Technology, Biotechnology	Jaypee Institute of Information Technology, Biotechnology	Sreejith,R., Rana,J., Dudha,N., Kumar,K., Gabrani,R., Sharma,S.K., Gupta,A., Vrati,S., Chaudhary,V.K. and Gupta,S.
EPI_ISL_17458797	Jaypee Institute of Information Technology, Biotechnology	Jaypee Institute of Information Technology, Biotechnology	Gupta,S., Dudha,N., Kumar,K., Gabrani,R., Sharma,S.K., Gupta,A. and Chaudhary,V.K.
EPI_ISL_17458798	Jaypee Institute of Information Technology, Biotechnology	Jaypee Institute of Information Technology, Biotechnology	Sreejith,R., Rana,J., Dudha,N., Kumar,K., Gabrani,R., Sharma,S.K., Gupta,A., Vrati,S., Chaudhary,V.K. and Gupta,S.
EPI_ISL_17458799, EPI_ISL_17458800, EPI_ISL_17458801, EPI_ISL_17458802, EPI_ISL_17458803	Jeffrey Cheah School of Medicine and Health Sciences, Monash University Malaysia	Jeffrey Cheah School of Medicine and Health Sciences, Monash University Malaysia	Ooi,M.K., Adnan,N.A.A., Hassan,S.S. and Dhanoa,A.
EPI_ISL_17458804	Jeffrey Cheah School of Medicine and Health Sciences, Monash University Malaysia	Jeffrey Cheah School of Medicine and Health Sciences, Monash University Malaysia	Ooi,M.K., Gan,H.M., Ahmad,R. and Sharifah,S.H.
EPI_ISL_17458805	Jeffrey Cheah School of Medicine and Health Sciences, Monash University Malaysia	Jeffrey Cheah School of Medicine and Health Sciences, Monash University Malaysia	Ooi,M.K., Adnan,N.A.A., Hassan,S.S. and Dhanoa,A.
EPI_ISL_17458806, EPI_ISL_17458807, EPI_ISL_17458808	Kansai Medical University	Kansai Medical University	Phommanivong,V., Kanda,S., Shimono,T., Lamaningao,P., Darcy,A.W., Mishima,N., Phaytanavanh,B. and Nishiyama,T.
EPI_ISL_17458809	Kenya Medical Research Institute (KEMRI)	Kenya Medical Research Institute (KEMRI)	Nyamwaya,D.K., Otiende,M., Omuoyo,D.O., Githinji,G., Karanja,H.K., Gitonga,J.N., deLaurent,Z.R., Otieno,J.R., Sang,R., Kamau,E., Cheruiyot,S., Otieno,E., Agoti,C.N., Bejon,P., Mwangi,T. and Warimwe,G.M.
EPI_ISL_17458810	Kenya Medical Research Institute (KEMRI)	Kenya Medical Research Institute (KEMRI)	Eyase,F., Langat,S., Berry,I.M., Mulwa,F., Nyunja,A., Mutisya,J., Owaka,S., Limbaso,S., Ofula,V., Koka,H., Koskei,E., Lutomiah,J., Jarman,R.G. and Sang,R.
EPI_ISL_17458811	Kenya Medical Research Institute	Kenya Medical Research Institute	Lutomiah,J., Mulwa,F., Mutisya,J., Koskei,E., Langat,S., Nyunja,A., Koka,H., Konongoi,S., Chepkorir,E., Ofula,V., Owaka,S., Eyase,F. and Sang,R.
EPI_ISL_17458812, EPI_ISL_17458813	Kenya Medical Research Institute (KEMRI)	Kenya Medical Research Institute (KEMRI)	Eyase,F., Langat,S., Berry,I.M., Mulwa,F., Nyunja,A., Mutisya,J., Owaka,S., Limbaso,S., Ofula,V., Koka,H., Koskei,E., Lutomiah,J., Jarman,R.G. and Sang,R.
EPI_ISL_17458814	Kenya Medical Research Institute (KEMRI)	Kenya Medical Research Institute (KEMRI)	Nyamwaya,D.K., Otiende,M., Omuoyo,D.O., Githinji,G., Karanja,H.K., Gitonga,J.N., deLaurent,Z.R., Otieno,J.R., Sang,R., Kamau,E., Cheruiyot,S., Otieno,E., Agoti,C.N., Bejon,P., Mwangi,T. and Warimwe,G.M.
EPI_ISL_17458815, EPI_ISL_17458816, EPI_ISL_17458817, EPI_ISL_17458818	Kenya Medical Research Institute (KEMRI)	Kenya Medical Research Institute (KEMRI)	Eyase,F., Langat,S., Berry,I.M., Mulwa,F., Nyunja,A., Mutisya,J., Owaka,S., Limbaso,S., Ofula,V., Koka,H., Koskei,E., Lutomiah,J., Jarman,R.G. and Sang,R.
EPI_ISL_17458819	Kenya Medical Research Institute (KEMRI)	Kenya Medical Research Institute (KEMRI)	Nyamwaya,D.K., Otiende,M., Omuoyo,D.O., Githinji,G., Karanja,H.K., Gitonga,J.N., deLaurent,Z.R., Otieno,J.R., Sang,R., Kamau,E., Cheruiyot,S., Otieno,E., Agoti,C.N., Bejon,P., Mwangi,T. and Warimwe,G.M.
EPI_ISL_17458820	Kenya Medical Research Institute (KEMRI)	Kenya Medical Research Institute (KEMRI)	Eyase,F., Langat,S., Berry,I.M., Mulwa,F., Nyunja,A., Mutisya,J., Owaka,S., Limbaso,S., Ofula,V., Koka,H., Koskei,E., Lutomiah,J., Jarman,R.G. and Sang,R.
EPI_ISL_17458821	Kenya Medical Research Institute (KEMRI)	Kenya Medical Research Institute (KEMRI)	Nyamwaya,D.K., Otiende,M., Omuoyo,D.O., Githinji,G., Karanja,H.K., Gitonga,J.N., deLaurent,Z.R., Otieno,J.R., Sang,R., Kamau,E., Cheruiyot,S., Otieno,E., Agoti,C.N., Bejon,P., Mwangi,T. and Warimwe,G.M.
EPI_ISL_17458822, EPI_ISL_17458823	Kenya Medical Research Institute (KEMRI)	Kenya Medical Research Institute (KEMRI)	Eyase,F., Langat,S., Berry,I.M., Mulwa,F., Nyunja,A., Mutisya,J., Owaka,S., Limbaso,S., Ofula,V., Koka,H., Koskei,E., Lutomiah,J., Jarman,R.G. and Sang,R.
EPI_ISL_17458824, EPI_ISL_17458825, EPI_ISL_17458826, EPI_ISL_17458827	Kenya Medical Research Institute (KEMRI)	Kenya Medical Research Institute (KEMRI)	Nyamwaya,D.K., Otiende,M., Omuoyo,D.O., Githinji,G., Karanja,H.K., Gitonga,J.N., deLaurent,Z.R., Otieno,J.R., Sang,R., Kamau,E., Cheruiyot,S., Otieno,E., Agoti,C.N., Bejon,P., Mwangi,T. and Warimwe,G.M.
EPI_ISL_17458828	Kenya Medical Research Institute (KEMRI)	Kenya Medical Research Institute (KEMRI)	Eyase,F., Langat,S., Berry,I.M., Mulwa,F., Nyunja,A., Mutisya,J., Owaka,S., Limbaso,S., Ofula,V., Koka,H., Koskei,E., Lutomiah,J., Jarman,R.G. and Sang,R.
EPI_ISL_17458829	Kenya Medical Research Institute	Kenya Medical Research Institute	Lutomiah,J., Mulwa,F., Mutisya,J., Koskei,E., Langat,S., Nyunja,A., Koka,H., Konongoi,S., Chepkorir,E., Ofula,V., Owaka,S., Eyase,F. and Sang,R.
EPI_ISL_17458830	Kenya Medical Research Institute	Kenya Medical Research Institute	Eyase,F., Langat,S., Berry,I.M., Mulwa,F., Nyunja,A., Mutisya,J., Owaka,S., Limbaso,S., Ofula,V., Koka,H., Koskei,E., Lutomiah,J., Jarman,R.G. and Sang,R.
EPI_ISL_17458831	Kenya Medical Research Institute	Kenya Medical Research Institute	Nyamwaya,D.K., Otiende,M., Omuoyo,D.O., Githinji,G., Karanja,H.K., Gitonga,J.N., deLaurent,Z.R., Otieno,J.R., Sang,R., Kamau,E., Cheruiyot,S., Otieno,E., Agoti,C.N., Bejon,P., Mwangi,T. and Warimwe,G.M.
EPI_ISL_17458832, EPI_ISL_17458833, EPI_ISL_17458834	Kenya Medical Research Institute	Kenya Medical Research Institute	Eyase,F., Langat,S., Berry,I.M., Mulwa,F., Nyunja,A., Mutisya,J., Owaka,S., Limbaso,S., Ofula,V., Koka,H., Koskei,E., Lutomiah,J., Jarman,R.G. and Sang,R.
EPI_ISL_17458835, EPI_ISL_17458836	Kenya Medical Research Institute	Kenya Medical Research Institute	Nyamwaya,D.K., Otiende,M., Omuoyo,D.O., Githinji,G., Karanja,H.K., Gitonga,J.N., deLaurent,Z.R., Otieno,J.R., Sang,R., Kamau,E., Cheruiyot,S., Otieno,E., Agoti,C.N., Bejon,P., Mwangi,T. and Warimwe,G.M.
EPI_ISL_17458837	Kenya Medical Research Institute	Kenya Medical Research Institute	Eyase,F., Langat,S., Berry,I.M., Mulwa,F., Nyunja,A., Mutisya,J., Owaka,S., Limbaso,S., Ofula,V., Koka,H., Koskei,E., Lutomiah,J., Jarman,R.G. and Sang,R.
EPI_ISL_17458838	Kenya Medical Research Institute	Kenya Medical Research Institute	Nyamwaya,D.K., Otiende,M., Omuoyo,D.O., Githinji,G., Karanja,H.K., Gitonga,J.N., deLaurent,Z.R., Otieno,J.R., Sang,R., Kamau,E., Cheruiyot,S., Otieno,E., Agoti,C.N., Bejon,P., Mwangi,T. and Warimwe,G.M.
EPI_ISL_17458839	Kenya Medical Research Institute	Kenya Medical Research Institute	Lutomiah,J., Mulwa,F., Mutisya,J., Koskei,E., Langat,S., Nyunja,A., Koka,H., Konongoi,S., Chepkorir,E., Ofula,V., Owaka,S., Eyase,F. and Sang,R.
EPI_ISL_17458840	Kenya Medical Research Institute	Kenya Medical Research Institute	Eyase,F., Langat,S., Berry,I.M., Mulwa,F., Nyunja,A., Mutisya,J., Owaka,S., Limbaso,S., Ofula,V., Koka,H., Koskei,E., Lutomiah,J., Jarman,R.G. and Sang,R.
EPI_ISL_17458841, EPI_ISL_17458842, EPI_ISL_17458843, EPI_ISL_17458844, EPI_ISL_17458845, EPI_ISL_17458846, EPI_ISL_17458847, EPI_ISL_17458848, EPI_ISL_17458849, EPI_ISL_17458850, EPI_ISL_17458851, EPI_ISL_17458852, EPI_ISL_17458853, EPI_ISL_17458854, EPI_ISL_17458855, EPI_ISL_17458856, EPI_ISL_17458857, EPI_ISL_17458858			
see above	Kenya Medical Research Institute, Center for Virus Research	Kenya Medical Research Institute, Center for Virus Research	Konongoi,L., Nyunja,A. and Sang,R.
EPI_ISL_17458859	Khon Kaen University	Khon Kaen University	Le,B.C.T., Pientong,C., Ekalaksananan,T., Aromseree,S., Thaewnongiew,K., Phanitchat,T., Phanthanawiboon,S., Suwannatrai,A.T., Alexander,N., Overgaard,H.J., Chuerduangphui,J. and Bangs,M.J.
EPI_ISL_17458860	King Abdulaziz University	King Abdulaziz University	Alguridi,H.I., Altayb,H. and Alzahrani,F.
EPI_ISL_17458861	King Abdulaziz University	King Abdulaziz University	Alguridi,H., Altayb,H. and Alzahrani,F.
EPI_ISL_17458862, EPI_ISL_17458863, EPI_ISL_17458864, EPI_ISL_17458865	King Institute of Preventive Medicine and Research	King Institute of Preventive Medicine and Research	Gunasekaran,P., Kaveri,K., Mohana,S., Kavitha,A., Saravanamurali,K., Senthilkumar,V., Nagaraj,V.G. and Gracy Fathima,S.
EPI_ISL_17458866, EPI_ISL_17458867, EPI_ISL_17458868, EPI_ISL_17458869, EPI_ISL_17458870	Korea Centers for Disease Control and Prevention, Korea National Institute of Health, Division of Arboviruses, Center for Immunology and Patholog	Korea Centers for Disease Control and Prevention, Korea National Institute of Health, Division of Arboviruses, Center for Immunology and Patholog	Lee,Y.-J., Lee,W.-J. and Kang,S.-Y.
EPI_ISL_17458871	Korea National Institute of Health	Korea National Institute of Health	Cha,G.W., Cho,J.E., Lee,E.J., Ju,Y.R., Han,M.G., Park,C. and Jeong,Y.E.
EPI_ISL_17458872	Korea University	Korea University	Song,M.J., Chung,W.-C., Lee,S. and Kim,Y.
EPI_ISL_17458873, EPI_ISL_17458874, EPI_ISL_17458875, EPI_ISL_17458876	Korea University Research and Business Foundation	Korea University Research and Business Foundation	Hwang,K.Y., Song,M.J., Son,J.H., Kim,S.E. and Chung,W.C.
EPI_ISL_17458877, EPI_ISL_17458878, EPI_ISL_17458879, EPI_ISL_17458880, EPI_ISL_17458881, EPI_ISL_17458882, EPI_ISL_17458883, EPI_ISL_17458884, EPI_ISL_17458885, EPI_ISL_17458886	Kunming University of Science and Technology	Kunming University of Science and Technology	Feng,Y., Xiao,H., Li,X., Lu,Y., Li,J., Zheng,M., Lv,D., Yuan,W., Zhang,Z., Zhou,Y., Liang,Y., Qin,W. and Xia,X.
EPI_ISL_17458887, EPI_ISL_17458888	Laboratorio Regional para el Diagnóstico del Dengue y otras Enfermedades Virales, Universidad de Carabobo	Laboratorio Regional para el Diagnóstico del Dengue y otras Enfermedades Virales, Universidad de Carabobo	Camacho,D.E., Reyes,J.D., Negredo,A.I., Sanchez-Seco,M.P., Araujo,R., Alcantara,A. and Comach,G.
EPI_ISL_17458889, EPI_ISL_17458890, EPI_ISL_17458891, EPI_ISL_17458892	Laboratorio Regional para el Diagnóstico del Dengue y otras Enfermedades Virales, Universidad de Carabobo	Laboratorio Regional para el Diagnóstico del Dengue y otras Enfermedades Virales, Universidad de Carabobo	Camacho,D.E., Negredo,A.I., Reyes,J.D., Comach,G.A. and Sanchez-Seco,M.P.
EPI_ISL_17458893, EPI_ISL_17458894	Laboratorio Regional para el Diagnóstico del Dengue y otras Enfermedades Virales, Universidad de Carabobo	Laboratorio Regional para el Diagnóstico del Dengue y otras Enfermedades Virales, Universidad de Carabobo	Camacho,D.E., Reyes,J.D., Negredo,A.I., Sanchez-Seco,M.P., Araujo,R., Alcantara,A. and Comach,G.
EPI_ISL_17458895	Laboratorio de Genetica, Hematologia e Biologia Computacional, Centro de Pesquisa Goncalo Moniz, Fiocruz Bahia	Laboratorio de Genetica, Hematologia e Biologia Computacional, Centro de Pesquisa Goncalo Moniz, Fiocruz Bahia	Santana do Rosario,M., Giovanetti,M., Pereira de Jesus,A., Santana Farias,D., Nunes,M., Guerreiro Rodrigues,S., Alcantara,L.C.J. and de Siqueira,I.C.
EPI_ISL_17458896, EPI_ISL_17458897, EPI_ISL_17458898, EPI_ISL_17458899, EPI_ISL_17458900, EPI_ISL_17458901, EPI_ISL_17458902, EPI_ISL_17458903, EPI_ISL_17458904, EPI_ISL_17458905, EPI_ISL_17458906, EPI_ISL_17458907, EPI_ISL_17458908, EPI_ISL_17458909, EPI_ISL_17458910, EPI_ISL_17458911, EPI_ISL_17458912			
see above	Laboratory of Virology, Faculty of Medicine, Federal University of Mato Grosso	Laboratory of Virology, Faculty of Medicine, Federal University of Mato Grosso	Heinen,L.B.S., Silva,E.R., Cardoso,B.F., Costa,M.C.S., Maia,L.M.S., Bezerra,M.C.F. and Dezengrini-Slhessarenko,R.
EPI_ISL_17458913, EPI_ISL_17458914, EPI_ISL_17458915, EPI_ISL_17458916, EPI_ISL_17458917, EPI_ISL_17458918,	Laboratório de Virologia e Rickettsiose da Faculdade de Medicina Veterinária,	Laboratório de Virologia e Rickettsiose da Faculdade de Medicina Veterinária,	Santos,M.A.M., Pavon,J.A.R., Viniski,A.E., Souza,C.L.C., Oliveira,E.C., Nunes,M.R.T. and Slhessarenko,R.D.

EPI_ISL_17458919	Universidade Federal do Mato Grosso	Universidade Federal do Mato Grosso	
EPI_ISL_17458920	Le Centre Hospitalier Universitaire de Toulouse	Le Centre Hospitalier Universitaire de Toulouse	Mansuy,J.M., Grouteau,E., Mengelle,C., Claudet,I. and Izopet,J.
EPI_ISL_17458921	Leiden University Medical Center	Leiden University Medical Center	Scholte,F.E., Tas,A., Martina,B.E., Cordioli,P., Narayanan,K., Makino,S., Snijder,E.J. and van Hemert,M.J.
EPI_ISL_17458922, EPI_ISL_17458923, EPI_ISL_17458924, EPI_ISL_17458925	Liverpool School of Tropical Medicine	Liverpool School of Tropical Medicine	Edwards,T., Signor,L.D., Williams,C., Donis,E., Cuevas,L.E. and Adams,E.R.
EPI_ISL_17458926	Liverpool School of Tropical Medicine	Liverpool School of Tropical Medicine	Edwards,T., Adams,E., Cuevas,L. and Williams,C.
EPI_ISL_17458927, EPI_ISL_17458928	Liverpool School of Tropical Medicine	Liverpool School of Tropical Medicine	Edwards,T., Signor,L.D., Williams,C., Donis,E., Cuevas,L.E. and Adams,E.R.
EPI_ISL_17458929, EPI_ISL_17458930, EPI_ISL_17458931, EPI_ISL_17458932, EPI_ISL_17458933, EPI_ISL_17458934, EPI_ISL_17458935, EPI_ISL_17458936, EPI_ISL_17458937, EPI_ISL_17458938, EPI_ISL_17458939, EPI_ISL_17458940, EPI_ISL_17458941, EPI_ISL_17458942, EPI_ISL_17458943, EPI_ISL_17458944, EPI_ISL_17458945, EPI_ISL_17458946, EPI_ISL_17458947, EPI_ISL_17458948, EPI_ISL_17458949, EPI_ISL_17458950, EPI_ISL_17458951, EPI_ISL_17458952, EPI_ISL_17458953			
see above	London School of Hygiene and Tropical Medicine	London School of Hygiene and Tropical Medicine	Stubbs,S.C.B., Johar,E., Yudhaputri,F.A., Yohan,B., Santoso,M.S., Hayati,R.F., Denis,D., Blacklaws,B.A., Powers,A.M., Sasmono,R.T., Myint,K.S.A. and Frost,S.D.W.
EPI_ISL_17458954	Ludwig-Maximilians University	Ludwig-Maximilians University	Pfeffer,M., Kinney,R.M. and Kaaden,O.R.
EPI_ISL_17458955	Ludwig-Maximilians University	Ludwig-Maximilians University	Pfeffer,M., Proebster,B., Kinney,R.M. and Kaaden,O.R.
EPI_ISL_17458956	Madras Veterinary College	Madras Veterinary College	Sasikalaveni,A. and Raja,A.
EPI_ISL_17458957, EPI_ISL_17458958, EPI_ISL_17458959	Madras Veterinary College	Madras Veterinary College	Dhinakar Raj,G., Rajanathan,C.T.M. and Ramadass,P.
EPI_ISL_17458960	Madras Veterinary College	Madras Veterinary College	Parthiban,M., Wilson,A., Sathiya,P. and Madhuvanthi,S.
EPI_ISL_17458961, EPI_ISL_17458962, EPI_ISL_17458963, EPI_ISL_17458964	Madras Veterinary College	Madras Veterinary College	Sasikalaveni,A. and Raja,A.
EPI_ISL_17458965	Madras Veterinary College	Madras Veterinary College	Dhinakar Raj,G., Rajanathan,C.T.M. and Ramadass,P.
EPI_ISL_17458966	Madras Veterinary College	Madras Veterinary College	Parthiban,M., Wilson,A., Sathiya,P. and Madhuvanthi,S.
EPI_ISL_17458967	Madras Veterinary College	Madras Veterinary College	Dhinakar Raj,G., Rajanathan,C.T.M. and Ramadass,P.
EPI_ISL_17458968	Madras Veterinary College	Madras Veterinary College	Sasikalaveni,A. and Raja,A.
EPI_ISL_17458969	Madras Veterinary College	Madras Veterinary College	Dhinakar Raj,G., Rajanathan,C.T.M. and Ramadass,P.
EPI_ISL_17458970, EPI_ISL_17458971, EPI_ISL_17458972, EPI_ISL_17458973, EPI_ISL_17458974, EPI_ISL_17458975, EPI_ISL_17458976, EPI_ISL_17458977, EPI_ISL_17458978, EPI_ISL_17458979, EPI_ISL_17458980, EPI_ISL_17458981, EPI_ISL_17458982, EPI_ISL_17458983, EPI_ISL_17458984, EPI_ISL_17458985, EPI_ISL_17458986, EPI_ISL_17458987, EPI_ISL_17458988, EPI_ISL_17458989, EPI_ISL_17458990, EPI_ISL_17458991, EPI_ISL_17458992, EPI_ISL_17458993, EPI_ISL_17458994, EPI_ISL_17458995, EPI_ISL_17458996, EPI_ISL_17458997, EPI_ISL_17458998, EPI_ISL_17458999, EPI_ISL_17459000, EPI_ISL_17459001, EPI_ISL_17459002, EPI_ISL_17459003, EPI_ISL_17459004, EPI_ISL_17459005, EPI_ISL_17459006, EPI_ISL_17459007, EPI_ISL_17459008, EPI_ISL_17459009, EPI_ISL_17459010, EPI_ISL_17459011, EPI_ISL_17459012, EPI_ISL_17459013, EPI_ISL_17459014, EPI_ISL_17459015			
see above	Mahidol University	Mahidol University	Sasayama,M., Benjathummarak,S., Kawashita,N., Rukmanee,P., Sangmukdanun,S., Masrinoul,P., Pitaksajjakul,P., Puiprom,O., Wuthisen,P., Kurosu,T., Chaichana,P., Maneekan,P., Ikuta,K., Ramasoota,P., Okabayashi,T., Singhasivanon,P. and Luplertlop,N.
EPI_ISL_17459016	Mahidol University	Mahidol University	Tuekprakhon,A., Puiprom,O., Sasaki,T., Michiels,J., Bartholomeeusen,K., Nakayama,E.E., Meno,M.K., Phadungsombat,J., Huits,R., Arien,K.K., Luplertlop,N., Shioda,T. and Leaungwutiwong,P.
EPI_ISL_17459017, EPI_ISL_17459018, EPI_ISL_17459019, EPI_ISL_17459020, EPI_ISL_17459021, EPI_ISL_17459022, EPI_ISL_17459023, EPI_ISL_17459024, EPI_ISL_17459025, EPI_ISL_17459026, EPI_ISL_17459027, EPI_ISL_17459028, EPI_ISL_17459029, EPI_ISL_17459030, EPI_ISL_17459031, EPI_ISL_17459032, EPI_ISL_17459033, EPI_ISL_17459034, EPI_ISL_17459035, EPI_ISL_17459036, EPI_ISL_17459037, EPI_ISL_17459038, EPI_ISL_17459039, EPI_ISL_17459040, EPI_ISL_17459041, EPI_ISL_17459042, EPI_ISL_17459043, EPI_ISL_17459044, EPI_ISL_17459045, EPI_ISL_17459046, EPI_ISL_17459047, EPI_ISL_17459048, EPI_ISL_17459049, EPI_ISL_17459050, EPI_ISL_17459051, EPI_ISL_17459052, EPI_ISL_17459053, EPI_ISL_17459054, EPI_ISL_17459055, EPI_ISL_17459056, EPI_ISL_17459057, EPI_ISL_17459058, EPI_ISL_17459059, EPI_ISL_17459060, EPI_ISL_17459061, EPI_ISL_17459062, EPI_ISL_17459063, EPI_ISL_17459064, EPI_ISL_17459065, EPI_ISL_17459066, EPI_ISL_17459067, EPI_ISL_17459068, EPI_ISL_17459069, EPI_ISL_17459070, EPI_ISL_17459071, EPI_ISL_17459072, EPI_ISL_17459073, EPI_ISL_17459074, EPI_ISL_17459075, EPI_ISL_17459076, EPI_ISL_17459077			
see above	Mahidol University	Mahidol University	Sasayama,M., Benjathummarak,S., Kawashita,N., Rukmanee,P., Sangmukdanun,S., Masrinoul,P., Pitaksajjakul,P., Puiprom,O., Wuthisen,P., Kurosu,T., Chaichana,P., Maneekan,P., Ikuta,K., Ramasoota,P., Okabayashi,T., Singhasivanon,P. and Luplertlop,N.
EPI_ISL_17459078	Mahidol University	Mahidol University	Pulmanausahakul,R. and Jaimipuk,T.
EPI_ISL_17459079, EPI_ISL_17459080, EPI_ISL_17459081, EPI_ISL_17459082, EPI_ISL_17459083	Mahidol University	Mahidol University	Sasayama,M., Benjathummarak,S., Kawashita,N., Rukmanee,P., Sangmukdanun,S., Masrinoul,P., Pitaksajjakul,P., Puiprom,O., Wuthisen,P., Kurosu,T., Chaichana,P., Maneekan,P., Ikuta,K., Ramasoota,P., Okabayashi,T., Singhasivanon,P. and Luplertlop,N.
EPI_ISL_17459084, EPI_ISL_17459085, EPI_ISL_17459086, EPI_ISL_17459087, EPI_ISL_17459088, EPI_ISL_17459089, EPI_ISL_17459090, EPI_ISL_17459091, EPI_ISL_17459092, EPI_ISL_17459093, EPI_ISL_17459094, EPI_ISL_17459095, EPI_ISL_17459096			
see above	Mahidol University, Faculty of Medicine Siriraj Hospital, Department of Immunology	Mahidol University, Faculty of Medicine Siriraj Hospital, Department of Immunology	Boonnak,K. and Tun,Y.M.
EPI_ISL_17459097, EPI_ISL_17459098, EPI_ISL_17459099, EPI_ISL_17459100	Mahidol-Osaka Center for Infectious Diseases, Faculty of Tropical Medicine, Mahidol University	Mahidol-Osaka Center for Infectious Diseases, Faculty of Tropical Medicine, Mahidol University	Phadungsombat,J., Imad,H., Rahman,M., Nakayama,E.E., Kludkleeb,S., Ponam,T., Rahim,R., Hasan,A., Poltep,K., Yamanaka,A., Matsee,W., Piyaphanee,W., Phumratanaprapin,W. and Shioda,T.
EPI_ISL_17459101, EPI_ISL_17459102, EPI_ISL_17459103	Mahidol-Osaka Center for Infectious Diseases, Faculty of Tropical Medicine, Mahidol University	Mahidol-Osaka Center for Infectious Diseases, Faculty of Tropical Medicine, Mahidol University	Phadungsombat,J., Tuekprakhon,A., Cnops,L., Michiels,J., Van Den Berg,R., Nakayama,E.E., Shioda,T., Arien,K.K. and Huits,R.
EPI_ISL_17459104	Mahidol-Osaka Center for Infectious Diseases, Faculty of Tropical Medicine, Mahidol University	Mahidol-Osaka Center for Infectious Diseases, Faculty of Tropical Medicine, Mahidol University	Phadungsombat,J., Imad,H., Rahman,M., Nakayama,E.E., Kludkleeb,S., Ponam,T., Rahim,R., Hasan,A., Poltep,K., Yamanaka,A., Matsee,W., Piyaphanee,W., Phumratanaprapin,W. and Shioda,T.
EPI_ISL_17459105	Mahidol-Osaka Center for Infectious Diseases, Faculty of Tropical Medicine, Mahidol University	Mahidol-Osaka Center for Infectious Diseases, Faculty of Tropical Medicine, Mahidol University	Imad,H.A., Phadungsombat,J., Nakayama,E.E., Suzuki,K., Ibrahim,A.M., Afaa,A., Azeema,A., Nazfa,A., Yazfa,A., Ahmed,A., Saeed,A., Waheed,A., Shareef,F., Islam,M.M., Anees,S.M., Saleem,S., Aroosha,A., Afzal,I., Leaungwutiwong,P., Piyaphanee,W., Phumratanaprapin,W. and Shioda,T.
EPI_ISL_17459106	Mahidol-Osaka Center for Infectious Diseases, Faculty of Tropical Medicine, Mahidol University	Mahidol-Osaka Center for Infectious Diseases, Faculty of Tropical Medicine, Mahidol University	Phadungsombat,J., Imad,H., Rahman,M., Nakayama,E.E., Kludkleeb,S., Ponam,T., Rahim,R., Hasan,A., Poltep,K., Yamanaka,A., Matsee,W., Piyaphanee,W., Phumratanaprapin,W. and Shioda,T.
EPI_ISL_17459107, EPI_ISL_17459108, EPI_ISL_17459109	Mahidol-Osaka Center for Infectious Diseases, Faculty of Tropical Medicine, Mahidol University	Mahidol-Osaka Center for Infectious Diseases, Faculty of Tropical Medicine, Mahidol University	Imad,H.A., Phadungsombat,J., Nakayama,E.E., Suzuki,K., Ibrahim,A.M., Afaa,A., Azeema,A., Nazfa,A., Yazfa,A., Ahmed,A., Saeed,A., Waheed,A., Shareef,F., Islam,M.M., Anees,S.M., Saleem,S., Aroosha,A., Afzal,I., Leaungwutiwong,P., Piyaphanee,W., Phumratanaprapin,W. and Shioda,T.
EPI_ISL_17459110	Mahidol-Osaka Center for Infectious Diseases, Faculty of Tropical Medicine, Mahidol University	Mahidol-Osaka Center for Infectious Diseases, Faculty of Tropical Medicine, Mahidol University	Imad,H.A., Phadungsombat,J., Nakayama,E.E., Kludkleeb,S., Matsee,W., Ponam,T., Suzuki,K., Leaungwutiwong,P., Piyaphanee,W., Phumratanaprapin,W. and Shioda,T.
EPI_ISL_17459111	Mahidol-Osaka Center for Infectious Diseases, Faculty of Tropical Medicine, Mahidol University	Mahidol-Osaka Center for Infectious Diseases, Faculty of Tropical Medicine, Mahidol University	Imad,H.A., Phadungsombat,J., Nakayama,E.E., Suzuki,K., Ibrahim,A.M., Afaa,A., Azeema,A., Nazfa,A., Yazfa,A., Ahmed,A., Saeed,A., Waheed,A., Shareef,F., Islam,M.M., Anees,S.M., Saleem,S., Aroosha,A., Afzal,I., Leaungwutiwong,P., Piyaphanee,W., Phumratanaprapin,W. and Shioda,T.
EPI_ISL_17459112	Mahidol-Osaka Center for Infectious Diseases, Faculty of Tropical Medicine, Mahidol University	Mahidol-Osaka Center for Infectious Diseases, Faculty of Tropical Medicine, Mahidol University	Phadungsombat,J., Tuekprakhon,A., Cnops,L., Michiels,J., Van Den Berg,R., Nakayama,E.E., Shioda,T., Arien,K.K. and Huits,R.
EPI_ISL_17459113	Mahidol-Osaka Center for Infectious Diseases, Faculty of Tropical Medicine, Mahidol University	Mahidol-Osaka Center for Infectious Diseases, Faculty of Tropical Medicine, Mahidol University	Imad,H.A., Phadungsombat,J., Nakayama,E.E., Suzuki,K., Ibrahim,A.M., Afaa,A., Azeema,A., Nazfa,A., Yazfa,A., Ahmed,A., Saeed,A., Waheed,A., Shareef,F., Islam,M.M., Anees,S.M., Saleem,S., Aroosha,A., Afzal,I., Leaungwutiwong,P., Piyaphanee,W., Phumratanaprapin,W. and Shioda,T.
EPI_ISL_17459114, EPI_ISL_17459115, EPI_ISL_17459116, EPI_ISL_17459117, EPI_ISL_17459118, EPI_ISL_17459119, EPI_ISL_17459120	Mahidol-Osaka Center for Infectious Diseases, Faculty of Tropical Medicine, Mahidol University	Mahidol-Osaka Center for Infectious Diseases, Faculty of Tropical Medicine, Mahidol University	Phadungsombat,J., Imad,H., Rahman,M., Nakayama,E.E., Kludkleeb,S., Ponam,T., Rahim,R., Hasan,A., Poltep,K., Yamanaka,A., Matsee,W., Piyaphanee,W., Phumratanaprapin,W. and Shioda,T.
EPI_ISL_17459121	Mahidol-Osaka Center for Infectious Diseases, Faculty of Tropical Medicine, Mahidol University	Mahidol-Osaka Center for Infectious Diseases, Faculty of Tropical Medicine, Mahidol University	Imad,H.A., Phadungsombat,J., Nakayama,E.E., Suzuki,K., Ibrahim,A.M., Afaa,A., Azeema,A., Nazfa,A., Yazfa,A., Ahmed,A., Saeed,A., Waheed,A., Shareef,F., Islam,M.M., Anees,S.M., Saleem,S., Aroosha,A., Afzal,I., Leaungwutiwong,P., Piyaphanee,W., Phumratanaprapin,W. and Shioda,T.
EPI_ISL_17459122	Mahidol-Osaka Center for Infectious Diseases, Faculty of Tropical Medicine, Mahidol University	Mahidol-Osaka Center for Infectious Diseases, Faculty of Tropical Medicine, Mahidol University	Phadungsombat,J., Imad,H., Rahman,M., Nakayama,E.E., Kludkleeb,S., Ponam,T., Rahim,R., Hasan,A., Poltep,K., Yamanaka,A., Matsee,W., Piyaphanee,W., Phumratanaprapin,W. and Shioda,T.
EPI_ISL_17459123, EPI_ISL_17459124, EPI_ISL_17459125	Mahidol-Osaka Center for Infectious Diseases, Faculty of Tropical Medicine, Mahidol University	Mahidol-Osaka Center for Infectious Diseases, Faculty of Tropical Medicine, Mahidol University	Imad,H.A., Phadungsombat,J., Nakayama,E.E., Suzuki,K., Ibrahim,A.M., Afaa,A., Azeema,A., Nazfa,A., Yazfa,A., Ahmed,A., Saeed,A., Waheed,A., Shareef,F., Islam,M.M., Anees,S.M., Saleem,S., Aroosha,A., Afzal,I., Leaungwutiwong,P., Piyaphanee,W., Phumratanaprapin,W. and Shioda,T.
EPI_ISL_17459126, EPI_ISL_17459127, EPI_ISL_17459128	Mahidol-Osaka Center for Infectious Diseases, Faculty of Tropical Medicine, Mahidol University	Mahidol-Osaka Center for Infectious Diseases, Faculty of Tropical Medicine, Mahidol University	Phadungsombat,J., Tuekprakhon,A., Cnops,L., Michiels,J., Van Den Berg,R., Nakayama,E.E., Shioda,T., Arien,K.K. and Huits,R.
EPI_ISL_17459129	Mahidol-Osaka Center for Infectious Diseases, Faculty of Tropical Medicine, Mahidol University	Mahidol-Osaka Center for Infectious Diseases, Faculty of Tropical Medicine, Mahidol University	Phadungsombat,J., Imad,H., Rahman,M., Nakayama,E.E., Kludkleeb,S., Ponam,T., Rahim,R., Hasan,A., Poltep,K., Yamanaka,A., Matsee,W., Piyaphanee,W., Phumratanaprapin,W. and Shioda,T.
EPI_ISL_17459130, EPI_ISL_17459131, EPI_ISL_17459132, EPI_ISL_17459133	Mahidol-Osaka Center for Infectious Diseases, Faculty of Tropical Medicine, Mahidol University	Mahidol-Osaka Center for Infectious Diseases, Faculty of Tropical Medicine, Mahidol University	Imad,H.A., Phadungsombat,J., Nakayama,E.E., Suzuki,K., Ibrahim,A.M., Afaa,A., Azeema,A., Nazfa,A., Yazfa,A., Ahmed,A., Saeed,A., Waheed,A., Shareef,F., Islam,M.M., Anees,S.M., Saleem,S., Aroosha,A., Afzal,I., Leaungwutiwong,P., Piyaphanee,W., Phumratanaprapin,W. and Shioda,T.
EPI_ISL_17459134, EPI_ISL_17459135	Mahidol-Osaka Center for Infectious Diseases, Faculty of Tropical Medicine, Mahidol University	Mahidol-Osaka Center for Infectious Diseases, Faculty of Tropical Medicine, Mahidol University	Imad,H.A., Phadungsombat,J., Nakayama,E.E., Kludkleeb,S., Matsee,W., Ponam,T., Suzuki,K., Leaungwutiwong,P., Piyaphanee,W., Phumratanaprapin,W. and Shioda,T.
EPI_ISL_17459136, EPI_ISL_17459137, EPI_ISL_17459138, EPI_ISL_17459139	Mahidol-Osaka Center for Infectious Diseases, Faculty of Tropical Medicine, Mahidol University	Mahidol-Osaka Center for Infectious Diseases, Faculty of Tropical Medicine, Mahidol University	Phadungsombat,J., Imad,H., Rahman,M., Nakayama,E.E., Kludkleeb,S., Ponam,T., Rahim,R., Hasan,A., Poltep,K., Yamanaka,A., Matsee,W., Piyaphanee,W., Phumratanaprapin,W. and Shioda,T.
EPI_ISL_17459140	Mahidol-Osaka Center for Infectious Diseases, Faculty of Tropical Medicine, Mahidol University	Mahidol-Osaka Center for Infectious Diseases, Faculty of Tropical Medicine, Mahidol University	Imad,H.A., Phadungsombat,J., Nakayama,E.E., Kludkleeb,S., Matsee,W., Ponam,T., Suzuki,K., Leaungwutiwong,P., Piyaphanee,W., Phumratanaprapin,W. and Shioda,T.
EPI_ISL_17459141	Mahidol-Osaka Center for Infectious Diseases, Faculty of Tropical Medicine, Mahidol University	Mahidol-Osaka Center for Infectious Diseases, Faculty of Tropical Medicine, Mahidol University	Phadungsombat,J., Imad,H., Rahman,M., Nakayama,E.E., Kludkleeb,S., Ponam,T., Rahim,R., Hasan,A., Poltep,K., Yamanaka,A., Matsee,W., Piyaphanee,W., Phumratanaprapin,W. and Shioda,T.
EPI_ISL_17459142	Mahidol-Osaka Center for Infectious Diseases, Faculty of Tropical Medicine, Mahidol University	Mahidol-Osaka Center for Infectious Diseases, Faculty of Tropical Medicine, Mahidol University	Imad,H.A., Phadungsombat,J., Nakayama,E.E., Kludkleeb,S., Matsee,W., Ponam,T., Suzuki,K., Leaungwutiwong,P., Piyaphanee,W., Phumratanaprapin,W. and Shioda,T.
EPI_ISL_17459143, EPI_ISL_17459144, EPI_ISL_17459145, EPI_ISL_17459146	Mahidol-Osaka Center for Infectious Diseases, Faculty of Tropical Medicine, Mahidol University	Mahidol-Osaka Center for Infectious Diseases, Faculty of Tropical Medicine, Mahidol University	Phadungsombat,J., Imad,H., Rahman,M., Nakayama,E.E., Kludkleeb,S., Ponam,T., Rahim,R., Hasan,A., Poltep,K., Yamanaka,A., Matsee,W., Piyaphanee,W., Phumratanaprapin,W. and Shioda,T.
EPI_ISL_17459147	Mahidol-Osaka Center for Infectious Diseases,	Mahidol-Osaka Center for Infectious Diseases,	Imad,H.A., Phadungsombat,J., Nakayama,E.E., Suzuki,K., Ibrahim,A.M., Afaa,A., Azeema,A., Nazfa,A., Yazfa,A., Ahmed,A., Saeed,A., Waheed,A., Shareef,F., Islam,M.M., Anees,S.M., Saleem,S., Aroosha,A., Afzal,I., Leaungwutiwong,P.,

	Faculty of Tropical Medicine, Mahidol University	Faculty of Tropical Medicine, Mahidol University	Piyaphanee,W., Phumratanaprapin,W. and Shioda,T.
EPI_ISL_17459148	Mahidol-Osaka Center for Infectious Diseases, Faculty of Tropical Medicine, Mahidol University	Mahidol-Osaka Center for Infectious Diseases, Faculty of Tropical Medicine, Mahidol University	Imad,H.A., Phadungsombat,J., Nakayama,E.E., Kludkleeb,S., Matsee,W., Ponam,T., Suzuki,K., Leaungwutiwong,P., Piyaphanee,W., Phumratanaprapin,W. and Shioda,T.
EPI_ISL_17459149	Mahidol-Osaka Center for Infectious Diseases, Faculty of Tropical Medicine, Mahidol University	Mahidol-Osaka Center for Infectious Diseases, Faculty of Tropical Medicine, Mahidol University	Imad,H.A., Phadungsombat,J., Nakayama,E.E., Suzuki,K., Ibrahim,A.M., Afaa,A., Azeema,A., Nazfa,A., Yazfa,A., Ahmed,A., Saeed,A., Waheed,A., Shareef,F., Islam,M.M., Anees,S.M., Saleem,S., Aroosha,A., Afzal,I., Leaungwutiwong,P., Piyaphanee,W., Phumratanaprapin,W. and Shioda,T.
EPI_ISL_17459150, EPI_ISL_17459151	Mahidol-Osaka Center for Infectious Diseases, Faculty of Tropical Medicine, Mahidol University	Mahidol-Osaka Center for Infectious Diseases, Faculty of Tropical Medicine, Mahidol University	Imad,H.A., Phadungsombat,J., Nakayama,E.E., Kludkleeb,S., Matsee,W., Ponam,T., Suzuki,K., Leaungwutiwong,P., Piyaphanee,W., Phumratanaprapin,W. and Shioda,T.
EPI_ISL_17459152, EPI_ISL_17459153, EPI_ISL_17459154, EPI_ISL_17459155, EPI_ISL_17459156, EPI_ISL_17459157	Mahidol-Osaka Center for Infectious Diseases, Faculty of Tropical Medicine, Mahidol University	Mahidol-Osaka Center for Infectious Diseases, Faculty of Tropical Medicine, Mahidol University	Imad,H.A., Phadungsombat,J., Nakayama,E.E., Suzuki,K., Ibrahim,A.M., Afaa,A., Azeema,A., Nazfa,A., Yazfa,A., Ahmed,A., Saeed,A., Waheed,A., Shareef,F., Islam,M.M., Anees,S.M., Saleem,S., Aroosha,A., Afzal,I., Leaungwutiwong,P., Piyaphanee,W., Phumratanaprapin,W. and Shioda,T.
EPI_ISL_17459158	Mahidol-Osaka Center for Infectious Diseases, Faculty of Tropical Medicine, Mahidol University	Mahidol-Osaka Center for Infectious Diseases, Faculty of Tropical Medicine, Mahidol University	Imad,H.A., Phadungsombat,J., Nakayama,E.E., Kludkleeb,S., Matsee,W., Ponam,T., Suzuki,K., Leaungwutiwong,P., Piyaphanee,W., Phumratanaprapin,W. and Shioda,T.
EPI_ISL_17459159	Mahidol-Osaka Center for Infectious Diseases, Faculty of Tropical Medicine, Mahidol University	Mahidol-Osaka Center for Infectious Diseases, Faculty of Tropical Medicine, Mahidol University	Imad,H.A., Phadungsombat,J., Nakayama,E.E., Suzuki,K., Ibrahim,A.M., Afaa,A., Azeema,A., Nazfa,A., Yazfa,A., Ahmed,A., Saeed,A., Waheed,A., Shareef,F., Islam,M.M., Anees,S.M., Saleem,S., Aroosha,A., Afzal,I., Leaungwutiwong,P., Piyaphanee,W., Phumratanaprapin,W. and Shioda,T.
EPI_ISL_17459160, EPI_ISL_17459161, EPI_ISL_17459162, EPI_ISL_17459163, EPI_ISL_17459164, EPI_ISL_17459165	Mahidol-Osaka Center for Infectious Diseases, Faculty of Tropical Medicine, Mahidol University	Mahidol-Osaka Center for Infectious Diseases, Faculty of Tropical Medicine, Mahidol University	Phadungsombat,J., Imad,H., Rahman,M., Nakayama,E.E., Kludkleeb,S., Ponam,T., Rahim,R., Hasan,A., Poltep,K., Yamanaka,A., Matsee,W., Piyaphanee,W., Phumratanaprapin,W. and Shioda,T.
EPI_ISL_17459166	Mahidol-Osaka Center for Infectious Diseases, Faculty of Tropical Medicine, Mahidol University	Mahidol-Osaka Center for Infectious Diseases, Faculty of Tropical Medicine, Mahidol University	Imad,H.A., Phadungsombat,J., Nakayama,E.E., Suzuki,K., Ibrahim,A.M., Afaa,A., Azeema,A., Nazfa,A., Yazfa,A., Ahmed,A., Saeed,A., Waheed,A., Shareef,F., Islam,M.M., Anees,S.M., Saleem,S., Aroosha,A., Afzal,I., Leaungwutiwong,P., Piyaphanee,W., Phumratanaprapin,W. and Shioda,T.
EPI_ISL_17459167, EPI_ISL_17459168	Mahidol-Osaka Center for Infectious Diseases, Faculty of Tropical Medicine, Mahidol University	Mahidol-Osaka Center for Infectious Diseases, Faculty of Tropical Medicine, Mahidol University	Phadungsombat,J., Imad,H., Rahman,M., Nakayama,E.E., Kludkleeb,S., Ponam,T., Rahim,R., Hasan,A., Poltep,K., Yamanaka,A., Matsee,W., Piyaphanee,W., Phumratanaprapin,W. and Shioda,T.
EPI_ISL_17459169	Mahidol-Osaka Center for Infectious Diseases, Faculty of Tropical Medicine, Mahidol University	Mahidol-Osaka Center for Infectious Diseases, Faculty of Tropical Medicine, Mahidol University	Imad,H.A., Phadungsombat,J., Nakayama,E.E., Suzuki,K., Ibrahim,A.M., Afaa,A., Azeema,A., Nazfa,A., Yazfa,A., Ahmed,A., Saeed,A., Waheed,A., Shareef,F., Islam,M.M., Anees,S.M., Saleem,S., Aroosha,A., Afzal,I., Leaungwutiwong,P., Piyaphanee,W., Phumratanaprapin,W. and Shioda,T.
EPI_ISL_17459170	Mahidol-Osaka Center for Infectious Diseases, Faculty of Tropical Medicine, Mahidol University	Mahidol-Osaka Center for Infectious Diseases, Faculty of Tropical Medicine, Mahidol University	Phadungsombat,J., Imad,H., Rahman,M., Nakayama,E.E., Kludkleeb,S., Ponam,T., Rahim,R., Hasan,A., Poltep,K., Yamanaka,A., Matsee,W., Piyaphanee,W., Phumratanaprapin,W. and Shioda,T.
EPI_ISL_17459171, EPI_ISL_17459172, EPI_ISL_17459173, EPI_ISL_17459174	Mahidol-Osaka Center for Infectious Diseases, Faculty of Tropical Medicine, Mahidol University	Mahidol-Osaka Center for Infectious Diseases, Faculty of Tropical Medicine, Mahidol University	Imad,H.A., Phadungsombat,J., Nakayama,E.E., Suzuki,K., Ibrahim,A.M., Afaa,A., Azeema,A., Nazfa,A., Yazfa,A., Ahmed,A., Saeed,A., Waheed,A., Shareef,F., Islam,M.M., Anees,S.M., Saleem,S., Aroosha,A., Afzal,I., Leaungwutiwong,P., Piyaphanee,W., Phumratanaprapin,W. and Shioda,T.
EPI_ISL_17459175	Mahidol-Osaka Center for Infectious Diseases, Faculty of Tropical Medicine, Mahidol University	Mahidol-Osaka Center for Infectious Diseases, Faculty of Tropical Medicine, Mahidol University	Phadungsombat,J., Imad,H., Rahman,M., Nakayama,E.E., Kludkleeb,S., Ponam,T., Rahim,R., Hasan,A., Poltep,K., Yamanaka,A., Matsee,W., Piyaphanee,W., Phumratanaprapin,W. and Shioda,T.
EPI_ISL_17459176	Mahidol-Osaka Center for Infectious Diseases, Faculty of Tropical Medicine, Mahidol University	Mahidol-Osaka Center for Infectious Diseases, Faculty of Tropical Medicine, Mahidol University	Imad,H.A., Phadungsombat,J., Nakayama,E.E., Suzuki,K., Ibrahim,A.M., Afaa,A., Azeema,A., Nazfa,A., Yazfa,A., Ahmed,A., Saeed,A., Waheed,A., Shareef,F., Islam,M.M., Anees,S.M., Saleem,S., Aroosha,A., Afzal,I., Leaungwutiwong,P., Piyaphanee,W., Phumratanaprapin,W. and Shioda,T.
EPI_ISL_17459177	Mahidol-Osaka Center for Infectious Diseases, Faculty of Tropical Medicine, Mahidol University	Mahidol-Osaka Center for Infectious Diseases, Faculty of Tropical Medicine, Mahidol University	Phadungsombat,J., Imad,H., Rahman,M., Nakayama,E.E., Kludkleeb,S., Ponam,T., Rahim,R., Hasan,A., Poltep,K., Yamanaka,A., Matsee,W., Piyaphanee,W., Phumratanaprapin,W. and Shioda,T.
EPI_ISL_17459178, EPI_ISL_17459179, EPI_ISL_17459180, EPI_ISL_17459181	Mahidol-Osaka Center for Infectious Diseases, Faculty of Tropical Medicine, Mahidol University	Mahidol-Osaka Center for Infectious Diseases, Faculty of Tropical Medicine, Mahidol University	Imad,H.A., Phadungsombat,J., Nakayama,E.E., Suzuki,K., Ibrahim,A.M., Afaa,A., Azeema,A., Nazfa,A., Yazfa,A., Ahmed,A., Saeed,A., Waheed,A., Shareef,F., Islam,M.M., Anees,S.M., Saleem,S., Aroosha,A., Afzal,I., Leaungwutiwong,P., Piyaphanee,W., Phumratanaprapin,W. and Shioda,T.
EPI_ISL_17459182	Mahidol-Osaka Center for Infectious Diseases, Faculty of Tropical Medicine, Mahidol University	Mahidol-Osaka Center for Infectious Diseases, Faculty of Tropical Medicine, Mahidol University	Phadungsombat,J., Tuekprakhon,A., Cnops,L., Michiels,J., Van Den Berg,R., Nakayama,E.E., Shioda,T., Arien,K.K. and Huits,R.
EPI_ISL_17459183, EPI_ISL_17459184, EPI_ISL_17459185	Maladies Infectieuses et Vecteurs : Écologie, Génétique, Évolution et Contrôle	Maladies Infectieuses et Vecteurs : Écologie, Génétique, Évolution et Contrôle	Matthieu,F., Raphael,T.T., Chantale,P., Christ,G., Michel,M., Eric,M.L. and Pierre,B.
EPI_ISL_17459186	Manipal Institute of Virology	Manipal Institute of Virology	Arunkumar,G., Shilpa,C., Kavitha,K., Sudheesh,N., Sabeena,S., Sanjay,R. and Ujwal,S.
EPI_ISL_17459187	Massachusetts Institute of Technology	Massachusetts Institute of Technology	Villarreal,R.G., de Melo,E.F. Jr., Sena,B.F., Henkin,A.J., Ding,H., Levine,S., Marques,E.T., Magalhaes,T., Gehrke,L. and Bosch,I.
EPI_ISL_17459188, EPI_ISL_17459189, EPI_ISL_17459190, EPI_ISL_17459191, EPI_ISL_17459192, EPI_ISL_17459193	Medical University of Vienna	Medical University of Vienna	Aberle,S.W.
EPI_ISL_17459194	Ministry of Health	Ministry of Health	Lustig,Y.
EPI_ISL_17459195, EPI_ISL_17459196, EPI_ISL_17459197, EPI_ISL_17459198, EPI_ISL_17459199, EPI_ISL_17459200	Moderna	Moderna	CIARAMELLA,G., HIMANSU,S., HUANG,E.Y. and ZAKS,T.
EPI_ISL_17459201	Moderna	Moderna	CIARAMELLA,G., HUANG,E.Y., BAHL,K., ZAKS,T. and HIMANSU,S.
EPI_ISL_17459202, EPI_ISL_17459203, EPI_ISL_17459204, EPI_ISL_17459205, EPI_ISL_17459206, EPI_ISL_17459207, EPI_ISL_17459208, EPI_ISL_17459209, EPI_ISL_17459210, EPI_ISL_17459211, EPI_ISL_17459212, EPI_ISL_17459213			
see above	Moderna	Moderna	CIARAMELLA,G., HIMANSU,S., HUANG,E.Y. and ZAKS,T.
EPI_ISL_17459214, EPI_ISL_17459215, EPI_ISL_17459216, EPI_ISL_17459217, EPI_ISL_17459218, EPI_ISL_17459219, EPI_ISL_17459220	Moderna	Moderna	CIARAMELLA,G., HUANG,E.Y., BAHL,K., ZAKS,T. and HIMANSU,S.
EPI_ISL_17459221, EPI_ISL_17459222	Moderna	Moderna	CIARAMELLA,G., HIMANSU,S., HUANG,E.Y. and ZAKS,T.
EPI_ISL_17459223	Moderna	Moderna	CIARAMELLA,G., HUANG,E.Y., BAHL,K., ZAKS,T. and HIMANSU,S.
EPI_ISL_17459224, EPI_ISL_17459225, EPI_ISL_17459226, EPI_ISL_17459227	Moderna	Moderna	CIARAMELLA,G., HIMANSU,S., HUANG,E.Y. and ZAKS,T.
EPI_ISL_17459228	Moderna	Moderna	CIARAMELLA,G., HUANG,E.Y., BAHL,K., ZAKS,T. and HIMANSU,S.
EPI_ISL_17459229, EPI_ISL_17459230	Moderna	Moderna	CIARAMELLA,G., HIMANSU,S., HUANG,E.Y. and ZAKS,T.
EPI_ISL_17459231	Moderna	Moderna	CIARAMELLA,G., HUANG,E.Y., BAHL,K., ZAKS,T. and HIMANSU,S.
EPI_ISL_17459232	Molecular Virology Laboratory, Rajiv Gandhi Centre for Biotechnology	Molecular Virology Laboratory, Rajiv Gandhi Centre for Biotechnology	Sreekumar,E., Issac,A., Nair,S., Hariharan,R., Janki,M.B., Arathy,D.S., Regu,R., Mathew,T., Anoop,M., Niyas,K.P. and Pillai,M.R.
EPI_ISL_17459233	Molecular Virology Laboratory, Rajiv Gandhi Centre for Biotechnology	Molecular Virology Laboratory, Rajiv Gandhi Centre for Biotechnology	Niyas,K.P., Abraham,R., Nair,S., Anoop,M., Issac,A. and Sreekumar,E.
EPI_ISL_17459234	Molecular Virology Laboratory, Rajiv Gandhi Centre for Biotechnology	Molecular Virology Laboratory, Rajiv Gandhi Centre for Biotechnology	Abraham,R., Manakkadan,A., Mudaliar,P., Joseph,I., Sivakumar,K.C., Nair,R.R. and Sreekumar,E.
EPI_ISL_17459235, EPI_ISL_17459236	Molecular Virology Laboratory, Rajiv Gandhi Centre for Biotechnology	Molecular Virology Laboratory, Rajiv Gandhi Centre for Biotechnology	Sreekumar,E., Issac,A., Nair,S., Hariharan,R., Janki,M.B., Arathy,D.S., Regu,R., Mathew,T., Anoop,M., Niyas,K.P. and Pillai,M.R.
EPI_ISL_17459237, EPI_ISL_17459238, EPI_ISL_17459239, EPI_ISL_17459240, EPI_ISL_17459241	Molecular Virology Laboratory, Rajiv Gandhi Centre for Biotechnology	Molecular Virology Laboratory, Rajiv Gandhi Centre for Biotechnology	Abraham,R., Manakkadan,A., Mudaliar,P., Joseph,I., Sivakumar,K.C., Nair,R.R. and Sreekumar,E.
EPI_ISL_17459242	Molecular Virology Laboratory, Rajiv Gandhi Centre for Biotechnology	Molecular Virology Laboratory, Rajiv Gandhi Centre for Biotechnology	Sreekumar,E., Issac,A., Nair,S., Hariharan,R., Janki,M.B., Arathy,D.S., Regu,R., Mathew,T., Anoop,M., Niyas,K.P. and Pillai,M.R.
EPI_ISL_17459243, EPI_ISL_17459244	Molecular Virology Laboratory, Rajiv Gandhi Centre for Biotechnology	Molecular Virology Laboratory, Rajiv Gandhi Centre for Biotechnology	Abraham,R., Manakkadan,A., Mudaliar,P., Joseph,I., Sivakumar,K.C., Nair,R.R. and Sreekumar,E.
EPI_ISL_17459245	Molecular Virology Laboratory, Rajiv Gandhi Centre for Biotechnology	Molecular Virology Laboratory, Rajiv Gandhi Centre for Biotechnology	Niyas,K.P., Abraham,R., Nair,S., Anoop,M., Issac,A. and Sreekumar,E.
EPI_ISL_17459246, EPI_ISL_17459247, EPI_ISL_17459248, EPI_ISL_17459249, EPI_ISL_17459250, EPI_ISL_17459251	Molecular Virology Laboratory, Rajiv Gandhi Centre for Biotechnology	Molecular Virology Laboratory, Rajiv Gandhi Centre for Biotechnology	Abraham,R., Manakkadan,A., Mudaliar,P., Joseph,I., Sivakumar,K.C., Nair,R.R. and Sreekumar,E.
EPI_ISL_17459252	Molecular Virology Laboratory, Rajiv Gandhi Centre for Biotechnology	Molecular Virology Laboratory, Rajiv Gandhi Centre for Biotechnology	Niyas,K.P., Abraham,R., Nair,S., Anoop,M., Issac,A. and Sreekumar,E.
EPI_ISL_17459253	Molecular Virology Laboratory, Rajiv Gandhi Centre for Biotechnology	Molecular Virology Laboratory, Rajiv Gandhi Centre for Biotechnology	Sreekumar,E., Issac,A., Nair,S., Hariharan,R., Janki,M.B., Arathy,D.S., Regu,R., Mathew,T., Anoop,M., Niyas,K.P. and Pillai,M.R.
EPI_ISL_17459254, EPI_ISL_17459255	Molecular Virology Laboratory, Rajiv Gandhi Centre for Biotechnology	Molecular Virology Laboratory, Rajiv Gandhi Centre for Biotechnology	Niyas,K.P., Abraham,R., Nair,S., Anoop,M., Issac,A. and Sreekumar,E.
EPI_ISL_17459256, EPI_ISL_17459257, EPI_ISL_17459258, EPI_ISL_17459259, EPI_ISL_17459260, EPI_ISL_17459261, EPI_ISL_17459262, EPI_ISL_17459263, EPI_ISL_17459264	Molecular Virology Laboratory, Rajiv Gandhi Centre for Biotechnology	Molecular Virology Laboratory, Rajiv Gandhi Centre for Biotechnology	Abraham,R., Manakkadan,A., Mudaliar,P., Joseph,I., Sivakumar,K.C., Nair,R.R. and Sreekumar,E.
EPI_ISL_17459265	Molecular Virology Laboratory, Rajiv Gandhi Centre for Biotechnology	Molecular Virology Laboratory, Rajiv Gandhi Centre for Biotechnology	Sreekumar,E., Issac,A., Nair,S., Hariharan,R., Janki,M.B., Arathy,D.S., Regu,R., Mathew,T., Anoop,M., Niyas,K.P. and Pillai,M.R.
EPI_ISL_17459266, EPI_ISL_17459267, EPI_ISL_17459268, EPI_ISL_17459269	Nagasaki University	Nagasaki University	Tun,M.M., Thant,K.Z., Inoue,S., Nabeshima,T., Aoki,K., Kyaw,A.K., Myint,T., Tar,T., Maung,K.T., Hayasaka,D. and Morita,K.

EPI_ISL_17459270	Nagasaki University	Nagasaki University	Hasebe,F., Pandey,B.D., Parquet,M.D.C., Morita,K., Mathenge,E.G.M., Balasubramaniam,V., Saat,Z., Yusop,A., Sinniah,M., Natkunam,S. and Igarashi,A.
EPI_ISL_17459271	Nagasaki University	Nagasaki University	Ushijima,Y., Abe,H., Mbadinga,M.J.V.M., Nguema-Ondo,G., Bikangui,R., Agnandji,S.T., Lell,B. and Yasuda,J.
EPI_ISL_17459272, EPI_ISL_17459273, EPI_ISL_17459274	Nagasaki University	Nagasaki University	Hasebe,F., Pandey,B.D., Parquet,M.D.C., Morita,K., Mathenge,E.G.M., Balasubramaniam,V., Saat,Z., Yusop,A., Sinniah,M., Natkunam,S. and Igarashi,A.
EPI_ISL_17459275	Nagasaki University	Nagasaki University	Khan,A.H., Morita,K., Parquet Md Mdel,C., Hasebe,F., Mathenge,E.G. and Igarashi,A.
EPI_ISL_17459276	Najit Technologies	Najit Technologies	Amanna,I.J. and Slifka,M.K.
EPI_ISL_17459277, EPI_ISL_17459278, EPI_ISL_17459279, EPI_ISL_17459280, EPI_ISL_17459281, EPI_ISL_17459282, EPI_ISL_17459283, EPI_ISL_17459284, EPI_ISL_17459285, EPI_ISL_17459286, EPI_ISL_17459287			
see above	National Cancer Institute, Bone Marrow Transplantation Center, Laboratory of Oncovirology	National Cancer Institute, Bone Marrow Transplantation Center, Laboratory of Oncovirology	Familiar-Macedo,D., Gama,B.E., Emmel,V.E., Vera-Lozada,G., Abdelhay,E., Martins,I.S. and Hassan,R.
EPI_ISL_17459288, EPI_ISL_17459289	National Cancer Institute, Bone Marrow Transplantation Center, Laboratory of Oncovirology	National Cancer Institute, Bone Marrow Transplantation Center, Laboratory of Oncovirology	Krsticevic,F., Emmel,V., Ezpeleta,J., Murillo,J., Ervatti Gama,B., Vera-Lozada,G., Bulacio,P., Abdelhay,E., Tapia,E. and Hassan,R.
EPI_ISL_17459290	National Cancer Institute, Bone Marrow Transplantation Center, Laboratory of Oncovirology	National Cancer Institute, Bone Marrow Transplantation Center, Laboratory of Oncovirology	Familiar-Macedo,D., Gama,B.E., Emmel,V.E., Vera-Lozada,G., Abdelhay,E., Martins,I.S. and Hassan,R.
EPI_ISL_17459291, EPI_ISL_17459292, EPI_ISL_17459293, EPI_ISL_17459294, EPI_ISL_17459295, EPI_ISL_17459296, EPI_ISL_17459297, EPI_ISL_17459298	National Cancer Institute, Bone Marrow Transplantation Center, Laboratory of Oncovirology	National Cancer Institute, Bone Marrow Transplantation Center, Laboratory of Oncovirology	Krsticevic,F., Emmel,V., Ezpeleta,J., Murillo,J., Ervatti Gama,B., Vera-Lozada,G., Bulacio,P., Abdelhay,E., Tapia,E. and Hassan,R.
EPI_ISL_17459299, EPI_ISL_17459300, EPI_ISL_17459301, EPI_ISL_17459302, EPI_ISL_17459303, EPI_ISL_17459304, EPI_ISL_17459305, EPI_ISL_17459306	National Cancer Institute, Bone Marrow Transplantation Center, Laboratory of Oncovirology	National Cancer Institute, Bone Marrow Transplantation Center, Laboratory of Oncovirology	Familiar-Macedo,D., Gama,B.E., Emmel,V.E., Vera-Lozada,G., Abdelhay,E., Martins,I.S. and Hassan,R.
EPI_ISL_17459307	National Cancer Institute, Bone Marrow Transplantation Center, Laboratory of Oncovirology	National Cancer Institute, Bone Marrow Transplantation Center, Laboratory of Oncovirology	Krsticevic,F., Emmel,V., Ezpeleta,J., Murillo,J., Ervatti Gama,B., Vera-Lozada,G., Bulacio,P., Abdelhay,E., Tapia,E. and Hassan,R.
EPI_ISL_17459308	National Cancer Institute, Bone Marrow Transplantation Center, Laboratory of Oncovirology	National Cancer Institute, Bone Marrow Transplantation Center, Laboratory of Oncovirology	Familiar-Macedo,D., Gama,B.E., Emmel,V.E., Vera-Lozada,G., Abdelhay,E., Martins,I.S. and Hassan,R.
EPI_ISL_17459309, EPI_ISL_17459310	National Centre for Disease Control	National Centre for Disease Control	Singh,P., Sharma,P., Rizvi,M.A., Mittal,V. and Rai,A.
EPI_ISL_17459311	National Centre for Disease Control	National Centre for Disease Control	Singh,P., Sharma,P. and Rai,A.
EPI_ISL_17459312	National Centre for Disease Control	National Centre for Disease Control	Malik,P., Sharma,P., Mittal,V. and Rai,A.
EPI_ISL_17459313	National Centre for Disease Control	National Centre for Disease Control	Singh,P., Sharma,P. and Rai,A.
EPI_ISL_17459314, EPI_ISL_17459315	National Centre for Disease Control	National Centre for Disease Control	Malik,P., Sharma,P., Mittal,V. and Rai,A.
EPI_ISL_17459316	National Centre for Disease Control	National Centre for Disease Control	Singh,P., Sharma,P. and Rai,A.
EPI_ISL_17459317, EPI_ISL_17459318, EPI_ISL_17459319, EPI_ISL_17459320	National Centre for Disease Control	National Centre for Disease Control	Singh,P., Sharma,P., Rizvi,M.A., Mittal,V. and Rai,A.
EPI_ISL_17459321	National Centre for Disease Control	National Centre for Disease Control	Singh,P., Sharma,P. and Rai,A.
EPI_ISL_17459322, EPI_ISL_17459323, EPI_ISL_17459324, EPI_ISL_17459325, EPI_ISL_17459326, EPI_ISL_17459327, EPI_ISL_17459328, EPI_ISL_17459329	National Centre for Disease Control	National Centre for Disease Control	Singh,P., Sharma,P., Rizvi,M.A., Mittal,V. and Rai,A.
EPI_ISL_17459330	National Centre for Disease Control	National Centre for Disease Control	Singh,P., Sharma,P., Mittal,V. and Rai,A.
EPI_ISL_17459331	National Centre for Disease Control	National Centre for Disease Control	Singh,P., Sharma,P., Rizvi,M.A., Mittal,V. and Rai,A.
EPI_ISL_17459332, EPI_ISL_17459333	National Centre for Disease Control	National Centre for Disease Control	Singh,P., Thakur,S., Rawat,D.S., Rai,A. and Pasha,S.T.
EPI_ISL_17459334, EPI_ISL_17459335, EPI_ISL_17459336, EPI_ISL_17459337, EPI_ISL_17459338, EPI_ISL_17459339, EPI_ISL_17459340	National Centre for Disease Control	National Centre for Disease Control	Malik,P., Sharma,P., Mittal,V. and Rai,A.
EPI_ISL_17459341	National Centre for Disease Control	National Centre for Disease Control	Singh,P., Sharma,P., Rizvi,M.A., Mittal,V. and Rai,A.
EPI_ISL_17459342	National Centre for Disease Control	National Centre for Disease Control	Singh,P., Sharma,P., Mittal,V. and Rai,A.
EPI_ISL_17459343	National Centre for Disease Control	National Centre for Disease Control	Singh,P., Sharma,P., Rizvi,M.A., Mittal,V. and Rai,A.
EPI_ISL_17459344, EPI_ISL_17459345	National Centre for Disease Control	National Centre for Disease Control	Singh,P., Sharma,P. and Rai,A.
EPI_ISL_17459346	National Centre for Disease Control	National Centre for Disease Control	Singh,P., Thakur,S., Rawat,D.S., Rai,A. and Pasha,S.T.
EPI_ISL_17459347	National Centre for Disease Control	National Centre for Disease Control	Singh,P., Chaudhary,A., Kukreti,H., Thakur,S., Singh,S., Sethi,P.L., Anand,R., Pasha,S.T., Rai,A. and Rawat,D.S.
EPI_ISL_17459348	National Centre for Disease Control	National Centre for Disease Control	Singh,P., Chaudhary,A., Kukreti,H., Thakur,S., Singh,S., Sethi,P.L., Anand,R., Pasha,S.T. and Rai,A.
EPI_ISL_17459349	National Centre for Disease Control	National Centre for Disease Control	Singh,P., Chaudhary,A., Kukreti,H., Thakur,S., Singh,S., Sethi,P.L., Anand,R., Pasha,S.T., Rai,A. and Rawat,D.S.
EPI_ISL_17459350	National Centre for Disease Control	National Centre for Disease Control	Singh,P., Chaudhary,A., Thakur,S., Kukreti,H., Singh,S., Anand,R., Sethi,P.L., Rai,A., Rawat,D.S. and Pasha,S.T.
EPI_ISL_17459351, EPI_ISL_17459352, EPI_ISL_17459353	National Centre for Disease Control	National Centre for Disease Control	Singh,P., Thakur,S., Rawat,D.S., Rai,A. and Pasha,S.T.
EPI_ISL_17459354	National Centre for Disease Control	National Centre for Disease Control	Malik,P., Sharma,P., Mittal,V. and Rai,A.
EPI_ISL_17459355, EPI_ISL_17459356, EPI_ISL_17459357, EPI_ISL_17459358, EPI_ISL_17459359, EPI_ISL_17459360, EPI_ISL_17459361	National Centre for Disease Control	National Centre for Disease Control	Singh,P., Sharma,P., Rizvi,M.A., Mittal,V. and Rai,A.
EPI_ISL_17459362	National Centre for Disease Control	National Centre for Disease Control	Malik,P. Jr., Mittal,V. V and Rai,A. V.
EPI_ISL_17459363	National Centre for Disease Control	National Centre for Disease Control	Singh,P., Sharma,P., Rizvi,M.A., Mittal,V. and Rai,A.
EPI_ISL_17459364, EPI_ISL_17459365, EPI_ISL_17459366, EPI_ISL_17459367, EPI_ISL_17459368, EPI_ISL_17459369, EPI_ISL_17459370	National Centre for Disease Control	National Centre for Disease Control	Malik,P. Jr., Mittal,V. V and Rai,A. V.
EPI_ISL_17459371, EPI_ISL_17459372, EPI_ISL_17459373, EPI_ISL_17459374, EPI_ISL_17459375, EPI_ISL_17459376, EPI_ISL_17459377, EPI_ISL_17459378, EPI_ISL_17459379, EPI_ISL_17459380, EPI_ISL_17459381, EPI_ISL_17459382, EPI_ISL_17459383, EPI_ISL_17459384, EPI_ISL_17459385, EPI_ISL_17459386, EPI_ISL_17459387, EPI_ISL_17459388, EPI_ISL_17459389, EPI_ISL_17459390, EPI_ISL_17459391, EPI_ISL_17459392, EPI_ISL_17459393, EPI_ISL_17459394, EPI_ISL_17459395, EPI_ISL_17459396, EPI_ISL_17459397, EPI_ISL_17459398, EPI_ISL_17459399			
see above	National Centre for Disease Control	National Centre for Disease Control	Singh,P., Sharma,P., Rizvi,M.A., Mittal,V. and Rai,A.
EPI_ISL_17459400, EPI_ISL_17459401, EPI_ISL_17459402	National Centre for Disease Control	National Centre for Disease Control	Malik,P., Sharma,P., Mittal,V. and Rai,A.
EPI_ISL_17459403, EPI_ISL_17459404, EPI_ISL_17459405, EPI_ISL_17459406, EPI_ISL_17459407, EPI_ISL_17459408, EPI_ISL_17459409, EPI_ISL_17459410, EPI_ISL_17459411, EPI_ISL_17459412, EPI_ISL_17459413, EPI_ISL_17459414, EPI_ISL_17459415, EPI_ISL_17459416, EPI_ISL_17459417, EPI_ISL_17459418, EPI_ISL_17459419, EPI_ISL_17459420, EPI_ISL_17459421, EPI_ISL_17459422, EPI_ISL_17459423			
see above	National Centre for Disease Control	National Centre for Disease Control	Singh,P., Sharma,P., Rizvi,M.A., Mittal,V. and Rai,A.
EPI_ISL_17459424	National Centre for Disease Control	National Centre for Disease Control	Singh,P., Sharma,P. and Rai,A.
EPI_ISL_17459425, EPI_ISL_17459426	National Centre for Disease Control	National Centre for Disease Control	Malik,P. Jr., Mittal,V. V and Rai,A. V.
EPI_ISL_17459427, EPI_ISL_17459428, EPI_ISL_17459429, EPI_ISL_17459430, EPI_ISL_17459431, EPI_ISL_17459432, EPI_ISL_17459433, EPI_ISL_17459434, EPI_ISL_17459435, EPI_ISL_17459436, EPI_ISL_17459437, EPI_ISL_17459438, EPI_ISL_17459439, EPI_ISL_17459440, EPI_ISL_17459441, EPI_ISL_17459442, EPI_ISL_17459443, EPI_ISL_17459444			
see above	National Centre for Disease Control	National Centre for Disease Control	Singh,P., Sharma,P., Rizvi,M.A., Mittal,V. and Rai,A.
EPI_ISL_17459445	National Centre for Disease Control	National Centre for Disease Control	Malik,P. Jr., Mittal,V. V and Rai,A. V.
EPI_ISL_17459446	National Centre for Disease Control	National Centre for Disease Control	Singh,P., Sharma,P., Rizvi,M.A., Mittal,V. and Rai,A.
EPI_ISL_17459447, EPI_ISL_17459448	National Centre for Disease Control	National Centre for Disease Control	Singh,P., Sharma,P. and Rai,A.
EPI_ISL_17459449	National Centre for Disease Control	National Centre for Disease Control	Singh,P., Sharma,P., Rizvi,M.A., Mittal,V. and Rai,A.
EPI_ISL_17459450	National Centre for Disease Control	National Centre for Disease Control	Malik,P. Jr., Mittal,V. V and Rai,A. V.
EPI_ISL_17459451, EPI_ISL_17459452, EPI_ISL_17459453, EPI_ISL_17459454, EPI_ISL_17459455, EPI_ISL_17459456, EPI_ISL_17459457, EPI_ISL_17459458, EPI_ISL_17459459, EPI_ISL_17459460, EPI_ISL_17459461, EPI_ISL_17459462			
see above	National Centre for Disease Control	National Centre for Disease Control	Singh,P., Sharma,P., Rizvi,M.A., Mittal,V. and Rai,A.
EPI_ISL_17459463, EPI_ISL_17459464, EPI_ISL_17459465	National Centre for Disease Control	National Centre for Disease Control	Malik,P. Jr., Mittal,V. V and Rai,A. V.
EPI_ISL_17459466, EPI_ISL_17459467, EPI_ISL_17459468	National Centre for Disease Control	National Centre for Disease Control	Singh,P., Sharma,P., Rizvi,M.A., Mittal,V. and Rai,A.

EPI_ISL_17459469, EPI_ISL_17459470, EPI_ISL_17459471	National Centre for Disease Control	National Centre for Disease Control	Malik,P. Jr., Mittal,V. V and Rai,A. V.
EPI_ISL_17459472, EPI_ISL_17459473, EPI_ISL_17459474, EPI_ISL_17459475, EPI_ISL_17459476, EPI_ISL_17459477, EPI_ISL_17459478	National Centre for Disease Control	National Centre for Disease Control	Singh,P., Sharma,P. and Rai,A.
EPI_ISL_17459479, EPI_ISL_17459480	National Centre for Disease Control	National Centre for Disease Control	Malik,P., Sharma,P., Mittal,V. and Rai,A.
EPI_ISL_17459481	National Centre for Disease Control	National Centre for Disease Control	Singh,P., Sharma,P., Rizvi,M.A., Mittal,V. and Rai,A.
EPI_ISL_17459482	National Centre for Disease Control	National Centre for Disease Control	Malik,P. Jr., Mittal,V. V and Rai,A. V.
EPI_ISL_17459483, EPI_ISL_17459484, EPI_ISL_17459485	National Centre for Disease Control	National Centre for Disease Control	Singh,P., Sharma,P., Rizvi,M.A., Mittal,V. and Rai,A.
EPI_ISL_17459486	National Centre for Disease Control	National Centre for Disease Control	Singh,P., Sharma,P., Mittal,V. and Rai,A.
EPI_ISL_17459487	National Centre for Disease Control	National Centre for Disease Control	Malik,P. Jr., Mittal,V. V and Rai,A. V.
EPI_ISL_17459488	National Centre for Disease Control	National Centre for Disease Control	Singh,P., Sharma,P., Rizvi,M.A., Mittal,V. and Rai,A.
EPI_ISL_17459489, EPI_ISL_17459490, EPI_ISL_17459491, EPI_ISL_17459492	National Centre for Disease Control	National Centre for Disease Control	Malik,P. Jr., Mittal,V. V and Rai,A. V.
EPI_ISL_17459493, EPI_ISL_17459494, EPI_ISL_17459495, EPI_ISL_17459496, EPI_ISL_17459497, EPI_ISL_17459498, EPI_ISL_17459499, EPI_ISL_17459500, EPI_ISL_17459501, EPI_ISL_17459502, EPI_ISL_17459503, EPI_ISL_17459504, EPI_ISL_17459505, EPI_ISL_17459506, EPI_ISL_17459507, EPI_ISL_17459508, EPI_ISL_17459509, EPI_ISL_17459510, EPI_ISL_17459511, EPI_ISL_17459512, EPI_ISL_17459513, EPI_ISL_17459514, EPI_ISL_17459515, EPI_ISL_17459516, EPI_ISL_17459517			
see above	National Centre for Disease Control	National Centre for Disease Control	Singh,P., Sharma,P., Rizvi,M.A., Mittal,V. and Rai,A.
EPI_ISL_17459518	National Centre for Disease Control	National Centre for Disease Control	Singh,P., Sharma,P. and Rai,A.
EPI_ISL_17459519	National Centre for Disease Control	National Centre for Disease Control	Singh,P., Sharma,P., Rizvi,M.A., Mittal,V. and Rai,A.
EPI_ISL_17459520	National Centre for Disease Control	National Centre for Disease Control	Malik,P. Jr., Mittal,V. V and Rai,A. V.
EPI_ISL_17459521, EPI_ISL_17459522, EPI_ISL_17459523, EPI_ISL_17459524, EPI_ISL_17459525, EPI_ISL_17459526, EPI_ISL_17459527, EPI_ISL_17459528, EPI_ISL_17459529, EPI_ISL_17459530, EPI_ISL_17459531, EPI_ISL_17459532, EPI_ISL_17459533, EPI_ISL_17459534, EPI_ISL_17459535, EPI_ISL_17459536, EPI_ISL_17459537			
see above	National Centre for Disease Control	National Centre for Disease Control	Singh,P., Sharma,P., Rizvi,M.A., Mittal,V. and Rai,A.
EPI_ISL_17459538	National Centre for Disease Control	National Centre for Disease Control	Singh,P., Sharma,P. and Rai,A.
EPI_ISL_17459539, EPI_ISL_17459540, EPI_ISL_17459541, EPI_ISL_17459542, EPI_ISL_17459543, EPI_ISL_17459544, EPI_ISL_17459545, EPI_ISL_17459546, EPI_ISL_17459547, EPI_ISL_17459548, EPI_ISL_17459549, EPI_ISL_17459550, EPI_ISL_17459551, EPI_ISL_17459552, EPI_ISL_17459553, EPI_ISL_17459554, EPI_ISL_17459555, EPI_ISL_17459556, EPI_ISL_17459557, EPI_ISL_17459558, EPI_ISL_17459559, EPI_ISL_17459560, EPI_ISL_17459561, EPI_ISL_17459562, EPI_ISL_17459563, EPI_ISL_17459564, EPI_ISL_17459565, EPI_ISL_17459566, EPI_ISL_17459567, EPI_ISL_17459568, EPI_ISL_17459569, EPI_ISL_17459570, EPI_ISL_17459571, EPI_ISL_17459572, EPI_ISL_17459573, EPI_ISL_17459574, EPI_ISL_17459575, EPI_ISL_17459576, EPI_ISL_17459577, EPI_ISL_17459578, EPI_ISL_17459579, EPI_ISL_17459580, EPI_ISL_17459581, EPI_ISL_17459582			
see above	National Centre for Disease Control	National Centre for Disease Control	Singh,P., Sharma,P., Rizvi,M.A., Mittal,V. and Rai,A.
EPI_ISL_17459583	National Centre for Disease Control	National Centre for Disease Control	Singh,P., Sharma,P. and Rai,A.
EPI_ISL_17459584, EPI_ISL_17459585, EPI_ISL_17459586, EPI_ISL_17459587, EPI_ISL_17459588	National Centre for Disease Control	National Centre for Disease Control	Singh,P., Sharma,P., Rizvi,M.A., Mittal,V. and Rai,A.
EPI_ISL_17459589, EPI_ISL_17459590, EPI_ISL_17459591, EPI_ISL_17459592, EPI_ISL_17459593	National Centre for Disease Control	National Centre for Disease Control	Singh,P., Sharma,P., Mittal,V. and Rai,A.
EPI_ISL_17459594	National Centre for Disease Control	National Centre for Disease Control	Singh,P., Sharma,P., Rizvi,M.A., Mittal,V. and Rai,A.
EPI_ISL_17459595	National Centre for Disease Control	National Centre for Disease Control	Singh,P., Sharma,P., Mittal,V. and Rai,A.
EPI_ISL_17459596, EPI_ISL_17459597	National Centre for Disease Control	National Centre for Disease Control	Singh,P., Sharma,P., Rizvi,M.A., Mittal,V. and Rai,A.
EPI_ISL_17459598, EPI_ISL_17459599	National Centre for Disease Control	National Centre for Disease Control	Singh,P., Sharma,P., Mittal,V. and Rai,A.
EPI_ISL_17459600	National Centre for Disease Control	National Centre for Disease Control	Singh,P., Sharma,P., Rizvi,M.A., Mittal,V. and Rai,A.
EPI_ISL_17459601, EPI_ISL_17459602, EPI_ISL_17459603, EPI_ISL_17459604, EPI_ISL_17459605	National Centre for Disease Control	National Centre for Disease Control	Singh,P., Sharma,P., Mittal,V. and Rai,A.
EPI_ISL_17459606	National Centre for Disease Control	National Centre for Disease Control	Singh,P., Sharma,P., Rizvi,M.A., Mittal,V. and Rai,A.
EPI_ISL_17459607	National Centre for Disease Control	National Centre for Disease Control	Singh,P., Sharma,P., Mittal,V. and Rai,A.
EPI_ISL_17459608, EPI_ISL_17459609	National Centre for Disease Control	National Centre for Disease Control	Singh,P., Sharma,P., Rizvi,M.A., Mittal,V. and Rai,A.
EPI_ISL_17459610, EPI_ISL_17459611, EPI_ISL_17459612, EPI_ISL_17459613	National Centre for Disease Control	National Centre for Disease Control	Singh,P., Sharma,P., Mittal,V. and Rai,A.
EPI_ISL_17459614, EPI_ISL_17459615, EPI_ISL_17459616, EPI_ISL_17459617	National Centre for Disease Control	National Centre for Disease Control	Singh,P., Sharma,P., Rizvi,M.A., Mittal,V. and Rai,A.
EPI_ISL_17459618	National Centre for Disease Control	National Centre for Disease Control	Singh,P., Sharma,P. and Rai,A.
EPI_ISL_17459619, EPI_ISL_17459620, EPI_ISL_17459621	National Centre for Disease Control	National Centre for Disease Control	Singh,P., Sharma,P., Rizvi,M.A., Mittal,V. and Rai,A.
EPI_ISL_17459622	National Centre for Disease Control	National Centre for Disease Control	Malik,P., Sharma,P., Mittal,V. and Rai,A.
EPI_ISL_17459623, EPI_ISL_17459624	National Centre for Disease Control	National Centre for Disease Control	Singh,P., Sharma,P., Rizvi,M.A., Mittal,V. and Rai,A.
EPI_ISL_17459625	National Centre for Disease Control	National Centre for Disease Control	Malik,P. Jr., Mittal,V. V and Rai,A. V.
EPI_ISL_17459626, EPI_ISL_17459627, EPI_ISL_17459628	National Centre for Disease Control	National Centre for Disease Control	Malik,P., Sharma,P., Mittal,V. and Rai,A.
EPI_ISL_17459629	National Centre for Disease Control	National Centre for Disease Control	Singh,P., Sharma,P., Rizvi,M.A., Mittal,V. and Rai,A.
EPI_ISL_17459630	National Centre for Disease Control	National Centre for Disease Control	Singh,P., Sharma,P., Mittal,V. and Rai,A.
EPI_ISL_17459631	National Centre for Disease Control	National Centre for Disease Control	Malik,P., Sharma,P., Mittal,V. and Rai,A.
EPI_ISL_17459632	National Centre for Disease Control	National Centre for Disease Control	Singh,P., Sharma,P., Mittal,V. and Rai,A.
EPI_ISL_17459633	National Centre for Disease Control	National Centre for Disease Control	Singh,P., Sharma,P., Rizvi,M.A., Mittal,V. and Rai,A.
EPI_ISL_17459634	National Centre for Disease Control	National Centre for Disease Control	Singh,P., Sharma,P., Mittal,V. and Rai,A.
EPI_ISL_17459635	National Centre for Disease Control	National Centre for Disease Control	Singh,P., Sharma,P., Rizvi,M.A., Mittal,V. and Rai,A.
EPI_ISL_17459636	National Centre for Disease Control	National Centre for Disease Control	Malik,P., Sharma,P., Mittal,V. and Rai,A.
EPI_ISL_17459637	National Centre for Disease Control	National Centre for Disease Control	Singh,P., Sharma,P. and Rai,A.
EPI_ISL_17459638	National Centre for Disease Control	National Centre for Disease Control	Singh,P., Sharma,P., Rizvi,M.A., Mittal,V. and Rai,A.
EPI_ISL_17459639	National Centre for Disease Control	National Centre for Disease Control	Singh,P., Sharma,P. and Rai,A.
EPI_ISL_17459640	National Centre for Disease Control	National Centre for Disease Control	Singh,P., Sharma,P., Rizvi,M.A., Mittal,V. and Rai,A.
EPI_ISL_17459641, EPI_ISL_17459642, EPI_ISL_17459643, EPI_ISL_17459644, EPI_ISL_17459645, EPI_ISL_17459646, EPI_ISL_17459647, EPI_ISL_17459648, EPI_ISL_17459649, EPI_ISL_17459650, EPI_ISL_17459651, EPI_ISL_17459652, EPI_ISL_17459653, EPI_ISL_17459654, EPI_ISL_17459655, EPI_ISL_17459656, EPI_ISL_17459657, EPI_ISL_17459658, EPI_ISL_17459659, EPI_ISL_17459660, EPI_ISL_17459661, EPI_ISL_17459662, EPI_ISL_17459663, EPI_ISL_17459664, EPI_ISL_17459665, EPI_ISL_17459666, EPI_ISL_17459667, EPI_ISL_17459668, EPI_ISL_17459669, EPI_ISL_17459670, EPI_ISL_17459671, EPI_ISL_17459672, EPI_ISL_17459673, EPI_ISL_17459674, EPI_ISL_17459675, EPI_ISL_17459676			
see above	National Centre for Disease Control, Division of Biochemistry & Biotechnology	National Centre for Disease Control, Division of Biochemistry & Biotechnology	Singh,P., Sharma,P., Rawat,D.S., Mittal,V. and Rai,A.
EPI_ISL_17459677, EPI_ISL_17459678, EPI_ISL_17459679, EPI_ISL_17459680, EPI_ISL_17459681, EPI_ISL_17459682, EPI_ISL_17459683, EPI_ISL_17459684, EPI_ISL_17459685, EPI_ISL_17459686, EPI_ISL_17459687, EPI_ISL_17459688, EPI_ISL_17459689, EPI_ISL_17459690, EPI_ISL_17459691, EPI_ISL_17459692, EPI_ISL_17459693, EPI_ISL_17459694, EPI_ISL_17459695, EPI_ISL_17459696, EPI_ISL_17459697, EPI_ISL_17459698, EPI_ISL_17459699, EPI_ISL_17459700, EPI_ISL_17459701, EPI_ISL_17459702			
see above	National Centre for Disease Control, Division of Zoonosis	National Centre for Disease Control, Division of Zoonosis	Sharma,P., Singh,P., Chhabra,M. and Venkatesh,S.
EPI_ISL_17459703, EPI_ISL_17459704, EPI_ISL_17459705, EPI_ISL_17459706, EPI_ISL_17459707, EPI_ISL_17459708, EPI_ISL_17459709, EPI_ISL_17459710, EPI_ISL_17459711, EPI_ISL_17459712, EPI_ISL_17459713			
see above	National Centre for Disease Control, Division of Zoonosis	National Centre for Disease Control, Division of Zoonosis	Singh,P., Sharma,P., Chhabra,M., Mittal,V. and Rai,A.
EPI_ISL_17459714, EPI_ISL_17459715, EPI_ISL_17459716, EPI_ISL_17459717, EPI_ISL_17459718, EPI_ISL_17459719, EPI_ISL_17459720, EPI_ISL_17459721, EPI_ISL_17459722, EPI_ISL_17459723, EPI_ISL_17459724, EPI_ISL_17459725, EPI_ISL_17459726, EPI_ISL_17459727, EPI_ISL_17459728, EPI_ISL_17459729, EPI_ISL_17459730, EPI_ISL_17459731, EPI_ISL_17459732, EPI_ISL_17459733, EPI_ISL_17459734			
see above	National Centre for Diseases Control	National Centre for Diseases Control	Singh,P., Sharma,P., Chhabra,M., Mittal,V. and Rai,A.
EPI_ISL_17459735, EPI_ISL_17459736	National Environment Agency, Environmental Health Institute	National Environment Agency, Environmental Health Institute	Hapuarachchi,H.C., Bandara,K.B., Sumanadasa,S.D., Hapugoda,M.D., Lai,Y.L., Lee,K.S., Tan,L.K., Lin,R.T., Ng,L.F., Bucht,G., Abeyewickreme,W. and Ng,L.C.
EPI_ISL_17459737, EPI_ISL_17459738	National Environment Agency, Environmental Health Institute	National Environment Agency, Environmental Health Institute	Leo,Y.S., Chow,A.L., Tan,L.K., Lye,D.C., Lin,L. and Ng,L.C.
EPI_ISL_17459739	National Environment Agency, Environmental Health Institute	National Environment Agency, Environmental Health Institute	Hapuarachchi,H.C., Bandara,K.B., Sumanadasa,S.D., Hapugoda,M.D., Lai,Y.L., Lee,K.S., Tan,L.K., Lin,R.T., Ng,L.F., Bucht,G., Abeyewickreme,W. and Ng,L.C.

EPI_ISL_17459740	National Infection Service, Public Health England	National Infection Service, Public Health England	Carter,D.P., Furneaux,J., Small,J., Griffiths,K., Osborne,J., Petridou,C., Herdman,T., Vipond,R., Herbert,R., Aarons,E., Brooks,T. and Pullan,S.T.
EPI_ISL_17459741	National Infection Service, Public Health England	National Infection Service, Public Health England	Logue,C.H., Wise,E.L., Lewandowski,K.S., Liberty,L., Alexander,S., Baker,N.P., Russell,J.E. and Pullan,S.T.
EPI_ISL_17459742	National Institute For Communicable Diseases, Division of Zoonosis	National Institute For Communicable Diseases, Division of Zoonosis	Kukreti,H., Anand,R., Chabbra,M., Mittal,V., Pasha,S.T. and Rai,A.
EPI_ISL_17459743	National Institute of Allergy and Infectious Diseases	National Institute of Allergy and Infectious Diseases	Yek,C., Bohl,J., Lay,S., Chea,S., Oum,M. and Manning,J.E.
EPI_ISL_17459744	National Institute of Allergy and Infectious Diseases	National Institute of Allergy and Infectious Diseases	Lay,S., Chea,S., Bohl,J., Yek,C., Oum,M. and Manning,J.
EPI_ISL_17459745, EPI_ISL_17459746, EPI_ISL_17459747	National Institute of Allergy and Infectious Diseases	National Institute of Allergy and Infectious Diseases	Yek,C., Bohl,J., Lay,S., Chea,S., Oum,M. and Manning,J.E.
EPI_ISL_17459748	National Institute of Allergy and Infectious Diseases	National Institute of Allergy and Infectious Diseases	Yek,C., Bohl,J., Chea,S., Lay,S., Oum,M. and Manning,J.E.
EPI_ISL_17459749	National Institute of Allergy and Infectious Diseases	National Institute of Allergy and Infectious Diseases	Yek,C., Bohl,J., Lay,S., Chea,S., Oum,M. and Manning,J.E.
EPI_ISL_17459750	National Institute of Allergy and Infectious Diseases	National Institute of Allergy and Infectious Diseases	Yek,C., Lay,S., Bohl,J., Oum,M., Chea,S. and Manning,J.E.
EPI_ISL_17459751	National Institute of Allergy and Infectious Diseases	National Institute of Allergy and Infectious Diseases	Yek,C., Bohl,J., Chea,S., Lay,S., Oum,M. and Manning,J.E.
EPI_ISL_17459752	National Institute of Allergy and Infectious Diseases	National Institute of Allergy and Infectious Diseases	Yek,C., Bohl,J., Lay,S., Oum,M., Chea,S. and Manning,J.E.
EPI_ISL_17459753	National Institute of Allergy and Infectious Diseases	National Institute of Allergy and Infectious Diseases	Yek,C., Bohl,J., Lay,S., Chea,S., Oum,M. and Manning,J.E.
EPI_ISL_17459754	National Institute of Allergy and Infectious Diseases	National Institute of Allergy and Infectious Diseases	Yek,C., Bohl,J., Lay,S., Oum,M., Chea,S. and Manning,J.E.
EPI_ISL_17459755	National Institute of Allergy and Infectious Diseases	National Institute of Allergy and Infectious Diseases	Yek,C., Bohl,J., Lay,S., Chea,S., Oum,M. and Manning,J.E.
EPI_ISL_17459756	National Institute of Allergy and Infectious Diseases	National Institute of Allergy and Infectious Diseases	Yek,C., Bohl,J., Lay,S., Oum,M., Chea,S. and Manning,J.E.
EPI_ISL_17459757	National Institute of Allergy and Infectious Diseases	National Institute of Allergy and Infectious Diseases	Yek,C., Bohl,J., Chea,S., Lay,S., Oum,M. and Manning,J.E.
EPI_ISL_17459758	National Institute of Allergy and Infectious Diseases	National Institute of Allergy and Infectious Diseases	Yek,C., Bohl,J., Oum,M., Lay,S., Chea,S. and Manning,J.E.
EPI_ISL_17459759, EPI_ISL_17459760, EPI_ISL_17459761, EPI_ISL_17459762	National Institute of Allergy and Infectious Diseases	National Institute of Allergy and Infectious Diseases	Yek,C., Bohl,J., Lay,S., Chea,S., Oum,M. and Manning,J.E.
EPI_ISL_17459763	National Institute of Communicable Diseases, Biotechnology	National Institute of Communicable Diseases, Biotechnology	Kukreti,H., Chaudhary,A., Anand,R., Rawat,D.S., Pasha,S.T., Mittal,V. and Rai,A.
EPI_ISL_17459764	National Institute of Communicable Diseases, Biotechnology	National Institute of Communicable Diseases, Biotechnology	Kukreti,H., Chaudhary,A., Anand,R., Pasha,S.T., Mittal,V., Lal,S. and Rai,A.
EPI_ISL_17459765	National Institute of Communicable Diseases, Biotechnology	National Institute of Communicable Diseases, Biotechnology	Kukreti,H., Chaudhary,A., Anand,R., Rawat,D.S., Pasha,S.T., Mittal,V. and Rai,A.
EPI_ISL_17459766	National Institute of Communicable Diseases, Biotechnology	National Institute of Communicable Diseases, Biotechnology	Kukreti,H., Chaudhary,A., Anand,R., Rawat,D.S., Pasha,S.T., Mittal,V. and Mittal,A.
EPI_ISL_17459767	National Institute of Communicable Diseases, Biotechnology	National Institute of Communicable Diseases, Biotechnology	Kukreti,H., Chaudhary,A., Anand,R., Sangwan,N., Pasha,S.T., Rawat,D.S., Mittal,V., Chabra,M., Kumar,Y., Lal,S. and Rai,A.
EPI_ISL_17459768	National Institute of Communicable Diseases, Biotechnology	National Institute of Communicable Diseases, Biotechnology	Kukreti,H., Anand,A., Anand,R., Sangwan,N., Pasha,S.T., Rawat,D.S., Mittal,V., Chabra,M., Kumar,Y., Lal,S. and Rai,A.
EPI_ISL_17459769	National Institute of Communicable Diseases, Biotechnology	National Institute of Communicable Diseases, Biotechnology	Kukreti,H., Kumar,Y., Sangwan,N., Rawat,D.S., Pasha,S.T., Anand,R., Mittal,V., Chabra,M., Chaudhary,A., Lal,S. and Rai,A.
EPI_ISL_17459770	National Institute of Communicable Diseases, Biotechnology	National Institute of Communicable Diseases, Biotechnology	Kukreti,H., Chaudhary,A., Anand,R., Rawat,D.S., Pasha,S.T., Mittal,V. and Rai,A.
EPI_ISL_17459771	National Institute of Communicable Diseases, Biotechnology	National Institute of Communicable Diseases, Biotechnology	Kukreti,H., Anand,R., Kumar,Y., Sangwan,N., Pasha,S.T., Rawat,D.S., Mittal,V., Jairaj,P., Chaudhary,A., Lal,S. and Rai,A.
EPI_ISL_17459772, EPI_ISL_17459773, EPI_ISL_17459774, EPI_ISL_17459775	National Institute of Health	National Institute of Health	Badar,N.
EPI_ISL_17459776	National Institute of Health	National Institute of Health	Badar,N., Ikram,A., Mushtaq,N., Arshad,Y., Alam,M., Salman,M. and Qazi,J.
EPI_ISL_17459777, EPI_ISL_17459778	National Institute of Health	National Institute of Health	Badar,N.
EPI_ISL_17459779	National Institute of Health	National Institute of Health	Badar,N., Ikram,A., Mushtaq,N., Arshad,Y., Alam,M., Salman,M. and Qazi,J.
EPI_ISL_17459780, EPI_ISL_17459781, EPI_ISL_17459782, EPI_ISL_17459783	National Institute of Health	National Institute of Health	Badar,N.
EPI_ISL_17459784	National Institute of Health	National Institute of Health	Badar,N., Ikram,A., Mushtaq,N., Arshad,Y., Alam,M., Salman,M. and Qazi,J.
EPI_ISL_17459785, EPI_ISL_17459786, EPI_ISL_17459787, EPI_ISL_17459788, EPI_ISL_17459789, EPI_ISL_17459790, EPI_ISL_17459791, EPI_ISL_17459792, EPI_ISL_17459793, EPI_ISL_17459794, EPI_ISL_17459795, EPI_ISL_17459796, EPI_ISL_17459797, EPI_ISL_17459798, EPI_ISL_17459799, EPI_ISL_17459800, EPI_ISL_17459801, EPI_ISL_17459802, EPI_ISL_17459803, EPI_ISL_17459804, EPI_ISL_17459805, EPI_ISL_17459806, EPI_ISL_17459807, EPI_ISL_17459808, EPI_ISL_17459809, EPI_ISL_17459810, EPI_ISL_17459811, EPI_ISL_17459812, EPI_ISL_17459813, EPI_ISL_17459814			
see above	National Institute of Health	National Institute of Health	Badar,N.
EPI_ISL_17459815	National Institute of Health	National Institute of Health	Badar,N., Ikram,A., Mushtaq,N., Arshad,Y., Alam,M., Salman,M. and Qazi,J.
EPI_ISL_17459816, EPI_ISL_17459817, EPI_ISL_17459818, EPI_ISL_17459819, EPI_ISL_17459820, EPI_ISL_17459821, EPI_ISL_17459822	National Institute of Health	National Institute of Health	Badar,N.
EPI_ISL_17459823	National Institute of Health	National Institute of Health	Badar,N., Ikram,A., Mushtaq,N., Arshad,Y., Alam,M., Salman,M. and Qazi,J.
EPI_ISL_17459825, EPI_ISL_17459826, EPI_ISL_17459827, EPI_ISL_17459828, EPI_ISL_17459829, EPI_ISL_17459830, EPI_ISL_17459831, EPI_ISL_17459832, EPI_ISL_17459833, EPI_ISL_17459834, EPI_ISL_17459835, EPI_ISL_17459836, EPI_ISL_17459837, EPI_ISL_17459838, EPI_ISL_17459839, EPI_ISL_17459840, EPI_ISL_17459841, EPI_ISL_17459842, EPI_ISL_17459843, EPI_ISL_17459844, EPI_ISL_17459845, EPI_ISL_17459846, EPI_ISL_17459847, EPI_ISL_17459848, EPI_ISL_17459849			
see above	National Institute of Health	National Institute of Health	Badar,N.
EPI_ISL_17459850	National Institute of Health	National Institute of Health	Badar,N., Ikram,A., Mushtaq,N., Arshad,Y., Alam,M., Salman,M. and Qazi,J.
EPI_ISL_17459851, EPI_ISL_17459852, EPI_ISL_17459853, EPI_ISL_17459854, EPI_ISL_17459855, EPI_ISL_17459856, EPI_ISL_17459857, EPI_ISL_17459858, EPI_ISL_17459859, EPI_ISL_17459860, EPI_ISL_17459861, EPI_ISL_17459862, EPI_ISL_17459863, EPI_ISL_17459864			
see above	National Institute of Health	National Institute of Health	Badar,N.
EPI_ISL_17459865, EPI_ISL_17459866	National Institute of Health	National Institute of Health	Badar,N., Ikram,A., Mushtaq,N., Arshad,Y., Alam,M., Salman,M. and Qazi,J.
EPI_ISL_17459867, EPI_ISL_17459868, EPI_ISL_17459869	National Institute of Health	National Institute of Health	Badar,N.
EPI_ISL_17459870	National Institute of Health	National Institute of Health	Badar,N., Ikram,A., Mushtaq,N., Arshad,Y., Alam,M., Salman,M. and Qazi,J.
EPI_ISL_17459871, EPI_ISL_17459872, EPI_ISL_17459873, EPI_ISL_17459874, EPI_ISL_17459875, EPI_ISL_17459876, EPI_ISL_17459877, EPI_ISL_17459878, EPI_ISL_17459879, EPI_ISL_17459880, EPI_ISL_17459881, EPI_ISL_17459882, EPI_ISL_17459883			
see above	National Institute of Health	National Institute of Health	Badar,N.
EPI_ISL_17459884	National Institute of Health	National Institute of Health	Badar,N., Ikram,A., Mushtaq,N., Arshad,Y., Alam,M., Salman,M. and Qazi,J.
EPI_ISL_17459885, EPI_ISL_17459886	National Institute of Health	National Institute of Health	Badar,N.
EPI_ISL_17459887	National Institute of Health	National Institute of Health	Badar,N., Ikram,A., Mushtaq,N., Arshad,Y., Alam,M., Salman,M. and Qazi,J.
EPI_ISL_17459888	National Institute of Health	National Institute of Health	Badar,N.
EPI_ISL_17459889, EPI_ISL_17459890, EPI_ISL_17459891, EPI_ISL_17459892, EPI_ISL_17459893, EPI_ISL_17459894, EPI_ISL_17459895, EPI_ISL_17459896, EPI_ISL_17459897, EPI_ISL_17459898, EPI_ISL_17459899, EPI_ISL_17459900, EPI_ISL_17459901, EPI_ISL_17459902, EPI_ISL_17459903, EPI_ISL_17459904, EPI_ISL_17459905, EPI_ISL_17459906, EPI_ISL_17459907, EPI_ISL_17459908, EPI_ISL_17459909, EPI_ISL_17459910, EPI_ISL_17459911, EPI_ISL_17459912, EPI_ISL_17459913, EPI_ISL_17459914, EPI_ISL_17459915, EPI_ISL_17459916			
see above	National Institute of Health Research and	National Institute of Health Research and	Sembiring,M.M. and Subangkit,S.

	Development	Development	
EPI_ISL_17459917, EPI_ISL_17459918, EPI_ISL_17459919, EPI_ISL_17459920	National Institute of Infectious Diseases	National Institute of Infectious Diseases	Nakayama,E., Tajima,S., Kotaki,A. and Tomohiko,T.
EPI_ISL_17459921, EPI_ISL_17459922	National Institute of Infectious Diseases	National Institute of Infectious Diseases	Lim,C.K., Nishibori,T., Watanabe,K., Ito,M., Kotaki,A., Tanaka,K., Kurane,I. and Takasaki,T.
EPI_ISL_17459923, EPI_ISL_17459924	National Institute of Infectious Diseases	National Institute of Infectious Diseases	Nakayama,E., Maeki,T., Tajima,S., Taniguchi,S., Lim,C.K., Nakamoto,T. and Kutsuna,S.
EPI_ISL_17459925	National Institute of Infectious Diseases	National Institute of Infectious Diseases	Tsuboi,M., Kutsuna,S., Kato,Y., Nakayama,E., Shibasaki,K., Tajima,S., Takasaki,T., Katanami,Y., Yamamoto,K., Takeshita,N., Hayakawa,K., Kanagawa,S. and Ohmagari,N.
EPI_ISL_17459926, EPI_ISL_17459927, EPI_ISL_17459928, EPI_ISL_17459929, EPI_ISL_17459930, EPI_ISL_17459931, EPI_ISL_17459932, EPI_ISL_17459933, EPI_ISL_17459934	National Institute of Infectious Diseases	National Institute of Infectious Diseases	Nakayama,E., Tajima,S., Kotaki,A. and Tomohiko,T.
EPI_ISL_17459935	National Institute of Infectious Diseases Lazzaro Spallanzani, Istituto di Ricovero e Cura a Carattere Scientifico (INMI-IRCCS)	National Institute of Infectious Diseases Lazzaro Spallanzani, Istituto di Ricovero e Cura a Carattere Scientifico (INMI-IRCCS)	Carletti,F., Castilletti,C., Meschi,S., Colavita,F., Lalle,E., Bordi,L., Nicastri,E., Vairo,F., Di Lallo,D., Panella,V., Di Caro,A., Ippolito,G. and Capobianchi,M.R.
EPI_ISL_17459936	National Institute of Infectious Diseases Lazzaro Spallanzani, Istituto di Ricovero e Cura a Carattere Scientifico	National Institute of Infectious Diseases Lazzaro Spallanzani, Istituto di Ricovero e Cura a Carattere Scientifico	Mombouli,J.V., Giombini,E., Carletti,F., Rueca,M., Messina,F., Gruber,C., Castilletti,C., Goma Nkoua,C., Vairo,F., Colavita,F., Lanini,S., Montaldo,C., Iannetta,M., Haider,N., Peko,S.M., Diafouka-Diatela,S., Kija Tungu,P., Ippolito,G., Zumla,A., Ntoumi,F. and Capobianchi,M.R.
EPI_ISL_17459937, EPI_ISL_17459938	National Institute of Infectious Diseases Lazzaro Spallanzani, Istituto di Ricovero e Cura a Carattere Scientifico	National Institute of Infectious Diseases Lazzaro Spallanzani, Istituto di Ricovero e Cura a Carattere Scientifico	Fusco,F.M., Puro,V., Di Caro,A., Nicastri,E., Carannante,N., Faella,F.S., Barzon,L., Di Cesare,S., Palu,G., Capobianchi,M.R. and Ippolito,G.
EPI_ISL_17459939	National Institute of Infectious Diseases Lazzaro Spallanzani, Istituto di Ricovero e Cura a Carattere Scientifico	National Institute of Infectious Diseases Lazzaro Spallanzani, Istituto di Ricovero e Cura a Carattere Scientifico	Bordi,L., Castilletti,C., Chiappini,R., Sambri,V., Cavrini,F., Capobianchi,M.R., Di Caro,A. and Carletti,F.
EPI_ISL_17459940	National Institute of Infectious Diseases Lazzaro Spallanzani, Istituto di Ricovero e Cura a Carattere Scientifico	National Institute of Infectious Diseases Lazzaro Spallanzani, Istituto di Ricovero e Cura a Carattere Scientifico	Fusco,F.M., Puro,V., Di Caro,A., Nicastri,E., Carannante,N., Faella,F.S., Barzon,L., Di Cesare,S., Palu,G., Capobianchi,M.R. and Ippolito,G.
EPI_ISL_17459941	National Institute of Infectious Diseases Lazzaro Spallanzani, Istituto di Ricovero e Cura a Carattere Scientifico	National Institute of Infectious Diseases Lazzaro Spallanzani, Istituto di Ricovero e Cura a Carattere Scientifico	Bordi,L., Castilletti,C., Chiappini,R., Sambri,V., Cavrini,F., Capobianchi,M.R., Di Caro,A. and Carletti,F.
EPI_ISL_17459942	National Institute of Infectious Diseases Lazzaro Spallanzani, Istituto di Ricovero e Cura a Carattere Scientifico	National Institute of Infectious Diseases Lazzaro Spallanzani, Istituto di Ricovero e Cura a Carattere Scientifico	Mombouli,J.V., Carletti,F., Castilletti,C., Goma Nkoua,C., Rueca,M., Vairo,F., Colavita,F., Lanini,S., Montaldo,C., Iannetta,M., Haider,N., Peko,S.M., Diafouka-Diatela,S., Kija Tungu,P., Ippolito,G., Zumla,A., Ntoumi,F. and Capobianchi,M.R.
EPI_ISL_17459943	National Institute of Infectious Diseases Lazzaro Spallanzani, Istituto di Ricovero e Cura a Carattere Scientifico	National Institute of Infectious Diseases Lazzaro Spallanzani, Istituto di Ricovero e Cura a Carattere Scientifico	Fusco,F.M., Puro,V., Di Caro,A., Nicastri,E., Carannante,N., Faella,F.S., Barzon,L., Di Cesare,S., Palu,G., Capobianchi,M.R. and Ippolito,G.
EPI_ISL_17459944	National Institute of Infectious Diseases Lazzaro Spallanzani, Istituto di Ricovero e Cura a Carattere Scientifico (INMI-IRCCS)	National Institute of Infectious Diseases Lazzaro Spallanzani, Istituto di Ricovero e Cura a Carattere Scientifico (INMI-IRCCS)	Colavita,F., Vita,S., Lalle,E., Carletti,F., Bordi,L., Vincenti,D., Pozzetto,I., Aiuti,M., Vairo,F., Capobianchi,M.R., Lichtner,M. and Castilletti,C.
EPI_ISL_17459945	National Institute of Infectious Diseases Lazzaro Spallanzani, Istituto di Ricovero e Cura a Carattere Scientifico	National Institute of Infectious Diseases Lazzaro Spallanzani, Istituto di Ricovero e Cura a Carattere Scientifico	Fusco,F.M., Puro,V., Di Caro,A., Nicastri,E., Carannante,N., Faella,F.S., Barzon,L., Di Cesare,S., Palu,G., Capobianchi,M.R. and Ippolito,G.
EPI_ISL_17459946	National Institute of Infectious Diseases Lazzaro Spallanzani, Istituto di Ricovero e Cura a Carattere Scientifico (INMI-IRCCS)	National Institute of Infectious Diseases Lazzaro Spallanzani, Istituto di Ricovero e Cura a Carattere Scientifico (INMI-IRCCS)	Carletti,F., Lalle,E., Castilletti,C. and Capobianchi,M.R.
EPI_ISL_17459947	National Institute of Infectious Diseases Lazzaro Spallanzani, Istituto di Ricovero e Cura a Carattere Scientifico	National Institute of Infectious Diseases Lazzaro Spallanzani, Istituto di Ricovero e Cura a Carattere Scientifico	Fusco,F.M., Puro,V., Di Caro,A., Nicastri,E., Carannante,N., Faella,F.S., Barzon,L., Di Cesare,S., Palu,G., Capobianchi,M.R. and Ippolito,G.
EPI_ISL_17459948	National Institute of Infectious Diseases Lazzaro Spallanzani, Istituto di Ricovero e Cura a Carattere Scientifico	National Institute of Infectious Diseases Lazzaro Spallanzani, Istituto di Ricovero e Cura a Carattere Scientifico	Bordi,L., Castilletti,C., Chiappini,R., Sambri,V., Cavrini,F., Capobianchi,M.R., Di Caro,A. and Carletti,F.
EPI_ISL_17459949, EPI_ISL_17459950	National Institute of Infectious Diseases Lazzaro Spallanzani, Istituto di Ricovero e Cura a Carattere Scientifico	National Institute of Infectious Diseases Lazzaro Spallanzani, Istituto di Ricovero e Cura a Carattere Scientifico	Fusco,F.M., Puro,V., Di Caro,A., Nicastri,E., Carannante,N., Faella,F.S., Barzon,L., Di Cesare,S., Palu,G., Capobianchi,M.R. and Ippolito,G.
EPI_ISL_17459951	National Institute of Infectious Diseases Lazzaro Spallanzani, Istituto di Ricovero e Cura a Carattere Scientifico (INMI-IRCCS)	National Institute of Infectious Diseases Lazzaro Spallanzani, Istituto di Ricovero e Cura a Carattere Scientifico (INMI-IRCCS)	Carletti,F., Marsella,P., Castilletti,C., Meschi,S., Colavita,F., Lalle,E., Bordi,L., Di Caro,A., Nicastri,E., Scognamiglio,P., Lanini,S., Vairo,F., Di Lallo,D., Panella,V., Ippolito,G. and Capobianchi,M.R.
EPI_ISL_17459952	National Institute of Infectious Diseases Lazzaro Spallanzani, Istituto di Ricovero e Cura a Carattere Scientifico (INMI-IRCCS)	National Institute of Infectious Diseases Lazzaro Spallanzani, Istituto di Ricovero e Cura a Carattere Scientifico (INMI-IRCCS)	Carletti,F., Castilletti,C., Meschi,S., Colavita,F., Lalle,E., Bordi,L., Nicastri,E., Vairo,F., Di Lallo,D., Panella,V., Di Caro,A., Ippolito,G. and Capobianchi,M.R.
EPI_ISL_17459953, EPI_ISL_17459954, EPI_ISL_17459955	National Institute of Infectious Diseases Lazzaro Spallanzani, Istituto di Ricovero e Cura a Carattere Scientifico	National Institute of Infectious Diseases Lazzaro Spallanzani, Istituto di Ricovero e Cura a Carattere Scientifico	Fusco,F.M., Puro,V., Di Caro,A., Nicastri,E., Carannante,N., Faella,F.S., Barzon,L., Di Cesare,S., Palu,G., Capobianchi,M.R. and Ippolito,G.
EPI_ISL_17459956	National Institute of Infectious Diseases Lazzaro Spallanzani, Istituto di Ricovero e Cura a Carattere Scientifico	National Institute of Infectious Diseases Lazzaro Spallanzani, Istituto di Ricovero e Cura a Carattere Scientifico	Bordi,L., Castilletti,C., Chiappini,R., Sambri,V., Cavrini,F., Capobianchi,M.R., Di Caro,A. and Carletti,F.
EPI_ISL_17459957	National Institute of Infectious Diseases Lazzaro Spallanzani, Istituto di Ricovero e Cura a Carattere Scientifico (INMI-IRCCS)	National Institute of Infectious Diseases Lazzaro Spallanzani, Istituto di Ricovero e Cura a Carattere Scientifico (INMI-IRCCS)	Carletti,F., Castilletti,C., Meschi,S., Colavita,F., Lalle,E., Bordi,L., Nicastri,E., Vairo,F., Di Lallo,D., Panella,V., Di Caro,A., Ippolito,G. and Capobianchi,M.R.
EPI_ISL_17459958	National Institute of Malaria Research	National Institute of Malaria Research	Kumar,S.
EPI_ISL_17459959, EPI_ISL_17459960, EPI_ISL_17459961, EPI_ISL_17459962, EPI_ISL_17459963, EPI_ISL_17459964, EPI_ISL_17459965, EPI_ISL_17459966, EPI_ISL_17459967, EPI_ISL_17459968, EPI_ISL_17459969, EPI_ISL_17459970, EPI_ISL_17459971, EPI_ISL_17459972, EPI_ISL_17459973, EPI_ISL_17459974, EPI_ISL_17459975, EPI_ISL_17459976, EPI_ISL_17459977, EPI_ISL_17459978			
see above	National Institute of Mental Health and Neurosciences	National Institute of Mental Health and Neurosciences	Pk,H., Pattabiraman,C., Rao,D., Reddy,V. and Mani,R.
EPI_ISL_17459979	National Institute of Research in Tribal Health	National Institute of Research in Tribal Health	Shukla,M.K. and Barde,P.V.
EPI_ISL_17459980	National Institute of Research in Tribal Health	National Institute of Research in Tribal Health	Barde,P.V. Sr., Shukla,M.K. Jr., Bharti,P.K. Sr., Jatav,J.K. Jr., Kori,B.K. Jr. and Singh,N. Sr.
EPI_ISL_17459981, EPI_ISL_17459982, EPI_ISL_17459983	National Institute of Research in Tribal Health	National Institute of Research in Tribal Health	Shukla,M.K. and Barde,P.V.
EPI_ISL_17459984, EPI_ISL_17459985, EPI_ISL_17459986, EPI_ISL_17459987	National Institute of Virology	National Institute of Virology	Arankalle,V.A.
EPI_ISL_17459988	National Institute of Virology	National Institute of Virology	Patil,J.A. and More,A.
EPI_ISL_17459989, EPI_ISL_17459990, EPI_ISL_17459991, EPI_ISL_17459992, EPI_ISL_17459993, EPI_ISL_17459994, EPI_ISL_17459995	National Institute of Virology	National Institute of Virology	Arankalle,V.A.
EPI_ISL_17459996	National Institute of Virology	National Institute of Virology	More,A., Patil,J. and Newase,P.K.
EPI_ISL_17459997, EPI_ISL_17459998	National Institute of Virology	National Institute of Virology	Arankalle,V.A.
EPI_ISL_17459999, EPI_ISL_17460000	National Institute of Virology	National Institute of Virology	More,A., Patil,J. and Newase,P.K.
EPI_ISL_17460001	National Institute of Virology	National Institute of Virology	Ranadive,S.N.
EPI_ISL_17460002, EPI_ISL_17460003	National Institute of Virology	National Institute of Virology	More,A., Patil,J. and Newase,P.K.
EPI_ISL_17460004	National Institute of Virology	National Institute of Virology	Arankalle,V.A.
EPI_ISL_17460005	National Institute of Virology	National Institute of Virology	More,A., Patil,J. and Newase,P.K.
EPI_ISL_17460006, EPI_ISL_17460007	National Institute of Virology	National Institute of Virology	Arankalle,V.A.
EPI_ISL_17460008, EPI_ISL_17460009, EPI_ISL_17460010, EPI_ISL_17460011	National Institute of Virology	National Institute of Virology	Cherian,S.S., Walimbe,A.M., Jadhav,S.M., Gandhe,S.S., Hundekar,S.L., Mishra,A.C. and Arankalle,V.A.
EPI_ISL_17460012	National Institute of Virology	National Institute of Virology	Arankalle,V.A.
EPI_ISL_17460013	National Institute of Virology	National Institute of Virology	More,A., Patil,J. and Newase,P.K.

EPI_ISL_17460014	National Institute of Virology	National Institute of Virology	Arankalle,V.A.
EPI_ISL_17460015	National Institute of Virology	National Institute of Virology	More,A., Patil,J. and Newase,P.K.
EPI_ISL_17460016	National Institute of Virology	National Institute of Virology	Paingankar,M.S., Gokhale,M.D., Vaishnav,K.G. and Shah,P.S.
EPI_ISL_17460017, EPI_ISL_17460018, EPI_ISL_17460019, EPI_ISL_17460020, EPI_ISL_17460021, EPI_ISL_17460022, EPI_ISL_17460023, EPI_ISL_17460024, EPI_ISL_17460025, EPI_ISL_17460026, EPI_ISL_17460027, EPI_ISL_17460028, EPI_ISL_17460029, EPI_ISL_17460030, EPI_ISL_17460031, EPI_ISL_17460032, EPI_ISL_17460033, EPI_ISL_17460034			
see above	National Institute of Virology	National Institute of Virology	Arankalle,V.A.
EPI_ISL_17460035, EPI_ISL_17460036, EPI_ISL_17460037, EPI_ISL_17460038	National Institute of Virology	National Institute of Virology	Yergolkar,P.N., Tandale,B.V., Arankalle,V.A., Sathe,P.S., Sudeep,A.B., Gandhe,S.S., Gokhle,M.D., Jacob,G.P., Hundekar,S.L. and Mishra,A.C.
EPI_ISL_17460039, EPI_ISL_17460040	National Institute of Virology	National Institute of Virology	Arankalle,V.A., Shrivastava,S., Cherian,S., Gunjikar,R.S., Walimbe,A.M., Jadhav,S.M., Sudeep,A.B. and Mishra,A.C.
EPI_ISL_17460041	National Institute of Virology	National Institute of Virology	Patil,J.A.
EPI_ISL_17460042	National Institute of Virology	National Institute of Virology	Arankalle,V.A.
EPI_ISL_17460043, EPI_ISL_17460044, EPI_ISL_17460045, EPI_ISL_17460046, EPI_ISL_17460047, EPI_ISL_17460048	National Institute of Virology	National Institute of Virology	Patil,J.A.
EPI_ISL_17460049, EPI_ISL_17460050, EPI_ISL_17460051, EPI_ISL_17460052, EPI_ISL_17460053, EPI_ISL_17460054, EPI_ISL_17460055, EPI_ISL_17460056, EPI_ISL_17460057	National Institute of Virology	National Institute of Virology	Arankalle,V.A.
EPI_ISL_17460058	National Institute of Virology	National Institute of Virology	Arankalle,V.A., Shrivastava,S., Cherian,S., Gunjikar,R.S., Walimbe,A.M., Jadhav,S.M., Sudeep,A.B. and Mishra,A.C.
EPI_ISL_17460059, EPI_ISL_17460060, EPI_ISL_17460061, EPI_ISL_17460062, EPI_ISL_17460063, EPI_ISL_17460064, EPI_ISL_17460065, EPI_ISL_17460066, EPI_ISL_17460067, EPI_ISL_17460068, EPI_ISL_17460069			
see above	National Institute of Virology	National Institute of Virology	Arankalle,V.A.
EPI_ISL_17460070	National Institute of Virology	National Institute of Virology	Yergolkar,P.N., Tandale,B.V., Arankalle,V.A., Sathe,P.S., Sudeep,A.B., Gandhe,S.S., Gokhle,M.D., Jacob,G.P., Hundekar,S.L. and Mishra,A.C.
EPI_ISL_17460071	National Institute of Virology	National Institute of Virology	Cherian,S.S., Walimbe,A.M., Jadhav,S.M., Gandhe,S.S., Hundekar,S.L., Mishra,A.C. and Arankalle,V.A.
EPI_ISL_17460072	National Institute of Virology	National Institute of Virology	Patil,J.A.
EPI_ISL_17460073, EPI_ISL_17460074	National Institute of Virology	National Institute of Virology	Arankalle,V.A.
EPI_ISL_17460075	National Institute of Virology	National Institute of Virology	Anukumar,B., Asia,D.T., Aishwarya,B., Jijo,K., Nikil,T.L., Sugunan,A.P., Sreelekha,K.P. and Kunjila,K.
EPI_ISL_17460076, EPI_ISL_17460077, EPI_ISL_17460078	National Institute of Virology	National Institute of Virology	Arankalle,V.A.
EPI_ISL_17460079	National Institute of Virology	National Institute of Virology	Patil,J., More,A., Patil,P., Jadhav,S., Newase,P., Agarwal,M., Amdekar,S., Raut,C.G., Parashar,D. and Cherian,S.S.
EPI_ISL_17460080, EPI_ISL_17460081, EPI_ISL_17460082, EPI_ISL_17460083, EPI_ISL_17460084, EPI_ISL_17460085	National Institute of Virology	National Institute of Virology	Arankalle,V.A.
EPI_ISL_17460086, EPI_ISL_17460087	National Institute of Virology	National Institute of Virology	Patil,J.A.
EPI_ISL_17460088	National Institute of Virology	National Institute of Virology	Patil,J., More,A., Patil,P., Jadhav,S., Newase,P., Agarwal,M., Amdekar,S., Raut,C.G., Parashar,D. and Cherian,S.S.
EPI_ISL_17460089, EPI_ISL_17460090, EPI_ISL_17460091	National Institute of Virology	National Institute of Virology	Yergolkar,P.N., Tandale,B.V., Arankalle,V.A., Sathe,P.S., Sudeep,A.B., Gandhe,S.S., Gokhle,M.D., Jacob,G.P., Hundekar,S.L. and Mishra,A.C.
EPI_ISL_17460092, EPI_ISL_17460093, EPI_ISL_17460094, EPI_ISL_17460095, EPI_ISL_17460096, EPI_ISL_17460097, EPI_ISL_17460098, EPI_ISL_17460099, EPI_ISL_17460100, EPI_ISL_17460101	National Institute of Virology	National Institute of Virology	Arankalle,V.A.
EPI_ISL_17460102, EPI_ISL_17460103	National Institute of Virology	National Institute of Virology	Yergolkar,P.N., Tandale,B.V., Arankalle,V.A., Sathe,P.S., Sudeep,A.B., Gandhe,S.S., Gokhle,M.D., Jacob,G.P., Hundekar,S.L. and Mishra,A.C.
EPI_ISL_17460104, EPI_ISL_17460105	National Institute of Virology	National Institute of Virology	More,A., Patil,J. and Newase,P.K.
EPI_ISL_17460106, EPI_ISL_17460107, EPI_ISL_17460108, EPI_ISL_17460109, EPI_ISL_17460110, EPI_ISL_17460111, EPI_ISL_17460112, EPI_ISL_17460113	National Institute of Virology	National Institute of Virology	Arankalle,V.A.
EPI_ISL_17460114	National Institute of Virology	National Institute of Virology	Anukumar,B., Asia,D.T., Aishwarya,B., Jijo,K., Nikil,T.L., Sugunan,A.P., Sreelekha,K.P. and Kunjila,K.
EPI_ISL_17460115, EPI_ISL_17460116, EPI_ISL_17460117	National Institute of Virology	National Institute of Virology	Arankalle,V.A.
EPI_ISL_17460118, EPI_ISL_17460119, EPI_ISL_17460120	National Institute of Virology	National Institute of Virology	Yergolkar,P.N., Tandale,B.V., Arankalle,V.A., Sathe,P.S., Sudeep,A.B., Gandhe,S.S., Gokhle,M.D., Jacob,G.P., Hundekar,S.L. and Mishra,A.C.
EPI_ISL_17460121, EPI_ISL_17460122, EPI_ISL_17460123, EPI_ISL_17460124	National Institute of Virology	National Institute of Virology	Arankalle,V.A.
EPI_ISL_17460125	National Institute of Virology	National Institute of Virology	Gurav,Y.K., Gopalkrishna,V., Shah,P.S., Patil,D.R., Mishra,M., Paingankar,M.S., Singh,A., Sathe,P.S. and Mishra,A.C.
EPI_ISL_17460126	National Institute of Virology	National Institute of Virology	Yergolkar,P.N., Tandale,B.V., Arankalle,V.A., Sathe,P.S., Sudeep,A.B., Gandhe,S.S., Gokhle,M.D., Jacob,G.P., Hundekar,S.L. and Mishra,A.C.
EPI_ISL_17460127, EPI_ISL_17460128	National Institute of Virology	National Institute of Virology	Arankalle,V.A.
EPI_ISL_17460129	National Institute of Virology	National Institute of Virology	Arankalle,V.A., Shrivastava,S., Cherian,S., Gunjikar,R.S., Walimbe,A.M., Jadhav,S.M., Sudeep,A.B. and Mishra,A.C.
EPI_ISL_17460130, EPI_ISL_17460131, EPI_ISL_17460132	National Institute of Virology	National Institute of Virology	Cherian,S.S., Walimbe,A.M., Jadhav,S.M., Gandhe,S.S., Hundekar,S.L., Mishra,A.C. and Arankalle,V.A.
EPI_ISL_17460133, EPI_ISL_17460134	National Institute of Virology	National Institute of Virology	Arankalle,V.A.
EPI_ISL_17460135	National Institute of Virology	National Institute of Virology	Arankalle,V.A., Shrivastava,S., Cherian,S., Gunjikar,R.S., Walimbe,A.M., Jadhav,S.M., Sudeep,A.B. and Mishra,A.C.
EPI_ISL_17460136	National Institute of Virology	National Institute of Virology	Gurav,Y.K., Gopalkrishna,V., Shah,P.S., Patil,D.R., Mishra,M., Paingankar,M.S., Singh,A., Sathe,P.S. and Mishra,A.C.
EPI_ISL_17460137, EPI_ISL_17460138, EPI_ISL_17460139, EPI_ISL_17460140	National Institute of Virology	National Institute of Virology	Arankalle,V.A.
EPI_ISL_17460141, EPI_ISL_17460142, EPI_ISL_17460143, EPI_ISL_17460144	National Institute of Virology	National Institute of Virology	Yergolkar,P.N., Tandale,B.V., Arankalle,V.A., Sathe,P.S., Sudeep,A.B., Gandhe,S.S., Gokhle,M.D., Jacob,G.P., Hundekar,S.L. and Mishra,A.C.
EPI_ISL_17460145	National Institute of Virology	National Institute of Virology	Arankalle,V.A.
EPI_ISL_17460146	National Institute of Virology	National Institute of Virology	Patil,J., More,A., Patil,P., Jadhav,S., Newase,P., Agarwal,M., Amdekar,S., Raut,C.G., Parashar,D. and Cherian,S.S.
EPI_ISL_17460147	National Institute of Virology	National Institute of Virology	Patil,J.A.
EPI_ISL_17460148, EPI_ISL_17460149, EPI_ISL_17460150	National Institute of Virology	National Institute of Virology	Arankalle,V.A.
EPI_ISL_17460151	National Institute of Virology	National Institute of Virology	Arankalle,V.A., Shrivastava,S., Cherian,S., Gunjikar,R.S., Walimbe,A.M., Jadhav,S.M., Sudeep,A.B. and Mishra,A.C.
EPI_ISL_17460152	National Institute of Virology	National Institute of Virology	Arankalle,V.A.
EPI_ISL_17460153	National Institute of Virology	National Institute of Virology	Patil,J., More,A., Patil,P., Jadhav,S., Newase,P., Agarwal,M., Amdekar,S., Raut,C.G., Parashar,D. and Cherian,S.S.
EPI_ISL_17460154	National Institute of Virology	National Institute of Virology	Arankalle,V.A.
EPI_ISL_17460155, EPI_ISL_17460156, EPI_ISL_17460157	National Institute of Virology	National Institute of Virology	Patil,J.A. and More,A.
EPI_ISL_17460158	National Institute of Virology	National Institute of Virology	Patil,J., More,A., Patil,P., Jadhav,S., Newase,P., Agarwal,M., Amdekar,S., Raut,C.G., Parashar,D. and Cherian,S.S.
EPI_ISL_17460159, EPI_ISL_17460160	National Institute of Virology	National Institute of Virology	Arankalle,V.A.
EPI_ISL_17460161, EPI_ISL_17460162	National Institute of Virology	National Institute of Virology	Patil,J., More,A., Patil,P., Jadhav,S., Newase,P., Agarwal,M., Amdekar,S., Raut,C.G., Parashar,D. and Cherian,S.S.
EPI_ISL_17460163, EPI_ISL_17460164, EPI_ISL_17460165, EPI_ISL_17460166, EPI_ISL_17460167, EPI_ISL_17460168, EPI_ISL_17460169, EPI_ISL_17460170	National Institute of Virology	National Institute of Virology	Arankalle,V.A.
EPI_ISL_17460171	National Institute of Virology	National Institute of Virology	Patil,J., More,A., Patil,P., Jadhav,S., Newase,P., Agarwal,M., Amdekar,S., Raut,C.G., Parashar,D. and Cherian,S.S.
EPI_ISL_17460172, EPI_ISL_17460173, EPI_ISL_17460174, EPI_ISL_17460175	National Institute of Virology	National Institute of Virology	Arankalle,V.A.
EPI_ISL_17460176	National Institute of Virology	National Institute of Virology	Arankalle,V.A., Shrivastava,S., Cherian,S., Gunjikar,R.S., Walimbe,A.M., Jadhav,S.M., Sudeep,A.B. and Mishra,A.C.
EPI_ISL_17460177, EPI_ISL_17460178, EPI_ISL_17460179, EPI_ISL_17460180, EPI_ISL_17460181, EPI_ISL_17460182, EPI_ISL_17460183, EPI_ISL_17460184, EPI_ISL_17460185, EPI_ISL_17460186, EPI_ISL_17460187			
see above	National Institute of Virology	National Institute of Virology	Arankalle,V.A.
EPI_ISL_17460188	National Institute of Virology	National Institute of Virology	Arankalle,V.A., Shrivastava,S., Cherian,S., Gunjikar,R.S., Walimbe,A.M., Jadhav,S.M., Sudeep,A.B. and Mishra,A.C.
EPI_ISL_17460189, EPI_ISL_17460190, EPI_ISL_17460191, EPI_ISL_17460192, EPI_ISL_17460193, EPI_ISL_17460194, EPI_ISL_17460195, EPI_ISL_17460196, EPI_ISL_17460197, EPI_ISL_17460198, EPI_ISL_17460199, EPI_ISL_17460200, EPI_ISL_17460201, EPI_ISL_17460202, EPI_ISL_17460203, EPI_ISL_17460204, EPI_ISL_17460205, EPI_ISL_17460206, EPI_ISL_17460207, EPI_ISL_17460208, EPI_ISL_17460209, EPI_ISL_17460210, EPI_ISL_17460211, EPI_ISL_17460212			
see above	National Institute of Virology	National Institute of Virology	Arankalle,V.A.

EPI_ISL_17460213	National Institute of Virology	National Institute of Virology	Munivenkatappa,A., Nyayanit,D.A., Yadav,P.D., R,M., Patil,S., Majumdar,T., Mohandas,S., Sinha,D.P. and Mj,M.
EPI_ISL_17460214, EPI_ISL_17460215, EPI_ISL_17460216, EPI_ISL_17460217, EPI_ISL_17460218, EPI_ISL_17460219, EPI_ISL_17460220, EPI_ISL_17460221	National Institute of Virology	National Institute of Virology	Arankalle,V.A.
EPI_ISL_17460222, EPI_ISL_17460223	National Institute of Virology	National Institute of Virology	Yergolkar,P.N., Tandale,B.V., Arankalle,V.A., Sathe,P.S., Sudeep,A.B., Gandhe,S.S., Gokhle,M.D., Jacob,G.P., Hundekar,S.L. and Mishra,A.C.
EPI_ISL_17460224, EPI_ISL_17460225, EPI_ISL_17460226, EPI_ISL_17460227, EPI_ISL_17460228, EPI_ISL_17460229	National Institute of Virology	National Institute of Virology	Arankalle,V.A.
EPI_ISL_17460230	National Institute of Virology	National Institute of Virology	Gurav,Y.K., Gopalkrishna,V., Shah,P.S., Patil,D.R., Mishra,M., Paingankar,M.S., Singh,A., Sathe,P.S. and Mishra,A.C.
EPI_ISL_17460231	National Institute of Virology	National Institute of Virology	More,A., Patil,J. and Newase,P.K.
EPI_ISL_17460232, EPI_ISL_17460233, EPI_ISL_17460234	National Institute of Virology	National Institute of Virology	Arankalle,V.A.
EPI_ISL_17460235	National Institute of Virology	National Institute of Virology	Yergolkar,P.N., Tandale,B.V., Arankalle,V.A., Sathe,P.S., Sudeep,A.B., Gandhe,S.S., Gokhle,M.D., Jacob,G.P., Hundekar,S.L. and Mishra,A.C.
EPI_ISL_17460236	National Institute of Virology	National Institute of Virology	Gurav,Y.K., Gopalkrishna,V., Shah,P.S., Patil,D.R., Mishra,M., Paingankar,M.S., Singh,A., Sathe,P.S. and Mishra,A.C.
EPI_ISL_17460237, EPI_ISL_17460238, EPI_ISL_17460239, EPI_ISL_17460240, EPI_ISL_17460241, EPI_ISL_17460242, EPI_ISL_17460243, EPI_ISL_17460244, EPI_ISL_17460245, EPI_ISL_17460246, EPI_ISL_17460247, EPI_ISL_17460248, EPI_ISL_17460249, EPI_ISL_17460250, EPI_ISL_17460251			
see above	National Institute of Virology	National Institute of Virology	Arankalle,V.A.
EPI_ISL_17460252	National Institute of Virology	National Institute of Virology	Gurav,Y.K., Gopalkrishna,V., Shah,P.S., Patil,D.R., Mishra,M., Paingankar,M.S., Singh,A., Sathe,P.S. and Mishra,A.C.
EPI_ISL_17460253	National Institute of Virology	National Institute of Virology	Arankalle,V.A.
EPI_ISL_17460254	National Institute of Virology, Diagnostic Virology Group and Hepatitis Group	National Institute of Virology, Diagnostic Virology Group and Hepatitis Group	Deshpande,G., Sapkal,G.N., Tilekar,B., Hundekar,S., Khutwad,K., Gunjikar,R.S. and Vidhate,S.
EPI_ISL_17460255, EPI_ISL_17460256, EPI_ISL_17460257, EPI_ISL_17460258, EPI_ISL_17460259, EPI_ISL_17460260	National University of Singapore	National University of Singapore	Reller,M.E., Akoroda,U., Nagahawatte,A., Devasiri,V., Kodikaarachchi,W., Strouse,J.J., Chua,R., Hou,Y., Chow,A., Sessions,O.M., Ostbye,T., Gubler,D.J., Woods,C.W. and Bodinayake,C.
EPI_ISL_17460261	National institution of Cholera and Enteric Diseases	National institution of Cholera and Enteric Diseases	Chatterjee,R.P., Chakraborty,D.N. and Chatterjee,D.S.
EPI_ISL_17460262	Ningbo International Travel Healthcare Center	Ningbo International Travel Healthcare Center	Zhou,D., Sun,D., Zheng,J., Ni,M., Qiu,J., Wan,Y., Yang,T., Long,Z. and Zai,M.
EPI_ISL_17460263	Ningbo International Travel Healthcare Center	Ningbo International Travel Healthcare Center	Zhou,D., Sun,D., Zheng,J., Qiu,J., Ni,M. and Shi,H.
EPI_ISL_17460264	Oita Universit	Oita Universit	Kamiyama,N., Saechue,B. and Kobayashi,T.
EPI_ISL_17460265, EPI_ISL_17460266	Osaka University	Osaka University	Phadungsombat,J., Imad,H.A., Nakayama,E.E., Leaungwutiwong,P., Ramasoota,P., Nguitragool,W., Matsee,W., Piyaphanee,W. and Shioda,T.
EPI_ISL_17460267	Osaka University	Osaka University	Mulyatno,K.C., Susilowati,H., Yamanaka,A., Soegijanto,S. and Konishi,E.
EPI_ISL_17460268, EPI_ISL_17460269	Osaka University	Osaka University	Phadungsombat,J., Imad,H.A., Nakayama,E.E., Leaungwutiwong,P., Ramasoota,P., Nguitragool,W., Matsee,W., Piyaphanee,W. and Shioda,T.
EPI_ISL_17460270, EPI_ISL_17460271	Osaka University	Osaka University	Mulyatno,K.C., Susilowati,H., Yamanaka,A., Soegijanto,S. and Konishi,E.
EPI_ISL_17460272, EPI_ISL_17460273, EPI_ISL_17460274, EPI_ISL_17460275, EPI_ISL_17460276	Osaka University	Osaka University	Phadungsombat,J., Imad,H.A., Nakayama,E.E., Leaungwutiwong,P., Ramasoota,P., Nguitragool,W., Matsee,W., Piyaphanee,W. and Shioda,T.
EPI_ISL_17460277	Osaka University	Osaka University	Mulyatno,K.C., Susilowati,H., Yamanaka,A., Soegijanto,S. and Konishi,E.
EPI_ISL_17460278	Osaka University	Osaka University	Phadungsombat,J., Imad,H.A., Nakayama,E.E., Leaungwutiwong,P., Ramasoota,P., Nguitragool,W., Matsee,W., Piyaphanee,W. and Shioda,T.
EPI_ISL_17460279, EPI_ISL_17460280, EPI_ISL_17460281, EPI_ISL_17460282, EPI_ISL_17460283	Osaka University	Osaka University	Mulyatno,K.C., Susilowati,H., Yamanaka,A., Soegijanto,S. and Konishi,E.
EPI_ISL_17460284, EPI_ISL_17460285, EPI_ISL_17460286, EPI_ISL_17460287, EPI_ISL_17460288, EPI_ISL_17460289	Osaka University	Osaka University	Phadungsombat,J., Imad,H.A., Nakayama,E.E., Leaungwutiwong,P., Ramasoota,P., Nguitragool,W., Matsee,W., Piyaphanee,W. and Shioda,T.
EPI_ISL_17460290, EPI_ISL_17460291, EPI_ISL_17460292, EPI_ISL_17460293, EPI_ISL_17460294, EPI_ISL_17460295	Osaka University	Osaka University	Mulyatno,K.C., Susilowati,H., Yamanaka,A., Soegijanto,S. and Konishi,E.
EPI_ISL_17460296, EPI_ISL_17460297	Osaka University	Osaka University	Phadungsombat,J., Imad,H.A., Nakayama,E.E., Leaungwutiwong,P., Ramasoota,P., Nguitragool,W., Matsee,W., Piyaphanee,W. and Shioda,T.
EPI_ISL_17460298	Osaka University	Osaka University	Mulyatno,K.C., Susilowati,H., Yamanaka,A., Soegijanto,S. and Konishi,E.
EPI_ISL_17460299, EPI_ISL_17460300, EPI_ISL_17460301, EPI_ISL_17460302, EPI_ISL_17460303, EPI_ISL_17460304, EPI_ISL_17460305	Osaka University	Osaka University	Phadungsombat,J., Imad,H.A., Nakayama,E.E., Leaungwutiwong,P., Ramasoota,P., Nguitragool,W., Matsee,W., Piyaphanee,W. and Shioda,T.
EPI_ISL_17460306, EPI_ISL_17460307	Osaka University	Osaka University	Mulyatno,K.C., Susilowati,H., Yamanaka,A., Soegijanto,S. and Konishi,E.
EPI_ISL_17460308	Osaka University	Osaka University	Phadungsombat,J., Imad,H.A., Nakayama,E.E., Leaungwutiwong,P., Ramasoota,P., Nguitragool,W., Matsee,W., Piyaphanee,W. and Shioda,T.
EPI_ISL_17460309	Osaka University	Osaka University	Mulyatno,K.C., Susilowati,H., Yamanaka,A., Soegijanto,S. and Konishi,E.
EPI_ISL_17460310	Oswaldo Cruz Foundation	Oswaldo Cruz Foundation	De Souza,T.M.A., de Lima,R.C., Solorzano,V.E.F., Damasco,P.V., de Souza,L.J., Sanchez-Arcila,J.C., Guimaraes,G.M.C., Paiva,I.A., da Rocha Queiroz Lima,M., de Bruycker-Nogueira,F., Tome,L.C.T., Coelho,M.R.I., da Silva,S.P., de Oliveira-Pinto,L.M., de Azeredo,E.L. and Dos Santos,F.B.
EPI_ISL_17460311, EPI_ISL_17460312	Oswaldo Cruz Foundation	Oswaldo Cruz Foundation	Lessa-Aquino,C., Trinta,K.S., Pestana,C.P., Ribeiro,M.O., Sucupira,M.V., Boia,M.N., Baptista,P.A., Cunha,R.V. and Medeiros,M.A.
EPI_ISL_17460313	Oswaldo Cruz Foundation	Oswaldo Cruz Foundation	De Souza,T.M.A., de Lima,R.C., Solorzano,V.E.F., Damasco,P.V., de Souza,L.J., Sanchez-Arcila,J.C., Guimaraes,G.M.C., Paiva,I.A., da Rocha Queiroz Lima,M., de Bruycker-Nogueira,F., Tome,L.C.T., Coelho,M.R.I., da Silva,S.P., de Oliveira-Pinto,L.M., de Azeredo,E.L. and Dos Santos,F.B.
EPI_ISL_17460314	Oswaldo Cruz Foundation	Oswaldo Cruz Foundation	Conteville,L.C., Zanella,L., Marin,M.A., Filippis,A.M., Nogueira,R.M., Vicente,A.C. and Mendonca,M.C.
EPI_ISL_17460315	Oswaldo Cruz Foundation	Oswaldo Cruz Foundation	De Souza,T.M.A., de Lima,R.C., Solorzano,V.E.F., Damasco,P.V., de Souza,L.J., Sanchez-Arcila,J.C., Guimaraes,G.M.C., Paiva,I.A., da Rocha Queiroz Lima,M., de Bruycker-Nogueira,F., Tome,L.C.T., Coelho,M.R.I., da Silva,S.P., de Oliveira-Pinto,L.M., de Azeredo,E.L. and Dos Santos,F.B.
EPI_ISL_17460316	Oswaldo Cruz Foundation	Oswaldo Cruz Foundation	Thompson,F., Tschoeke,D., Souza,T., Rangel,Y., Delatorre,E., Leomil,L., Morel,C., Lipkin,I. and Mishra,N.
EPI_ISL_17460317	Oswaldo Cruz Foundation	Oswaldo Cruz Foundation	Conteville,L.C., Zanella,L., Marin,M.A., Filippis,A.M., Nogueira,R.M., Vicente,A.C. and Mendonca,M.C.
EPI_ISL_17460318	Oswaldo Cruz Foundation	Oswaldo Cruz Foundation	Lessa-Aquino,C., Trinta,K.S., Pestana,C.P., Ribeiro,M.O., Sucupira,M.V., Boia,M.N., Baptista,P.A., Cunha,R.V. and Medeiros,M.A.
EPI_ISL_17460319	Oswaldo Cruz Foundation	Oswaldo Cruz Foundation	De Souza,T.M.A., de Lima,R.C., Solorzano,V.E.F., Damasco,P.V., de Souza,L.J., Sanchez-Arcila,J.C., Guimaraes,G.M.C., Paiva,I.A., da Rocha Queiroz Lima,M., de Bruycker-Nogueira,F., Tome,L.C.T., Coelho,M.R.I., da Silva,S.P., de Oliveira-Pinto,L.M., de Azeredo,E.L. and Dos Santos,F.B.
EPI_ISL_17460320	Oswaldo Cruz Foundation	Oswaldo Cruz Foundation	Lessa-Aquino,C., Trinta,K.S., Pestana,C.P., Ribeiro,M.O., Sucupira,M.V., Boia,M.N., Baptista,P.A., Cunha,R.V. and Medeiros,M.A.
EPI_ISL_17460321	Oswaldo Cruz Foundation	Oswaldo Cruz Foundation	Pena,L.J., Guarines,K.M., Silva,A., Junior,A.F.P., Cordeiro,M.T., Brito,C.A.A., Silva,V.G., Rezende,A.M., Machado,L.C., Rezende,T.M.T. and Wallau,G.L.
EPI_ISL_17460322, EPI_ISL_17460323	Oswaldo Cruz Foundation	Oswaldo Cruz Foundation	Lessa-Aquino,C., Trinta,K.S., Pestana,C.P., Ribeiro,M.O., Sucupira,M.V., Boia,M.N., Baptista,P.A., Cunha,R.V. and Medeiros,M.A.
EPI_ISL_17460324	Oswaldo Cruz Foundation	Oswaldo Cruz Foundation	De Souza,T.M.A., de Lima,R.C., Solorzano,V.E.F., Damasco,P.V., de Souza,L.J., Sanchez-Arcila,J.C., Guimaraes,G.M.C., Paiva,I.A., da Rocha Queiroz Lima,M., de Bruycker-Nogueira,F., Tome,L.C.T., Coelho,M.R.I., da Silva,S.P., de Oliveira-Pinto,L.M., de Azeredo,E.L. and Dos Santos,F.B.
EPI_ISL_17460325, EPI_ISL_17460326, EPI_ISL_17460327	Oswaldo Cruz Foundation	Oswaldo Cruz Foundation	de Souza,T.M.A., de Lima,R.C., Solorzano,V.E.F., Damasco,P.V., de Souza,L.J., Sanchez-Arcila,J.C., Guimaraes,G.M.C., Paiva,I.A., da Rocha Queiroz Lima,M., de Bruycker-Nogueira,F., Tome,L.C.T., Coelho,M.R.I., da Silva,S.P., de Oliveira-Pinto,L.M., de Azeredo,E.L. and Dos Santos,F.B.
EPI_ISL_17460328, EPI_ISL_17460329, EPI_ISL_17460330, EPI_ISL_17460331, EPI_ISL_17460332, EPI_ISL_17460333	Oswaldo Cruz Foundation	Oswaldo Cruz Foundation	Thompson,F., Tschoeke,D., Souza,T., Rangel,Y., Delatorre,E., Leomil,L., Morel,C., Lipkin,I. and Mishra,N.
EPI_ISL_17460334	Oswaldo Cruz Foundation	Oswaldo Cruz Foundation	Lessa-Aquino,C., Trinta,K.S., Pestana,C.P., Ribeiro,M.O., Sucupira,M.V., Boia,M.N., Baptista,P.A., Cunha,R.V. and Medeiros,M.A.
EPI_ISL_17460335	Oswaldo Cruz Foundation	Oswaldo Cruz Foundation	Conteville,L.C., Zanella,L., Marin,M.A., Filippis,A.M., Nogueira,R.M., Vicente,A.C. and Mendonca,M.C.
EPI_ISL_17460336	Oswaldo Cruz Foundation	Oswaldo Cruz Foundation	Lessa-Aquino,C., Trinta,K.S., Pestana,C.P., Ribeiro,M.O., Sucupira,M.V., Boia,M.N., Baptista,P.A., Cunha,R.V. and Medeiros,M.A.
EPI_ISL_17460337, EPI_ISL_17460338, EPI_ISL_17460339, EPI_ISL_17460340, EPI_ISL_17460341, EPI_ISL_17460342, EPI_ISL_17460343, EPI_ISL_17460344, EPI_ISL_17460345	Oswaldo Cruz Foundation	Oswaldo Cruz Foundation	Thompson,F., Tschoeke,D., Souza,T., Rangel,Y., Delatorre,E., Leomil,L., Morel,C., Lipkin,I. and Mishra,N.
EPI_ISL_17460346	Oswaldo Cruz Foundation	Oswaldo Cruz Foundation	de Souza,T.M.A., de Lima,R.C., Solorzano,V.E.F., Damasco,P.V., de Souza,L.J., Sanchez-Arcila,J.C., Guimaraes,G.M.C., Paiva,I.A., da Rocha Queiroz Lima,M., de Bruycker-Nogueira,F., Tome,L.C.T., Coelho,M.R.I., da Silva,S.P., de Oliveira-Pinto,L.M., de Azeredo,E.L. and Dos Santos,F.B.
EPI_ISL_17460347	Oswaldo Cruz Foundation	Oswaldo Cruz Foundation	Lessa-Aquino,C., Trinta,K.S., Pestana,C.P., Ribeiro,M.O., Sucupira,M.V., Boia,M.N., Baptista,P.A., Cunha,R.V. and Medeiros,M.A.
EPI_ISL_17460348	Oswaldo Cruz Foundation	Oswaldo Cruz Foundation	de Souza,T.M.A., de Lima,R.C., Solorzano,V.E.F., Damasco,P.V., de Souza,L.J., Sanchez-Arcila,J.C., Guimaraes,G.M.C., Paiva,I.A., da Rocha Queiroz Lima,M., de Bruycker-Nogueira,F., Tome,L.C.T., Coelho,M.R.I., da Silva,S.P., de Oliveira-Pinto,L.M., de Azeredo,E.L. and Dos Santos,F.B.
EPI_ISL_17460349	Oswaldo Cruz Foundation	Oswaldo Cruz Foundation	Lessa-Aquino,C., Trinta,K.S., Pestana,C.P., Ribeiro,M.O., Sucupira,M.V., Boia,M.N., Baptista,P.A., Cunha,R.V. and Medeiros,M.A.
EPI_ISL_17460350, EPI_ISL_17460351, EPI_ISL_17460352, EPI_ISL_17460353, EPI_ISL_17460354	Oswaldo Cruz Foundation	Oswaldo Cruz Foundation	Pena,L.J., Guarines,K.M., Silva,A., Junior,A.F.P., Cordeiro,M.T., Brito,C.A.A., Silva,V.G., Rezende,A.M., Machado,L.C., Rezende,T.M.T. and Wallau,G.L.

EPI_ISL_17460355	Oswaldo Cruz Foundation	Oswaldo Cruz Foundation	Conteville,L.C., Zanella,L., Marin,M.A., Filippis,A.M., Nogueira,R.M., Vicente,A.C. and Mendonca,M.C.
EPI_ISL_17460356	Oswaldo Cruz Foundation	Oswaldo Cruz Foundation	Pena,L.J., Guarines,K.M., Silva,A., Junior,A.F.P., Cordeiro,M.T., Brito,C.A.A., Silva,V.G., Rezende,A.M., Machado,L.C., Rezende,T.M.T. and Wallau,G.L.
EPI_ISL_17460357, EPI_ISL_17460358, EPI_ISL_17460359, EPI_ISL_17460360, EPI_ISL_17460361	Oswaldo Cruz Foundation - Goncalo Moniz Institute, Laboratory of Experimental Pathology	Oswaldo Cruz Foundation - Goncalo Moniz Institute, Laboratory of Experimental Pathology	Goes de Jesus,J., da Luz Wallau,G., Lima Maia,M., Xavier,J., Oliveira Lima,M.A., Fonseca,V., Salgado de Abreu,A., Fraga de Oliveira Tosta,S., Ramos do Amaral,H., Andrade Barbosa Lima,I., Viana Silva,P., Carlos Dos Santos,D., Sousa de Oliveira,A., Campos de Souza,S., Barreto Falcao,M., Cerqueira,E., Ceschini Machado,L., Sobral,M.C., Teodoro Rezende,T.M., Ribeiro Pereira,M., Mota Pereira,F., Pereira Gusmao Maia,Z., Freitas de Oliveira Franca,R., Luiz de Abreu,A., Campelo de Albuquerque E Melo,C.F., Rodrigues Faria,N., Venancio da Cunha,R., Giovanetti,M. and Alcantara,L.C.J.
EPI_ISL_17460362	Oswaldo Cruz Institute	Oswaldo Cruz Institute	Damasceno dos Santos Rodrigues,C., Torres,M.C., Lima de Mendonca,M.C., Ribeiro Nogueira,R.M. and Bispo de Filippis,A.M.
EPI_ISL_17460363	Oswaldo Cruz Institute	Oswaldo Cruz Institute	Fabri,A.A., Rodrigues,C.Dd.S., Santos,C.Cd., Chalhoub,F.L.L., Sampaio,S.A., Faria,N.Rd.C., Torres,M.C., Fonseca,V., Brasil,P., Calvet,G., Alcantara,L.C.J., Filippis,A.M.Bd., Giovanetti,M. and de Bruycker-Nogueira,F.
EPI_ISL_17460364	Oswaldo Cruz Institute	Oswaldo Cruz Institute	Damasceno dos Santos Rodrigues,C., Torres,M.C., Lima de Mendonca,M.C., Ribeiro Nogueira,R.M. and Bispo de Filippis,A.M.
EPI_ISL_17460365	Oswaldo Cruz Institute	Oswaldo Cruz Institute	Damasceno dos Santos Rodrigues,C., Torres,M.C., Bispo de Filippis,A.M. and Lima de Mendonca,M.C.
EPI_ISL_17460366, EPI_ISL_17460367, EPI_ISL_17460368	Oswaldo Cruz Institute	Oswaldo Cruz Institute	Damasceno dos Santos Rodrigues,C., Torres,M.C., Lima de Mendonca,M.C., Ribeiro Nogueira,R.M. and Bispo de Filippis,A.M.
EPI_ISL_17460369	Oswaldo Cruz Institute	Oswaldo Cruz Institute	Damasceno dos Santos Rodrigues,C., Torres,M.C., Bispo de Filippis,A.M. and Lima de Mendonca,M.C.
EPI_ISL_17460370	Oswaldo Cruz Institute	Oswaldo Cruz Institute	de Souza,T.M., Ribeiro,E.D., Correa,V.C., Damasco,P.V., Santos,C.C., de Bruycker-Nogueira,F., Chouin-Carneiro,T., Faria,N.R., Nunes,P.C., Heringer,M., Lima,M.D., Badolato-Correa,J., Cipitelli,M.D., Azeredo,E.L., Nogueira,R.M. and Dos Santos,F.B.
EPI_ISL_17460371	Oswaldo Cruz Institute	Oswaldo Cruz Institute	Damasceno dos Santos Rodrigues,C., Torres,M.C., Bispo de Filippis,A.M. and Lima de Mendonca,M.C.
EPI_ISL_17460372	Oswaldo Cruz Institute	Oswaldo Cruz Institute	Fabri,A.A., Rodrigues,C.Dd.S., Santos,C.Cd., Chalhoub,F.L.L., Sampaio,S.A., Faria,N.Rd.C., Torres,M.C., Fonseca,V., Brasil,P., Calvet,G., Alcantara,L.C.J., Filippis,A.M.Bd., Giovanetti,M. and de Bruycker-Nogueira,F.
EPI_ISL_17460373, EPI_ISL_17460374	Oswaldo Cruz Institute	Oswaldo Cruz Institute	Damasceno dos Santos Rodrigues,C., Torres,M.C., Bispo de Filippis,A.M. and Lima de Mendonca,M.C.
EPI_ISL_17460375, EPI_ISL_17460376	Oswaldo Cruz Institute	Oswaldo Cruz Institute	Fabri,A.A., Rodrigues,C.Dd.S., Santos,C.Cd., Chalhoub,F.L.L., Sampaio,S.A., Faria,N.Rd.C., Torres,M.C., Fonseca,V., Brasil,P., Calvet,G., Alcantara,L.C.J., Filippis,A.M.Bd., Giovanetti,M. and de Bruycker-Nogueira,F.
EPI_ISL_17460377	Oswaldo Cruz Institute	Oswaldo Cruz Institute	Damasceno dos Santos Rodrigues,C., Torres,M.C., Bispo de Filippis,A.M. and Lima de Mendonca,M.C.
EPI_ISL_17460378, EPI_ISL_17460379, EPI_ISL_17460380	Oswaldo Cruz Institute	Oswaldo Cruz Institute	de Souza,T.M., Ribeiro,E.D., Correa,V.C., Damasco,P.V., Santos,C.C., de Bruycker-Nogueira,F., Chouin-Carneiro,T., Faria,N.R., Nunes,P.C., Heringer,M., Lima,M.D., Badolato-Correa,J., Cipitelli,M.D., Azeredo,E.L., Nogueira,R.M. and Dos Santos,F.B.
EPI_ISL_17460381	Oswaldo Cruz Institute	Oswaldo Cruz Institute	Damasceno dos Santos Rodrigues,C., Torres,M.C., Lima de Mendonca,M.C., Ribeiro Nogueira,R.M. and Bispo de Filippis,A.M.
EPI_ISL_17460382, EPI_ISL_17460383	Oswaldo Cruz Institute	Oswaldo Cruz Institute	Fabri,A.A., Rodrigues,C.Dd.S., Santos,C.Cd., Chalhoub,F.L.L., Sampaio,S.A., Faria,N.Rd.C., Torres,M.C., Fonseca,V., Brasil,P., Calvet,G., Alcantara,L.C.J., Filippis,A.M.Bd., Giovanetti,M. and de Bruycker-Nogueira,F.
EPI_ISL_17460384	Oswaldo Cruz Institute	Oswaldo Cruz Institute	de Souza,T.M., Ribeiro,E.D., Correa,V.C., Damasco,P.V., Santos,C.C., de Bruycker-Nogueira,F., Chouin-Carneiro,T., Faria,N.R., Nunes,P.C., Heringer,M., Lima,M.D., Badolato-Correa,J., Cipitelli,M.D., Azeredo,E.L., Nogueira,R.M. and Dos Santos,F.B.
EPI_ISL_17460385	Oswaldo Cruz Institute	Oswaldo Cruz Institute	Fabri,A.A., Rodrigues,C.Dd.S., Santos,C.Cd., Chalhoub,F.L.L., Sampaio,S.A., Faria,N.Rd.C., Torres,M.C., Fonseca,V., Brasil,P., Calvet,G., Alcantara,L.C.J., Filippis,A.M.Bd., Giovanetti,M. and de Bruycker-Nogueira,F.
EPI_ISL_17460386	Oswaldo Cruz Institute	Oswaldo Cruz Institute	de Souza,T.M., Ribeiro,E.D., Correa,V.C., Damasco,P.V., Santos,C.C., de Bruycker-Nogueira,F., Chouin-Carneiro,T., Faria,N.R., Nunes,P.C., Heringer,M., Lima,M.D., Badolato-Correa,J., Cipitelli,M.D., Azeredo,E.L., Nogueira,R.M. and Dos Santos,F.B.
EPI_ISL_17460387, EPI_ISL_17460388, EPI_ISL_17460389	Oswaldo Cruz Institute	Oswaldo Cruz Institute	Fabri,A.A., Rodrigues,C.Dd.S., Santos,C.Cd., Chalhoub,F.L.L., Sampaio,S.A., Faria,N.Rd.C., Torres,M.C., Fonseca,V., Brasil,P., Calvet,G., Alcantara,L.C.J., Filippis,A.M.Bd., Giovanetti,M. and de Bruycker-Nogueira,F.
EPI_ISL_17460390	Oswaldo Cruz Institute	Oswaldo Cruz Institute	Damasceno dos Santos Rodrigues,C., Torres,M.C., Bispo de Filippis,A.M. and Lima de Mendonca,M.C.
EPI_ISL_17460391	Oswaldo Cruz Institute	Oswaldo Cruz Institute	Fabri,A.A., Rodrigues,C.Dd.S., Santos,C.Cd., Chalhoub,F.L.L., Sampaio,S.A., Faria,N.Rd.C., Torres,M.C., Fonseca,V., Brasil,P., Calvet,G., Alcantara,L.C.J., Filippis,A.M.Bd., Giovanetti,M. and de Bruycker-Nogueira,F.
EPI_ISL_17460392, EPI_ISL_17460393, EPI_ISL_17460394, EPI_ISL_17460395, EPI_ISL_17460396, EPI_ISL_17460397, EPI_ISL_17460398	Oswaldo Cruz Institute	Oswaldo Cruz Institute	de Souza,T.M., Ribeiro,E.D., Correa,V.C., Damasco,P.V., Santos,C.C., de Bruycker-Nogueira,F., Chouin-Carneiro,T., Faria,N.R., Nunes,P.C., Heringer,M., Lima,M.D., Badolato-Correa,J., Cipitelli,M.D., Azeredo,E.L., Nogueira,R.M. and Dos Santos,F.B.
EPI_ISL_17460399, EPI_ISL_17460400	Oswaldo Cruz Institute	Oswaldo Cruz Institute	Damasceno dos Santos Rodrigues,C., Torres,M.C., Bispo de Filippis,A.M. and Lima de Mendonca,M.C.
EPI_ISL_17460401	Oswaldo Cruz Institute	Oswaldo Cruz Institute	de Souza,T.M., Ribeiro,E.D., Correa,V.C., Damasco,P.V., Santos,C.C., de Bruycker-Nogueira,F., Chouin-Carneiro,T., Faria,N.R., Nunes,P.C., Heringer,M., Lima,M.D., Badolato-Correa,J., Cipitelli,M.D., Azeredo,E.L., Nogueira,R.M. and Dos Santos,F.B.
EPI_ISL_17460402, EPI_ISL_17460403, EPI_ISL_17460404	Oswaldo Cruz Institute	Oswaldo Cruz Institute	Fabri,A.A., Rodrigues,C.Dd.S., Santos,C.Cd., Chalhoub,F.L.L., Sampaio,S.A., Faria,N.Rd.C., Torres,M.C., Fonseca,V., Brasil,P., Calvet,G., Alcantara,L.C.J., Filippis,A.M.Bd., Giovanetti,M. and de Bruycker-Nogueira,F.
EPI_ISL_17460405	Oswaldo Cruz Institute	Oswaldo Cruz Institute	Damasceno dos Santos Rodrigues,C., Torres,M.C., Bispo de Filippis,A.M. and Lima de Mendonca,M.C.
EPI_ISL_17460406, EPI_ISL_17460407, EPI_ISL_17460408	Oswaldo Cruz Institute	Oswaldo Cruz Institute	de Souza,T.M., Ribeiro,E.D., Correa,V.C., Damasco,P.V., Santos,C.C., de Bruycker-Nogueira,F., Chouin-Carneiro,T., Faria,N.R., Nunes,P.C., Heringer,M., Lima,M.D., Badolato-Correa,J., Cipitelli,M.D., Azeredo,E.L., Nogueira,R.M. and Dos Santos,F.B.
EPI_ISL_17460409	Oswaldo Cruz Institute	Oswaldo Cruz Institute	Damasceno dos Santos Rodrigues,C., Torres,M.C., Bispo de Filippis,A.M. and Lima de Mendonca,M.C.
EPI_ISL_17460410, EPI_ISL_17460411, EPI_ISL_17460412, EPI_ISL_17460413, EPI_ISL_17460414, EPI_ISL_17460415, EPI_ISL_17460416, EPI_ISL_17460417, EPI_ISL_17460418	Oswaldo Cruz Institute	Oswaldo Cruz Institute	Fabri,A.A., Rodrigues,C.Dd.S., Santos,C.Cd., Chalhoub,F.L.L., Sampaio,S.A., Faria,N.Rd.C., Torres,M.C., Fonseca,V., Brasil,P., Calvet,G., Alcantara,L.C.J., Filippis,A.M.Bd., Giovanetti,M. and de Bruycker-Nogueira,F.
EPI_ISL_17460419	Oxford University Clinical Research Unit	Oxford University Clinical Research Unit	Quyen,N.T.H., Kien,D.T.H., Rabaa,M., Tuan,N.M., Vi,T.T., Van Tan,L., Hung,N.T., Tuan,H.M., Van Tram,T., Le Da Ha,N., Quang,H.K., Doanh,N.Q., Van Vinh Chau,N., Wills,B. and Simmons,C.P.
EPI_ISL_17460420	Oxford University Clinical Research Unit	Oxford University Clinical Research Unit	Tan,L.V., Nguyet,L.A., Duong,K.T.H., Vi,T.T. and Simmons,C.P.
EPI_ISL_17460421, EPI_ISL_17460422	Oxford University Clinical Research Unit	Oxford University Clinical Research Unit	Quyen,N.T.H., Kien,D.T.H., Rabaa,M., Tuan,N.M., Vi,T.T., Van Tan,L., Hung,N.T., Tuan,H.M., Van Tram,T., Le Da Ha,N., Quang,H.K., Doanh,N.Q., Van Vinh Chau,N., Wills,B. and Simmons,C.P.
EPI_ISL_17460423, EPI_ISL_17460424	Oxford University Clinical Research Unit	Oxford University Clinical Research Unit	Quyen,N.T.H., Rabaa,M., Tuan,N.M., Kien,D.H., Wills,B. and Simmons,C.P.
EPI_ISL_17460425	Oxford University Clinical Research Unit	Oxford University Clinical Research Unit	Quyen,N.T.H., Kien,D.T.H., Rabaa,M., Tuan,N.M., Vi,T.T., Van Tan,L., Hung,N.T., Tuan,H.M., Van Tram,T., Le Da Ha,N., Quang,H.K., Doanh,N.Q., Van Vinh Chau,N., Wills,B. and Simmons,C.P.
EPI_ISL_17460426, EPI_ISL_17460427, EPI_ISL_17460428, EPI_ISL_17460429, EPI_ISL_17460430, EPI_ISL_17460431, EPI_ISL_17460432, EPI_ISL_17460433, EPI_ISL_17460434, EPI_ISL_17460435, EPI_ISL_17460436, EPI_ISL_17460437, EPI_ISL_17460438, EPI_ISL_17460439, EPI_ISL_17460440, EPI_ISL_17460441, EPI_ISL_17460442, EPI_ISL_17460443, EPI_ISL_17460444, EPI_ISL_17460445			
see above	Padjadjaran University	Padjadjaran University	Kosasih,H., de Mast,Q., Widjaja,S., Sudjana,P., Antonjaya,U., Ma'roef,C., Riswari,S.F., Porter,K.R., Burgess,T.H., Alisjahbana,B., van der Ven,A. and Williams,M.
EPI_ISL_17460446, EPI_ISL_17460447, EPI_ISL_17460448	Papua New Guinea Institute of Medical Research	Papua New Guinea Institute of Medical Research	Horwood,P.F., Reimer,L.J., Dagina,R., Susapu,M., Bande,G., Katusele,M., Koimbu,G., Jimmy,S., Ropa,B., Siba,P.M. and Pavlin,B.I.
EPI_ISL_17460449	Pasteur Institute of Iran	Pasteur Institute of Iran	Pouriayevali,M.H., Jalai,T., Mohammadi,T., Fereydouni,Z., TavakoliRad,M., Azadmanjiri,S., Khakifirouz,S., Hosseini,M., Ghalejoogh,M., Azizizadeh,S., Baniasadi,V., Fazlalipour,M. and Salehi-Vaziri,M.
EPI_ISL_17460450	Pasteur Institute of Iran	Pasteur Institute of Iran	Pouriayevali,M.H., Jalali,T., Mohammadi,T., Fereydouni,Z., TavakoliRad,M., Azadmanjiri,S., Khakifirouz,S., Hosseini,M., Ghalejoogh,M., Baniasadi,V., Fazlalipour,M. and Salehi-Vaziri,M.
EPI_ISL_17460451, EPI_ISL_17460452, EPI_ISL_17460453	Pasteur Institute of Iran	Pasteur Institute of Iran	Pouriayevali,M.H., Jalai,T., Mohammadi,T., Fereydouni,Z., TavakoliRad,M., Azadmanjiri,S., Khakifirouz,S., Hosseini,M., Ghalejoogh,M., Azizizadeh,S., Baniasadi,V., Fazlalipour,M. and Salehi-Vaziri,M.
EPI_ISL_17460454, EPI_ISL_17460455	Postgraduate Institute of Medical Education and Research	Postgraduate Institute of Medical Education and Research	Mishra,B., Sharma,M., Pujhari,S.K., Ratho,R.K., Gopal,D.S., Kumar,C.N., Sarangi,G., Chayani,N. and Varma,S.C.
EPI_ISL_17460456	Prince of Songkla University	Prince of Songkla University	Na nakorn,N., Vongpunsawad,S., Poovorawan,Y. and Pengsakul,T.
EPI_ISL_17460457	Prince of Songkla University	Prince of Songkla University	Na Nakorn,N., Vongpunsawad,S., Poovorawan,Y. and Pengsakul,T.
EPI_ISL_17460458	Prince of Songkla University	Prince of Songkla University	Na nakorn,N., Vongpunsawad,S., Poovorawan,Y. and Pengsakul,T.
EPI_ISL_17460459, EPI_ISL_17460460	Prince of Songkla University	Prince of Songkla University	Na Nakorn,N., Vongpunsawad,S., Poovorawan,Y. and Pengsakul,T.
EPI_ISL_17460461, EPI_ISL_17460462, EPI_ISL_17460463, EPI_ISL_17460464, EPI_ISL_17460465, EPI_ISL_17460466, EPI_ISL_17460467, EPI_ISL_17460468	Prince of Songkla University	Prince of Songkla University	Na nakorn,N., Vongpunsawad,S., Poovorawan,Y. and Pengsakul,T.
EPI_ISL_17460469	Prince of Songkla University	Prince of Songkla University	Na Nakorn,N., Vongpunsawad,S., Poovorawan,Y. and Pengsakul,T.
EPI_ISL_17460470, EPI_ISL_17460471, EPI_ISL_17460472, EPI_ISL_17460473, EPI_ISL_17460474	Prince of Songkla University	Prince of Songkla University	Na nakorn,N., Vongpunsawad,S., Poovorawan,Y. and Pengsakul,T.
EPI_ISL_17460475	Prince of Songkla University	Prince of Songkla University	Na Nakorn,N., Vongpunsawad,S., Poovorawan,Y. and Pengsakul,T.
EPI_ISL_17460476, EPI_ISL_17460477, EPI_ISL_17460478, EPI_ISL_17460479, EPI_ISL_17460480, EPI_ISL_17460481, EPI_ISL_17460482, EPI_ISL_17460483, EPI_ISL_17460484	Public Health England	Public Health England	Bower,H., El Karsany,M., Adam,A.A.A.H., Idriss,M.I., Alzain,M.A., Alfakiyousif,M.E.A., Mohamed,R., Mahmoud,I., Albadri,O., Mahmoud,S.A.A., Abdalla,O.I., Eldigail,M., Elagib,N., Arnold,U., Gutierrez,B., Pybus,O.G., Carter,D.P., Pullan,S.T., Jacob,S.T., Abdallah,T.M., Gannon,B. and Fletcher,T.E.
EPI_ISL_17460485	Public Health England	Public Health England	Kafetzopoulou,L.E., Efthymiadis,K., Lewandowski,K., Crook,A., Carter,D., Osborne,J., Aarons,E., Hewson,R., Hiscox,J.A., Carroll,M.W., Vipond,R. and Pullan,S.T.
EPI_ISL_17460486, EPI_ISL_17460487, EPI_ISL_17460488, EPI_ISL_17460489	Public Health England	Public Health England	Bower,H., El Karsany,M., Adam,A.A.A.H., Idriss,M.I., Alzain,M.A., Alfakiyousif,M.E.A., Mohamed,R., Mahmoud,I., Albadri,O., Mahmoud,S.A.A., Abdalla,O.I., Eldigail,M., Elagib,N., Arnold,U., Gutierrez,B., Pybus,O.G., Carter,D.P., Pullan,S.T., Jacob,S.T., Abdallah,T.M., Gannon,B. and Fletcher,T.E.
EPI_ISL_17460490, EPI_ISL_17460491	Public Health England	Public Health England	Kafetzopoulou,L.E., Efthymiadis,K., Lewandowski,K., Crook,A., Carter,D., Osborne,J., Aarons,E., Hewson,R., Hiscox,J.A., Carroll,M.W., Vipond,R. and Pullan,S.T.
EPI_ISL_17460492	Public Health England	Public Health England	Bower,H., El Karsany,M., Adam,A.A.A.H., Idriss,M.I., Alzain,M.A., Alfakiyousif,M.E.A., Mohamed,R., Mahmoud,I., Albadri,O., Mahmoud,S.A.A., Abdalla,O.I., Eldigail,M., Elagib,N., Arnold,U., Gutierrez,B., Pybus,O.G., Carter,D.P., Pullan,S.T., Jacob,S.T., Abdallah,T.M., Gannon,B. and Fletcher,T.E.
EPI_ISL_17460493	Public Health England	Public Health England	Kafetzopoulou,L.E., Efthymiadis,K., Lewandowski,K., Crook,A., Carter,D., Osborne,J., Aarons,E., Hewson,R., Hiscox,J.A., Carroll,M.W., Vipond,R. and Pullan,S.T.
EPI_ISL_17460494, EPI_ISL_17460495, EPI_ISL_17460496, EPI_ISL_17460497, EPI_ISL_17460498, EPI_ISL_17460499, EPI_ISL_17460500, EPI_ISL_17460501	Public Health England	Public Health England	Bower,H., El Karsany,M., Adam,A.A.A.H., Idriss,M.I., Alzain,M.A., Alfakiyousif,M.E.A., Mohamed,R., Mahmoud,I., Albadri,O., Mahmoud,S.A.A., Abdalla,O.I., Eldigail,M., Elagib,N., Arnold,U., Gutierrez,B., Pybus,O.G., Carter,D.P., Pullan,S.T., Jacob,S.T., Abdallah,T.M., Gannon,B. and Fletcher,T.E.
EPI_ISL_17460502	Public Health England	Public Health England	Kafetzopoulou,L.E., Efthymiadis,K., Lewandowski,K., Crook,A., Carter,D., Osborne,J., Aarons,E., Hewson,R., Hiscox,J.A., Carroll,M.W., Vipond,R. and Pullan,S.T.
EPI_ISL_17460503, EPI_ISL_17460504, EPI_ISL_17460505, EPI_ISL_17460506, EPI_ISL_17460507	Public Health England	Public Health England	Bower,H., El Karsany,M., Adam,A.A.A.H., Idriss,M.I., Alzain,M.A., Alfakiyousif,M.E.A., Mohamed,R., Mahmoud,I., Albadri,O., Mahmoud,S.A.A., Abdalla,O.I., Eldigail,M., Elagib,N., Arnold,U., Gutierrez,B., Pybus,O.G., Carter,D.P., Pullan,S.T., Jacob,S.T., Abdallah,T.M., Gannon,B. and Fletcher,T.E.

EPI_ISL_17460508, EPI_ISL_17460509, EPI_ISL_17460510, EPI_ISL_17460511, EPI_ISL_17460512	Public Health England	Public Health England	Kafetzopoulou,L.E., Efthymiadis,K., Lewandowski,K., Crook,A., Carter,D., Osborne,J., Aarons,E., Hewson,R., Hiscox,J.A., Carroll,M.W., Vipond,R. and Pullan,S.T.
EPI_ISL_17460513	Public Health England	Public Health England	Bower,H., El Karsany,M., Adam,A.A.A.H., Idriss,M.I., Alzain,M.A., Alfakiyousif,M.E.A., Mohamed,R., Mahmoud,I., Albadri,O., Mahmoud,S.A.A., Abdalla,O.I., Eldigail,M., Elagib,N., Arnold,U., Gutierrez,B., Pybus,O.G., Carter,D.P., Pullan,S.T., Jacob,S.T., Abdallah,T.M., Gannon,B. and Fletcher,T.E.
EPI_ISL_17460514, EPI_ISL_17460515	Public Health England	Public Health England	Kafetzopoulou,L.E., Efthymiadis,K., Lewandowski,K., Crook,A., Carter,D., Osborne,J., Aarons,E., Hewson,R., Hiscox,J.A., Carroll,M.W., Vipond,R. and Pullan,S.T.
EPI_ISL_17460516, EPI_ISL_17460517, EPI_ISL_17460518, EPI_ISL_17460519, EPI_ISL_17460520, EPI_ISL_17460521, EPI_ISL_17460522	Public Health England	Public Health England	Bower,H., El Karsany,M., Adam,A.A.A.H., Idriss,M.I., Alzain,M.A., Alfakiyousif,M.E.A., Mohamed,R., Mahmoud,I., Albadri,O., Mahmoud,S.A.A., Abdalla,O.I., Eldigail,M., Elagib,N., Arnold,U., Gutierrez,B., Pybus,O.G., Carter,D.P., Pullan,S.T., Jacob,S.T., Abdallah,T.M., Gannon,B. and Fletcher,T.E.
EPI_ISL_17460523	Public Health England	Public Health England	Kafetzopoulou,L.E., Efthymiadis,K., Lewandowski,K., Crook,A., Carter,D., Osborne,J., Aarons,E., Hewson,R., Hiscox,J.A., Carroll,M.W., Vipond,R. and Pullan,S.T.
EPI_ISL_17460524	Public Health England	Public Health England	Bower,H., El Karsany,M., Adam,A.A.A.H., Idriss,M.I., Alzain,M.A., Alfakiyousif,M.E.A., Mohamed,R., Mahmoud,I., Albadri,O., Mahmoud,S.A.A., Abdalla,O.I., Eldigail,M., Elagib,N., Arnold,U., Gutierrez,B., Pybus,O.G., Carter,D.P., Pullan,S.T., Jacob,S.T., Abdallah,T.M., Gannon,B. and Fletcher,T.E.
EPI_ISL_17460525	Public Health England	Public Health England	Kafetzopoulou,L.E., Efthymiadis,K., Lewandowski,K., Crook,A., Carter,D., Osborne,J., Aarons,E., Hewson,R., Hiscox,J.A., Carroll,M.W., Vipond,R. and Pullan,S.T.
EPI_ISL_17460526, EPI_ISL_17460527, EPI_ISL_17460528, EPI_ISL_17460529	Public Health England	Public Health England	Bower,H., El Karsany,M., Adam,A.A.A.H., Idriss,M.I., Alzain,M.A., Alfakiyousif,M.E.A., Mohamed,R., Mahmoud,I., Albadri,O., Mahmoud,S.A.A., Abdalla,O.I., Eldigail,M., Elagib,N., Arnold,U., Gutierrez,B., Pybus,O.G., Carter,D.P., Pullan,S.T., Jacob,S.T., Abdallah,T.M., Gannon,B. and Fletcher,T.E.
EPI_ISL_17460530	Public Health England	Public Health England	Kafetzopoulou,L.E., Efthymiadis,K., Lewandowski,K., Crook,A., Carter,D., Osborne,J., Aarons,E., Hewson,R., Hiscox,J.A., Carroll,M.W., Vipond,R. and Pullan,S.T.
EPI_ISL_17460531, EPI_ISL_17460532, EPI_ISL_17460533, EPI_ISL_17460534, EPI_ISL_17460535	Public Health England	Public Health England	Bower,H., El Karsany,M., Adam,A.A.A.H., Idriss,M.I., Alzain,M.A., Alfakiyousif,M.E.A., Mohamed,R., Mahmoud,I., Albadri,O., Mahmoud,S.A.A., Abdalla,O.I., Eldigail,M., Elagib,N., Arnold,U., Gutierrez,B., Pybus,O.G., Carter,D.P., Pullan,S.T., Jacob,S.T., Abdallah,T.M., Gannon,B. and Fletcher,T.E.
EPI_ISL_17460536	Public Health England	Public Health England	Kafetzopoulou,L.E., Efthymiadis,K., Lewandowski,K., Crook,A., Carter,D., Osborne,J., Aarons,E., Hewson,R., Hiscox,J.A., Carroll,M.W., Vipond,R. and Pullan,S.T.
EPI_ISL_17460537, EPI_ISL_17460538, EPI_ISL_17460539, EPI_ISL_17460540, EPI_ISL_17460541, EPI_ISL_17460542, EPI_ISL_17460543, EPI_ISL_17460544, EPI_ISL_17460545, EPI_ISL_17460546, EPI_ISL_17460547, EPI_ISL_17460548, EPI_ISL_17460549, EPI_ISL_17460550			
see above	Public Health England	Public Health England	Bower,H., El Karsany,M., Adam,A.A.A.H., Idriss,M.I., Alzain,M.A., Alfakiyousif,M.E.A., Mohamed,R., Mahmoud,I., Albadri,O., Mahmoud,S.A.A., Abdalla,O.I., Eldigail,M., Elagib,N., Arnold,U., Gutierrez,B., Pybus,O.G., Carter,D.P., Pullan,S.T., Jacob,S.T., Abdallah,T.M., Gannon,B. and Fletcher,T.E.
EPI_ISL_17460551, EPI_ISL_17460552	Public Health England	Public Health England	Kafetzopoulou,L.E., Efthymiadis,K., Lewandowski,K., Crook,A., Carter,D., Osborne,J., Aarons,E., Hewson,R., Hiscox,J.A., Carroll,M.W., Vipond,R. and Pullan,S.T.
EPI_ISL_17460553, EPI_ISL_17460554, EPI_ISL_17460555, EPI_ISL_17460556, EPI_ISL_17460557, EPI_ISL_17460558, EPI_ISL_17460559, EPI_ISL_17460560, EPI_ISL_17460561, EPI_ISL_17460562, EPI_ISL_17460563, EPI_ISL_17460564, EPI_ISL_17460565, EPI_ISL_17460566, EPI_ISL_17460567, EPI_ISL_17460568, EPI_ISL_17460569, EPI_ISL_17460570, EPI_ISL_17460571, EPI_ISL_17460572, EPI_ISL_17460573, EPI_ISL_17460574, EPI_ISL_17460575, EPI_ISL_17460576, EPI_ISL_17460577, EPI_ISL_17460578, EPI_ISL_17460579, EPI_ISL_17460580, EPI_ISL_17460581			
see above	Public Health England	Public Health England	Bower,H., El Karsany,M., Adam,A.A.A.H., Idriss,M.I., Alzain,M.A., Alfakiyousif,M.E.A., Mohamed,R., Mahmoud,I., Albadri,O., Mahmoud,S.A.A., Abdalla,O.I., Eldigail,M., Elagib,N., Arnold,U., Gutierrez,B., Pybus,O.G., Carter,D.P., Pullan,S.T., Jacob,S.T., Abdallah,T.M., Gannon,B. and Fletcher,T.E.
EPI_ISL_17460582	Public Health England	Public Health England	Kafetzopoulou,L.E., Efthymiadis,K., Lewandowski,K., Crook,A., Carter,D., Osborne,J., Aarons,E., Hewson,R., Hiscox,J.A., Carroll,M.W., Vipond,R. and Pullan,S.T.
EPI_ISL_17460583, EPI_ISL_17460584, EPI_ISL_17460585, EPI_ISL_17460586, EPI_ISL_17460587, EPI_ISL_17460588, EPI_ISL_17460589, EPI_ISL_17460590, EPI_ISL_17460591	Public Health England	Public Health England	Bower,H., El Karsany,M., Adam,A.A.A.H., Idriss,M.I., Alzain,M.A., Alfakiyousif,M.E.A., Mohamed,R., Mahmoud,I., Albadri,O., Mahmoud,S.A.A., Abdalla,O.I., Eldigail,M., Elagib,N., Arnold,U., Gutierrez,B., Pybus,O.G., Carter,D.P., Pullan,S.T., Jacob,S.T., Abdallah,T.M., Gannon,B. and Fletcher,T.E.
EPI_ISL_17460592	Public Health England	Public Health England	Kafetzopoulou,L.E., Efthymiadis,K., Lewandowski,K., Crook,A., Carter,D., Osborne,J., Aarons,E., Hewson,R., Hiscox,J.A., Carroll,M.W., Vipond,R. and Pullan,S.T.
EPI_ISL_17460593, EPI_ISL_17460594, EPI_ISL_17460595, EPI_ISL_17460596, EPI_ISL_17460597, EPI_ISL_17460598, EPI_ISL_17460599, EPI_ISL_17460600, EPI_ISL_17460601, EPI_ISL_17460602, EPI_ISL_17460603, EPI_ISL_17460604, EPI_ISL_17460605, EPI_ISL_17460606, EPI_ISL_17460607, EPI_ISL_17460608, EPI_ISL_17460609, EPI_ISL_17460610, EPI_ISL_17460611, EPI_ISL_17460612, EPI_ISL_17460613, EPI_ISL_17460614, EPI_ISL_17460615, EPI_ISL_17460616, EPI_ISL_17460617, EPI_ISL_17460618, EPI_ISL_17460619, EPI_ISL_17460620, EPI_ISL_17460621, EPI_ISL_17460622, EPI_ISL_17460623, EPI_ISL_17460624, EPI_ISL_17460625, EPI_ISL_17460626, EPI_ISL_17460627, EPI_ISL_17460628, EPI_ISL_17460629, EPI_ISL_17460630, EPI_ISL_17460631			
see above	Public Health Reference Laboratory	Public Health Reference Laboratory	Perti,T., Lucero-Obusan,C.A., Schirmer,P.L., Winters,M.A. and Holodniy,M.
EPI_ISL_17460632	QIMR Berghofer Medical Research Centre	QIMR Berghofer Medical Research Centre	Poo,Y.S., Rudd,P.A., Gardner,J., Wilson,J.A., Larcher,T., Colle,M.A., Le,T.T., Nakaya,H.I., Warrilow,D., Allcock,R., Bielefeldt-Ohmann,H., Schroder,W.A., Khromykh,A.A., Lopez,J.A. and Suhrbier,A.
EPI_ISL_17460633	Queensland Health Forensic and Scientific Services	Queensland Health Forensic and Scientific Services	Pyke,A.T., Moore,P.R. and McMahon,J.
EPI_ISL_17460634	Queensland Health Forensic and Scientific Services	Queensland Health Forensic and Scientific Services	Huang,B., Pyke,A.T., McMahon,J. and Warrilow,D.
EPI_ISL_17460635	Queensland Health Forensic and Scientific Services	Queensland Health Forensic and Scientific Services	Pyke,A.T., Moore,P.R. and McMahon,J.
EPI_ISL_17460636, EPI_ISL_17460637, EPI_ISL_17460638	Queensland Health Forensic and Scientific Services	Queensland Health Forensic and Scientific Services	Pyke,A.T.
EPI_ISL_17460639, EPI_ISL_17460640, EPI_ISL_17460641, EPI_ISL_17460642, EPI_ISL_17460643, EPI_ISL_17460644, EPI_ISL_17460645, EPI_ISL_17460646, EPI_ISL_17460647	Queensland Health Forensic and Scientific Services	Queensland Health Forensic and Scientific Services	Pyke,A.T., Moore,P.R. and McMahon,J.
EPI_ISL_17460648	Queensland Institute of Medical Research	Queensland Institute of Medical Research	Gardner,J., Anraku,I., Le,T.T., Larcher,T., Major,L., Roques,P., Schroder,W.A., Higgs,S. and Suhrbier,A.
EPI_ISL_17460649, EPI_ISL_17460650, EPI_ISL_17460651	RAS Lifesciences	RAS Lifesciences	Tripathi,R., Dayakar,S., Shukla,T., Iravathy,G.K. and Munpally,S.K.
EPI_ISL_17460652	Rajiv Gandhi Centre for Biotechnology	Rajiv Gandhi Centre for Biotechnology	Tsetsarkin,K.A., Chen,R., Yun,R., Rossi,S.L., Plante,K.S., Guerbois,M., Forrester,N., Perng,G.C., Sreekumar,E., Leal,G., Huang,J., Mukhopadhyay,S. and Weaver,S.C.
EPI_ISL_17460653	Rajiv Gandhi Centre for Biotechnology	Rajiv Gandhi Centre for Biotechnology	Sreekumar,E., Babu,J.M., Issac,A., Arathy,D.S., Thomas,A.R., Soman,S.S., Hariharan,R. and Pillai,M.R.
EPI_ISL_17460654	Rajiv Gandhi Centre for Biotechnology	Rajiv Gandhi Centre for Biotechnology	Tsetsarkin,K.A., Chen,R., Yun,R., Rossi,S.L., Plante,K.S., Guerbois,M., Forrester,N., Perng,G.C., Sreekumar,E., Leal,G., Huang,J., Mukhopadhyay,S. and Weaver,S.C.
EPI_ISL_17460655, EPI_ISL_17460656, EPI_ISL_17460657, EPI_ISL_17460658	Rajiv Gandhi Centre for Biotechnology	Rajiv Gandhi Centre for Biotechnology	Sreekumar,E., Babu,J.M., Issac,A., Arathy,D.S., Thomas,A.R., Soman,S.S., Hariharan,R. and Pillai,M.R.
EPI_ISL_17460659	Rajiv Gandhi Centre for Biotechnology	Rajiv Gandhi Centre for Biotechnology	Tsetsarkin,K.A., Chen,R., Yun,R., Rossi,S.L., Plante,K.S., Guerbois,M., Forrester,N., Perng,G.C., Sreekumar,E., Leal,G., Huang,J., Mukhopadhyay,S. and Weaver,S.C.
EPI_ISL_17460660, EPI_ISL_17460661, EPI_ISL_17460662, EPI_ISL_17460663, EPI_ISL_17460664, EPI_ISL_17460665, EPI_ISL_17460666, EPI_ISL_17460667, EPI_ISL_17460668, EPI_ISL_17460669, EPI_ISL_17460670, EPI_ISL_17460671, EPI_ISL_17460672			
see above	Regional Medical Research Center	Regional Medical Research Center	Das,B., Sahu,A., Das,M., Patra,A., Dwibedi,B., Kar,S.K. and Hazra,R.K.
EPI_ISL_17460673	Regional Medical Research Center	Regional Medical Research Center	Sahu,A., Das,B., Das,M., Patra,A., Biswal,S., Kar,S.K. and Hazra,R.K.
EPI_ISL_17460674	Regional Medical Research Center	Regional Medical Research Center	Das,B., Sahu,A., Das,M., Patra,A., Dwibedi,B., Kar,S.K. and Hazra,R.K.
EPI_ISL_17460675, EPI_ISL_17460676	Regional Medical Research Center	Regional Medical Research Center	Sahu,A., Das,B., Das,M., Patra,A., Biswal,S., Kar,S.K. and Hazra,R.K.
EPI_ISL_17460677, EPI_ISL_17460678, EPI_ISL_17460679, EPI_ISL_17460680	Regional Medical Research Center	Regional Medical Research Center	Das,B., Sahu,A., Das,M., Patra,A., Dwibedi,B., Kar,S.K. and Hazra,R.K.
EPI_ISL_17460681, EPI_ISL_17460682	Regional Medical Research Center	Regional Medical Research Center	Sahu,A., Das,B., Das,M., Patra,A., Biswal,S., Kar,S.K. and Hazra,R.K.
EPI_ISL_17460683	Regional Medical Research Center	Regional Medical Research Center	Das,B., Sahu,A., Das,M., Patra,A., Dwibedi,B., Kar,S.K. and Hazra,R.K.
EPI_ISL_17460684	Regional Medical Research Center	Regional Medical Research Center	Sahu,A., Das,B., Das,M., Patra,A., Biswal,S., Kar,S.K. and Hazra,R.K.
EPI_ISL_17460685, EPI_ISL_17460686, EPI_ISL_17460687, EPI_ISL_17460688, EPI_ISL_17460689, EPI_ISL_17460690, EPI_ISL_17460691, EPI_ISL_17460692, EPI_ISL_17460693	Regional Medical Research Center	Regional Medical Research Center	Das,B., Sahu,A., Das,M., Patra,A., Dwibedi,B., Kar,S.K. and Hazra,R.K.
EPI_ISL_17460694, EPI_ISL_17460695, EPI_ISL_17460696	Regional Medical Research Centre	Regional Medical Research Centre	Khan,S.A., Dutta,P., Topno,R., Borah,J., Chowdhury,P. and Mahanta,J.
EPI_ISL_17460697, EPI_ISL_17460698	Regional Medical Research Centre	Regional Medical Research Centre	Dutta,P., Khan,S.A., Chetry,S. and Apum,B.
EPI_ISL_17460699, EPI_ISL_17460700, EPI_ISL_17460701, EPI_ISL_17460702	Regional Medical Research Centre	Regional Medical Research Centre	Dutta,P., Khan,S.A., Chetry,S. and Hazarika,N.K.
EPI_ISL_17460703, EPI_ISL_17460704, EPI_ISL_17460705, EPI_ISL_17460706, EPI_ISL_17460707, EPI_ISL_17460708, EPI_ISL_17460709	Research and Development Foundation	Research and Development Foundation	BROWN,D.T. and HERNANDEZ,R.
EPI_ISL_17460710	Rostock University Medical Center	Rostock University Medical Center	Thomas,S., Rai,J., John,L., Gunther,S., Drosten,C., Putzer,B.M. and Schaefer,S.
EPI_ISL_17460711	Rubber Research Institute of India	Rubber Research Institute of India	Venkatachalam,P., Mohamed Sathik,M.B., Mani,P.P., Kumari,V., Saha,T. and Jacob,J.
EPI_ISL_17460712	SCMS Institute of Bioscience and Biotechnology Research & Development	SCMS Institute of Bioscience and Biotechnology Research & Development	Julia,M.J., Krishna,K.Y., Salini,B. and Mohankumar,C.
EPI_ISL_17460713	SCMS Institute of Bioscience and Biotechnology Research & Development	SCMS Institute of Bioscience and Biotechnology Research & Development	Joseph,A.Y., Babu,V.S., Dev,S.S., Gopalakrishnapai,J., Harish,M., Rajesh,M.D., Anisha,S. and Mohankumar,C.
EPI_ISL_17460714	SCMS Institute of Bioscience and Biotechnology Research & Development	SCMS Institute of Bioscience and Biotechnology Research & Development	Yathi,K.K., Bhasker,S. and Chinnamma,M.
EPI_ISL_17460715, EPI_ISL_17460716	SCMS Institute of Bioscience and Biotechnology Research & Development	SCMS Institute of Bioscience and Biotechnology Research & Development	Joseph,A.Y., Babu,V.S., Dev,S.S., Gopalakrishnapai,J., Harish,M., Rajesh,M.D., Anisha,S. and Mohankumar,C.
EPI_ISL_17460717	Sabin Laboratory	Sabin Laboratory	Barra,G.B.

EPI_ISL_17460718	Sanjay Gandhi Post Graduate Institute of Medical Sciences	Sanjay Gandhi Post Graduate Institute of Medical Sciences	Srivastava,N., Shukla,D., Kakkar,K., Pandey,A., Pandey,S. and Dhole,T.
EPI_ISL_17460719, EPI_ISL_17460720, EPI_ISL_17460721, EPI_ISL_17460722	Sanjay Gandhi Post Graduate Institute of Medical Sciences	Sanjay Gandhi Post Graduate Institute of Medical Sciences	Singh,R.K., Tiwari,S., Mishra,V.K., Tiwari,R. and Dhole,T.N.
EPI_ISL_17460723	Sanjay Gandhi Post Graduate Institute of Medical Sciences	Sanjay Gandhi Post Graduate Institute of Medical Sciences	Srivastava,N., Shukla,D., Kakkar,K., Pandey,A., Pandey,S. and Dhole,T.
EPI_ISL_17460724	Sanjay Gandhi Post Graduate Institute of Medical Sciences	Sanjay Gandhi Post Graduate Institute of Medical Sciences	Tiwari,S., Singh,R.K., Mishra,V.K. and Dhole,T.N.
EPI_ISL_17460725	Sanjay Gandhi Post Graduate Institute of Medical Sciences	Sanjay Gandhi Post Graduate Institute of Medical Sciences	Singh,R.K., Tiwari,S., Mishra,V.K., Tiwari,R. and Dhole,T.N.
EPI_ISL_17460726	Sanjay Gandhi Post Graduate Institute of Medical Sciences	Sanjay Gandhi Post Graduate Institute of Medical Sciences	Srivastava,N., Shukla,D., Kakkar,K., Pandey,A., Pandey,S. and Dhole,T.
EPI_ISL_17460727	Sanjay Gandhi Post Graduate Institute of Medical Sciences	Sanjay Gandhi Post Graduate Institute of Medical Sciences	Tiwari,S., Singh,R.K., Mishra,V.K. and Dhole,T.N.
EPI_ISL_17460728, EPI_ISL_17460729	Sanjay Gandhi Post Graduate Institute of Medical Sciences	Sanjay Gandhi Post Graduate Institute of Medical Sciences	Srivastava,N., Shukla,D., Kakkar,K., Pandey,A., Pandey,S. and Dhole,T.
EPI_ISL_17460730, EPI_ISL_17460731, EPI_ISL_17460732, EPI_ISL_17460733, EPI_ISL_17460734	Sanjay Gandhi Post Graduate Institute of Medical Sciences	Sanjay Gandhi Post Graduate Institute of Medical Sciences	Tiwari,S., Singh,R.K., Mishra,V.K. and Dhole,T.N.
EPI_ISL_17460735	Sanjay Gandhi Postgraduate Institute of Medical Sciences	Sanjay Gandhi Postgraduate Institute of Medical Sciences	Nyari,N., Maan,H.S., Pandey,A., Kumar,M. and Dhole,T.N.
EPI_ISL_17460736	Sanjay Gandhi Postgraduate Institute of Medical Sciences	Sanjay Gandhi Postgraduate Institute of Medical Sciences	Nyari,N., Maan,H.S., Pandey,A. and Dhole,T.N.
EPI_ISL_17460737	Sanjay Gandhi Postgraduate Institute of Medical Sciences	Sanjay Gandhi Postgraduate Institute of Medical Sciences	Nyari,N., Maan,H., Pandey,A. and Dhole,T.N.
EPI_ISL_17460738	Sanjay Gandhi Postgraduate Institute of Medical Sciences	Sanjay Gandhi Postgraduate Institute of Medical Sciences	Nyari,N., Maan,H.S., Pandey,A., Kumar,M. and Dhole,T.N.
EPI_ISL_17460739, EPI_ISL_17460740	Sanjay Gandhi Postgraduate Institute of Medical Sciences	Sanjay Gandhi Postgraduate Institute of Medical Sciences	Nyari,N., Maan,H.S., Kumar,M., Pandey,A. and Dhole,T.N.
EPI_ISL_17460741	Sanjay Gandhi Postgraduate Institute of Medical Sciences	Sanjay Gandhi Postgraduate Institute of Medical Sciences	Nyari,N., Maan,H.S., Pandey,A. and Dhole,T.N.
EPI_ISL_17460742	Sanjay Gandhi Postgraduate Institute of Medical Sciences	Sanjay Gandhi Postgraduate Institute of Medical Sciences	Srivastava,N.N., Kakkar,K. and Dhole,T.N.
EPI_ISL_17460743	Sanjay Gandhi Postgraduate Institute of Medical Sciences	Sanjay Gandhi Postgraduate Institute of Medical Sciences	Nyari,N., Maan,H.S., Pandey,A., Kumar,M. and Dhole,T.N.
EPI_ISL_17460744, EPI_ISL_17460745, EPI_ISL_17460746, EPI_ISL_17460747, EPI_ISL_17460748, EPI_ISL_17460749, EPI_ISL_17460750, EPI_ISL_17460751, EPI_ISL_17460752, EPI_ISL_17460753, EPI_ISL_17460754, EPI_ISL_17460755, EPI_ISL_17460756, EPI_ISL_17460757, EPI_ISL_17460758, EPI_ISL_17460759, EPI_ISL_17460760, EPI_ISL_17460761, EPI_ISL_17460762, EPI_ISL_17460763, EPI_ISL_17460764			
see above	Sapporo Medical University	Sapporo Medical University	Melan,A., Aung,M.S., Khanam,F., Paul,S.K., Riaz,B.K., Tahmina,S., Kabir,M.I., Hossain,M.A. and Kobayashi,N.
EPI_ISL_17460765, EPI_ISL_17460766, EPI_ISL_17460767, EPI_ISL_17460768, EPI_ISL_17460769	Sapporo Medical University School of Medicine, Department of Hygiene	Sapporo Medical University School of Medicine, Department of Hygiene	Ghosh,S., Aung,M.S., Aung,T.S., Win,N. and Kobayashi,N.
EPI_ISL_17460770, EPI_ISL_17460771, EPI_ISL_17460772, EPI_ISL_17460773, EPI_ISL_17460774, EPI_ISL_17460775, EPI_ISL_17460776, EPI_ISL_17460777, EPI_ISL_17460778, EPI_ISL_17460779, EPI_ISL_17460780, EPI_ISL_17460781, EPI_ISL_17460782, EPI_ISL_17460783, EPI_ISL_17460784, EPI_ISL_17460785, EPI_ISL_17460786, EPI_ISL_17460787, EPI_ISL_17460788, EPI_ISL_17460789, EPI_ISL_17460790, EPI_ISL_17460791, EPI_ISL_17460792, EPI_ISL_17460793, EPI_ISL_17460794, EPI_ISL_17460795, EPI_ISL_17460796, EPI_ISL_17460797, EPI_ISL_17460798, EPI_ISL_17460799, EPI_ISL_17460800, EPI_ISL_17460801, EPI_ISL_17460802, EPI_ISL_17460803			
see above	School of Public Health, University of California, Division of Infectious Diseases and Vaccinology	School of Public Health, University of California, Division of Infectious Diseases and Vaccinology	Wang,C., Saborio,S., Gresh,L., Eswarappa,M., Wu,D., Fire,A., Parameswaran,P., Balmaseda,A. and Harris,E.
EPI_ISL_17460804, EPI_ISL_17460805	Shenzhen Center for Disease Control and Prevention	Shenzhen Center for Disease Control and Prevention	Zhang,X., Huang,Y., Huang,S., Liu,Y., Yang,F., Wan,C., Cheng,J. and Zhang,R.
EPI_ISL_17460806	Shenzhen Third People's Hospital	Shenzhen Third People's Hospital	Yang,Y., Bi,Y., Xu,Z., Liu,Y. and Gao,G.F.
EPI_ISL_17460807	Sri Ramachandra Medical College and Research Institute	Sri Ramachandra Medical College and Research Institute	Gopalsamy,S., Mani,M., Seshapoornima,B., Ramya,B., Sheriff,A., Gracy Fathima,S., Gunasekaran,P., Damodharan,J., Muthumani,K. and Srikanth,P.
EPI_ISL_17460808, EPI_ISL_17460809, EPI_ISL_17460810, EPI_ISL_17460811, EPI_ISL_17460812	Sri Ramachandra Medical College and Research Institute	Sri Ramachandra Medical College and Research Institute	Gopalsamy,S., Seshapoornima,B., Monika,M., Ramya,D., Sheriff,A., Gracy Fathima,S., Gunasekaran,P., Damodharan,J., Muthumani,K. and Srikanth,P.
EPI_ISL_17460813	Sri Ramachandra Medical College and Research Institute	Sri Ramachandra Medical College and Research Institute	Gopalsamy,S., Mani,M., Seshapoornima,B., Ramya,B., Sheriff,A., Gracy Fathima,S., Gunasekaran,P., Damodharan,J., Muthumani,K. and Srikanth,P.
EPI_ISL_17460814	Sri Ramachandra Medical College and Research Institute	Sri Ramachandra Medical College and Research Institute	Gopalsamy,S., Seshapoornima,B., Monika,M., Ramya,D., Sheriff,A., Gracy Fathima,S., Gunasekaran,P., Damodharan,J., Muthumani,K. and Srikanth,P.
EPI_ISL_17460815, EPI_ISL_17460816, EPI_ISL_17460817, EPI_ISL_17460818, EPI_ISL_17460819, EPI_ISL_17460820, EPI_ISL_17460821	Sri Ramachandra Medical College and Research Institute	Sri Ramachandra Medical College and Research Institute	Gopalsamy,S., Mani,M., Seshapoornima,B., Ramya,B., Sheriff,A., Gracy Fathima,S., Gunasekaran,P., Damodharan,J., Muthumani,K. and Srikanth,P.
EPI_ISL_17460822	Sri Ramachandra Medical College and Research Institute	Sri Ramachandra Medical College and Research Institute	Gopalsamy,S., Seshapoornima,B., Monika,M., Ramya,D., Sheriff,A., Gracy Fathima,S., Gunasekaran,P., Damodharan,J., Muthumani,K. and Srikanth,P.
EPI_ISL_17460823, EPI_ISL_17460824, EPI_ISL_17460825, EPI_ISL_17460826	Sri Ramachandra Medical College and Research Institute	Sri Ramachandra Medical College and Research Institute	Gopalsamy,S., Mani,M., Seshapoornima,B., Ramya,B., Sheriff,A., Gracy Fathima,S., Gunasekaran,P., Damodharan,J., Muthumani,K. and Srikanth,P.
EPI_ISL_17460827	Sri Ramachandra Medical College and Research Institute	Sri Ramachandra Medical College and Research Institute	Gopalsamy,S., Seshapoornima,B., Monika,M., Ramya,D., Sheriff,A., Gracy Fathima,S., Gunasekaran,P., Damodharan,J., Muthumani,K. and Srikanth,P.
EPI_ISL_17460828, EPI_ISL_17460829, EPI_ISL_17460830	Sri Ramachandra Medical College and Research Institute	Sri Ramachandra Medical College and Research Institute	Gopalsamy,S., Mani,M., Seshapoornima,B., Ramya,B., Sheriff,A., Gracy Fathima,S., Gunasekaran,P., Damodharan,J., Muthumani,K. and Srikanth,P.
EPI_ISL_17460831	Sri Ramachandra Medical College and Research Institute	Sri Ramachandra Medical College and Research Institute	Gopalsamy,S., Seshapoornima,B., Monika,M., Ramya,D., Sheriff,A., Gracy Fathima,S., Gunasekaran,P., Damodharan,J., Muthumani,K. and Srikanth,P.
EPI_ISL_17460832	Sri Ramachandra Medical College and Research Institute	Sri Ramachandra Medical College and Research Institute	Gopalsamy,S., Mani,M., Seshapoornima,B., Ramya,B., Sheriff,A., Gracy Fathima,S., Gunasekaran,P., Damodharan,J., Muthumani,K. and Srikanth,P.
EPI_ISL_17460833	Sri Ramachandra Medical College and Research Institute	Sri Ramachandra Medical College and Research Institute	Gopalsamy,S., Seshapoornima,B., Monika,M., Ramya,D., Sheriff,A., Gracy Fathima,S., Gunasekaran,P., Damodharan,J., Muthumani,K. and Srikanth,P.
EPI_ISL_17460834	Sri Venkateswara University	Sri Venkateswara University	Naresh Kumar,C. and Sai Gopal,D.
EPI_ISL_17460835, EPI_ISL_17460836	Sri Venkateswara University	Sri Venkateswara University	Naresh Kumar,C.V.M. and Sai Gopal,D.V.R.
EPI_ISL_17460837	Sri Venkateswara University	Sri Venkateswara University	Naresh Kumar,C. and Sai Gopal,D.
EPI_ISL_17460838	Sri Venkateswara University	Sri Venkateswara University	Naresh Kumar,C.V.M. and Sai Gopal,D.V.R.
EPI_ISL_17460839	St. Luke's Medical Center	St. Luke's Medical Center	Kawashima,K.D., Suarez,L.A., Labayo,H.K., Liles,V.R., Salvoza,N.C., Klinzing,D.C., Daroy,M.L., Matias,R.R. and Natividad,F.F.
EPI_ISL_17460840	State Resarch Center of Virology and Biotechnology 'Vector'	State Resarch Center of Virology and Biotechnology 'Vector'	Mikriukova,T.P., Bayandin,R.B., Protopopova,E.V., Ternovoy,V.A. and Loktev,V.B.
EPI_ISL_17460841, EPI_ISL_17460842, EPI_ISL_17460843, EPI_ISL_17460844, EPI_ISL_17460845, EPI_ISL_17460846, EPI_ISL_17460847, EPI_ISL_17460848, EPI_ISL_17460849, EPI_ISL_17460850, EPI_ISL_17460851, EPI_ISL_17460852, EPI_ISL_17460853, EPI_ISL_17460854			
see above	Taiwan Centers for Disease Control	Taiwan Centers for Disease Control	Yang,C.F., Su,C.L., Hsu,T.C., Chang,S.F., Lin,C.C., Huang,J.C. and Shu,P.Y.
EPI_ISL_17460855	Taiwan Centers for Disease Control, Research and Diagnostic Center	Taiwan Centers for Disease Control, Research and Diagnostic Center	Yang,C.F., Su,C.L., Hsu,T.C., Chang,S.F., Lin,C.C., Huang,J.C. and Shu,P.Y.
EPI_ISL_17460856, EPI_ISL_17460857, EPI_ISL_17460858, EPI_ISL_17460859, EPI_ISL_17460860, EPI_ISL_17460861, EPI_ISL_17460862, EPI_ISL_17460863, EPI_ISL_17460864, EPI_ISL_17460865, EPI_ISL_17460866, EPI_ISL_17460868, EPI_ISL_17460869, EPI_ISL_17460870, EPI_ISL_17460871, EPI_ISL_17460872, EPI_ISL_17460873, EPI_ISL_17460874, EPI_ISL_17460875, EPI_ISL_17460876, EPI_ISL_17460877, EPI_ISL_17460878, EPI_ISL_17460879, EPI_ISL_17460880, EPI_ISL_17460881			
see above	Taiwan Centers for Disease Control	Taiwan Centers for Disease Control	Yang,C.F., Su,C.L., Hsu,T.C., Chang,S.F., Lin,C.C., Huang,J.C. and Shu,P.Y.
EPI_ISL_17460882, EPI_ISL_17460883, EPI_ISL_17460884, EPI_ISL_17460885, EPI_ISL_17460886, EPI_ISL_17460887, EPI_ISL_17460888, EPI_ISL_17460889, EPI_ISL_17460890, EPI_ISL_17460891, EPI_ISL_17460892, EPI_ISL_17460893, EPI_ISL_17460894, EPI_ISL_17460895, EPI_ISL_17460896, EPI_ISL_17460897, EPI_ISL_17460898, EPI_ISL_17460899, EPI_ISL_17460900, EPI_ISL_17460901			
see above	Taiwan Centers for Disease Control, Center for Diagnostics and Vaccine Development	Taiwan Centers for Disease Control, Center for Diagnostics and Vaccine Development	Chen,M.-Y., Huang,A.S.-E., Yang,C.-F., Hsu,T.-C., Wang,T.-C., Su,C.-L., Chang,M.-C., Peng,S.-H. and Shu,P.-Y.

EPI_ISL_17460902, EPI_ISL_17460903, EPI_ISL_17460904, EPI_ISL_17460905, EPI_ISL_17460906, EPI_ISL_17460907, EPI_ISL_17460908	Taiwan Centers for Disease Control, Research and Diagnostic Center	Taiwan Centers for Disease Control, Research and Diagnostic Center	Huang,J.H., Yang,C.F., Su,C.L., Chang,S.F., Cheng,C.H., Yu,S.K., Lin,C.C. and Shu,P.Y.
EPI_ISL_17460909	Taiwan Centers for Disease Control, Research and Diagnostic Center	Taiwan Centers for Disease Control, Research and Diagnostic Center	Huang,J.H., Su,C.L., Yang,C.F., Liao,T.L., Hsu,T.C., Chang,S.F., Lin,C.C. and Shu,P.Y.
EPI_ISL_17460910, EPI_ISL_17460911	Taiwan Centers for Disease Control, Research and Diagnostic Center	Taiwan Centers for Disease Control, Research and Diagnostic Center	Huang,J.H., Yang,C.F., Su,C.L., Chang,S.F., Cheng,C.H., Yu,S.K., Lin,C.C. and Shu,P.Y.
EPI_ISL_17460912	Taiwan Centers for Disease Control, Research and Diagnostic Center	Taiwan Centers for Disease Control, Research and Diagnostic Center	Chang,S.F., Su,C.L., Shu,P.Y., Yang,C.F., Liao,T.L., Cheng,C.H., Hu,H.C. and Huang,J.H.
EPI_ISL_17460913	Taiwan Centers for Disease Control, Research and Diagnostic Center	Taiwan Centers for Disease Control, Research and Diagnostic Center	Shu,P.Y., Yang,C.F., Su,C.L., Chen,C.Y., Chang,S.F., Tsai,K.H., Cheng,C.H. and Huang,J.H.
EPI_ISL_17460914	Taiwan Centers for Disease Control, Research and Diagnostic Center	Taiwan Centers for Disease Control, Research and Diagnostic Center	Huang,J.H., Yang,C.F., Su,C.L., Chang,S.F., Cheng,C.H., Yu,S.K., Lin,C.C. and Shu,P.Y.
EPI_ISL_17460915	Taiwan Centers for Disease Control, Research and Diagnostic Center	Taiwan Centers for Disease Control, Research and Diagnostic Center	Shu,P.Y., Yang,C.F., Su,C.L., Chen,C.Y., Chang,S.F., Tsai,K.H., Cheng,C.H. and Huang,J.H.
EPI_ISL_17460916, EPI_ISL_17460917, EPI_ISL_17460918, EPI_ISL_17460919	Taiwan Centers for Disease Control, Research and Diagnostic Center	Taiwan Centers for Disease Control, Research and Diagnostic Center	Huang,J.H., Yang,C.F., Su,C.L., Chang,S.F., Cheng,C.H., Yu,S.K., Lin,C.C. and Shu,P.Y.
EPI_ISL_17460920, EPI_ISL_17460921	The Chinese University of Hong Kong	The Chinese University of Hong Kong	Lee,N., Wong,C.K., Lam,W.Y., Wong,A., Lim,W., Lam,C.W., Cockram,C.S., Chan,P.K. and Tang,J.W.
EPI_ISL_17460922, EPI_ISL_17460923	The Chinese University of Hong Kong	The Chinese University of Hong Kong	Ho,D.T.W., Chan,D.P.C., Lam,C.Y., Liang,D.C., Lee,S.S. and Kam,J.K.M.
EPI_ISL_17460924, EPI_ISL_17460925	The Russian Academy of Sciences	The Russian Academy of Sciences	Ignatyev,G., Oksanich,A., Samartseva,T., Kaa,K. and Antonova,L.
EPI_ISL_17460926	Tianjin Centers for Disease Control and Prevention	Tianjin Centers for Disease Control and Prevention	Tan,Z., Lv,L., Xie,T. and Li,L.
EPI_ISL_17460927, EPI_ISL_17460928, EPI_ISL_17460929, EPI_ISL_17460930, EPI_ISL_17460931, EPI_ISL_17460932, EPI_ISL_17460933, EPI_ISL_17460934, EPI_ISL_17460935, EPI_ISL_17460936, EPI_ISL_17460937, EPI_ISL_17460938, EPI_ISL_17460939, EPI_ISL_17460940, EPI_ISL_17460941, EPI_ISL_17460942, EPI_ISL_17460943, EPI_ISL_17460944, EPI_ISL_17460945, EPI_ISL_17460946, EPI_ISL_17460947, EPI_ISL_17460948, EPI_ISL_17460949, EPI_ISL_17460950, EPI_ISL_17460951, EPI_ISL_17460952, EPI_ISL_17460953, EPI_ISL_17460954, EPI_ISL_17460955, EPI_ISL_17460956, EPI_ISL_17460957			
see above	Tohoku University	Tohoku University	Sy,A.K., Saito-Obata,M., Medado,I.A., Tohma,K., Dapat,C., Segubre-Mercado,E., Tandoc,A. 3rd, Lupisan,S. and Oshitani,H.
EPI_ISL_17460958	Tokyo Metropolitan Institute of Public Health	Tokyo Metropolitan Institute of Public Health	Yoshida,I., Kaku,E., Hasegawa,M., Nagashima,M., Shinakai,T. and Sadamasu,K.
EPI_ISL_17460959, EPI_ISL_17460960, EPI_ISL_17460961	Translational Health Science and Technology Institute	Translational Health Science and Technology Institute	Kumar,S., Maurya,R., Chandele,A., Kanakan,A., Chattopadhyay,P., Lodha,R., Kabra,S.K., Singh,B., Pandey,R. and Medigeshi,G.R.
EPI_ISL_17460962	Translational Health Science and Technology Institute	Translational Health Science and Technology Institute	Sreejith,R., Rana,J., Dudha,N., Kumar,K., Gabrani,R., Sharma,S.K., Gupta,A., Vrati,S., Chaudhary,V.K. and Gupta,S.
EPI_ISL_17460963, EPI_ISL_17460964	Translational Health Science and Technology Institute	Translational Health Science and Technology Institute	Kumar,S., Maurya,R., Chandele,A., Kanakan,A., Chattopadhyay,P., Lodha,R., Kabra,S.K., Singh,B., Pandey,R. and Medigeshi,G.R.
EPI_ISL_17460965	Translational Health Science and Technology Institute	Translational Health Science and Technology Institute	Sunil,G., Saurabh,K., Akshay,K., Ranjeet,M., S V,J., Neha,J., Priyanka,M., R,L., Rajesh,P. and Guruprasad,M.R.
EPI_ISL_17460966	Translational Medicine Center, The Sixth People's Hospital of Zhengzhou	Translational Medicine Center, The Sixth People's Hospital of Zhengzhou	Ma,J., Li,S., Huo,Y. and Zheng,L.
EPI_ISL_17460967	Trinity College Dublin	Trinity College Dublin	Logue,C.H., Chamberlain,J.F. and Atkins,G.J.
EPI_ISL_17460968	Trinity College Dublin	Trinity College Dublin	Logue,C.H. and Atkins,G.J.
EPI_ISL_17460969	U.S Naval Medical Research Unit No. 3	U.S Naval Medical Research Unit No. 3	Fahmy,N.T., Klena,J.D., Mohamed,A.S., Zayed,A. and Villinski,J.T.
EPI_ISL_17460970	U.S. Department of Health and Human Services	U.S. Department of Health and Human Services	Graham,B.S., Kanekiyo,M. and Yassine,H.M.
EPI_ISL_17460971, EPI_ISL_17460972	U.S. Department of Health and Human Services	U.S. Department of Health and Human Services	Baptista,C.S., Wu,X. and Munroe,D.J.
EPI_ISL_17460973	U.S. Department of Health and Human Services	U.S. Department of Health and Human Services	Graham,B.S., Kanekiyo,M. and Yassine,H.M.
EPI_ISL_17460974, EPI_ISL_17460975, EPI_ISL_17460976	U.S. Department of Health and Human Services	U.S. Department of Health and Human Services	Baptista,C.S., Wu,X. and Munroe,D.J.
EPI_ISL_17460977	U.S. Department of Health and Human Services	U.S. Department of Health and Human Services	Graham,B.S., Kanekiyo,M. and Yassine,H.M.
EPI_ISL_17460978, EPI_ISL_17460979, EPI_ISL_17460980, EPI_ISL_17460981	U.S. Department of Health and Human Services	U.S. Department of Health and Human Services	Baptista,C.S., Wu,X. and Munroe,D.J.
EPI_ISL_17460982	U.S. Department of Health and Human Services	U.S. Department of Health and Human Services	Graham,B.S., Kanekiyo,M. and Yassine,H.M.
EPI_ISL_17460983	U.S. Department of Health and Human Services	U.S. Department of Health and Human Services	Baptista,C.S., Wu,X. and Munroe,D.J.
EPI_ISL_17460984	US Army Medical Component of the Armed Forces Research Institute of the Medical Sciences	US Army Medical Component of the Armed Forces Research Institute of the Medical Sciences	Chinnawirotpisan,P., Chusri,S., Manasatienkij,W., Joonlasak,K., Huang,A.T., Poolpanichupatam,Y., Lohachanakul,J., Thaisomboonsuk,B., Anthony,J.R., Fernandez,S. and Klungthong,C.
EPI_ISL_17460985	US Army Medical Component of the Armed Forces Research Institute of the Medical Sciences	US Army Medical Component of the Armed Forces Research Institute of the Medical Sciences	Velasco,J.M., Valderama,M.T., Lopez,M.N., Chua,D., Develos,M., Latog,R., Roque,V., Corpuz,J., Tandoc,A., Klungthong,C., Rodpradit,P., Hussem,K., Poolpanichupatam,Y., Macareo,L., Fernandez,S. and Yoon,I.K.
EPI_ISL_17460986, EPI_ISL_17460987, EPI_ISL_17460988, EPI_ISL_17460989	US Army Medical Component of the Armed Forces Research Institute of the Medical Sciences	US Army Medical Component of the Armed Forces Research Institute of the Medical Sciences	Anderson,K.B., Farmer,A., Buddhari,D., Chusri,S., Hortiwakul,T., Charernmak,B., Thaisomboonsuk,B., Wongstitwilairoong,T., Hunsawong,T., Klungthong,C., Chinnawirotpisan,P. and Fernandez,S.
EPI_ISL_17460990	US Army Medical Component of the Armed Forces Research Institute of the Medical Sciences	US Army Medical Component of the Armed Forces Research Institute of the Medical Sciences	Chinnawirotpisan,P., Chusri,S., Manasatienkij,W., Joonlasak,K., Huang,A.T., Poolpanichupatam,Y., Lohachanakul,J., Thaisomboonsuk,B., Anthony,J.R., Fernandez,S. and Klungthong,C.
EPI_ISL_17460991	US Army Medical Component of the Armed Forces Research Institute of the Medical Sciences	US Army Medical Component of the Armed Forces Research Institute of the Medical Sciences	Anderson,K.B., Farmer,A., Buddhari,D., Chusri,S., Hortiwakul,T., Charernmak,B., Thaisomboonsuk,B., Wongstitwilairoong,T., Hunsawong,T., Klungthong,C., Chinnawirotpisan,P. and Fernandez,S.
EPI_ISL_17460992	US Army Medical Component of the Armed Forces Research Institute of the Medical Sciences	US Army Medical Component of the Armed Forces Research Institute of the Medical Sciences	Velasco,J.M., Valderama,M.T., Lopez,M.N., Chua,D., Develos,M., Latog,R., Roque,V., Corpuz,J., Tandoc,A., Klungthong,C., Rodpradit,P., Hussem,K., Poolpanichupatam,Y., Macareo,L., Fernandez,S. and Yoon,I.K.
EPI_ISL_17460993	US Army Medical Component of the Armed Forces Research Institute of the Medical Sciences	US Army Medical Component of the Armed Forces Research Institute of the Medical Sciences	Chusri,S., Siripaitoon,P., Silpapojakul,K., Hortiwakul,T., Charernmak,B., Chinnawirotpisan,P., Nisalak,A., Thaisomboonsuk,B., Klungthong,C., Gibbons,R.V. and Jarman,R.G.
EPI_ISL_17460994, EPI_ISL_17460995, EPI_ISL_17460996, EPI_ISL_17460997, EPI_ISL_17460998	US Army Medical Component of the Armed Forces Research Institute of the Medical Sciences	US Army Medical Component of the Armed Forces Research Institute of the Medical Sciences	Chinnawirotpisan,P., Chusri,S., Manasatienkij,W., Joonlasak,K., Huang,A.T., Poolpanichupatam,Y., Lohachanakul,J., Thaisomboonsuk,B., Anthony,J.R., Fernandez,S. and Klungthong,C.
EPI_ISL_17460999	US Army Medical Component of the Armed Forces Research Institute of the Medical Sciences	US Army Medical Component of the Armed Forces Research Institute of the Medical Sciences	Anderson,K.B., Farmer,A., Buddhari,D., Chusri,S., Hortiwakul,T., Charernmak,B., Thaisomboonsuk,B., Wongstitwilairoong,T., Hunsawong,T., Klungthong,C., Chinnawirotpisan,P. and Fernandez,S.
EPI_ISL_17461000	US Army Medical Component of the Armed Forces Research Institute of the Medical Sciences	US Army Medical Component of the Armed Forces Research Institute of the Medical Sciences	Chinnawirotpisan,P., Chusri,S., Manasatienkij,W., Joonlasak,K., Huang,A.T., Poolpanichupatam,Y., Lohachanakul,J., Thaisomboonsuk,B., Anthony,J.R., Fernandez,S. and Klungthong,C.
EPI_ISL_17461001	US Army Medical Component of the Armed Forces Research Institute of the Medical Sciences	US Army Medical Component of the Armed Forces Research Institute of the Medical Sciences	Chusri,S., Siripaitoon,P., Silpapojakul,K., Hortiwakul,T., Charernmak,B., Chinnawirotpisan,P., Nisalak,A., Thaisomboonsuk,B., Klungthong,C., Gibbons,R.V. and Jarman,R.G.
EPI_ISL_17461002, EPI_ISL_17461003, EPI_ISL_17461004, EPI_ISL_17461005	US Army Medical Component of the Armed Forces Research Institute of the Medical Sciences	US Army Medical Component of the Armed Forces Research Institute of the Medical Sciences	Chinnawirotpisan,P., Chusri,S., Manasatienkij,W., Joonlasak,K., Huang,A.T., Poolpanichupatam,Y., Lohachanakul,J., Thaisomboonsuk,B., Anthony,J.R., Fernandez,S. and Klungthong,C.
EPI_ISL_17461006	US Army Medical Component of the Armed Forces Research Institute of the Medical Sciences	US Army Medical Component of the Armed Forces Research Institute of the Medical Sciences	Yoon,I.K., Alera,M.T., Lago,C., Villa,D., Fernandez,S., Thaisomboonsuk,B., Klungthong,C., Levy,J.R., Velasco,J.M., Roque,V.G. Jr., Macareo,L.R., Nisalak,A. and Srikiatkhachorn,A.
EPI_ISL_17461007	US Army Medical Component of the Armed Forces Research Institute of the Medical Sciences	US Army Medical Component of the Armed Forces Research Institute of the Medical Sciences	Chinnawirotpisan,P., Chusri,S., Manasatienkij,W., Joonlasak,K., Huang,A.T., Poolpanichupatam,Y., Lohachanakul,J., Thaisomboonsuk,B., Anthony,J.R., Fernandez,S. and Klungthong,C.

EPI_ISL_17461008	US Army Medical Component of the Armed Forces Research Institute of the Medical Sciences	US Army Medical Component of the Armed Forces Research Institute of the Medical Sciences	Wangchuk,S., Chinnawirotpisan,P., Dorji,T., Tobgay,T., Dorji,T., Yoon,I.K. and Fernandez,S.
EPI_ISL_17461009, EPI_ISL_17461010, EPI_ISL_17461011, EPI_ISL_17461012, EPI_ISL_17461013	US Army Medical Component of the Armed Forces Research Institute of the Medical Sciences	US Army Medical Component of the Armed Forces Research Institute of the Medical Sciences	Chusri,S., Siripaitoon,P., Silpapojakul,K., Hortiwakul,T., Charernmak,B., Chinnawirotpisan,P., Nisalak,A., Thaisomboonsuk,B., Klungthong,C., Gibbons,R.V. and Jarman,R.G.
EPI_ISL_17461014	US Army Medical Component of the Armed Forces Research Institute of the Medical Sciences	US Army Medical Component of the Armed Forces Research Institute of the Medical Sciences	Chinnawirotpisan,P., Chusri,S., Manasatienkij,W., Joonlasak,K., Huang,A.T., Poolpanichupatam,Y., Lohachanakul,J., Thaisomboonsuk,B., Anthony,J.R., Fernandez,S. and Klungthong,C.
EPI_ISL_17461015	US Army Medical Component of the Armed Forces Research Institute of the Medical Sciences	US Army Medical Component of the Armed Forces Research Institute of the Medical Sciences	Chusri,S., Siripaitoon,P., Silpapojakul,K., Hortiwakul,T., Charernmak,B., Chinnawirotpisan,P., Nisalak,A., Thaisomboonsuk,B., Klungthong,C., Gibbons,R.V. and Jarman,R.G.
EPI_ISL_17461016, EPI_ISL_17461017, EPI_ISL_17461018	US Army Medical Component of the Armed Forces Research Institute of the Medical Sciences	US Army Medical Component of the Armed Forces Research Institute of the Medical Sciences	Yoon,I.K., Alera,M.T., Lago,C., Villa,D., Fernandez,S., Thaisomboonsuk,B., Klungthong,C., Levy,J.R., Velasco,J.M., Roque,V.G. Jr., Macareo,L.R., Nisalak,A. and Srikiatkhachorn,A.
EPI_ISL_17461019, EPI_ISL_17461020, EPI_ISL_17461021	US Army Medical Component of the Armed Forces Research Institute of the Medical Sciences	US Army Medical Component of the Armed Forces Research Institute of the Medical Sciences	Wangchuk,S., Chinnawirotpisan,P., Dorji,T., Tobgay,T., Dorji,T., Yoon,I.K. and Fernandez,S.
EPI_ISL_17461022, EPI_ISL_17461023, EPI_ISL_17461024	US Army Medical Component of the Armed Forces Research Institute of the Medical Sciences	US Army Medical Component of the Armed Forces Research Institute of the Medical Sciences	Chinnawirotpisan,P., Chusri,S., Manasatienkij,W., Joonlasak,K., Huang,A.T., Poolpanichupatam,Y., Lohachanakul,J., Thaisomboonsuk,B., Anthony,J.R., Fernandez,S. and Klungthong,C.
EPI_ISL_17461025, EPI_ISL_17461026, EPI_ISL_17461027, EPI_ISL_17461028, EPI_ISL_17461029	US Army Medical Component of the Armed Forces Research Institute of the Medical Sciences	US Army Medical Component of the Armed Forces Research Institute of the Medical Sciences	Klungthong,C., Rutvisuttinunt,W., Rodpradit,P., Chinnawirotpisan,P., Manasatienkij,W., Alera,M.T., Thaisomboonsuk,B., Melendrez,M.C., Jarman,R.G., Huang,A., Srikiatkhachorn,A., Macareo,L.R., Levy,J.W., Fernandez,S. and Yoon,I.K.
EPI_ISL_17461030, EPI_ISL_17461031	US Army Medical Component of the Armed Forces Research Institute of the Medical Sciences	US Army Medical Component of the Armed Forces Research Institute of the Medical Sciences	Chinnawirotpisan,P., Chusri,S., Manasatienkij,W., Joonlasak,K., Huang,A.T., Poolpanichupatam,Y., Lohachanakul,J., Thaisomboonsuk,B., Anthony,J.R., Fernandez,S. and Klungthong,C.
EPI_ISL_17461032	US Army Medical Component of the Armed Forces Research Institute of the Medical Sciences	US Army Medical Component of the Armed Forces Research Institute of the Medical Sciences	Yoon,I.K., Alera,M.T., Lago,C., Villa,D., Fernandez,S., Thaisomboonsuk,B., Klungthong,C., Levy,J.R., Velasco,J.M., Roque,V.G. Jr., Macareo,L.R., Nisalak,A. and Srikiatkhachorn,A.
EPI_ISL_17461033, EPI_ISL_17461034	US Army Medical Component of the Armed Forces Research Institute of the Medical Sciences	US Army Medical Component of the Armed Forces Research Institute of the Medical Sciences	Wangchuk,S., Chinnawirotpisan,P., Dorji,T., Tobgay,T., Dorji,T., Yoon,I.K. and Fernandez,S.
EPI_ISL_17461035, EPI_ISL_17461036, EPI_ISL_17461037	US Army Medical Component of the Armed Forces Research Institute of the Medical Sciences	US Army Medical Component of the Armed Forces Research Institute of the Medical Sciences	Chinnawirotpisan,P., Chusri,S., Manasatienkij,W., Joonlasak,K., Huang,A.T., Poolpanichupatam,Y., Lohachanakul,J., Thaisomboonsuk,B., Anthony,J.R., Fernandez,S. and Klungthong,C.
EPI_ISL_17461038	US Army Medical Component of the Armed Forces Research Institute of the Medical Sciences	US Army Medical Component of the Armed Forces Research Institute of the Medical Sciences	Chusri,S., Siripaitoon,P., Silpapojakul,K., Hortiwakul,T., Charernmak,B., Chinnawirotpisan,P., Nisalak,A., Thaisomboonsuk,B., Klungthong,C., Gibbons,R.V. and Jarman,R.G.
EPI_ISL_17461039, EPI_ISL_17461040	US Army Medical Research Institute of Infectious Diseases	US Army Medical Research Institute of Infectious Diseases	Glass,P.J., Lybarger,E.A., Teehee,M.L. and Parker,M.D.
EPI_ISL_17461041	US Food and Drug Administration	US Food and Drug Administration	Morrison,T., Hawman,D., Powers,A., Agnihothram,S., Zhao,X., Nagaraj,S., Vavikolanu,K., Nadendla,S., Sadzewicz,L., Tallon,L.J. and Sichtig,H.
EPI_ISL_17461042	US Food and Drug Administration	US Food and Drug Administration	Anez,G., Heisey,D.A. and Rios,M.
EPI_ISL_17461043	US Food and Drug Administration	US Food and Drug Administration	Morrison,T., Hawman,D., Powers,A., Agnihothram,S., Zhao,X., Nagaraj,S., Vavikolanu,K., Nadendla,S., Sadzewicz,L., Tallon,L.J. and Sichtig,H.
EPI_ISL_17461044, EPI_ISL_17461045, EPI_ISL_17461046	US Food and Drug Administration	US Food and Drug Administration	Morrison,T., Hawman,D., Agnihothram,S., Zhao,X., Nagaraj,S., Vavikolanu,K., Nadendla,S., Sadzewicz,L., Tallon,L.J. and Sichtig,H.
EPI_ISL_17461047, EPI_ISL_17461048, EPI_ISL_17461049	Udayana University	Udayana University	Yuliandari,P., Fatmawati,N.N.D., Mayura,I.P.B., Hidayati,W. and Budayanti,N.N.S.
EPI_ISL_17461050	United States Army Medical Research Institute of Infectious Diseases	United States Army Medical Research Institute of Infectious Diseases	Parker,M.D.
EPI_ISL_17461051	Universidad Autonoma de Nuevo Leon	Universidad Autonoma de Nuevo Leon	Rodriguez-Sanchez,I.P., Zamudio-Osuna,M.J., Gomez-Govea,M.A., Ponce-Garcia,G., De-La-O-Cavazos,M.E., Tavitas-Aguilar,M.I., Flores-Suarez,A.E. and Villarrea-Perez,J.Z.
EPI_ISL_17461052	Universidad Autonoma de Nuevo Leon - Facultad de Medicina	Universidad Autonoma de Nuevo Leon - Facultad de Medicina	Galan-Huerta,K.A., Martinez-Landeros,E., Delgado-Gallegos,J.L., Caballero-Sosa,S., Malo-Garcia,I.R., Fernandez-Salas,I., Ramos-Jimenez,J. and Rivas-Estilla,A.M.
EPI_ISL_17461053	Universidad Autonoma de Nuevo Leon - Facultad de Medicina	Universidad Autonoma de Nuevo Leon - Facultad de Medicina	Galan-Huerta,K.A., Delgado-Gallegos,J.L., Martinez-Landeros,E., Caballero-Sosa,S., Fernandez-Salas,I., Ramos-Jimenez,J. and Rivas-Estilla,A.M.
EPI_ISL_17461054, EPI_ISL_17461055, EPI_ISL_17461056, EPI_ISL_17461057, EPI_ISL_17461058, EPI_ISL_17461059	Universidad Autonoma de Nuevo Leon - Facultad de Medicina	Universidad Autonoma de Nuevo Leon - Facultad de Medicina	Galan-Huerta,K.A., Zomosa-Signoret,V.C., Vidaltamayo,R., Caballero-Sosa,S., Fernandez-Salas,I., Ramos-Jimenez,J. and Rivas-Estilla,A.M.
EPI_ISL_17461060	Universidad Autonoma de Nuevo Leon - Facultad de Medicina	Universidad Autonoma de Nuevo Leon - Facultad de Medicina	Galan-Huerta,K.A., Martinez-Landeros,E., Delgado-Gallegos,J.L., Caballero-Sosa,S., Malo-Garcia,I.R., Fernandez-Salas,I., Ramos-Jimenez,J. and Rivas-Estilla,A.M.
EPI_ISL_17461061, EPI_ISL_17461062	Universidad Autonoma de Nuevo Leon - Facultad de Medicina	Universidad Autonoma de Nuevo Leon - Facultad de Medicina	Galan-Huerta,K.A., Zomosa-Signoret,V.C., Vidaltamayo,R., Caballero-Sosa,S., Fernandez-Salas,I., Ramos-Jimenez,J. and Rivas-Estilla,A.M.
EPI_ISL_17461063, EPI_ISL_17461064, EPI_ISL_17461065, EPI_ISL_17461066, EPI_ISL_17461067	Universidad Autonoma de Nuevo Leon - Facultad de Medicina	Universidad Autonoma de Nuevo Leon - Facultad de Medicina	Galan-Huerta,K.A., Martinez-Landeros,E., Delgado-Gallegos,J.L., Caballero-Sosa,S., Malo-Garcia,I.R., Fernandez-Salas,I., Ramos-Jimenez,J. and Rivas-Estilla,A.M.
EPI_ISL_17461068	Universidad Autonoma de Nuevo Leon - Facultad de Medicina	Universidad Autonoma de Nuevo Leon - Facultad de Medicina	Galan-Huerta,K.A., Zomosa-Signoret,V.C., Vidaltamayo,R., Caballero-Sosa,S., Fernandez-Salas,I., Ramos-Jimenez,J. and Rivas-Estilla,A.M.
EPI_ISL_17461069	Universidad Autonoma de Nuevo Leon - Facultad de Medicina	Universidad Autonoma de Nuevo Leon - Facultad de Medicina	Galan-Huerta,K.A., Martinez-Landeros,E., Delgado-Gallegos,J.L., Caballero-Sosa,S., Malo-Garcia,I.R., Fernandez-Salas,I., Ramos-Jimenez,J. and Rivas-Estilla,A.M.
EPI_ISL_17461070	Universidad Autonoma de Nuevo Leon - Facultad de Medicina	Universidad Autonoma de Nuevo Leon - Facultad de Medicina	Galan-Huerta,K.A., Zomosa-Signoret,V.C., Vidaltamayo,R., Caballero-Sosa,S., Fernandez-Salas,I., Ramos-Jimenez,J. and Rivas-Estilla,A.M.
EPI_ISL_17461071	Universidad Autonoma de Nuevo Leon - Facultad de Medicina	Universidad Autonoma de Nuevo Leon - Facultad de Medicina	Galan-Huerta,K.A., Martinez-Landeros,E., Delgado-Gallegos,J.L., Caballero-Sosa,S., Malo-Garcia,I.R., Fernandez-Salas,I., Ramos-Jimenez,J. and Rivas-Estilla,A.M.
EPI_ISL_17461072, EPI_ISL_17461073, EPI_ISL_17461074, EPI_ISL_17461075, EPI_ISL_17461076, EPI_ISL_17461077, EPI_ISL_17461078	Universidad Autonoma de Nuevo Leon - Facultad de Medicina	Universidad Autonoma de Nuevo Leon - Facultad de Medicina	Galan-Huerta,K.A., Zomosa-Signoret,V.C., Vidaltamayo,R., Caballero-Sosa,S., Fernandez-Salas,I., Ramos-Jimenez,J. and Rivas-Estilla,A.M.
EPI_ISL_17461079	Universidad Autonoma de Nuevo Leon - Facultad de Medicina	Universidad Autonoma de Nuevo Leon - Facultad de Medicina	Galan-Huerta,K.A., Delgado-Gallegos,J.L., Martinez-Landeros,E., Caballero-Sosa,S., Fernandez-Salas,I., Ramos-Jimenez,J. and Rivas-Estilla,A.M.
EPI_ISL_17461080, EPI_ISL_17461081, EPI_ISL_17461082	Universidad Autonoma de Nuevo Leon - Facultad de Medicina	Universidad Autonoma de Nuevo Leon - Facultad de Medicina	Galan-Huerta,K.A., Zomosa-Signoret,V.C., Vidaltamayo,R., Caballero-Sosa,S., Fernandez-Salas,I., Ramos-Jimenez,J. and Rivas-Estilla,A.M.
EPI_ISL_17461083	Universidad Autonoma de Nuevo Leon - Facultad de Medicina	Universidad Autonoma de Nuevo Leon - Facultad de Medicina	Galan-Huerta,K.A., Delgado-Gallegos,J.L., Martinez-Landeros,E., Caballero-Sosa,S., Fernandez-Salas,I., Ramos-Jimenez,J. and Rivas-Estilla,A.M.
EPI_ISL_17461084	Universidad Autonoma de Nuevo Leon - Facultad de Medicina	Universidad Autonoma de Nuevo Leon - Facultad de Medicina	Galan-Huerta,K.A., Zomosa-Signoret,V.C., Vidaltamayo,R., Caballero-Sosa,S., Fernandez-Salas,I., Ramos-Jimenez,J. and Rivas-Estilla,A.M.
EPI_ISL_17461085, EPI_ISL_17461086	Universidad Autonoma de Nuevo Leon - Facultad de Medicina	Universidad Autonoma de Nuevo Leon - Facultad de Medicina	Galan-Huerta,K.A., Delgado-Gallegos,J.L., Martinez-Landeros,E., Caballero-Sosa,S., Fernandez-Salas,I., Ramos-Jimenez,J. and Rivas-Estilla,A.M.
EPI_ISL_17461087	Universidad Autonoma de Nuevo Leon - Facultad de Medicina	Universidad Autonoma de Nuevo Leon - Facultad de Medicina	Galan-Huerta,K.A., Zomosa-Signoret,V.C., Vidaltamayo,R., Caballero-Sosa,S., Fernandez-Salas,I., Ramos-Jimenez,J. and Rivas-Estilla,A.M.
EPI_ISL_17461088	Universidad Autonoma de Nuevo Leon - Facultad de Medicina	Universidad Autonoma de Nuevo Leon - Facultad de Medicina	Galan-Huerta,K.A., Martinez-Landeros,E., Delgado-Gallegos,J.L., Caballero-Sosa,S., Malo-Garcia,I.R., Fernandez-Salas,I., Ramos-Jimenez,J. and Rivas-Estilla,A.M.
EPI_ISL_17461089	Universidad El Bosque	Universidad El Bosque	Archila,E., Lopez,L.S., Castellanos,J.E. and Calvo,E.P.
EPI_ISL_17461090	Universidad El Bosque	Universidad El Bosque	Calvo,E.P. and Castellanos,J.E.
EPI_ISL_17461091, EPI_ISL_17461092	Universidad Industrial de Santander	Universidad Industrial de Santander	Ocazionez,R.E. and Maria Fernanda,C.
EPI_ISL_17461093	Universidad de Cordoba	Universidad de Cordoba	Mattar,S., Villero,Y., Puerta,A., Arrieta,G. and Muskus,C.

EPI_ISL_17461094	Universidad de Cordoba	Universidad de Cordoba	Serpa,J.A.
EPI_ISL_17461095, EPI_ISL_17461096, EPI_ISL_17461097, EPI_ISL_17461098	Universidad de Cordoba	Universidad de Cordoba	Mattar,S., Villero,Y., Puerta,A., Arrieta,G. and Muskus,C.
EPI_ISL_17461099	Universidad de Cordoba	Universidad de Cordoba	Aponte Serpa,J.D., Mattar Velilla,S.D.C., Gonzalez Tous,M., Contreras Martinez,H.I. and Aleman Romero,A.L.
EPI_ISL_17461100, EPI_ISL_17461101, EPI_ISL_17461102	Universidad de Cordoba	Universidad de Cordoba	Mattar,S., Villero,Y., Puerta,A., Arrieta,G. and Muskus,C.
EPI_ISL_17461103, EPI_ISL_17461104	Universidad de Cordoba	Universidad de Cordoba	Cordoba,U.
EPI_ISL_17461105	Universidad de Cordoba	Universidad de Cordoba	Mattar,S., Villero,Y., Puerta,A., Arrieta,G. and Muskus,C.
EPI_ISL_17461106	Universidad de Cordoba	Universidad de Cordoba	Serpa,J.A.
EPI_ISL_17461107	Universidad de Cordoba	Universidad de Cordoba	Mattar,S., Villero,Y., Puerta,A., Arrieta,G. and Muskus,C.
EPI_ISL_17461108	Universidad de Cordoba	Universidad de Cordoba	Aponte Serpa,J.D., Gonzalez Tous,M., Mattar Velilla,S.D.C. and Contreras Martinez,H.I.
EPI_ISL_17461109, EPI_ISL_17461110	Universidad de Cordoba	Universidad de Cordoba	Mattar,S., Villero,Y., Puerta,A., Arrieta,G. and Muskus,C.
EPI_ISL_17461111, EPI_ISL_17461112, EPI_ISL_17461113	Universidad de Cordoba	Universidad de Cordoba	Aponte Serpa,J.
EPI_ISL_17461114, EPI_ISL_17461115	Universidad de Cordoba	Universidad de Cordoba	Mattar,S., Villero,Y., Puerta,A., Arrieta,G. and Muskus,C.
EPI_ISL_17461116	Universidad de Cordoba	Universidad de Cordoba	Cordoba,U.
EPI_ISL_17461117	Universidad de Cordoba	Universidad de Cordoba	Mattar,S., Villero,Y., Puerta,A., Arrieta,G. and Muskus,C.
EPI_ISL_17461118	Universidad de Cordoba	Universidad de Cordoba	Contreras Martinez,H.I.
EPI_ISL_17461119	Universidad de Cordoba	Universidad de Cordoba	Mattar,S., Miranda,J., Pinzon,H., Tique,V., Aponte,J., Contreras,H., Bolanos,A., Arieta,G., Gonzalez,M., Barrios,K., Alvarez,J. and Aleman,A.
EPI_ISL_17461120	Universidad de Cordoba	Universidad de Cordoba	Mattar,S., Villero,Y., Puerta,A., Arrieta,G. and Muskus,C.
EPI_ISL_17461121, EPI_ISL_17461122, EPI_ISL_17461123	Universidad de Sucre	Universidad de Sucre	Camacho,E., Paternina-Gomez,M., Cabarcas,S., Martinez,S. and Blanco,P.
EPI_ISL_17461124, EPI_ISL_17461125, EPI_ISL_17461126, EPI_ISL_17461127, EPI_ISL_17461128, EPI_ISL_17461129, EPI_ISL_17461130, EPI_ISL_17461131, EPI_ISL_17461132, EPI_ISL_17461133, EPI_ISL_17461134			
see above	Universidade Ceuma	Universidade Ceuma	de Melo,B.O., Junior,A.D.S., Costa,A.K.S., da Silva,L.C.N., Turri,Rd.J.G., Zagmignan,A. and Bomfim,M.R.Q.
EPI_ISL_17461135	Universidade Ceuma	Universidade Ceuma	Melo,B.O., Silva Pinto,C.M.F., Turri,R.D.J.G. and Bomfim,M.R.Q.
EPI_ISL_17461136, EPI_ISL_17461137, EPI_ISL_17461138, EPI_ISL_17461139, EPI_ISL_17461140, EPI_ISL_17461141, EPI_ISL_17461142, EPI_ISL_17461143, EPI_ISL_17461144, EPI_ISL_17461145, EPI_ISL_17461146, EPI_ISL_17461147, EPI_ISL_17461148, EPI_ISL_17461149, EPI_ISL_17461150, EPI_ISL_17461151, EPI_ISL_17461152, EPI_ISL_17461153, EPI_ISL_17461154, EPI_ISL_17461155, EPI_ISL_17461156, EPI_ISL_17461157, EPI_ISL_17461158, EPI_ISL_17461159, EPI_ISL_17461160, EPI_ISL_17461161, EPI_ISL_17461162, EPI_ISL_17461163, EPI_ISL_17461164, EPI_ISL_17461165, EPI_ISL_17461166, EPI_ISL_17461167, EPI_ISL_17461168, EPI_ISL_17461169, EPI_ISL_17461170, EPI_ISL_17461171, EPI_ISL_17461172, EPI_ISL_17461173			
see above	Universidade Ceuma	Universidade Ceuma	de Melo,B.O., Junior,A.D.S., Costa,A.K.S., da Silva,L.C.N., Turri,Rd.J.G., Zagmignan,A. and Bomfim,M.R.Q.
EPI_ISL_17461174, EPI_ISL_17461175, EPI_ISL_17461176, EPI_ISL_17461177, EPI_ISL_17461178	Universidade Estadual de Maringa	Universidade Estadual de Maringa	Moreira,D.C., Junior,F.F.D., Junior,J.R.P., Rando,F.S., Jorge,F.A., Presibella,M.M., Riediger,I.N., Fernandez,M.A., Delatorre,E., Souza,T.M.L. and Bertolini,D.A.
EPI_ISL_17461179, EPI_ISL_17461180, EPI_ISL_17461181, EPI_ISL_17461182, EPI_ISL_17461183, EPI_ISL_17461184, EPI_ISL_17461185, EPI_ISL_17461186, EPI_ISL_17461187, EPI_ISL_17461188, EPI_ISL_17461189, EPI_ISL_17461190, EPI_ISL_17461191, EPI_ISL_17461192, EPI_ISL_17461193, EPI_ISL_17461194, EPI_ISL_17461195, EPI_ISL_17461196, EPI_ISL_17461197, EPI_ISL_17461198, EPI_ISL_17461199, EPI_ISL_17461200, EPI_ISL_17461201, EPI_ISL_17461202, EPI_ISL_17461203, EPI_ISL_17461204, EPI_ISL_17461205, EPI_ISL_17461206, EPI_ISL_17461207, EPI_ISL_17461208, EPI_ISL_17461209, EPI_ISL_17461210, EPI_ISL_17461211, EPI_ISL_17461212, EPI_ISL_17461213, EPI_ISL_17461214			
see above	Universidade Federal de Alagoas	Universidade Federal de Alagoas	Taniele-Silva,J., de Oliveira,M.J.T.T.C., de Souza,S.J.M., Brandao,J.A., Anderson,L. and Bassi,E.J.
EPI_ISL_17461215, EPI_ISL_17461216	Universidade Federal de Mato Grosso	Universidade Federal de Mato Grosso	Ramirez Pavon,J.A., Neves,N.A.S., de Azevedo,F.K., de Figueiredo Junior,J.A.B., Nunes,M.R.T. and Slhessarenko,R.D.
EPI_ISL_17461217	Universidade Federal de Mato Grosso	Universidade Federal de Mato Grosso	da Silva Pessoa Vieira,C.J., Ferreira da Silva,D.J., Rigotti Kubiszeski,J., Ceschini Machado,L., Pena,L.J., de Morais Bronzoni,R.V. and da Luz Wallau,G.
EPI_ISL_17461218, EPI_ISL_17461219, EPI_ISL_17461220, EPI_ISL_17461221	Universidade Federal de Mato Grosso	Universidade Federal de Mato Grosso	Ramirez Pavon,J.A., Neves,N.A.S., de Azevedo,F.K., de Figueiredo Junior,J.A.B., Nunes,M.R.T. and Slhessarenko,R.D.
EPI_ISL_17461222	Universidade Federal de Mato Grosso, Laboratorio de Virologia	Universidade Federal de Mato Grosso, Laboratorio de Virologia	Perin,M.Y., Genaro,M.S., Merchi,M.S., Slhessarenko,R.D. and Cosso,I.S.
EPI_ISL_17461223, EPI_ISL_17461224, EPI_ISL_17461225, EPI_ISL_17461226, EPI_ISL_17461227, EPI_ISL_17461228, EPI_ISL_17461229, EPI_ISL_17461230, EPI_ISL_17461231, EPI_ISL_17461232, EPI_ISL_17461233, EPI_ISL_17461234, EPI_ISL_17461235, EPI_ISL_17461236			
see above	Universidade de Sao Paulo	Universidade de Sao Paulo	Lazari,C.D.S., Ramundo,M.S., Ten-Caten,F., Bressan,C.S., de Filippis,A.M.B., Manuli,E.R., de Moraes,I., Pereira,G.M., Cortes,M.F., Candido,D.D.S., Gerber,A.L., Guimaraes,A.P., Faria,N.R., Nakaya,H.I., Vasconcelos,A.T.R., Brasil,P., Paranhos-Baccala,G. and Sabino,E.C.
EPI_ISL_17461237	Universidade de Sao Paulo	Universidade de Sao Paulo	Costa-da-Silva,A.L., Ioshino,R.S., Petersen,V., Lima,A.F., Duarte,D.C., Cunha,M.P., Wiley,M.R., Ladner,J.T., Prieto,K., Palacios,G., Suesdek,L., Zanotto,P.M.A. and Capurro,M.L.
EPI_ISL_17461238, EPI_ISL_17461239, EPI_ISL_17461240, EPI_ISL_17461241, EPI_ISL_17461242, EPI_ISL_17461243, EPI_ISL_17461244, EPI_ISL_17461245	Universidade de Sao Paulo	Universidade de Sao Paulo	Lazari,C.D.S., Ramundo,M.S., Ten-Caten,F., Bressan,C.S., de Filippis,A.M.B., Manuli,E.R., de Moraes,I., Pereira,G.M., Cortes,M.F., Candido,D.D.S., Gerber,A.L., Guimaraes,A.P., Faria,N.R., Nakaya,H.I., Vasconcelos,A.T.R., Brasil,P., Paranhos-Baccala,G. and Sabino,E.C.
EPI_ISL_17461246	Universidade de Sao Paulo, Laboratorio de Biologia Molecular	Universidade de Sao Paulo, Laboratorio de Biologia Molecular	Souza,J.V.C., Santos,H.O., Leite,A.B., Giovanetti,M., Bezerra,R.D.S., Carvalho,E., Bernardino,J.S.T., Viala,V.L., Haddad,R., Ciccozzi,M., Alcantara,L.C.J., Sampaio,S.C., Covas,D.T., Kashima,S., Elias,M.C. and Slavov,S.N.
EPI_ISL_17461247	Universiti Malaysia Sarawak	Universiti Malaysia Sarawak	Andrew,A. and Sum,M.S.H.
EPI_ISL_17461248, EPI_ISL_17461249, EPI_ISL_17461250, EPI_ISL_17461251, EPI_ISL_17461252, EPI_ISL_17461253, EPI_ISL_17461254, EPI_ISL_17461255, EPI_ISL_17461256, EPI_ISL_17461257	Universiti Malaysia Sarawak	Universiti Malaysia Sarawak	Perera,D.
EPI_ISL_17461258, EPI_ISL_17461259	University College Dublin	University College Dublin	McCartin,A.M., Hall,N., Duffy,M., Dunford,L., Conway,A., Connell,J., Carr,M.J. and Hall,W.W.
EPI_ISL_17461260, EPI_ISL_17461261, EPI_ISL_17461262, EPI_ISL_17461263	University Malaya	University Malaya	Sam,I.C., Chan,Y.F., Chan,S.Y., Loong,S.K., Chin,H.K., Hooi,P.S., Ganeswrie,R. and Abubakar,S.
EPI_ISL_17461264	University Malaya	University Malaya	Sam,I.C., Chan,Y.F., Loong,S.K., Chan,S.Y., Khor,C.S., Chiam,C.W., Chang,R. and AbuBakar,S.
EPI_ISL_17461265	University Malaya	University Malaya	Sam,I.C., Chan,Y.F., Chan,S.Y., Loong,S.K., Chin,H.K., Hooi,P.S., Ganeswrie,R. and Abubakar,S.
EPI_ISL_17461266	University Malaya	University Malaya	Sam,I.C., Kamarulzaman,A., Ong G,S.Y., Veriah,R.S., Ponnampalavanar,S., Chan,Y.F. and Abu Bakar,S.
EPI_ISL_17461267	University Malaya	University Malaya	Sam,I.C., Chan,Y.F., Chan,S.Y., Loong,S.K., Chin,H.K., Hooi,P.S., Ganeswrie,R. and Abubakar,S.
EPI_ISL_17461268	University Malaya	University Malaya	Sam,I.C., Chan,Y.F., Loong,S.K., Chan,S.Y., Khor,C.S., Chiam,C.W., Chang,R. and AbuBakar,S.
EPI_ISL_17461269, EPI_ISL_17461270	University Malaya	University Malaya	Sam,I.C., Chan,Y.F., Chan,S.Y., Loong,S.K., Chin,H.K., Hooi,P.S., Ganeswrie,R. and Abubakar,S.
EPI_ISL_17461271, EPI_ISL_17461272, EPI_ISL_17461273, EPI_ISL_17461274, EPI_ISL_17461275, EPI_ISL_17461276	University Malaya	University Malaya	Sam,I.C., Chan,Y.F., Loong,S.K., Chan,S.Y., Khor,C.S., Chiam,C.W., Chang,R. and AbuBakar,S.
EPI_ISL_17461277, EPI_ISL_17461278, EPI_ISL_17461279	University Malaya	University Malaya	Sam,I.C., Chan,Y.F., Chan,S.Y., Loong,S.K., Chin,H.K., Hooi,P.S., Ganeswrie,R. and Abubakar,S.
EPI_ISL_17461280	University Malaya	University Malaya	Sam,I.C., Chan,Y.F., Loong,S.K., Chan,S.Y., Khor,C.S., Chiam,C.W., Chang,R. and AbuBakar,S.
EPI_ISL_17461281, EPI_ISL_17461282	University Malaya	University Malaya	Sam,I.C., Chan,Y.F., Chan,S.Y., Loong,S.K., Chin,H.K., Hooi,P.S., Ganeswrie,R. and Abubakar,S.
EPI_ISL_17461283	University Wuerzburg	University Wuerzburg	Kowalzik,S., Xuan,N.V., Weissbrich,B., Scheiner,B., Schied,T., Drosten,C., Muller,A., Stich,A., Rethwilm,A. and Bodem,J.
EPI_ISL_17461284	University of Bologna	University of Bologna	Venturi,G., Aberle,S.W., Avsic-Zupanc,T., Barzon,L., Batejat,C., Burdino,E., Carletti,F., Charrel,R., Christova,I., Connell,J., Corman,V.M., Emmanouil,M., Jaaskelainen,A.J., Kurolt,I., Lustig,Y., Martinez,M.J., Koopmans,M., Nagy,O., Nguyen,T., Papa,A., Perez-Ruiz,M., Pfeffer,M., Protic,J., Reimerink,J., Rossini,G., Sanchez-Seco Farinas,M.P., Schmidt-Chanasit,J., Soderholm,S., Sudre,B., Van Esbroeck,M. and Reusken,C.B.
EPI_ISL_17461285, EPI_ISL_17461286	University of Bologna	University of Bologna	Rossini,G., Gaibani,P., Vocale,C., Finarelli,A.C. and Landini,M.P.
EPI_ISL_17461287	University of Bonn Medical Centre	University of Bonn Medical Centre	Panning,M., Hess,M., Fischer,W., Grywna,K., Pfeffer,M. and Drosten,C.
EPI_ISL_17461288	University of Bonn Medical Centre	University of Bonn Medical Centre	Moreira-Soto,A. and Drexler,J.F.
EPI_ISL_17461289, EPI_ISL_17461290, EPI_ISL_17461291, EPI_ISL_17461292, EPI_ISL_17461293, EPI_ISL_17461294, EPI_ISL_17461295, EPI_ISL_17461296, EPI_ISL_17461297, EPI_ISL_17461298, EPI_ISL_17461299, EPI_ISL_17461300			
see above	University of Bonn Medical Centre	University of Bonn Medical Centre	Grywna,K., Drosten,C. and Kummerer,B.M.
EPI_ISL_17461301, EPI_ISL_17461302, EPI_ISL_17461303, EPI_ISL_17461304, EPI_ISL_17461305, EPI_ISL_17461306	University of Brasilia	University of Brasilia	Vasconcellos,A.F., Silva,J.M., de Oliveira,A.S., Prado,P.S., Nagata,T. and Resende,R.O.
EPI_ISL_17461307, EPI_ISL_17461308	University of California	University of California	Chiu,C.Y., Bres,V., Yu,G., Krysztof,D., Naccache,S.N., Lee,D., Pfeil,J., Linnen,J.M. and Stramer,S.L.
EPI_ISL_17461309	University of California	University of California	Lyra,P.P., Campos,G.S., Bandeira,I.D., Sardi,S.I., Costa,L.F., Santos,F.R., Ribeiro,C.A., Jardim,A.M., Santiago,A.C., de Oliveira,P.M. and Moreira,L.M.
EPI_ISL_17461310	University of California	University of California	Chiu,C.Y., Bres,V., Yu,G., Krysztof,D., Naccache,S.N., Lee,D., Pfeil,J., Linnen,J.M. and Stramer,S.L.
EPI_ISL_17461311	University of California	University of California	Lyra,P.P., Campos,G.S., Bandeira,I.D., Sardi,S.I., Costa,L.F., Santos,F.R., Ribeiro,C.A., Jardim,A.M., Santiago,A.C., de Oliveira,P.M. and Moreira,L.M.
EPI_ISL_17461312, EPI_ISL_17461313, EPI_ISL_17461314, EPI_ISL_17461315, EPI_ISL_17461316, EPI_ISL_17461317, EPI_ISL_17461318, EPI_ISL_17461319, EPI_ISL_17461321, EPI_ISL_17461322, EPI_ISL_17461323, EPI_ISL_17461324, EPI_ISL_17461325, EPI_ISL_17461326, EPI_ISL_17461327, EPI_ISL_17461328, EPI_ISL_17461329, EPI_ISL_17461330, EPI_ISL_17461331			
see above	University of California San Francisco	University of California San Francisco	Saha,S., Ramesh,A., Kalantar,K., Malaker,R., Hasanuzzaman,M., Khan,L.M., Mayday,M.Y., Sajib,M.S.I., Li,L.M., Langelier,C., Rahman,H., Crawford,E.D., Tato,C.M., Islam,M., Juan,Y.F., de Bourcy,C., Dimitrov,B., Wang,J., Tang,J., Sheu,J., Egger,R., De Carvalho,T.R., Wilson,M.R., Saha,S.K. and DeRisi,J.L.
EPI_ISL_17461332	University of California, Davis	University of California, Davis	PREDICT Consortium

EPI_ISL_17461333	University of Colorado Denver	University of Colorado Denver	Morrison,T.E., Oko,L., Montgomery,S.A., Whitmore,A.C., Lotstein,A.R., Gunn,B.M., Elmore,S.A. and Heise,M.T.
EPI_ISL_17461334, EPI_ISL_17461335, EPI_ISL_17461336	University of Florida	University of Florida	Prakoso,D., Barr,K.L., Imtiaz,K., Farooqi,J., Malik,F., Khan,E. and Long,M.T.
EPI_ISL_17461337, EPI_ISL_17461338	HUSLAB Tullinpuomi Laboratory	HUSLAB Tullinpuomi Laboratory	Venturi,G., Aberle,S., AvsicZupanc,T., Barzon,L., Batejat,C., Burdino,E., Charrel,R., Castilletti,C., Christova,I., Hewson,R., Jaaskelainen,A.J., Kurolt,I., Lustig,Y., Mary,E., Papa,A., Perez-Ruiz,M., Martinez,M.J., Molenkamp,R., Nagy,O., Reimerink,J., Rossini,G., Paz Sanches-Seco Farinas,M., Schmidt-Chanasit,J., Soderholm,S., van Esbroeck,M., Group,C.-W. and Reusken,C.B.
EPI_ISL_17461339, EPI_ISL_17461340	Department of Virology, Haartman Institute, Faculty of Medicine, University of Helsinki	Department of Virology, Haartman Institute, Faculty of Medicine, University of Helsinki	Kurkela,S., Sane,J., Deren,E., Huhtamo,E., Suomalainen,I., Kantele,A. and Vapalahti,O.
EPI_ISL_17461341, EPI_ISL_17461342, EPI_ISL_17461343, EPI_ISL_17461344, EPI_ISL_17461345, EPI_ISL_17461346, EPI_ISL_17461347, EPI_ISL_17461348, EPI_ISL_17461349, EPI_ISL_17461350	University of Khartoum	University of Khartoum	Aradainb,I.E.
EPI_ISL_17461351, EPI_ISL_17461352	University of Madras	University of Madras	Sangeetha,K., Indu,P. and Rajarajan,S.
EPI_ISL_17461353, EPI_ISL_17461354, EPI_ISL_17461355, EPI_ISL_17461356, EPI_ISL_17461357, EPI_ISL_17461358, EPI_ISL_17461359, EPI_ISL_17461360, EPI_ISL_17461361, EPI_ISL_17461362, EPI_ISL_17461363, EPI_ISL_17461364, EPI_ISL_17461365, EPI_ISL_17461366, EPI_ISL_17461367			
see above	University of Malaya, Faculty of Medicine	University of Malaya, Faculty of Medicine	AbuBakar,S., Sam,I.C., Wong,P.F., MatRahim,N., Hooi,P.S. and Roslan,N.
EPI_ISL_17461368, EPI_ISL_17461369, EPI_ISL_17461370	University of Nebraska Medical Center	University of Nebraska Medical Center	Agbodzi,B., Berlange Sado Yousseu,F., Brice Nemg Simo,F., Kumordjie,S., Yeboah,C., Mosore,M.-T., Bentil,R.E., Prieto,K., Attram,N., Nimo-Paintstil,S., Fox,A.T., Bonney,J.H.K., Ampofo,W., Wolfe,D.M., Wiley,M., Demanou,M. and Letizia,A.G.
EPI_ISL_17461371	University of North Carolina	University of North Carolina	Madden,E.A.
EPI_ISL_17461372	University of North Carolina	University of North Carolina	Jones,J.E., Long,K.M., Whitmore,A.C., Sanders,W., Thurlow,L.R., Brown,J.A., Morrison,C.R., Vincent,H., Peck,K.M., Browning,C., Moorman,N., Lim,J.K. and Heise,M.T.
EPI_ISL_17461373, EPI_ISL_17461374, EPI_ISL_17461375, EPI_ISL_17461376, EPI_ISL_17461377, EPI_ISL_17461378, EPI_ISL_17461379, EPI_ISL_17461380	University of North Carolina	University of North Carolina	Sanders,W., Long,K., Rivera,B., Vincent,H.A., Heise,M.T., Rodriguez-Orengo,J.F. and Moorman,N.J.
EPI_ISL_17461381	University of North Carolina	University of North Carolina	Madden,E.A.
EPI_ISL_17461382, EPI_ISL_17461383	University of North Carolina	University of North Carolina	Sanders,W., Long,K., Rivera,B., Vincent,H.A., Heise,M.T., Rodriguez-Orengo,J.F. and Moorman,N.J.
EPI_ISL_17461384	University of North Carolina	University of North Carolina	Madden,E.A.
EPI_ISL_17461385, EPI_ISL_17461386, EPI_ISL_17461387, EPI_ISL_17461388, EPI_ISL_17461389	University of North Carolina	University of North Carolina	Sanders,W., Long,K., Rivera,B., Vincent,H.A., Heise,M.T., Rodriguez-Orengo,J.F. and Moorman,N.J.
EPI_ISL_17461390, EPI_ISL_17461391, EPI_ISL_17461392, EPI_ISL_17461393, EPI_ISL_17461394, EPI_ISL_17461395, EPI_ISL_17461396, EPI_ISL_17461397, EPI_ISL_17461398	University of Oklahoma	University of Oklahoma	Franca,C.M., Loayza,R. and Miller,M.J.
EPI_ISL_17461399, EPI_ISL_17461400, EPI_ISL_17461401, EPI_ISL_17461402, EPI_ISL_17461403, EPI_ISL_17461404, EPI_ISL_17461405, EPI_ISL_17461406, EPI_ISL_17461407, EPI_ISL_17461408, EPI_ISL_17461409, EPI_ISL_17461410, EPI_ISL_17461411, EPI_ISL_17461412, EPI_ISL_17461413			
see above	University of Pune	University of Pune	Yadav,P., Shouche,Y.S., Munot,H.P., Mishra,A.C. and Mourya,D.T.
EPI_ISL_17461414	University of Sao Paulo	University of Sao Paulo	Cunha,M.S., Maeda,A.Y., Bisordi,I., Rocco,I.M., Silva,F.G., Souza,R.P., Coimbra,T.L.M., Nogueira,J.S., Kisielius,J.J., Silveira,V.R., Santos,S.J.S., Oliveira,A.L.R., Esposito,D.L.A., Fonseca,B.A.L. and Suzuki,A.
EPI_ISL_17461415	University of Sao Paulo	University of Sao Paulo	Esposito,D.L.A., Cunha,M.S., Maeda,A.Y., Bisordi,I., Rocco,I.M., Silva,F.G., Souza,R.P., Coimbra,T.L.M., Nogueira,J.S., Kisielius,J.J., Silveira,V.R., Santos,S.J.S., Oliveira,A.L.R., Suzuki,A. and Fonseca,B.A.L.
EPI_ISL_17461417, EPI_ISL_17461418, EPI_ISL_17461419, EPI_ISL_17461420, EPI_ISL_17461421	University of São Paulo	University of São Paulo	Lima,S.T.S., Souza,W.M., Cavalcante,J.W., da Silva Candido,D., Fumagalli,M.J., Carrera,J.P., Simoes Mello,L.M., de Carvalho Araujo,F.M., Cavalcante Ramalho,I.L., de Almeida Barreto,F.K., de Melo Braga,D.N., Simiao,A.R., Miranda da Silva,M.J., Oliveira,R.M.A.B., Lima,C.P.S., Sousa Lins,C., Barata,R.R., Melo,M.N.P., de Souza,M.P.C., Franco,L.M., Tavora,F.R.F., Queiroz Lemos,D.R., Alencar,C.H.M., Jesus,R., Souza Fonseca,V., Dutra,L.H., Abreu,A.L., Araujo,E.L.L., Ribas Freitas,A.R., Goncalves Vianez Junior,J.L.D.S., Pybus,O.G., Moraes Figueiredo,L.T., Faria,N.R., Teixeira Nunes,M.R., Goes Cavalcanti,L.P. and Miyajima,F.
EPI_ISL_17461422, EPI_ISL_17461423, EPI_ISL_17461424, EPI_ISL_17461425, EPI_ISL_17461426, EPI_ISL_17461427, EPI_ISL_17461428, EPI_ISL_17461429, EPI_ISL_17461430, EPI_ISL_17461431, EPI_ISL_17461432			
see above	University of Tartu	University of Tartu	LULLA,A., UTT,A. and VARJAK,M.
EPI_ISL_17461433, EPI_ISL_17461434	University of Texas Medical Branch	University of Texas Medical Branch	Faye,O., Diallo,M., Sow,A., Diallo,D., Faye,O., Diagne,C.T., Guerbois,M., Weidmann,M., Faye,A., Sadio,B., Ndiaye,O., Mondo,M., Hanley,K.A., Diop,O., Weaver,S.C. and Sall,A.A.
EPI_ISL_17461435, EPI_ISL_17461436	University of Texas Medical Branch	University of Texas Medical Branch	Langsjoen,R.M., Rubinstein,R.J., Kautz,T.F., Auguste,A.J., Erasmus,J.H., Kiaty-Figueroa,L., Gerhardt,R., Lin,D., Hari,K.L., Jain,R., Ruiz,N., Muruato,A.E., Silfa,J., Bido,F., Dacso,M. and Weaver,S.C.
EPI_ISL_17461437, EPI_ISL_17461438, EPI_ISL_17461439	University of Texas Medical Branch	University of Texas Medical Branch	Rodas,J.D., Kautz,T., Camacho,E., Paternina,L., Guzman,H., Diaz,F.J., Blanco,P., Tesh,R. and Weaver,S.C.
EPI_ISL_17461440	University of Texas Medical Branch	University of Texas Medical Branch	Chen,R., Puri,V., Fedorova,N., Lin,D., Hari,K.L., Jain,R., Rodas,J.D., Das,S.R., Shabman,R.S. and Weaver,S.C.
EPI_ISL_17461441	University of Texas Medical Branch	University of Texas Medical Branch	Rodas,J.D., Kautz,T., Camacho,E., Paternina,L., Guzman,H., Diaz,F.J., Blanco,P., Tesh,R. and Weaver,S.C.
EPI_ISL_17461442, EPI_ISL_17461443	University of Texas Medical Branch	University of Texas Medical Branch	Faye,O., Diallo,M., Sow,A., Diallo,D., Faye,O., Diagne,C.T., Guerbois,M., Weidmann,M., Faye,A., Sadio,B., Ndiaye,O., Mondo,M., Hanley,K.A., Diop,O., Weaver,S.C. and Sall,A.A.
EPI_ISL_17461444, EPI_ISL_17461445	University of Texas Medical Branch	University of Texas Medical Branch	Kautz,T.F., Diaz-Gonzalez,E.E., Erasmus,J.H., Malo-Garcia,I.R., Langsjoen,R.M., Patterson,E.I., Auguste,D.I., Forrester,N.L., Sanchez-Casas,R.M., Hernandez-Avila,M., Alpuche-Aranda,C.M., Weaver,S.C. and Fernandez-Salas,I.
EPI_ISL_17461446	University of Texas Medical Branch	University of Texas Medical Branch	Rodas,J.D., Kautz,T., Camacho,E., Paternina,L., Guzman,H., Diaz,F.J., Blanco,P., Tesh,R. and Weaver,S.C.
EPI_ISL_17461447, EPI_ISL_17461448, EPI_ISL_17461449, EPI_ISL_17461450, EPI_ISL_17461451, EPI_ISL_17461452	University of Texas Medical Branch	University of Texas Medical Branch	Kautz,T.F., Diaz-Gonzalez,E.E., Erasmus,J.H., Malo-Garcia,I.R., Langsjoen,R.M., Patterson,E.I., Auguste,D.I., Forrester,N.L., Sanchez-Casas,R.M., Hernandez-Avila,M., Alpuche-Aranda,C.M., Weaver,S.C. and Fernandez-Salas,I.
EPI_ISL_17461453	University of Texas Medical Branch	University of Texas Medical Branch	Diaz-Gonzalez,E.E., Kautz,T.F., Dorantes-Delgado,A., Malo-Garcia,I.R., Laguna-Aguilar,M., Langsjoen,R.M., Chen,R., Auguste,D.I., Sanchez-Casas,R.M., Danis-Lozano,R., Weaver,S.C. and Fernandez-Salas,I.
EPI_ISL_17461454, EPI_ISL_17461455	University of Texas Medical Branch	University of Texas Medical Branch	Kautz,T.F., Diaz-Gonzalez,E.E., Erasmus,J.H., Malo-Garcia,I.R., Langsjoen,R.M., Patterson,E.I., Auguste,D.I., Forrester,N.L., Sanchez-Casas,R.M., Hernandez-Avila,M., Alpuche-Aranda,C.M., Weaver,S.C. and Fernandez-Salas,I.
EPI_ISL_17461456, EPI_ISL_17461457, EPI_ISL_17461458, EPI_ISL_17461459	University of Texas Medical Branch	University of Texas Medical Branch	Diaz-Gonzalez,E.E., Kautz,T.F., Dorantes-Delgado,A., Malo-Garcia,I.R., Laguna-Aguilar,M., Langsjoen,R.M., Chen,R., Auguste,D.I., Sanchez-Casas,R.M., Danis-Lozano,R., Weaver,S.C. and Fernandez-Salas,I.
EPI_ISL_17461460	University of Texas Medical Branch	University of Texas Medical Branch	Rodas,J.D., Kautz,T., Camacho,E., Paternina,L., Guzman,H., Diaz,F.J., Blanco,P., Tesh,R. and Weaver,S.C.
EPI_ISL_17461461	University of Texas Medical Branch	University of Texas Medical Branch	Kautz,T.F., Diaz-Gonzalez,E.E., Erasmus,J.H., Malo-Garcia,I.R., Langsjoen,R.M., Patterson,E.I., Auguste,D.I., Forrester,N.L., Sanchez-Casas,R.M., Hernandez-Avila,M., Alpuche-Aranda,C.M., Weaver,S.C. and Fernandez-Salas,I.
EPI_ISL_17461462	University of Texas Medical Branch	University of Texas Medical Branch	Kautz,T.F., Forrester,N.L., Lin,D., Hari,K. and Weaver,S.C.
EPI_ISL_17461463	University of Texas Medical Branch	University of Texas Medical Branch	Kautz,T.F., Diaz-Gonzalez,E.E., Erasmus,J.H., Malo-Garcia,I.R., Langsjoen,R.M., Patterson,E.I., Auguste,D.I., Forrester,N.L., Sanchez-Casas,R.M., Hernandez-Avila,M., Alpuche-Aranda,C.M., Weaver,S.C. and Fernandez-Salas,I.
EPI_ISL_17461464	University of Texas Medical Branch	University of Texas Medical Branch	Powers,A.M., Brault,A.C., Tesh,R.B. and Weaver,S.C.
EPI_ISL_17461465, EPI_ISL_17461466	University of Texas Medical Branch	University of Texas Medical Branch	Rodas,J.D., Kautz,T., Camacho,E., Paternina,L., Guzman,H., Diaz,F.J., Blanco,P., Tesh,R. and Weaver,S.C.
EPI_ISL_17461467	University of Texas Medical Branch	University of Texas Medical Branch	Guerbois,M., Forrester,N.L., Rossi,S.L., Lin,D., Hari,K. and Weaver,S.C.
EPI_ISL_17461468	University of Texas Medical Branch	University of Texas Medical Branch	Rodas,J.D., Kautz,T., Camacho,E., Paternina,L., Guzman,H., Diaz,F.J., Blanco,P., Tesh,R. and Weaver,S.C.
EPI_ISL_17461469, EPI_ISL_17461470, EPI_ISL_17461471, EPI_ISL_17461472	University of Texas Medical Branch	University of Texas Medical Branch	Powers,A.M., Brault,A.C., Tesh,R.B. and Weaver,S.C.
EPI_ISL_17461473	University of Texas Medical Branch	University of Texas Medical Branch	Faye,O., Diallo,M., Sow,A., Diallo,D., Faye,O., Diagne,C.T., Guerbois,M., Weidmann,M., Faye,A., Sadio,B., Ndiaye,O., Mondo,M., Hanley,K.A., Diop,O., Weaver,S.C. and Sall,A.A.
EPI_ISL_17461474	University of Texas Medical Branch	University of Texas Medical Branch	Vanlandingham,D.L., Hong,C., Klingler,K., Tsetsarkin,K., McElroy,K.L., Powers,A.M., Lehane,M.J. and Higgs,S.
EPI_ISL_17461475, EPI_ISL_17461476, EPI_ISL_17461477	University of Texas Medical Branch	University of Texas Medical Branch	Faye,O., Diallo,M., Sow,A., Diallo,D., Faye,O., Diagne,C.T., Guerbois,M., Weidmann,M., Faye,A., Sadio,B., Ndiaye,O., Mondo,M., Hanley,K.A., Diop,O., Weaver,S.C. and Sall,A.A.
EPI_ISL_17461478	University of Texas Medical Branch	University of Texas Medical Branch	Powers,A.M., Brault,A.C., Tesh,R.B. and Weaver,S.C.
EPI_ISL_17461479, EPI_ISL_17461480, EPI_ISL_17461481, EPI_ISL_17461482	University of Texas Medical Branch	University of Texas Medical Branch	Faye,O., Diallo,M., Sow,A., Diallo,D., Faye,O., Diagne,C.T., Guerbois,M., Weidmann,M., Faye,A., Sadio,B., Ndiaye,O., Mondo,M., Hanley,K.A., Diop,O., Weaver,S.C. and Sall,A.A.
EPI_ISL_17461483	University of Texas Medical Branch	University of Texas Medical Branch	Powers,A.M., Brault,A.C., Tesh,R.B. and Weaver,S.C.
EPI_ISL_17461484	University of Texas Medical Branch	University of Texas Medical Branch	Tsetsarkin,K.A., Perng,G.C. and Weaver,S.C.
EPI_ISL_17461485	University of Texas Medical Branch	University of Texas Medical Branch	Powers,A.M., Brault,A.C., Tesh,R.B. and Weaver,S.C.
EPI_ISL_17461486	University of Texas Medical Branch	University of Texas Medical Branch	Tsetsarkin,K.A., Perng,G.C. and Weaver,S.C.
EPI_ISL_17461487, EPI_ISL_17461488	University of Texas Medical Branch	University of Texas Medical Branch	Faye,O., Diallo,M., Sow,A., Diallo,D., Faye,O., Diagne,C.T., Guerbois,M., Weidmann,M., Faye,A., Sadio,B., Ndiaye,O., Mondo,M., Hanley,K.A., Diop,O., Weaver,S.C. and Sall,A.A.
EPI_ISL_17461489	University of Texas Medical Branch	University of Texas Medical Branch	Rodas,J.D., Kautz,T., Camacho,E., Paternina,L., Guzman,H., Diaz,F.J., Blanco,P., Tesh,R. and Weaver,S.C.
EPI_ISL_17461490, EPI_ISL_17461491, EPI_ISL_17461492	University of Texas Medical Branch	University of Texas Medical Branch	Faye,O., Diallo,M., Sow,A., Diallo,D., Faye,O., Diagne,C.T., Guerbois,M., Weidmann,M., Faye,A., Sadio,B., Ndiaye,O., Mondo,M., Hanley,K.A., Diop,O., Weaver,S.C. and Sall,A.A.
EPI_ISL_17461493, EPI_ISL_17461494, EPI_ISL_17461495	University of Texas Medical Branch	University of Texas Medical Branch	Powers,A.M., Brault,A.C., Tesh,R.B. and Weaver,S.C.
EPI_ISL_17461496, EPI_ISL_17461497, EPI_ISL_17461498, EPI_ISL_17461499, EPI_ISL_17461500, EPI_ISL_17461501, EPI_ISL_17461502, EPI_ISL_17461503, EPI_ISL_17461504, EPI_ISL_17461505, EPI_ISL_17461506, EPI_ISL_17461507			
see above	University of Texas Medical Branch	University of Texas Medical Branch	Faye,O., Diallo,M., Sow,A., Diallo,D., Faye,O., Diagne,C.T., Guerbois,M., Weidmann,M., Faye,A., Sadio,B., Ndiaye,O., Mondo,M., Hanley,K.A., Diop,O., Weaver,S.C. and Sall,A.A.
EPI_ISL_17461508	University of Texas Medical Branch	University of Texas Medical Branch	Tsetsarkin,K.A., Perng,G.C. and Weaver,S.C.
EPI_ISL_17461509	University of Texas Medical Branch	University of Texas Medical Branch	Faye,O., Diallo,M., Sow,A., Diallo,D., Faye,O., Diagne,C.T., Guerbois,M., Weidmann,M., Faye,A., Sadio,B., Ndiaye,O., Mondo,M., Hanley,K.A., Diop,O., Weaver,S.C. and Sall,A.A.
EPI_ISL_17461510, EPI_ISL_17461511, EPI_ISL_17461512, EPI_ISL_17461513	University of Texas Medical Branch	University of Texas Medical Branch	Rodas,J.D., Kautz,T., Camacho,E., Paternina,L., Guzman,H., Diaz,F.J., Blanco,P., Tesh,R. and Weaver,S.C.
EPI_ISL_17461514	University of Texas Medical Branch	University of Texas Medical Branch	Langsjoen,R.M., Rubinstein,R.J., Kautz,T.F., Auguste,A.J., Erasmus,J.H., Kiaty-Figueroa,L., Gerhardt,R., Lin,D., Hari,K.L., Jain,R., Ruiz,N., Muruato,A.E., Silfa,J., Bido,F., Dacso,M. and Weaver,S.C.

EPI_ISL_17461515	University of Texas Medical Branch	University of Texas Medical Branch	Tsetsarkin,K.A., Perng,G.C. and Weaver,S.C.
EPI_ISL_17461516	University of Texas Medical Branch	University of Texas Medical Branch	Faye,O., Diallo,M., Sow,A., Diallo,D., Faye,O., Diagne,C.T., Guerbois,M., Weidmann,M., Faye,A., Sadio,B., Ndiaye,O., Mondo,M., Hanley,K.A., Diop,O., Weaver,S.C. and Sall,A.A.
EPI_ISL_17461517	University of Texas Medical Branch	University of Texas Medical Branch	Langsjoen,R.M., Rubinstein,R.J., Kautz,T.F., Auguste,A.J., Erasmus,J.H., Kiaty-Figueroa,L., Gerhardt,R., Lin,D., Hari,K.L., Jain,R., Ruiz,N., Muruato,A.E., Silfa,J., Bido,F., Dacso,M. and Weaver,S.C.
EPI_ISL_17461518	University of Texas Medical Branch	University of Texas Medical Branch	Kautz,T.F., Diaz-Gonzalez,E.E., Erasmus,J.H., Malo-Garcia,I.R., Langsjoen,R.M., Patterson,E.I., Auguste,D.I., Forrester,N.L., Sanchez-Casas,R.M., Hernandez-Avila,M., Alpuche-Aranda,C.M., Weaver,S.C. and Fernandez-Salas,I.
EPI_ISL_17461519	University of Texas Medical Branch	University of Texas Medical Branch	Kautz,T.F., Forrester,N.L., Hari,K., Lin,D. and Forrester,N.L.
EPI_ISL_17461520	University of Texas Medical Branch	University of Texas Medical Branch	Kautz,T.F., Forrester,N.L., Hari,K., Lin,D. and Weaver,S.C.
EPI_ISL_17461521	University of Texas Medical Branch	University of Texas Medical Branch	Auguste,A.J., Guerbois,M., Forrester,N.L., Rossi,S.L., Lin,D., Hari,K. and Weaver,S.C.
EPI_ISL_17461522	University of Texas Medical Branch	University of Texas Medical Branch	Guerbois,M., Forrester,N.L., Rossi,S.L., Lin,D., Hari,K. and Weaver,S.C.
EPI_ISL_17461523	University of Texas Medical Branch	University of Texas Medical Branch	Langsjoen,R.M., Rubinstein,R.J., Kautz,T.F., Auguste,A.J., Erasmus,J.H., Kiaty-Figueroa,L., Gerhardt,R., Lin,D., Hari,K.L., Jain,R., Ruiz,N., Muruato,A.E., Silfa,J., Bido,F., Dacso,M. and Weaver,S.C.
EPI_ISL_17461524	University of Texas Medical Branch	University of Texas Medical Branch	Chen,R., Puri,V., Fedorova,N., Lin,D., Hari,K.L., Jain,R., Rodas,J.D., Das,S.R., Shabman,R.S. and Weaver,S.C.
EPI_ISL_17461525	University of Texas Medical Branch	University of Texas Medical Branch	Powers,A.M., Brault,A.C., Tesh,R.B. and Weaver,S.C.
EPI_ISL_17461526, EPI_ISL_17461527	University of Texas Medical Branch	University of Texas Medical Branch	Langsjoen,R.M., Rubinstein,R.J., Kautz,T.F., Auguste,A.J., Erasmus,J.H., Kiaty-Figueroa,L., Gerhardt,R., Lin,D., Hari,K.L., Jain,R., Ruiz,N., Muruato,A.E., Silfa,J., Bido,F., Dacso,M. and Weaver,S.C.
EPI_ISL_17461528	University of Texas Medical Branch	University of Texas Medical Branch	Guerbois,M., Forrester,N.L., Hari,K., Lin,D. and Weaver,S.C.
EPI_ISL_17461529, EPI_ISL_17461530, EPI_ISL_17461531	University of Texas Medical Branch	University of Texas Medical Branch	Powers,A.M., Brault,A.C., Tesh,R.B. and Weaver,S.C.
EPI_ISL_17461532	University of Texas Medical Branch	University of Texas Medical Branch	Faye,O., Diallo,M., Sow,A., Diallo,D., Faye,O., Diagne,C.T., Guerbois,M., Weidmann,M., Faye,A., Sadio,B., Ndiaye,O., Mondo,M., Hanley,K.A., Diop,O., Weaver,S.C. and Sall,A.A.
EPI_ISL_17461533, EPI_ISL_17461534	University of Texas Medical Branch	University of Texas Medical Branch	Powers,A.M., Brault,A.C., Tesh,R.B. and Weaver,S.C.
EPI_ISL_17461535	University of Texas Medical Branch	University of Texas Medical Branch	Faye,O., Diallo,M., Sow,A., Diallo,D., Faye,O., Diagne,C.T., Guerbois,M., Weidmann,M., Faye,A., Sadio,B., Ndiaye,O., Mondo,M., Hanley,K.A., Diop,O., Weaver,S.C. and Sall,A.A.
EPI_ISL_17461536	University of Texas Medical Branch	University of Texas Medical Branch	Powers,A.M., Brault,A.C., Tesh,R.B. and Weaver,S.C.
EPI_ISL_17461537	University of Texas Medical Branch	University of Texas Medical Branch	Langsjoen,R.M., Rubinstein,R.J., Kautz,T.F., Auguste,A.J., Erasmus,J.H., Kiaty-Figueroa,L., Gerhardt,R., Lin,D., Hari,K.L., Jain,R., Ruiz,N., Muruato,A.E., Silfa,J., Bido,F., Dacso,M. and Weaver,S.C.
EPI_ISL_17461538	University of Texas Medical Branch	University of Texas Medical Branch	Faye,O., Diallo,M., Sow,A., Diallo,D., Faye,O., Diagne,C.T., Guerbois,M., Weidmann,M., Faye,A., Sadio,B., Ndiaye,O., Mondo,M., Hanley,K.A., Diop,O., Weaver,S.C. and Sall,A.A.
EPI_ISL_17461539, EPI_ISL_17461540	University of Texas Medical Branch	University of Texas Medical Branch	Langsjoen,R.M., Rubinstein,R.J., Kautz,T.F., Auguste,A.J., Erasmus,J.H., Kiaty-Figueroa,L., Gerhardt,R., Lin,D., Hari,K.L., Jain,R., Ruiz,N., Muruato,A.E., Silfa,J., Bido,F., Dacso,M. and Weaver,S.C.
EPI_ISL_17461541, EPI_ISL_17461542	University of Texas Medical Branch	University of Texas Medical Branch	Tsetsarkin,K.A., Perng,G.C. and Weaver,S.C.
EPI_ISL_17461543, EPI_ISL_17461544, EPI_ISL_17461545	University of Western Australia	University of Western Australia	Harapan,H., Michie,A., Ernst,T., Panta,K., Mudatsir,M., Yohan,B., Haryanto,S., McCarthy,S., Sasmono,R.T. and Imrie,A.
EPI_ISL_17461546, EPI_ISL_17461547, EPI_ISL_17461548, EPI_ISL_17461549	Université de la Méditerranée	Université de la Méditerranée	Parola,P., de Lamballerie,X., Jourdan,J., Rovery,C., Vaillant,V., Minodier,P., Brouqui,P., Flahault,A., Raoult,D. and Charrel,R.N.
EPI_ISL_17461550, EPI_ISL_17461551, EPI_ISL_17461552, EPI_ISL_17461553, EPI_ISL_17461554	VLP Therapeutics	VLP Therapeutics	Ueno,R. and Akahata,W.
EPI_ISL_17461555	VLP Therapeutics	VLP Therapeutics	Akahata,W. and Ueno,R.
EPI_ISL_17461556, EPI_ISL_17461557, EPI_ISL_17461558	VLP Therapeutics	VLP Therapeutics	Ueno,R. and Akahata,W.
EPI_ISL_17461559	VLP Therapeutics	VLP Therapeutics	Akahata,W. and Ueno,R.
EPI_ISL_17461560, EPI_ISL_17461561, EPI_ISL_17461562	VLP Therapeutics	VLP Therapeutics	Ueno,R. and Akahata,W.
EPI_ISL_17461563, EPI_ISL_17461564, EPI_ISL_17461565, EPI_ISL_17461566	VLP Therapeutics	VLP Therapeutics	UENO,R. and AKAHATA,W.
EPI_ISL_17461567, EPI_ISL_17461568, EPI_ISL_17461569, EPI_ISL_17461570, EPI_ISL_17461571, EPI_ISL_17461572	VLP Therapeutics	VLP Therapeutics	Ueno,R. and Akahata,W.
EPI_ISL_17461573	Valneva SE	Valneva SE	Fritzer,A., Meinke,A., Lundberg,U., Nebenfuhr,M., Heindl-Wruss,J., Schlegl,R. and Leon,A.
EPI_ISL_17461574	Valneva SE	Valneva SE	FRITZER,A., MEINKE,A., LUNDBERG,U., NEBENFUHR,M., HEINDL-WRUSS,J., SCHLEGL,R. and LEON,A.
EPI_ISL_17461575	Valneva SE	Valneva SE	REINISCH,C., SCHLEGL,R. and HEIDL-WRUSS,J.
EPI_ISL_17461576	Valneva SE	Valneva SE	Wressnigg,N. and Hochreiter,R.
EPI_ISL_17461577	Valneva SE	Valneva SE	BARBERO,C.J., NEBENFUHR,M., SCHLEGL,R., WEBER,M. and WRUSS,J.
EPI_ISL_17461578	Valneva SE	Valneva SE	Reinisch,C., Schlegl,R. and Heindl-Wruss,J.
EPI_ISL_17461579, EPI_ISL_17461580	Valneva SE	Valneva SE	SCHLEGL,R., NEBENFUHR,M., WEBER,M. and WRUSS,J.
EPI_ISL_17461581, EPI_ISL_17461582	Valneva SE	Valneva SE	BARBERO,C.J., NEBENFUHR,M., SCHLEGL,R., WEBER,M. and WRUSS,J.
EPI_ISL_17461583	Valneva SE	Valneva SE	WRESSNIGG,N. and HOCHREITER,R.
EPI_ISL_17461584	Valneva SE	Valneva SE	BARBERO,C.J., NEBENFUHR,M., SCHLEGL,R., WEBER,M. and WRUSS,J.
EPI_ISL_17461585, EPI_ISL_17461586, EPI_ISL_17461587, EPI_ISL_17461588	Vanderbilt University	Vanderbilt University	CROWE,J.E., SMITH,S.A., DERMODY,T. and SILVA,L.
EPI_ISL_17461589	Vanderbilt University	Vanderbilt University	Crowe,J.E., Smith,S.A., Dermody,T. and Silva,L.
EPI_ISL_17461590, EPI_ISL_17461591, EPI_ISL_17461592, EPI_ISL_17461593	Vanderbilt University	Vanderbilt University	CROWE,J.E., SMITH,S.A., DERMODY,T. and SILVA,L.
EPI_ISL_17461594, EPI_ISL_17461595, EPI_ISL_17461596, EPI_ISL_17461597, EPI_ISL_17461598, EPI_ISL_17461599, EPI_ISL_17461600, EPI_ISL_17461601, EPI_ISL_17461602, EPI_ISL_17461603	Vanderbilt University	Vanderbilt University	Crowe,J.E., Smith,S.A., Dermody,T. and Silva,L.
EPI_ISL_17461604	Vanderbilt University	Vanderbilt University	CROWE,J.E., SMITH,S.A., DERMODY,T. and SILVA,L.
EPI_ISL_17461605, EPI_ISL_17461606	Vanderbilt University	Vanderbilt University	Crowe,J.E., Smith,S.A., Dermody,T. and Silva,L.
EPI_ISL_17461607	Vanderbilt University	Vanderbilt University	CROWE,J.E., SMITH,S.A., DERMODY,T. and SILVA,L.
EPI_ISL_17461608, EPI_ISL_17461609, EPI_ISL_17461610, EPI_ISL_17461611, EPI_ISL_17461612, EPI_ISL_17461613	Vanderbilt University	Vanderbilt University	Crowe,J.E., Smith,S.A., Dermody,T. and Silva,L.
EPI_ISL_17461614	Vanderbilt University	Vanderbilt University	CROWE,J.E., SMITH,S.A., DERMODY,T. and SILVA,L.
EPI_ISL_17461615, EPI_ISL_17461616, EPI_ISL_17461617, EPI_ISL_17461618	Vanderbilt University	Vanderbilt University	Crowe,J.E., Smith,S.A., Dermody,T. and Silva,L.
EPI_ISL_17461619	Vanderbilt University	Vanderbilt University	CROWE,J.E., SMITH,S.A., DERMODY,T. and SILVA,L.
EPI_ISL_17461620	Vanderbilt University	Vanderbilt University	Crowe,J.E., Smith,S.A., Dermody,T. and Silva,L.
EPI_ISL_17461621, EPI_ISL_17461622	Vector Control Research Centre	Vector Control Research Centre	Pradeep Kumar,N., Jambulingam,P. and Krishnamoorthy,N.
EPI_ISL_17461623	Vector Control Research Centre	Vector Control Research Centre	Kumar,N.P., Sabesan,S., Krishnamoorthy,K. and Jambulingam,P.
EPI_ISL_17461624	Vector Control Research Centre	Vector Control Research Centre	Pradeep Kumar,N., Jambulingam,P. and Krishnamoorthy,N.
EPI_ISL_17461625	Vector Control Research Centre	Vector Control Research Centre	Kumar,N.P., Sabesan,S., Krishnamoorthy,K. and Jambulingam,P.
EPI_ISL_17461626, EPI_ISL_17461627	Vector Control Research Centre	Vector Control Research Centre	Pradeep Kumar,N., Jambulingam,P. and Krishnamoorthy,N.
EPI_ISL_17461628	Vector Control Research Centre	Vector Control Research Centre	Kumar,N.P., Sabesan,S., Krishnamoorthy,K. and Jambulingam,P.
EPI_ISL_17461629	Vector Control Research Centre	Vector Control Research Centre	Pradeep Kumar,N., Jambulingam,P. and Krishnamoorthy,N.
EPI_ISL_17461630	Vector Control Research Centre	Vector Control Research Centre	Kumar,N.P., Sabesan,S., Krishnamoorthy,K. and Jambulingam,P.
EPI_ISL_17461631, EPI_ISL_17461632	Vector Control Research Centre	Vector Control Research Centre	Pradeep Kumar,N., Jambulingam,P. and Krishnamoorthy,N.
EPI_ISL_17461633	Vector Control Research Centre	Vector Control Research Centre	Kumar,N.P., Sabesan,S., Krishnamoorthy,K. and Jambulingam,P.
EPI_ISL_17461634	Vector Control Research Centre	Vector Control Research Centre	Pradeep Kumar,N., Jambulingam,P. and Krishnamoorthy,N.
EPI_ISL_17461635, EPI_ISL_17461636, EPI_ISL_17461637	Vector Control Research Centre, Indian Council of Medical Research	Vector Control Research Centre, Indian Council of Medical Research	Pradeep Kumar,N., Jambulingam,P., Yuvaraj,J. and Das,P.K.
EPI_ISL_17461638	Vector Control Research Centre, Indian Council of Medical Research	Vector Control Research Centre, Indian Council of Medical Research	Kumar,N.P., Joseph,R., Kamaraj,T. and Jambulingam,P.
EPI_ISL_17461639	Vector Control Research Centre, Indian Council of Medical Research	Vector Control Research Centre, Indian Council of Medical Research	Pradeep Kumar,N., Jambulingam,P., Yuvaraj,J. and Das,P.K.

EPI_ISL_17461640, EPI_ISL_17461641, EPI_ISL_17461642, EPI_ISL_17461643	Vector Control Research Centre, Indian Council of Medical Research	Vector Control Research Centre, Indian Council of Medical Research	Kumar,N.P., Joseph,R., Kamaraj,T. and Jambulingam,P.
EPI_ISL_17461644	Vector Control Research Centre, Indian Council of Medical Research	Vector Control Research Centre, Indian Council of Medical Research	Pradeep Kumar,N., Jambulingam,P., Yuvaraj,J. and Das,P.K.
EPI_ISL_17461645	Vector Control Research Centre, Indian Council of Medical Research	Vector Control Research Centre, Indian Council of Medical Research	Pradeep Kumar,N., Yuvaraj,J., Jambulingam,P. and Das,P.K.
EPI_ISL_17461646, EPI_ISL_17461647	Vector Control Research Centre, Indian Council of Medical Research	Vector Control Research Centre, Indian Council of Medical Research	Kumar,N.P., Joseph,R., Kamaraj,T. and Jambulingam,P.
EPI_ISL_17461648	Vector Control Research Centre, Indian Council of Medical Research	Vector Control Research Centre, Indian Council of Medical Research	Pradeep Kumar,N., Jambulingam,P., Yuvaraj,J. and Das,P.K.
EPI_ISL_17461649, EPI_ISL_17461650, EPI_ISL_17461651	Vector Control Research Centre, Indian Council of Medical Research	Vector Control Research Centre, Indian Council of Medical Research	Kumar,N.P., Joseph,R., Kamaraj,T. and Jambulingam,P.
EPI_ISL_17461652, EPI_ISL_17461653, EPI_ISL_17461654, EPI_ISL_17461655, EPI_ISL_17461656	Vector Control Research Centre, Indian Council of Medical Research	Vector Control Research Centre, Indian Council of Medical Research	Pradeep Kumar,N., Jambulingam,P., Yuvaraj,J. and Das,P.K.
EPI_ISL_17461657	Vector Control Research Centre, Indian Council of Medical Research	Vector Control Research Centre, Indian Council of Medical Research	Kumar,N.P., Joseph,R., Kamaraj,T. and Jambulingam,P.
EPI_ISL_17461658	Vector Control Research Centre, Indian Council of Medical Research	Vector Control Research Centre, Indian Council of Medical Research	Pradeep Kumar,N., Yuvaraj,J., Jambulingam,P. and Das,P.K.
EPI_ISL_17461659	Vector Control Research Centre, Indian Council of Medical Research	Vector Control Research Centre, Indian Council of Medical Research	Kumar,N.P., Joseph,R., Kamaraj,T. and Jambulingam,P.
EPI_ISL_17461660, EPI_ISL_17461661	Vector Control Research Centre, Indian Council of Medical Research	Vector Control Research Centre, Indian Council of Medical Research	Pradeep Kumar,N., Yuvaraj,J., Jambulingam,P. and Das,P.K.
EPI_ISL_17461662, EPI_ISL_17461663	Vector Control Research Centre, Indian Council of Medical Research	Vector Control Research Centre, Indian Council of Medical Research	Kumar,N.P., Joseph,R., Kamaraj,T. and Jambulingam,P.
EPI_ISL_17461664	Vector Control Research Centre, Indian Council of Medical Research	Vector Control Research Centre, Indian Council of Medical Research	Pradeep Kumar,N., Jambulingam,P., Yuvaraj,J. and Das,P.K.
EPI_ISL_17461665, EPI_ISL_17461666, EPI_ISL_17461667, EPI_ISL_17461668, EPI_ISL_17461669	Vector Control Research Centre, Indian Council of Medical Research	Vector Control Research Centre, Indian Council of Medical Research	Kumar,N.P., Joseph,R., Kamaraj,T. and Jambulingam,P.
EPI_ISL_17461670	Vector Control Research Centre, Indian Council of Medical Research	Vector Control Research Centre, Indian Council of Medical Research	Pradeep Kumar,N., Jambulingam,P., Yuvaraj,J. and Das,P.K.
EPI_ISL_17461671	Vector Control Research Centre, Indian Council of Medical Research	Vector Control Research Centre, Indian Council of Medical Research	Sankari,T., Hoti,S.L., Yuvaraj,J. and Das,P.K.
EPI_ISL_17461672, EPI_ISL_17461673, EPI_ISL_17461674	Vector Control Research Centre, Indian Council of Medical Research	Vector Control Research Centre, Indian Council of Medical Research	Sankari,T., Hoti,S.L., Sarangapani, Govindaraj,V. and Das,P.K.
EPI_ISL_17461675, EPI_ISL_17461676	Vector Control Research Centre, Indian Council of Medical Research	Vector Control Research Centre, Indian Council of Medical Research	Pradeep Kumar,N., Jambulingam,P., Yuvaraj,J. and Das,P.K.
EPI_ISL_17461677	Vector Control Research Centre, Indian Council of Medical Research	Vector Control Research Centre, Indian Council of Medical Research	Kumar,N.P., Joseph,R., Kamaraj,T. and Jambulingam,P.
EPI_ISL_17461678	Vellore Institute of Technology	Vellore Institute of Technology	Dhanasekaran,D. and Deepika,L.
EPI_ISL_17461679	Victorian Infectious Diseases Reference Laboratory	Victorian Infectious Diseases Reference Laboratory	Druce,J.D., Johnson,D.F., Tran,T., Richards,M.J. and Birch,C.J.
EPI_ISL_17461680, EPI_ISL_17461681, EPI_ISL_17461682, EPI_ISL_17461683, EPI_ISL_17461684	Armed Forces Institute of Pathology	Armed Forces Institute of Pathology	Hussain,T., Niazi,S.K., Sawal,H.A., Ghani,E. and Noor,M.
EPI_ISL_17461685, EPI_ISL_17461686, EPI_ISL_17461687, EPI_ISL_17461688, EPI_ISL_17461689, EPI_ISL_17461690, EPI_ISL_17461691, EPI_ISL_17461692	Walailak University	Walailak University	Phumee,A., Intayot,P., Sor-Suwan,S., Jittmittraphap,A. and Siriyasatien,P.
EPI_ISL_17461693	Walter Reed Army Institute of Research	Walter Reed Army Institute of Research	Maljkovic Berry,I., Eyase,F., Pollett,S., Limbaso Konongoi,S., Figueroa,K., Ofula,V., Koka,H., Koskei,E., Nyunja,A., Mancuso,J.D., Jarman,R.G. and Sang,R.
EPI_ISL_17461694	Walter Reed Army Institute of Research	Walter Reed Army Institute of Research	Berry,I.M.
EPI_ISL_17461695	Walter Reed Army Institute of Research	Walter Reed Army Institute of Research	Maljkovic Berry,I., Eyase,F., Pollett,S., Limbaso Konongoi,S., Figueroa,K., Ofula,V., Koka,H., Koskei,E., Nyunja,A., Mancuso,J.D., Jarman,R.G. and Sang,R.
EPI_ISL_17461696	Walter Reed Army Institute of Research	Walter Reed Army Institute of Research	Berry,I.M.
EPI_ISL_17461697	Walter Reed Army Institute of Research	Walter Reed Army Institute of Research	Maljkovic Berry,I., Eyase,F., Pollett,S., Limbaso Konongoi,S., Figueroa,K., Ofula,V., Koka,H., Koskei,E., Nyunja,A., Mancuso,J.D., Jarman,R.G. and Sang,R.
EPI_ISL_17461698, EPI_ISL_17461699	Walter Reed Army Institute of Research	Walter Reed Army Institute of Research	Berry,I.M.
EPI_ISL_17461700	Walter Reed Army Institute of Research	Walter Reed Army Institute of Research	Maljkovic Berry,I., Eyase,F., Pollett,S., Limbaso Konongoi,S., Figueroa,K., Ofula,V., Koka,H., Koskei,E., Nyunja,A., Mancuso,J.D., Jarman,R.G. and Sang,R.
EPI_ISL_17461701, EPI_ISL_17461702	Walter Reed Army Institute of Research	Walter Reed Army Institute of Research	Berry,I.M.
EPI_ISL_17461703	Walter Reed Army Institute of Research	Walter Reed Army Institute of Research	Maljkovic Berry,I., Eyase,F., Pollett,S., Limbaso Konongoi,S., Figueroa,K., Ofula,V., Koka,H., Koskei,E., Nyunja,A., Mancuso,J.D., Jarman,R.G. and Sang,R.
EPI_ISL_17461704, EPI_ISL_17461705, EPI_ISL_17461706, EPI_ISL_17461707, EPI_ISL_17461708	Walter Reed Army Institute of Research	Walter Reed Army Institute of Research	Berry,I.M.
EPI_ISL_17461709, EPI_ISL_17461710, EPI_ISL_17461711	Walter Reed Army Institute of Research	Walter Reed Army Institute of Research	Maljkovic Berry,I., Eyase,F., Pollett,S., Limbaso Konongoi,S., Figueroa,K., Ofula,V., Koka,H., Koskei,E., Nyunja,A., Mancuso,J.D., Jarman,R.G. and Sang,R.
EPI_ISL_17461712	Walter Reed Army Institute of Research	Walter Reed Army Institute of Research	Berry,I.M.
EPI_ISL_17461713	Walter Reed Army Institute of Research	Walter Reed Army Institute of Research	Maljkovic Berry,I., Eyase,F., Pollett,S., Limbaso Konongoi,S., Figueroa,K., Ofula,V., Koka,H., Koskei,E., Nyunja,A., Mancuso,J.D., Jarman,R.G. and Sang,R.
EPI_ISL_17461714	Walter Reed Army Institute of Research	Walter Reed Army Institute of Research	Berry,I.M.
EPI_ISL_17461715, EPI_ISL_17461716, EPI_ISL_17461717, EPI_ISL_17461718	Walter Reed Army Institute of Research	Walter Reed Army Institute of Research	Maljkovic Berry,I., Eyase,F., Pollett,S., Limbaso Konongoi,S., Figueroa,K., Ofula,V., Koka,H., Koskei,E., Nyunja,A., Mancuso,J.D., Jarman,R.G. and Sang,R.
EPI_ISL_17461719	Walter Reed Army Institute of Research	Walter Reed Army Institute of Research	Berry,I.M.
EPI_ISL_17461720	Walter Reed Army Institute of Research	Walter Reed Army Institute of Research	Maljkovic Berry,I., Eyase,F., Pollett,S., Limbaso Konongoi,S., Figueroa,K., Ofula,V., Koka,H., Koskei,E., Nyunja,A., Mancuso,J.D., Jarman,R.G. and Sang,R.
EPI_ISL_17461721, EPI_ISL_17461722, EPI_ISL_17461723, EPI_ISL_17461724, EPI_ISL_17461725, EPI_ISL_17461726, EPI_ISL_17461727, EPI_ISL_17461728, EPI_ISL_17461729, EPI_ISL_17461730, EPI_ISL_17461731			
see above	Walter Reed Army Institute of Research	Walter Reed Army Institute of Research	Berry,I.M.
EPI_ISL_17461732	Walter Reed Army Institute of Research	Walter Reed Army Institute of Research	Maljkovic Berry,I., Eyase,F., Pollett,S., Limbaso Konongoi,S., Figueroa,K., Ofula,V., Koka,H., Koskei,E., Nyunja,A., Mancuso,J.D., Jarman,R.G. and Sang,R.
EPI_ISL_17461733, EPI_ISL_17461734, EPI_ISL_17461735, EPI_ISL_17461736, EPI_ISL_17461737, EPI_ISL_17461738, EPI_ISL_17461739	Wuhan Institute of Virology, Chinese Academy of Sciences	Wuhan Institute of Virology, Chinese Academy of Sciences	Shi,J.M., Shen,S., Deng,F., Jehan,S. and Jamil,N.
EPI_ISL_17461740	Zhejiang Provincial Center for Disease Control and Prevention	Zhejiang Provincial Center for Disease Control and Prevention	Sun,Y., Yan,J., Mao,H., Zhang,L., Lyu,Q., Wu,Z., Zheng,W., Feng,C. and Zhang,Y.
EPI_ISL_17461741	Zhejiang Provincial Center for Disease Control and Prevention	Zhejiang Provincial Center for Disease Control and Prevention	Pan,J.
EPI_ISL_17461742	Zhejiang Provincial Center for Disease Control and Prevention	Zhejiang Provincial Center for Disease Control and Prevention	Pan,J., Zhang,Y. and Yan,J.
EPI_ISL_17461743, EPI_ISL_17461744	Zhejiang Provincial Center for Disease Control and Prevention	Zhejiang Provincial Center for Disease Control and Prevention	Pan,J.
EPI_ISL_17461745, EPI_ISL_17461746, EPI_ISL_17461747, EPI_ISL_17461748, EPI_ISL_17461749, EPI_ISL_17461750, EPI_ISL_17461751, EPI_ISL_17461752, EPI_ISL_17461753, EPI_ISL_17461754, EPI_ISL_17461755, EPI_ISL_17461756, EPI_ISL_17461757, EPI_ISL_17461758			
see above	bioMérieux	bioMérieux	Telles,J.N.
EPI_ISL_17461759	unknown	unknown	Yajima,M., Yamamoto,N., Nikaido,M., Gemma,N. and Takahashi,M.
EPI_ISL_17461760, EPI_ISL_17461761, EPI_ISL_17461762, EPI_ISL_17461763, EPI_ISL_17461764, EPI_ISL_17461765, EPI_ISL_17461766, EPI_ISL_17461767	Virology, Pedro Kouri Institute of Tropical Medicine	Virology, Pedro Kouri Institute of Tropical Medicine	Diaz Gutierrez,G., Rodriguez-Roche,R., Alvarez Vera,M., Mesa Castillo,E., Santana Acosta,E. and Guzman Tirado,M.
EPI_ISL_17462237, EPI_ISL_17462238, EPI_ISL_17462239, EPI_ISL_17462240, EPI_ISL_17462241, EPI_ISL_17462242, EPI_ISL_17462243, EPI_ISL_17462244, EPI_ISL_17462245, EPI_ISL_17462246, EPI_ISL_17462247, EPI_ISL_17462248, EPI_ISL_17462249			
see above	Instituto de Microbiologia	Instituto de Microbiologia	Marquez,S., Lee,G., Zuniga,J., Andrade,P., Trueba,G., Eisenberg,J. and Coloma,J.

EPI_ISL_17462250	Department of Virolgy, Institute of Tropical Medicine, Nagasaki University	Department of Virolgy, Institute of Tropical Medicine, Nagasaki University	Khan,A.H., Morita,K., Parquet Md Mdel,C., Hasebe,F., Mathenge,E.G. and Igarashi,A.
EPI_ISL_17673039, EPI_ISL_17673040, EPI_ISL_17673041, EPI_ISL_17673042, EPI_ISL_17673043, EPI_ISL_17673044	King Abdulaziz University	King Abdulaziz University	Alguridi,H.I., Alzahrani,F., Altayb,H.N., Almalki,S., Zaki,E., Algarni,S., Assiri,A. and Memish,Z.A.
EPI_ISL_17673045, EPI_ISL_17673046, EPI_ISL_17673047, EPI_ISL_17673048, EPI_ISL_17673049, EPI_ISL_17673050, EPI_ISL_17673051, EPI_ISL_17673052, EPI_ISL_17673053, EPI_ISL_17673054, EPI_ISL_17673055, EPI_ISL_17673056, EPI_ISL_17673057, EPI_ISL_17673058, EPI_ISL_17673059, EPI_ISL_17673060, EPI_ISL_17673061, EPI_ISL_17673062, EPI_ISL_17673063, EPI_ISL_17673064, EPI_ISL_17673065, EPI_ISL_17673066, EPI_ISL_17673067, EPI_ISL_17673068, EPI_ISL_17673069, EPI_ISL_17673070, EPI_ISL_17673071, EPI_ISL_17673072, EPI_ISL_17673073, EPI_ISL_17673074, EPI_ISL_17673075, EPI_ISL_17673076, EPI_ISL_17673077, EPI_ISL_17673078, EPI_ISL_17673079, EPI_ISL_17673080, EPI_ISL_17673081, EPI_ISL_17673082, EPI_ISL_17673083, EPI_ISL_17673084, EPI_ISL_17673085, EPI_ISL_17673086, EPI_ISL_17673087, EPI_ISL_17673088, EPI_ISL_17673089, EPI_ISL_17673090, EPI_ISL_17673091, EPI_ISL_17673092, EPI_ISL_17673093, EPI_ISL_17673094, EPI_ISL_17673095, EPI_ISL_17673096, EPI_ISL_17673097, EPI_ISL_17673098, EPI_ISL_17673099, EPI_ISL_17673100, EPI_ISL_17673101, EPI_ISL_17673102, EPI_ISL_17673103, EPI_ISL_17673104, EPI_ISL_17673105			
see above	Department of Pathology, University of Texas Medical Branch	Department of Pathology, University of Texas Medical Branch	de Souza,W.M., de Lima,S.T.S., Simoes Mello,L.M., Candido,D.S., Buss,L., Whittaker,C., Claro,I.M., Chandradeva,N., Granja,F., de Jesus,R., Lemos,P.S., Toledo-Teixeira,D.A., Barbosa,P.P., Firmino,A.C.L., Amorim,M.R., Duarte,L.M.F., Pessoa,I.B. Jr., Forato,J., Vasconcelos,I.L., Maximo,A.C.B.M., Araujo,E.L.L., Perdigao Mello,L., Sabino,E.C., Proenca-Modena,J.L., Faria,N.R. and Weaver,S.C.
EPI_ISL_17673106, EPI_ISL_17673107, EPI_ISL_17673108	Ricardo Gutierrez Children's Hospital	Ricardo Gutierrez Children's Hospital	Rojo,G.L., Goya,S., Viegas,M., Nabaes Jodar,M.S., Lopez,M.C.A., Mistchenko,A.S. and Valinoto,L.E.
EPI_ISL_17673109, EPI_ISL_17673110, EPI_ISL_17673111, EPI_ISL_17673112, EPI_ISL_17673113, EPI_ISL_17673114, EPI_ISL_17673115, EPI_ISL_17673116, EPI_ISL_17673117, EPI_ISL_17673118, EPI_ISL_17673119, EPI_ISL_17673120, EPI_ISL_17673121, EPI_ISL_17673122, EPI_ISL_17673123, EPI_ISL_17673124, EPI_ISL_17673125, EPI_ISL_17673126, EPI_ISL_17673127, EPI_ISL_17673128, EPI_ISL_17673129, EPI_ISL_17673130, EPI_ISL_17673131, EPI_ISL_17673132, EPI_ISL_17673133, EPI_ISL_17673134, EPI_ISL_17673135, EPI_ISL_17673136, EPI_ISL_17673137, EPI_ISL_17673138, EPI_ISL_17673139, EPI_ISL_17673140, EPI_ISL_17673141, EPI_ISL_17673142			
see above	Federal University of Rio de Janeiro	Federal University of Rio de Janeiro	Moreira,F.R.R., Menezes,M.T., Salgado-Benvindo,C., Paula,H.H.S., Martins,A.F., Chagas,R.R., Brasil,R.D.V., Candido,D.S., Herlinger,A.L., Paula,M.C., Voloch,C.M., Tanuri,A., Iani,F., Cardozo,S.V. and Faria,N.R.
EPI_ISL_17673143	Université de la Méditerranée	Université de la Méditerranée	Moyen,N., Thiberville,S.D., Pastorino,B., Nougairede,A., Thirion,L., Mombouli,J.V., Dimi,Y., Leparc-Goffart,I., Capobianchi,M.R., Lepfoundzou,A.D. and de Lamballerie,X.
EPI_ISL_17680329, EPI_ISL_17680330, EPI_ISL_17680331, EPI_ISL_17680332, EPI_ISL_17680333, EPI_ISL_17680334	Instituo Adolfo Lutz Central	Instituto Adolfo Lutz - Strategic Laboratory	Claudio Tavares Sacchi, Karoline Rodrigues Campos, Marlon Benedito Nascimento Santos, Juliana Silva Nogueira
EPI_ISL_17680335, EPI_ISL_17680336, EPI_ISL_17680337, EPI_ISL_17680338, EPI_ISL_17680339, EPI_ISL_17680340, EPI_ISL_17680341, EPI_ISL_17680342, EPI_ISL_17680343	LACEN Mato Grosso do Sul	Instituto Adolfo Lutz - Strategic Laboratory	Claudio Tavares Sacchi, Karoline Rodrigues Campos, Marlon Benedito Nascimento Santos, Juliana Silva Nogueira
EPI_ISL_17680344, EPI_ISL_17680345, EPI_ISL_17680346, EPI_ISL_17680347, EPI_ISL_17680348, EPI_ISL_17680349, EPI_ISL_17680350, EPI_ISL_17680351, EPI_ISL_17680352, EPI_ISL_17680353, EPI_ISL_17680354, EPI_ISL_17680355, EPI_ISL_17680356, EPI_ISL_17680357, EPI_ISL_17680358, EPI_ISL_17680359, EPI_ISL_17680360, EPI_ISL_17680361, EPI_ISL_17680362, EPI_ISL_17680363, EPI_ISL_17680364, EPI_ISL_17680365, EPI_ISL_17680366, EPI_ISL_17680367, EPI_ISL_17680368, EPI_ISL_17680369, EPI_ISL_17680371, EPI_ISL_17680372			
see above	LACEN do Parana	Instituto Adolfo Lutz - Strategic Laboratory	Claudio Tavares Sacchi, Karoline Rodrigues Campos, Marlon Benedito Nascimento Santos, Juliana Silva Nogueira
EPI_ISL_17680374, EPI_ISL_17680375, EPI_ISL_17680376, EPI_ISL_17680377, EPI_ISL_17680378, EPI_ISL_17680379	Instituo Adolfo Lutz Central	Instituto Adolfo Lutz - Strategic Laboratory	Claudio Tavares Sacchi, Karoline Rodrigues Campos, Marlon Benedito Nascimento Santos, Juliana Silva Nogueira
EPI_ISL_17680380, EPI_ISL_17680381	LACEN Mato Grosso do Sul	Instituto Adolfo Lutz - Strategic Laboratory	Claudio Tavares Sacchi, Karoline Rodrigues Campos, Marlon Benedito Nascimento Santos, Juliana Silva Nogueira
EPI_ISL_17700289, EPI_ISL_17700290, EPI_ISL_17700291, EPI_ISL_17700292, EPI_ISL_17700293, EPI_ISL_17700294, EPI_ISL_17700295, EPI_ISL_17700296, EPI_ISL_17700297, EPI_ISL_17700298, EPI_ISL_17700299, EPI_ISL_17700300, EPI_ISL_17700301, EPI_ISL_17700302			
see above	Instituto Adolfo Lutz - Strategic Laboratory	Instituto Adolfo Lutz - Strategic Laboratory	Claudio Tavares Sacchi, Karoline Rodrigues Campos, Marlon Benedito Nascimento Santos, Juliana Silva Nogueira
EPI_ISL_17700303, EPI_ISL_17700304, EPI_ISL_17700305	LACEN de Sergipe	Instituto Adolfo Lutz - Strategic Laboratory	Claudio Tavares Sacchi, Karoline Rodrigues Campos, Marlon Benedito Nascimento Santos, Gabriela Vasconcelos Brito Bezerra
EPI_ISL_17700306, EPI_ISL_17700307, EPI_ISL_17700308, EPI_ISL_17700309, EPI_ISL_17700310, EPI_ISL_17700311, EPI_ISL_17700312, EPI_ISL_17700313	LACEN de Tocantins	Instituto Adolfo Lutz - Strategic Laboratory	Claudio Tavares Sacchi, Karoline Rodrigues Campos, Marlon Benedito Nascimento Santos, Franciano Dias Pereira Cardoso
EPI_ISL_17705015	LACEN do Parana	Instituto Adolfo Lutz - Strategic Laboratory	Claudio Tavares Sacchi, Karoline Rodrigues Campos, Marlon Benedito Nascimento Santos, Juliana Silva Nogueira
EPI_ISL_17705016	Instituto Adolfo Lutz Central	Instituto Adolfo Lutz - Strategic Laboratory	Claudio Tavares Sacchi, Karoline Rodrigues Campos, Marlon Benedito Nascimento Santos, Juliana Silva Nogueira
EPI_ISL_17722447, EPI_ISL_17722448, EPI_ISL_17722449, EPI_ISL_17722450, EPI_ISL_17722451, EPI_ISL_17722452, EPI_ISL_17722453, EPI_ISL_17722454, EPI_ISL_17722455, EPI_ISL_17722456, EPI_ISL_17722457, EPI_ISL_17722458, EPI_ISL_17722459, EPI_ISL_17722460, EPI_ISL_17722461, EPI_ISL_17722462, EPI_ISL_17722463, EPI_ISL_17722464, EPI_ISL_17722465, EPI_ISL_17722466, EPI_ISL_17722467			
see above	Blood Center of Ribeirao Preto, University of Sao Paulo	Blood Center of Ribeirao Preto, University of Sao Paulo	Marinho,R., Santos,L., Duro,R., Hunter,J., Teles,M., Milagres,F., Sabino,E., Diaz,R., Kawakubo,F., Khouri,R. and Komninakis,S.
EPI_ISL_17765199, EPI_ISL_17765200, EPI_ISL_17765201, EPI_ISL_17765202, EPI_ISL_17765203, EPI_ISL_17765204, EPI_ISL_17765205, EPI_ISL_17765206, EPI_ISL_17765207, EPI_ISL_17765208, EPI_ISL_17765209, EPI_ISL_17765210, EPI_ISL_17765211, EPI_ISL_17765212, EPI_ISL_17765213			
see above	LACEN de Tocantins	Instituto Adolfo Lutz - Strategic Laboratory	Claudio Tavares Sacchi, Karoline Rodrigues Campos, Marlon Benedito Nascimento Santos, Franciano Dias Pereira Cardoso
EPI_ISL_17765214, EPI_ISL_17765215, EPI_ISL_17765216, EPI_ISL_17765217	LACEN de Sergipe	Instituto Adolfo Lutz - Strategic Laboratory	Claudio Tavares Sacchi, Karoline Rodrigues Campos, Marlon Benedito Nascimento Santos, Gabriela Vasconcelos Brito Bezerra
EPI_ISL_17765218, EPI_ISL_17765219, EPI_ISL_17765220, EPI_ISL_17765221	Arbovirus	Instituto Adolfo Lutz - Strategic Laboratory	Claudio Tavares Sacchi, Karoline Rodrigues Campos, Marlon Benedito Nascimento Santos, Juliana Silva Nogueira
EPI_ISL_17778393	Intituto de Virologia	Intituto de Virologia	Spinsanti,L.
EPI_ISL_17797612	Institute of Virology, Wenzhou University	Institute of Virology, Wenzhou University	Xiao,P.
EPI_ISL_17809532, EPI_ISL_17809533, EPI_ISL_17809534, EPI_ISL_17809535, EPI_ISL_17809536, EPI_ISL_17809537, EPI_ISL_17809538	NIV - ICMR-National Institute of Virology - Indian Council of Medical Research - Hepatitis Group	NIV - ICMR-National Institute of Virology - Indian Council of Medical Research - Hepatitis Group	Nath,A., Kumar,S., Gurav,Y.K., Gangopadhayya,A., Ghuge,O., Sreelakshmi,P.R., Jadhav,A., Rajan,L.S., Yadav,K., Shinde,K., Shil,P., Sudeep,A. and Lole,K.S.
EPI_ISL_17885078	Mechnikov Research Institute for Vaccines and Sera	Mechnikov Research Institute for Vaccines and Sera	Ignatyev,G.M., Oksanich,A.S. and Samartseva,T.G.
EPI_ISL_17953926, EPI_ISL_17953927, EPI_ISL_17953928, EPI_ISL_17953929, EPI_ISL_17953930, EPI_ISL_17953931, EPI_ISL_17953932, EPI_ISL_17953933, EPI_ISL_17953934, EPI_ISL_17953935, EPI_ISL_17953936, EPI_ISL_17953937, EPI_ISL_17953938, EPI_ISL_17953939, EPI_ISL_17953940, EPI_ISL_17953941, EPI_ISL_17953942, EPI_ISL_17953943, EPI_ISL_17953944, EPI_ISL_17953945, EPI_ISL_17953946, EPI_ISL_17953947, EPI_ISL_17953948, EPI_ISL_17953949, EPI_ISL_17953950, EPI_ISL_17953951, EPI_ISL_17953952, EPI_ISL_17953953, EPI_ISL_17953954, EPI_ISL_17953955, EPI_ISL_17953956, EPI_ISL_17953957, EPI_ISL_17953958, EPI_ISL_17953959, EPI_ISL_17953960, EPI_ISL_17953961, EPI_ISL_17953962, EPI_ISL_17953963, EPI_ISL_17953964, EPI_ISL_17953965, EPI_ISL_17953966, EPI_ISL_17953967, EPI_ISL_17953968, EPI_ISL_17953969, EPI_ISL_17953970, EPI_ISL_17953971, EPI_ISL_17953972, EPI_ISL_17953973, EPI_ISL_17953974, EPI_ISL_17953975, EPI_ISL_17953976, EPI_ISL_17953977, EPI_ISL_17953978, EPI_ISL_17953979, EPI_ISL_17953980, EPI_ISL_17953981, EPI_ISL_17953982, EPI_ISL_17953983, EPI_ISL_17953984, EPI_ISL_17953985, EPI_ISL_17953986, EPI_ISL_17953987, EPI_ISL_17953988, EPI_ISL_17953989, EPI_ISL_17953990, EPI_ISL_17953991, EPI_ISL_17953992, EPI_ISL_17953993, EPI_ISL_17953994, EPI_ISL_17953995, EPI_ISL_17953996, EPI_ISL_17953997, EPI_ISL_17953998, EPI_ISL_17953999, EPI_ISL_17954000, EPI_ISL_17954001, EPI_ISL_17954002, EPI_ISL_17954003, EPI_ISL_17954004, EPI_ISL_17954005, EPI_ISL_17954006, EPI_ISL_17954007, EPI_ISL_17954008, EPI_ISL_17954009, EPI_ISL_17954010, EPI_ISL_17954011, EPI_ISL_17954012, EPI_ISL_17954013, EPI_ISL_17954014, EPI_ISL_17954015, EPI_ISL_17954016, EPI_ISL_17954017, EPI_ISL_17954018, EPI_ISL_17954019, EPI_ISL_17954020, EPI_ISL_17954021, EPI_ISL_17954022, EPI_ISL_17954023, EPI_ISL_17954024, EPI_ISL_17954025, EPI_ISL_17954026, EPI_ISL_17954027, EPI_ISL_17954028, EPI_ISL_17954029, EPI_ISL_17954030, EPI_ISL_17954031, EPI_ISL_17954032, EPI_ISL_17954033, EPI_ISL_17954034, EPI_ISL_17954035, EPI_ISL_17954036, EPI_ISL_17954037, EPI_ISL_17954038, EPI_ISL_17954039, EPI_ISL_17954040, EPI_ISL_17954041, EPI_ISL_17954042, EPI_ISL_17954043, EPI_ISL_17954044, EPI_ISL_17954045, EPI_ISL_17954046, EPI_ISL_17954047, EPI_ISL_17954048, EPI_ISL_17954049, EPI_ISL_17954050, EPI_ISL_17954051, EPI_ISL_17954052, EPI_ISL_17954053, EPI_ISL_17954054, EPI_ISL_17954055, EPI_ISL_17954056, EPI_ISL_17954057, EPI_ISL_17954058, EPI_ISL_17954059, EPI_ISL_17954060, EPI_ISL_17954061, EPI_ISL_17954062, EPI_ISL_17954063, EPI_ISL_17954064, EPI_ISL_17954065, EPI_ISL_17954066, EPI_ISL_17954067, EPI_ISL_17954068, EPI_ISL_17954069, EPI_ISL_17954070, EPI_ISL_17954071, EPI_ISL_17954072, EPI_ISL_17954073, EPI_ISL_17954074, EPI_ISL_17954075, EPI_ISL_17954076, EPI_ISL_17954077, EPI_ISL_17954078, EPI_ISL_17954079, EPI_ISL_17954080, EPI_ISL_17954081, EPI_ISL_17954082, EPI_ISL_17954083, EPI_ISL_17954084, EPI_ISL_17954085, EPI_ISL_17954086, EPI_ISL_17954087, EPI_ISL_17954088, EPI_ISL_17954089, EPI_ISL_17954090, EPI_ISL_17954091, EPI_ISL_17954092, EPI_ISL_17954140, EPI_ISL_17954185, EPI_ISL_17954186, EPI_ISL_17954187, EPI_ISL_17954188, EPI_ISL_17954189, EPI_ISL_17954190, EPI_ISL_17954191, EPI_ISL_17954192, EPI_ISL_17954193, EPI_ISL_17954194, EPI_ISL_17954195, EPI_ISL_17954196, EPI_ISL_17954197, EPI_ISL_17954198, EPI_ISL_17954199, EPI_ISL_17954200, EPI_ISL_17954201, EPI_ISL_17954202, EPI_ISL_17954203, EPI_ISL_17954204, EPI_ISL_17954205, EPI_ISL_17954206, EPI_ISL_17954207, EPI_ISL_17954208, EPI_ISL_17954209, EPI_ISL_17954210, EPI_ISL_17954211, EPI_ISL_17954212, EPI_ISL_17954213, EPI_ISL_17954214, EPI_ISL_17954215, EPI_ISL_17954216, EPI_ISL_17954217, EPI_ISL_17954218, EPI_ISL_17954219, EPI_ISL_17954220, EPI_ISL_17954221, EPI_ISL_17954222, EPI_ISL_17954223, EPI_ISL_17954224, EPI_ISL_17954225, EPI_ISL_17954226, EPI_ISL_17954227, EPI_ISL_17954228, EPI_ISL_17954229, EPI_ISL_17954230, EPI_ISL_17954231, EPI_ISL_17954232, EPI_ISL_17954233, EPI_ISL_17954234, EPI_ISL_17954235, EPI_ISL_17954236, EPI_ISL_17954237, EPI_ISL_17954238, EPI_ISL_17954239, EPI_ISL_17954240, EPI_ISL_17954241, EPI_ISL_17954242, EPI_ISL_17954243, EPI_ISL_17954244, EPI_ISL_17954245, EPI_ISL_17954246, EPI_ISL_17954247, EPI_ISL_17954248, EPI_ISL_17954249, EPI_ISL_17954250, EPI_ISL_17954251, EPI_ISL_17954252, EPI_ISL_17954253, EPI_ISL_17954254, EPI_ISL_17954255, EPI_ISL_17954256, EPI_ISL_17954257, EPI_ISL_17954258, EPI_ISL_17954259, EPI_ISL_17954260, EPI_ISL_17954261, EPI_ISL_17954262, EPI_ISL_17954263, EPI_ISL_17954264, EPI_ISL_17954265, EPI_ISL_17954266, EPI_ISL_17954267, EPI_ISL_17954268, EPI_ISL_17954269, EPI_ISL_17954270, EPI_ISL_17954271, EPI_ISL_17954272, EPI_ISL_17954273, EPI_ISL_17954274, EPI_ISL_17954275, EPI_ISL_17954276, EPI_ISL_17954277, EPI_ISL_17954278, EPI_ISL_17954279, EPI_ISL_17954280, EPI_ISL_17954281, EPI_ISL_17954282, EPI_ISL_17954283, EPI_ISL_17954284, EPI_ISL_17954285, EPI_ISL_17954286, EPI_ISL_17954287, EPI_ISL_17954288, EPI_ISL_17954289, EPI_ISL_17954290, EPI_ISL_17954291, EPI_ISL_17954292, EPI_ISL_17954293, EPI_ISL_17954294, EPI_ISL_17954295, EPI_ISL_17954296, EPI_ISL_17954297, EPI_ISL_17954298, EPI_ISL_17954299, EPI_ISL_17954300, EPI_ISL_17954301, EPI_ISL_17954302, EPI_ISL_17954303, EPI_ISL_17954304, EPI_ISL_17954305, EPI_ISL_17954306, EPI_ISL_17954307, EPI_ISL_17954308, EPI_ISL_17954309, EPI_ISL_17954310, EPI_ISL_17954311, EPI_ISL_17954312, EPI_ISL_17954313, EPI_ISL_17954314, EPI_ISL_17954315, EPI_ISL_17954316, EPI_ISL_17954317, EPI_ISL_17954318, EPI_ISL_17954319, EPI_ISL_17954320, EPI_ISL_17954321, EPI_ISL_17954322, EPI_ISL_17954323, EPI_ISL_17954324, EPI_ISL_17954325, EPI_ISL_17954326, EPI_ISL_17954327, EPI_ISL_17954328, EPI_ISL_17954329, EPI_ISL_17954330, EPI_ISL_17954331, EPI_ISL_17954332, EPI_ISL_17954333, EPI_ISL_17954334, EPI_ISL_17954335, EPI_ISL_17954336, EPI_ISL_17954337, EPI_ISL_17954338, EPI_ISL_17954339, EPI_ISL_17954340, EPI_ISL_17954341, EPI_ISL_17954342, EPI_ISL_17954343, EPI_ISL_17954344, EPI_ISL_17954345, EPI_ISL_17954346, EPI_ISL_17954347, EPI_ISL_17954348, EPI_ISL_17954349, EPI_ISL_17954350, EPI_ISL_17954351, EPI_ISL_17954352, EPI_ISL_17954353, EPI_ISL_17954354, EPI_ISL_17954355, EPI_ISL_17954356, EPI_ISL_17954357, EPI_ISL_17954358, EPI_ISL_17954359, EPI_ISL_17954360, EPI_ISL_17954361, EPI_ISL_17954362, EPI_ISL_17954363, EPI_ISL_17954364, EPI_ISL_17954365, EPI_ISL_17954366, EPI_ISL_17954367, EPI_ISL_17954368, EPI_ISL_17954369, EPI_ISL_17954370, EPI_ISL_17954371, EPI_ISL_17954372, EPI_ISL_17954373, EPI_ISL_17954374, EPI_ISL_17954375, EPI_ISL_17954376, EPI_ISL_17954377, EPI_ISL_17954378, EPI_ISL_17954379, EPI_ISL_17954380, EPI_ISL_17954381, EPI_ISL_17954382, EPI_ISL_17954383, EPI_ISL_17954384, EPI_ISL_17954385, EPI_ISL_17954386, EPI_ISL_17954387, EPI_ISL_17954388, EPI_ISL_17954389, EPI_ISL_17954390, EPI_ISL_17954391, EPI_ISL_17954392, EPI_ISL_17954393, EPI_ISL_17954394, EPI_ISL_17954395, EPI_ISL_17954396, EPI_ISL_17954397, EPI_ISL_17954398, EPI_ISL_17954399, EPI_ISL_17954400, EPI_ISL_17954401, EPI_ISL_17954402, EPI_ISL_17954403, EPI_ISL_17954404, EPI_ISL_17954405, EPI_ISL_17954406, EPI_ISL_17954407, EPI_ISL_17954408, EPI_ISL_17954409, EPI_ISL_17954410, EPI_ISL_17954411, EPI_ISL_17954412, EPI_ISL_17954413, EPI_ISL_17954414, EPI_ISL_17954415, EPI_ISL_17954416, EPI_ISL_17954417, EPI_ISL_17954418, EPI_ISL_17954419, EPI_ISL_17954420, EPI_ISL_17954421, EPI_ISL_17954422, EPI_ISL_17954423, EPI_ISL_17954424, EPI_ISL_17954425, EPI_ISL_17954426, EPI_ISL_17954427, EPI_ISL_17954428, EPI_ISL_17954429, EPI_ISL_17954430, EPI_ISL_17954431, EPI_ISL_17954432, EPI_ISL_17954433, EPI_ISL_17954434, EPI_ISL_17954435, EPI_ISL_17954436, EPI_ISL_17954437, EPI_ISL_17954438, EPI_ISL_17954439, EPI_ISL_17954440			
see above	Undisclosed Laboratory in Brazil for the Health Emergency Information and Risk Assessment (HIM) Unit of PAHO	Undisclosed Laboratory in Brazil for the Health Emergency Information and Risk Assessment (HIM) Unit of PAHO	Xavier,J., Fonseca,V., Oliveira,C., Lima,M., Fritsch,H., Iani,F., Adelino,T., Guimaraes,N., Castro,E., Tosta,S., Evaristo,M., Sandoval,E., Santos,E.V., Alcantara,L.C.J., Filippis,A.M.B. and Giovanetti,M.
EPI_ISL_17972132	LACEN de Tocantins	Instituto Adolfo Lutz - Strategic Laboratory	Claudio Tavares Sacchi, Karoline Rodrigues Campos, Marlon Benedito Nascimento Santos, Franciano Dias Pereira Cardoso
EPI_ISL_17972133, EPI_ISL_17972134	LACEN do Parana	Instituto Adolfo Lutz - Strategic Laboratory	Claudio Tavares Sacchi, Karoline Rodrigues Campos, Marlon Benedito Nascimento Santos
EPI_ISL_17972135, EPI_ISL_17972136, EPI_ISL_17972137, EPI_ISL_17972138	LACEN de Sergipe	Instituto Adolfo Lutz - Strategic Laboratory	Claudio Tavares Sacchi, Karoline Rodrigues Campos, Marlon Benedito Nascimento Santos, Gabriela Vasconcelos Brito Bezerra
EPI_ISL_17972139, EPI_ISL_17972140, EPI_ISL_17972141, EPI_ISL_17972142, EPI_ISL_17972143, EPI_ISL_17972144, EPI_ISL_17972145, EPI_ISL_17972146, EPI_ISL_17972147, EPI_ISL_17972148, EPI_ISL_17972149, EPI_ISL_17972150, EPI_ISL_17972151, EPI_ISL_17972152, EPI_ISL_17972153			
see above	LACEN de Tocantins	Instituto Adolfo Lutz - Strategic Laboratory	Claudio Tavares Sacchi, Karoline Rodrigues Campos, Marlon Benedito Nascimento Santos, Franciano Dias Pereira Cardoso
EPI_ISL_17972648, EPI_ISL_17972649, EPI_ISL_17972650, EPI_ISL_17972651, EPI_ISL_17972652	Undisclosed Laboratory in Brazil for the Health Emergency Information and Risk Assessment (HIM) Unit of PAHO	Undisclosed Laboratory in Brazil for the Health Emergency Information and Risk Assessment (HIM) Unit of PAHO	Xavier,J., Fonseca,V., Oliveira,C., Lima,M., Fritsch,H., Iani,F., Adelino,T., Guimaraes,N., Castro,E., Tosta,S., Evaristo,M., Sandoval,E., Santos,E.V., Alcantara,L.C.J., Filippis,A.M.B. and Giovanetti,M.
EPI_ISL_17975781, EPI_ISL_17975782, EPI_ISL_17975783, EPI_ISL_17975784, EPI_ISL_17975785, EPI_ISL_17975786, EPI_ISL_17975787, EPI_ISL_17975788, EPI_ISL_17975789, EPI_ISL_17975790	LACEN de Tocantins	Instituto Adolfo Lutz - Strategic Laboratory	Claudio Tavares Sacchi, Karoline Rodrigues Campos, Marlon Benedito Nascimento Santos, Franciano Dias Pereira Cardoso
EPI_ISL_17975791, EPI_ISL_17975792, EPI_ISL_17975793, EPI_ISL_17975794, EPI_ISL_17975795, EPI_ISL_17975796, EPI_ISL_17975797, EPI_ISL_17975798, EPI_ISL_17975799, EPI_ISL_17975800, EPI_ISL_17975801, EPI_ISL_17975802, EPI_ISL_17975803			
see above	LACEN do Parana	Instituto Adolfo Lutz - Strategic Laboratory	Claudio Tavares Sacchi, Karoline Rodrigues Campos, Marlon Benedito Nascimento Santos
EPI_ISL_17981829	Unidade Pronto Atendimento Cristo Redentor	Laboratório Central de Saúde Pública do Ceará	Tayna da Silva Fiuza, Vânia Angélica Feitosa Viana, Shirlene Telmos Silva de Lima

EPI_ISL_17981830, EPI_ISL_17981831, EPI_ISL_17981832, EPI_ISL_17981833, EPI_ISL_17981834, EPI_ISL_17981835, EPI_ISL_17981836, EPI_ISL_17981837, EPI_ISL_17981838, EPI_ISL_17981839, EPI_ISL_17981840, EPI_ISL_17981841, EPI_ISL_17981842, EPI_ISL_17981843, EPI_ISL_17981844, EPI_ISL_17981845, EPI_ISL_17981846, EPI_ISL_17981847, EPI_ISL_17981848, EPI_ISL_17981849, EPI_ISL_17981850, EPI_ISL_17981851, EPI_ISL_17981852, EPI_ISL_17981853, EPI_ISL_17981854			
see above	Servico De Verificacao De Obito Svo Sesa (Fortaleza)	Laboratório Central de Saúde Pública do Ceará	Tayna da Silva Fiuza, Vânia Angélica Feitosa Viana, Shirlene Telmos Silva de Lima
EPI_ISL_17981855	Unidade Basica De Saude Francisco Paiva De Andrade (Erere)	Laboratório Central de Saúde Pública do Ceará	Tayna da Silva Fiuza, Vânia Angélica Feitosa Viana, Shirlene Telmos Silva de Lima
EPI_ISL_18011101, EPI_ISL_18011102, EPI_ISL_18011103, EPI_ISL_18011104, EPI_ISL_18011105	LACEN-MA	Laboratório Central de Saúde Pública do Ceará	Tayna da Silva Fiuza, Vânia Angélica Feitosa Viana, Shirlene Telmos Silva de Lima on behalf of LACEN-MA
EPI_ISL_18011106, EPI_ISL_18011107	LACEN-PB	Laboratório Central de Saúde Pública do Ceará	Tayna da Silva Fiuza, Vânia Angélica Feitosa Viana, Shirlene Telmos Silva de Lima on behalf of LACEN-PB
EPI_ISL_18011108, EPI_ISL_18011109, EPI_ISL_18011110, EPI_ISL_18011111	LACEN-RN	Laboratório Central de Saúde Pública do Ceará	Tayna da Silva Fiuza, Vânia Angélica Feitosa Viana, Shirlene Telmos Silva de Lima on behalf of LACEN-RN
EPI_ISL_18011112	Hospital Geral Dr Waldemar Alcantara	Laboratório Central de Saúde Pública do Ceará	Tayna da Silva Fiuza, Vânia Angélica Feitosa Viana, Shirlene Telmos Silva de Lima
EPI_ISL_18011113, EPI_ISL_18011114, EPI_ISL_18011115, EPI_ISL_18011116	Hospital Municipal Raimunda Timbo Camelo (Tamboril)	Laboratório Central de Saúde Pública do Ceará	Tayna da Silva Fiuza, Vânia Angélica Feitosa Viana, Shirlene Telmos Silva de Lima
EPI_ISL_18011117	Unidade Pronto Atendimento Jangurussu	Laboratório Central de Saúde Pública do Ceará	Tayna da Silva Fiuza, Vânia Angélica Feitosa Viana, Shirlene Telmos Silva de Lima
EPI_ISL_18011118	Hospital Municipal Raimunda Timbo Camelo (Tamboril)	Laboratório Central de Saúde Pública do Ceará	Tayna da Silva Fiuza, Vânia Angélica Feitosa Viana, Shirlene Telmos Silva de Lima
EPI_ISL_18011453, EPI_ISL_18011454, EPI_ISL_18011455, EPI_ISL_18011456, EPI_ISL_18011457, EPI_ISL_18011458, EPI_ISL_18011459, EPI_ISL_18011460, EPI_ISL_18011461, EPI_ISL_18011462, EPI_ISL_18011463			
see above	Instituto Adolfo Lutz - Strategic Laboratory	Instituto Adolfo Lutz - Strategic Laboratory	Sacchi,C.T., Campos,K.R. and Santos,M.B.N.
EPI_ISL_18011464	Instituto Adolfo Lutz - Strategic Laboratory	Instituto Adolfo Lutz - Strategic Laboratory	Sacchi,C.T., Campos,K.R., Santos,M.B.N. and Nogueira,J.S.
EPI_ISL_18011465, EPI_ISL_18011466	Instituto Adolfo Lutz - Strategic Laboratory	Instituto Adolfo Lutz - Strategic Laboratory	Sacchi,C.T., Campos,K.R. and Santos,M.B.N.
EPI_ISL_18041742, EPI_ISL_18041743, EPI_ISL_18041744, EPI_ISL_18041745, EPI_ISL_18041746, EPI_ISL_18041747, EPI_ISL_18041748, EPI_ISL_18041749	Infection and Immunology, Translational Health Science and Technology Institute	Infection and Immunology, Translational Health Science and Technology Institute	Kumar,P., Singh,B., Shashi,P., Lodha,R. and Medigeshi,G.
EPI_ISL_18044775, EPI_ISL_18044776, EPI_ISL_18044777, EPI_ISL_18044778, EPI_ISL_18044779, EPI_ISL_18044780, EPI_ISL_18044781, EPI_ISL_18044782, EPI_ISL_18044783, EPI_ISL_18044784, EPI_ISL_18044785, EPI_ISL_18044786, EPI_ISL_18044787, EPI_ISL_18044788, EPI_ISL_18044789, EPI_ISL_18044790, EPI_ISL_18044791, EPI_ISL_18044792, EPI_ISL_18044793, EPI_ISL_18044794, EPI_ISL_18044795, EPI_ISL_18044796, EPI_ISL_18044797, EPI_ISL_18044798, EPI_ISL_18044799, EPI_ISL_18044800, EPI_ISL_18044801, EPI_ISL_18044802, EPI_ISL_18044803, EPI_ISL_18044804, EPI_ISL_18044805, EPI_ISL_18044806, EPI_ISL_18044807, EPI_ISL_18044808, EPI_ISL_18044809, EPI_ISL_18044810, EPI_ISL_18044811, EPI_ISL_18044812, EPI_ISL_18044813, EPI_ISL_18044814, EPI_ISL_18044815, EPI_ISL_18044816, EPI_ISL_18044817, EPI_ISL_18044818, EPI_ISL_18044819, EPI_ISL_18044820, EPI_ISL_18044821, EPI_ISL_18044822, EPI_ISL_18044823, EPI_ISL_18044824, EPI_ISL_18044825, EPI_ISL_18044826, EPI_ISL_18044827, EPI_ISL_18044828, EPI_ISL_18044829, EPI_ISL_18044830, EPI_ISL_18044831, EPI_ISL_18044832, EPI_ISL_18044833, EPI_ISL_18044834, EPI_ISL_18044835, EPI_ISL_18044836, EPI_ISL_18044837, EPI_ISL_18044838, EPI_ISL_18044839, EPI_ISL_18044840, EPI_ISL_18044841, EPI_ISL_18044842, EPI_ISL_18044843, EPI_ISL_18044844, EPI_ISL_18044845, EPI_ISL_18044846, EPI_ISL_18044847, EPI_ISL_18044848, EPI_ISL_18044849, EPI_ISL_18044850, EPI_ISL_18044851, EPI_ISL_18044852, EPI_ISL_18044853, EPI_ISL_18044854, EPI_ISL_18044855, EPI_ISL_18044856, EPI_ISL_18044857, EPI_ISL_18044858, EPI_ISL_18044859, EPI_ISL_18044860, EPI_ISL_18044861, EPI_ISL_18044862, EPI_ISL_18044863, EPI_ISL_18044864, EPI_ISL_18044865, EPI_ISL_18044866, EPI_ISL_18044867, EPI_ISL_18044868, EPI_ISL_18044869, EPI_ISL_18044870, EPI_ISL_18044871, EPI_ISL_18044872, EPI_ISL_18044873, EPI_ISL_18044874, EPI_ISL_18044875, EPI_ISL_18044876, EPI_ISL_18044877, EPI_ISL_18044878, EPI_ISL_18044879, EPI_ISL_18044880, EPI_ISL_18044881, EPI_ISL_18044882, EPI_ISL_18044883, EPI_ISL_18044884, EPI_ISL_18044885, EPI_ISL_18044886, EPI_ISL_18044887, EPI_ISL_18044888, EPI_ISL_18044889, EPI_ISL_18044890, EPI_ISL_18044891, EPI_ISL_18044892, EPI_ISL_18044893, EPI_ISL_18044894, EPI_ISL_18044895, EPI_ISL_18044896, EPI_ISL_18044897, EPI_ISL_18044898, EPI_ISL_18044899, EPI_ISL_18044900, EPI_ISL_18044901, EPI_ISL_18044902, EPI_ISL_18044903, EPI_ISL_18044904, EPI_ISL_18044905, EPI_ISL_18044906, EPI_ISL_18044907, EPI_ISL_18044908, EPI_ISL_18044909, EPI_ISL_18044910, EPI_ISL_18044911, EPI_ISL_18044912, EPI_ISL_18044913, EPI_ISL_18044914, EPI_ISL_18044915, EPI_ISL_18044916, EPI_ISL_18044917, EPI_ISL_18044918, EPI_ISL_18044919, EPI_ISL_18044920, EPI_ISL_18044921, EPI_ISL_18044922, EPI_ISL_18044923, EPI_ISL_18044924, EPI_ISL_18044925, EPI_ISL_18044926, EPI_ISL_18044927, EPI_ISL_18044928, EPI_ISL_18044929, EPI_ISL_18044930, EPI_ISL_18044931, EPI_ISL_18044932, EPI_ISL_18044933, EPI_ISL_18044934, EPI_ISL_18044935, EPI_ISL_18044936, EPI_ISL_18044937, EPI_ISL_18044938, EPI_ISL_18044939, EPI_ISL_18044940, EPI_ISL_18044941, EPI_ISL_18044942, EPI_ISL_18044943, EPI_ISL_18044944, EPI_ISL_18044945, EPI_ISL_18044946, EPI_ISL_18044947, EPI_ISL_18044948			
see above	Undisclosed Laboratory in Brazil for the Health Emergency Information and Risk Assessment (HIM) Unit of PAHO	Undisclosed Laboratory in Brazil for the Health Emergency Information and Risk Assessment (HIM) Unit of PAHO	Giovanetti,M., Vazquez,C., Lima,M., Castro,E., Rojas,A., Gomez de la Fuente,A., Aquino,C., Cantero,C., Fleitas,F., Torales,J., Barrios,J., Ortega,M.J., Gamarra,M.L., Villalba,S., Alfonzo,T., Xavier,J., Adelino,T., Fritsch,H., Iani,F.C.M., Pereira,G.C., de Oliveira,C., Schuab,G., Rodrigues,E.S., Kashima,S., Leite,J., Gresh,L., Franco,L., Tegally,H., Van Voorhis,W.C., Lessels,R., Bispo de Filippis,A.M., Ojeda,A., Sequera,G., Montoya,R., Holmes,E.C., de Oliveira,T., Roco,J.M., Lourenco,J., Fonseca,V. and Alcantara,L.C.J.
EPI_ISL_18064578	Undisclosed Laboratory in Brazil for the Health Emergency Information and Risk Assessment (HIM) Unit of PAHO	Undisclosed Laboratory in Brazil for the Health Emergency Information and Risk Assessment (HIM) Unit of PAHO	Giovanetti,M., Vazquez,C., Lima,M., Castro,E., Rojas,A., Gomez de la Fuente,A., Aquino,C., Cantero,C., Fleitas,F., Torales,J., Barrios,J., Ortega,M.J., Gamarra,M.L., Villalba,S., Alfonzo,T., Xavier,J., Adelino,T., Fritsch,H., Iani,F.C.M., Pereira,G.C., de Oliveira,C., Schuab,G., Rodrigues,E.S., Kashima,S., Leite,J., Gresh,L., Franco,L., Tegally,H., Van Voorhis,W.C., Lessels,R., de Filippis,A.M.B., Ojeda,A., Sequera,G., Montoya,R., Holmes,E.C., de Oliveira,T., Rico,J.M., Lourenco,J., Fonseca,V. and Alcantara,L.C.J.
EPI_ISL_18064579	Undisclosed Laboratory in Brazil for the Health Emergency Information and Risk Assessment (HIM) Unit of PAHO	Undisclosed Laboratory in Brazil for the Health Emergency Information and Risk Assessment (HIM) Unit of PAHO	Vazquez,C., Giovanetti,M., Fonseca,V., Alcantara,L.C.J. and Filippis,A.M.B.
EPI_ISL_18064580, EPI_ISL_18064581	Undisclosed Laboratory in Brazil for the Health Emergency Information and Risk Assessment (HIM) Unit of PAHO	Undisclosed Laboratory in Brazil for the Health Emergency Information and Risk Assessment (HIM) Unit of PAHO	Giovanetti,M., Vazquez,C., Lima,M., Castro,E., Rojas,A., Gomez de la Fuente,A., Aquino,C., Cantero,C., Fleitas,F., Torales,J., Barrios,J., Ortega,M.J., Gamarra,M.L., Villalba,S., Alfonzo,T., Xavier,J., Adelino,T., Fritsch,H., Iani,F.C.M., Pereira,G.C., de Oliveira,C., Schuab,G., Rodrigues,E.S., Kashima,S., Leite,J., Gresh,L., Franco,L., Tegally,H., Van Voorhis,W.C., Lessels,R., de Filippis,A.M.B., Ojeda,A., Sequera,G., Montoya,R., Holmes,E.C., de Oliveira,T., Rico,J.M., Lourenco,J., Fonseca,V. and Alcantara,L.C.J.
EPI_ISL_18064582	Undisclosed Laboratory in Brazil for the Health Emergency Information and Risk Assessment (HIM) Unit of PAHO	Undisclosed Laboratory in Brazil for the Health Emergency Information and Risk Assessment (HIM) Unit of PAHO	Vazquez,C., Giovanetti,M., Fonseca,V., Alcantara,L.C.J. and Filippis,A.M.B.
EPI_ISL_18064583	Undisclosed Laboratory in Brazil for the Health Emergency Information and Risk Assessment (HIM) Unit of PAHO	Undisclosed Laboratory in Brazil for the Health Emergency Information and Risk Assessment (HIM) Unit of PAHO	Giovanetti,M., Vazquez,C., Lima,M., Castro,E., Rojas,A., Gomez de la Fuente,A., Aquino,C., Cantero,C., Fleitas,F., Torales,J., Barrios,J., Ortega,M.J., Gamarra,M.L., Villalba,S., Alfonzo,T., Xavier,J., Adelino,T., Fritsch,H., Iani,F.C.M., Pereira,G.C., de Oliveira,C., Schuab,G., Rodrigues,E.S., Kashima,S., Leite,J., Gresh,L., Franco,L., Tegally,H., Van Voorhis,W.C., Lessels,R., de Filippis,A.M.B., Ojeda,A., Sequera,G., Montoya,R., Holmes,E.C., de Oliveira,T., Rico,J.M., Lourenco,J., Fonseca,V. and Alcantara,L.C.J.
EPI_ISL_18064584	Undisclosed Laboratory in Brazil for the Health Emergency Information and Risk Assessment (HIM) Unit of PAHO	Undisclosed Laboratory in Brazil for the Health Emergency Information and Risk Assessment (HIM) Unit of PAHO	Vazquez,C., Giovanetti,M., Fonseca,V., Alcantara,L.C.J. and Filippis,A.M.B.
EPI_ISL_18095507, EPI_ISL_18095508, EPI_ISL_18095509, EPI_ISL_18095510, EPI_ISL_18095511, EPI_ISL_18095512, EPI_ISL_18095513, EPI_ISL_18095514, EPI_ISL_18095515, EPI_ISL_18095516, EPI_ISL_18095517, EPI_ISL_18095518, EPI_ISL_18095519, EPI_ISL_18095520, EPI_ISL_18095521			
see above	LACEN do Rio de Janeiro	Instituto Adolfo Lutz - Strategic Laboratory	Claudio Tavares Sacchi, Claudia Regina Gonçalves, Karoline Rodrigues Campos, Marlon Benedito N. Santos, Ariadne Ferreira Amarante, Maria Carmelita dos Santos Vaz
EPI_ISL_18095522, EPI_ISL_18095523, EPI_ISL_18095524, EPI_ISL_18095525, EPI_ISL_18095526, EPI_ISL_18095527, EPI_ISL_18095528, EPI_ISL_18095529, EPI_ISL_18095530, EPI_ISL_18095531, EPI_ISL_18095532, EPI_ISL_18095533, EPI_ISL_18095534, EPI_ISL_18095535, EPI_ISL_18095536, EPI_ISL_18095537, EPI_ISL_18095538			
see above	LACEN de Tocantins	Instituto Adolfo Lutz - Strategic Laboratory	Claudio Tavares Sacchi, Claudia Regina Gonçalves, Karoline Rodrigues Campos, Marlon Benedito N. Santos, Ariadne Ferreira Amarante, Franciano Dias Pereira Cardoso
EPI_ISL_18106751, EPI_ISL_18106752, EPI_ISL_18106753, EPI_ISL_18106754, EPI_ISL_18106755, EPI_ISL_18106756, EPI_ISL_18106757, EPI_ISL_18106758, EPI_ISL_18106759, EPI_ISL_18106760, EPI_ISL_18106761, EPI_ISL_18106762, EPI_ISL_18106763, EPI_ISL_18106764, EPI_ISL_18106765, EPI_ISL_18106766, EPI_ISL_18106767, EPI_ISL_18106768, EPI_ISL_18106769, EPI_ISL_18106770, EPI_ISL_18106771, EPI_ISL_18106772, EPI_ISL_18106773, EPI_ISL_18106774, EPI_ISL_18106775, EPI_ISL_18106776, EPI_ISL_18106777, EPI_ISL_18106778, EPI_ISL_18106779, EPI_ISL_18106780			
see above	Central Public Health Laboratory Goncalo Moniz	Central Public Health Laboratory Goncalo Moniz	Rego,F., Andrade,G., Lima,T., Nardy,V., Leal,A. and Pereira,F.
EPI_ISL_18106781, EPI_ISL_18106782, EPI_ISL_18106783, EPI_ISL_18106784, EPI_ISL_18106785	International Centre for Genetic Engineering and Biotechnology	International Centre for Genetic Engineering and Biotechnology	Dixit,K., Nayak,K., Khan,N., Jain,V., Sunil,S., Medigeshi,G.R., Ray,P., Kaja,M.K. and Chandele,A.
EPI_ISL_18123967, EPI_ISL_18123968, EPI_ISL_18123969, EPI_ISL_18123970, EPI_ISL_18123971, EPI_ISL_18123972, EPI_ISL_18123973, EPI_ISL_18123974, EPI_ISL_18123975, EPI_ISL_18123976, EPI_ISL_18123977, EPI_ISL_18123978, EPI_ISL_18123979, EPI_ISL_18123980, EPI_ISL_18123981, EPI_ISL_18123982, EPI_ISL_18123983, EPI_ISL_18123984, EPI_ISL_18123985, EPI_ISL_18123986, EPI_ISL_18123987, EPI_ISL_18123988, EPI_ISL_18123989, EPI_ISL_18123990, EPI_ISL_18123991, EPI_ISL_18123992, EPI_ISL_18123993, EPI_ISL_18123994, EPI_ISL_18123995, EPI_ISL_18123996, EPI_ISL_18123997, EPI_ISL_18123998, EPI_ISL_18123999, EPI_ISL_18124000, EPI_ISL_18124001, EPI_ISL_18124002, EPI_ISL_18124003, EPI_ISL_18124004, EPI_ISL_18124005, EPI_ISL_18124006, EPI_ISL_18124007, EPI_ISL_18124008, EPI_ISL_18124009, EPI_ISL_18124010, EPI_ISL_18124011, EPI_ISL_18124012, EPI_ISL_18124013, EPI_ISL_18124014, EPI_ISL_18124015, EPI_ISL_18124016, EPI_ISL_18124017, EPI_ISL_18124018, EPI_ISL_18124019, EPI_ISL_18124020, EPI_ISL_18124021, EPI_ISL_18124022, EPI_ISL_18124023, EPI_ISL_18124024, EPI_ISL_18124025, EPI_ISL_18124026, EPI_ISL_18124027, EPI_ISL_18124028, EPI_ISL_18124029, EPI_ISL_18124030			
see above	Central Public Health Laboratory - LACEN - Salvador, Bahia, Brazil	Central Public Health Laboratory - LACEN - Salvador, Bahia, Brazil	Filipe Rego, Vanessa Nardy, Gabriela Menezes, Aline Salomao, Felicidade Pereira, Arabela Leal
EPI_ISL_18131736, EPI_ISL_18131737, EPI_ISL_18131738, EPI_ISL_18131739, EPI_ISL_18131740	Department of Virology and Immunity, Guangzhou Center for Disease Control and Prevention	Department of Virology and Immunity, Guangzhou Center for Disease Control and Prevention	Su,W.
EPI_ISL_18220031, EPI_ISL_18220032, EPI_ISL_18220033, EPI_ISL_18220034, EPI_ISL_18220035, EPI_ISL_18220036, EPI_ISL_18220037, EPI_ISL_18220038, EPI_ISL_18220039, EPI_ISL_18220040, EPI_ISL_18220041, EPI_ISL_18220042, EPI_ISL_18220043, EPI_ISL_18220044, EPI_ISL_18220045, EPI_ISL_18220046, EPI_ISL_18220047, EPI_ISL_18220048, EPI_ISL_18220049, EPI_ISL_18220050, EPI_ISL_18220051, EPI_ISL_18220052, EPI_ISL_18220053, EPI_ISL_18220054, EPI_ISL_18220055, EPI_ISL_18220056, EPI_ISL_18220057, EPI_ISL_18220058, EPI_ISL_18220059, EPI_ISL_18220060, EPI_ISL_18220061, EPI_ISL_18220062, EPI_ISL_18220063, EPI_ISL_18220064, EPI_ISL_18220065, EPI_ISL_18220066, EPI_ISL_18220067			
see above	Central Laboratory of Public Health of the Espirito Santo - Lacen ES	Central Laboratory of Public Health of the Espirito Santo - Lacen ES	Emerson de Castro Barbosa, Felipe Donateli Gatti, Gabriela Colombo de Mendonça, Geovani de Oliveira Ribeiro, Joana Zorzal Nodari, Lucas André Silva Bonela, Luiz Carlos Junior Alcantara, Mariane Evaristo, Maurício Teixeira Lima, Natália Rocha Guimarães, Thiago de Jesus Sousa, Vagner de Souza Fonseca, Jaqueline Pegoretti Goulart, Rodrigo Ribeiro Rodrigues as General Coordinator
EPI_ISL_18230334, EPI_ISL_18230335	NC - Molecular Biology, Central Public Health Laboratory Dr. Giovanni Cysneiros (LACEN-GO)	NC - Molecular Biology, Central Public Health Laboratory Dr. Giovanni Cysneiros (LACEN-GO)	Mendonca,A.F., Andrade,C.F., Sobreiro,M.B., Almeida,R.M., Silva,V.L. and Pereira,L.A.
EPI_ISL_18230336, EPI_ISL_18230337	NC - Molecular Biology, Central Public Health Laboratory Dr. Giovanni Cysneiros (LACEN-GO)	NC - Molecular Biology, Central Public Health Laboratory Dr. Giovanni Cysneiros (LACEN-GO)	Mendonca,A.
EPI_ISL_18230338	NC - Molecular Biology, Central Public Health Laboratory Dr. Giovanni Cysneiros (LACEN-GO)	NC - Molecular Biology, Central Public Health Laboratory Dr. Giovanni Cysneiros (LACEN-GO)	Mendonca,A.F., Andrade,C.F., Sobreiro,M.B., Almeida,R.M., Silva,V.L. and Pereira,L.A.
EPI_ISL_18230339	NC - Molecular Biology, Central Public Health Laboratory Dr. Giovanni Cysneiros (LACEN-GO)	NC - Molecular Biology, Central Public Health Laboratory Dr. Giovanni Cysneiros (LACEN-GO)	Mendonca,A.
EPI_ISL_18230340, EPI_ISL_18230341	NC - Molecular Biology, Central Public Health Laboratory Dr. Giovanni Cysneiros (LACEN-GO)	NC - Molecular Biology, Central Public Health Laboratory Dr. Giovanni Cysneiros (LACEN-GO)	Mendonca,A.F., Andrade,C.F., Sobreiro,M.B., Almeida,R.M., Silva,V.L. and Pereira,L.A.
EPI_ISL_18230342, EPI_ISL_18230343	NC - Molecular Biology, Central Public Health Laboratory Dr. Giovanni Cysneiros (LACEN-GO)	NC - Molecular Biology, Central Public Health Laboratory Dr. Giovanni Cysneiros (LACEN-GO)	Mendonca,A.
EPI_ISL_18230344	NC - Molecular Biology, Central Public Health Laboratory Dr. Giovanni Cysneiros (LACEN-GO)	NC - Molecular Biology, Central Public Health Laboratory Dr. Giovanni Cysneiros (LACEN-GO)	Mendonca,A.F., Andrade,C.F., Sobreiro,M.B., Almeida,R.M., Silva,V.L. and Pereira,L.A.
EPI_ISL_18230345	NC - Molecular Biology, Central Public Health	NC - Molecular Biology, Central Public Health	Mendonca,A.

	Laboratory Dr. Giovanni Cysneiros (LACEN-GO)	Laboratory Dr. Giovanni Cysneiros (LACEN-GO)	
EPI_ISL_18230346	NC - Molecular Biology, Central Public Health Laboratory Dr. Giovanni Cysneiros (LACEN-GO)	NC - Molecular Biology, Central Public Health Laboratory Dr. Giovanni Cysneiros (LACEN-GO)	Mendonca,A.F., Andrade,C.F., Sobreiro,M.B., Almeida,R.M., Silva,V.L. and Pereira,L.A.
EPI_ISL_18230347	NC - Molecular Biology, Central Public Health Laboratory Dr. Giovanni Cysneiros (LACEN-GO)	NC - Molecular Biology, Central Public Health Laboratory Dr. Giovanni Cysneiros (LACEN-GO)	Mendonca,A.
EPI_ISL_18230348, EPI_ISL_18230349, EPI_ISL_18230350, EPI_ISL_18230351, EPI_ISL_18230352	NC - Molecular Biology, Central Public Health Laboratory Dr. Giovanni Cysneiros (LACEN-GO)	NC - Molecular Biology, Central Public Health Laboratory Dr. Giovanni Cysneiros (LACEN-GO)	Mendonca,A.F., Andrade,C.F., Sobreiro,M.B., Almeida,R.M., Silva,V.L. and Pereira,L.A.
EPI_ISL_18230353, EPI_ISL_18230354	NC - Molecular Biology, Central Public Health Laboratory Dr. Giovanni Cysneiros (LACEN-GO)	NC - Molecular Biology, Central Public Health Laboratory Dr. Giovanni Cysneiros (LACEN-GO)	Mendonca,A.
EPI_ISL_18230355, EPI_ISL_18230356, EPI_ISL_18230357, EPI_ISL_18230358	NC - Molecular Biology, Central Public Health Laboratory Dr. Giovanni Cysneiros (LACEN-GO)	NC - Molecular Biology, Central Public Health Laboratory Dr. Giovanni Cysneiros (LACEN-GO)	Mendonca,A.F., Andrade,C.F., Sobreiro,M.B., Almeida,R.M., Silva,V.L. and Pereira,L.A.
EPI_ISL_18230359	NC - Molecular Biology, Central Public Health Laboratory Dr. Giovanni Cysneiros (LACEN-GO)	NC - Molecular Biology, Central Public Health Laboratory Dr. Giovanni Cysneiros (LACEN-GO)	Mendonca,A.
EPI_ISL_18230360, EPI_ISL_18230361, EPI_ISL_18230362, EPI_ISL_18230363, EPI_ISL_18230364	NC - Molecular Biology, Central Public Health Laboratory Dr. Giovanni Cysneiros (LACEN-GO)	NC - Molecular Biology, Central Public Health Laboratory Dr. Giovanni Cysneiros (LACEN-GO)	Mendonca,A.F., Andrade,C.F., Sobreiro,M.B., Almeida,R.M., Silva,V.L. and Pereira,L.A.
EPI_ISL_18230365	NC - Molecular Biology, Central Public Health Laboratory Dr. Giovanni Cysneiros (LACEN-GO)	NC - Molecular Biology, Central Public Health Laboratory Dr. Giovanni Cysneiros (LACEN-GO)	Mendonca,A.
EPI_ISL_18230366	NC - Molecular Biology, Central Public Health Laboratory Dr. Giovanni Cysneiros (LACEN-GO)	NC - Molecular Biology, Central Public Health Laboratory Dr. Giovanni Cysneiros (LACEN-GO)	Mendonca,A.F., Andrade,C.F., Sobreiro,M.B., Almeida,R.M., Silva,V.L. and Pereira,L.A.
EPI_ISL_18230367, EPI_ISL_18230368	NC - Molecular Biology, Central Public Health Laboratory Dr. Giovanni Cysneiros (LACEN-GO)	NC - Molecular Biology, Central Public Health Laboratory Dr. Giovanni Cysneiros (LACEN-GO)	Mendonca,A.
EPI_ISL_18230369, EPI_ISL_18230370	NC - Molecular Biology, Central Public Health Laboratory Dr. Giovanni Cysneiros (LACEN-GO)	NC - Molecular Biology, Central Public Health Laboratory Dr. Giovanni Cysneiros (LACEN-GO)	Mendonca,A.F., Andrade,C.F., Sobreiro,M.B., Almeida,R.M., Silva,V.L. and Pereira,L.A.
EPI_ISL_18230371	NC - Molecular Biology, Central Public Health Laboratory Dr. Giovanni Cysneiros (LACEN-GO)	NC - Molecular Biology, Central Public Health Laboratory Dr. Giovanni Cysneiros (LACEN-GO)	Mendonca,A.
EPI_ISL_18230372	NC - Molecular Biology, Central Public Health Laboratory Dr. Giovanni Cysneiros (LACEN-GO)	NC - Molecular Biology, Central Public Health Laboratory Dr. Giovanni Cysneiros (LACEN-GO)	Mendonca,A.F., Andrade,C.F., Sobreiro,M.B., Almeida,R.M., Silva,V.L. and Pereira,L.A.
EPI_ISL_18230373	NC - Molecular Biology, Central Public Health Laboratory Dr. Giovanni Cysneiros (LACEN-GO)	NC - Molecular Biology, Central Public Health Laboratory Dr. Giovanni Cysneiros (LACEN-GO)	Mendonca,A.
EPI_ISL_18230374, EPI_ISL_18230375	NC - Molecular Biology, Central Public Health Laboratory Dr. Giovanni Cysneiros (LACEN-GO)	NC - Molecular Biology, Central Public Health Laboratory Dr. Giovanni Cysneiros (LACEN-GO)	Mendonca,A.F., Andrade,C.F., Sobreiro,M.B., Almeida,R.M., Silva,V.L. and Pereira,L.A.
EPI_ISL_18230376, EPI_ISL_18230377	NC - Molecular Biology, Central Public Health Laboratory Dr. Giovanni Cysneiros (LACEN-GO)	NC - Molecular Biology, Central Public Health Laboratory Dr. Giovanni Cysneiros (LACEN-GO)	Mendonca,A.
EPI_ISL_18230378	NC - Molecular Biology, Central Public Health Laboratory Dr. Giovanni Cysneiros (LACEN-GO)	NC - Molecular Biology, Central Public Health Laboratory Dr. Giovanni Cysneiros (LACEN-GO)	Mendonca,A.F., Andrade,C.F., Sobreiro,M.B., Almeida,R.M., Silva,V.L. and Pereira,L.A.
EPI_ISL_18230379, EPI_ISL_18230380	NC - Molecular Biology, Central Public Health Laboratory Dr. Giovanni Cysneiros (LACEN-GO)	NC - Molecular Biology, Central Public Health Laboratory Dr. Giovanni Cysneiros (LACEN-GO)	Mendonca,A.
EPI_ISL_18230381, EPI_ISL_18230382, EPI_ISL_18230383	NC - Molecular Biology, Central Public Health Laboratory Dr. Giovanni Cysneiros (LACEN-GO)	NC - Molecular Biology, Central Public Health Laboratory Dr. Giovanni Cysneiros (LACEN-GO)	Mendonca,A.F., Andrade,C.F., Sobreiro,M.B., Almeida,R.M., Silva,V.L. and Pereira,L.A.
EPI_ISL_18230384, EPI_ISL_18230385, EPI_ISL_18230386, EPI_ISL_18230387	NC - Molecular Biology, Central Public Health Laboratory Dr. Giovanni Cysneiros (LACEN-GO)	NC - Molecular Biology, Central Public Health Laboratory Dr. Giovanni Cysneiros (LACEN-GO)	Mendonca,A.
EPI_ISL_18230388	NC - Molecular Biology, Central Public Health Laboratory Dr. Giovanni Cysneiros (LACEN-GO)	NC - Molecular Biology, Central Public Health Laboratory Dr. Giovanni Cysneiros (LACEN-GO)	Mendonca,A.F., Andrade,C.F., Sobreiro,M.B., Almeida,R.M., Silva,V.L. and Pereira,L.A.
EPI_ISL_18230389	NC - Molecular Biology, Central Public Health Laboratory Dr. Giovanni Cysneiros (LACEN-GO)	NC - Molecular Biology, Central Public Health Laboratory Dr. Giovanni Cysneiros (LACEN-GO)	Mendonca,A.
EPI_ISL_18230390, EPI_ISL_18230391	NC - Molecular Biology, Central Public Health Laboratory Dr. Giovanni Cysneiros (LACEN-GO)	NC - Molecular Biology, Central Public Health Laboratory Dr. Giovanni Cysneiros (LACEN-GO)	Mendonca,A.F., Andrade,C.F., Sobreiro,M.B., Almeida,R.M., Silva,V.L. and Pereira,L.A.
EPI_ISL_18230392, EPI_ISL_18230393	NC - Molecular Biology, Central Public Health Laboratory Dr. Giovanni Cysneiros (LACEN-GO)	NC - Molecular Biology, Central Public Health Laboratory Dr. Giovanni Cysneiros (LACEN-GO)	Mendonca,A.
EPI_ISL_18230394, EPI_ISL_18230395, EPI_ISL_18230396	NC - Molecular Biology, Central Public Health Laboratory Dr. Giovanni Cysneiros (LACEN-GO)	NC - Molecular Biology, Central Public Health Laboratory Dr. Giovanni Cysneiros (LACEN-GO)	Mendonca,A.F., Andrade,C.F., Sobreiro,M.B., Almeida,R.M., Silva,V.L. and Pereira,L.A.
EPI_ISL_18230397	NC - Molecular Biology, Central Public Health Laboratory Dr. Giovanni Cysneiros (LACEN-GO)	NC - Molecular Biology, Central Public Health Laboratory Dr. Giovanni Cysneiros (LACEN-GO)	Mendonca,A.
EPI_ISL_18230398, EPI_ISL_18230399	NC - Molecular Biology, Central Public Health Laboratory Dr. Giovanni Cysneiros (LACEN-GO)	NC - Molecular Biology, Central Public Health Laboratory Dr. Giovanni Cysneiros (LACEN-GO)	Mendonca,A.F., Andrade,C.F., Sobreiro,M.B., Almeida,R.M., Silva,V.L. and Pereira,L.A.
EPI_ISL_18230400	NC - Molecular Biology, Central Public Health Laboratory Dr. Giovanni Cysneiros (LACEN-GO)	NC - Molecular Biology, Central Public Health Laboratory Dr. Giovanni Cysneiros (LACEN-GO)	Mendonca,A.
EPI_ISL_18230401, EPI_ISL_18230402	NC - Molecular Biology, Central Public Health Laboratory Dr. Giovanni Cysneiros (LACEN-GO)	NC - Molecular Biology, Central Public Health Laboratory Dr. Giovanni Cysneiros (LACEN-GO)	Mendonca,A.F., Andrade,C.F., Sobreiro,M.B., Almeida,R.M., Silva,V.L. and Pereira,L.A.
EPI_ISL_18274468, EPI_ISL_18274469, EPI_ISL_18274470, EPI_ISL_18274471, EPI_ISL_18274472, EPI_ISL_18274473, EPI_ISL_18274474	LACEN de Sergipe	Instituto Adolfo Lutz, Rapid Response Center, Strategic Laboratory	Claudio Tavares Sacchi, Claudia Regina Gonçalves, Karoline Rodrigues Campos, Marlon Benedito N. Santos, Ariadne Ferreira Amarante, Gabriela Vasconcelos Brito Bezerra
EPI_ISL_18274475, EPI_ISL_18274476, EPI_ISL_18274477, EPI_ISL_18274478, EPI_ISL_18274479, EPI_ISL_18274480, EPI_ISL_18274481, EPI_ISL_18274482, EPI_ISL_18274483, EPI_ISL_18274484, EPI_ISL_18274485, EPI_ISL_18274486, EPI_ISL_18274487, EPI_ISL_18274488, EPI_ISL_18274489, EPI_ISL_18274490, EPI_ISL_18274491, EPI_ISL_18274492, EPI_ISL_18274493, EPI_ISL_18274494, EPI_ISL_18274495, EPI_ISL_18274496, EPI_ISL_18274497, EPI_ISL_18274498, EPI_ISL_18274499, EPI_ISL_18274500, EPI_ISL_18274501, EPI_ISL_18274502, EPI_ISL_18274503, EPI_ISL_18274504			
see above	LACEN de Tocantins	Instituto Adolfo Lutz, Rapid Response Center, Strategic Laboratory	Claudio Tavares Sacchi, Claudia Regina Gonçalves, Karoline Rodrigues Campos, Marlon Benedito N. Santos, Ariadne Ferreira Amarante, Franciano Dias Pereira Cardoso
EPI_ISL_18360695, EPI_ISL_18360696, EPI_ISL_18360697, EPI_ISL_18360698, EPI_ISL_18360699, EPI_ISL_18360700, EPI_ISL_18360701, EPI_ISL_18360702, EPI_ISL_18360703	Department of Microbiology, University of Sao Paulo	Department of Microbiology, University of Sao Paulo	Cirilo,M.V.F., Zaki Pour,S., Benedetti,V.F., Farias,J.P., Fogaca,M.M.C., Simoes,R.C., Vidal,P.O., Birbrair,A., Zanotto,P.M.A., Luiz,W.B. and Amorim,J.H.
EPI_ISL_18393198, EPI_ISL_18393200, EPI_ISL_18393202	Oswaldo Cruz Foundation, Instituto Carlos Chagas	Oswaldo Cruz Foundation, Instituto Carlos Chagas	Sampaio,M., Rosario,M., Fritsch,H., Xavier,J., Mello,A., Pereira,F., Giovanetti,M., Siqueira,I. and Graf,T.
EPI_ISL_18458198	NC - Biochemistry	NC - Biochemistry	Alguridi,H.I., Zaki,E.A., Altayb,H.N., Almalki,S.A. and Alzahrani,A.
EPI_ISL_18474264, EPI_ISL_18474265	Manipal Centre for Virus Research, Manipal Academy of Higher Education	Manipal Centre for Virus Research, Manipal Academy of Higher Education	Kavitha,K., Ujwal,S., Sudheesh,N., Piya,P. and Anitha,J.
EPI_ISL_18474282, EPI_ISL_18474283, EPI_ISL_18474284, EPI_ISL_18474285, EPI_ISL_18474286, EPI_ISL_18474287, EPI_ISL_18474288, EPI_ISL_18474289, EPI_ISL_18474290, EPI_ISL_18474291, EPI_ISL_18474292, EPI_ISL_18474293, EPI_ISL_18474294, EPI_ISL_18474295, EPI_ISL_18474296, EPI_ISL_18474297, EPI_ISL_18474298, EPI_ISL_18474299, EPI_ISL_18474300, EPI_ISL_18474301, EPI_ISL_18474302, EPI_ISL_18474303, EPI_ISL_18474304, EPI_ISL_18474542, EPI_ISL_18480979, EPI_ISL_18480980, EPI_ISL_18480981, EPI_ISL_18480982, EPI_ISL_18480983, EPI_ISL_18480984			
see above	Undisclosed Laboratory in Brazil for the Health Emergency Information and Risk Assessment (HIM) Unit of PAHO	Undisclosed Laboratory in Brazil for the Health Emergency Information and Risk Assessment (HIM) Unit of PAHO	Cortinas,M.N., Fonseca,V., Giovanetti,M., Martin,R.S., Lima,M., Burgueno,A., Guimaraes,N., Morel,M.N., Bormida,V.B., Castro,E., Alcantara,L.C.J. and Chiparelli,H.
EPI_ISL_18490191, EPI_ISL_18490192, EPI_ISL_18490193, EPI_ISL_18490194, EPI_ISL_18490195, EPI_ISL_18490196, EPI_ISL_18490197, EPI_ISL_18490198, EPI_ISL_18490199, EPI_ISL_18490200, EPI_ISL_18490201, EPI_ISL_18490202, EPI_ISL_18490203, EPI_ISL_18490204, EPI_ISL_18490205, EPI_ISL_18490206, EPI_ISL_18490207, EPI_ISL_18490208, EPI_ISL_18490209, EPI_ISL_18490210, EPI_ISL_18490211, EPI_ISL_18490212, EPI_ISL_18490213, EPI_ISL_18490214, EPI_ISL_18490215, EPI_ISL_18490216, EPI_ISL_18490217, EPI_ISL_18490218, EPI_ISL_18490219, EPI_ISL_18490220, EPI_ISL_18490221, EPI_ISL_18490222, EPI_ISL_18490223, EPI_ISL_18490224, EPI_ISL_18490225, EPI_ISL_18490226, EPI_ISL_18490227, EPI_ISL_18490228, EPI_ISL_18490229, EPI_ISL_18490230, EPI_ISL_18490231, EPI_ISL_18490232, EPI_ISL_18490233, EPI_ISL_18490234, EPI_ISL_18490235, EPI_ISL_18490236, EPI_ISL_18490237, EPI_ISL_18490238, EPI_ISL_18490239, EPI_ISL_18490240, EPI_ISL_18490241, EPI_ISL_18490242, EPI_ISL_18490243, EPI_ISL_18490244, EPI_ISL_18490245			
see above	Undisclosed Laboratory in Brazil for the Health Emergency Information and Risk Assessment (HIM) Unit of PAHO	Undisclosed Laboratory in Brazil for the Health Emergency Information and Risk Assessment (HIM) Unit of PAHO	Fabbri,C., Giovanetti,M., Fonseca,V., Franco,L., Mendez,J., Alcantara,L.C.J. and Morales,M.A.
EPI_ISL_18497709, EPI_ISL_18497710, EPI_ISL_18497711, EPI_ISL_18497712, EPI_ISL_18497713, EPI_ISL_18497714, EPI_ISL_18497715, EPI_ISL_18497716, EPI_ISL_18497717, EPI_ISL_18497718, EPI_ISL_18497719, EPI_ISL_18497720, EPI_ISL_18497721, EPI_ISL_18497722, EPI_ISL_18497723, EPI_ISL_18497724, EPI_ISL_18497725, EPI_ISL_18497726, EPI_ISL_18497727, EPI_ISL_18497728, EPI_ISL_18497729, EPI_ISL_18497730, EPI_ISL_18497731			
see above	Central Public Health Laboratory, State Health Surveillance Center of the Rio Grande do Sul State Health Department (LACEN/CEVS/SES-RS)	State Center for Health Surveillance of the Secretary of Health of the State of Rio Grande do Sul (CDCT/CEVS/SES-RS)	Fernanda Godinho, Tatiana Schäffer Gregianini, Amanda Pellenz Ruivo, Júlio Augusto Schroder, Viviane Horn, Franciellen Machado dos Santos, Regina Bones Barcellos, Fernanda Letícia Martiny, Érica Bortoli Möllmann, Gabriel da Luz Wallau, Richard Steiner Salvato
EPI_ISL_18498014, EPI_ISL_18498015, EPI_ISL_18498016, EPI_ISL_18498017, EPI_ISL_18498019, EPI_ISL_18498021, EPI_ISL_18498022, EPI_ISL_18498026, EPI_ISL_18498028, EPI_ISL_18498029, EPI_ISL_18498030, EPI_ISL_18498031, EPI_ISL_18498032, EPI_ISL_18498034, EPI_ISL_18498035, EPI_ISL_18498036, EPI_ISL_18498037, EPI_ISL_18498038, EPI_ISL_18498039			

see above	Kedougou Health Centre	IRESSEF and CERI, Stellenbosch University	Padane A, Tegally H, Ramphal Y, Ndiaye Seyni, Sarr M, Diop M M, Diedhiou K C, Mboup A, Diouf ND, Souaré A, Diagne ND, Aza-Gnandji M, Dabo NA, Dia YA, Diaw NA, Leye N, Diaw PA, Ahouidi A, Cissé B , Diallo AS, Diop O, Diallo AA, Ndoye S, Sanko T J, Baxter, C, Wilkinson E, San J, Tshabuila D, Naidoo Y, Pillay S, Lessells R, Cissé K, Leye A , Mbaye K A , Dramane K , Tinto B, Ouedraogo AS, Lou renço J, Giovanetti M,Giandhari J, de Oliveira T , Mboup S
EPI_ISL_18539020	Guangzhou Center for Disease Control and Prevention, Department of Virology and Immunology	Guangzhou Center for Disease Control and Prevention, Department of Virology and Immunology	Su,W., Lu,W. and Cao,Y.
EPI_ISL_18553672, EPI_ISL_18553673	LACEN de Sergipe	Instituto Adolfo Lutz - Strategic Laboratory	Claudio Tavares Sacchi, Claudia Regina Gonçalves, Karoline Rodrigues Campos, Marlon Benedito N. Santos, Gabriela Vasconcelos Brito Bezerra
EPI_ISL_18553674, EPI_ISL_18553675, EPI_ISL_18553676, EPI_ISL_18553677, EPI_ISL_18553678, EPI_ISL_18553679, EPI_ISL_18553680, EPI_ISL_18553681, EPI_ISL_18553682, EPI_ISL_18553683, EPI_ISL_18553684, EPI_ISL_18553685, EPI_ISL_18553686, EPI_ISL_18553687, EPI_ISL_18553688, EPI_ISL_18553689			
see above	LACEN de Tocantins	Instituto Adolfo Lutz - Strategic Laboratory	Claudio Tavares Sacchi, Claudia Regina Gonçalves, Karoline Rodrigues Campos, Marlon Benedito N. Santos, Franciano Dias Pereira Cardoso
EPI_ISL_18553690, EPI_ISL_18553691, EPI_ISL_18553692, EPI_ISL_18553693, EPI_ISL_18553694, EPI_ISL_18553695, EPI_ISL_18553696, EPI_ISL_18553697, EPI_ISL_18553698, EPI_ISL_18553699	Instituto Adolfo Lutz - NDTV	Instituto Adolfo Lutz - Strategic Laboratory	Claudio Tavares Sacchi, Claudia Regina Gonçalves, Karoline Rodrigues Campos, Marlon Benedito N. Santos
EPI_ISL_18554084	Guangzhou Center for Disease Control and Prevention, Department of Virology and Immunology	Guangzhou Center for Disease Control and Prevention, Department of Virology and Immunology	Su,W., Lu,W. and Cao,Y.
EPI_ISL_18554085, EPI_ISL_18554086, EPI_ISL_18554087, EPI_ISL_18554088, EPI_ISL_18554089, EPI_ISL_18554090	Institute of Virology, Wenzhou University	Institute of Virology, Wenzhou University	Xiao,P.
EPI_ISL_18554091, EPI_ISL_18554092, EPI_ISL_18554093, EPI_ISL_18554094, EPI_ISL_18554095, EPI_ISL_18554096, EPI_ISL_18554097, EPI_ISL_18554098, EPI_ISL_18554099, EPI_ISL_18554100, EPI_ISL_18554101, EPI_ISL_18554102, EPI_ISL_18554103, EPI_ISL_18554104, EPI_ISL_18554105, EPI_ISL_18554106, EPI_ISL_18554107, EPI_ISL_18554108, EPI_ISL_18554109			
see above	Zoonosis, Universidad Autonoma de Yucatan	Zoonosis, Universidad Autonoma de Yucatan	Yeh-Gorocica,A.B., Rivero Juarez,A. and Torres-Castro,M.A.
EPI_ISL_18679895, EPI_ISL_18679896, EPI_ISL_18679897, EPI_ISL_18679898, EPI_ISL_18679899, EPI_ISL_18679900, EPI_ISL_18679901, EPI_ISL_18679902, EPI_ISL_18679903, EPI_ISL_18679904	LACEN de Tocantis	Instituto Adolfo Lutz, Rapid Response Center, Strategic Laboratory	Claudio Tavares Sacchi, Claudia Regina Gonçalves, Karoline Rodrigues Campos, Marlon Benedito Nascimento Santos, Franciano Dias Pereira Cardoso
EPI_ISL_18679905, EPI_ISL_18679906, EPI_ISL_18679907, EPI_ISL_18679908, EPI_ISL_18679909, EPI_ISL_18679910, EPI_ISL_18679911	NDTV	Instituto Adolfo Lutz, Rapid Response Center, Strategic Laboratory	Claudio Tavares Sacchi, Claudia Regina Gonçalves, Karoline Rodrigues Campos, Marlon Benedito Nascimento Santos, Juliana Silva Nogueira
EPI_ISL_18699308	UPA Leste - Luis Atílio Losi Viana	HemocentroRP-FMRP	Debora Glenda Lima de La-Roque, Elaine Vieira Santos, Mariane Evaristo, Aparecida Yulie Yamamoto, Dimas Tadeu Covas, Luiz Carlos Júnior Alcântara, Simone Kashima
EPI_ISL_18699309, EPI_ISL_18699310	UPA Oeste Prof Dr João José Carneiro	HemocentroRP-FMRP	Debora Glenda Lima de La-Roque, Elaine Vieira Santos, Mariane Evaristo, Aparecida Yulie Yamamoto, Dimas Tadeu Covas, Luiz Carlos Júnior Alcântara, Simone Kashima
EPI_ISL_18699311	UPA Leste - Luis Atílio Losi Viana	HemocentroRP-FMRP	Debora Glenda Lima de La-Roque, Elaine Vieira Santos, Mariane Evaristo, Aparecida Yulie Yamamoto, Dimas Tadeu Covas, Luiz Carlos Júnior Alcântara, Simone Kashima
EPI_ISL_18699312	UBS Ribeirão Verde	HemocentroRP-FMRP	Debora Glenda Lima de La-Roque, Elaine Vieira Santos, Mariane Evaristo, Aparecida Yulie Yamamoto, Dimas Tadeu Covas, Luiz Carlos Júnior Alcântara, Simone Kashima
EPI_ISL_18699313	UPA Leste - Luis Atílio Losi Viana	HemocentroRP-FMRP	Debora Glenda Lima de La-Roque, Elaine Vieira Santos, Mariane Evaristo, Aparecida Yulie Yamamoto, Dimas Tadeu Covas, Luiz Carlos Júnior Alcântara, Simone Kashima
EPI_ISL_18699314	UPA Oeste Prof Dr João José Carneiro	HemocentroRP-FMRP	Debora Glenda Lima de La-Roque, Elaine Vieira Santos, Mariane Evaristo, Aparecida Yulie Yamamoto, Dimas Tadeu Covas, Luiz Carlos Júnior Alcântara, Simone Kashima
EPI_ISL_18699315	UPA Leste - Luis Atílio Losi Viana	HemocentroRP-FMRP	Debora Glenda Lima de La-Roque, Elaine Vieira Santos, Mariane Evaristo, Aparecida Yulie Yamamoto, Dimas Tadeu Covas, Luiz Carlos Júnior Alcântara, Simone Kashima
EPI_ISL_18699316	UPA Oeste Prof Dr João José Carneiro	HemocentroRP-FMRP	Debora Glenda Lima de La-Roque, Elaine Vieira Santos, Mariane Evaristo, Aparecida Yulie Yamamoto, Dimas Tadeu Covas, Luiz Carlos Júnior Alcântara, Simone Kashima
EPI_ISL_18699317	UPA Norte - Nelson Mandela	HemocentroRP-FMRP	Debora Glenda Lima de La-Roque, Elaine Vieira Santos, Mariane Evaristo, Aparecida Yulie Yamamoto, Dimas Tadeu Covas, Luiz Carlos Júnior Alcântara, Simone Kashima
EPI_ISL_18699318	UPA Oeste Prof Dr João José Carneiro	HemocentroRP-FMRP	Debora Glenda Lima de La-Roque, Elaine Vieira Santos, Mariane Evaristo, Aparecida Yulie Yamamoto, Dimas Tadeu Covas, Luiz Carlos Júnior Alcântara, Simone Kashima
EPI_ISL_18699319, EPI_ISL_18699320, EPI_ISL_18699321	Laboratório de Virologia - Hospital das Clínicas FMRP	HemocentroRP-FMRP	Debora Glenda Lima de La-Roque, Elaine Vieira Santos, Mariane Evaristo, Aparecida Yulie Yamamoto, Dimas Tadeu Covas, Luiz Carlos Júnior Alcântara, Simone Kashima
EPI_ISL_18704529, EPI_ISL_18704530, EPI_ISL_18704531, EPI_ISL_18704532, EPI_ISL_18704533, EPI_ISL_18704534, EPI_ISL_18704535, EPI_ISL_18704536, EPI_ISL_18704537, EPI_ISL_18704538, EPI_ISL_18704539, EPI_ISL_18704540, EPI_ISL_18704541, EPI_ISL_18704542, EPI_ISL_18704543, EPI_ISL_18704544, EPI_ISL_18704545, EPI_ISL_18704546, EPI_ISL_18704547, EPI_ISL_18704548, EPI_ISL_18704549, EPI_ISL_18704550, EPI_ISL_18704551, EPI_ISL_18704552, EPI_ISL_18704553, EPI_ISL_18704554, EPI_ISL_18704555, EPI_ISL_18704556, EPI_ISL_18704557, EPI_ISL_18704558, EPI_ISL_18704559, EPI_ISL_18704560, EPI_ISL_18704561, EPI_ISL_18704562, EPI_ISL_18704563, EPI_ISL_18704564, EPI_ISL_18704565, EPI_ISL_18704566, EPI_ISL_18704567, EPI_ISL_18704568, EPI_ISL_18704569, EPI_ISL_18704570, EPI_ISL_18704571, EPI_ISL_18715402, EPI_ISL_18715403, EPI_ISL_18715405, EPI_ISL_18715406, EPI_ISL_18715407, EPI_ISL_18715408, EPI_ISL_18715409, EPI_ISL_18715410, EPI_ISL_18715411, EPI_ISL_18715412, EPI_ISL_18715413, EPI_ISL_18715414, EPI_ISL_18715415, EPI_ISL_18715416, EPI_ISL_18715417, EPI_ISL_18715419, EPI_ISL_18715420, EPI_ISL_18715422, EPI_ISL_18715423, EPI_ISL_18715425, EPI_ISL_18715426, EPI_ISL_18715428, EPI_ISL_18715430, EPI_ISL_18715431, EPI_ISL_18715432, EPI_ISL_18715433, EPI_ISL_18715434, EPI_ISL_18715435, EPI_ISL_18715436, EPI_ISL_18715437, EPI_ISL_18715438, EPI_ISL_18715439, EPI_ISL_18715440, EPI_ISL_18715441, EPI_ISL_18715442, EPI_ISL_18715444, EPI_ISL_18715445, EPI_ISL_18715447, EPI_ISL_18715448, EPI_ISL_18715450, EPI_ISL_18715451, EPI_ISL_18715453			
see above	Evandro Chagas Institute, Department of Arbovirology and Hemorrhagic Fevers	Evandro Chagas Institute, Department of Arbovirology and Hemorrhagic Fevers	Santos,A.C.M., Silva,S.P. and Cruz,A.C.R.
EPI_ISL_18855720, EPI_ISL_18855721	University of Malaya, Faculty of Medicine, Department of Medical Microbiology	University of Malaya, Faculty of Medicine, Department of Medical Microbiology	Chua,C.L., Chan,Y.F. and Sam,I.C.
EPI_ISL_18870910, EPI_ISL_18870911, EPI_ISL_18870912, EPI_ISL_18870913, EPI_ISL_18870914, EPI_ISL_18870915, EPI_ISL_18870916, EPI_ISL_18870917, EPI_ISL_18870918, EPI_ISL_18870919, EPI_ISL_18870920, EPI_ISL_18870921, EPI_ISL_18870922, EPI_ISL_18870923, EPI_ISL_18870924, EPI_ISL_18870925, EPI_ISL_18870926, EPI_ISL_18870927, EPI_ISL_18870928, EPI_ISL_18870929, EPI_ISL_18870930, EPI_ISL_18870931, EPI_ISL_18870932, EPI_ISL_18870933, EPI_ISL_18870934, EPI_ISL_18870935, EPI_ISL_18870936			
see above	Nucleo de Doencas de Transmissao Vetorial	Instituto Adolfo Lutz, Rapid Response Center, Strategic Laboratory	Claudio Tavares Sacchi, Claudia Regina Gonçalves, Karoline Rodrigues Campos, Marlon Benedito Nascimento Santos, Juliana Silva Nogueira
EPI_ISL_18883605, EPI_ISL_18883606, EPI_ISL_18883607, EPI_ISL_18883608, EPI_ISL_18883609, EPI_ISL_18883610, EPI_ISL_18883611, EPI_ISL_18883612, EPI_ISL_18883613, EPI_ISL_18883614, EPI_ISL_18883615, EPI_ISL_18883616, EPI_ISL_18883617, EPI_ISL_18883618, EPI_ISL_18883619, EPI_ISL_18883620, EPI_ISL_18883621, EPI_ISL_18883622, EPI_ISL_18883623, EPI_ISL_18883624			
see above	Laboratório Central de Saúde Pública do Espírito Santo	Laboratório Central de Saúde Pública de Minas Gerais	Talita Émile Ribeiro Adelino, Natália Rocha Guimarães, Felipe Campos de Melo Iani
EPI_ISL_18883625, EPI_ISL_18883626, EPI_ISL_18883627, EPI_ISL_18883628, EPI_ISL_18883629	Laboratório Central de Saúde Pública de Santa Catarina	Laboratório Central de Saúde Pública de Minas Gerais	Talita Émile Ribeiro Adelino, Natália Rocha Guimarães, Felipe Campos de Melo Iani
EPI_ISL_18891551, EPI_ISL_18891552, EPI_ISL_18891553, EPI_ISL_18891554, EPI_ISL_18891555, EPI_ISL_18891557	Institute for Medical Research, National Institutes of Health	Institute for Medical Research, National Institutes of Health	Kalyanasundram,J., Kamel,K.A., Mohd Zawawi,Z., Anasir,M.I., Aroidoss,E.T., Ellan,K., Sari,M.M., Mohd Zain,R. and Thayan,R.
EPI_ISL_18916844, EPI_ISL_18916845	Department of Environmental and Global Health, University of Florida	Department of Environmental and Global Health, University of Florida	Moquin,T.L., Louis,R., Morris,J.G. Jr. and Lednicky,J.A.
EPI_ISL_18928532, EPI_ISL_18928533, EPI_ISL_18928534, EPI_ISL_18928535, EPI_ISL_18928536, EPI_ISL_18928537	Central Laboratory of Public Health of the Espirito Santo - Lacen ES	Central Laboratory of Public Health of the Espirito Santo - Lacen ES	Felipe Donateli Gatti, Gabriela Colombo de Mendonça, Lucas André Silva Bonela, Thiago de Jesus Sousa, Jaqueline Pegoretti Goulart, Rodrigo Ribeiro Rodrigues as General Coordinator
EPI_ISL_18953961, EPI_ISL_18953962, EPI_ISL_18953963, EPI_ISL_18953964, EPI_ISL_18953965, EPI_ISL_18953966, EPI_ISL_18953967, EPI_ISL_18953968	Laboratório Central de Saude Publica do Espirito Santo	Laboratório Central de Saude Publica de Minas Gerais	Talita Émile Ribeiro Adelino, Natália Rocha Guimarães, Luiz Marcelo Ribeiro Tomé, Felipe Campos de Melo Iani
EPI_ISL_18980221, EPI_ISL_18980222, EPI_ISL_18980223, EPI_ISL_18980224, EPI_ISL_18980225, EPI_ISL_18980226, EPI_ISL_18980227, EPI_ISL_18980228, EPI_ISL_18980229, EPI_ISL_18980230, EPI_ISL_18980231, EPI_ISL_18980232, EPI_ISL_18980233, EPI_ISL_18980234, EPI_ISL_18980235, EPI_ISL_18980236, EPI_ISL_18980237, EPI_ISL_18980238, EPI_ISL_18980239, EPI_ISL_18980240, EPI_ISL_18980241, EPI_ISL_18980242, EPI_ISL_18980243, EPI_ISL_18980244, EPI_ISL_18980245, EPI_ISL_18980246, EPI_ISL_18980247, EPI_ISL_18980248, EPI_ISL_18980249, EPI_ISL_18980250, EPI_ISL_18980251, EPI_ISL_18980252, EPI_ISL_18980253, EPI_ISL_18980254, EPI_ISL_18980255, EPI_ISL_18980256, EPI_ISL_18980257, EPI_ISL_18980258, EPI_ISL_18980259, EPI_ISL_18980260, EPI_ISL_18980261, EPI_ISL_18980262, EPI_ISL_18980263, EPI_ISL_18980264, EPI_ISL_18980265, EPI_ISL_18980266, EPI_ISL_18980267, EPI_ISL_18980268, EPI_ISL_18980269, EPI_ISL_18980270, EPI_ISL_18980271, EPI_ISL_18980272, EPI_ISL_18980273, EPI_ISL_18980274, EPI_ISL_18980275, EPI_ISL_18980276, EPI_ISL_18980277, EPI_ISL_18980278, EPI_ISL_18980279, EPI_ISL_18980280, EPI_ISL_18980281, EPI_ISL_18980282, EPI_ISL_18980283, EPI_ISL_18980284, EPI_ISL_18980285, EPI_ISL_18980286, EPI_ISL_18980287, EPI_ISL_18980288, EPI_ISL_18980289, EPI_ISL_18980290, EPI_ISL_18980291, EPI_ISL_18980292, EPI_ISL_18980293, EPI_ISL_18980294, EPI_ISL_18980295, EPI_ISL_18980296, EPI_ISL_18980297, EPI_ISL_18980298, EPI_ISL_18980299, EPI_ISL_18980300			
see above	Institute Hermes Pardini - Fleury Group	Laboratório de Biologia Integrativa	Paula Luize Camargos Fonseca,Augusto César Parreiras de Jesus, Hugo José Alves, Diego Menezes Bonfim, João Victor Rodrigues Dutra, Filipe Romero Rebello Moreira, Cristiane Pinheiro Toscano de B Mendonça, Jéssica Silqueira Hickson Rios, Joice do Prado Silva, Frederico Scott Varella Malta, Isabela Braga-Paz, João Locke Ferreira de Araújo, Jaqueline Silva de Oliveira, Carolina Senra Alves de Souza, Sandra Elisa Barbosa da Silva, Danielle Costa Capistrano Chaves, Renée da Silva Carvalho, Eneida Santos de Oliveira, Rennan Garcias Moreira, Renan Pedra de Souza, Danielle Alves Gomes Zauli, Renato Santana Aguiar
EPI_ISL_18980345, EPI_ISL_18980346, EPI_ISL_18980347	Federal University of Tocantins, Bioinformatics and Biotechnology Laboratory	Federal University of Tocantins, Bioinformatics and Biotechnology Laboratory	Souza,U.J.B., Barreto,H.G., Manuli,E.R., Santos,G.C.A., Sabino,E.C. and Milagres,F.A.P.
EPI_ISL_18992460	Guangzhou Blood Center	Guangzhou Blood Center	Xu,R., Yan,Y., Han,H., Sun,W., Liang,J., Li,B., Zhao,Y., Su,P., Fu,Y., Rong,X. and Wang,L.
EPI_ISL_18992743	Guangzhou Blood Center, Institute of Clinical Blood Transfusion	Guangzhou Blood Center, Institute of Clinical Blood Transfusion	Xu,R., Yan,Y., Han,H., Sun,W., Liang,J., Li,B., Zhao,Y., Su,P., Fu,Y., Rong,X. and Wang,L.
EPI_ISL_19007927, EPI_ISL_19007928, EPI_ISL_19007929, EPI_ISL_19007930, EPI_ISL_19007931, EPI_ISL_19007932, EPI_ISL_19007933, EPI_ISL_19007934, EPI_ISL_19007935, EPI_ISL_19007936, EPI_ISL_19007937, EPI_ISL_19007938, EPI_ISL_19007939, EPI_ISL_19007940, EPI_ISL_19007941, EPI_ISL_19007942, EPI_ISL_19007943, EPI_ISL_19007944, EPI_ISL_19007945, EPI_ISL_19007946, EPI_ISL_19007947, EPI_ISL_19007948, EPI_ISL_19007949, EPI_ISL_19007950, EPI_ISL_19007951, EPI_ISL_19007952, EPI_ISL_19007953, EPI_ISL_19007954, EPI_ISL_19007955, EPI_ISL_19007956, EPI_ISL_19007957, EPI_ISL_19007958, EPI_ISL_19007959, EPI_ISL_19007960, EPI_ISL_19007961, EPI_ISL_19007962, EPI_ISL_19007963, EPI_ISL_19007964, EPI_ISL_19007965, EPI_ISL_19007966, EPI_ISL_19007967, EPI_ISL_19007968, EPI_ISL_19007969, EPI_ISL_19007970, EPI_ISL_19007971, EPI_ISL_19007972, EPI_ISL_19007973, EPI_ISL_19007974, EPI_ISL_19007975, EPI_ISL_19007976, EPI_ISL_19007977			
see above	Microbiology, Immunology, and Molecular Genetics, University of Kentucky	Microbiology, Immunology, and Molecular Genetics, University of Kentucky	de Souza,W.M., Fumagalli,M.J., de Lima,S.T.S., Parise,P.L., Carvalho,D.C.M., Hernandez,C., de Jesus,R., Delafiori,J., Candido,D.S., Carregari,V.C., Muraro,S.P., Souza,G.F., Simoes Mello,L.M., Claro,I.M., Diaz,Y., Kato,R.B., Trentin,L.N., Costa,C.H.S., Maximo,A.C.B.M., Cavalcante,K.F., Fiuza,T.S., Viana,V.A.F., Melo,M.E.L., Ferraz,C.P.M., Silva,D.B., Duarte,L.M.F., Barbosa,P.P., Amorim,M.R., Judice,C.C., Toledo-Teixeira,D.A., Ramundo,M.S., Aguilar,P.V., Araujo,E.L.L., Costa,F.T.M., Cerqueira-Silva,T., Khouri,R., Boaventura,V.S., Figueiredo,L.T.M., Fang,R., Moreno,B., Lopez-Verges,S., Mello,L.P., Skaf,M.S., Catharino,R.R., Granja,F., Martins-de-Souza,D., Plante,J.A., Plante,K.S., Sabino,E.C., Diamond,M.S., Eugenin,E., Proenca-Modena,J.L., Faria,N.R. and Weaver,S.C.
EPI_ISL_19007978, EPI_ISL_19007979, EPI_ISL_19007980, EPI_ISL_19007981, EPI_ISL_19007982, EPI_ISL_19007983, EPI_ISL_19007984, EPI_ISL_19007985, EPI_ISL_19007986, EPI_ISL_19007987, EPI_ISL_19007988, EPI_ISL_19007989, EPI_ISL_19007990, EPI_ISL_19007991, EPI_ISL_19007992, EPI_ISL_19007993, EPI_ISL_19007994, EPI_ISL_19007995, EPI_ISL_19007996, EPI_ISL_19007997, EPI_ISL_19007998			
see above	National Institute of Virology, Dengue Group	National Institute of Virology, Dengue Group	Patil,J.A. and Alagrasu,K.
EPI_ISL_19008004, EPI_ISL_19008005, EPI_ISL_19008006, EPI_ISL_19008007, EPI_ISL_19008008	Microbiology, Immunology, and Molecular Genetics, University of Kentucky	Microbiology, Immunology, and Molecular Genetics, University of Kentucky	de Souza,W.M., Fumagalli,M.J., de Lima,S.T.S., Parise,P.L., Carvalho,D.C.M., Hernandez,C., de Jesus,R., Delafiori,J., Candido,D.S., Carregari,V.C., Muraro,S.P., Souza,G.F., Simoes Mello,L.M., Claro,I.M., Diaz,Y., Kato,R.B., Trentin,L.N., Costa,C.H.S., Maximo,A.C.B.M., Cavalcante,K.F., Fiuza,T.S., Viana,V.A.F., Melo,M.E.L., Ferraz,C.P.M., Silva,D.B., Duarte,L.M.F., Barbosa,P.P., Amorim,M.R., Judice,C.C., Toledo-Teixeira,D.A., Ramundo,M.S., Aguilar,P.V., Araujo,E.L.L., Costa,F.T.M., Cerqueira-Silva,T., Khouri,R., Boaventura,V.S., Figueiredo,L.T.M., Fang,R., Moreno,B., Lopez-Verges,S., Mello,L.P., Skaf,M.S., Catharino,R.R., Granja,F., Martins-de-Souza,D., Plante,J.A., Plante,K.S., Sabino,E.C., Diamond,M.S., Eugenin,E., Proenca-

			Modena,J.L., Faria,N.R. and Weaver,S.C.
EPI_ISL_19035650, EPI_ISL_19035651, EPI_ISL_19035652, EPI_ISL_19035653, EPI_ISL_19035654, EPI_ISL_19035655, EPI_ISL_19035656, EPI_ISL_19035657, EPI_ISL_19035658, EPI_ISL_19035659, EPI_ISL_19035660, EPI_ISL_19035661, EPI_ISL_19035662, EPI_ISL_19035663, EPI_ISL_19035664, EPI_ISL_19035665, EPI_ISL_19035666			
see above	Department of Medical Research, Virology Research Division	Department of Medical Research, Virology Research Division	Ngwe Tun,M.M., Kyaw,A.K., Nabeshima,T., Dumre,S.P., Soe,A.M., Nwe,K.M., Myaing,S.S., Lwin,E.P., Win,Y.T., Inoue,S., Takamatsu,Y., Urano,T., Thu,H.M., Thant,K.Z., Htun,Z.T. and Morita,K.
EPI_ISL_19035680	LACEN-RJ - Laboratório Central de Saúde Pública Noel Nutels	Laboratório de Arbovírus e Vírus Hemorrágicos - LARBOH/IOC/FIOCRUZ	Carla de Oliveira, Carolina Cardoso dos Santos, Cintia Damasceno, Fernanda de Bruycker-Nogueira, Andréa Cony, Maria Carmelita dos Santos Vaz, Felipe Gomes Naveca and Ana Maria Bispo de Filippis
EPI_ISL_19053771	Institute of Life Sciences, Department of Infectious Disease Biology	Institute of Life Sciences, Department of Infectious Disease Biology	Laha,E., Biswas,V.K., Jena,D., Singh,S., Raghav,S.K., Pattnaik,A.K. and Chattopadhyay,S.
EPI_ISL_19053772	University of Washington, Virology, Greninger Lab	University of Washington, Virology, Greninger Lab	Meephaendee,L., Posanacharoen,A., Naemkhunthot,S., Rojanawiwat,A., Roychoudhury,P., Sobolik,E.B., Sereewit,J. and Greninger,A.L.
EPI_ISL_19069872, EPI_ISL_19069873, EPI_ISL_19069874, EPI_ISL_19069875	University of Washington, Virology, Greninger Lab	University of Washington, Virology, Greninger Lab	Dangsagul,W., Puthavathana,P., Rojanawiwat,A., Meephaendee,L., Posanacharoen,A., Sereewit,J., Sobolik,E.B., Roychoudhury,P. and Greninger,A.L.
EPI_ISL_19075520, EPI_ISL_19075521, EPI_ISL_19075522	Laboratório Central de Saude Publica do Espirito Santo	Laboratório Central de Saude Publica de Minas Gerais	Talita Émile Ribeiro Adelino, Natália Rocha Guimarães, Luiz Marcelo Ribeiro Tomé, Felipe Campos de Melo Iani
EPI_ISL_19085665	Guangzhou Center for Disease Control and Prevention, Department of Virology	Guangzhou Center for Disease Control and Prevention, Department of Virology	Su,W., Lu,W., Liu,P. and Cao,Y.
EPI_ISL_19096373	Laboratório de Comportamento de Insetos e Laboratório de Biologia Molecular do Instituto Federal do Norte de Minas Gerais campus Salinas	Laboratório Central de Saúde Publica do Estado de Minas Gerais (Lacen-MG)	Felipe Campos de Melo Iani, Natália Rocha Guimarães.
EPI_ISL_19096381, EPI_ISL_19096382, EPI_ISL_19096383, EPI_ISL_19096384, EPI_ISL_19096385, EPI_ISL_19096386, EPI_ISL_19096387, EPI_ISL_19096388, EPI_ISL_19096389, EPI_ISL_19096390, EPI_ISL_19096391, EPI_ISL_19096392, EPI_ISL_19096393, EPI_ISL_19096394, EPI_ISL_19096395, EPI_ISL_19096396, EPI_ISL_19096397, EPI_ISL_19096398, EPI_ISL_19096399, EPI_ISL_19096400, EPI_ISL_19096401, EPI_ISL_19096402			
see above	Central Laboratory of Public Health of the Espirito Santo - Lacen ES	Central Laboratory of Public Health of the Espirito Santo - Lacen ES	Gabriela Colombo de Mendonça, Lucas André Silva Bonela, Thiago de Jesus Sousa, Jaqueline Pegoretti Goulart, Rodrigo Ribeiro Rodrigues as General Coordinator
EPI_ISL_19116393	NDTV	Instituto Adolfo Lutz, Rapid Response Center, Strategic Laboratory	Claudio Tavares Sacchi, Karoline Rodrigues Campos, Marlon Benedito Nascimento Santos, Juliana Silva Nogueira
EPI_ISL_19116394, EPI_ISL_19116395, EPI_ISL_19116396, EPI_ISL_19116397, EPI_ISL_19116398, EPI_ISL_19116399, EPI_ISL_19116400, EPI_ISL_19116401, EPI_ISL_19116402, EPI_ISL_19116403, EPI_ISL_19116404, EPI_ISL_19116405, EPI_ISL_19116406, EPI_ISL_19116407, EPI_ISL_19116408, EPI_ISL_19116409, EPI_ISL_19116410, EPI_ISL_19116411, EPI_ISL_19116412, EPI_ISL_19116413, EPI_ISL_19116414, EPI_ISL_19116415, EPI_ISL_19116416, EPI_ISL_19116417, EPI_ISL_19116418, EPI_ISL_19116419, EPI_ISL_19116420			
see above	LACEN do Mato Grosso do Sul	Instituto Adolfo Lutz, Rapid Response Center, Strategic Laboratory	Claudio Tavares Sacchi, Karoline Rodrigues Campos, Marlon Benedito Nascimento Santos, Suzana Teruya Hiane
EPI_ISL_19116421, EPI_ISL_19116422, EPI_ISL_19116423, EPI_ISL_19116424, EPI_ISL_19116425, EPI_ISL_19116426, EPI_ISL_19116427, EPI_ISL_19116428, EPI_ISL_19116429	LACEN de Tocantins	Instituto Adolfo Lutz, Rapid Response Center, Strategic Laboratory	Claudio Tavares Sacchi, Karoline Rodrigues Campos, Marlon Benedito Nascimento Santos, Franciano Dias Pereira Cardoso
EPI_ISL_19131640, EPI_ISL_19131641	Central Laboratory of Public Health of the Espirito Santo - Lacen ES	Central Laboratory of Public Health of the Espirito Santo - Lacen ES	Felipe Donateli Gatti, Gabriela Colombo de Mendonça, Lucas André Silva Bonela, Thiago de Jesus Sousa, Jaqueline Pegoretti Goulart, Rodrigo Ribeiro Rodrigues as General Coordinator
