# Supplementary figures and images for: CavitOmiX Drug Discovery: Engineering Antivirals with Enhanced Spectrum and Reduced Side Effects for Arboviral Diseases
Source: Viruses. 2024 Jul 24;16(8):1186. doi: 10.3390/v16081186 (PMC11360613; doi:10.3390/v16081186)

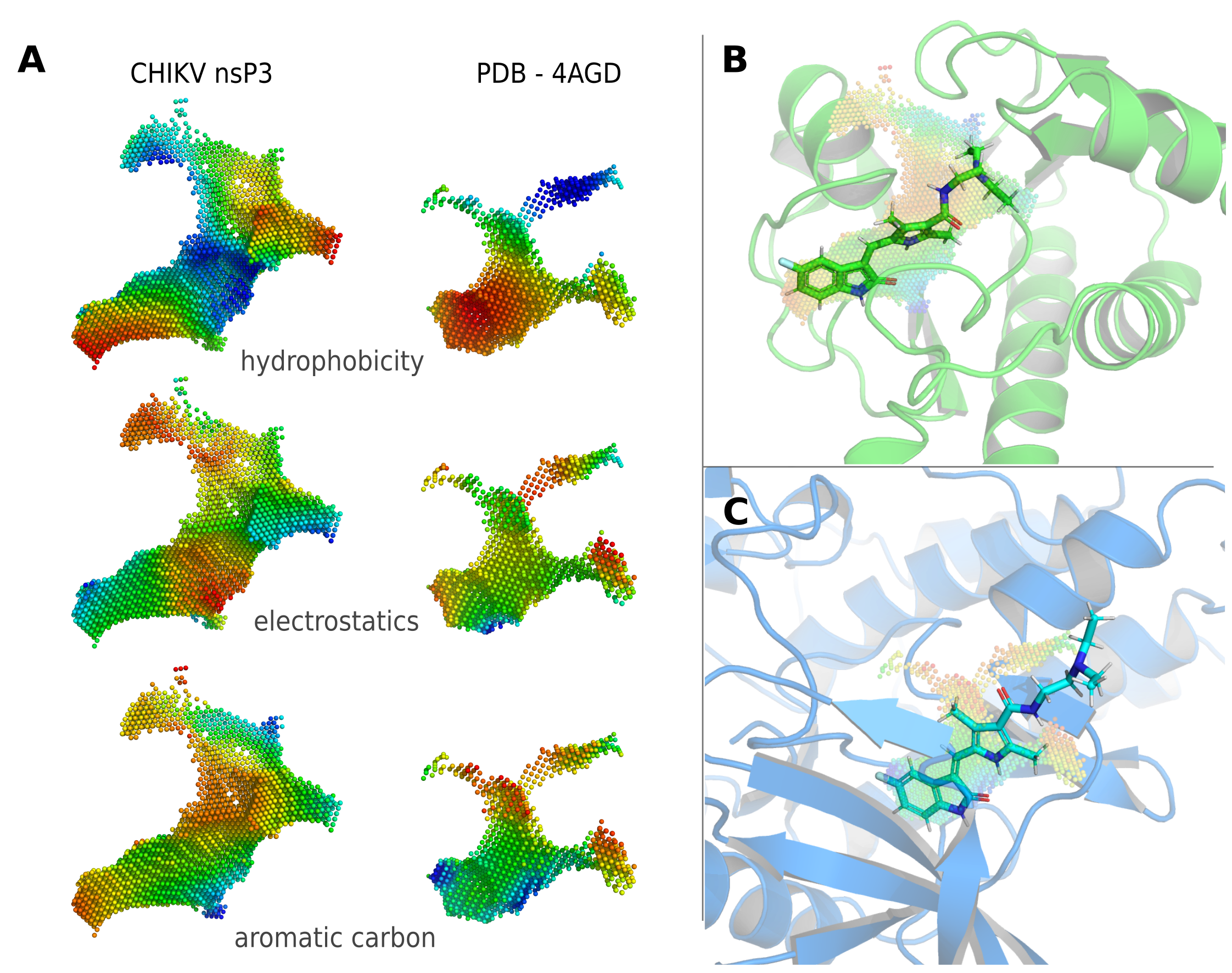

Supplement: Supplementary file 1 [file viruses-16-01186-s001.zip › FigS1.png]

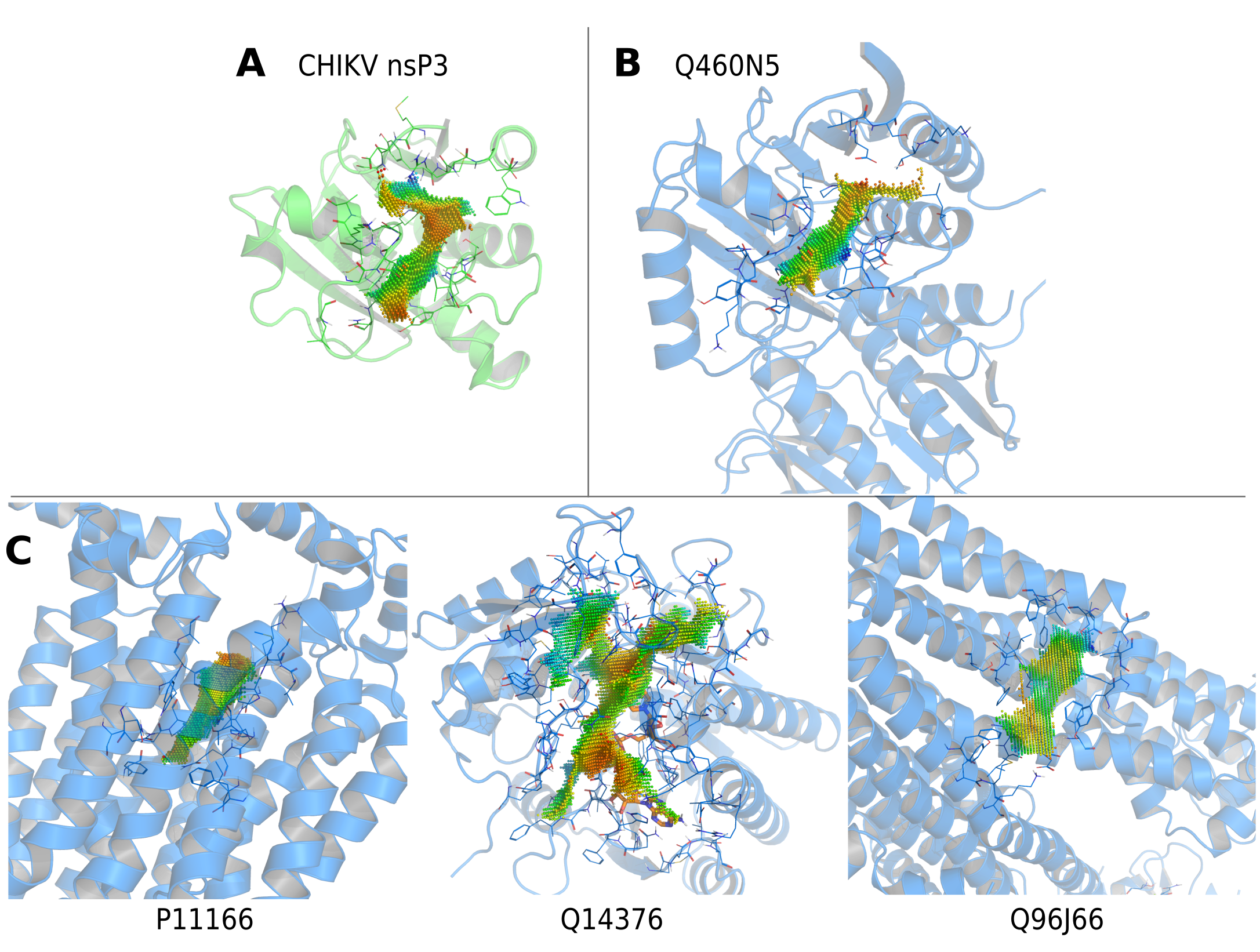

Supplement: Supplementary file 1 [file viruses-16-01186-s001.zip › FigS2.png]

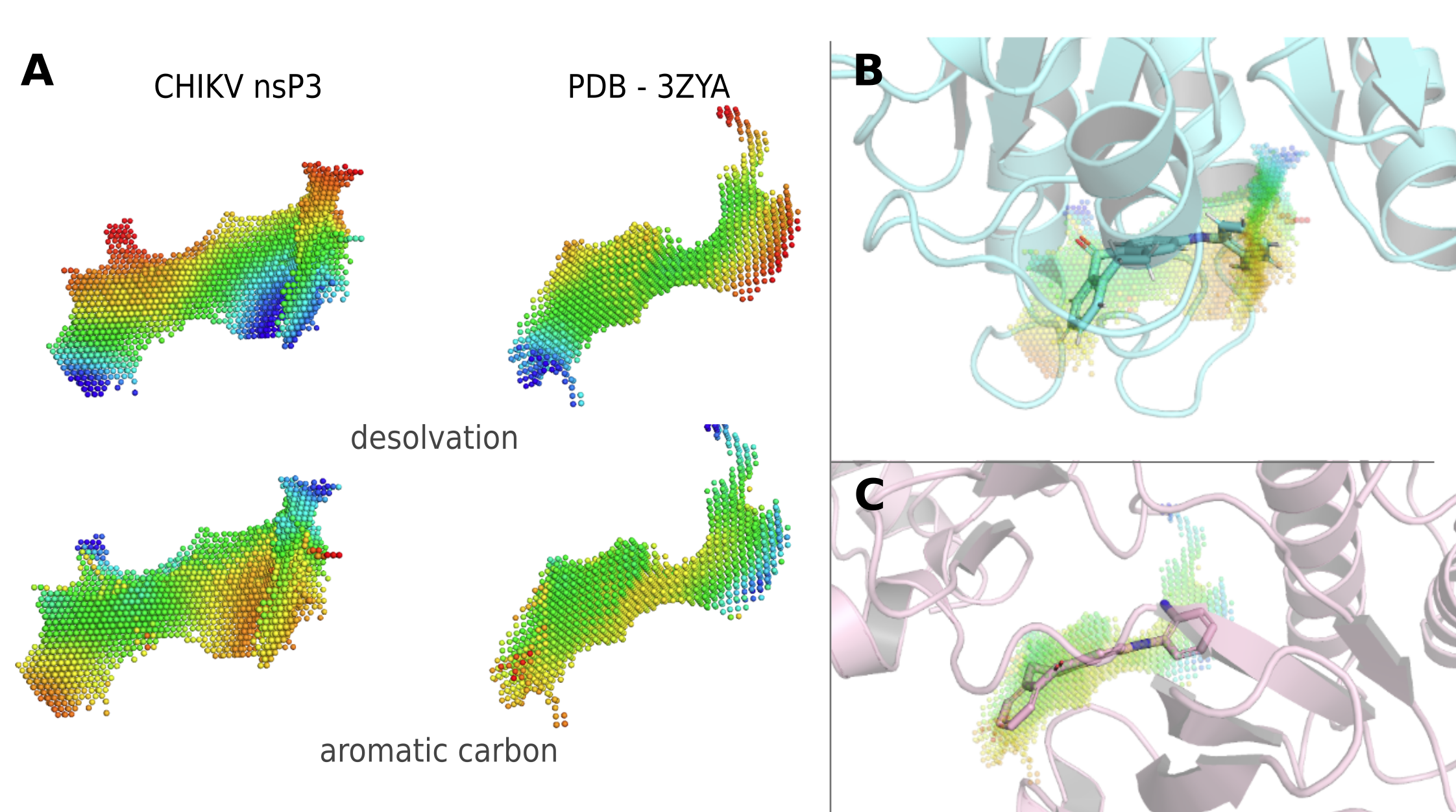

Supplement: Supplementary file 1 [file viruses-16-01186-s001.zip › FigS3.png]
